# Associations between depressive symptoms, socio-economic factors, traumatic exposure and recent intimate partner violence experiences among women in Zimbabwe: a cross-sectional study

**DOI:** 10.1186/s12905-022-01796-w

**Published:** 2022-06-22

**Authors:** Mercilene Machisa, Simukai Shamu

**Affiliations:** 1 South African Medical Research Council Gender and Health Research Unit, 1 Soutpansberg Road, Private Bag X385, Pretoria, 0001 South Africa; 2 School of Public Health, Faculty of Health Sciences, University of Witwatersrand, Johannesburg, South Africa; 3 Health Systems Strengthening Division, Foundation for Professional Development, Pretoria, South Africa

**Keywords:** Depression, Intimate partner violence, Childhood trauma, Social support, Food insecurity, Women

## Abstract

**Background:**

Population-based research on the cumulative effects of socio-economic conditions and trauma exposures, particularly women’s experiences of intimate partner violence (IPV) on their mental health in Zimbabwe, has been limited.

**Aim:**

Our study aimed to determine the associations between depressive symptoms and socio-economic factors, IPV, and traumatic exposures among a nationally representative sample of women from Zimbabwe.

**Methods:**

Data was collected from 2905 women who volunteered to participate in a survey that had a multi-stage random sampling design. Depression was measured using the Centre for Epidemiologic Studies Depression Scale (CESD). Traumatic exposures included childhood trauma, life events, and experiences of IPV in the past year. We compared mean depression scores for different categories of variables, conducted linear regression modelling to investigate the bivariate and multivariate associations between variables and depressive symptoms’ outcomes, and applied Structural Equation Modelling (SEM) to investigate the inter-relationships between variables and depressive symptoms’ outcomes.

**Results:**

Fifteen percent of women self-reported depressive symptoms (CESD score ≥ 21). Higher depressive symptomatology was associated with lower socio-economic status, experiencing IPV, history of childhood and other traumatic events, experiencing non-partner rape, and HIV positive status. Women who could find money in an emergency and sought informal or professional emotional support were less at risk of severe depressive symptoms. Conversely, seeking informal and formal social support was positively associated with more severe depressive symptoms.

**Conclusion:**

This study contributes evidence showing that economic hardship, exposure to traumas including IPV, living with HIV, and low social support have a cumulative negative toll on mental health among Zimbabwean women from the general population. Programmes and services that respond to the mental ill-health effects reported by Zimbabwean women and prevention interventions that tackle the multiple risk factors for depression that we have identified must be prioritised.

**Supplementary Information:**

The online version contains supplementary material available at 10.1186/s12905-022-01796-w.

## Background

Depression is a leading contributor to global morbidity and disability, warranting its ranking as “the most disabling mental disorder” worldwide [1]. About 322 million people (4.4% of the global population) were estimated to have depression in 2015, and its prevalence was markedly higher in developing than developed countries [1, 2]. The prevalence of depression was highest in African studies (5.4%) compared to the other regions of the world [2]. In all regions of the world, women are disproportionately affected, more vulnerable, and report a higher prevalence of depression compared to men [3]. Most research conducted in Zimbabwe has been done in primary health care settings, and about 25% have reported depressive symptoms [4, 5]. Studies have shown high vulnerability to depression among women, especially during the postpartum period [5–7] and among people living with HIV [8, 9].

Socio-economic conditions are well-established risk factors for depression, alcohol consumption, dependence, and abuse [10]. Hence, people living in less-developed nations or those living in poorer conditions are more vulnerable to mental ill-health [10–14]. Long-term exposure to stress emanating from the worries and uncertainties of living in poverty and volatile circumstances negatively affects mental health [14]. Having lower educational attainment, being unemployed, receiving low wages, living in poor housing or overcrowded settlements, living in communities that have high incidence of community violence or having food insecurity, may exacerbate life stress and increases vulnerability to depression, anxiety, alcohol abuse and other comorbid mental disorders [10, 12–18]. Particularly, the uncertainties presented by socio-political or environmental factors such as conflict, forced migration, economic recession, and natural disasters also threaten mental health [15].

Other factors that may mediate the pathways from poverty to mental ill-health include physical illness or disability. Being physically ill or disabled is associated with loss of income and livelihood uncertainties that affect the socio-economic status and may trigger depressive symptoms [15]. Poverty and poor living conditions also negatively impact childhood development and mental health [14]. In addition, harsh early environments and childhood trauma are associated with biological changes in the brain structure. These include the altered sensitivity of receptors, reduction in the hippocampal volume, or physical alteration of the hypothalamic–pituitary–adrenal (HPA) axis, which exacerbates the brain’s vulnerability to future psychopathology such as mood and anxiety disorders [19, 20].

Depression, alcohol abuse, and comorbid disorders are among the established consequences of traumatic exposures, including intimate partner violence (IPV) and non-partner rape experiences reported by adult women [21–26]. Depression is also a risk factor for women’s IPV and non-partner rape re-victimisation [21, 27–29]. Depression and alcohol abuse in adulthood are also among the long-term effects and disorders associated with omen’s experiences of childhood abuse and other traumatic life exposures [30–36]. Moreover, histories of childhood abuse are associated with elevated risk, higher frequency and severity of IPV, and non-partner rape experiences among women. These relationships may be mediated by depression and comorbid disorders [21–23, 27, 30–32, 37–39]. The effects of multiple trauma exposures include the interaction of post-traumatic stress disorder (PTSD)’s emotional numbing symptoms and the affective symptoms of depression. Emotional numbing symptoms lead to suppressed emotional responsiveness, internalising problems, and impede trauma-exposed women’s capability to detect or respond to actual risk [40–43]. The affective symptoms of depression reduce the cognitive and affective capacity required to detect potential abusers, physical IPV triggers, or make decisions to avoid risk [41, 44]. Features of depression such as low energy and motivation, feelings of guilt, helplessness, and hopelessness may also impede women’s resolve to leave violent relationships or avoid dangerous situations [41, 44].

The prevalence of depression and comorbid disorders are higher in communities that have greater social inequality, lower social cohesion and where its inhabitants are likely to have lower social support [12, 13, 15]. Seeking emotional, instrumental, or informational support from a supportive and sympathetic informal network have been found to enhance victims’ coping skills and mitigate the adverse mental health outcomes of IPV, including depressive symptoms [45–55]. Other than mitigating the negative impacts of abuse, having higher social support reduces women’s risk for IPV experiences [45, 48]. Moreover, abused women who have a close, supportive network are also more likely to utilize formal support services that mitigate the adverse mental and other effects of abuse [13, 16, 19, 46, 48, 56]. Abused women who receive both informal and formal support experience the compounded benefits evident through their recovery [57].

Since the 2000s’, Zimbabwe’s formal economy slumped, giving way to marked negative socio-economic impacts on its population, which include worsening unemployment rates, higher rates of poverty, food insecurity, and reliance on the informal economy for livelihoods [58]. The mental health impacts of the poor socio-economic conditions on women were documented in studies that have been conducted among localised and facility-based samples of pregnant women and those living with HIV [4, 6]. Some studies have shown an increased prevalence of women’s experiences of IPV in the past decade and attributed this to women’s lower levels of economic autonomy and household decision-making [6, 59–61]. Notwithstanding, research exploring the cumulative effects of socio-economic conditions and trauma exposures on both mental ill-health and women’s experiences of IPV using population-based samples in Zimbabwe has been limited. We aimed to determine the associations and inter-relationships between depressive symptoms and socio-economic factors, IPV, and traumatic exposures and among a nationally representative sample of women from Zimbabwe. Based on evidence generated from other studies, the study tested the following hypotheses: food insecurity, childhood, and other trauma are associated with women’s experiences of IPV and mental ill-health; women’s experiences of IPV co-relate with non-partner rape experience; lower social support indicators, including finding money in emergency and support have bi-directional relationships with women’s IPV experiences and mental-ill health.

## Methods

### Survey design and sampling

The study is a secondary analysis of data collected through a nationally representative cross-sectional survey conducted in 2012 in Zimbabwe [62]. A multi-stage random sampling method was used. Firstly, districts were selected from the 10 provinces list using a proportionate to size method. From the selected districts 225 enumeration areas, which are defined as the smallest geographic units for which census information is aggregated [12], were selected [62]. In each selected enumeration area, 20 households were selected. In each selected household, only one eligible adult woman, normally resident in the selected household, defined as one who slept at least four nights a week in the household, was randomly selected for participation [62]. Women who were visitors in selected households or intoxicated or not in a mental state to complete questionnaires were excluded [62]. The study recruited 3274 women in selected households. The survey’s overall response rate was 78% [62, 63]. For this secondary analysis we included women who had complete data for the depressive symptoms’ outcome (*N* = 2905), and we excluded those whose data from the measurement of any of the 20 Centre for Epidemiologic Studies Depression Scale (CESD) items was incomplete or missing (*N* = 369).

### Ethical considerations

The household survey was conducted by Gender Links, a regional non-governmental organisation in collaboration with and approved by the Government of Zimbabwe’s Ministry of Women Affairs, Gender and Community Development (MWAGCD) [62]. The first author oversaw the design and implementation of the study [62]. All participants provided written informed consent before participating. The World Health Organisation’s (WHO) Ethical guidelines and Safety Recommendations for conducting violence against women (VAW) research were followed [64]. To ensure participant safety, participants were assured of confidentiality and took the survey in privacy. Female research assistants were trained to detect participant distress and provided referral information about local psychosocial support services [62].

### Data collection

Based on the WHO’s Ethical and Safety Recommendations for Research on Domestic Violence against Women and to facilitate participant disclosure of violence experiences, female research assistants were trained to collect the data [62, 64]. Accredited professional translators were contracted to translate the questionnaire into local languages. The translated questionnaires were tested and validated with the inputs of local technical experts and the research assistants during training [62]. Participants self-completed a structured survey questionnaire which was loaded on personal digital assistants (PDAs), and the researchers assisted if needed [62]. The participants completed the survey in their language of preference i.e., in either English, Shona, or Ndebele [62].

### Variable measurement

Depressive symptoms were measured using the self-report and 20-item CESD (Cronbach’s alpha = 0.88). Responses to items were “0 = Rarely or none of the time (0), 1 = Some or a little of the time (1–2 days), 2 = Moderate amount of time (3–4 days) and 3 = Most or all of the time (5–7 days) [65]. In addition, we created a continuous CES-D score by summing up responses to items (Range 0–60) and used this as the primary outcome of all analyses. Higher scores were indicative of more severe depressive symptoms (Additional file 1).

Women’s experiences of emotional, sexual, physical, economic, and IPV and non-partner rape were measured using an adapted and pretested version of the WHO Multi-Country Study on Women’s Health and Domestic Violence: Core Questionnaire and WHO Instrument—Version 9 designed for use in developing countries [63]. Sexual IPV experience was measured by three items which included being forced by a man to have sex or perform sexual acts against one’s consent by using physical force or other means by a current or previous intimate partner. Physical IPV experience was measured by five acts of violence, including being slapped, pushed, kicked, hit, dragged, choked, beaten, burnt, threatened with a weapon, or having dangerous objects thrown at by a current or previous male intimate partner. Emotional IPV experience was measured by six items that include a male partner insulting or making a woman feel bad, making threats to hurt, scaring, intimidating, humiliating her in public, not allowing her to see friends, and bringing his girlfriends or other sexual partners home. Economic violence was measured by four items, including being forbidden to work, having earnings taken, not being given money for home essentials when the male partner could provide, or the male partner taking the woman’s earnings. Experience of IPV in the past 12 months was measured using a follow-up question to each set of items as follows: “Have any of these things happened in the past 12 months?” We created a three-category past year IPV experience: (0) No IPV experience, (2) experience of economic with physical, sexual, or emotional IPV (3) experience of physical, sexual, or emotional but without economic IPV experience.

Childhood abuse experience was measured through fourteen items of a modified version of the short form of the Childhood Trauma Questionnaire (CTQ) (Cronbach alpha = 0.75) [66]. Possible responses to items were 1 = Never, 2 = Sometimes, 3 = Often and 4 = Very often [66]. We created a continuous childhood abuse score by summing up the response items (Range 14–56). A score greater than 14 was indicative of childhood abuse experience. We created a binary category of no childhood abuse (score ≤ 14) and any childhood abuse experience (score ≥ 15).

Experience of other traumatic life events was measured through 10 items adapted from the Life Events Checklist from the PTSD Checklist [67]. Trauma events included being imprisoned, experiencing civil unrest or war, being seriously injured and requiring hospitalization, being close to death, witnessing the murder of a family member, friend or stranger, unnatural death of a family member or friend, being tortured, robbed, hijacked or kidnapped. Responses to items were either 0 = no or 1 = yes. We created a continuous life events score by summing up the response items (Range 0–10). We used the traumatic live events score to create a binary trauma variable of no trauma exposure (score = 0) and any trauma exposure (score ≥ 1). Participants’ alcohol consumption in the past year was also measured using one item of the Alcohol Use Disorder Identification Test (AUDIT) scale i.e. “Have you drunk alcohol in the past 12 months?” [68].

Data was collected on the demographic indicators, which included age group, number of children, and level of education attained. Socio-economic data were collected, and this included earning income in the past year and food insecurity. Indicators for social support which were measured included the participants’ability to find money in an emergency. Women were also asked about their help-seeking when dealing with emotional difficulties—i.e., whether they had sought help from family members, friends, or other people in their social network and whether they had sought professional help from a counsellor, doctor, psychologist, or any other formal service provider. Women were also asked whether they had tested for HIV, collected their results, and self-reported their HIV test results.

### Data analysis

We conducted analyses in Stata version 16 and used svy: commands which took into account the survey’s multi-stage sample design. We compared the mean depression scores for dichotomous variables using the t tests and mv test syntax codes for variables with more than two categories. We used linear regression modelling to investigate the bivariate and multivariate associations between depressive symptoms and explanatory factors. Only explanatory factors that had a p-value less than 0.2 in bivariate analyses were included in the multivariate regression models [69]. We used a stepwise backward elimination approach to eliminate non-significant associations until we obtained the final model, which was parsimonious [69].

We used structural equation modelling (SEM) with maximum likelihood estimation to investigate the inter-relationships of depression and explanatory factors. For our apriori-hypothesis, we assumed direct paths between explanatory variables and the primary outcome of depressive scores based on the results of the multivariate regression model—i.e. education, food insecurity, trauma factors, HIV positive status had effects on depressive scores. We also hypothesized paths between explanatory factors as guided by literature cited in the introduction section i.e. food insecurity, childhood and other trauma have effects on IPV, IPV co-relates with non-partner rape, lower social support indicators, including finding money in an emergency and support have bi-directional relationships with IPV. We specified model paths and allowed the errors of indicator variables to co-vary when the covariance improved the model fit and was theoretically justifiable [70]. We estimated the SEM model and tested for the goodness of fit of the model by assessing the Comparative Fit Index and the Root mean squared error of approximation (RMSEA) [70, 71].

## Results

### Study population characteristics

Table 1 illustrates the description of the sample including by depressive symptoms. A total of 40.6% were under 30 years, while almost a quarter were aged 45 + years. Slightly more than half of the sample had only primary education as their highest level of education, with only 1 in 20 having gone through tertiary education. The data shows that significant proportions of women were economically challenged based on our three measures of socio-economic status: 42% reported food insecurity, 78% had not engaged in any activity to earn income in the past year and they had relatively low instrumental social support, and 75% would find it difficult or very difficult to find money in an emergency. Regarding social support, about two-thirds of women had sought emotional social support: 44% sought either informal or professional emotional support and 22% sought both informal and formal social support. Slightly over a quarter (26%) of women had experienced IPV in the past year, 87% experienced some childhood trauma, and 47% had experienced other traumatic life events. Almost similar proportions of women experienced non-partner rape in their lifetime (6.8%) or self-reported an HIV-positive status (6.1%).

**Table 1:** Mean depression scores disaggregated by different variables and bivariate associations

Variable	Total		Mean depression score comparison				Bivariate regression				
	N	%	Mean depression score	95% CI		*P* value	Coefficient	95% CI			*P* Value
Age											
18–24 years	624	21.48	12.37	11.59	13.15	0.316	1.00				
25–39 years	1382	47.57	13.04	12.46	13.63		0.68	− 0.16	1.52	0.114	
40 + yeas	899	30.95	12.94	12.06	13.81		0.57	− 0.42	1.56	0.258	
Primary school education and lower	1479	50.91	12.99	12.26	13.73	0.06	1.00				
Secondary education	1275	43.89	12.54	11.94	13.14		− 0.46	− 1.29	0.38	0.282	
Tertiary education	151	5.20	14.34	12.92	15.77		1.35	− 0.24	2.94	0.096	
Food insecure	1,219	41.96	13.48	12.92	14.05	0.002	1.00				
Food secure	1,686	58.04	12.42	12.01	12.83		− 1.07	− 1.89	− 0.24	0.011	
No income	2,261	77.83	12.65	12.26	13.04	0.017	1.00				
Income	644	22.17	13.63	12.95	14.32		0.98	0.06	1.90	0.037	
Instrumental support-Difficult to find money	2,193	75.49	13.17	12.76	13.57	0.002	1.00				
Instrumental support-Easy to find money	712	24.51	11.94	11.36	12.52		− 1.23	− 2.06	− 0.39	0.004	
Currently not in relationship	585	20.14	12.98	12.06	13.90	0.096	1.00				
Not cohabiting with partner	478	16.45	13.65	12.64	14.66		0.67	− 0.56	1.90	0.285	
Cohabiting with partner	1842	63.41	12.62	12.07	13.18		− 0.35	− 1.29	0.58	0.456	
No IPV past year	2151	74.04	11.78	11.22	12.33	< 0.0001	1.00				
Economic IPV & other forms past year	384	13.22	17.14	16.04	18.23		5.36	4.21	6.51	< 0.0001	
Other forms and no economic IPV past year	370	12.74	14.76	13.76	15.75		2.98	1.92	4.04	< 0.0001	
No trauma exposure	1,554	53.49	10.18	9.77	10.58	< 0.0001	1.00				
Trauma exposure	1,351	46.51	15.96	15.45	16.47		5.79	4.94	6.63	< 0.0001	
No childhood abuse	390	13.43	8.800	8.05	9.55	< 0.0001	1.00				
Any childhood abuse	2515	86.57	13.50	13.13	13.86		4.70	3.74	5.65	< 0.0001	
No lifetime non-partner rape	2,707	93.18	12.60	12.26	12.94	< 0.0001	1.00				
Lifetime partner rape	198	6.82	16.57	14.91	18.24		3.98	2.14	5.81	< 0.0001	
No alcohol consumption past year	2659	91.53	12.70	12.35	13.05	< 0.0017	1.00				
Alcohol consumption past year	246	8.47	14.64	13.41	15.88		1.94	0.64	3.25	0.004	
No boyfriend/husband	585	20.14	12.98	12.06	13.90	0.223	1.00				
Partner no alcohol consumption past year	1330	45.78	12.55	11.94	13.17		− 0.43	− 1.40	0.54	0.387	
Partner alcohol consumption past year	990	34.08	13.22	12.48	13.95		0.24	− 0.79	1.27	0.649	
No help sought	990	34.08	11.60	10.78	12.43	< 0.0001	1.00				
Sought informal or professional emotional support	1274	43.86	13.38	12.79	13.96		1.77	0.90	2.65	< 0.0001	
Sought both informal and professional emotional support	641	22.07	13.79	12.85	14.74		2.19	1.00	3.38	< 0.0001	
HIV status undisclosed	2728	93.91	12.59	12.25	12.92	< 0.0001	1.00				
HIV positive self-reported	177	6.09	17.17	15.30	19.03		4.58	2.68	6.48	< 0.0001	

The mean depression score for all participants was 12.9 [95%CI (12.35, 13.38)], and 15.4% had a depressive symptom score of 21 or higher. Table 1 shows the mean depressive symptoms score disaggregated by education levels, food security, earning income, finding money in emergencies and experiences of childhood abuse, non-partner rape, IPV or other trauma, HIV status, and seeking emotional support. Being food secure, having earned an income, and having relative ease of finding money in an emergency were independently protective of higher depressive symptom scores (*p* < 0.05). History of childhood or other trauma, lifetime non-partner rape, or any IPV in the past year were independently associated with increased depressive symptom scores. Women who self-reported a positive HIV status were more likely to have higher depressive symptom scores compared to those who did not (*p* < 0.05). Women’s use of either or both informal and formal support was associated with higher depressive symptoms score (*p* < 0.05).

Table 2 shows the results from multivariate linear regression analysis. Women who attained secondary or tertiary education had an increased risk of higher depressive symptom scores compared to those who had primary or no education. Women who experienced IPV in the past year or non-partner rape were more likely to have higher depressive symptom scores compared to women who did not experience IPV. The strength of the association was greater for women who experienced IPV forms, including economic IPV compared to those who experienced IPV but not of economic nature. Having experienced childhood or other traumas also increased women’s risk of depressive symptoms. Women who tested HIV- positive were more likely to score higher depressive symptom scores compared to women who were HIV-negative or who did not know their status. Women who sought emotional support were more likely to have higher depressive symptom scores compared to women who did not seek emotional support. Women who found it easy to find money in an emergency were less likely to have high depressive symptom scores compared to those who found it difficult.

**Table 2:** Multivariate linear regression model for factors associated with depressive symptoms scores

	Coef	95%CI		*P* value
Primary school education and lower	1.00			
Secondary education	0.04	−0.72	0.79	0.92
Tertiary education	1.90	0.35	3.45	0.016
Food secure	−0.69	−1.51	0.12	0.095
Income past year	0.07	−0.77	0.91	0.876
Easy to find money in emergency	−0.80	−1.60	0.00	0.05
No IPV past year	1.00			
Economic IPV & other forms past year	3.96	2.92	5.00	< 0.0001
Other forms and no economic IPV past year	2.07	1.09	3.06	< 0.0001
Other trauma exposure	4.96	4.19	5.73	< 0.0001
Any childhood abuse	2.61	1.67	3.54	< 0.0001
Any lifetime non-partner rape	2.29	0.54	4.04	0.01
HIV + self-report	3.25	1.45	5.06	< 0.0001
No help sought	1.00			
Sought either informal or professional support	0.83	0.03	1.63	0.042
Sought both informal and professional support	1.05	0.02	2.09	0.046

**Fig. 1:**
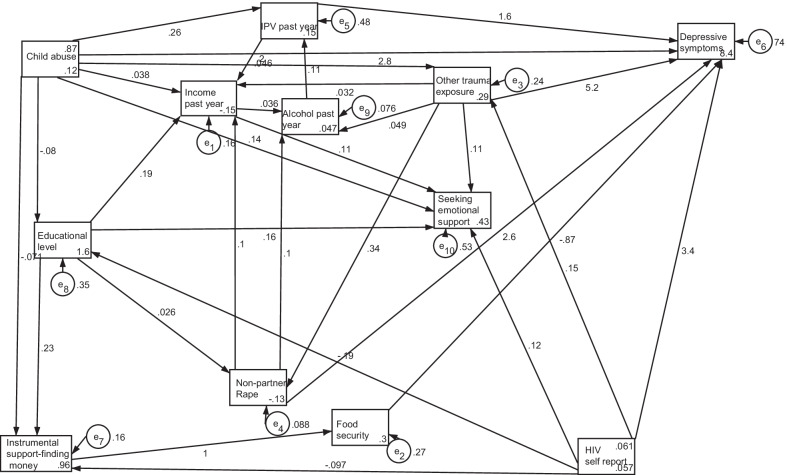
Structural equation model pathway diagram

The SEM model had good fit to the data (TLI = 0.988; CFI = 0.972; RMSEA = 0.018). Figure 1 and Tables 3 and 4 show the inter-relationships of variables among women from SEM analysis. Other trauma, IPV, non-partner rape, childhood abuse, and HIV status had strong direct effects on depressive symptoms. Food insecurity also directly impacted on depressive symptoms. The indirect effects of education level, income, HIV status, childhood, and other life trauma on depression were moderated by instrumental social support and emotional help-seeking. Instrumental social support impacted on food security. Violent and traumatic experiences, including partner violence, non-partner rape, and childhood abuse had impacts on women’s engagement in income-generating activities.

**Table 3:** SEM model statistical output

Structural	Direct effects				Indirect effects				Total effects			
	Coef	95% CI		*P* > z	Coef	95% CI		*P* > z	Coef	95% CI		*P* > z
*Depressive symptoms*												
← Income past year	0.000				0.006	0.000	0.013	0.065	0.006	0.000	0.013	0.065
← Alcohol past year	0.000				0.175	0.023	0.326	0.024	0.175	0.023	0.326	0.024
← Food secure	− 0.868	− 1.505	− 0.231	0.008	0.000				− 0.868	− 1.505	− 0.231	0.008
← Other trauma exposure	5.158	4.520	5.795	< 0.0001	0.908	0.356	1.459	0.001	6.065	5.235	6.896	< 0.0001
← IPV past year	1.555	1.104	2.007	< 0.0001	0.000	0.000	0.001	0.082	1.555	1.104	2.007	< 0.0001
← Instrumental support-finding money	0.000				− 0.893	− 1.558	− 0.228	0.008	− 0.893	− 1.558	− 0.228	0.008
← Educational level	0.000				− 0.137	− 0.297	0.024	0.095	− 0.137	− 0.297	0.024	0.095
← Non-partner rape	2.589	1.340	3.839	< 0.0001	0.019	0.001	0.037	0.037	2.608	1.359	3.857	< 0.0001
← Childhood abuse	2.794	1.856	3.732	< 0.0001	1.669	1.292	2.047	< 0.0001	4.464	3.489	5.439	< 0.0001
← HIV self-report	3.436	2.115	4.757	< 0.0001	0.999	0.585	1.413	< 0.0001	4.435	3.068	5.802	< 0.0001
*Income past year*												
← Alcohol past year	0.000				0.005	0.000	0.010	0.031	0.005	0.000	0.010	0.031
← Other trauma exposure	0.032	0.003	0.061	0.030	0.036	0.012	0.060	0.003	0.068	0.031	0.105	< 0.0001
← IPV past year	0.046	0.026	0.067	< 0.0001	0.000	0.000	0.000	0.136	0.046	0.026	0.067	< 0.0001
← Educational level	0.192	0.168	0.217	< 0.0001	0.003	0.001	0.005	0.014	0.195	0.171	0.220	< 0.0001
← Non-partner rape	0.104	0.047	0.161	< 0.0001	0.001	0.000	0.001	0.046	0.105	0.048	0.162	< 0.0001
← Childhood abuse	0.038	− 0.005	0.081	0.082	0.010	− 0.006	0.026	0.217	0.048	0.004	0.092	0.033
← HIV self-report	0.000				− 0.028	− 0.047	− 0.008	0.005	− 0.028	− 0.047	− 0.008	0.005
*Alcohol past year*												
← Income past year	0.036	0.011	0.060	0.004	0.000	0.000	0.000	0.213	0.036	0.011	0.060	0.004
← Other trauma exposure	0.049	0.029	0.069	< 0.0001	0.038	0.019	0.058	< 0.0001	0.088	0.060	0.115	< 0.0001
← IPV past year	0.000				0.002	0.000	0.003	0.014	0.002	0.000	0.003	0.014
← Educational level	0.000				0.010	0.005	0.015	< 0.0001	0.010	0.005	0.015	< 0.0001
← Non-partner rape	0.105	0.065	0.145	< 0.0001	0.004	0.000	0.007	0.025	0.108	0.068	0.148	< 0.0001
← Childhood abuse	0.000				0.018	0.012	0.025	< 0.0001	0.018	0.012	0.025	< 0.0001
← HIV self-report	0.000				0.011	0.004	0.018	0.002	0.011	0.004	0.018	0.002
*Emotional support*												
← Income past year	0.107	0.041	0.173	0.002	0.000	0.000	0.000	0.118	0.107	0.041	0.173	0.002
← Alcohol past year	0.000				0.001	0.000	0.001	0.076	0.001	0.000	0.001	0.076
← Other trauma exposure	0.113	0.060	0.167	< 0.0001	0.007	0.001	0.013	0.017	0.121	0.067	0.174	< 0.0001
← IPV past year	0.000				0.005	0.001	0.009	0.010	0.005	0.001	0.009	0.010
← Educational level	0.157	0.110	0.203	< 0.0001	0.021	0.008	0.034	0.002	0.178	0.133	0.222	< 0.0001
← Non-partner rape	0.000				0.011	0.002	0.020	0.017	0.011	0.002	0.020	0.017
← Childhood abuse	0.142	0.063	0.221	< 0.0001	0.015	− 0.003	0.033	0.104	0.157	0.078	0.236	< 0.0001
← HIV self-report	0.118	0.007	0.228	0.037	− 0.017	− 0.038	0.005	0.123	0.101	− 0.011	0.213	0.076
*Food secure*												
← Instrumental support-finding money	1.029	0.903	1.156	< 0.0001	0.000				1.029	0.903	1.156	< 0.0001
← Educational level	0.000				0.236	0.208	0.264	< 0.0001	0.236	0.208	0.264	< 0.0001
← Childhood abuse	0.000				− 0.092	− 0.133	− 0.050	< 0.0001	− 0.092	− 0.133	− 0.050	< 0.0001
← HIV self-report	0.000				− 0.145	− 0.205	− 0.085	< 0.0001	− 0.145	− 0.205	− 0.085	< 0.0001
*Other trauma exposure*												
← Childhood abuse	0.197	0.143	0.251	< 0.0001	0.000				0.197	0.143	0.251	< 0.0001
← HIV self-report	0.146	0.083	0.209	< 0.0001	0.000				0.146	0.083	0.209	< 0.0001
*IPV past year*												
← Income past year	0.000				0.004	0.000	0.008	0.055	0.004	0.000	0.008	0.055
← Alcohol past year	0.112	0.021	0.204	0.016	0.000	0.000	0.000	0.263	0.112	0.021	0.204	0.016
← Other trauma exposure	0.000				0.010	0.001	0.018	0.025	0.010	0.001	0.018	0.025
← IPV past year	0.000				0.000	0.000	0.000	0.072	0.000	0.000	0.000	0.072
← Educational level	0.000				0.001	0.000	0.002	0.038	0.001	0.000	0.002	0.038
← Non partner rape	0.000				0.012	0.001	0.023	0.029	0.012	0.001	0.023	0.029
← Childhood abuse	0.258	0.184	0.333	< 0.0001	0.002	0.000	0.004	0.026	0.261	0.186	0.335	< 0.0001
← HIV self-report	0.000				0.001	0.000	0.002	0.058	0.001	0.000	0.002	0.058
*Instrumental support-finding money*												
← Educational level	0.230	0.205	0.254	< 0.0001	0.000				0.230	0.205	0.254	< 0.0001
← Childhood abuse	− 0.071	− 0.108	− 0.033	< 0.0001	− 0.018	− 0.033	− 0.004	0.014	− 0.089	− 0.129	− 0.049	< 0.0001
← HIV self-report	− 0.097	− 0.149	− 0.044	< 0.0001	− 0.044	− 0.065	− 0.023	< 0.0001	− 0.141	− 0.197	− 0.085	< 0.0001
Educational level												
← Childhood abuse	− 0.080	− 0.143	− 0.017	0.013	0.000				− 0.080	− 0.143	− 0.017	0.013
← HIV self-report	− 0.193	− 0.283	− 0.103	< 0.0001	0.000				− 0.193	− 0.283	− 0.103	< 0.0001
*Non partner rape*												
← Other trauma exposure	0.345	0.212	0.477	< 0.0001	0.000				0.345	0.212	0.477	< 0.0001
← Educational level	0.026	0.010	0.041	0.001	0.000				0.026	0.010	0.041	0.001
← Childhood abuse	0.000				0.066	0.044	0.088	< 0.0001	0.066	0.044	0.088	< 0.0001
← HIV self-report	0.000				0.045	0.017	0.074	0.002	0.045	0.017	0.074	0.002

**Table 4:** Covariances- SEM output

	Coef	Standard error	95% CI		*P* > z
Cov(e. Income past year,e. finding money)	0.019	0.003	0.014	0.024	< 0.0001
Cov(e. Emotional support,e.Depressive symptoms)	0.315	0.116	0.088	0.542	0.007
Cov(e. Emotional support,e. IPV past year)	0.023	0.009	0.004	0.041	0.015
Cov(e. Food secure,e. finding money)	−0.110	0.011	−0.132	−0.088	< 0.0001
Cov(e. Other trauma exposure,e. IPV past year)	0.025	0.006	0.014	0.036	< 0.0001
Cov(e. Other trauma exposure,e. Non partner rape)	−0.079	0.017	−0.111	−0.046	< 0.0001
Cov( Childhood abuse, HIV self-report)	0.004	0.002	0.001	0.007	0.004

## Discussion

Our study aimed to investigate individual-level factors that were associated with depressive symptoms among a nationally representative sample of women from Zimbabwe. We found that many Zimbabwean women lived in circumstances of economic deprivation: 50% of the sampled women had only a primary education or less, 42% reported food insecurity, 78% had not engaged in activity to earn income in the past year and 75% would find it difficult or very difficult to find a low sum of money in an emergency. We found a relatively high prevalence of women’s experience of IPV in the past year compared to what was reported in studies that have used similar methods elsewhere in the Southern African region [72–74]. The prevalence of IPV in this violence against women-focused survey was comparable to that found in the most recent omnibus Zimbabwe Demographic Health Survey [59, 75]. We also found high reporting of childhood traumas and other adult traumas. Moreover, our study confirmed that these multiple individual-level factors of economic hardship and trauma had cumulative impacts on women’s depression, as has been shown in other settings [12–14, 21, 23]. Our findings also show higher depression amongst HIV-positive women, who have also been found to have higher experience of IPV [76–78]. This study also extends the body of evidence of these associations within the general population. Similar impacts were reported in Zimbabwean studies that involved women attending antenatal clinics, people living with HIV, and adolescents in primary health care settings [4, 6, 7, 79, 80].

Having support networks that could provide instrumental support, in this case money in emergency, was protective of depressive symptoms. Yet, research shows that women survivors of violence will have less social support and networks compared to women who are not abused [81]. Among abused women, those who have higher social support or networks or become members of survivor support groups were shown to have lower depressive symptomatology, more resilience, and faster recovery [81–84]. Our findings also indicate the beneficial impacts of seeking help in dealing with emotional difficulties as a mitigator in the observed relationships between economic hardship, trauma exposure, and depressive symptoms among women. The mitigating effects of social support among women exposed to abuse and in fostering resilience are well documented in the literature [46, 81–83, 85–87]. However, women who sought both informal and professional support appeared not to be protected from depression. This could be explained by the fact that women who seek both types of support are those exhibiting high symptomatology and that the interventions were not effective in addressing the high symptomology. These findings warrant further research to deepen understanding of what interventions are effective to address the cumulative mental ill-health effects of economic hardship and trauma exposure and tailored to the different groups of women in Zimbabwean settings. Community support and group-based interventions have been tested and proven effective in Zimbabwean settings. These must be brought to scale because they have the potential to alleviate depressive symptoms through strengthening social support available to women and building problem-solving skills among women survivors and those living with HIV [8, 88–90]. Notwithstanding, the provision of trauma-focused psychotherapies must continue to be a priority in services for women survivors of violence to facilitate healing and better mental health [84].

While women’s experiences of IPV and non-partner violence impact mental health, violence against women and girls (VAWG) is preventable. It has been demonstrated that theoretically grounded, and culturally-adapted interventions can reduce both men’s perpetration and women’s experiences of violence [91–93]. A robust body of evidence indicates a repertoire of VAWG strategies and interventions that were proven effective in low- and middle-income country settings [91–94]. It will be important that scholars in the field adapt, test, and scale-up effective VAWG interventions in Zimbabwean settings. Based on the findings from this study, it will be important that interventions chosen for further research and adaptation have a combined focus on reducing VAWG, poverty, and mental ill-health [91–95].

Our study has several limitations. Due to the secondary design of this study, we did not have control in the measurement of all the variables used in the analysis. We are unable to quantify the possibility of recruitment bias and differences between women who consented to participate in the survey and those that did not participate. Self-report and symptomatic measures were used for variables, and we acknowledge that clinical diagnosis of depression and HIV may have yielded different results from these. The associations were deduced from cross-sectional data, but longitudinal data would have had better control in terms of the direction of associations. However, the fit statistics obtained in SEM analysis confirm our assumed directionality between variables. Finally, this study involved a secondary analysis of data that was collected several years ago. Things may have changed between the time of data collection and the time of analysis. Still, the robust analysis and the findings’ agreement with previous studies’ findings testify to the importance of the issues and direction of outcomes. In addition, our study provides valuable insights that elucidate the drivers of women’s depression using data from a representative sample, making its findings generalisable to the population of Zimbabwean women.

## Conclusion

We have demonstrated that multiple individual-level factors including economic hardship, exposure to traumas including IPV, living with HIV, and having low social support have a cumulative negative toll on mental health among Zimbabwean women from the general population. Interventions are needed which respond to the mental ill health effects in addition to prevention interventions that tackle the multiple risk factors that we have identified. Further research is necessary to identify, adapt, test, and scale-up interventions that have been effective in other low- and middle-income settings.

## Supplementary Information

**Additional file 1.** Minimal data set. Raw data of depressive symptoms, socio-economic factors, traumatic exposure and recent intimate partner violence experiences among women in Zimbabwe.

## Data Availability

The datasets generated and/or analysed during the current study are not publicly available due to them being needed for further analysis and use for student research projects, however they are available from the corresponding author on reasonable request.
