# Supplementary material for: Associations between depressive symptoms, socio-economic factors, traumatic exposure and recent intimate partner violence experiences among women in Zimbabwe: a cross-sectional study
Source: BMC Womens Health. 2022 Jun 22;22:248. doi: 10.1186/s12905-022-01796-w (PMC9219137; doi:10.1186/s12905-022-01796-w)
Supplement: Supplementary file 1 — Additional file 1. Minimal data set. Raw data of depressive symptoms, socio-economic factors, traumatic exposure and recent intimate partner violence experiences among women in Zimbabwe. [file 12905_2022_1796_MOESM1_ESM.pdf]

moneyfnd	eat	lived	beatenm	toldlazy	touchgen	humiliat	beaten	sexolder	pardrunk
1	1	1	1	1	1	1	1	1	1
3	1	1	1	1	1	1	1	1	1
1	1	1	1	1	1	1	1	1	1
1	2	1	3	1	1	1	1	2	1
1	1	1	1	1	1	1	1		1
1	1	1	1	1	1	1	1	1	1
1	1	1	1	1	1	1	1	2	1
1	1	1	1	1	1	1	1	1	1
1	1	1	1	2	1	1	1	1	1
1	2	2	2	2	1	1	1		1
1	3	1	3	2	3	2	3	1	3
	1	1	1	2	2	1	1	1	1
1	1	1	1	1	2	1	1	2	1
1	1	1	2	2	1	1	1	1	1
1	1	3	1	1	1	1	1	1	1
1	1	1	1	2	1	1	1	1	1
1	1	1	1	1	1	1	1	1	1
3	1	1	1	2	1	1	1	1	1
1	4	2	1	1	1	1	4	1	1
2	1	1	1	1	1	2	2	1	1
2	1	2	2	3	4	1	1	3	1
2	2	2	1	2	1	2	1	1	1
3	1	1	2	2	1	2	1	1	1
3	2	3	3	3	1	1	1	1	1
1	3	3	2	2	1	1	4	1	2
1	1	1	1	1	1	1	2	1	1
2	1	1	1	2	1	1	2	1	1
2	1	2	2	4	2	1	4	2	1
1	4	2	1	1	2	1	4	1	1
3	2	3	2	2	1	2	2	1	1
3	1	1	1	2	1	1	1	1	1
3	1	1	1	1	1	1	1	1	1
2	1	4	3	1	1	2	1	1	1
2	1	1	2	2	1	1	1	1	1
2	1	2	2	2	1	1	1	1	1
4	1	2	2	2	1	1	1	1	1
3	2	2	1	1	1	1	2	2	1
1	1	1	1	2	1	1	1	1	2
2	1	1	1	3	1	3	1	1	5
2	1	1	1	2	1	4	3	1	1
2	1	1	1	2	1	1	1	1	1
3	1	1	1	1	1	1	2	1	1
2	1	1	1	2	1	2	1		1
1	2	3	1	2	1	2	1	1	1
2	1	2	1	1	1	1	1		1
3	1	1	1	1		1	1		1

3	1	3	2	1	1	1	2	1	1
2	1	1	1	2	1	2	1	1	1
2	1	1	3	1	1	1	1	1	1
2	1	1	1	1	4	1	4	1	1
3	1	1	1	1	1	1	1	1	1
2	1	2	1	1	1	1		1	1
1	1	1	1	4	1	2	4	1	1
1	1	1	1	4	1	2	4	1	1
2	1	1	2	1	1	2	1	1	1
2	3	3	4	2	1	2	3	1	5
3	1	1	1	1	1	1	1	1	1
3	2	1	1	1	1	1	1	1	1
2	2	1	3	1	1	1	1	1	2
2	1	2	2	1	1	1	1	1	1
3	1	2	2	1	1	1	1	1	1
3	1	1	1	4	1	1	2	1	1
2	1	1	3	1	1	1	1	1	1
3	1	3	1	1	1	1	1	1	1
2	4	1	2	1	1	1	2	1	1
2	1	1	1	2	1	1	1	1	1
3	1	1	3	1	1	1	1	1	1
3	1	1	1	1	1	1	3	1	1
2	1	1	1	1	1	1	1	1	1
1	1	1	2	2	1	1	2	1	1
1	2	1	1	2	1	1	1	1	1
3	1	1	1	1	1	1	1	1	1
1	2	1	1	1	1	1	1	1	1
4	2	3	2	3	4	4	1	1	1
1	1	2	1	2	1	1	2	1	3
1	1	2	3	2	2	2	2	1	1
1	2	2	5	2	2	3	3	1	5
1	1	1	1	2	1	2	1	2	1
3	4	4	2	2	1	2	2	2	2
4	3	4	3	4	3	4	3	4	1
1	2	2	2	1	2	1	2	2	1
3	1	1	1	1	1	1	1	1	1
2	1	1	1	1	1	1	1	1	1
3	1	1	1	1	1	1	1	1	1
2	1	1	1	1	1	1	1	1	1
3	1	1	1	1	1	1	1	1	1
1	1	1	1	1	1	1	1	1	1
1	1	1	1	1	1	1	1	1	1
1	3	2	1	2	1	2	2	1	2
1	1	3	1	1	2	1	1	1	1
2	2	1	2	2	2	1	1	1	1
4	1	1	1	1	1	1	1	1	1
1	2	1	2	2	2	2	2	2	1

4	3	3	1	1	1	3	3	3	3
2	1	1	1	1	1	1	1	1	1
2	1	1	1	1	1	2	2	2	1
2	2	2	4	3	1	3	2	3	1
2	1	1	1	1	1	1	1	3	1
2	1	1	2	2	2	1	1	1	1
2	1	1	2	2	2	1	2	1	1
2	1	2	2	3	1	2	1	1	1
1	2	1	3	3	1	1	1	1	1
1	1	1	3	3	2	1	1	1	1
2	1	2	3	3	1	2	3	1	1
2	1	2	2	1	1	1	1	3	1
2	1	3	2	1	1	2	2	1	1
2	2	1	4	3	2	2	2	1	2
2	4	1	1	2	2	1	1	3	1
3	2	2	4	2	1	1	1	1	1
1	2	2	3	3	3	3	2	1	1
1	1	2	4	3	1	3	1	4	1
1	2	2	3	3	1	1	1	1	1
2	1	1	1	1	1	1	1	1	1
3	2	1	2	1	2	1	1	1	1
2	1	1	4	1	1	3	2	4	1
2	1	3	2	3	1	2	1	3	1
3	2	2	1	2	1	1	1	3	1
2	1	2	1	3	1	1	1	1	1
1	3	1	2	1	2	2	2	1	1
2	1	3	3	3	2	2	3	1	1
2	2	2	3	1	1	3	1	1	1
2	2	2	3	1	1	3	1	1	1
1	1	1	2	1	1	1	1	1	1
3	1	1	2	2	1	2	1	1	1
3	1	3	1	1	1	1	2	1	1
1	2	2	2	2	2	1	2	1	1
1	1	1	1	1	1	1	1	1	1
2	1	1	3	1	2	1	1	1	
2	1	1	2	1	1	1	1	1	1
3	1	1	1	1	1	1	1	1	1
2	1	2	1	1	1	1	1	1	1
4	1	1	1	1	1	1	1	1	1
4	2	3	4	2	2	3	2	3	2
2	3	1	1	1	1	1	2	1	1
2	3	1	3	3	1	1	3	1	1
3	2	3	1	3	3	1	2	1	1
1	1	1	2	2	1	1	1	1	1
	1	1	1	1	1	1	1	1	1
2	1	1	1	3	1	1	1	1	1
1	3	3	1	2	1	1	3	1	1

2	1	2	1	1	2	1	1	1	1
2	1	2	3	1	1	1	1	2	1
2	1	1	2	2	1	1	1	1	1
1	2	2	3	2	2	1	1	1	1
3	2	1	2	3	2	1	2	1	1
1	1	2	1	1	2	1	1	1	1
1	2	2	3	2	2	2	1	1	1
3	1	1	2	1	1	1	3	4	2
3	1	1	1	1	1	1	1	1	1
3	1	1	1	1	1	1	1	1	1
2	2	2	1	1	2	1	1	1	1
2	1	1	1	2	2	1	1	1	4
2	1	2	4	1	1	4	1	1	1
1	1	3	2	1	1	1	2	1	1
2	2	2	2	2	2	1	1	2	1
2	1	1	1	1	1	1	1	1	1
2	1	1	1	1	1	1	1	1	1
3	1	1	3	3	2	3	3	2	1
2	1	1	1	2	1	1	1	1	1
2	1	1	1	2	1	1	1	1	1
2	1	1	1	2	1	1	1	1	1
2	1	1	1	1	1	2	2	1	1
4	1	3	4	1	1	1	1	1	1
2	1	1	3	2	2	3	2	1	1
2	2	1	3	1	1	1	2	1	1
3	1	2	2	1	1	1	1	1	1
2	1	2	2	1	1	1	2	1	1
3	2	2	1	1	1	1	3	1	1
3	1	2	1	1	1	1	1	1	1
2	3	1	1	1	2	1	2	1	1
2	1	2	1	1	1	1	1	1	1
2	2	3	1	1	1	1	1	1	1
3	1	1	1	1	1	1	1	1	1
3	1	2	1	1	1	1	1	1	1
2	1	1	1	1	1	1	1	1	1
2	2	3	1	1	1	1	1	1	1
2	1	1	1	1	1	1	1	1	1
2	3	3	5	3	1	1	1	1	5
2	2	2	1	1	1	1	1	1	1
2	2	2	1	1	1	1	1	1	1
2	1	2	1	1	1	1	1	1	1
3	1	2	3	3	1	3	3	1	1
3	1	1	1	1	1	1	1	1	1
2	1	1	2	2	1	1	1	2	1
2	2	1	3	2	1	1	2	1	1
3	1	1	1	1	1	2	1	1	1
2	1	1	1	2	1	1	1	1	1

3	1	1	1	1	1	1	1	1	1
3	1	1	1	2	1	1	2	1	1
2	1	2	3	2	1	2	1	1	1
2	2	3	2	2	1	2	1	1	1
2	3	3	1	2	1	1	1	1	1
3	1	1	2	2	1	1	1	1	1
2	2	1	1	2	2	2	1	1	1
2	1	4	1	1	1	1	1	1	1
3	1	2	2	3	2	1	2	1	1
3	1	1	1	1	1	1	1	1	1
2	1	1	1	1	1	2	2	1	1
3	1	1	1	1	1	1	1	1	1
2	1	2	1	1	1	1	1	1	5
2	1	1	1	2	1	2	2	1	1
3	1	1	1	1	1	1	1	1	1
1	2	1	4	1	1	1	1	1	1
1	3	1	2	2	1	1	1	1	1
3	1	2	1	1	1	1	1	1	1
2	4	2	2	1	1	1	1	1	1
1	2	1	1	1	1	1	1	1	1
1	4	1	1	1	1	1	1	1	3
1	2	2	3	1	1	1	1	1	1
1	2	2	1	2	1	1	2	1	1
1	4	1	1	1	1	1	1	1	1
1	2	1	4	1	1	1	1	1	1
1	4	1	1	1	1	1	1	1	1
1	1	1	1	1	1	1	1	1	1
1	1	1	1	1	1	1	1	1	1
1	2	1	1	1	1	1	1	2	1
2	3	3	1	1	1	1	1	1	1
1	3	1	1	1	2	1	2	1	2
1	3	1	1	1	1	1	1	1	1
1	4	1	1	1	1	1	1	1	1
1	4	1	1	1	1	1	1	1	1
1	2	1	1	3	2	2	2	1	1
1	2	1	1	3	3	1	4	1	3
1	1	1	1	4	2	3	1	1	1
2	2	3	1	2	2	1	1	1	1
1	3	1	2	1	1	1	2	1	1
3	1	1	1	1	1	2	1	1	1
2	1	1	1	1	1	1	1	1	1
2	1	3	4	3	3	3	3	3	3
1	2	1	3	2	1	2	3	1	3
2	1	2	3	2	2	1	3	1	2
2	4	2	2	2	1	1	1	1	1
1	2	1	1	2	1	2	2	1	3
1	1	1	1	2	2	2	2	1	2

1	1	1	1	1	1	1	1	1	1
1	1	1	1	2	1	2	2	1	1
1	1	1	2	2	1	1	1	1	1
3	1	1	1	1	1	1	1	1	1
3	1	1	1	1	1	1	1	1	1
2	1	1	1	1	1	1	1	1	1
1	2	1	3	1	1	1	2	1	1
3	2	2	2	1	1	1	1	1	2
2	2	2	2	2	1	2	2	1	1
2	2	2	1	1	1	1	1	1	1
2	1	2	1	2	1	1	1	1	5
2	1	1	1	3	1	1	1	1	1
2	1	1	1	1	1	1	1	1	1
2	4	3	1	1	1	1	1	1	1
2	1	1	1	1	1	1	1	1	1
2	1	2	1	3	1	1	1	1	1
2	1	2	1	1	1	1	1	1	1
1	1	1	1	3	1	1	1	1	1
1	1	1	1	3	1	1	1	1	1
2	2	1	1	1	1	1	1	1	1
1	1	1	1	1	1	1	1	1	1
2	1	1	1	1	2	1	3	1	1
2	1	1	3	4	1	2	1	1	1
2	1	3	5	1	1	3	4	1	5
2	2	3	2	2	1	1	1	1	5
3	1	1	1	1	1	1	1	1	1
1	1	3	3	1	4	2	1	3	3
3	1	1	1	1	1	1	1	1	1
2	1	1	1	2	1	1	2	1	3
2	1	1	1	1	1	1	1	1	4
1	1	2	1	1	1	1	1	1	1
3	4	3	1	1	1	1	1	1	1
3	4	3	1	1	1	1	1	1	1
2	2	2	1	1	1	1	1	2	1
1	1	1		1	2	2	2	2	5
4	2	1	2	1	1	1	1	1	1
3	1	2	2	2	1	1	2	1	1
3	1	2	2	1	1	1	1	3	1
3	1	2	1	1	3	2	2	1	1
2	4	4	1	3	2	1	2	4	1
1	1	2	2	1	1	1	1	1	1
1	1	1	1	1	1	1	1	2	1
2	1	1	1	2	1	1	1	1	1
3	1	2	1	1	1	1	1	1	2
1	1	1	1	1	1	1	1	1	1
2	1	1	1	1	1	1	1	1	1
3	1	1	1	1	1	1	1	1	

3	1	2	2	1	2	2	2	2	
1	1	3	1	1	1	3	2	1	1
1	1	1	1	1	1	1	1	1	1
	1	1	1	1	1	1	2	1	1
1	1	1	1	1	1	1	1	1	1
1	2	2	2	1	1	1	1	1	1
2	1	1	2	1	1	2	1	1	1
4	1	1	1	1	1	1	1	1	1
2	2	3	1	1	1	2	3	1	1
3	2	1	2	2	1	1	3	1	2
1	1	1	1	1	1	1	1	1	1
2	1	1	2	1	1	1	1	1	1
1	1	1	3	1	1	1	1	1	1
3	1	1	1	1	1	1	3	1	1
3	1	1	1	1	1	1	3	1	1
1	1	2	1	2	1	1	2	1	1
2	1	4	2	4	1	2	2	1	5
3	1	1	1	1	1	1	1	1	1
2	3	1	1	2	1	1	1	1	1
4	1	2	1	2	1	2	1	1	1
1	1	1	3	4	1	1	1	1	1
2	1	1	3	1	1	2	1	1	1
2	2	1	1	1	1	3	1	1	1
2	1	3	1	1	1	1	1	1	1
2	2	1	2	2	1	1	1	1	2
4	1	1	1	2	1	3	2	1	1
2	1	1	1	1	1	1	1	1	1
2	1	1	2	2	1	2	2	1	1
2	3	1		1	1	1	1	1	1
1	2	1	3	3	1	4	2	2	1
2	1	1	4	2	1	2	3	4	1
1	2	1	2	2	1	1	1	1	1
2	2	2	2	2	1	2	1	1	1
2	1	2	2	2	3	3	3	1	1
2	2	1	1	2	1	2	2	1	3
1	2	1	1	1	1	2	2	1	1
2	1	3	1	2	1	1	1	1	1
2	1	2	1	3	1	1	1	1	1
2	3	2	1	1	1	2	1	1	1
2	1	1	1	1	1	1	1	1	1
3	1	1	1	2	1	3	1	1	1
1	1	3	2	3	2	4	1	1	1
3	1	1	1	1	1	1	1	1	1
3	1	1	1	2	1	2	2	1	3
3	2	2	3	2	1	2	2	4	2
2	1	1	1	2	1	1	1	1	1
2	2	1	1	1	1	1	1	1	1

2	2	1	1	1	1	1	1	1	1
2	1	1	1	1	1	1	1	1	1
3	1	2	2	2	2	2	2	3	2
2	1	2	3	1	1	2	1	1	1
3	2	1	1	2	1	2	3	1	3
3	1	1	1	2	4	2	2	3	3
1	3	1	1	1	1	1	1	1	1
1	3	1	1	1	1	1	1	1	1
2	1	1	2	1	1	1	1	1	1
1	3	1	1	1	1	1	1	1	1
2	1	2	3	4	1	2	1	1	1
1	3	3	4	3	1	1	4	2	1
2	2	1	4	2	1	2	2	4	1
1	2	2	3	3	1	2	1	1	1
2	2	1	1	1	1	1	2	1	2
1	1	4	2	3	1	2	2	1	1
3	2	1	1	2	1	1	1	1	1
2	1	1	4	1	1	1	1	1	1
2	4	2	1	2	1	3	2	4	3
2	1	2	4	2	1	3	1	1	3
4	1	1	1	1	1	1	1	1	1
1	1	2	2	1	1	1	1	1	1
4	3	1	1	2	1	1	3	1	1
2	2	3	4	1	1	1	3	1	2
2	1	1	1	4	1	2	1	1	1
2	4	1	1	1	1	1	2	1	1
2	1	1	1	4	1	1	1	1	1
3	1	1	1	2	1	2	1	1	1
2	1	3	1	2	1	2	3	1	1
3	2	1	1	2	1	1	1	1	1
3	1	1	2	1	1	2	1	1	2
2	2	2	2	2	2	2	1	1	1
3	1	1	1	1	1	1	1	1	1
2	1	1	2	2	1	1	1	1	1
2	1	1	3	2	1	2	2	2	2
4	1	1	5	1	1	1	1	1	5
2	1	1	1	2	1	2	1	1	1
3	1	1	1	1	1	1	1	1	1
2	1	1	1	1	1	1	1	1	1
2	2	1	1	1	1	1	1	1	1
1	2	1	1	2	2	2	2	1	2
3	1	1	1	1	1	1	1	1	1
1	1	1	2	2	2	2	2	2	2
2	2	2	2	2	2	2	2	1	2
2	2	4	5	3	1	1	1	1	1
1	3	3	1	3	1	1	1	1	1
3	1	1	1	1	1	1	1	1	1

2	1	1	2	2	1	1	1	1	1
3	1	1	1	1	1	1	1	1	1
3	1	1	1	1	1	1	2	1	1
2	1	1	1	1	1	1	1	1	1
2	2	3	2	2	1	1	1	1	1
1	1	1	1	1	1	1	1	1	1
3	1	1	1	1	1	1	1	1	1
3	1	1	1	1	1	1	1	1	1
2	1	3	2	2	1	2	1	1	2
1	1	1	3	3	3	2	2	1	3
1	2	2	2	3	3	3	3	1	1
3	1	1	1	1	1	1	1	1	1
2	2	2	4	2	2	2	1	1	1
2	1	1	3	3	1	2	1	3	1
2	1	1	3	3	2	2	1	1	1
2		1	1	1	1	1	1	3	1
2	4	3	1	2	1	1	1	1	1
3	1	1	1	1	1	1	1	1	1
2	1	2	4	3	3	1	1	1	1
2	1	1	3	3	1	1	1	1	1
2	4	3	1	1	3	1	1	1	2
1	1	1	4	4	1	2	2	4	1
1	1	2	4	3	1	2	3	1	1
2	1	1	4	2	1	2	2	1	1
2	2	3	3	2	1	2	1	1	5
2	3	1	4	2	1	3	1	1	1
1	2	3	1	3	1	1	3	1	1
1	2	1	1	1	1	1	2	3	2
1	2	1	1	1	1	2	1	1	1
3	1	1	4	3	1	1	1	1	1
2	2	1	2	3	1	1	2	3	5
1	1	1	1	1	1	1	1	1	1
4	1	1	3	2	1	1	1	1	1
1	2	1	2	2	1	1	2	1	1
3	1	1	1	1	1	1	1	1	1
3	1	1	3	1	1	2	2	1	1
1	1	2	1	1	1	1	2	1	1
1	2	1	1	1	1	1	1	1	1
3	1	1	2	1	1	1	1	1	1
1	1	2	2	2	1	1	1	1	1
1	1	1	1	2	3	1	1	1	1
2	2	2	1	1	1	1	1	1	1
2	2	3	3	2	1	1	2	1	1
4	1	2	1	2	1	1	1	1	1
4	2	2	2	2	1	2	2	1	1
4	1	1	1	1	1	1	2	1	1
1	2	2	2	3	1	1	1	1	1

1	2	1	2	2	1	1	1	1	1
1	1	1	2	1	1	1	1	1	1
2	1	1	2	1	2	1	2	1	1
2	1	1	3	2	1	1	1	1	1
2	2	1	2	1	1	1	1	1	1
1	2	1	3	2	2	1	2	1	1
1	3	2	3	2	1	1	2	1	4
1	2	2	3	2	1	1	1	1	1
1	1	1	1	2	1	1	1	1	1
1	1	1	2	1	1	1	1	1	1
3	2	3	1	1	1	1	1	1	1
1	1	1	1	1	1	1	1	1	1
2	2	2	1	1	1	1	1	1	1
1	1	1	5	1	1	2	1	4	1
1	2	2	2	1	2	1	1	1	2
3	1	1	1	1	1	1	1	1	1
2	1	2	1	4	1	1	1	1	2
1	2	1	2	2	1	1	2	1	1
3	1	1	2	1	1	1	1	1	1
4	1	1	1	1	1	1	1	1	1
2	1	1	1	2	1	1	2	1	1
2	2	1	1	3	1	2	3	1	1
2	1	1	3	2	1	1	3	1	1
3	2	2	1	1	1	1	1	1	4
2	2	1	1	1	1	1	1	1	1
2	3	1	1	1	1	1	2	1	1
3	1	2	2	4	1	4	3	1	1
2	2	1	2	2	1	2	2	1	1
3	1	1	2	1	1	1	1	1	1
2	1	1	2	2	1	1	2	1	1
2	2	2	2	2	1	2	1	1	1
2	1	1	1	1	1	1	1	1	2
2	1	1	1	1	1	1	1	1	2
3	2	1	1	1	1	3	2	1	1
2	1	1	1	1	1	1	2	1	1
2	1	2	3	4	1	1	1	1	1
2	1	1	4	1	1	3	3	1	1
1	2	1	1	1	1	1	1	1	1
2	1	1	1	2	1	1	3	1	1
3	1	1	3	2	1	1	2	1	1
2	2	1	2	1	1	1	1	1	1
2	2	2	1	4	3	3	1	2	1
3	1	2	1	1	1	2	1	1	1
3	1	1	2	2	1	1	2	1	1
3	1	1	3	1	1	1	2	1	1
3	1	2	1	2	1	2	1	1	1
1	2	2	3	2	1	2	2	1	1

1	2	3	3	2	1	1	1	1	2
3	1	2	1	1	1	1	1	1	1
2	1	1	1	2	1	1	1	1	1
3	2	2	5	1	1	1	2	1	1
1	2	1	1	1	1	1	1	1	1
3	1	1	1	2	1	1	1	1	1
4	2	2	2	2	1	1	1	1	1
2	1	1	3	2	1	1	1	1	1
2	2	1	1	2	1	1	1	1	1
2	1	1	4	2	1	1	1	1	1
2	2	2	2	2	1	1	1	1	1
1	2	2	4	2	1	2	2	1	1
2	2	1	1	2	1	2	2	1	1
2	1	2	1	1	2	1	1	1	1
2	2	1	2	1	1	1	1	1	2
2	1	1	2	1	2	1	2	1	1
3	1	2	3	2	1	2	3	1	1
2	1	1	1	2	2	1	1	1	1
2	1	2	1	2	1	1	2	1	1
2	1	2	1	2	1	1	1	1	1
2	1	1	1	2	1	1	1	1	1
2	2	2	4	2	2	2	2	1	1
2	1	2	2	2	1	2	2	1	1
1	2	2	1	2	1	1	1	1	1
2	1	3	1	1	1	1	1	1	2
2	1	3	2	2	1	1	1	1	1
3	1	1	1	2	1	2	1	1	1
3	1	1	3	2	1	1	1	1	1
3	1	1	3	1	1	1	2	1	2
2	1	3	2	2	1	1	1	1	1
3	1	1	3	1	1	1	2	1	1
3	1	1	1	1	1	1	2	1	1
2	1	1	3	1	2	2	1	2	1
2	1	2	3	2	2	2	2	1	1
3	1	1	2	2	1	1	1	1	1
3	1	1	1	1	1	1	1	1	1
3	1	1	3	1	1	1	1	1	1
2	2	2	2	2	1	2	2	1	1
2	1	1	3	2	1	1	2	1	1
3	1	2	1	2	1	1	1	1	1
1	2	1	1	2	1	1	1	1	1
2	2	1	3	2	2	1	3	1	1
2	1	3	1	1	1	2	1	1	1
1	1	3	2	1	1	2	1	1	2
1	2	1	1	2	1	1	2	1	1
1	2	1	1	1	1	1	1	1	1
1	2	1	3	1	1	1	1	1	1

1	1	2	1	1	1	1	1	1	1
1	1	2	3	1	1	1	1	1	2
1	1	1	3	1	1	1	1	1	2
2	1	1	1	1	1	1	2	1	1
1	2	2	3	2	1	1	1	1	1
1	2	2	3	2	1	1	1	1	1
1	1	1	1	1	1	1	1	1	1
1	1	1	2	2	1	1	2	1	1
1	1	1	3	1	1	1	1	1	2
1	1	1	1	1	1	1	1	1	1
1	1	1	3	1	1	1	1	1	1
1	1	1	1	1	1	1	1	3	1
1	1	1	1	1	1	1	1	1	1
1	2	1	1	1	1	1	1	1	1
3	4	1	1	1	1	1	1	1	1
1	2	1	1	1	1	1	1	1	1
1	1	1	1	2	1	1	1	1	1
1	4	1	1	1	1	1	1	1	1
1	2	2	2	2	1	3	1	1	2
1	1	1	1	1	1	1	1	1	1
1	1	1	1	1	1	2	1	1	1
1	2	1	1	1	1	1	1	1	1
1	1	1	1	2	1	1	1	1	1
2	2	2	1	3	1	2	1	1	1
1	3	2	2	2	1	1	1	1	2
2	1	2	1	2	1	2	1	1	2
1	1	1	1	1	1	1	3	1	1
1	1	1	2	1	1	1	1	1	1
1	2	1	3	3	1	2	3	1	1
2	2	1	2	2	2	1	1	1	1
1	1	2	1	2	1	1	1	1	1
2	2	2	1	1	1	2	3	1	1
3	1	1	2	2	1	1	1	1	1
2	1	1	1	4	1	4	4	1	1
2	3	3	3	3	1	2	3	1	1
1	2	1	1	1	1	1	2	1	1
1	1	2	1	2	1	2	2	1	2
2	1	1	1	1	1	1	1	2	1
2	3	1	4	3	1	1	2	1	1
1	1	3	1	3	1	3	3	1	5
2	1	1	1	1	2	1	1	1	1
1	1	3	1	4	1	1	4	1	1
1	1	1	1	2	1	2	1	1	1
1	3	1	3	1	3	1	1	1	2
1	1	2	1	2	1	1	1	1	1
1	3	2	3	2	1	1	1	1	2
1	2	3	3	2	1	2	2	1	1

1	2	2	2	2	1	2	2	1	1
2	2	1	3	1	1	1	1	1	1
2	1	2	1	1	1	1	2	1	1
2	1	1	3	3	2	2	1	1	1
3	1	1	1	2	1	1	1	1	1
2	1	1	2	1	1	1	1	1	1
2	3	1	1	2	1	1	1	1	1
2	2	2	1	1	1	1	1	1	1
2	1	2	2	2	1	2	1	3	1
2	1	1	2	1	1	3	2	1	1
1	2	1	1	1	1	1	1	1	1
1	2	1	3	1	1	1	1	1	3
3	1	1	1	1	1	1	1	1	4
2	2	2	1	2	2	1	2	1	2
2	1	2	1	1	1	1	1	1	1
3	1	1	4	2	1	2	1	1	1
2	1	1	5	1	1	1	1	1	1
2	1	1	1	1	1	1	1	1	1
2	1	1	3	3	1	1	1	2	1
3	1	1	3	1	1	1	1	1	1
2	3	1	1	2	1	2	3	1	5
3	1	1	1	2	1	1	1	1	1
2	1	1	1	1	1	1	1	1	1
2	1	2	1	1	1	1	1	1	1
2	1	1	2	2	1	1	1	1	1
3	3	1	1	1	1	1	1	1	1
1	1	2	1	2	1	1	2	1	1
2	1	1	1	1	1	1	1	1	1
1	1	1	2	1	1	1	1	1	1
2	1	1	1	1	1	1	1	1	1
2	1	1	4	3	1	3	3	1	1
2	1	4	2	1	1	3	1	1	1
2	1	1	1	1	1	1	1	1	1
2	1	1	1	1	1	1	1	3	1
2	1	1	1	1	1	1	1	1	1
3	1	1	1	1	1	1	1	1	1
2	1	1	1	1	1	1	3	1	1
2	2	2	2	2	1	1	2	1	1
2	1	2	1	3	1	1	1	1	1
2	1	1	3	2	1	1	1	1	4
3	1	1	1	2	1	1	1	1	1
1	1	1	1	1	1	1	2	1	2
2	1	1	3	2	1	1	1	1	1
2	3	3	3	1	1	1	1	1	1
2	1	1	2	1	1	1	1	1	1
3	1	1	1	1	1	1	1	1	1
2	1	1	2	1	4	1	1	1	4

2	1	2	2	1	1	2	1	1	1
2	1	1	3	1	1	1	1	1	1
2	1	1	3	1	1	1	1	1	1
2	2	1	2	2	1	1	2	1	1
2	1	2	1	1	1	1	1	1	1
2	1	1	1	1	1	1	1	1	1
2	4	1	1	2	1	1	1	1	4
1	1	1	3	1	2	1	1	1	1
3	1	3	1	1	1	2	1	1	1
2	1	2	3	1	1	1	3	1	1
3	1	1	4	1	1	1	1	1	1
1	1	1	2	2	1	1	1	1	2
2	1	1	1	1	1	1	1	1	1
1	3	1	2	1	2	1	1	1	1
1	3	1	2	1	1	1	1	1	1
1	1	2	1	1	1	2	1	1	1
1	1	1	1	1	1	1	1	1	1
1	1	1	2	1	1	1	1	1	1
1	1	2	3	2	1	2	1	1	1
1	1	1	1	1	1	1	1	1	1
3	1	1	3	1	1	1	1	1	1
1	1	1	1	1	1	1	1	1	1
1	1	1	1	1	1	1	1	1	1
1	2	2	2	1	2	2	2	1	1
1	1	2	1	1	1	1	1	1	1
2	2	4	4	1	1	4	2	1	3
1	1	1	1	1	1	1	1	1	1
1	1	2	1	1	1	1	1	1	1
1	2	2	4	1	2	1	2	1	4
2	2	4	1	1	1	1	2	1	3
1	1	3	2	1	1	1	1	1	1
4	3	4	1	1	2	1	2	1	1
2	3	2	1	1	1	1	1	1	1
3	1	1	1	2	1	1	1	1	1
3	1	1	1	1	1	1	1	1	1
4	4	1	4	2	1	2	2	1	2
3	2	1	2	1	1	1	1	1	1
2	1	1	1	1	1	1	1	1	1
3	1	1	1	2	1	2	1	1	1
2	1	1	1	1	1	1	1	1	1
1	2	1	4	2	2	2	2	1	2
1	2	3	4	1	4	4	4	4	4
3	1	2	3	2	2	2	2	1	1
3	2	4	4	3	1	3	4	1	1
3	1	1	2	2	1	2	1	1	1
2	3	1	1	1	1	1	1	1	1
3	1	1	1	2	1	1	1	1	1

2	1	1	1	1	1	1	1	1	1
1	1	3	2	1	1	1	1	1	1
3	3	1	2	4	1	1	2	1	4
3	2	1	1	1	1	1	1	1	1
2	2	1	2	1	1	2	1	3	3
2	2	1	2	1	1	1	3	1	1
1	1	2	2	2	1	1	1	1	1
1	1	2	2	2	1	1	1	1	1
3	1	1	1	1	1	1	1	1	1
3	4	2	2	1	1	1	1	1	1
1	3	1	1	1	1	1	1	1	1
2	1	2	3	2	1	3	2	1	1
2	1	1	2	2	1	1	1	3	1
3	1	2	1	2	1	1	2	1	1
2	1	1	1	1	1	1	1	1	1
3	2	1	1	1	1	1	1	1	1
2	1	1	3	1	2	3	1	1	1
2	1	1	1	1	1	1	2	1	1
2	2	3	2	1	1	1	2	1	1
1	1	1	2	1	1	1	2	1	1
1	4	1	1	1	1	1	1	1	1
1	1	1	1	2	1	1	1	1	1
2	1	1	1	1	1	1	1	1	1
2	1	1	1	2	1	2	1	1	1
2	1	2	1	1	1	1	1	1	1
2	2	1	3	3	1	3	1	1	1
2	1	1	1	1	1	1	1	1	1
2	1	2	2	2	2	2	3	3	3
1	2	1	2	4	1	1	1	1	1
2	2	3	1	3	1	3	1	1	1
2	1	1	3	2	1	1	3	1	3
3	1	1	2	2	1	1	1	1	1
3	1	1	2	2	1	1	1	1	1
2	1	1	1	1	1	1	1	1	1
2	1	2	1	2	1	1	2	1	1
2	1	2	1	2	1	1	2	1	1
2	1	1	1	1	1	1	1	1	1
2	1	1	1	1	1	1	1	1	1
1	1	1	1	2	1	1	2	1	1
2	2	2	1	1	1	1	2	1	1
2	1	1	1	1	1	1	1	1	1
2	1	1	3	2	1	1	1	1	1
2	3	1	1	1	1	1	1	1	1
3	2	1	1	2	1	2	3	2	1
2	2	1	1	1	1	2	1	1	1
2	1	2	2	1	2	1	2	1	1
4	1	1	1	2	1	2	2	1	1

2	1	1	1	1	1	1	1	1	1
2	1	1	1	1	1	1	1	1	1
2	1	1	1	2	1	1	1	1	1
2	2	1	2	2	1	1	2	1	1
2	4	2	3	1	1	3	1	1	2
2	3	3	2	2	1	1	2	1	3
2	3	3	2	2	1	1	2	1	3
2	1	1	2	1	1	1	1	1	1
2	1	1	2	1	1	1	1	1	1
1	1	1	1	1	1	1	2	1	2
2	1	1	3	2	1	3	1	1	1
2	1	1	3	2	1	3	1	1	1
2	2	1	2	2	1	1	2	1	1
2	1	1	2	1	1	1	1	1	1
2	3	1	3	1	3	2	3	1	1
1	3	1	1	4	1	1	1	1	3
2	2	2	2	4	2	3	2	1	4
3	1	1	1	1	1	2	2	1	1
3	2	1	1	1	1	1	1	1	1
3	3	1	4	2	1	1	4	1	1
2	1	1	1	1	2	1	1	1	1
2	1	1	4	4	1	2	2	1	1
4	1	1	1	1	1	2	1	1	2
3	1	1	1	2	1	1	1	1	1
3	1	1	1	2	1	1	1	1	1
2	1	1	1	2	1	1	1	1	2
2	2	1	1	2	2	1	2	2	1
2	3	3	4	3	1	1	3	1	4
2	2	1	3	3	1	2	2	1	1
3	1	1	1	1	1	1	1	1	1
3	1	3	2	1	1	1	1	1	1
4	1	1	1	1	1	1	2	1	1
3	2	1	1	1	1	1	1	1	1
3	1	1	2	2	1	2	2	1	1
3	1	1	1	1	1	1	1	1	1
3	1	1	1	2	1	1	1	1	1
3	1	1	1	2	1	1	1	1	1
2	1	1	1	1	1	1	1	1	1
2	1	2	2	2	1	2	2	1	2
2	1	1	2	1	1	1	1	1	1
1	2	2	2	2		2	2	1	1
2	1	2	1	1	1	1	2	1	1
3	2	2	3	4	1	4	1	1	1
2	1	1	1	2	1	1	1	1	1
3	4	1	1	1	1	1	1	1	1
2	1	1	2	1	1	1	1	1	1
3	1	1	2	2	1	1	1	1	1

2	1	1	1	1	1	1	1	1	1
3	1	1	1	1	1	1	1	1	5
3	1	1	2	1	1	2	1	1	1
2	1	1	1	1	1	1	1	1	1
1	4	4	1	1	1	1	1	1	1
1	1	1	1	1	1	1	1	1	1
1	1	2	1	1	1	1	1	1	1
1	2	2	2	2	2	3	1	1	1
2	1	1	2	2	1	2	1	1	1
2	2	2	1	1	1	1	2	1	3
3	1	1	1	1	1	1	1	1	1
3	1	1	4	4	4	4	3	1	1
2	1	1	1	3	1	1	1	1	1
3	1	1	1	1	1	1	1	1	1
3	1	1	1	1	1	1	1	1	1
1	2	2	2	2	2	2	2	2	2
1	3	2	3	1	1	1	1	1	1
2	2	2	2	1	1	2	2	2	2
1	1	2	2	3	2	2	2	1	1
1	2	2	2	1	1	1	1	1	1
2	2	3	2	2	1	1	1	1	1
1	1	2	2	2	1	1	1	1	1
3	1	1	1	1	1	1	1	1	1
1	2	2	2	3	1	1	2	1	1
2	1	1	1	1	1	1	1	1	1
3	1	1	4	2	1	1	2	1	4
2	1	1	1	1	1	1	1	1	1
2	1	1	1	1	1	1	1	1	1
2	1	1	1	1	1	1	1	1	1
2	1	1	1	2	2	2	2	1	1
2	1	3	4	2	1	1	1	1	1
2	2	2	3	2	1	1	2	1	1
1	2	2	2	3	2	2	1	1	1
2	1	2	1	2	1	1	1	1	1
4	1	1	1	2	1	2	2	3	3
4	2	2	3	2	2	1	2	2	2
2	1	1	2	2	1	1	1	1	1
2	1	1	2	2	2	1	1	1	1
4	1	1	3	3	3	3	3	3	2
2	1	1	1	1	1	1	1	1	1
2	1	1	1	1	1	1	1	1	1
1	2	2	2	2	2	2	2	2	2
1	1	1	1	1	1	1	1	1	1
2	4	4	1	1	1	1	1	1	1
4	1	1	1	1	4	2	1	1	1
4	1	1	1	1	1	1	1	1	1
2	1	1	1	2	1	1	1	1	1

1	2	2	2	2	2	3	1	1	1
2	2	1	3	3	3	3	2	1	2
4	1	1	1	1	2	2	2	2	2
4	1	1	1	1	1	1	1	1	1
4	1	1	1	1	1	2	2	2	1
4	4	4	1	1	2	1	1	1	1
1	2	1	2	2	2	1	1	1	3
1	1	2	4	3	2	2	3	2	1
2	2	2	4	2	2	2	2	1	2
1	2	3	4	3	1	3	3	1	3
2	1	3	3	1	1	1	2	3	1
2	2	2	3	3	3	2	1	1	3
2	2	3	4	3	3	3	3	1	1
2	3	1	3	2	1	1	2	3	1
2	1	1	3	2	1	1	1	1	1
2	1	1	2	2	2	1	1	1	1
3	1	1	1	1	1	1	1	1	1
2	3	3	4	2	1	3	2	1	1
4	1	1	3	2	3	1	1	2	1
3	1	1	3	3	1	1	3	1	1
2	1	1	2	1	1	2	1	1	1
2	1	1	4	3	1	1	1	1	1
1	1	1	3	2	1	1	3	1	1
2	1	2	1	2	1	1	1	1	1
1	1	1	1	2	1	1	1	1	1
3	1	3	1	1	1	1	1	1	1
3	2	1	2	2	1	1	1	2	1
2	1	1	1	1	2	1	1	1	1
2	1	1	3	3	1	1	1	1	1
2	1	1	1	2	2	1	1	1	1
2	2	1	3	1	1	1	1	3	3
2	2	2	4	3	3	1	3	1	1
2	2	2	4	3	3	1	3	1	1
3	1	2	1	3	1	1	2	1	1
2	1	1	4	2	1	1	1	1	1
3	1	1	1	2	2	1	1	1	1
3	1	2	4	2	1	1	2	3	1
3	1	1	1	1	1	1	1	1	1
2	3	1	3	1	1	1	1	1	1
2	1	1	2	2	1	1	1	1	1
2	1	1	3	3	1	2	3	1	1
2	1	2	3	3	1	2	3	1	1
2	2	2	4	2	1	1	1	1	1
2	2	2	2	3	1	2	2	1	1
2	1	1	1	1	1	1	1	1	1
2	1	1	3	1	1	1	1	1	1
2	1	2	1	2	1	2	2	1	1

2	1	1	1	1	2	1	1	2	1
3	1	1	4	2	3	1	2	3	1
1	1	1	2	1	1	1	1	1	1
3	3	1	3	3	1	2	4	1	1
4	1	1	1	1	1	1	1	1	1
1	1	1	3	2	2	1	1	1	1
4	1	1	1	1	1	1	1	1	1
1	2	1	4	3	1	1	1	1	1
1	1	2	1	2	1	1	1	1	1
1	1	1	1	1	1	1	1	1	1
2	2	1	1	1	1	2	1	1	1
1	3	1	1	1	1	2	1	1	1
2	2	1	2	1	1	1	1	1	3
2	1	2	1	1	1	1	1	1	1
1	1	2	2	1	1	1	1	1	1
2	2	1	1	1	1	1	1	1	1
1	1	2	1	2	2	1	1	2	5
3	1	4	1	1	1	1	1	1	1
4	1	1	2	1	1	1	1	1	1
4	1	1	2	1	1	1	1	1	1
4	1	2	2	1	1	1	1	1	1
2	1	1	1	1	1	1	1	1	1
2	1	1	3	2	1	1	2	1	1
2	2	2	3	2	1	2	1	1	2
1	2	1	2	2	1	1	1	1	1
2	1	1	2	1	1	1	1	1	1
3	1	1	2	1	1	1	1	1	1
2	1	1	1	1	1	1	1	1	1
1	1	1	2	1	1	1	1	1	1
2	1	2	1	1	1	1	1	1	1
1	1	1	1	1	1	1	1	1	1
3	1	2	5	2	1	1	1	1	1
3	1	1	1	1	1	1	1	1	1
2	1	2	1	1	1	1	1	1	1
1	1	1	2	2	1	2	1	1	1
2	1	1	1	1	1	1	1	1	1
2	1	1	2	1	1	1	1	1	1
2	1	1	3	1	1	1	3	2	1
3	1	1	1	1	1	1	1	1	1
2	2	1	1	1	1	1	1	1	1
2	2	1	1	1	1	1	1	1	1
3	1	2	1	1	1	1	2	1	1
1	1	1	1	1	1	1	1	1	1
1	1	1	3	1	1	2	1	1	1
4	1	1	2	1	1	1	1	1	1
1	1	1	1	1	1	1	1	1	1
1	2	1	2	1	1	1	1	1	1

2	2	2	2	1	1	1	2	1	1
2	2	2	2	1	1	1	2	1	1
3	2	1	1	1	1	1	1	1	1
1	3	1	3	3	1	3	1	1	1
2	2	3	4	3	1	2	1	1	1
1	4	4	2	2	1	1	2	2	1
1	1	2	2	2	2	2	2	2	1
1	1	2	2	2	1	1	1	1	1
2	3	1	5	1	1	1	3	2	1
1	1	1	2	1	1	2	1	1	1
1	1	1	4	1	1	1	1	1	1
1	1	1	4	1	1	1	3	1	1
4	1	1	1	1	1	1	1	1	1
3	1	1	1	1	1	1	2	1	1
3	1	1	1	1	1	1	2	1	1
3	2	1	1	1	1	1	2	1	1
2	1	1	2	2	1	2	2	1	1
2	2	2	2	3	1	1	1	1	1
3	1	1	1	1	1	1	1	1	1
4	2	2	2	3	2	1	2	3	3
2	1	2	1	1	1	1	1	1	1
2	1	1	2	2	1	1	2	1	1
2	1	1	1	2	1	1	2	1	2
3	4	1	1	4	1	4	1	1	1
3	1	2	4	1	1	2	1	1	1
2	1	3	1	1	1	2	2	1	1
3	1	1	2	2	1	1	2	1	1
1	2	1	4	3	1	1	1	1	1
3	1	2	2	2	2	2	2	1	2
3	2	1	1	3	1	1	1	1	1
3	1	1	1	2	1	2	2	1	1
2	1	2	2	1	1	1	1	1	2
3	2	1	1	2	1	2	3	1	1
2	2	3	1	1	1	1	2	1	1
2	1	2	2	2	1	1	1	1	2
1	1	1	1	2	1	1	1	1	1
3	1	1	1	1	1	1	1	1	1
3	1	1	2	1	1	1	2	1	1
3	1	1	2	2	1	2	2	1	1
2	1	1	1	2	2	1	1	1	1
1	2	1	1	1	1	1	1	1	1
2	2	1	1	2	1	1	2	1	1
2	1	1	3	2	1	2	2	1	2
3	1	1	1	1	1	1	1	1	1
1	2	3	2	2	1	1	2	1	3
2	2	2	2	2	1	2	1	1	3
1	1	4	4	4	4	1	4	1	1

1	1	4	4	4	4	1	4	1	1
2	2	2	1	2	1	1	2	1	1
2	2	2	4	4	1	3	4	1	1
4	1	2	2	1	2	2	1	2	1
4	3	3	3	1	1	1	2	1	1
2	1	1	1	2	1	1	1	1	1
2	1	1	1	2	1	1	2	1	2
2	1	1	1	2	1	1	2	1	2
1	1	1	1	1	1	3	1	1	1
1	2	2	3	2	1	1	2	1	2
2	2	1	3	3	2	1	2	1	1
2	2	3	3	1	1	1	1	1	1
3	1	1	1	2	1	1	4	1	2
3	2	1	2	1	1	1	2	1	4
2	4	3	2	2	1	1	3	1	
1	1	1	1	1	1	1	2	1	4
2	1	2	1	1	1	1	1	1	4
2	1	1	2	1	1	1	1	1	1
2	3	2	2	3	2	2	2	1	1
1	1	2	3	2	1	1	2	1	1
3	3	3	2	3	2	1	2	1	1
2	1	1	2	1	1	1	1	1	1
2	1	1	2	1	1	2	1	1	1
2	1	2	2	2	1	2	2	1	1
2	2	2	3	2	1	1	3	1	2
1	2	1	4	1	2	1	2	1	1
3	1	1	3	1	1	1	2	1	1
3	1	1	1	1	1	1	1	1	1
1	2	2	1	1	1	1	2	1	2
1	2	2	3	1	2	1	1	1	1
3	1	1	3	1	1	3	2	1	1
2	1	2	1	1	1	1	1	1	2
2	3	2	1	3	1	1	1	2	1
1	2	2	1	1	1	1	1	1	1
2	1	1	2	1	1	2	1	1	1
3	1	1	1	2	1	2	2	1	1
3	1	1	1	1	1	1	1	1	1
3	1	1	2	1	1	1	1	1	1
4	1	1	1	1	1	1	2	1	1
3	1	4	1	1	1	1	1	1	1
2	1	1	2	2	1	1	1	1	1
2	1	1	1	2	1	1	1	1	1
3	1	1	1	2	1	1	1	1	1
2	4	1	2	2	1	1	1	1	1
2	2	1	1	2	1	1	1	1	1
2	2	1	4	2	2	2	2	1	2
2	2	1	4	1	1	1	1	1	1

3	1	2	1	2	1	1	1	1	1
1	1	2	5	2	1	1	1	1	1
2	1	1	1	1	1	2	2	1	2
3	1	1	1	2	1	1	1	1	1
1	2	2	1	1	1	1	1	1	1
2	2	2	4	2	1	1	1	1	1
2	1	2	1	2	1	1	2	1	1
2	1	1	3	1	1	1	1	1	1
2	2	1	1	1	2	1	1	1	1
3	1	1	2	2	1	1	1	1	1
2	2	2	3	2	1	1	2	1	3
2	1	1	2	2	1	1	1	1	1
2	3	1	1	2	1	1	1	1	1
3	1	1	1	2	1	1	1	1	1
1	1	2	1	1	1	2	2	1	1
2	2	2	3	2	1	2	2	2	1
4	1	1	1	2	1	1	2	1	1
2	2	1	3	1	2	1	2	1	1
3	1	1	1	1	1	1	1	1	2
3	1	1	1	1	1	1	1	1	1
1	2	1	1	1	1	1	1	1	1
2	4	3	3	2	1	1	2	1	1
2	3	1	1	2	1	2	1	1	1
3	1	1	2	1	1	1	1	1	1
2	1	2	2	2	1	1	1	1	1
2	2	1	3	2	2	1	2	1	1
2	1	1	1	2	1	1	2	1	1
3	1	1	1	1	1	1	2	1	1
2	2	1	3	2	1	1	1	1	1
2	1	1	2	2	1	3	1	1	1
3	2	4	1	3	2	1	1	1	1
2	2	2	3	2	2	2	2	2	1
3	3	3	1	1	1	1	1	1	1
1	2	1	1	1	1	1	1	1	1
3	1	1	1	2	1	1	1	1	1
2	2	1	1	2	1	1	2	1	1
2	1	2	3	2	1	1	1	1	1
2	1	2	1	2	1	1	2	1	1
3	1	1	3	1	1	1	1	1	1
2	2	1	3	3	1	1	1	1	1
3	1	1	1	1	1	1	2	1	1
3	1	1	3	2	1	1	1	1	1
2	1	1	2	2	1	2	2	1	1
2	1	1	3	1	1	1	2	1	1
3	1	1	3	2	1	1	2	1	1
3	1	1	3	2	1	1	1	1	1
3	1	1	2	2	1	1	1	1	1

3	1	1	3	2	1	2	1	1	1
3	1	2	2	3	2	2	2	1	1
3	1	1	2	2	1	1	1	1	1
2	1	1	1	1	1	1	1	1	1
2	2	2	2	2	1	1	2	1	1
2	1	3	3	2	1	2	1	2	1
2	2	1	2	1	1	1	2	1	1
3	3	1	1	2	2	1	1	1	1
2	2	2	1	1	1	1	1	1	1
1	1	3	1	2	1	2	2	1	2
2	1	1	2	3	1	1	1	1	1
1	4	1	1	1	1	1	1	1	1
1	4	1	1	1	1	1	1	1	1
2	2	1	3	1	1	1	1	1	1
1	1	1	1	1	1	1	1	1	1
3	1	1	1	1	1	1	1	1	1
1	1	1	1	1	1	1	1	1	1
1	1	1	1	1	1	1	1	1	1
1	1	1	1	1	1	1	1	1	1
1	1	1	3	1	1	1	1	1	1
1	1	1	3	1	1	1	1	1	1
2	1	1	4	1	1	1	1	1	1
1	1	1	1	2	1	1	2	1	1
1	3	1	1	1	1	1	1	1	1
1	1	2	2	2	1	1	1	1	2
1	2	1	3	1	1	1	1	1	1
2	1	1	1	1	1	1	1	1	1
3	1	1	4	1	1	1	1	1	1
3	1	1	1	1	1	1	1	1	1
3	1	1	1	1	1	1	1	1	1
1	1	1	1	1	1	1	1	1	1
1	4	1	1	1	2	2	2	2	2
4	1	1	1	1	1	1	1	1	1
2	2	2	1	1	1	1	1	2	1
1	2	1	2	2	1	2	2	1	1
1	1	1	1	1	1	1	1	1	1
1	1	1	1	1	1	1	1	1	1
1	1	1	1	1	1	1	1	1	2
1	1	1	1	1	1	1	3	1	1
2	1	1	1	1	1	1	2	1	1
1	1	3	2	1	1	3	1	2	1
2	1	1	1	1	1	2	2	1	1
1	1	2	3	3	2	1	2	1	2
1	4	4	2	2	1	1	1	1	1
1	2	2	2	1	1	1	1	1	1
1	1	1	4	4	1	1	4	1	1
1	2	3	1	2	1	1	1	1	3

1	3	1	3	3	3	2	3	1	3
1	2	1	4	2	2	1	2	1	1
1	1	3	3	2	2	2	1	2	2
1	1	2	2	1	1	1	1	1	1
1	2	1	2	1	1	1	1	1	1
1	1	1	1	1	1	1	1	1	1
1	1	1	4	1	2	1	1	1	3
1	1	1	1	1	1	1	1	1	1
1	2	4	1	3	4	2	2	1	2
3	1	1	1	2	1	1	1	1	1
1	3	3	3	2	2	2	3	2	3
2	1	2	2	2	1	1	1	1	1
1	1	1	1	1	1	1	1	1	1
2	1	1	1	1	1	1	1	2	1
2	1	1	1	1	1	1	3	1	1
1	1	2	2	2	2	2	2	1	2
2	1	1	1	3	1	1	1	1	1
1	1	1	3	3	1	3	1	1	1
2	2	3	1	1	1	2	1	1	2
3	1	1	1	1	1	1	1	1	4
3	1	1	3	2	1	1	1	1	1
3	1	1	1	2	1	2	2	1	1
3	1	1	1	1	1	2	1	1	1
2	1	1	1	2	1	2	1	1	1
2	1	1	2	2	1	1	1	1	1
2	1	2	1	1	2	1	1	1	1
3	1	1	1	1	1	2	1	1	1
3	1	1	1	1	1	2	1	1	1
2	1	1	2	1	1	1	1	1	1
2	1	1	2	1	1	1	1	1	1
2	1	1	1	2	1	1	1	1	1
2	1	1	1	1	1	1	2	1	1
3	1	1	1	1	1	1	1	1	1
2	2	1	2	2	1	1	1	1	1
3	1	1	1	1	1	1	1	1	1
3	1	1	1	1	1	1	1	1	1
3	1	2	2	3	1	2	1	1	1
4	1	2	1	2	1	2	1	1	1
1	1	1	3	2	1	2	1	1	1
3	1	1	1	1	1	1	1	1	1
2	1	1	1	1	1	1	1	1	1
2	1	1	1	1	1	1	1	1	1
3	1	1	1	2	1	1	1	1	1
3	1	2	2	1	1	1	1	1	1
2	2	1	1	1	1	2	2	1	1
2	2	1	1	1	1	2	2	1	1
2	1	1	1	1	1	1	1	1	1

3	1	2	1	1	1	1	1	1	1
3	1	2	1	2	1	2	1	1	1
3	1	2	1	2	1	1	2	1	1
1	1	2	2	2	1	1	1	1	1
2	2	2	1	3	1	3	3	3	4
1	1	1	1	1	1	1	1	1	1
2	2	2	3	3	1	3	3	1	2
2	1	2	1	1	1	1	1	1	1
2	2	1	1	1	1	1	2	1	1
2	2	1	4	3	2	1	1	1	1
2	1	2	1	1	1	1	1	1	1
1	2	2	3	3	1	3	3	1	3
2	1	1	1	2	1	1	1	1	1
2	1	1	1	2	1	1	1	1	1
3	1	1	1	1	1	1	1	1	1
2	1	1	1	1	1	3	1	1	1
2	1	1	2	1	1	1	1	1	1
2	3	1	2	1	1	1	1	1	1
2	1	1	1	1	1	1	1	1	1
2	2	1	2	2	1	1	1	1	1
2	2	3	1	2	1	1	1	1	1
2	1	2	1	2	1	1	1	1	1
3	1	1	1	1	1	2	1	1	1
2	1	2	1	3	1	1	1	1	1
3	1	1	1	3	2	1	1	2	1
3	1	1	1	1	1	1	1	1	1
2	1	1	2	1	1	1	1	1	4
2	1	1	2	1	1	1	1	1	1
3	1	1	2	3	1	1	3	1	1
3	1	1	1	1	1	1	1	1	1
3	1	1	1	2	1	3	1	1	1
2	2	2	1	1	1	1	1	1	1
3	1	2	1	2	1	1	2	1	5
1	3	3	3	3	3	2	3	3	1
3	1	1	1	1	1	1	1	1	5
3	2	2	2	2	1	1	1	1	1
2	1	1	1	1	1	1	1	1	1
1	4	4	3	2	1	3	3	1	4
2	1	1	1	1	2	1	1	1	1
2	3	3	3	3	1	1	3	1	3
2	4	1	3	1	1	1	1	1	1
3	2	1	1	2	1	1	2	1	1
3	4	3	3	2	2	1	3	3	3
2	1	1	1	1	1	1	1	1	4
2	2	2	2	2	1	1	1	1	2
2	2	2	1	1	1	1	1	1	1
3	2	1	1	1	1	1	2	1	1

1	1	1	2	1	2	1	1	1	1
1	1	1	1	1	2	1	1	1	1
1	1	2	1	1	1	1	1	1	1
3	1	1	1	1	1	1	1	1	1
1	1	2	1	1	1	1	1	1	1
4	1	1	1	1	2	1	1	1	1
1	1	2	4	1	1	2	1	1	5
1	1	1	2	1	1	1	1	1	1
1	2	2	2	1	1	1	1	1	1
3	1	2	1	1	1	1	2	1	1
1	1	2	1	1	1	1	1	1	1
2	1	1	2	1	1	1	1	1	1
1	1	1	1	1	2	1	1	1	1
1	1	1	1	1	1	1	2	1	2
1	4	1	1	2	1	1	1	1	1
2	1	1	1	4	1	1	3	1	1
1	1	1	4	1	3	1	1	1	1
1	1	1	1	1	1	1	1	1	1
1	1	3	1	1	1	2	2	2	3
2	1	1	1	2	1	1	1	1	1
1	1	1	1	1	1	1	1	1	1
3	1	1	1	2	1	1	1	1	2
1	1	1	3	1	1	1	4	1	1
3	1	1	1	1	1	1	1	1	1
2	1	1	1	1	1	1	1	1	1
3	1	1	2	1	1	1	1	1	1
2	2	1	1	2	1	1	1	1	1
2	2	1	1	2	1	2	1	1	1
3	2	1	1	1	1	2	2	1	1
3	2	1	1	1	1	2	2	1	1
2	1	1	2	1	1	1	1	1	1
2	2	1	2	2	1	1	1		4
2	1	1	1	1	1	1	1	1	1
3	1	1	3	2	1	1	1	1	2
3	2	2	2	2	1	2	2	1	1
1	1	2	3	2	1	1	1	1	1
3	3	1	1	1	1	1	2	1	1
3	3	1	1	1	1	1	2	1	1
3	4	1	1	1	1	1	1	1	1
1	1	1	1	2	1	1	1	1	1
1	1	1	4	1	1	1	3	1	1
2	1	1	1	1	1	1	1	1	1
2	1	1	4	1	1	1	1	1	2
2	1	1	2	1	1	1	1	1	1
2	2	3	3	2	2	3	2	2	2
2	2	3	3	2	2	3	2	2	2
2	1	1	1	1	1	1	1	1	1

2	1	1	1	1	1	1	1	1	1
2	1	2	1	1	1	1	1	1	1
1	2	3	4	1	1	1	1	1	1
2	1	1	1	1	1	1	1	1	1
2	1	1	2	3	1	1	1	1	1
1	2	2	3	1	1	1	1	1	1
2	1	3	1	1	1	1	1	1	1
2	1	1	1	1	1	1	1	1	1
2	2	2	1	1	1	1	2	1	1
1	1	1	2	1	1	2	1	2	1
2	1	2	2	1	1	2	1	1	1
2	1	2	2	1	1	2	1	1	1
1	2	1	1	1	1	1	1	1	1
2	1	1	1	1	1	1	1	1	1
2	2	1	1	2	1	2	3	1	3
2	2	3	4	2	1	2	2	3	2
2	2	2	1	1	1	1	1	1	1
2	1	1	1	1	1	1	1	1	1
3	1	3	1	1	1	1	1	1	1
1	1	2	4	2	1	2	2	1	1
4	3	1	2	2	1	1	2	1	1
2	1	1	2	1	1	1	1	1	1
2	1	1	2	2	2	1	1	3	1
2	2	1	1	2	2	1	1	1	1
2	4	1	2	1	1	3	4	1	5
4	1	1	1	3	1	1	1	1	1
2	2	1	2	2	2	1	1	2	1
2	2	1	3	1	1	1	2	1	1
2	2	1	3	1	1	1	2	1	1
2	1	1	1	2	1	2	3	1	2
4	2	1	1	2	2	1	3	1	1
1	2	3	4	3	1	2	4	1	3
1	1	1	2	1	1	2	2	1	2
2	1	2	3	2	2	1	1	1	1
3	2	2	2	1	1	1	2	1	1
1	3	1	4	1	1	1	2	1	1
4	1	1	2	2	3	2	2	3	1
2	3	3	3	2	1	3	2	1	2
1	2	1	3	1	1	1	1	1	1
3	1	1	1	1	1	2	1	1	1
3	3	3	3	1	2	2	3		4
4	1	1	2	2	2	4	2	1	2
2	2	2	1	1	1	1	1	1	2
2	4	1	3	2	2	2	1	1	1
2	4	1	3	2	2	2	1	1	1
2	1	1	1	2	1	2	2	1	1
3	2	1	2	2	1	1	1	1	1

2	1	2	1	2	1	1	1	1	1
2	2	2	2	2	1	2	1	2	1
3	1	1	2	2	1	3	1	2	1
4	1	1	2	2	1	1	1	1	1
3	1	1	1	2	1	1	1	1	1
3	1	1	1	2	1	1	1	1	1
4	1	3	2	2	2	2	3	1	2
4	1	3	2	2	2	2	3	1	2
4	1	3	2	2	2	2	3	1	2
2	2	2	2	1	1	1	1	1	1
2	1	2	2	2	2		1	1	1
2	1	1	1	1	1	1	1	1	1
2	1	3	2	1	1	1	1	1	1
3	1	1	1	1	1	1	1	1	1
3	1	2	1	2	1	1	2	1	1
2	1	1	1	1	1	1	1	1	1
4	2	2	2	2	2	3	3	3	3
2	1	1	1	1	1	1	1	1	1
1	1	1	1	1	1	1	1	1	1
1	2	2	3	1	1	1	1	1	1
1	1	1	1	3	2	2	1	1	1
1	1	3	1	2	1	1	1	1	1
2	2	1	1	1	1	2	2	1	2
2	2	2	2	2	1	1	1	1	1
2	1	2	1	2	1	2	1	1	1
1	1	1	1	1	1	1	1	1	1
1	1	1	1	1	1	1	1	1	1
2	1	1	1	2	1	1	1	1	1
2	1	1	1	2	1	2	2	1	2
2	1	1	1	1	1	2	2	2	1
3	1	1	1	1	1	1	1	1	1
2	1	1	1	1	1	1	1	1	1
4	1	1	1	1	1	1	2	2	2
1	2	3	3	3	2	1	1		1
2	1	1	1	1	1	1	1	1	1
3	1	2	1	2	1	1	2	1	2
2	1	1	2	3	1	1	1	1	2
2	1	1	1	1	1	1	1	1	1
2	1	1	1	1	1	1	1	1	1
1	1	1	1	1	1	1	1	1	1
1	2	2	2	2	2	2	2	2	2
2	1	1	1	1	1	1	1	1	1
4	1	1	1	1	1	1	1	1	1
1	1	1	1	1	1	1	2	1	2
4	1	1	2	2	2	2	2	2	2
1	1	1	1	1	1	1	2	1	1
1	1	1	1	1	1	1	1	1	1

1	1	1	1	1	1	1	1	1	1
3	1	1	2	1	1	2	2	1	1
1	1	2	2	2	1	1	1	1	1
1	1	1	1	1	1	1	1	1	1
1	1	1	1	1	1	1	2	1	1
1	1	1	1	1	1	1	2	1	2
1	1	1	1	1	1	1	1	1	1
4	1	1	1	1	1	1	1	1	1
2	2	3	2	3	2	2	1	1	1
2	1	1	2	2	1	1	2	1	1
3	1	1	1	2	1	1	1	1	1
2	1	1	1	1	1	1	1	1	1
1	2	2	2	2	2	2	2	2	2
2	1	1	1	1	1	1	1	1	1
3	1	1	1	1	1	1	1	1	1
2	1	1	1	1	1	1	1	1	1
4	1	1	1	1	1	3	3	1	1
1	1	1	1	1	2	2	1	2	1
1	1	1	1	1	1	1	1	1	1
1	1	1	1	1	1	1	1	1	1
3	2	2	3	3	1	4	3	1	3
3	1	1	1	1	1	1	1	1	1
3	1	1	1	1	2	1	1	1	2
1	2	2	3	2	1	1	2	1	2
4	1	1	3	2	1	3	3	1	1
1	1	1	1	1	2	1	1	2	1
2	1	1	1	1	1	2	2	1	2
2	2	1	1	1	1	1	1	1	1
1	1	1	1	1	1	1	1	1	1
4	1	1	1	2	2	2	2	1	1
2	1	1	2	2	1	2	1	1	2
3	4	4	4	4	4	4	4	3	3
1	1	1	1	1	1	1	1	2	1
2	1	1	1	1	1	1	1	1	1
1	2	2	2	2	2	2	2	1	1
4	1	1	1	1	1	2	2	2	2
3	2	2	2	2	1	1	1	1	2
1	1	1	1	1	1	1	1	1	1
4	1	1	1	1	1	1	1	1	1
1	1	2	1	1	1	1	1	1	1
4	1	1	1	1	1	1	1	1	1
	4	4	4	4	4	4	4	4	4
2	1	1	1	2	1	3	1	1	1
2	2	2	2	3	2	1	1	1	4
4	2	2	2	2	1	1	1	1	1
2	2	1	4	3	2	3	1	1	1
2	1	1	2	1	1	1	2	1	1

1	1	2	4	3	1	3	3	1	1
2	3	3	3	3	1	3	3	1	3
2	2	1	4	4	1	2	2	1	1
2	1	1	2	2	1	2	2	1	1
1	2	1	3	2	2	1	1	1	1
1	1	2	3	3	2	3	2	1	1
1	1	2	2	2	2	3	3	1	1
3	1	1	1	1	1	1	1	1	1
1	1	2	3	3	1	1	3	1	3
3	1	2	1	1	1	2	2	1	1
2	2	3	2	2	1	1	3	1	1
2	2	3	2	2	1	1	3	1	1
1	2	3	1	2	1	1	1	1	1
1	2	3	1	2	1	1	1	1	1
2	1	1	1	2	3	2	3	2	2
1	1	1	3	3	1	3	3	1	1
2	1	2	1	1	1	1	2	2	1
2	1	1	3	2	1	1	2	1	2
2	2	1	4	3	1	1	2	1	1
4	1	1	2	1	1	1	1	1	1
2	2	3	1	2	1	2	1	1	5
2	1	2	3	3	1	3	2	1	1
2	1	1	2	1	1	1	1	1	1
1	1	2	3	3	2	2	3	1	1
2	1	1	2	1	1	1	1	1	1
1	1	2	3	4	1	1	1	2	1
2	1	1	4	1	1	2	1	1	1
2	1	3	2	1	1	1	1	1	1
2	2	2	2	3	3	1	3	1	2
2	2	2	2	3	3	1	3	1	2
1	1	1	1	3	1	1	3	1	1
2	1	1	2	3	1	1	3	1	1
3	1	2	4	4	1	2	1	1	1
2	1	1	1	1	1	1	2	1	1
3	2	2	3	1	1	1	2	1	2
2	2	1	4	3	2	1	1	1	1
2	2	1	4	3	1	1	1	1	1
2	1	1	3	3	1	2	2	1	1
2	1	1	3	3	1	3	3	1	1
1	1	3	3	3	1	2	3	1	4
1	1	3	3	3	1	2	3	1	4
2	1	1	2	1	1	1	1	1	1
1	1	2	1	1	1	1	1	1	1
4	1	3	4	1	2	1	2	1	1
1	1	1	2	3	1	1	3	1	1
2	1	1	3	1	1	1	1	1	1
4	1	1	2	2	1	1	1	1	1

1	1	2	1	1	1	1	1	1	1
3	1	1	1	1	1	1	1	1	1
2	1	1	4	1	1	1	1	1	1
1	1	1	2	2	1	1	1	1	1
3	2	1	1	2	1	1	1	1	1
1	2	2	2	2	1	3	2	1	1
4	1	1	2	1	1	1	1	1	1
1	1	1	1	1	1	1	1	1	1
4	2	1	1	3	1	1	2	1	1
2	2	1	2	1	1	1	1	1	1
2	2	1	2	1	1	1	1	1	1
1	2	1	3	2	2	1	3	1	1
2	1	1	1	1	1	1	1	1	1
2	2	4	4	3	2	1	1	1	5
1	2	1	1	1	1	1	1	1	1
1	2	1	1	1	1	1	1	1	1
3	2	1	1	1	1	1	1	1	1
1	2	3	3	2	1	1	1	1	1
1	1	1	3	1	2	1	2	1	1
1	1	1	4	1	1	1	1	1	1
2	1	1	1	1	1	1	1	1	1
3	1	1	2	1	1	1	2	1	1
2	3	2	4	1	2	1	2	1	1
1	2	2	2	2	2	1	2	1	1
4	1	1	2	1	1	1	1	1	1
4	1	1	2	1	1	1	1	1	1
2	2	2	1	1	1	1	1	1	1
1	1	1	2	1	1	1	1	1	1
1	3	1	3	1	1	1	1	1	1
1	1	3	4	1	1	1	1	1	1
4	1	1	1	1	1	1	1	1	1
3	1	1	3	1	3	1	1	1	1
1	1	1	4	2	1	1	1	1	1
2	1	1	1	1	1	1	1	1	1
2	1	1	3	2	1	1	1	1	1
3	1	2	4	1	1	1	2	1	2
3	2	1	2	1	1	1	1	1	1
2	1	1	3	2	2	2	2	1	1
1	2	1	2	2	1	1	2	1	3
1	2	1	2	2	1	1	2	1	3
2	2	2	2	1	1	1	1	1	1
3	1	2	1	4	1	1	2	1	1
3	1	1	2	2	1	1	2	1	1
2	1	2	1	2	1	1	2	1	1
2	2	2	2	2	1	1	1	1	4
2	2	2	2	2	1	1	1	1	4
3	1	1	1	1	1	1	1	1	1

2	2	3	3	3	2	2	2	2	1
2	4	1	3	4	1	1	3	1	3
3	1	1	1	1	1	1	1	1	1
2	2	2	1	1	2	1	2	2	2
2	2	2	2	2	1	1	2	1	2
3	1	2	2	2	2	1	1	2	1
2	1	2	4	3	1	3	2	1	1
2	1	2	2	2	1	3	2	3	1
3	1	2	1	3	1	1	1	1	1
2	1	1	4	3	1	1	3	3	1
1	1	4	1	1	1	1	1	1	1
2	1	1	2	2	1	1	2	1	1
2	1	2	2	2	1	2	1	1	1
1	1	1	1	1	1	1	1	1	1
3	1	1	1	2	1	1	2	1	1
3	1	1	1	1	1	1	1	1	1
2	1	1	1	1	1	1	1	1	1
1	2	2	1	1	1	1	1	1	1
2	1	1	1	1	1	1	1	1	1
3	1	1	1	2	1	2	2	1	1
2	1	1	1	2	1	2	2	1	1
1	1	1	1	1	1	1	2	1	1
1	1	1	1	1	1	1	2	1	1
2	3	3	1	1	1	1	1	1	1
2	3	3	1	1	1	1	1	1	1
2	2	2	2	1	1	1	1	1	2
2	2	2	2	2	1	2	2	1	1
3	2	1	2	2	1	1	3	1	2
2	2	1	2	1	3	1	1	1	3
2	2	1	2	1	3	1	1	1	3
2	2	2	1	2	1	1	2	1	2
2	2	2	1	2	1	1	2	1	2
2	2	1	4	2	1	1	1	1	1
2	2	3	1	1	1	1	2	1	3
2	1	1	1	1	1	1	1	1	1
2	1	1	1	3	1	1	1	1	1
2	1	2	2	2	1	2	2	1	4
1	1	1	1	2	1	1	1	1	2
2	1	1	2	1	1	1	1	1	1
3	1	1	2	2	2	1	2	1	1
2	1	2	1	1	1	1	1	1	1
3	1	2	1	2	1	1	1	1	1
2	2	1	2	2	1	2	1	1	1
2	1	2	1	2	1	1	1	1	1
3	3	1	2	2	1	1	1	1	2
2	1	2	1	2	1	1	1	1	1
3	1	2	2	2	1	2	2	1	1

1	1	1	3	1	1	2	2	1	1
2	1	1	3	1	1	2	3	1	1
2	1	1	2	2	1	2	1	1	1
2	3	3	5	2	1	2	3	1	1
2	1	3	2	2	1	2	2	1	2
3	1	1	2	1	2	1	1	2	1
2	1	2	2	1	1	1	1	1	1
2	2	2	3	2	1	2	2	1	1
2	2	2	3	1	1	1	2	1	1
2	2	2	3	3	1	1	1	2	2
2	1	1	1	1	1	1	2	1	1
1	1	1	4	2	1	1	1	1	1
2	1	1	1	1	1	1	2	1	1
2	1	1	3	1	1	1	1	1	1
2	3	2	3	1	1	1	1	1	5
3	1	1	4	1	1	1	2	1	1
3	2	1	1	2	1	1	1	1	2
3	2	1	2	2	1	1	1	1	2
2	2	1	1	1	1	1	1	1	1
2	2	1	2	2	1	1	2	1	1
1	3	1	2	1	1	1	1	1	1
2	1	1	1	2	1	1	1	1	1
3	1	1	1	1	1	1	1	1	1
3	2	1	2	2	1	1	1	1	1
1	1	2	1	1	1	1	1	1	1
3	1	1	1	1	1	1	1	1	1
2	1	1	1	1	1	1	1	1	1
3	1	1	1	1	1	1	1	1	1
2	1	2	3	2	1	2	2	1	1
3	1	1	1	1	1	1	1	1	1
2	2	2	2	2	1	1	1	1	1
3	2	1	3	2	2	1	3	1	1
2	1	1	1	1	2	1	2	1	1
3	1	1	1	1	1	1	1	1	1
2	1	1	1	2	1	1	2	1	1
2	1	1	1	2	1	1	1	1	1
3	2	1	3	2	1	1	1	1	1
2	1	1	1	1	1	1	1	1	1
2	2	2	5	1	1	1	1	1	1
4	3	2	1	3	1	1	1	1	5
2	2	1	1	1	1	1	2	2	1
3	1	1	2	1	1	1	1	1	1
3	1	1	1	1	2	2	3	1	1
3	1	1	2	2	1	1	2	1	1
2	1	1	1	2	1	1	1	1	1
2	3	1	3	2	1	1	1	1	1
1	2	1	4	2	2	1	1	2	1

2	2	2	2	1	1	1	1	1	1
1	1	1	1	1	1	1	1	1	1
1	2	2	2	2	2	2	1	1	1
2	1	1	1	1	1	1	1	1	1
1	2	1	1	1	1	1	1	1	1
1	2	1	1	1	1	1	1	1	1
1	4	1	1	1	1	1	1	1	1
3	1	1	1	2	1	1	1	1	1
1	1	1	4	1	1	1	2	1	1
2	2	1	1	1	1	1	1	1	1
3	4	1	1	1	1	1	1	1	1
1	2	1	1	1	1	1	1	1	1
1	2	1	2	1	1	1	1	1	1
1	4	1	1	1	1	1	1	1	1
1	2	2	3	1	1	1	1	1	2
1	2	1	3	2	1	3	2	1	1
1	1	1	1	1	1	1	1	1	1
1	2	1	1	1	1	1	1	1	1
1	1	2	3	1	1	1	1	1	2
1	1	2	1	1	2	1	1	2	1
1	1	1	3	2	1	3	1	1	1
2	2	2	2	1	1	1	1	1	1
1	2	1	1	1	1	1	1	1	1
1	3	2	4	1	4	1	3	1	1
1	1	1	1	1	1	1	1	1	1
2	3	1	4	2	3	2	1	1	4
3	1	2	3	3	3	2	3	2	3
4	2	1	3	2	1	3	4	1	5
2	3	2	3	2	1	3	2	1	2
3	1	2	1	2	1	1	2	1	1
3	1	2	1	1	2	1	1	1	1
1	1	2	1	1	1	1	1	1	1
1	1	1	1	1	1	1	1	1	1
1	1	1	1	1	1	1	1	1	1
3	3	3	1	2	2	1	1	1	5
1	2	3	2	3	3	3	3	3	4
3	1	1	1	2	1	1	1	1	1
1	2	2	1	1	1	1	2	1	1
1	2	1	4	1	1	1	1	1	1
1	2	1	1	2	1	2	2	1	2
1	1	1	1	1	1	1	1	1	1
3	2	2	2	1	2	1	1	1	1
1	2	1	2	2	1	1	1	1	2
1	1	1	1	1	1	1	3	1	1
1	1	1	1	3	1	1	4	1	1
1	1	1	1	4	1	1	3	2	1
1	1	1	1	4	1	1	1	1	1

2	1	1	3	3	1	4	1	1	1
1	1	2	1	2	1	1	1	1	1
2	1	1	3	1	1	1	1	1	1
3	1	1	1	1	1	1	2	1	1
3	1	1	1	1	1	1	2	1	1
3	1	4	4	1	1	1	1	1	4
2	1	2	2	2	1	1	2	1	1
1	1	1	2	1	1	1	1	1	1
3	1	1	4	1	1	1	1	1	1
3	1	1	1	1	1	1	1	1	1
3	1	1	2	2	1	2	1	1	1
3	1	2	1	1	1	1	1	1	1
3	1	1	1	3	1	1	1	1	1
3	1	1	1	1	1	1	1	1	1
3	1	1	1	1	1	1	1	1	1
2	1	4	4		1		2	1	1
3	1	1	1	1	1	1	1	1	1
2	2	1	2	1	1	1	1	1	1
3	4	1	4	2	1	3	3	1	4
2	1	1	1	1	2	1	1	1	1
2	1	1	1	1	1	1	1	1	1
2	2	1	3	2	3	2	3	1	1
2	1	1	1	1	1	1	1	1	1
2	1	1	1	1	3	1	1	1	1
2	2	1	2	1	1	1	1	1	1
1	1	2	1	1	1	1	1	1	1
1	1	1	4	2	1	1	1	1	1
2	1	1	1	1	1	1	1	1	1
2	1	1	1	2	1	1	1	1	1
2	3	1	1	1	1	1	1	1	1
2	1	1	1	1	1	1	1	1	1
2	1	1	1	1	1	2	1	1	1
2	1	1	1	1	3	1	1	1	1
3	1	1	1	1	1	1	1	1	2
1	1	1	1	2	1	1	2	1	3
2	1	1	2	2	1	1	3	1	1
2	1	1	2	1	1	1	1	1	1
3	1	1	1	1	1	1	1	1	1
2	1	1	3	1	1	1	1	1	1
2	1	1	1	2	1	1	1	1	1
3	2	1	2	2	1	1	1	1	1
2	1	3	1	1	1	1	2	1	1
4	1	1	1	2	1	2	2	1	1
2	2	2	1	1	1	1	1	1	1
3	1	1	2	1	2	1	2	1	1
2	1	1	1	1	1	1	2	1	1
2	1	1	1	1	1	1	1	1	1

2	3	2	1	2	2	1	3	1	1
2	1	1	1	1	1	1	1	1	1
1	2	1	1	3	1	1	1	1	1
3	1	1	1	1	1	1	1	1	1
3	1	1	1	1	1	1	1	1	1
2	1	1	1	1	1	1	1	1	2
2	1	1	1	1	1	1	1	1	2
2	1	1	1	2	1	1	1	1	1
3	1	1	2	1	1	1	1	1	1
2	4	1	2	2	1	1	1	1	1
1	1	1	1	2	1	1	1	1	1
3	1	1	2		1	1	2	1	1
2	2	2	1	1	1	1	2	1	1
3	2	1	1	1	1	1	1	1	4
2	4	1	3	2	1	1	2	1	4
2	1	1	2	2	1	1	1	1	1
2	1	4	4	1	1	1	4	1	2
2	1	1	4	2	2	1	1	1	1
1	2	2	1	2	2	1	1	1	1
1	3	4	1	1	1	1	1	4	2
1	1	1	2	1	1	1	1	1	1
3	1	1	1	1	1	1	1	1	1
1	1	2	2	1	1	1	1	1	1
1	2	2	1	1	1	1	1	1	1
2	1	1	3	1	1	2	2	1	1
1	2	2	1	2	1	2	2	4	1
1	1	1	1	1	1	1	1	1	1
3	1	2	1	1	1	1	2	1	1
1	1	1	1	1	1	2	2	1	1
3	1	1	1	1	1	1	2	1	1
1	1	1	1	1	1	1	1	2	1
1	2	2	2	2	1	1	2	1	1
1	1	1	1	1	1	1	1	1	1
1	1	1	1	1	1	1	1	1	1
2	1	1	2	1	1	1	1	1	1
1	4	4	2		2	1	1	2	2
2	1	1	1	1	1	1	1	1	1
4	2	2	2	2	1	1	1	1	1
1	3	3	2	2	2	2	3	2	1
1	1	2	1	1	1	1	1	1	1
1	2	2	2	1		1	1		1
4	4	3	3	2	1	3	2	4	1
4	1	1	1	1	1	1	1	1	1
2	1	1	1	1	1	1	1	1	1
2	3	1	3	1	1	2	1	1	
3	1	1	1	1	1	2	2	1	1
3	2	1	3	2	1	2	2	1	1

2	3	2	4	2	1	2	1	1	4
2	1	1	1	1	1	1	1	1	1
3	3	1	2	1	1	1	1	1	1
2	1	1	3	2	1	2	1	1	1
2	2	1	3	2	1	1	3	1	2
1	2	1	2	1	1	1	1	1	1
2	1	1	1	2	1	2	2	4	2
3	1	1	1	1	1	1	1	1	1
2	2	3	3	1	1	1	3	1	3
2	2	1	1	1	1	1	1	1	1
2	1	2	2	1	1	1	1	1	1
2	1	1	2	1	1	1	1	1	1
1	1	1	2	1	1	1	1	1	1
3	3	1	4	2	3	2	3	1	1
2	1	1	1	1	1	1	1	1	1
3	2	3	3	2	1	1	1	1	1
2	2	1	1	1	1	1	2		1
2	1	1	1	2	1	3	3	1	1
3	1	1	1	1	1	1	1	1	1
2	1	1	1	1	1	2	1	1	1
2	1	1	1	2	1	1	1	2	1
2	1	1	1	1	2	1	1	1	1
2	1	3	1	1	1	2	1	1	1
2	1	1	2	1	1	1	1	1	1
2	1	1	3	3	3	2	3	2	1
1	2	1	2	1	1	1	2	1	1
1	1	1	4	1	1	2	1	1	1
2	1	2	1	1	1	1	2	1	1
2	1	1	1	2	1	1	1	1	1
2	1	1	1	1	1	1	1	1	1
2	1	1	1	1	1	1	1	1	1
1	2	2	3	3	1	1	2	1	1
3	1	1	1	1	1	1	1	1	1
4	1	1	1	1	1	1	1	4	1
2	1	1	2	1	1	1	2	1	1
1	4	2	4	1	1	1	1	2	1
2	3	1	1	1	1	1	2	1	1
1	1	1	3	3	2	1	3	2	1
2	2	2	5	2	1	2	2	2	2
1	1	1	1	1	1	1	1	1	1
3	1	1	3	2	1	1	1	1	1
2	1	1	4	1	1	1	2	1	1
3	1	2	3	3	2	2	1	1	1
1	2	2	3	2	1	1	2	1	5
4	1	1	4	1	1	1	1	1	1
1	1	1	1	1	1	1	1	1	1
1	2	2	2	1	1	1	1	1	1

1	1	1	1	1	1	1	1	1	1
2	1	1	1	1	1	1	1	1	1
2	1	1	3	2	2	2	2	1	1
2	2	2	2	2	1	2	2	1	2
2	1	1	1	1	1	1	1	1	1
2	3	3							
2	2	2	2	2	2	2	2	2	2
4	1	1	1	2	2	2	1	2	1
4	3	2	2	2	1	1	1	2	2
1	2	2	2	2	2	2	2	1	1
1	2	2	1	3	2	1	1	1	3
1	2	3	2	2	1	2	3	1	2
1	2	2	1	2	1	2	2	1	1
4	1	1	1	2	3	2	2	2	2
2	2	1	2	1	2	1	2	1	2
2	1	2	2	1	1	1	1	1	1
1	1	2	2	2	2	2	2	2	2
1	1	2	1	2	1	2	1	2	2
1	1	1	1	2	1	2	1	1	1
2	1	1	1	1	1	1	1	1	1
1	1	1	1	1	1	1	1	1	1
1	4	1	1	1	1	2	1	1	1
4	1	1	1	1	1	1	1	1	1
3	1	1	1	1	1	1	1	1	1
1	1	1	1	1	1	1	1	1	1
2	1	1	2	2	2	2	2	2	2
3	1	1	2	2	1	1	1	1	1
4	1	1	2	2	1	1	1	1	1
4	1	1	3	3	3	3	3	1	3
2	1	1	1	1	1	1	1	1	1
2	1	1	1	1	1	1	1	1	1
4	2	1	1	2	1	2	2	2	2
4	1	1	1	1	1	1	2	2	2
4	1	1	1	1	1	2	2	2	2
3	1	1	1	1	1	1	1	1	1
2	2	3	2	2	1	1	2	2	2
2	1	1	1	1	1	1	1	1	1
4	1	1	1	2	3	2	2	2	1
2	2	2	2	2	2	2	2	2	2
2	1	1	2	1	1	1	1	1	1
2	1	1	4	4	1	3	1	1	1
1	1	1	1	2	1	1	1	1	1
3	1	1	1	1	1	1	1	1	1
2	2	3	3	3	1	3	3	1	5
2	3	1	1	1	1	1	1	1	1
2	3	1	1	1	1	1	1	1	1
1	2	2	4		2	2	1	1	1

1	2	2	4		2	2	1	1	1
2	1	2	3	2	1	1	1	1	1
2	1	2	3	2	1	1	1	1	1
2	2	2	1	2	1	1	1	1	2
2	2	2	1	2	1	1	1	1	2
2	1	2	3	3	1	1	2	1	1
4	1	2	2	1	1	3	2	1	1
2	1	2	1	1	1	1	2	1	3
2	1	1	3	3	1	4	3	1	1
1	2	1	1	1	1	1	1	1	1
2	1	2	1	3	1	1	2	1	1
2	1	2	1	1	1	1	1	1	1
2	1	1	4	3	1	2	3	1	1
2	1	2	3	3	2	2	3	1	1
1	2	2	1	2	1	1	1	1	1
2	3	1	4	2	1	1	1	1	1
2	3	3	3	3	2	1	1	1	2
2	1	1	2	1	1	1	1	1	1
2	1	1	3	1	1	1	1	1	2
2	2	2	1	1	2	1	1	1	2
3	4	2	2	1	2	1	1	1	1
1	2	2	1	3	3	1	1	1	1
2	1	2	1	1	1	1	1	1	1
1	2	2	1	1	1	1	1	1	1
3	1	2	2	1	1	1	1	1	1
1	2	1	2	2	2	2	2	1	1
1	1	2	1	1	1	1	1	1	1
1	2	1	3	1	1	1	1	1	1
1	1	1	3	1	1	1	1	1	1
2	1	1	1	1	1	1	2	1	1
2	1	2	1	1	1	1	1	1	1
1	1	1	3	3	1	1	1	1	2
1	2	2	1	1	1	1	1	1	1
1	1	1	1	1	1	1	1	1	1
4	1	1	2	2	1	1	1	1	1
4	1	1	1	1	1	1	1	1	1
4	1	1	1	2	1	1	1	1	1
1	2	1	2	2	2	1	1	1	1
2	2	2	2	1	1	1	1	1	1
2	2	1	3	2	1	1	1	1	1
2	1	1	2	1	1	1	1	1	1
2	4	1	2	2	1	1	1	1	1
1	1	1	1	1	1	1	1	1	1
2	2	3	2	2	1	2	1	1	1
1	3	3	2	3	1	1	2	1	1
1	4	2	4	3	1	3	1	1	1
1	2	1	1	1	1	1	1	1	1

2	2	1	1	1	1	1	1	1	1
3	1	3	1	1	1	1	1	1	1
1	3	1	1	2	2	2	1	1	1
1	2	3	1	1	1	1	1	1	1
3	1	1	4	2	1	1	2	1	1
3	1	1	2	2	1	1	1	1	1
2	1	1	1	2	1	1	1	1	1
1	1	1	3	1	1	2	3	1	1
3	1	1	2	1	1	2	2	1	1
2	1	2	3	1	2	1	1	1	4
4	2	1	1	1	1	1	1	1	1
2	1	2	2	2	1	1	2	1	1
2	1	1	2	2	1	2	2	1	1
1	1	1	2	1	1	1	1	1	2
2	1	1	1	1	1	1	1	1	1
2	2	1	1	1	1	1	1	1	1
3	1	2	2	1	1	1	2	1	3
3	1	2	2	1	1	1	2	1	3
2	1	3	2	1	1	1	1	1	1
1	1	1	1	3	1	1	1	1	1
2	4	1	1	3	1	1	1	1	1
2	1	1	3	1	1	1	1	1	1
3	1	2	2	2	1	2	4	1	1
2	1	1	1	1	1	1	1	1	1
2	2	2	1	2	1	1	4	1	4
1	3	1	1	1	2	1	1	2	1
2	2	1	1	3	2	3	3	1	3
1	1	2	2	1	1	1	1	1	3
2	1	4	2	1	1	1	1	1	1
2	1	1	1	1	1	1	1	1	1
2	1	1	1	2	1	1	1	1	1
3	1	1	1	1	2	1	1	1	1
2	2	2	2	1	1	1	1	1	1
3	1	1	1	1	1	1	2	1	1
3	1	1	2	2	1	2	1	1	1
1	1	1	2	1	1	1	1	1	1
1	1	1	2	1	1	1	1	1	1
2	2	2	2	2	1	1	1	1	1
2	1	1	2	1	1	1	2	1	1
2	1	2	2	2	1	2	2	1	3
2	2	2	2	2	2	2	2	2	5
1	3	2	2	1	1	1	2	1	2
3	1	1	1	2	1	1	1	1	1
3	1	1	1	2	1	1	1	1	1
1	2	1	4	1	1	3	2	1	4
2	1	1	1	1	1	1	1	1	1
1	2	1	3	1	1	1	1	1	1

1	2	1	1	1	1	1	1	1	1
2	2	1	1	1	1	1	3	1	1
2	3	2	3	1	1	1	1	1	1
2	1	1	3	2	1	2	2	3	1
2	2	2	2	2	1	1	3	1	3
3	1	1	1	1	1	1	1	1	1
3	2	3	2	2	1	1	1	1	1
3	1	1	1	1	1	1	1	1	1
2	2	1	3	2	1	1	1	1	1
2	2	1	2	1	1	1	2	1	2
4	1	1	2	1	1	1	1	1	1
2	1	1	1	1	1	1	1	1	1
3	1	1	2	4	1	1	1	1	1
2	2	2	2	2	1	2	2	1	1
2	1	1	1	2	1	2	2	1	2
3	1	2	1	1	1	1	1	1	1
1	1	2	4	2	1	2	3	1	1
2	1	1	2	2	1	1	1	1	1
2	1	1	2	1	1	1	2	1	1
3	3	1	3	2	1	1	1	1	1
1	1	1	2	2	1	2	1	1	1
1	1	1	1	1	1	1	1	1	1
3	2	1	1		1	1	1	1	1
2	1	1	2	2	1	1	2	1	1
3	1	1	1	1	2	1	1	1	1
2	2	3	3	2	1	1	1	1	3
3	1	2	4	2	1	2	2	1	1
3	2	2	3	1	3	1	1	1	1
3	1	1	2	2	1	1	2	1	1
3	1	1	3	1	1	1	2	1	1
2	1	1	1	2	1	1	1	1	1
3	1	1	3	2	1	1	1	1	1
2	2	1	1	1	1	1	1	1	1
2	2	2	1	1	1	1	2	1	1
3	1	1	2	1	1	1	1	1	1
3	1	1	1	1	1	1	1	1	1
2	2	1	1	3	2	1	1	3	1
1	2	2	3	2	1	2	1	1	4
2	2	1	3	4	1	3	1	1	4
2	1	1	1	2	1	1	1	1	1
2	2	2	3	2	1	1	1	1	3
2	1	1	1	1	1	1	1	1	1
3	1	2	2	1	1	1	1	1	1
3	1	2	2	1	1	1	1	1	1
3	3	1	1	1	1	1	1	1	1
1	2	3	2	2	1	1	2	2	1
2	2	1	4	3	1	1	1	2	1

2	1	3	2	1	1	1	1	1	1
2	1	1	1	1	1	1	1	1	1
1	2	1	1	1	1	1	2	1	2
3	2	2	4	3	3	2	3	3	3
1	4	1	1	1	1	1	1	1	1
1	2	2	3	3	1	1	1	1	1
1	1	1	1	1	1	1	1	1	1
1	1	1	3	1	1	1	1	2	1
1	1	1	3	1	1	1	1	2	1
1	2	2	1	2	1	1	2	1	2
1	2	1	1	2	1	1	1	1	1
3	1	2	2	1	1	1	1	1	1
1	1	1	1	1	1	1	1	1	1
1	2	1	3	2	1	1	1	1	2
2	1	1	1	1	1	1	1	1	1
1	1	1	3	1	1	1	1	1	1
1	3	1	2	1	1	1	1	1	1
1	1	1	1	1	1	1	1	1	1
1	4	1	1	1	1	1	1	1	1
1	1	1	1	1	1	2	2	1	1
1	1	2	2	1	1	1	1	2	1
1	1	1	1	1	1	2	1	2	1
1	1	1	1	1	1	1	1	1	1
1	1	1	1	1	1	1	1	1	1
1	2	1	3	1	1	1	1	1	1
3	1	1	1	1	1	1	1	1	1
2	2	1	2	2	1	1	3	1	1
1	3	1	1	2	1	1	2	1	1
1	3	3	3	3	2	1	2	1	4
1	3	1	1	1	1	1	1	1	1
2	1	1	2	3	2	2	2	1	1
1	2	2	2	3	2	1	1	1	1
1	2	1	1	2	1	2	1	1	2
1	1	3	3	1	1	3	3	1	1
2	1	1	2	2	2	2	1	2	1
1	1	1	1	1	1	1	1	1	3
1	1	1	2	2	1	2	1	1	1
1	1	1	3	4	1	1	4	1	2
1	1	2	1	1	1	1	1	1	1
2	1	1	1	1	1	1	1	1	1
2	1	1	1	1	1	1	1	1	1
2	1	1	1	1	1	1	1	1	1
1	2	2	1	1	1	2		1	1
3	4	1	1	1	1	1	1	1	5
2	2	1	2	2	1	1	1	1	1
1	2	1	2	1	1	1	1	1	2
2	1	1	2	2	1	1	1	1	1

2	2	1	3	2	1	3	3	1	1
1	1	1	2	2	1	1	1	1	1
2	1	2	3	3	1	3	1	1	1
1	3	1	1	2	1	1	1	1	1
3	3	1	3	1	1	2	2	1	1
1	3	1	2	3	2	1	2	1	3
1	3	1	1	1	1	1	1	1	1
1	2	2	2	2	1	2	2	1	1
3	1	1	1	1	1	1	1	1	1
2	2	2	2	2	1	2	2	1	1
2	1	1	1	1	1	1	1	1	1
1	1	1	1	1	1	1	1	1	1
3	1	1	1	2	1	1	1	1	1
2	1	1	2	1	1	1	1	1	1
3	2	1	1	1	3	2	2	3	1
3	1	1	1	1	1	1	1	1	1
3	1	1	4	1	1	1	1	1	1
2	2	2	1	2	1	1	1	1	1
2	4	1	1	2	1	1	2	1	1
2	1	1	1	3	1	1	1	1	1
2	1	1	2	1	1	1	2	1	1
3	1	1	3	2	1	2	1	1	1
3	1	1	1	2	1	1	2	1	1
2	1	3	1	1	1	1	1	1	4
2	1	1	3	2	1	1	2	2	1
2	1	1	1	2	1	2	1	1	1
2	1	1	1	1	1	1	1	1	1
2	2	1	1	2	1	1	2	1	1
1	1	1	1	1	1	1	1	1	1
3	1	2	2	2	1	1	2	1	1
1	1	1	2	2	2	3	1	1	1
3	1	1	4	2	1	1	2	1	1
3	1	1	4	2	1	1	2	1	1
1	1	2	1	1	1	1	1	1	1
1	1	1	1	1	1	1	1	1	1
1	1	1	1	1	1	1	1	1	1
1	1	3	1	2	1	1	1	1	1
1	1	1	1	1	1	1	1	1	1
1	2	2	2	2	1	1	2	4	1
1	3	1	1	1	1	1	1	1	1
1	1	2	2	2	1	1	1	1	1
3	1	1	1	1	1	1	1	1	1
2	2	1	2	2	1	1	2	1	1
4	1	1	1	1	2	1	1	4	1
2	2	1	2	2	1	1	1	1	1
3	1	1	2	1	1	1	2	1	2
2	2	1	2	1	1	1	1	3	1

2	1	1	1	1	1	1	1	1	1
2	3	1	4	2	1	1	3	1	2
2	1	1	1	1	1	1	1		1
2	1	1	1	1	1	1	1		1
2	1	1	3	2	1	1	1	1	1
4	1	1	1	1	1	3	2	4	1
3	1	1	3	1	1	3	1	1	1
3	1	1	3	1	1	3	1	1	1
4	1	1	1	1	1	1	1	1	1
2	1	1	4	3	1	3	1	4	2
2	1	1	1	1	1	1	1	1	1
2	3	1	4	2	1	3	3	1	1
2	2	2	2	2	3	2	2	3	1
3	3	1	3	2	1	2	1	1	1
2	3	1	3	2	1	1	1	3	2
3	1	1	1	1	1	1	1	1	1
2	2	1	1	1	1	1	1	1	1
1	3	1	4	3	1	3	1	1	3
1	3	1	4	3	1	3	1	1	3
1	1	1	1	2	1	2	1	1	2
4	1	1	1	1	1	1	1	1	1
4	1	1	1	1	1	1	1	1	1
2	2	2	2	4	2	2	2	1	2
3	1	1	1	1	1	1	1	1	1
2	1	1	1	1	1	1	1	1	1
1	2	2	2	2	2	2	2	2	2
4	1	1	2	1	1	1	2	1	1
2	1	1	1	1	1	1	1	1	1
1	2	2	2	2	2	2	2	2	2
2	2	2	2	2	1	1	1	1	2
1	4	1	3	2	1	1	1	1	1
2	1	1	2	2	3	3	3	3	1
3	1	1	1	1	1	1	1	1	1
4	1	1	1	1	1	1	1	1	1
4	1	1	1	1	1	1	2	2	2
1	2	2	1	2	1	2	1	1	1
3	2	2	2	2	1	2	2	1	3
2	1	1	1	1	1	1	2	1	1
2	1	1	1	1	4	2	1	1	1
1	2	2	2	2	2	2	2	2	2
4	4	3	2	2	2	2	3	2	3
4	3	3	3	3	3	3	3	3	3
1	1	2	2	1	1	2	1	4	3
4	1	1	1	1	1	2	2	2	2
1	1	1	1	2	1	1	2	1	4
2	1	2	1	2	2	1	2	1	1
2	1	1	2	2	2	1	2	1	1

3	1	1	3	3	1	1	2	1	1
4	2	2	2	2	1	1	1	1	1
2	2	1	4	3	2	2	1	1	1
2	1	1	4	1	1	1	1	1	2
2	1	2	3	3	1	2	2	3	4
2	1	1	1	1	1	1	2	1	1
3	1	1	1	2	1	1	1	1	1
3	1	1	1	2	1	1	1	1	1
2	1	1	1	3	1	1	3	1	1
2	2	4	4	4	1	3	3	1	1
2	3	2	1	1	1	1	1	1	1
2	2	3	4	3	2	1	2	1	1
2	2	3	3	3	2	1	3	1	1
2	1	1	1	1	1	1	2	1	1
3	1	1	1	1	1	1	1	1	1
1	1	1	1	1	1	1	1	1	1
1	1	1	1	1	1	1	1	1	1
1	1	1	1	1	1	1	1	1	1
1	1	1	2	2	1	1	2	1	1
2	1	2	2	1	1	1	1	1	1
1	2	4	5	1	1	1	1	1	5
1	1	1	1	1	1	1	1	1	1
1	1	2	2	1	1	1	2	1	1
2	1	1	2	2	2	2	2	1	1
2	1	1	1	1	1	1	1	1	1
1	3	3	1	3	1	2	3	1	1
1	1	1	1	1	1	1	1	1	1
1	2	1	1	1	1	1	1	1	1
1	1	1	2	1	1	1	1	1	1
2	4	1	4	2	1	1	2	1	2
2	1	1	2	1	1	1	1	1	1
3	2	2	1	1	1	1	1	1	1
2	3	3	3	1	1	1	3	1	1
2	3	1	1	1	1	1	2	1	1
1	3	3	2	2	1	1	2	1	1
1	1	1	1	1	1	1	1	1	1
1	1	1	1	1	1	1	1	1	1
4	1	1	1	1	1	1	1	1	1
1	1	1	2	1	1	1	1	1	1
1	1	1	2	1	1	1	1	1	1
2	1	1	1	1	1	1	1	1	1
1	2	1	3	1	1	1	1	1	1
2	1	2	2	2	1	1	2	1	1
2	2	1	2	1	1	1	1	1	1
2	1	1	1	2	2	1	1	1	1
1	1	1	3	1	1	1	2	1	2
4	1	1	3	1	1	1	1	1	1

2	2	1	1	1	1	1	2	2	2
3	1	1	1	1	1	1	1	1	1
2	1	1	2	1	1	1	2	1	1
2	2	1	2	2	1	1	1	1	2
1	1	2	2	2	1	1	2	1	1
2	2	2	2	2	2	1	1	1	1
3	2	1	1	1	1	1	1	1	2
	1	2	1	1	1	1	1	1	1
3	1	2	3	1	2	1	1	1	1
2	1	1	4	1	1	1	1	1	1
2	1	1	1	2	1	1	2	1	4
2	1	2	1	1	1	1	2	1	1
2	1	1	1	1	1	1	1	1	1
2	2	1	4	2	2	1	1	1	1
3	1	1	1	1	1	1	2	1	1
2	2	3	2	2	1	1	1	1	1
2	2	2	3	2	1	1	2	1	1
3	2	3	2	2	2	2	2	1	1
3	4	1	3	2	1	1	1	1	1
1	3	1	2	1	2	2	1	3	2
3	2	2	2	1	1	2	2	1	1
2	2	1	1	1	1	1	1	1	1
3	1	1	2	2	1	1	1	1	1
2	2	1	4	3	1	1	3	1	1
2	2	3	3	2	1	2	3	2	1
2	1	1	1	2	1	1	1	1	1
2	2	2	1	2	1	1	1	1	1
2	2	1	1	2	1	1	1	1	1
3	1	1	1	1	1	1	1	1	1
3	1	1	1	2	1	2	1	1	1
3	1	2	1	1	1	1	2	1	1
2	2	2	3	2	1	2	2	1	1
2	1	1	2	1	1	1	1	1	1
3	1	3	5	2	1	2	2	1	1
3	2	1	3	1	1	1	2	1	1
2	1	2	1	1	1	1	1	1	1
3	1	1	4	3	1	3	1	1	1
2	1	1	2	2	1	1	1	1	1
2	1	1	1	1	1	1	1	1	1
2	1	2	1	1	1	1	2	1	1
2	3	2	1	2	1	1	2	1	1
3	2	3	2	1	1	1	3	1	1
4	2	2	1	1	1	1	1	1	1
2	2	1	1	1	1	1	1	1	1
3	1	1	2	2	1	1	2	1	1
2	1	2	3	1	1	1	2	1	1
2	2	2	3	3	1	2	3	1	1

2	2	1	3	2	1	1	2	1	1
3	1	1	2	2	1	1	2	1	1
2	2	2	4	2	1	2	3	1	1
3	1	1	2	1	1	1	2	1	1
4	1	1	1	2	1	2	2	1	1
1	2	1	2	2	1	1	1	1	1
2	1	1	1	1	1	1	1	1	1
1	1	1	2	1	1	1	1	1	1
1	1	1	2	1	1	1	1	1	1
1	1	2	3	1	1	1	1	1	2
1	2	1	1	1	1	1	1	1	1
1	1	1	2	1	1	1	1	1	1
1	1	1	1	1	1	1	1	1	1
1	1	1	2	1	1	1	1	1	1
1	1	1	1	1	1	1	1	1	1
1	3	1	1	1	1	1	1	1	
1	1	1	3	1	1	1	2	1	1
1	1	1	1	1	1	1	1	1	1
2	1	1	1	1	1	1	2	1	1
3	2	3	3	1	1	1	1	1	2
1	1	1	1	1	1	1	1	2	1
1	1	2	2	2	1	2	1	1	1
2	1	1	3	1	1	1	1	1	1
1	3	2	1	2	1	2	2	1	4
2	1	1	1	3	1	2	2	1	1
1	1	1	3	1	1	2	1	1	1
3	1	3	1	1	1	1	4	1	1
1	3	1	4	3	1	2	3	1	4
2	3	1	3	1	1	1	2	1	1
1	1	1	3	2	1	1	1	2	1
1	1	1	3	2	1	1	2	1	1
1	1	1	2	1	1	1	1	1	1
1	3	1	1	1	3	1	1	1	1
1	1	1	2	1	2	2	3	3	2
1	2	1	3	1	1	1	2	1	1
3	1	2	2	2	1	1	3	1	1
3	1	1	4	1	1	1	3	1	1
3	1	1	4	1	1	1	1	1	1
2	2	2	2	2	1	1	1	1	1
1	1	1	2	2	2	3	3	1	1
2	1	1	1	1	1	1	1	1	1
1	2	2	3	3	1	1	1	1	1
2	2	1	1	1	1	1	1	1	1
3	1	1	4	1	1	1	1	1	5
2	4	1	1	1	1	1	1	1	3
1	2	1	4	1	1	1	2	1	1
3	3	1	1	2	1	1	1	1	1

2	4	2	1	2	1	1	1	1	1
2	1	1	1	1	1	1	2	1	3
3	2	1	1	1	1	1	2	1	1
2	1	2	1	1	1	1	1	1	1
2	1	1	2	2	1	1	1	1	1
3	1	1	2	2	1	2	2	1	1
2	2	2	2	2	2	1	1	1	2
3	1	2	1	2	2	2	2	1	1
1	3	3	1	1	1	1	1	1	1
2	1	1	1	1	1	1	1	1	1
2	1	2	1	1	1	1	1	1	1
2	2	1	1	1	1	1	1	1	1
1	1	1	2	2	1	2	2	4	1
4	1	1	3	2	1	1	1	1	1
3	3	1	1	2	1	1	1	1	1
1	1	1	3	3	1	3	3	1	1
2	1	1	3	2	1	1	1	1	1
2	4	1	4	2	4	1	1	1	5
4	1	1	1	1	1	1	1	1	1
3	1	1	1	1	1	1	1	1	1
4	1	2	2	2	2	1	2	3	1
1	2	2	4	2	2	1	1	1	1
1	2	1	1	2	1	3	2	1	1
4	1	3	1	1	1	1	1	1	1
1	1	1	1	2	1	1	2	1	1
1	1	1	1	1	1	1	1	1	1
2	1	1	2	1	1	1	1	1	1
1	1	1	2	1	1	1	1	1	1
1	1	1	3	1	1	1	4	1	1
1	1	1	2	1	1	1	1	1	1
2	1	1	1	1	1	1	1	1	1
1	1	1	1	1	1	1	1	1	1
1	1	1	2	1	2	3	1	1	1
2	2	1	1	1	1	1	1	1	1
1	1	1	4	1	4	1	4	4	2
2	2	3	3	3	1	2	3	1	1
2	1	1	1	1	1	1	1	1	1
2	1	1	1	1	1	1	2	1	1
2	1	1	1	1	1	1	2	1	1
2	2	2	2		1	1	1	1	1
3	2	4	4	3	2	2	1	4	3
2	1	1	1	1	1	1	1	1	1
1	1	1	3	2	1	1	1	1	1
1	2	3	2	2	4	1	1	3	1
1	1	1	1	4	1	4	4	1	1
1	3	1	3	3	1	3	1	1	2
3	1	3	1	2	1	1	2	1	1

2	1	1	2	2	1	1	3	1	1
3	1	1	4	2	2	2	1	1	2
3	1	1	4	2	2	2	1	1	2
3	1	1	1	1	1	1	1	1	1
2		1	2	1	2	1		2	1
1	1	1	1	3	1	1	3	1	1
3	1	1	1	2	1	3	3	1	1
1	1	2	3	3	1	1	3	1	1
2	2	3	1	1	3	1	1	2	1
1	2	2	2	1	1	1	1	1	1
2	1	1	1	1	1	1	1	1	1
2	2	3	1	1	3	1	1	2	1
2	3	2	2	1	1	1	1	1	1
1	1	1	2	2	1	2	2	1	1
2	2	2	3	2	1	2	2	1	2
1	1	1	1	1	1	1	1	1	1
4	1	1	2	2	1	1	3	3	3
2	2	1	2	2	1	2	1	1	2
2	2	2	1	2	1	1	2	1	1
2	2	2	1	2	1	1	2	1	1
2	1	1	2	2	1	1	2	1	2
2	2	2	2	2	2	2	2	2	1
1	2	3	2	3	1	2	1	1	1
2	1	1	1	1	1	1	1	1	1
3	1	1	1	1	1	1	1	1	1
1	2	2	2	2	1	1	2	1	1
2	2	2	1	2	2	2	2	2	2
3	1	2	3	3	1	2	1	1	1
2	2	2	1	1	1	1	1	1	1
2	2	1	4	3	1	2	2	1	1
2	1	1	1	1	1	1	1	1	1
2	1	2	4	3	1	3	3	1	1
1	2	1	1	1	1	1	1	1	1
2	1	2	3	3	3	1	3	1	5
2	4	1	2	2	1	1	3	1	2
1	1	2	2	2	1	1	1	1	1
2	3	1	2	1	1	1	1	1	1
1	2	1	2	2	1	1	2	1	1
1	1	1	4	1	1	1	1	1	1
1	1	2	1	1	1	1	1	1	1
1	1	1	1	1	1	1	1	1	1
2	1	1	2	2	1	1	2	1	1
3	1	3	2	1	1	1	1	1	1
1	2	1	1	1	1	1	1	1	1
1	1	1	2	1	1	2	2	1	1
1	1	1	2	1	1	1	1	1	1
1	4	4	1	1	1	1	1	1	1

2	1	1	1	1	1	1	1	1	1
3	1	1	1	1	1	2	1	1	1
2	3	3	4	1	1	1	1	1	1
4	3	3	1	2	2	2	3	1	1
1	3	2	4	3	1	2	2	1	3
2	1	1	3	1	1	1	3	1	1
1	1	1	2	1	1	1	2	1	1
4	1	1	2	2	1	2	3	1	1
1	2	1	3	1	1	1	1	1	1
2	1	1	1	1	1	1	1	1	1
2	1	1	1	1	1	1	1	1	1
2	2	2	1	1	1	1	1	1	1
2	2	2	1	1	1	1	1	1	1
2	4	3	3	3	1	1	2	3	1
2	1	1	2	2	3	1	1	1	2
2	2	1	2	2	1	1	1	1	1
1	1	1	1	1	2	2	1	1	1
1	1	1	2	2	1	1	1	1	1
2	1	1	2	3	1	1	2	1	1
2	1	1	4	2	1	2	2	1	1
1	1	2	2	1	1	1	1	1	1
2	1	1	1	1	1	1	1	1	1
3	2	2	2	1	2	1	2	4	2
3	1	1	1	1	1	1	1	1	1
3	1	1	2	2	1	1	2	1	1
1	1	1	2	3	1	1	1	1	1
2	1	1	1	1	1	1	1	1	1
1	2	2	1	1	1	1	1	1	1
2	3	2	2	2	2	1	2	1	2
3	1	2	4	2	1	1	3	1	3
3	2	1	2	1	1	2	1	1	1
2	1	2	3	2	1	1	1	1	1
2	2	1	2	1	1	1	1	1	1
3	1	2	1	2	1	1	1	1	1
2	1	1	1	1	1	1	1	1	1
3	2	1	2	2	2	1	2	3	1
4	1	1	1	2	2	2	1	1	1
1	1	1	2	1	1	1	1	1	1
2	1	1	2	2	2	2	3	4	1
3	1	1	2	1	1	1	2	1	1
2	1	1	1	1	1	1	2	1	1
2	2	2	2	1	1	1	1	1	1
2	2	2	1	2	1	1	1	1	2
3	1	1	1	1	1	1	3	1	1
3	1	1	1	2	1	1	2	1	1
2	2	2	3	2	1	1	2	1	1
3	1	2	2	1	1	1	1	1	1

2	1	1	2	2	1	1	1	1	1
2	2	2	2	2	2	1	2	1	2
2	2	1	1	1	1	1	2	1	1
2	2	1	1	2	3	1	1	1	1
3	2	1	2	2	3	1	2	1	1
1	2	4	4	2	2	2	2	1	1
1	2	2	2	2	2	2	1	1	2
1	2	2	3	3	3	2	3	2	3
1	2	4	4	2	2	2	2	1	1
1	4	1	1	1	1	1	1	2	1
1	1	1	2	1	1	1	1	1	1
1	1	1	1	1	1	1	1	1	1
1	2	2	3	3	3	2	3	2	3
3	1	1	1	1	1	1	1	1	1
1	2	1	3	2	1	1	1	1	1
1	1	1	1	1	1	1	1	1	1
1	2	2	2	2	2	2	1	1	2
3	1	1	1	1	1	1	1	1	1
1	1	1	1	1	1	1	1	1	1
3	2	1	1	3	2	1	2	1	1
1	2	1	2	1	1	1	1	1	1
1	2	1	1	1	1	1	1	1	1
1	2	1	1	1	1	1	3	1	1
1	2	3	3		1	1	4	1	2
1	3	3	4	3	3	1	3	3	3
1	1	1	1	2	2	1	3	1	1
1	2	1	1	1	1	1	1	2	1
2	2	1	1	2	1	2	2	1	1
2	3	1	1	2	2	1	4	1	1
2	1	1	1	2	1	1	2	1	1
1	1	1	3	3	3	1	1	1	2
1	1	1	4	3	3	3	3	3	1
2	1	1	3	2	1	1	1	1	1
1	1	1	1	1	1	1	3	1	2
1	1	1	1	2	1	1	1	1	1
1	1	1	4	3	2	2	1	1	1
1	3	3	3	1	1	1	3	1	1
1	1	1	1	1	1	1	1	1	1
1	2	1	1	1	1	1	1	1	1
2	1	1	1	1	1	1	1	1	1
2	1	1	2	1	1	1	1	1	1
3	2	1	3	1	3	1	3	1	4
2	1	1	1	2	1	1	1	1	5
2	2	2	4	3	1	1	3	1	1
1	2	1	1	2	1	1	1	1	1
3	2	4	2	2	1	1	1	1	5
1	2	1	1	1	1	1	1	1	1

3	1	1	1	1	1	1	1	1	1
2	2	2	2	2	1	1	1	1	
2	1	1	3	2	1	2	1	1	3
2	1	1	2	2	1	2	1	1	1
2	1	1	1	1	2	1	1	1	1
2	1	1	1	1	3	2	1	1	1
3	1	1	3	2	2	1	3	1	4
2	2	1	1	1	1	1	1	1	1
1	3	1	1	1	1	1	1	3	1
3	1	1	1	2	1	1	1	1	1
2	1	1	2	2	1	1	1	1	1
2	2	1	3	2	1	1	1	1	4
3	1	1	1	1	1	1	1	1	1
2	1	1	1	1	1	1	1	1	1
1	1	1	1	2	1	1	1	1	1
1	1	1	2	1	1	1	1	1	1
3	1	1	1	1	1	1	1	1	1
1	1	1	1	1	1	1	1	1	1
1	1	1	2	1	1	1	1	1	1
2	1	1	2	1	1	1	1	1	1
2	2	1	1	3	1	1	1	1	2
1	1	1	1	1	1	1	1	1	1
1	1	1	2	1	1	1	1	1	1
4	1	2	1	1	1	1	1	1	1
1	2	1	4	2	2	3	2	1	1
3	1	1	1	1	1	1	1	1	1
3	1	1	4	3	1	2	1	1	1
2	4	1	2	2	1	1	1	3	3
3	1	1	3	1	2	2	1	2	1
3	1	1	1	1	1	1	1	1	1
2	1	1	3	2	1	2	1	1	2
2	3	1	3	3	1	1	1	1	1
2	2	1	1	2	1	1	1	1	1
2	2	1	1	2	1	1	1	1	1
2	1	1	3	2	3	1	1	1	1
2	1	1	3	2	3	1	1	1	1
1	2	2	2	1	1	1	3	3	1
2	1	1	2	1	1	1	1	1	1
2	2	1	1	2	1	2	1	1	1
1	3	3	3	2	1	1	2	2	1
1	1	1	2	1	1	1	1	1	1
2	1	1	2	2	1	2	1	1	1
3	1	1	1	2	1	1	1	1	1
3	1	1	1	1	1	1	1	1	1
1	1	1	1	1	1	1	1	1	1
4	2	2	2	2	1	1	1	1	1
2	2	1	4	1	1	1	1	1	1

1	2	1	3	1	1	1	1	1	1
1	1	1	1	1	1	1	1	1	1
2	1	1	1	1	1	1	1	1	1
3	1	1	1	1	1	1	1	1	1
1	2	2	3	2	2	2	1	1	1
3	1	1	1	1	2	2	2	1	1
4	1	1	1	1	1	1	1	1	1
4	2	2	3	3	3	3	3	1	1
2	1	1	1	3	1	1	1	1	1
4	1	1	1	1	1	1	1	1	1
1	1	3	2	2	1	2	1	1	4
2	1	1	3	2	1	1	2	1	1
1	3	1	2	2	1	2	2	1	1
3	1	1	1	1	1	1	1	1	1
2	1	1	3	2	2	2	2	1	1
2	1	1	1	1	1	1	1	1	1
2	2	1	2	2	1	2	1	1	1
2	2	1	2	2	1	2	1	1	1
2	2	1	1	1	3	1	1	1	1
2	1	1	2	3	2	2	3	3	1
1	1	2	3	4	1	1	1	1	4
2	2	2	2	2	1	1	1	1	1
2	3	1	1	1	1	1	1	1	1
2	2	2	3	1	1	1	1	1	1
1	2	2	1	1	1	1	1	1	1
1	1	2	4	2	1	1	1	1	1
1	1	1	1	1	1	1	1	1	1
2	1	1	2	1	1	2	1	1	1
2	1	1	1	2	1	1	1	1	1
2	1	1	2	2	1	1	1	1	1
2	1	1	2	1	1	1	1	1	1
4	1	2	1	1	1	1	2	1	1
1	2	2	1	1	2	1	1	2	1
1	1	2	5	1	1	1	1	1	5
1	2	1	2	2	1	1	1	1	1
1	4	2	1	1	1	1	1	1	1
3	1	1	2	1	2	1	1	2	1
2	3	1	1	1	1	1	1	1	1
2	1	1	2	1	1	1	1	1	1
2	2	1	4	2	1	1	1	1	1
3	3	1	3	1	2	3	3	1	3
4	1	1	2	1	2	3	3	1	1
1	1	1	2	1	1	1	1	1	1
2	2	1	3	2	3	1	1	1	1
1	1	1	1	1	1	1	1	1	1
1	2	1	1	1	1	1	1	1	1
2	1	1	1	1	1	1	1	1	1

1	1	1	2	2	1	1	1	1	1
3	1	1	1	2	1	1	1	1	1
3	1	1	3	1	1	1	1	1	1
2		2	1	1	1	1	2	1	1
2	1	1	3	1	2	2	2	2	1
1	2	2	2	2	2	1	1	1	1
2	1	1	1	1	1	1	1	1	3
1	1	2	4	2	2	2	2	1	1
2	4	1	4	4	1	4	3	1	1
3	1	1	1	1	1	1	1	1	1
1	2	1	2	1	1	1	1	1	2
2	1	1	3	1	1	1	2	1	1
2	1	1	3	1	1	1	2	1	1
2	1	2	4	1	1	1	2	1	1
1	1	1	4	4	4	2	3	1	1
1	1	1	4	4	4	2	3	1	1
1	1	2	2	2	1	2	1	1	1
3	4	2	3	3	1	1	4	1	1
3	1	1	2	2	1	1	2	1	1
1	2		2	2	1	2	1		1
1	1	1	1	1	1	1	1	1	2
2	1	1	1	1	1	1	1	1	1
2	1	1	2	2	1	1	1	1	1
2	2	2	2	2	1	1	2	1	1
2	1	1	2	1	1	1	1	1	1
2	1	1	3	2	2	1	2	1	1
2	1	1	2	1	1	1	1	1	2
2	2	1	2	1	1	1	1	1	3
3	1	1	3	2	1	1	1	1	1
3	2	2	2	3	1	1	1	1	2
2	1	1	1	2	1	1	1	1	1
2	2	3	3	1	1	1	1	1	1
1	2	2	2	2	1	2	3	1	2
1	2	1	1	1	1	1	1	1	1
1	3	1	1	1	1	1	1	1	1
2	1	1	3	1	1	1	1	1	1
1	2	1	3	1	1	1	1	1	1
1	1	1	1	1	1	1	1	1	1
1	1	1	1	1	1	1	1	1	1
1	2	2	3	3	3	2	2	2	2
1	1	1	3	2	1	1	1	1	1
1	2	1	1	1	1	1	1	1	1
1	1	1	1	1	1	1	1	1	1
1	1	2	1	2	1	1	2	1	1
3	1	1	1	1	1	1	1	1	1
1	1	1	1	1	1	1	1	1	1
1	2	1	1	2	1	1	1	1	1

2	2	1	2	3	2	2	2	1	2
1	1	1	4	2	3	1	1	1	1
1	1	1	2	2	2	3	3	1	3
1	1	1	2	2	1	1	1	1	1
1	3	3	2	4	1	1	2	1	1
2	1	1	1	3	1	2	2	1	1
1	2	1	1	3	3	4	1	1	1
2	2	3	3	3	1	1	2	1	1
2	2	1	1	1	1	1	1	1	1
3	1	1	1	1	1	1	1	1	1
2	2	2	3	1	2	2	2	1	1
2	3	1	1	2	2	2	2	1	5
2	1	1	3	2	1	2	3	1	1
1	1	1	1	1	1	1	1	1	1
2	1	1	1	1	1	1	1	2	1
2	3	2	2	1	1	1	1	1	1
2	1	1	1	1	1	1	1	1	1
3	1	1	3	2	1	1	1	1	1
2	4	1	1	2	1	1	1	1	1
3	4	1	4	1	1	2	3	1	1
2	1	1	1	1	1	1	1	2	1
2	2	1	2	1	1	1	1	1	1
3	1	1	1	1	1	1	1	1	1
1	2	3	3	1	1	1	1	1	3
1	2	3	3	1	1	1	1	1	3
3	1	1	2	1	1	1	1	1	4
2	1	2	1	2	2	1	1	1	1
3	3	2	2	1	1	1	1	1	1
1	3	1	4	2	1	1	2	1	3
3	1	1	4	2	4	1	1	1	4
3	1	1	1	2	1	2	1	1	1
1	3	2	2	2	1	1	1	1	1
1	1	1	3	2	2	1	1	1	1
3	1	1	4	2	2	1	2	1	1
3	1	1	4	2	2	1	2	1	1
3	2	1	2	2	1	1	2	1	2
2	1	1	1	2	1	1	2	1	4
2	4	1	1	1	1	1	2	1	4
1	1	2	2	1	1	2	1	1	1
1	2	4	4	4	1	1	1	1	5
2	1	2	1	1	1	2	1	1	3
1	4	2	3		3	1	1	2	1
1	1	2	1	1	1	1	1	1	1
1	1	1	1	1	1	1	1	1	1
3	1	2	2	1	1	1	1	1	1
1	1	2	2	2	1	1	3	2	1
1	1	2	1	1	1	1	2	1	1

2	1	2	2	1	1	4	4	1	1
1	1	1	3	1	1	1	1	1	1
1	1	1	2	1	1	1	1	1	2
1	2	2	2	2	2	2	2	1	2
1	1	1	3	1	2	2	2	1	1
3	1	1	2	2	1	1	1	1	1
2	2	1	2	3	1	1	2	2	1
2	1	1	1	1	1	1	1	1	1
2	2	1	1	1	1	4	3	1	1
1	1	1	1	1	1	1	2	1	1
2	2	3	4	1	1	1	1	1	1
3	1	1	1	1	1	1	1	1	1
1	1	1	1	1	1	1	1	1	1
2	2	2	1	1	1	1	1	1	1
1	1	2		1	1	1	2	1	1
2	2	2	4	2	1	2	2	3	3
2	1	2	3	3	3	3	3	1	4
2	1	1	2	1	1	1	1	1	2
1	4	1	3	2	1	1	1	1	1
3	1	1	3	2	1	1	2	1	3
2	1	1	1	1	1	1	1	1	1
2	4	1	1	1	1	1	1	1	1
1	4	1	4	2	1	3	1	1	2
2	2	1	2	1	1	1	1	1	1
2	1	1	2	1	1	1	1	1	1
2	1	1	1	1	1	1	1	1	1
2	1	1	1	1	1	1	1	1	1
2	2	2	3	2	1	1	3	1	2
1	1	1	1	1	1	2	2	1	1
1	1	1	1	1	1	1	1	1	1
1	3	1	1	1	1	1	1	1	1
3	1	1	3	2	2	1	1	1	1
2	4	1	4	3	1	1	3	1	1
2	4	4	4	3	1	1	1	3	1
3	1	1	1	1	1	1	1	1	1
1	1	2	2	2	1	1	2	2	1
1	1	1	2	1	1	1	2	2	1
1	1	1	2	2	2	2	2	2	2
1	2	2	2	2	2	2	2	2	2
2	1	1	1	1	1	1	1	1	1
2	1	1	1	1	1	1	1	1	1
3	1	1	2	1	2	1	1	1	1
2	1	1	1	1	1	1	1	1	1
1	2	2	2	2	2	2	2	2	2
1	1	1	4	3	3	3			
3	1	1	1	1	1	1	1	1	1
1	1	1	1	1	1	1	1	1	1

1	2	2	3	3	1	1	1	1	1
1	2	1	1	1	1	1	1	1	1
3	1	1	1	1	1	1	1	1	1
1	1	3	2	2	2	2	1	1	5
1	1	1	1	1	1	1	1	1	1
1	1	1	1	1				1	1
2	1	2	2	1	1	1	1	1	1
1	1	1	1	1	1	1	1	1	2
3	3	1	1	2	2	2	3	3	3
2	2	1	3	3	1	3	3	1	3
2	1	1	1	1	1	1	1	1	2
1	3	2	1	2	1	2	2	1	1
2	2	2	3	2	1	2	1	1	4
4	1	1	1	1	1	1	1	1	1
2	2	2	2	2	2	2	2	2	2
2	1	1	3	2	2	1	2	1	1
1	3	1	5	1	1	1	2	1	1
2	1	1	2	1	1	1	2	1	1
2	2	2	2	1	1	1	1	1	1
2	1	1	3	3	2	2	2	1	1
2	1	2	1	3	1	1	1	1	1
2	1	1	2	3	1	2	2	1	1
1	2	2	3	3	1	1	2	2	2
1	2	2	3	3	1	1	2	2	2
1	2	2	2	2	2	2	2	2	2
1	4	3	2	2	2	1	1	1	1
1	1	1	3	1	1	1	1	1	2
2	1	1	3	3	2	1	2	1	1
1	1	1	3	3	2	1	2	1	1
2	1	1	1	1	1	2	1	1	1
1	1	1	4	3	3	1	1	3	1
2	2	3	2	2	2	2	2	1	1
1	2	2	4					1	1
1	1	1	4	2	2	1	1	1	1
3	1	1	3	2	1	1	2	1	1
1	1	1	2	1	1	1	1	1	1
1	1	1	2	1	1	1	1	1	1
3	1	1	3	1	1	1	1	1	1
3	1	1	3	1	1	1	1	1	1
2	1	1	2	3	1	2	3	1	1
1	1	1	2	2	1	1	1	1	1
1	1	1	4	1	1	1	1	1	1
2	1	1	4	3	3	3	2	3	1
2	1	1	1	1	1	1	2	1	1
2	1	1	1	1	1	1	2	1	1
1	2	1	3	3	1	2	2	1	2
1	2	1	3	3	1	2	2	1	2

2	3	1	1	1	1	1	1	1	2
1	2	2	3	3	1	1	2	1	1
1	2	1	3	1	1	2	1	1	1
1	1	1	2	2	1	1	2	1	1
1	1	1	1	2	1	1	2	1	1
1	1	1	1	1	2	1	2	1	1
1	2	1	3	1	1	1	2	1	4
1	2	2	2	1	1	1	1	1	1
2	1	2	1	1	1	1	1	1	1
1	2	2	2	2	1	1	1	1	1
1	3	1	4	1	1	1	1	1	1
1	2	4	5	2	2	2	1	1	1
1	2	1	2	2	2	2	2	1	1
1	2	1	1	2	1	1	1	1	2
2	1	1	1	1	1	1	1	1	1
1	2	1	2	1	1	1	1	1	1
1	1	1	1	1	1	1	1	1	1
1	1	4	1	3	1	2	1	1	5
1	1	1	1	1	1	1	1	1	1
2	1	1	2	2	1	1	1	1	1
1	2	1	3	1	1	1	1	1	1
2	1	1	1	1	1	1	2	1	1
1	1	1	3	1	1	1	1	1	1
2	2	2	2	1	1	1	1	1	1
2	1	1	1	1	1	1	1	1	1
1	1	4	3	1	1	1	3	2	1
1	1	1	1	1	1	1	1	1	1
1	1	2	3	1	1	1	2	1	1
2	2	1	3	1	1	1	2	1	1
1	1	1	1	1	1	1	1	1	1
1	1	2	1	1	1	1	1	1	1
2	1	1	4	1	1	1	1	1	1
1	1	2	5	1	1	1	2	2	1
1	1	2	3	1	2	2	2	1	1
2	1	1	1	1	1	1	1	1	1
1	2	2	1	1	1	1	1	1	1
2	1	1	1	1	1	1	1	1	1
2	1	1	4	1	1	1	1	1	1
2	1	1	1	1	1	1	1	1	1
2	1	1	1	1	1	1	1	1	1
1	1	2	1	1	1	1	1	1	1
1	1	1	2	1	1	1	1	1	4
2	1	1	3	3	1	1	1	1	1
2	1	1	1	1	1	1	1	1	1
1	3	2	2	1	2	1	1	2	2
2	1	1	4	1	4	1	2	1	1
2	1	2	3	1	1	1	2	2	1

2	1	1	1	1	1	1	1	1	1
3	1	1	2	2	2	1	1	1	1
2	1	1	1	1	1	1	1	1	4
1	1	1	4	3	1	1	1	1	4
2	3	1	3	3	1	1	1	1	1
2	3	1	3	3	1	1	1	1	1
2	1	1	1	1	1	1	1	1	1
1	1	1	3	3	1	1	2	3	1
2	1	1	3	2	1	1	1	1	1
2	1	2	4	1	1	4	2	1	2
1	1	1	2	2	1	1	1	1	1
2	1	1	2	1	1	1	2	1	1
1	3	2	2	1	1	1	2	1	3
3	1	1	1	1	1	1	1	1	1
2	1	1	1	1	1	1	1	1	1
1	1	1	1	2	1	1	2	1	1
2	2	2	1	1	1	1	1	1	1
2	1	1	1	4	1	3	1	1	1
2	1	1	1	4	1	3	1	1	1
2	1	2	2	1	1	1	1	1	1
2	2	1	1	1	1	1	1	1	1
2	1	1	1	1	1	1	1	1	1
1	1	2	3	2	3	2	2	1	1
2	1	1	1	1	1	1	1	1	1
2	1	1	4	1	2	1	1	1	4
2	2	1	1	1	1	1	1	1	1
2	1	1	1	1	1	1	1	1	1
2	4	2	4	1	1	1	2	1	4
2	1	1	1	1	1	1	2	1	1
3	2	3	4	1	1	1	1	1	1
2	1	1	3	1	2	1	3	1	1
2	2	3	4	4	1	1	2	1	1
3	1	1	1	2	2	1	2	1	1
2	3	1	1	2	1	1	1	1	1
2	2	2	1	2	1	1	2	1	2
2	4	2	5	4	1	3	3	1	5
2	1	1	2	1	1	1	1	1	1
2	1	1	2	2	1	2	2	1	1
2	4	3	1	2	1	1	1	1	1
2	2	2	2	3	2	1	2	1	1
2	1	2	1	1	1	2	1	1	1
3	2	1	1	1	1	1	1	1	1
3	1	1	1	2	1	2	1	1	2
3	1	2	1	1	1	1	1	1	1
2	2	2	2	2	1	1	1	1	1
2	2	1	1	1	1	1	1	1	1
2	1	2	1	1	1	1	2	1	1

1	2	1	1	2	1	1	1	1	1
2	1	2	2	1	1	1	1	1	1
2	3	1	2	1	1	1	1	1	1
3	1	1	1	2	1	1	1	1	1
3	2	1	2	2	1	1	2	1	3
3	1	1	2	2	2	1	1	1	3
2	2	1	1	1	1	1	1	1	1
1	1	2	1	1	1	1	1	1	1
1	2	1	3	2	2	2	2	1	1
1	2	2	2	2	3	2	1	1	1
1	1	1	2	1	1	1	1	1	1
1	4	1	1	1	1	1	1	1	1
1	1	2	2	2	1	1	1	1	1
1	1	1	1	1	1	1	1	1	1
1	3	1	1	1	1	1	1	1	1
1	2	2	2	1	1	1	1	1	1
1	1	1	2	1	1	1	2	2	1
1	1	1	1	1	1	1	1	1	1
1	1	1	3	2	3	2	3	1	1
1	1	1	1	1	1	1	1	1	1
1	1	1	2	1	1	1	1	1	1
1	1	1	1	1	1	1	2	1	1
2	1	1	3	1	1	1	1	1	1
1	1	1	1	1	1	1	1	1	1
1	4	1	1	1	1	1	1	1	1
1	4	1	1	1	1	1	1	1	1
1	1	1	2	1	1	1	1	1	1
1	1	1	2	1	1	1	1	1	1
2	1	1	1	1	1	1	1	1	1
1	1	1	1	1	1	1	1	1	5
1	2	1	1	1	1	1	1	1	1
1	1	1	2	1	1	1	1	1	1
1	1	1	1	1	1	1	1	1	1
1	1	1	1	1	1	1	1	1	1
1	1	1	1	1	1	1	1	1	1
1	1	1	3	2	1	1	1	1	1
1	1	2	2	2	1	2	3	1	1
1	1	2	3	2	2	1	1	1	2
1	4	1	1	1	1	1	1	1	1
1	1	1	1	1	1	1	1	1	1
1	1	1	1	1	1	1	1	1	1
1	1	2	2	1	1	2	1	2	1
1	1	1	2	1	1	1	1	1	1
1	1	1	1	3	1	1	2	1	1
1	1	1	3	3	2	2	3	1	1
1	1	3	3	3	1	3	3	3	1
1	1	2	2	1	1	1	1	1	1

3	2	3	3	4	3	3	4	1	1
1	1	1	2	3	2	1	3	1	1
1	3	1	3	3	3	3	3	1	3
1	1	1	2	1	1	1	1	1	1
1	4	1	1	4	1	1	2	1	3
3	2	2	1	1	2	1	1	1	1
1	1	1	1	3	2	1	1	1	1
1	1	1	1	1	1	1	1	1	1
1	1	2	1	1	1	1	1	1	1
1	1	1	1	1	1	1	1	1	1
1	1	1	1	4	1	1	1	1	1
1	1	1	1	1	3	1	1	3	5
1	1	1	1	1	1	1	1	1	1
2	2	3	1	2	2	1	2	2	4
1	1	1	4	4	4	4	4	4	4
1	2	1	1	2	1	2	2	1	1
1	1	1	4	2	3	1	3	1	1
1	3	1	3	3	1	2	2	1	2
1	2	3	4	4	1	3	4	1	1
1	1	1	1	2	1	1	1	1	1
1	2	1	1	1	1	1	3	1	1
2	2	2	2	1	2	1	1	1	4
1	4	1	1	1	1	1	1	2	2
1	1	1	2	1	2	1	1	1	1
1	1	1	1	3	3	1	4	3	3
1	3	3	1	2	1	1	1	1	4
1	3	1	1	1	1	1	1	1	1
2	1	1	1	3	1	1	1	1	4
2	1	1	1	1	1	1	1	1	1
3	2	1	1	2	1	1	3	1	1
2	1	1	1	1	1	1	3	1	1
3	1	1	1	1	1	1	1	1	1
2	3	1	2	1	1	1	1	1	1
2	1	1	1	2	1	1	1	1	1
2	1	1	4	2	1	1	1	1	1
2	1	1	1	1	1	1	1	1	1
2	2	1	2	2	1	1	1	1	1
2	1	1	3	3	1	1	1	1	1
3	2	1	1	1	1	1	1	1	1
1	1	1	4	1	1	1	1	1	1
2	2	1	1	1	1	1	1	1	1
1	1	1	1	1	1	1	1	1	1
2	1	2	4	3	2	2	2	1	1
2	1	1	1	1	1	1	1	1	1
2	1	1	1	1	1	1	1	1	1
2	1	1	1	1	1	1	2	1	1
2	2	3	3	1	1	1	1	1	1

3	4	1	1	3	1	1	1	1	1
2	3	3	3	3	1	1	1	1	1
1	2	2	2	1	1	1	1	1	2
2	2	1	1	1	1	1	1	1	1
1	1	1	4	1	1	1	1	3	1
1	1	1	1	1	1	1	1	1	1
1	1	1	1	1	1	1	1	1	3
2	1	1	1	2	3	1	1	1	1
2	1	1	3	2	1	1	1	1	1
3	1	1	4	1	1	1	1	1	1
2	2	3	2	1	1	1	1	1	1
2	1	1	1	2	1	1	1	1	1
2	1	1	1	3	1	1	1	1	1
2	1	1	1	1	1	1	1	1	1
3	1	1	2	1	1	2	1	1	1
1	2	1	2	2	1	1	1	1	1
1	1	1	1	1	1	1	3	1	1
2	2	1	1	1	1	1	1	1	1
2	1	1	1	1	1	1	1	1	1
2	4	3	4	3	1	2	2	1	5
2	3	3	3	3	3	3	3	1	3
1	3	1	2	2	1	1	1	1	1
2	1	2	1	3	1	1	1	1	1
2	3	1	1	4	1	1	1	1	1
2	1	1	1	2	1	1	1	1	1
3	1	1	1	1	1	1	1	1	1
2	4	1	1	1	1	1	1	1	1
1	3	1	2	2	1	1	1	1	1
2	3	1	1	1	1	1	1	1	1
2	3	1	2	2	1	1	1	1	1
2	1	2	2	1	1	1	1	1	1
2	4	2	1	1	1	1	2	1	4
2	2	1	1	2	1	1	1	1	1
3	2	1	1	1	1	1	1	1	1
2	2	1	3	1	1	1	1	1	1
2	1	1	1	1	1	1	1	1	1
2	1	2	1	1	1	2	1	1	1
1	1	1	1	2	3	2	2	2	2
1	1	1	1	1	1	1	1	1	2
1	3	1	1	1	1	1	1	1	1

beatmark	parknow	forcesex	teacher	bfforce	feelbad	feelbd	yellsmas	thrthurt	stopfrie
1	1	1	1	1	1	1	1	1	1
1	1	1	1	1	1	1	1	1	1
1	1	1	1	1	1	1	1	1	1
1	1	1	2	1	1	1	1	1	1
1	1	1	1	1	1	1	1	1	1
1	1	1	1	1	1	1	1	1	1
1	1	1	1	1	1	1	1	2	2
1	1	1	1	1	1	1	1	1	1
1	1	1	1	1	1	1	1	1	1
1	1	1	2	1	1	1	1	1	1
3	1	1	1	1	2	1	1	1	1
1	3	1	2	1	4	4	1	1	3
1	1	1	1	1	1	1	1	1	1
1	1	1	1	1	1	1	1	1	1
1	1	1	1	1	1	1	1	1	1
1	1	1	1		1	1	1	1	1
1	1	2	2	1	1	1	1	1	1
1	2	1	2	1	3	1	1	1	1
4	1	1	4	1	3	1	4	4	4
1	1	1	1	2	1	1	1	1	1
1	1	1	1	3	1	1	1	1	1
1	2	1	1	1	1	1	1	1	1
1	1	1	1	1	1	1	1	1	3
1	1	1	1	1	1	1	1	1	1
1	1	1	1	1	3	1	3	3	1
1	1	1	1	1	1	1	1	1	1
3	1	1	1	1	1	1	1	1	1
2	1	1	1	4	4	1	1	1	1
1	1	1	2	1	2	1	1	1	1
1	2	1	1	1	1	1	1	1	1
1	1	1	1	1	1	1	1	1	1
1	1	1	2	1	1	1	1	1	1
1	1	1	4	1	3	1	3	1	1
1	1	1	2	1	1	1	1	1	3
1	1	1	2	1	1	1	1	1	1
2	1	1	4	1	1	1	1	1	1
1	1	1	3	1	2	2	2	2	3
2	2	1	1	1	1	1	1	1	1
1	1	1	4	1	1	1	1	1	1
3	2	1	2	1	1	1	1	1	3
1	1	1	1	1	1	1	1	1	1
1	1	1	3	1	1	1	1	1	2
1	1	1	2	1	1	1	1	1	1
1	1	1	2	1	2	2	1	1	1
1	1	1	1						
1	1	1	2						

1	2	1	4	1	3	1	1	1	2
1	1	1	2	1	1	1	1	1	1
1	1	1	1	1	1	1	3	1	1
1	1	1	3	1	1	1	1	1	1
1	1	1	1	1	1	1	1	1	1
1	1	1	4	1	1	1	1	1	1
2	1	1	1	1	1	1	1	1	1
2	1	1	1	1	1	1	1	1	1
1	1	1	1	1	1	1	2	1	1
2	3	1	3	1	4	1	1	1	1
1	1	1	1	1	3	1	1	1	1
1	1	1	1	1	1	1	1	1	1
1	1	1	1	1	1	1	1	1	1
1	1	1	2	1	1	1	1	1	3
1	1	1	2	1	1	1	1	1	1
2	2	1	1	1	1	2	3	1	4
1	1	1	1	1	1	1	1	1	1
1	1	1	1	1	1	1	1	1	1
1	1	1	2	1	1	1	1	1	1
2	2	1	2	1	2	1	3	1	1
1	1	1	1	1	1	1	1	1	1
1	1	1	1	1	1	1	1	1	1
1	1	1	1	1	1	1	1	1	1
1	1	1	2	1	1	1	1	1	1
1	1	1	2	1	1	1	1	1	1
1	1	1	1	1	1	1	1	1	1
1	1	1	1	1	1	1	1	1	1
1	5	2	1	1	1	1	1	3	3
1	2	1	2	1	1	1	1	1	1
1	1	1	1	1	1	1	1	1	1
2	1	1	2	1	1	1	1	1	1
1	1	1	4	1	1	1	1	1	1
2	2	2	2	2	1	1	1	1	1
2	3	2	3	4					
1	2	1	2	1	1	1	1	1	1
1	1	1	1	1	1	1	1	1	1
1	1	1	1	1	1	1	1	1	1
1	1	1	2	2	1	1	1	1	1
1	1	1	1	1	4	4	4	4	4
1	1	1	1	1	3	1	1	1	1
1	1	1	1	1	1	1	1	1	1
1	1	1	1	1	1	1	1	1	1
2	1	1	2	1	1	1	1	1	2
1	2	1	1	1	3	3	2	3	3
1	1	1	1	1	1	1	1	1	1
1	1	1	1	1	1	1	1	1	1
2	1	1	2	1	1	1	1	1	1

3	5	4	4	4	2	2	2	3	2
1	1	1	1	1	2	2	1	1	1
1	1	1	1	1					
1	1	1	3	1	4	1	4	4	1
1	1	1	2	1	4	1	4	4	3
1	2	1	2	1	3	1	3	1	1
1	2	1	2	2	3	1	3	1	1
1	1	1	2	1	3	1	2	1	1
1	2	1	3	2	4	1	4	1	4
2	1	1	3	1	1	1	1	1	1
3	1	1	3	1	3	1	1	1	1
1	1	1	1	1	3	1	1	1	1
2	2	1	2	1	1	1	1	1	4
2	2	1	2	1	1	1	1	1	1
1	1	1	1	1	4	1	1	1	1
1	1	1	1	1				1	1
1	1	1	3	1	4	1	3	1	1
1	1	1	3	1	4	3	4	1	4
1	1	1	3	1	3	1	3	3	1
1	1	1	1	1	4	4	4	4	4
1	1	1	1	1	3	1	1	1	1
1	1	1	3	3	1	1	4	1	1
1	1	1	3	1	3	1	3	3	3
1	1	1	1	1		1	1	1	1
1	1	1	2	1	3	1	1	1	1
1	1	1	1	2	1	1	1	1	1
2	1	1	2	1	1	1	1	1	1
1	1	1	1	1	1	4	4	1	4
1	1	1	1	1	1	4	4	1	4
1	1	1	1	1	1	1	1	1	1
1	1	1	2	1	1	1	1	1	3
1	1	1	1	1	1	1	1	1	1
2	1	1	2	2	1	1	1	1	1
1	1	1	2	1	1	1	1	1	1
					1	1	1	1	1
1	1	1	3	1	3	3	1	1	3
1	1	1	1	1	1	1	1	1	1
1	1	1	1	1	1	1	1	1	1
1	1	1	1	1	1	1	1	1	1
1	4	3	2	1	1	2	3	1	2
2	1	1	1	1	1	1	1	1	1
1	2	1	1	1	1	1	3	1	1
1	1	1	1	1	1	1	1	1	1
1	1	1	2	1	1	1	1	1	1
1	1	1	1	1	1	1	1	1	1
3	4	1	1	1	2	3	2	1	1
4	1	1	1	1	4	4	3	1	1

1	1	1	1	3	1	1	3	1	1
1	1	1	1	1	1	1	1	1	1
1	1	1	3	1	2	1	1	1	1
1	1	1	1	1	1	1	1	1	1
1	1	2	3	1	1	1	1	1	1
1	1	1	2	1	1	1	1	1	1
1	1	1	1	1	3	3	1	1	1
1	1	1	4	1	1	1	1	1	1
1	1	1	1	1	1	1	3	1	1
1	1	1	1	1	1	1	3	1	1
1	1	1	3	2	2	1	1	1	1
1	1	1	2	1	1	1	1	1	1
1	1	1	4	1	1	1	1	1	1
1	1	1	1	1	1	1	1	1	1
1	1	1	1	1	2	4	3	4	4
1	1	1	1	1	1	1	1	1	1
1	1	1	1	1	1	1	1	1	1
1	1	1	2	1	1	1	1	1	1
1	1	1	1	1	3	1	1	1	1
1	1	1	1	1	3	1	1	1	1
1	1	1	3	2	2	1	1	1	1
1	1	1	3	1	3	1	1	1	1
1	1	1	3	1	2	1	1	1	1
1	1	1	4	3	1	1	1	1	1
1	1	1	1	1	3	1	1	1	3
1	1	1	1	1	1	1	1	1	1
1	1	1	1	1	1	1	1	1	3
1	1	1	3	1	1	1	2	1	2
1	1	1	3	1	1	1	1	1	1
2	1	1	3	1	1	1	1	1	1
1	1	1	2	1	1	1	1	1	1
1	1	1	3	1	1	1	1	1	1
1	1	1	3	1	1	1	1	1	1
1	1	1	1	1	1	1	1	1	1
1	1	1	1	1					
1	1	1	3	2	3	1	1	1	1
1	1	1	3	1	1	1	1	1	1
3	1	1	3	1	3	1	1	1	2
1	1	1	4	1	1	1	1	1	1
1	1	1	1	1	4	4	4	4	1
1	1	1	1	1					
1	1	1	3	1	4	1	1	2	4
1	1	1	2	1	3	1	1	1	1
1	1	1	3	1	1	1	1	1	1
1	1	1	2	1	1	1	1	1	1
1	2	1	2	1	3	1	1	1	2
1	1	1	1	1	1	1	1	1	1

1	1	1	2	1	1	1	1	1	1
1	1	1	3	1	1	1	1	3	1
1	1	1	4	1	1	1	1		
1	1	1	3	1	2	2	3	1	3
1	1	1	2	1	1	1	1	2	3
1	1	1	1	1	3	1	1	1	2
1	1	1	4	1	1	1	2	2	1
1	1	1	2	1	1	1	1	1	1
1	1	1	3	2	1	1	1	1	1
1	1	1	1	1	3	1	1	1	1
1	1	1	2	1	1	1	1	1	1
1	1	1	2	1	1	1	1	1	1
1	1	1	2	2	1	2	1	1	1
1	1	1	3	1					
1	1	1	1	1	1	1	1	1	1
1	1	1	1	1	4	1	4	4	1
1	1	1	1	1	1	1	1	1	1
1	1	1	1	1	1	1	1	1	1
1	1	1	1	1	1	1	1	1	1
1	1	1	1	1	1	1	1	1	1
1	1	1	1	2	1	1	1	1	1
1	1	1	1	1	2	1	1	1	1
2	1	1	2	1	1	1	1	1	1
1	1	1	1	1	1	1	1	1	1
1	1	1	1	1	4	1	4	4	1
1	1	2	1	1	1	1	1	1	1
1	1	1	1	1	1	1	1	1	1
1	1	1	1	1	1	1	1	1	1
1	1	1	1	1	1	1	1	1	1
1	1	1	1	1	1	1	1	1	1
2	1	1	2	1	4	4	4	4	1
1	1	1	2	1					
1	1	1	1	1	1	1	1	1	1
1	1	1	1	1					
1	2	1	3	1					
3	1	1	4	1	3	1	1	1	4
1	1	1	2	1					
2	2	2	1	1	2	1	1	1	1
1	1	1	3	1	3	1	1	1	3
1	1	1	1	1	1	1	1	1	1
1	1	1	1	1	2	4	1	1	1
3	3	3	1	3	1	1	2	1	2
3	1	1	3	1	1	1	3	1	3
1	2	1	2	2	3	3	3	4	1
1	1	1	1	1	1	1	1	1	1
1	1	1	1	1	1	1	1	1	1
1	2	1	2	1	1	1	1	1	1

2	1	1	3	1					
2	3	1	3	1	3	2	4	2	4
1	1	1	2	1	1	1	1	1	1
1	1	1	1	1	1	1	1	1	1
1	1	1	1	1	1	1	1	1	1
1	1	1	3	1	1	1	1	1	1
2	1	1	1	1	1	1	1	1	1
1	1	1	2	1	1	1	1	1	1
2	1	1	2	1	2	1	1	1	1
1	1	1	1	1	4	1	1	1	1
1	2	1	1	1	3	1	1	1	1
1	2	1	2	1	1	1	1	1	1
1	1	1	1	1	1	1	1	1	1
1	1	1	3	1	1	1	1	1	1
1	1	1	1	1	1	1	1	1	1
1	1	1	1	1	1	1	1	1	1
1	1	1	1	1	1	1	3	1	1
1	1	1	1	1	1	1	1	1	1
1	1	1	1	1	1	1	1	1	1
1	1	1	1	1	1	1	1	1	1
1	1	1	1	1	1	1	1	1	1
2	1	1	2	1	3	1	3	1	3
3	4	1	1	1	3	1	4	1	4
4	4	1	1	1	4	1	4	1	4
1	3	1	2	1	3	1	3	1	1
1	1	1	3	1	1	1	1	1	1
1	1	3	3	4	1	1	1	1	1
2	2	1	1	1	1	1	1	1	2
1	2	1	2	1	1	1	2	1	1
2	2	1	2	1	2	1	1	1	2
1	1	1	2	1	1	1	1	1	1
1	2	1	1	1	1	1	1	1	1
1	2	1	1	1	1	1	1	1	1
1	1	1	1	1	1	1	1	2	3
1		2	1	1	4	2	3	1	1
1	1	1	1	1	1	1	1	1	1
1	1	1	2	1	3	3	1	1	1
1	1	1	1	1	2	2	2	2	2
2	1	2	1	1	4	3	4	4	4
1	1	1	3	1	1	2	2	2	1
1	1	1	1	1	1	1	1	1	1
1	1	1	1	1	2	1	1	2	2
1	1	1	1	1	1	1	1	1	1
1	1	1	2	1	3	1	1	1	1
1	1	1	1	1	1	2	2	2	1
1	1	1	3	1	1	1	1	1	1
1	1	1	1	1	2	2	2	1	1

2	1	3	2	2	1	1	1	1	1
1	1	1	1	1	1	1	1	1	1
1	1	1	1	1	1	1	1	1	1
1	1	1	2	1	1	1	1	1	1
1	1	1	1	1					
1	1	1	1	1	4	1	1	1	3
1	2	1	3	1	1	1	1	1	1
1	1	1	1	1	1	1	1	1	1
3	1	1	1	1	1	1	1	1	1
3	3	1	3	1	2	3	3	3	3
1	1	1	1	1	1	1	1	1	1
1	2	1	1	1	2	1	1	1	3
1	1	1	1	1	1	1	1	1	1
1	1	1	1	1	1	1	1	1	1
1	1	1	1	1	1	1	1	1	1
1	1	1	4	1	2	1	2	1	1
2	2	1	2	1	2	1	4	1	1
1	1	1	1	1	1	1	1	1	1
1	1	1	2	1	1	1	1	1	1
3	1	1	3	1	1	1	1	1	1
1	1	1	3	1	1	1	1	1	1
1	1	1	2	1	3	3	4	1	1
1	1	1	3	2	4	3	3	3	4
1	1	1	1	1	3	1	1	1	1
1	1	1	2	1	2	1	1	1	1
1	2	1	2	1	4	4	4	4	4
1	1	1	2	1	1	1	1	1	1
2	1	1	1	1	1	1	1	1	1
1	1	1	3	1	1	1	1	1	1
1	4	2	2	1	3	1	3	3	2
1	2	1	1	1	3	3	1	2	1
2	1	1	1	1	1	1	1	1	1
1	1	1	2	1	1	1	1	1	1
1	2	1	3	1	1	1	1	1	1
1	4	1	1	1	1	1	1	1	1
1	1	1	2	1	4	4	1	1	1
1	1	1	1	1	1	1	1	1	1
1	1	1	2	1	3	1	1	1	1
1	1	1	4	1	1	1	1	1	1
1	1		1	1	1	1	1	1	1
1	2	1	3	1	1	1	1	1	1
1	1	1	2	1	3	1	1	1	3
1	1	1	1	1	1	1	1	2	2
1	1	1	2	1	3	3	3	2	1
1	1	1	2	1	1	1	1	1	1
3	2	1	1	1	1	1	1	1	1
1	2	1	2	3	3	3	3	3	3

1	2	1	2	3	3	3	3	3	3
1	2	1	2	1	1	1	1	1	1
1	1	2	1	3	2	2	2	2	1
1	1	1	1	1	3	3	3	1	3
1	2	1	2	1	1	1	1	1	1
1	2	1	2	1	1	1	1	1	1
1	1	1	1	1	1	1	1	1	1
1	1	1	1	1	1	1	1	1	1
1	1	1	2	1	1	1	1	1	1
1	1	1	4	1	1	1	1	1	1
1	1	1	2	1	1	1	3	1	3
1	1	1	4	1	1	1	1	1	1
1	3	1	3	1	4	4	4	4	2
1	1	1	2	1	3	3	1	1	2
1	1	1	2	1	1	1	1	1	1
1	1	1	1	1	3	3	1	1	2
1	1	1	3	1	1	1	1	1	1
1	3	1	1	1	1	1	1	1	3
1	2	1	3	1	4	4	4	4	4
1	1	1	2	1	3	3	3	4	2
1	1	1	1	1	1	1	1	1	1
1	1	1	1	1	2	1	1	1	3
1	1	1	1	1	1	1	1	1	1
1	1	1	3	1	1	1	1	1	1
1	1	1	3	1	1	1	1	1	3
1	1	1	2	1	1	1	1	1	1
1	1	1	2	1					
1	1	1	1	1	1	1	1	1	1
1	1	1	2	1	3	3	3	1	2
2	1	1	2	2	3	1	1	1	1
1	1	1	2	1	3	1	3	1	3
1	1	1	1	1	1	1	1	1	1
1	1	1	1	1	1	1	1	1	1
1	1	1	2	1	1	3	1	1	1
1	1	1	2	1	2	2	3	3	1
1	1	1	1	2	3	1	1	1	3
1	1	1	2	1	3	1	3	1	3
1	1	1	1	1	1	1	1	1	1
1	1	1	1	1	1	1	1	1	1
1	1	1	1	1	1	1	1	1	1
2	1	1	1	1	2	1	1	1	1
1	1	1	1	1	1	1	1	1	1
2	2	2	2	2	1	1	1	1	2
2	2	1	1	1	1	1	1	1	1
1	1	1	1	1	4	1	1	1	1
1	1	1	1	1	4	1	1	1	1
1	1	1	1	1	1	1	1	1	1

1	1	1	1	1	1	1	1	1	1
1	1	1	1	1	1	2	1	1	1
1	1	1	1	1	1	1	1	1	1
1	1	1	1	1	1	1	1	1	1
1	1	1	1	1	4	4	4	4	4
1	1	1	1	1	2	2	2	2	2
1	1	1	1	1	1	1	1	1	1
1	1	1	1	1	1	1	1	1	1
1	1	1	2	1	1	2	1	1	1
3	2	1	3	1	3	3	3	2	2
3	1	1	3	1	2	2	1	1	1
1	1	1	2	1	3	1	2	1	1
1	1	1	3	1	4	4	4	4	4
1	1	1	3	2	4	1	3	1	1
1	1	1	3	1	4	1	3	3	1
1	1	1	2	1	4	4	1	1	1
1	1	1	1	1	3	1	1	1	1
1	1	1	1	1	3	1	1	1	1
1	1	1	3	2	4	4	4	4	3
1	1	1	1	1	1	1	1	1	1
1	1	1	1	1	4	4	1	4	1
2	1	1	1	4	4	4	4	4	4
1	1	1	1	1	4	4	4	4	4
1	1	1	1	1	4	1	4	4	1
1	2	1	1	1	3	1	1	1	3
1	1	1	1	1	4	1	3	3	1
1	1	1	4	3	4	4	4	4	1
2	2	1	1	1	4	3	1	1	1
1	1	1	2	1	1	1	1	1	1
1	1	1	3	1	3	1	1	1	1
2	1	2	2	2	1	1	1	1	1
1	1	1	1	1	1	1	1	1	1
1	1	1	2	1	1	1	1	1	1
1	1	1	1	1	1	1	1	1	1
1	1	1	1	1	1	1	1	1	1
1	1	1	2	1	3	1	1	1	1
1	1	1	1	1	1	1	1	1	1
1	1	1	1	1	1	1	1	1	1
1	1	1	2	1	1	1	1	1	1
1	1	1	2	3	3	2	1	2	2
1	1	1	1	2	1	1	1	1	1
1	1	1	1	1	4	1	4	1	1
3	2	1	3	1	2	2	3	3	2
1	1	1	2	1	1	1	1	1	1
1	1	1	2	1	1	1	1	1	1
2	1	1	1	1	1	1	1	2	1
1	1	1	2	1	1	1	1	1	1

1	1	1	1	1	1	1	1	1	1
1	1	1	2	1	1	1	3	1	1
1	1	1	1	1	3	1	4	1	1
1	1	1	2	1	3	1	3	1	1
1	1	1	1	1	1	1	1	1	1
1	1	1	2	1	3	3	1	1	1
3	3	1	1	1	1	1	1	1	1
1	1	1	1	1	1	1	1	1	1
2	2	1	3	1	2	1	1	1	1
1	1	1	1	1	3	1	1	1	1
1	1	1	1	1	1	1	1	1	1
1	1	1	1	1	4	1	4	1	1
1	2	1	1	1	1	1	1	2	1
1	1	1	1	1	1	1	1	1	1
1	1	1	1	1	1	1	1	1	1
1	1	1	1	1	1	1	1	1	1
2	1	1	2	1	3	1	4	2	4
1	1	1	1	1	4	4	1	1	4
1	1	1	1	1	2	1	1	1	1
1	1	1	4	1	1	1	1	1	1
1	1	1	2	1	3	1	3	1	1
3	1	1	1	1	1	1	1	1	1
2	1	1	3	1	1	1	1	1	2
1	1	1	4	1	1	1	1	1	1
1	1	1	2	1	1	1	1	1	1
1	1	1	2	1	1	1	1	1	1
2	1	1	4	1	2	3	1	1	1
1	1	1	1	1	4	4	4	4	4
2	1	1	1	1	3	1	3	1	1
1	1	1	2	1	1	1	1	1	1
2	1	1	2	1	3	2	1	2	1
1	1	1	2	1	1	1	1	1	1
1	1	1	2	1	1	1	1	1	1
1	1	1	3	1	1	1	1	1	1
1	1	1	2	1	1	1	1	1	1
2	1	1	1	1	1	1	4	3	4
1	1	1	1	1	4	1	1	1	1
1	1	1	1	1	4	4	4	4	4
1	1	1	4	1	3	1	3	3	1
1	1	1	1	1	1	1	4	1	4
1	1	1	1	1	3	1	3	1	1
3	3	2	1	1	1	1	1	1	1
1	1	1	1	1	3	1	4	1	3
1	1	1	2	1	2	2	3	1	2
1	1	1	2	1	4	4	4	3	3
1	1	1	2	1	3	1	1	1	3
1	1	1	2	1	3	1	1	1	1

1	1	1	1	1	3	3	1	1	2
1	1	1	3	1	1	1	1	1	1
1	1	1	1	1	1	1	1	1	1
1	2	1	2	1	3	1	3	1	3
1	1	1	1	1	2	1	1	1	2
2	1	1	2	1	3	1	1	1	1
1	1	1	3	1	1	1	1	1	1
1	1	1	4	1	3	1	3	1	1
1	1	1	3	1	1	1	1	1	3
1	1	1	4	1	1	2	1	1	1
1	1	1	3	1	3	1	1	1	1
2	1	1	2	1	4	3	1	1	3
1	1	1	2	1	1	1	1	1	1
1	1	1	1	1	1	1	1	1	1
1	1	1	3	1	1	1	1	1	1
1	1	1	2	1	1	1	1	1	1
1	1	1	2	1	3	1	2	3	3
1	1	1	2	2	1	1	1	1	1
1	1	1	2	1	3	1	1	1	1
1	1	2	3	1	4	1	4	1	3
1	1	1	4	1	1	1	2	1	2
1	1	1	1	1	1	1	1	1	1
1	1	1	3	1	1	1	1	1	1
1	1	1	2	1	1	1	1	1	1
1	1	1	1	1	3	1	1	1	1
1	1	1	1	1	3	1	3	1	1
1	1	1	1	1	1	1	1	1	1
1	1	1	4	1	2	1	1	1	1
1	1	1	4	1	1	1	1	1	1
2	1	1	4	1	2	1	1	1	1
1	1	1	4	1	1	1	1	4	3
1	1	1	3	1	1	1	1	1	1
1	1	2	3	1	1	1	1	1	1
2	1	1	1	2	3	3	1	1	1
1	1	1	4	1	1	1	1	1	2
1	1	1	3	1	1	1	1	1	1
1	1	1	3	1	1	1	1	1	1
1	1	1	2	1	3	1	1	1	1
1	1	1	2	1	3	1	1	1	1
1	1	1	1	1	3	1	1	1	1
1	1	1	2	1	3	1	1	1	1
2	1	1	3	1	4	4	4	1	3
1	1	1	3	1	4	1	1	1	1
1	1	1	3	1	3	1	1	1	3
1	1	1	3	1	1	1	1	1	1
1	1	1	1	1	3	1	1	1	1
1	1	1	1	1	1	1	1	1	1

1	1	1	1	1	1	1	1	1	1
1	1	1	1	1	2	1	1	1	1
2	1	1	1	1	3	1	1	1	1
1	1	1	1	1	1	1	1	1	1
1	1	1	2	1	3	1	1	1	1
1	1	1	2	1	3	1	1	1	1
1	1	1	1	1	4	1	4	4	1
1	1	1	2	1	4	1	4	1	1
2	1	1	1	1	1	1	4	1	1
1	1	1	1	1	1	1	1	1	1
1	1	1	1	1	3	1	1	1	1
1	1	1	2	1	4	1	4	1	1
1	1	1	1	2	1	1	1	1	1
1	1	1	1	1					
1	1	1	1	1	1	1	1	1	1
1	1	1	1	1	1	1	1	1	1
1	1	1	1	1	4	4	4	1	4
1	1	1	1	1	1	1	1	1	1
1	1	1	1	1	3	1	1	1	1
1	1	1	1	1	1	1	1	1	1
1	1	1	1	1	1	1	1	1	1
1	1	1	1	1	3	1	1	1	1
1	1	1	1	1	1	1	1	1	1
1	1	1	1	1	3	1	3	1	1
1	1	1	1	1	2	1	3	3	1
1	1	1	2	1	1	1	1	1	1
1	1	1	4	1	1	1	1	1	1
1	1	1	1	1	3	1	2	1	1
4	3	1	2	1	1	2	3	1	1
1	1	2	2	1	2	1	3	3	1
1	1	1	1	1	1	1	1	1	1
2	1	1	3	1	2	1	3	2	2
1	1	1	1	1	2	1	1	1	1
3	1	3	1	1	2	2	1	1	2
1	1	1	4	1	3	3	3	3	2
1	1	1	1	1	1	1	1	1	1
1	1	1	2	1	1	1	1	2	1
1	1	1	1	1	1	1	1	1	1
3	1	1	2	1	2	2	3	3	2
3	1	1	4	1	1	1	1	1	1
1	1	2	3	1	1	1	1	1	1
1	1	1	4	1	3	1	1	1	1
1	1	1	2	1	1	1	2	1	1
1	1	2	3	1	2	1	1	1	3
1	1	1	2	1	4	4	4	4	4
1	1	1	3	1	1	1	1	1	1
2	2	1	1	1	3	3	3	1	1

3	2	1	2	1	2	3	1	3	3
2	1	1	1	1	4	1	4	1	1
1	1	1	3	1	3	1	1	1	1
1	2	1	2	1	4	2	3	1	4
1	1	1	1	1	1	1	1	1	1
1	1	1	1	1	1	1	1	1	1
1	1	1	1	1	4	1	1	2	1
1	1	1	1	1	1	1	1	1	1
1	1	1	1	1	4	1	1	1	1
1	1	1	3	2	3	1	1	1	1
1	1	1	1	1	1	1	1	1	1
1	1	1	2	1	1	1	1	1	2
1	2	1	2	1	4	1	1	1	1
1	1	1	1	1	1	3	1	1	1
1	1	1	1	1	1	1	1	1	1
1	1	1	1	1	4	3	4	4	4
1	1	1	1	1					
1	1	1	1	1	2	1	1	1	1
1	1	1	1	1	1	1	1	1	1
1	1	1	2	1	3	1	3	1	4
2	2	1	1	1	1	1	1	1	1
1	1	1	2	1	1	1	1	1	1
1	1	1	1	1	1	1	1	1	1
1	1	1	2	1	3	1	1	1	1
1	1	1	1	1	1	1	1	1	1
1	1	1	1	1	4	1	1	1	1
1	1	1	4	1	3	1	3	1	1
1	3	1	2	1	4	1	1	1	3
1	1	1	2	1	4	1	1	1	1
1	1	1	1	1	3	1	1	1	1
2	2	1	3	1	4	4	4	1	1
1	1	1	1	1	3	1	3	1	1
1	1	1	1	1	1	1	1	1	1
1	1	1	1	1	4	1	3	1	1
1	1	1	2	1	1	1	1	1	1
1	1	1	1	1	1	1	1	1	1
1	1	1	3	1	3	1	2	1	4
1	1	1	1	1	1	1	1	1	1
1	1	1	2	1	4	3	4	1	1
1	1	1	1	1	1	1	1	1	1
1	1	1	1	1	1	1	1	1	1
1	1	1	1	1	4	3	1	1	1
2	1	1	3	1	1	1	1	1	3
1	1	1	1	1	3	1	1	1	1
1	1	1	2	1	1	1	1	1	1
1	1	1	1	1	1	1	1	1	1
1	1	1	1	1	4	1	1	1	1

1	1	1	2	1	2	1	1	1	1
1	1	1	2	1	1	1	1	1	1
1	1	1	2	1	1	1	1	1	1
2	1	1	1	1	3	1	3	2	1
1	1	1	1	1	4	1	1	1	1
1	1	1	1	1	1	1	1	1	1
1	1	1	1	1	1	1	1	1	1
1	1	2	1	1	4	1	4	1	3
1	1	1	2	1	2	1	1	1	1
2	1	1	3	3	1	1	1	1	1
1	1	1	3	1	1	1	1	1	1
1	1	1	1	1	1	1	1	1	1
1	1	1	1	1	1	1	1	1	1
2	1	1	2	2	1	1	2	3	1
1	1	1	1	1		1	1	1	1
1	1	1	2	1	1	1	1	1	1
1	1	1	1	1	1	1	1	1	1
1	2	1	2	1	1	1	1	1	1
1	1	1	2	1	1	1	1	1	1
1	1	1	1	1	2	1	1	2	2
1	1	1	1	1	1	1	1	1	1
1	1	1	1	1	1	1	1	1	1
1	1	1	2	1	1	1	1	1	1
1	1	1	1	1	2	2	3	3	2
1	1	1	2	1	1	1	1	1	1
1	1	1	2	1	3	4	1	3	3
1	1	1	1	1					
1	1	1	1	1	1	1	1	1	1
1	1	2	2	1	4	4	4	4	4
2	1	1	1	1	1	1	1	1	1
1	1	2	1	1	4	1	1	1	1
1	1	1	5	1	1	1	1	1	1
1	1	1	1	1	1	1	1	1	1
1	1	1	3	1	2	1	1	1	2
1	1	1	1	1	3	3	3	1	1
1	1	1	2	1	1	1	1	1	1
1	1	1	4	1	1	1	1	1	1
1	1	1	1	2	2	2	3	1	2
1	1	1	3	1	1	1	1	1	3
1	1	1	1	1	1	1	1	1	1
1	1	1	2	1	4	4	4	3	4
1	1	3	1	1	1	1	1	1	1
1	1	1	2	1	1	1	1	1	1
1	1	1	3	1	1	1	1	1	1
1	2	1	2	1	1	1	1	1	1
1	1	1	1	1	1	1	3	3	1
1	1	1	1	1	1	1	1	1	1

1	1	1	2	1	1	1	1	1	1
1	1	1	1	1	1	1	2	1	1
1	1	1	1	1	3	3	3	3	3
1	1	1	1	1	3	1	2		3
2	1	1	1	1	4	4	4	4	4
2	1	1	4	1	4	2	4	1	3
1	1	1	4	1	4	4	4	3	3
1	1	1	4	1	4	4	4	3	3
1	1	1	2	1	2	1	1	1	1
1	1	1	1	1	3	1	1	3	1
1	1	1	1	1	1	1	1	1	1
1	1	1	1	1	3	1	1	1	1
1	1	1	1	1	1	1	1	1	1
1	1	1	1	1	3	1	3	1	3
1	1	1	1	1	1	1	1	1	1
1	1	1	2	1	1	1	1	1	1
2	1	1	2	3	3	1	3	1	2
1	1	1	1	2	3	1	1	1	3
1	1	1	2	1	1	1	1	1	1
1	1	1	3	1	1	1	1	1	1
1	1	1	2	1	4	1	1	1	1
1	1	1	3	1	3	1	3	3	3
1	1	1	1	1	1	1	1	1	1
1	1	1	1	1	1	1	1	1	1
1	1	1	2	1	1	1	1	1	1
1	1	1	1	1	3	3	3	3	3
1	1	1	1	1	3	2	1	1	2
1	2	4	3	4	1	1	1	1	1
1	1	1	3	1	1	1	1	1	1
1	1	1	3	1	1	3	1	1	1
1	1	1	3	1	3	3	3	3	3
1	1	1	1	1	1	1	2	1	3
1	1	1	1	1	1	1	2	1	3
1	1	1	4	1	1	1	1	1	1
1	1	1	2	1	4	1	2	4	4
1	1	1	2	1	4	1	2	4	4
1	1	1	1	1	1	1	1	1	1
1	1	1	1	1	1	1	1	1	1
1	1	1	1	1	2	1	1	1	1
1	1	1	4	1	4	1	1	1	2
1	1	1	1	1	1	1	1	1	1
2	1	1	1	1	1	1	1	1	1
1	1	1	2	1	4	4	4	4	4
2	2	1	3	1	1	1	1	1	1
2	1	1	2	1	1	1	1	1	1
1	1	1	4	1	1	1	1	1	1
1	1	1	2	1	1	1	1	1	1

1	1	1	2	1	1	1	1	1	1
1	1	1	1	1	1	1	1	1	3
1	1	1	1	1	2	1	1	1	1
2	4	1	2	1	3	1	3	2	1
1	1	1	1	1	3	3	1	1	1
3	1	2	1	3	3	3	3	3	3
3	1	2	1	3	3	3	3	3	3
1	1	1	3	2	3	1	1	1	3
1	1	1	1	1	3	3	1	3	1
1	1	1	2	1	4	4	4	3	4
1	1	1	1	1	1	4	4	3	4
1	1	1	1	1	1	4	4	3	4
1	1	1	1	1	3	1	3	3	1
1	1	1	1	1	3	3	1	1	3
3	1	1	2	1	1	1	1	1	4
1	1	1	1	1	1	1	1	1	4
1	1	1	4	2	3	1	3	1	1
2	1	1	1	1	1	1	2	4	4
1	1	1	2	1	3	3	1	1	1
1	1	1	2	1	3	2	4	3	4
1	1	1	1	1	1	1	1	1	1
1	1	1	3	1	3	1	3	1	3
1	1	1	2	1	2	3	1	1	1
1	1	1	1	1	1	1	1	1	1
1	1	1	1	1	1	1	1	1	1
1	2	1	2	1	1	1	1	1	1
1	2	1	1	3	1	1	1	1	1
1	1	1	1	1	1	1	3	2	1
1	2	1	1	1	3	3	3	3	3
1	1	1	1	1	2	1	1	1	3
1	1	1	1	1	1	1	1	1	1
1	1	1	2	1	1	1	1	1	1
1	1	1	1	1	1	1	1	1	1
1	1	1	4	1	1	1	1	1	1
1	1	1	1	1	1	1	1	1	1
1	1	1	2	1	3	2	1	1	1
1	1	1	2	1	3	2	1	1	1
1	1	1	2	1	1	1	1	1	1
1	1	1	2	1	1	1	1	3	1
1	1	1	1	1	3	1	1	1	1
2	1	1	2	1	1	1	4	3	1
1	2	1	2	1	1	1	1	2	4
2	1	1	4	1	1	1	1	1	1
1	1	1	1	1	1	1	1	1	1
1	1	1	2	1	1	1	1	1	1
1	1	1	1	1	3	1	3	1	3
1	1	1	4	1	1	1	1	1	1

1	1	1	1	1	1	1	3	1	1
1	1	1	3	1	3	1	1	1	1
2	1	2	1	2	1	2	3	1	1
1	1	1	1	1	3	3	3	3	3
1	1	1	1	1	1	1	1	1	1
1	1	1	1	1	1	1	1	1	1
1	1	1	2	1	1	3	3	3	3
1	1	1	2	1	1	2	3	3	3
1	1	1	2	1	1	1	1	1	1
1	2	1	4	1	4	4	4	4	3
1	1	1	1	1	3	3	3	3	3
1	1	1	1	1	1	1	1	1	1
1	1	1	1	1	1	1	1	1	1
1	1	1	1	1	1	1	1	1	1
1	1	1	1	1	4	4	4	4	4
2	2	2	2	2	1	1	1	1	1
1	1	1	1	1	2	1	4	1	4
2	2	2	2	1	2	2	2	2	2
1	1	1	2	1	1	1	1	1	2
1	1	1	1	1	1	1	1	1	1
1	1	1	1	1	1	1	1	1	1
1	1	1	2	1	1	1	1	1	1
1	1	1	1	1	1	1	1	1	1
1	1	1	2	1	4	4	4	1	1
1	1	1	1	1	1	1	1	1	1
1	1	1	2	1	1	1	1	1	1
1	1	1	1	1	1	1	1	1	1
1	1	1	1	1	1	1	1	1	1
1	1	1	1	1	1	1	1	1	1
1	1	1	1	1	1	1	1	1	1
1	1	1	1	1	4	4	4	4	4
1	1	1	2	1	1	1	1	1	1
1	1	1	3	1	3	3	3	1	1
1	1	2	2	2	1	1	1	1	1
3	3	2	2	2	2	3	2	3	2
2	2	2	2	2	4	4	4	4	4
1	2	1	1	1	1	1	1	1	1
1	1	1	1	1	3	3	3	1	1
3	1	3	3	3	1	1	1	1	1
1	1	1	1	1	1	1	1	1	1
1	1	1	1	1	2	1	1	1	1
2	2	2	2	2	1	1	1	1	1
1	1	1	1	1	4	4	3	3	3
1	1	1	1	1	1	1	1	1	1
1	1	1	1	2	1	1	1	1	1
1	1	1	1	1	3	3	3	2	2
1	2	1	1	1	1	1	1	1	1

1	2	2	2	1	1	1	1	1	1
2	1	1	4	1	2	2	3	3	3
2	2	2	2	2	3	3	3	2	2
1	1	1	1	1	3	3	3	3	3
1	1	1	1	1	3	3	3	3	3
1	1	1	1	1	1	1	1	1	3
2	2	1	2	1	3	3	3	3	3
1	2	1	3	2	4	4	4	4	4
1	2	1	2	1	1	1	2	1	1
3	1	1	3	1	3	1	1	1	1
1	1	1	2	1	4	4	4	4	4
1	1	1	1	2	1	1	1	3	1
2	2	1	3	3	4	3	4	4	3
2	1	2	2	4	3	4	4	4	1
1	1	1	3	1	4	1	3	1	1
1	1	1	1	1	3	3	1	1	3
1	1	1	1	1	4	1	1	1	1
1	1	1	3	2	1	1	1	1	1
1	1	2	2	1	1	1	1	1	1
1	1	1	4	1	2	1	1	1	1
1	1	1	1	1	1	2	4	4	3
1	1	1	1	1	4	1	4	4	4
3	1	1	3	1	4	1	1	1	1
1	1	1	4	1	3	1	3	1	1
1	1	1	4	1	3	1	3	1	1
1	1	1	1	1	1	1	1	1	2
1	2	1	1	1	3	1	1	1	1
2	1	1	2	1	3	1	1	1	1
1	1	1	1	1	4	4	4	4	1
1	1	1	2	1	1	1	4	1	1
1	1	1	1	1	4	4	4	4	4
2	1	1	1	2	4	1	3	1	1
2	1	1	1	2	4	1	3	1	1
1	1	1	4	1	2	1	1	1	1
2	2	1	1	1	4	4	4	4	2
1	1	2	1	1	3	3	3	1	4
1	2	1	2	1	4	4	1	4	4
1	1	1	3	1	1	1	1	1	1
1	1	1	1	1	1	1	1	1	1
1	1	1	3	1	4	1	3	1	1
1	2	1	2	1	4	4	4	4	4
1	1	1	3	1	4	3	4	4	1
1	1	1	1	1	4	1	1	1	1
1	1	1	3	2	4	3	3	2	1
1	1	1	1	1	4	4	1	1	1
1	1	1	3	1	1	1	4	2	4
1	1	1	1	1	1	1	1	1	1

1	1	1	1	2	1	1	1	1	1
1	1	1	3	1	1	1	3	1	1
1	1	1	1	1	1	1	1	1	1
1	1	1	2	2	2	2	2	3	4
1	1	1	1	1	1	1	1	1	1
1	1	1	2	1	3	1	1	2	1
1	1	1	1	1	1	1	1	1	1
1	1	1	1	1	1	1	1	1	1
1	1	1	1	1	4	1	1	1	1
1	1	1	1	1	1	1	1	1	1
1	1	1	1	1	3	1	1	1	1
1	1	1	2	1	3	1	1	1	1
1	1	1	2	2	1	1	1	1	1
1	1	1	2	1	1	1	1	1	1
1	1	1	2	1	1	1	1	1	1
1	1	1	1	1	1	1	1	1	1
1	1	1	2	1	4	1	1	1	1
1	1	1	1	1	1	1	1	1	1
1	1	1	1	1	3	1	1	1	1
1	1	1	1	1	3	1	1	1	1
1	1	1	2	1	3	1	1	1	1
1	1	1	2	1	3	1	1	1	1
1	1	1	1	1	4	1	1	2	1
1	3	1		1	1	1	2	1	1
1	1	1	1	1	1	1	1	1	1
1	1	1	1	1	3	1	1	1	1
1	1	1	1	1	1	1	1	1	1
1	1	1	1	1	3	1	1	1	1
1	1	1	1	2	3	1	1	1	1
1	1	1	1	1	3	4	3	4	4
1	1	1	1	1	4	4	3	4	3
1	1	1	1	1	4	4	4	4	1
1	1	1	1	1	1	1	1	1	1
1	1	1	1	1	1	1	1	1	1
1	1	1	2	1	3	1	3	1	1
1	1	1	1	1	1	1	1	1	1
1	1	1	2	1	3	1	1	1	1
1	1	1	2	2	1	1	1	1	1
1	1	1	1	1	1	1	1	1	1
1	1	1	1	1	3	1	1	1	1
1	1	1	1	1	3	1	1	1	1
1	1	1	1	2	2	2	1	1	1
1	1	1	1	1	3	1	1	1	1
1	1	1	2	1	3	1	1	1	1
1	1	1	1	1	1	1	1	1	1
1	1	1	1	1	3	3	1	3	1
1	1	1	1	1	1	1	1	1	1

2	1	1	1	2	3	2	3	3	1
2	1	1	1	2	3	2	3	3	1
1	1	1	1	1	2	1	1	1	1
1	3	1	1	1	1	1	1	1	1
1	1	1	1	1	2	1	1	1	1
1	1	1		1	3	3	3	3	3
1	2	2	2	2	3	1	1	1	1
1	1	1	1	1	3	1	1	1	4
2	1	1	1	1	3	2	1	1	1
1	1	2	1	1	4	1	4	4	4
1	1	1	1	1	4	1	1	1	1
1	1	1	1	1	1	1	1	1	1
1	1	1	1	1	2	2	2	2	2
1	1	1	2	1	3	2	3		3
1	1	1	2	1	3	2	3		3
1	1	1	4	1	1	1	1	1	1
1	1	1	1	1	2	1	1	1	2
1	3	1	4	1	2	1	1	1	1
1	3	1	2	1	1	1	1	1	1
3	2	2	4	2	2	1	1	1	1
1	1	1	2	1	1	1	1	1	1
1	1	1	2	1	1	1	1	1	1
1	1	1	4	1	1	1	1	1	1
1	1	1	1	1	1	1	1	1	1
1	2	1	4	4	4	4	4	4	4
1	1	1	1	1	3	1	1	1	1
1	1	1	2	1	1	1	1	1	1
1	1	1	1	3	1	1	1	1	1
1	2	1	4	1	2	2	2	1	1
1	1	1	1	1	1	1	1	1	4
1	1	1	2	1	3	1	4	1	1
1	1	1	1	1	4	1	4	1	1
1	1	1	2	1	4	1	4	1	1
1	1	1	2	2	4	1	1	1	1
1	1	1	4	1	1	1	1	1	1
1	1	1	1	1	3	1	1	1	1
1	1	1	1	1	1	1	1	1	1
1	1	1	2	1	1	1	1	1	1
1	1	1	1	1	3	1	3	1	1
1	1	1	3	1	2	1	1	2	1
1	1	1	1	1	3	2	3	3	3
1	1	1	2	1	2	1	1	1	2
1	1	1	2	3	2	1	3	1	2
1	1	1	2	1	3	3	1	1	1
1	1	1	1	1	2	1	2	1	1
1	2	1	5	1	4	4	4	1	1
1	1	1	1	4	4	4	4	1	1

1	1	1	1	4	4	4	4	1	1
1	1	1	1	1	1	1	1	1	1
1	2	1	4	1	1	1	2	3	3
1	3	2	2	1	1	1	3	1	2
1	1	1	1	1	1	1	1	1	1
1	1	1	1	1	1	1	1	1	1
1	1	1	2	1	1	1	1	3	1
1	1	1	2	1	1	1	1	3	1
1	1	1	3	1	1	1	3	2	1
1	1	1	1	1	2	1	1	1	1
2	1	1	2	1	1	1	1	1	4
1	1	1	3	1	1	1	1	1	1
2	1	1	3	2	1	1	3	1	1
2	1	1	2	1	1	1	3	1	1
1	1	1	4	2	4	4	4	4	3
1	1	1	1	1	1	1	1	1	1
1	1	1	2	1	1	1	1	1	1
1	1	1	1	1	3	1	1	1	1
1	1	2	2	1	2	2	3	3	3
1	1	1	2	1	4	1	4	1	1
1	1	1	1	1	4	2	1	1	1
1	1	1	1	1	1	1	1	1	1
1	1	1	2	1	1	1	3	1	1
1	1	1	4	1	1	1	1	1	1
1	1	1	3	1	4	4	4	4	4
1	3	1	4	1	4	4	4	4	4
2	1	1	1	1	1	1	1	1	1
1	1	1	1	1	1	1	1	1	1
2	1	1	2	1	4	3	4	2	4
1	1	1	1	1	2	2	3	1	2
1	1	1	3	1	1	1	3	1	1
1	1	1	2	1	1	1	1	1	1
1	1	1	3	1	3	1	3	1	1
1	1	1	2	1	3	1	3	4	4
1	4	1	1	1	3	2	1	1	1
1	1	1	2	1	1	1	1	1	1
1	1	1	2	1	3	2	2	1	3
1	1	1	3	1	1	1	3	1	1
1	1	1	1	1	1	1	1	1	1
1	1	1	3	1	1	1	1	1	1
1	1	1	1	1	1	1	1	1	1
1	1	1	3	1	1	1	1	1	1
1	1	1	2	1	3	1	1	1	1
1	1	1	3	1	2	2	1	1	3
1	1	1	1	1	3	1	1	1	1
1	1	1	4	1	1	1	1	1	1
1	1	1	4	2	1	1	3	1	1

1	1	1	2	1	1	1	1	1	1
1	1	1	2	1	1	1	1	1	2
1	1	1	2	1	1	1	1	1	1
1	1	1	1	1	1	1	1	1	1
1	1	1	1	1	3	1	1	1	1
1	1	1	1	1	3	1	1	1	1
1	1	1	3	1	1	1	1	1	1
1	1	1	1	1	3	2	3	1	1
1	1	1	1	1	1	1	1	1	1
1	1	1		1	4	4	4	1	1
1	1	1	2	1	3	1	3	1	2
1	1	1	1	1	3	1	1	1	1
1	1	1	3	2	1	1	2	1	2
1	2	1	1	1	2	1	1	1	1
1	1	1	1	1	1	1	1	1	1
1	1	1	2	3	3	3	3	1	3
1	1	1	1	1	1	1	1	1	1
1	1	1	1	1	3	3	3	1	1
1	1	1	3	1	2	1	2	1	2
1	1	1	1	1	1	1	1	1	1
1	1	1	2	1	3	1	1	1	2
1	1	1	3	1	1	1	2	1	1
1	1	1	3	1	1	1	1	1	1
1	1	1	2	1	1	1	1	1	1
1	1	1	1	1	1	1	1	1	1
1	2	1	3	1	3	1	3	1	1
1	1	1	3	1	3	3	3	1	1
1	1	1	3	1	1	1	1	1	1
1	1	1	3	1	1	1	1	1	1
1	1	1	4	1	3	1	1	1	1
1	1	1	4	1	1	1	1	1	1
1	1	1	2	2	4	4	4	3	2
1	1	1	3	1	1	1	1	1	1
1	1	1	1	1	1	1	1	1	1
1	1	1	1	1	3	1	1	1	1
1	1	1	2	1	3	1	3	1	1
2	1	1	3	1	3	1	1	1	1
1	1	1	3	1	1	1	1	1	1
1	1	1	3	1	1	1	1	1	1
1	1	1	4	1	1	1	1	1	1
1	1	1	3	1	3	1	1	1	1
1	1	1	4	1	1	2	2	1	2
2	1	1	4	1	4	4	1	1	1
2	1	1	3	1	1	1	1	1	1
1	1	1	3	1	3	1	3	1	1
1	1	1	1	1	1	1	1	1	2
1	1	1	3	1	1	1	1	1	1

1	1	1	3	1	1	1	1	1	1
3	1	1	3	1	1	1	1	1	1
1	1	1	3	1	1	1	1	1	3
1	1	1	1	1	3	1	1	1	1
1	1	1	1	1	3	1	3	1	3
1	1	1	3	1	2	1	1	1	3
1	1	2	2	1	3	3	1	1	1
1	1	1	3	1	1	1	1	1	1
1	1	1	1	1	3	1	1	1	1
2	1	1	3	1	4	1	3	1	1
1	1	1	3	1	1	1	1	1	2
1	1	1	1	1	1	1	1	1	1
1	1	1	1	1	1	1	1	1	1
1	1	1	1	1	1	1	1	1	4
1	1	1	1	1	1	1	1	1	1
1	1	1	1	1	4	1	3	3	3
1	1	1	1	1	1	1	1	1	1
1	1	1	1	1	1	1	1	1	1
1	1	1	1	1	1	1	1	1	1
1	1	1	1	1	1	1	1	1	1
1	1	1	1	1	1	1	1	1	1
1	1	1	1	1	2	1	1	1	1
1	1	1	1	1	4	1	1	4	1
1	1	1	1	1		1	1	1	1
1	1	1	1	1	1	1	1	1	1
1	1	1	1	1	1	1	1	1	1
1	1	1	1	1	1	1	1	1	1
1	1	1	1	1	1	1	1	1	1
1	1	1	1	1	4	1	3	3	3
1	1	1	1	1	1	1	1	1	1
1	1	1	1	1	1	1	1	1	1
2	1	1	1	1	1	1	1	1	1
1	1	1	1	1	4	1	1	1	4
1	1	1	1	1	4	4	4	4	4
2	1	1	1	1	2	1	3	1	1
1	1	1	1	1	1	1	1	1	1
1	1	1	1	1	1	1	1	1	1
1	1	1	1	1	1	1	1	1	1
3	1	1	4	1	3	1	4	4	1
2	1	1	1	1	1	1	1	1	1
1	1	1	3	1	4	1	1	1	1
1	1	1	2	1	1	1	1	1	1
1	1	1	3	3	1	1	2	1	1
1	1	1	1	1	2	2	1	3	2
1	1	1	1	1	1	1	1	1	1
3	1	1	3	1	4	1	1	4	4
1	1	1	3	1	1	1	1	1	1

1	1	1	2	1	1	1	1	1	1
1	2	1	3	1	4	1	3	4	1
3	3	1	3	1					
1	1	1	2	1	1	1	1	1	1
1	1	1	2	2	1	1	1	1	1
1	1	1	2	1	1	1	1	1	1
1	3	1	2	1	1	1	1	1	2
1	1	1	2	1	1	1	1	1	1
1	1	1	3	1	1	1	4	2	1
1	1	1	4	1	2	1	1	1	1
3	3	1	4	1	2	2	2	1	1
1	1	1	1	1	3	1	1	1	1
1	1	1	1	1	3	1	1	1	1
1	1	2	1	1	1	1	3	1	1
1	1	1	1	1	1	1	1	1	1
2	2	1	2	1	3	1	1	1	1
1	1	1	2	1	1	1	1	1	1
2	1	1	1	1	1	1	4	1	4
2	1	1	1	1	3	1	1	1	1
1	1	1	1	1	1	1	1	1	1
1	1	2	1	1	4	4	4	4	1
1	1	1	2	1	3	1	3	2	1
1	1	1	2	1	1	1	1	1	1
1	1	1	1	1	4	1	1	1	1
1	1	1	2	1	1	1	1	1	1
1	1	1	2	2	1	1	3	1	1
1	1	1	2	1	3	1	1	1	1
1	1	1	2	1	3	1	1	1	1
1	1	1	2	1	1	1	3	1	1
1	1	1	2	1	1	1	3	1	1
1	1	1	1	1	1	1	1	1	1
1	1	1	2	1	1	1	1	1	1
1	1	1	1	1	1	1	1	1	1
1	1	1	1	1	1	1	1	1	1
1	1	1	1	1	1	1	3	1	4
1	1	1	1	1	1	1	3	1	4
1	1	1	2	1	2	1	3	1	1
1	1	1	2	1	3	1	1	1	1
2	1	1	1	1	1	1	1	1	3
1	1	1	1	1	1	1	1	1	1
1	1	1	1	1	1	1	1	1	1
1	1	1	1	1	1	1	1	1	1
1	1	1	1	1	1	1	1	1	1
1	1	1	1	1	4	1	4	1	1
1	1	1	1	1	3	1	1	1	1
1	1	1	2	2	1	1	1	1	1
1	1	1	1	1	3	1	1	1	1

1	1	1	2	1	2	1	1	1	2
1	1	1	1	1	1	1	1	1	1
1	1	1	1	1	3	1	3	1	3
1	1	1	2	1	2	1	1	1	1
1	1	1	2	1	1	1	3	1	1
1	1	1	2	1	1	1	1	1	1
1	1	1	1	1	4	1	1	1	1
1	1	1	1	1	1	1	1	1	1
1	1	1	1	1	1	1	1	1	1
1	2	1	1	1	1	1	1	1	1
1	1	1	1	1	2	1	1	1	1
1	1	1	3	1	2	1	1	1	1
1	1	1	1	1	4	1	1	1	1
1	1	1	1	1	4	1	1	1	1
1	1	1	2	1	4	4	1	1	4
1	1	1	1	1	4	4	1	1	1
1	1	1	1	1	1	1	1	1	1
1	1	1	1	1	4	4	3	3	1
1	1	1	1	2	1	1	1	1	1
1	1	1	1	1	1	1	1	1	1
1	1	1	1	1	4	1	1	1	4
1	1	1	1	1					
1	1	1	1	1	3	1	1	1	1
1	1	1	1	1	1	1	1	1	1
1	2	2	1	1	3	2	1	1	2
1	1	1	1	1	1	1	1	1	1
1	1	1	1	1	4	4	1	1	4
2	1	1	2	1	3	1	2	2	1
1	1	1	1	1	1	1	1	1	1
1	1	1	2	1	1	1	1	1	1
1	1	1	1	1	1	1	1	1	1
1	1	1	2	1	1	1	1	1	1
2	1	1	1	1	4	1	4	1	4
1	3	1	1	1					
1	1	1	2	1	1	1	1	1	1
1	1	1	1	1	3	1	1	1	4
1	1	1	1	1	1	1	1	1	1
3	1	1	2	1	4	1	1	1	1
1	1	1	1	1	1	1	1	1	1
3	1	1	3	1	4	4	4	4	4
1	1	1	1	1	1	1	3	1	1
1	1	1	2	1	3	1	1	1	1
1	1	1	3	1	1	1	3	1	4
1	1	1	1	1	1	1	1	1	1
2	1	1	1	1	1	1	1	1	1
1	1	1	1	1	4	1	1	1	1
1	1	1	1	1	3	1	1	1	1

1	1	1	1	1	1	1	1	1	1
1	1	1	1	1	1	1	1	1	1
1	1	1	1	1	1	1	1	1	1
1	1	1	1	1	1	1	1	1	1
1	1	1	1	1	1	1	1	1	1
1	1	1	1	1	1	1	1	1	1
1	1	1	2	1	4	4	4	4	1
1	1	1	1	1	1	1	1	1	1
1	1	1	1	1	1	1	2	2	2
1	1	1	2	1	1	1	1	1	1
1	1	1	1	1	1	1	1	1	1
1	1	1	1	1	1	1	1	1	1
1	1	1	1	1	1				
1	1	1	1	1	1	1	1	1	1
1	1	1	1	1	1	1	1	1	1
1	1	1	3	1	2	1	2	2	1
1	1	1	1	1	2	2	2	3	3
1	1	1	1	1	1	1	1	1	1
3	2	1	1	1	3	3	3	1	1
1	1	1	1	1	1	1	1	1	1
1	1	1	1	1	1	2	2	1	1
1	1	1	4	1	3	1	1	1	3
1	1	1	3	1	1	1	3	3	1
1	1	1	1	1	1	1	1	1	1
1	1	1	1	1	1	1	1	1	1
1	1	1	1	1	2	1	1	1	1
1	1	1	2	1	4	4	4	4	4
1	1	1	1	1	1	1	1	1	1
1	1	1	1	1	4	3	4	4	4
1	1	1	1	1	4	3	4	4	4
1	1	1	1	1	1	1	1	1	1
2	1	1	2	1	2	1	1	1	3
1	1	1	1	1	1	1	1	1	1
1	1	1	1	1	3	1	3	1	1
2	1	1	2	1	1	1	1	1	1
1	1	1	2	1	4	2	4	4	1
1	1	1	3	1	1	1	1	1	1
1	1	1	3	1	1	1	1	1	1
1	1	1	1	1	1	1	1	1	1
1	1	1	2	1	1	1	1	1	1
1	1	1	3	1	1	1	1	1	1
1	1	1	1	1	1	1	1	1	1
1	1	1	4	1	1	1	1	1	1
1	1	1	1	1	1	1	1	1	1
2	3	2	2	3	1	1	1	1	1
2	3	2	2	3	1	1	1	1	1
1	1	1	2	1	1	1	1	1	1

1	1	1	2	1	1	1	1	1	1
1	1	1	2	1	1	1	3	1	1
1	1	1	4	1	4	1	4	1	4
1	1	1	1	1	3	1	1	1	1
1	1	1	2	3	4	1	1	1	4
1	1	1	2	1	3	1	4	3	4
1	1	1	2	1	3	4	4	4	4
1	1	1	4	1	3	1	1	1	1
1	1	1	1	1	1	1	1	1	1
1	2	1	2	1	1	1	3	2	3
1	1	1	1	1	1	1	1	1	1
1	1	1	1	1	1	1	1	1	1
1	1	1	4	1	1	1	1	2	1
2	2	1	2	1	3	2	2	1	1
1	1	1	3	1	4	4	4	4	4
1	2	1	2	3	4	2	4	2	3
1	1	1	1	1	1	1	1	1	1
1	1	1	1	1	1	1	1	1	1
1	1	1	1	4	1	1	2	1	1
1	2	1	2	1	1	1	1	1	1
1	2	1	3	1	3	3	3	2	1
1	1	1	1	1	1	1	1	1	1
1	2	1	1	1	1	1	3	1	1
1	1	1	1	1	3	1	1	1	1
1	1	1	4	1	4	4	1	3	4
1	1	1	4	1	2	1	1	1	1
1	1	1	1	1	1	3	3	3	3
2	1	1	1	1	2	3	3	3	1
2	1	1	1	1	2	3	3	3	1
1	2	2	2	1	1	1	1	1	1
2	1	1	2	1	1	1	1	1	1
4	2	1	2	2	1	1	3	1	3
1	1	1	1	1	3	1	3	3	3
1	1	1	1	2	1	1	1	1	1
1	1	1	2	1	1	1	1	1	1
1	1	1	3	1	4	4	4	4	4
2	4	1	1	1	4	3	3	4	4
2	2	1	2	1	2	2	3	3	2
1	1	1	1	1	4	4	4	4	1
1	1	1	2	1	4	4	3	3	1
3	2		1		1	1	1	1	1
1	1	1	2	1	2	1	1	1	1
1	1	1	1	1		1	3	3	3
2	1	1	1	1	3	3	4	1	4
2	1	1	1	1	3	3	4	1	4
1	1	1	2	1	3	1	1	1	1
1	1	1	2	1	1	1	1	1	1

1	1	1	2	1	3	1	1	1	1
1	2	1	2	1	1	1	1	1	1
1	2	1	3	1	3	1	1	3	1
1	2	1	2	1	2	1	1	1	3
1	1	1	1	1	3	3	3	3	3
1	1	1	1	1	3	3	3	3	3
1	1	1	3	1	4	4	3	3	3
1	1	1	3	1	4	4	3	3	3
1	1	1	3	1	4	4	3	3	3
1	1	1	2	1	3	1	1	1	1
1	1	1	1	1	3	1	3	1	1
1	1	1	1	1	1	1	1	1	1
1	1	1	3	1	4	1	3	3	1
1	1	1	1	1	3	3	1	1	1
2	1	1	2	1	3	3	3	3	1
1	1	1	1	1	1	1	1	1	1
3	3	2	2	3	3	3	2	2	2
1	1	1	1	1	1	1	1	1	1
1	1	1	1	1	1	1	1	1	1
1	1	1	2	1	3	3	1	1	1
1	1	1	1	1	4	4	4	4	4
1	1	1	1	1	4	1	1	1	1
1	2	1	1	2	4	2	3	1	1
1	1	1	1	1	2	2	2	2	2
1	2	1	2	1	1	1	1	1	1
1	1	1	1	1	1	1	2	1	1
1	1	1	1	1	1	1	1	1	1
1	1	1	1	1	1	1	1	1	1
1	2	1	3	1	3	1	1	1	1
1	1	1	1	1	1	1	1	2	2
1	1	1	1	1	1	1	1	1	1
1	1	1	1	1	1	1	1	1	1
2	2	2	2	2	4	4	4	4	4
1	1	1	1	1	3	3	1	1	2
1	1	1	1	1	1	1	1	1	1
1	1	1	3	1	3	1	1	1	3
1	1	1	1	1	1	1	2	1	1
1	1	1	1	1	1	1	2	2	1
1	1	1	2	1	3	3	3	3	3
1	1	1	1	1	1	1	1	1	1
2	2	2	2	3	3	3	3	3	3
1	1	1	1	1	1	1	1	1	1
1	1	1	1	1	1	1	1	1	1
2	1	2	1	2	2	2	2	1	1
2	2	2	2	2	3	3	3	3	3
2	1	2	1	1					
1	2	1	2	1	1	1	1	1	1

1	1	1	1	1	1	1	1	1	1
1	1	1	2	1	3	1	1	1	1
1	2	1	2	1	3	3	3	1	1
1	1	1	1	1	4	4	4	4	4
1	1	1	1	1	1	1	1	1	1
1	2	1	2	1	1	1	1	1	1
1	1	1	1	1	1	1	1	1	1
1	1	1	1	1	1	1	1	1	1
1	1	1	2	1	1	1	1	1	1
1	1	1	1	1	3	3	1	1	3
1	1	1	3	1	2	1	3	2	1
1	1	1	2	1	3	1	1	1	1
2	2	2	2	2	1	1	1	1	1
1	1	1	1	1	1	1	1	1	1
1	1	1	1	1	1	1	1	1	1
1	1	1	1	1	1	1	1	1	1
1	1	1	1	1	2	2	2	2	2
2	1	2	1	2	2	1	1	1	1
1	1	1	1	1	1	1	1	2	2
1	1	1	1	1					
1	1	2	1	2	1	1	1	2	1
1	1	1	1	1	1	1	1	1	1
1	1	1	3	2	3	1	3	3	3
1	1	1	2	1	1	1	1	1	1
3	1	1	3	1	1	1	1	1	1
2	1	1	2	1	2	1	2	2	2
1	2	2	1	2	2	1	1	1	1
1	1	1	1	1	1	1	2	1	1
1	1	1	1	1	1	1	1	1	1
1	1	1	1	1	2	2	2	2	3
1	2	1	2	1	3	1	3	1	1
1	3	3	3	3	1	1	1	1	1
2	2	2	2	2	1	1	1	1	1
1	1	1	1	1	1	1	1	1	1
2	1	2	1	2	1	1	1	1	1
2	2	2	2	2	4	4	4	4	4
1	1	1	1	1	4	3	3	3	3
1	1	1	1	1	1	1	1	1	1
1	1	1	1	1	1	1	1	1	1
1	1	1	2	1	3	3	3	1	1
1	1	1	1	1	1	1	1	1	1
4	4	4	4	4	1	1	1	1	1
1	1	2	3	1	4	4	1	1	3
1	1	1	3	1	1	1	1	1	3
1	1	1	1	1	3	1	1	1	1
1	1	1	3	1	4	3	3	3	3
1	1	1	1	1	4	1	1	1	1

1	3	1	3	1	4	1	3	1	1
3	3	1	2	2	4	1	1	1	1
1	1	1	2	1	4	2	4	4	4
2	1	1	2	1	3	1	1	1	1
1	1	1	3	1	1	1	1	1	1
2	2	1	3	1	4	4	3	3	1
1	1	1	2	1	4	4	2	1	1
1	1	1	1	1	1	1	1	1	1
2	1	1	2	1	4	4	4	4	4
1	1	1	1	1	4	1	1	1	1
2	1	1	2	1	1	1	1	1	2
2	1	1	2	1	1	1	1	1	2
1	1	2	1	1	4	4	4	4	4
1	1	2	1	1	4	4	4	4	4
1	1	1	1	4	4	4	4	4	4
1	1	1	4	1	4	3	4	3	3
1	1	1	3	1	4	4	1	4	1
2	1	1	1	1	4	1	4	4	1
1	1	1	3	1	3	1	2	1	1
1	1	1	1	1	4	1	4	4	1
1	1	1	1	1	4	1	4	4	1
1	1	1	3	1	4	4	3	1	1
1	1	1	1	1	3	1	1	1	1
1	1	1	3	1	4	3	3	1	1
1	1	1	2	1	3	1	1	1	1
1	1	1	1	1	4	1	3	4	3
1	1	1	2	1	1	1	1	1	4
1	1	1	1	1	4	3	1	1	1
1	1	1	1	1	4	4	4	4	4
1	1	1	1	1	4	4	4	4	4
1	1	1	4	1	4	1	3	3	3
1	1	1	4	2	1	1	1	1	1
1	1	1	4	1	1	1	3	1	1
1	1	1	3	1	4	1	4	4	4
1	1	1	3	1	4	3	3	2	3
1	1	1	3	1	4	1	3	3	1
1	1	1	4	1	4	4	4	4	4
1	1	1	2	1	3	1	3	3	1
1	1	1	1	1	1	3	3	1	4
1	4	1	1	1	1	1	1	1	1
1	4	1	1	1	1	1	1	1	1
1	1	1	1	1	1	1	1	1	1
1	1	1	3	1	4	1	1	1	1
2	1	1	1	1	1	1	1	1	1
1	1	1	2	1	3	3	1	1	3
1	1	1	1	1	3	1	1	1	1
1	1	1	2	1	1	1	1	1	1

1	1	1	1	1	3	1	1	1	1
1	1	1	1	1	1	1	1	1	1
1	1	1	1	1	1	1	1	1	1
1	1	1	2	2	1	1	1	1	1
1	1	1	1	1	3	1	1	1	1
1	1	1	2	1	2	1	1	1	1
1	1	1	1	1	1	1	1	1	1
1	1	1	1	1	1	1	1	1	1
1	2	1	2	1	3	1	1	1	1
1	1	1	1	2	1	1	1	1	1
1	1	1	1	2	1	1	1	1	1
1	1	1	1	1	1	1	1	1	1
1	1	1	1	1	1	1	1	1	1
1	1	1	2	1	4	4	3	2	3
1	1	1	1	1	3	1	1	1	1
1	1	1	1	1	3	1	1	1	1
1	1	1	2	1	4	1	4	1	1
1	1	1	1	1	1	1	1	1	1
1	1	1	2	1	1	1	1	1	1
1	1	1	1	1	1	1	1	1	1
1	1	1	1	1	3	1	1	1	1
1	1	1	2	1	1	1	1	1	1
2	1	1	1	1	3	1	1	1	3
1	1	1	1	2	3	3	1	3	3
1	1	1	1	1	1	1	1	1	1
1	1	1	1	1	1	1	1	1	1
1	1	1	1	1	3	1	1	1	1
1	1	1	1	1	1	1	1	1	1
1	1	1	1	1	1	1	1	1	1
2	1	1	1	1	1	1	1	1	1
1	1	1	1	1	1	1	1	1	1
1	1	2	1	3	1	1	1	1	1
1	1	1		1	1	1	1	1	1
1	1	1	1	1	1	1	1	1	1
1	1	1	2	1	4	4	4	1	4
1	1	1	2	1	1	1	1	1	1
1	1	1	1	1	3	1	1	1	1
1	1	1	1	1	1	1	1	1	3
2	2	1	4	1	1	1	1	1	1
2	2	1	4	1	1	1	1	1	1
1	1	1	2	1	2	1	1	1	1
1	1	1	3	1	1	1	2	1	1
1	1	1	2	1	1	1	1	1	2
1	1	1	2	1	3	1	3	3	1
1	1	1	4	1	1	1	1	1	1
1	1	1	4	1	1	1	1	1	1
1	1	1	1	1	1	1	1	1	1

1	1	1	2	2	4	4	3	3	3
1	1	1	3	1	1	1	1	1	1
1	1	1	4	1	1	1	1	1	1
3	1	1	4	1	1	1	1	1	1
3	1	1	1	1	4	4	4	4	4
1	2	2	2	2	3	3	2	2	2
1	1	1	1	1	2	2	3	1	1
2	1	1	2	1	3	3	1	1	1
1	1	1	3	1	4	4	4	1	4
1	1	1	3	1	3	1	4	1	4
1	1	1	1	1	4	4	4	4	4
1	1	1	4	1	3	3	2	1	1
1	1	1	2	1	1	1	1	1	3
1	1	1	1	1	3	1	1	1	4
1	1	2	3	1	4	1	4	4	4
1	1	1	1	1	1	1	1	1	1
1	1	1	2	1	1	1	1	1	1
1	1	1	1	1	4	3	3	4	3
1	1	1	1	1	2	3	3	1	1
1	1	1	2	1	1	1	1	1	1
1	1	1	2	1	2	1	1	1	1
1	1	1	1	1	1	1	1	1	1
1	1	1	1	1	1	1	1	1	1
1	1	1	1	1	1	1	1	1	1
1	1	1	1	1	1	1	1	1	1
3	1	1	1	1	1	1	1	1	1
1	1	1	2	1	1	2	2	1	3
1	1	1	2	2	3	3	4	3	3
1	1	1	1	1	4	3	4	3	3
1	1	1	1	1	4	3	4	3	3
1	2	1	2	1	4	4	4	3	4
1	2	1	2	1	4	4	4	3	4
1	1	1	2	1	4	1	1	1	1
1	1	1	1	2	1	1	1	1	1
1	1	1	1	1	1	1	1	1	1
1	1	1	1	1	1	1	1	1	1
2	1	1	4	1	4	3	3	3	3
2	1	1	4	1	1	1	1	1	1
1	1	1	2	1	1	1	1	1	1
1	1	1	2	1	3	1	1	1	1
1	1	1	1	1	3	1	1	1	1
1	1	1	2	1	3	1	1	1	1
1	1	1	2	1	3	1	1	1	1
1	1	1	2	1	3	1	1	1	1
1	1	1	4	1	2	1	2	1	1
1	1	1	3	1	1	1	1	1	1
1	1	1	3	1	3	2	1	1	1

2	1	1	1	1	3	3	3	3	3
1	1	1	1	1	3	3	3	3	4
1	1	1	1	1	4	4	4	4	4
1	1	1	4	3	3	2	3	1	4
1	1	1	3	1	3	1	3	1	3
1	1	1	3	1	1	1	1	1	2
1	1	1	1	1	1	1	1	1	1
1	1	1	1	1	3	1	1	1	1
1	1	1	1	1	4	1	1	1	3
2	2	1	3	1	1	1	1	1	2
1	1	1	3	1	3	1	1	1	1
1	1	1	1	1	3	1	1	1	1
1	1	1	2	1	1	1	1	1	1
1	1	1	3	1	3	1	1	1	1
1	1	1	3	1	3	1	1	2	2
1	1	1	2	1	1	1	1	1	2
1	1	1	2	1	1	1	1	1	1
1	1	1	3	1	2	1	1	1	1
1	1	1	3	1	2	1	1	1	2
2	1	1	1	1	1	1	1	1	1
1	1	1	1	1	3	1	1	1	1
1	1	1	4	1	1	1	1	1	1
1	1	1	1	1	4	1	1	1	1
1	1	1	3	1	1	1	1	1	1
1	1	1	1	1	3	1	2	2	2
1	1	1	1	1	1	1	1	1	1
1	1	1	1	1	1	1	1	1	1
1	1	1	1	1	1	1	1	1	1
1	1	1	3	1	3	1	1	1	1
1	1	1	1	1	1	1	1	1	1
1	1	1	2	1	1	1	1	1	1
1	1	1	3	1	1	1	1	1	1
1	1	1	2	1	3	1	1	1	1
1	1	1	2	1	2	1	1	1	1
1	1	1	2	1	3	3	4	1	3
1	1	1	1	1	3	3	1	1	2
1	1	1	3	1	1	1	1	1	1
1	1	1	1	1	3	1	1	1	1
1	1	1	3	1	3	1	1	1	1
1	1	1	4	1	1	2	1	1	2
1	1	1	3	1	4	4	4	4	4
1	1	1	2	1	1	1	3	1	3
1	2	1	4	1	1	1	1	1	1
1	1	1	3	1	3	1	1	1	1
1	1	1	3	1	2	1	1	1	1
1	1	1	3	1	1	1	1	1	1
1	3	1	3	1	1	1	1	1	1

1	1	1	3	1	4	2	3	4	3
1	1	1	1	1	1	1	1	1	1
1	1	1	3	1	1	1	1	1	1
1	1	1	2	1	3	1	1	1	1
1	1	1	1	1	1	1	1	1	1
1	1	1	1	1	1	1	1	1	1
1	1	1	1	1	1	1	1	1	1
1	1	1	1	1	1	1	1	1	1
1	1	1	1	1	1	1	1	1	1
1	1	1	1	1	1	1	1	1	1
1	1	1	1	1	1	1	1	1	1
1	1	1	1	1	1	1	1	1	1
1	1	1	1	1	1	1	1	1	1
1	1	1	1	1	1	1	1	1	1
1	1	1	1	1	2	1	3	1	1
2	1	1	1		3	1	3	3	2
	1	1	1	1	1	1	1	1	1
1	1	1	1	1	1	1	1	1	1
2	1	1	1	1	3	1	1	1	1
2	1	2	1	1	1	1	1	1	1
1	1	1	1	1	1	1	1	1	1
2	1	1	1	1	1	1	1	1	1
1	1	1	1	1					
1	1	1	4	1	1	1	1	1	1
1	1	1	1	1	1	1	1	1	1
1	1	1	3	2	1	1	3	1	1
3	3	3	3	3	3	1	1	1	1
3	3	3	3	1	1	1	1	1	1
1	1	1	2	1	1	1	1	2	1
1	1	1	1	1	2	2	1	1	1
1	1	1	1	1	1	1	1	1	1
1	1	1	1	1	1	1	1	1	1
1	1	1	1	1	1	1	1	1	1
1	1	1	1	1	2	4	4	1	1
1	1	1	4	1	1	1	1	1	1
3	2	4	3	1	3	1	3	2	1
1	1	1	3	1	1	1	1	3	3
1	1	1	1	1	1	1	1	1	1
1	1	1	4	1	2	1	1	1	1
1	1	1	2	1	3	3	2	3	1
2	1	1	1	1	1	1	1	1	1
1	1	1	3	1	2	1	1	2	1
1	1	1	3	1	1	1	1	1	1
1	1	1	4	1	4	1	2	3	1
1	1	1	4	1	3	1	1	1	1
1	1	1	4	1	3	3	4	1	2
1	1	1	4	1	4	1	1	1	1

1	1	1	1	1	4	4	4	3	3
1	1	1	1	1	2	1	1	1	1
1	1	1	1	1	1	1	1	1	1
1	1	1	2	1	3	1	1	1	1
1	1	1	2	1	3	1	1	1	1
1	2	1	1	1	4	2	1	1	1
1	1	1	2	1	3	3	1	1	1
1	1	1	1	1	3	1	1	1	1
1	1	1	2	1	1	1	1	1	1
1	1	1	1	1	2	1	3	2	4
1	1	1	2	1	1	1	2	1	1
1	1	1	1	1	3	1	1	1	1
1	1	1	1	1	3	1	1	1	1
1	1	1	2	1	3	1	1	1	1
1	1	1	1	1	4	1	1	1	1
1	1	1	2	1	3	1	3	1	1
1	1	1	1	1	4	1	1	1	1
1	1	1	2	1	1	1	1	1	1
3	1	1	1	4	1	1	1	1	1
1	1	1	1	1	4	1	1	1	4
1	1	1	1	1	3	1	1	1	1
1	1	1	3	3	3	1	1	1	1
1	1	1	1	1	4	1	4	1	1
1	1	1	1	3	1	1	1	1	1
1	1	1	2	1	1	1	1	1	1
1	1	1	2	1	3	1	1	1	1
1	1	1	1	1	1	1	1	1	3
1	1	1	1	1					
1	1	1	1	1	4	1	1	1	1
1	1	1	1	1	1	1	1	1	1
1	1	1	1	1	3	1	1	1	1
1	1	1	1	1	4	3	3	1	1
1	1	1	2	1	1	1	1	1	1
1	1	1	2	1	1	1	4	1	1
1	1	1	1	1	1	1	1	1	1
3	1	1	2	1	3	1	2	1	1
1	1	1	1	1	1	3	3	1	3
1	1	1	1	1	1	1	1	1	1
1	1	1	1	1	1	1	1	1	1
1	1	1	1	1	1	1	1	1	1
1	1	1	2	1	1	1	1	1	1
1	1	1	1	1	1	1	1	1	1
1	1	1	1	1	3	1	1	1	1
1	1	1	1	2	4	4	4	4	4
1	2	1	1	1	4	3	1	4	4
1	1	1	3	1	1	1	1	1	1
1	1	1	1	1	1	1	1	1	1

1	1	1	1	1	4	1	4	1	4
1	1	1	1	1	1	1	1	1	1
1	1	1	1	1	1	1	1	1	1
1	1	1	1	1	1	1	1	1	1
1	1	1	1	1	1	1	1	1	1
1	1	1	2	1	1	1	1	1	1
1	1	1	2	1	1	1	1	1	1
1	1	1	1	1	1	1	1	1	1
1	1	1	2	1	1	1	1	1	1
1	1	1	1	1	1	1	1	1	1
1	1	1	3	1	3	3	1	1	1
1		1	1	1	4	1	1	1	1
1	1	1	2	1	4	1	3	1	1
3	3	1	1	1	4	1	3	2	1
1	1	1	2	1	4	1	1	1	1
1	1	1	1	1	3	1	1	1	1
3	1	1	1	1	3	1	1	1	1
1	1	1	2	2	2	1	1	1	2
1	1	1	1	1	3	1	1	1	1
3	1	3	4	1	4	4	4	2	3
1	1	1	1	1	3	2	1	1	1
1	1	1	1	1	1	1	1	1	1
1	1	1	1	1	1	1	1	1	1
1	1	1	1	1	1	1	3	3	1
1	1	1	1	1	3	3	1	1	1
3	1	1	2		3	3	3	3	3
1	1	1	2	1	3	1	1	1	1
1	1	1	2	1	1	1	1	1	1
1	1	1	2	1	1	1	1	1	1
1	1	1	1	1	1	1	1	1	1
1	1	1	1	1	1	1	2	2	1
1	1	1	1	1	1	1	1	1	1
1	1	1	1	1	1	1	1	1	1
1	1	1	1	1	2	2	2	3	3
1	1	1	1	1	1	1	1	1	1
1	1	1	2	1	1	1	1	1	1
1	1	1	1	1	1	1	1	1	1
1	1	1	1	1	1	1	1	1	1
3	3	3	1	1	2	4			
1	1	1	1	1	3	1	1	1	1
1	1	1	2	1	1	1	2	1	1
1	2	1	3	1	2	2	3	2	3
1	1	1	1	1	3	1	1	2	1
1	1	1	1	1	1	1	1	1	1
1	1	1	3	2	1	1	1	1	1
1	1	1	1	1	2	1	1	1	1
1	2	1	3	1	3	2	1	1	2

1	1	1	1	1	4	4	4	2	1
1	1	1	1	1	1	1	1	1	1
1	1	1	2	1	1	1	1	1	1
1	2	1	4	1	1	1	1	1	1
1	1	2	4	3	1	1	1	1	1
1	1	1	2	1					
2	2	1	2	1	4	4	4	3	1
1	1	1	1	1		1	1	1	1
3	3	1	3	1	1	1	1	1	1
1	1	1	1	1	3	3	3	3	1
1	1	1	1	1	3	3	4	3	1
1	1	1	1	1	1	1	1	1	3
2	1	1	1	1	1	2	1	1	1
1	1	1	2	1	2	2	2	2	1
1	1	1	1	1	1	1	1	1	1
1	1	1	1	2	3	4	3	3	3
1	1	1	1						
3	1	3	3	1	4	4	4	4	4
2	2	2	2	2	3	1	2	1	2
1	1	1	1	1	1	1	3	1	1
1	1	1	4	1	3	1	1	1	1
1	1	1	2	1	1	1	1	1	1
1	1	1	2	1	1	1	1	1	1
1	1	1	1	1	1	1	1	1	1
1	1	2	1	1	1	1	1	1	1
1	1	1	1	1	3	1	1	1	4
1	1	1	2	1	4	4	4	4	4
1	1	1	2	1	3	1	1	1	1
1	1	1	1	1	1	3	3	1	1
1	1	1	1	1	3	3	3	1	1
1	1	1	3	1	3	1	1	1	3
1	1	1	1	1	4	1	4	4	2
1	1	1	1	1	1	1	1	1	1
1	2	1	1	1	3	1	2	1	1
1	1	1	1	1	1	1	1	1	1
1	1	1	2	1	3	1	3	1	3
1	1	1	4	2	3	1	3	1	1
2	1	2	1	1	1	1	3	1	1
2	2	1	1	1	4	4	4	4	4
1	1	1	1	1	1	1	3	1	1
1	1	1	3	1	1	1	1	1	1
3	1	1	3	2	3	1	1	1	1
1	1	1	3	1	2	2	2	2	2
1	1	1	5	1	1	1	1	1	1
1	1	1	1	1	1	1	1	1	1
1	1	1	1	1					
1	1	1	1	1	1	1	1	1	1

1	1	1	1	1	2	1	1	2	1
1	1	1	1	1	2	2	2	2	2
1	1	1	1	1	1	1	1	1	1
1	1	1	2	1					
1	1	1	1	1	4	3	3	3	3
					2	2	2	2	2
2	2	2	2	2	2	2	2	2	2
1	1	1	2	2	4	4	4	4	4
2	2	2	2	2	1	1	1	1	1
2	2	2	2	2	1	1	1	1	1
1	1	1	3	1	3	3	1	3	3
3	1	1	2	1	2	2	2	1	1
1	1	1	2	1	1	1	1	1	1
2	2	2	2	1	1	1	1	1	1
1	1	1	1	1	1	1	1	1	1
1	1	1	3	1	1	1	1	1	1
2	2	2	2	2	1	1	1	1	1
2	2	2	1	2	1	1	1	1	2
1	1	1	1	1	4	1	1	1	1
1	1	1	1	1	1	1	1	1	1
1	1	1	1	1	1	2	2	2	4
1	1	1	1	1	1	1	1	1	1
1	1	1	1	1	3	3	2	1	3
1	1	1	1	1	1	1	1	1	1
1	1	1	1	1	1	1	1	1	1
2	2	2	2	2	1	1	1	1	1
1	1	1	1	1	1	1	1	1	1
1	1	1	1	1	3	3	3	1	1
3	2	1	3	3	1	2	2	3	3
1	1	1	3	1	1	2	2	2	2
1	1	1	1	1	1	1	1	1	1
2	2	2	2	2	4	4	4	4	4
2	2	2	2	2	4	4	4	4	4
2	2	2	2	2	4	4	4	4	4
1	1	1	1	1	1	1	1	1	1
2	1	1	2	1	3	3	1	1	1
1	1	1	1	1	3	3	1	1	1
1	1	1	1	1	3	3	3	3	3
2	2	1	2	2	1	1	1	1	2
1	1	2	1	2	1	1	1	1	1
1	1	1	2	1	4	4	1	1	1
1	1	1	2	1	4	4	4	4	4
1	1	1	1	1	4	1	4	1	1
1	1	1	3	1	4	4	4	4	1
1	1	1	1	1	3	1	1	1	4
1	1	1	1	1	3	1	1	1	4
1	1	1	1	1	4	4	4	4	4

1	1	1	1	1	4	4	4	4	4
1	1	1	1	1	4	1	1	1	4
1	1	1	1	1	4	1	1	1	4
1	1	1	1	1	4	4	4	4	4
1	1	1	1	1	4	4	4	4	4
1	1	1	3	1	4	1	1	1	1
1	1	1	3	1	3	2	4	1	1
3	2	2	1	1	3	1	1	1	1
1	1	1	4	1	1	1	1	1	1
1	1	1	1	1	3	1	1	1	1
1	1	1	3	1	1	1	1	1	1
1	1	1	1	1	4	1	1	1	1
2	1	1	3	1	3	1	1	1	1
1	1	1	2	3	4	3	4	3	4
1	1	1	1	1	1	1	1	1	1
1	1	1	3	1	2	1	1	1	1
1	1	1	2	1	3	1	2	3	1
1	1	1	1	1	3	1	1	1	1
1	1	1	2	1	1	1	1	1	1
2	1	1	3	1	1	1	4	1	1
2	1	1	1	1	1	1	1	1	1
1	1	1	2	1	4	1	4	1	1
1	1	1	1	1	1	1	1	1	1
1	1	1	1	2	1	1	1	1	1
1	1	1	1	1	1	1	1	1	1
1	2	1	2	1	1	1	1	1	1
1	1	1	2	1	4	1	1	1	1
1	1	1	1	1	3	1	1	1	1
1	1	1	1	1	1	1	1	1	1
1	1	1	1	1	1	1	1	1	1
1	1	1	1	1	1	1	1	1	1
1	1	1	1	1	3	3	1	1	1
1	1	1	1	1	1	1	1	1	1
1	1	1	1	1	3	1	1	1	1
1	1	1	2	1	1	1	1	1	1
1	1	1	1	1	1	1	1	1	1
1	1	1	2	1	1	1	1	1	1
1	1	1	1	2	3	3	2	1	1
1	1	1	2	1	4	4	1	1	1
1	1	1	1	1	1	1	1	1	1
1	1	1	1	1	1	1	1	1	1
1	1	1	1	1	1	1	1	1	1
1	1	1	1	1	1	1	1	1	1
1	1	1	1	1	1	1	1	1	1
1	1	1	3	1	1	1	1	1	1
2	1	1	1	3	3	1	1	1	1
1	1	1	1	1	1	1	1	1	1

1	1	1	1	1	1	1	1	1	1
1	1	1	1	1	1	1	1	1	1
1	1	1	1	1	1	1	1	1	1
1	1	1	1	1	3	1	2	1	1
1	1	1	1	1	1	1	1	1	1
1	1	1	1	1	1	1	1	1	1
1	1	1	1	1	4	1	3	2	4
2	1	1	1	1	1	1	1	1	3
1	1	1	1	1	1	1	1	1	1
1	2	2	1	2	3	1	1	1	2
1	1	1	1	1	1	1	1	1	1
1	1	1	2	1	2	1	1	1	1
1	1	1	2	1	3	3	3	1	1
2	2	1	1	1	3	3	1	2	3
1	1	1	1	1	1	1	1	1	1
1	1	1	1	1	1	1	1	1	1
3	1	1	2	1	1	1	1	1	1
3	1	1	2	1	1	1	1	1	1
1	1	1	3	1	1	1	1	1	1
1	1	1	2	1	1	1	1	1	1
1	1	1	1	1	1	1	3	1	1
1	1	1	1	1	1	1	1	1	1
1	2	1	1	1	1	1	1	1	1
1	1	1	2	1	1	1	1	1	1
3	1	1	4	1	4	4	4	4	3
1	1	2	1	2	3	1	1	1	3
2	1	1	1	1	1	1	1	1	1
2	1	1	1	1	1	1	1	1	1
1	1	1	1	1	2	3	2	3	3
1	1	1	4	1	2	1	1	1	1
1	1	1	2	1	1	1	1	1	3
1	1	1	4	2	1	1	1	1	1
1	1	1	1	1	2	1	1	1	1
1	1	1	3	1	2	1	1	1	1
1	1	1	1	2	3	2	3	1	3
1	1	1	2	1	1	1	1	1	1
1	1	1	2	1	1	1	1	1	1
1	1	1	2	1	1	1	1	1	1
1	1	1	1	1	1	1	1	1	1
1	2	1	4	1	2	3	3	1	3
2	2	2	2	1	2	3	2	2	3
1	2	1	4	1	2	3	2	3	2
1	1	1	2	1	2	1	3	1	1
1	1	1	2	1	2	1	3	1	1
1	1	1	1	1	1	1	1	1	1
1	1	1	1	1	4	1	1	1	1
1	1	1	1	1	4	4	1	1	1

1	1	1	1	1	1	1	1	1	1
1	1	1	3	1	4	4	4	4	1
1	1	1	1	1	1	1	1	1	1
1	1	1	2	3	4	4	2	4	1
1	1	1	2	1	4	3	3	3	3
1	1	1	1	1	2	1	1	1	1
1	1	1	3	1	3	1	4	1	1
1	1	1	2	1	3	1	1	1	1
1	1	1	3	1	2	1	3	1	3
1	1	1	1	1	3	1	1	1	1
1	1	1	1	1	1	1	1	1	2
1	1	1	1	1	3	3	2	1	2
1	1	1	2	1	2	2	1	1	1
1	1	1	3	1	3	1	1	1	1
1	1	1	3	1	1	1	1	1	1
1	1	1	2	1	1	1	1	1	1
1	1	1	2	2	3	2	1	1	1
1	1	1	3	1	2	1	1	1	2
1	1	1	3	1					
1	1	1	4	1	1	1	1	1	2
2	1	1	2	1	4	2	1	1	2
1	1	1	3	1	3	1	3	1	1
1	1	1	2	1	1	1	1	2	2
1	2	1	2	1	4	3	4	1	3
1	1	1	3	1	1	1	1	1	1
1	1	1	4	1	1	1	1	1	3
1	1	1	2	2	3	1	1	1	3
1	1	1	1	1	3	2	1	1	1
1	1	1	3	1	3	1	1	1	1
1	1	1	4	1	4	1	1	1	1
1	1	1	1	1	1	1	1	1	1
1	1	1	2	1	4	4	4	4	2
1	1	1	1	1	3	1	1	1	1
1	1	1	1	1	1	1	1	1	1
1	1	1	3	1	3	1	1	1	1
1	1	1	1	1	1	1	1	1	1
1	1	1	3	1	1	1	1	3	1
1	1	1	1	1	3	2	1	1	1
1	1	1	3	1	4	2	3	1	1
1	1	1	1	1	1	1	1	1	1
1	1	1	1	1	4	4	4	1	4
1	1	1	1	1	4	4	4	4	1
1	1	1	3	1	1	1	1	1	1
1	1	1	3	1	1	1	1	1	1
1	1	1	3	1	1	1	1	1	1
3	1	1	2	1					
1	1	1	3	1	1	1	1	1	1

1	1	1	3	1	2	2	1	1	2
1	1	1	1	1	1	1	1	1	1
1	1	1	1	1	3	1	3	1	1
2	4	2	3	2	2	3	4	3	2
1	1	1	1	1	1	1	1	1	1
2	1	1	1	1	3	1	1	1	1
1	1	1	1	1	1	1	1	1	1
2	1	3	1	2					
2	1	1	1	1	3	1	1	1	1
2	1	1	1	1	1	1	3	3	1
1	1	1	1	1	3	1	1	1	1
1	1	1	1	1	1	1	1	1	1
1	1	1	1	1	2	1	1	1	1
2	1	1	1	1	1	1	4	1	1
1	1	1	1	1	1	1	1	1	1
1	1	1	1	1	1	1	1	1	1
1	1	1	1	1	1	1	1	1	1
1	1	1	1	1	1	1	1	1	1
1	1	1	1	1	1	1	1	1	1
2	1	1	2	1	1	1	1	1	1
1	1	1	1	1					
1	1	1	1	1	1	1	1	1	1
1	1	1	1	1	1	1	1	1	1
1	1	1	1	1	1	1	2	1	1
1	1	1	1	1	2	1	1	1	1
1	1	1	1	1	1	1	1	1	1
2	1	1	3	1	3	3	3	3	3
1	1	1	2	1	1	1	1	1	1
3	1	1	2	1	1	1	1	1	1
1	1	1	1	1	2	3	2	1	1
1	1	1	3	1	3	1	3	1	1
1	1	1	2	1	1	1	1	1	1
1	1	1	2	1	4	4	4	4	1
1	1	1	3	1	1	3	3	4	3
1	1	2	2	2					
1	1	1	1	1	3	1	1	1	1
3	1	1	1	1					
1	1	1	3	1	3	3	3	4	3
1	1	1	2	1	1	1	1	1	1
1	1	1	1	1	4	1	1	1	1
1	1	1	1	1	1	1	1	1	1
1	1	1	1	1					
1	1	1	1	1	3	1	4	1	1
1	1	1	1	1	3	1	1	1	1
1	1	1	1	1	1	1	1	1	1
1	1	1	1	1	4	1	3	3	3
1	1	1	2	1	2	1	1	1	2

1	1	1	1	1	3	1	1	1	1
1	1	1	1	1	1	1	1	1	1
1	1	1	2	2	1	1	1	1	1
1	1	1	1	1	1	1	1	1	1
1	2	1	3	1	4	4	1	1	1
3	1	1	2	2	1	3	1	3	3
1	1	1	1	1	2	1	1	1	1
1	1	1	1	1	3	1	3	1	1
1	1	1	1	1	1	1	1	1	1
3	1	1	2	1	4	1	4	1	1
1	1	1	1	1	1	1	1	1	1
1	1	1	1	1	1	1	1	1	1
1	1	1	3	1	4	1	1	1	1
1	1	1	1	1	1	1	1	1	1
1	1	1	2	1	4	1	4	1	1
1	1	1	1	1	1	1	1	1	1
1	1	1	1	1	4	1	4	1	1
1	1	1	1	1	1	1	1	1	1
1	1	1	2	1	1	1	1	1	1
1	1	1	4	1	1	1	1	1	1
1	1	1	1	1	1	1	1	1	1
1	1	1	1	1	4	1	4	1	1
1	1	1	1	1	4	4	4	1	1
1	1	1	1	1	3	1	1	1	1
1	1	1	2	1	1	1	1	1	1
1	1	1	2	1	2	1	1	1	1
1	1	1	2	1	1	1	1	1	1
1	1	1	1	1	1	1	1	1	1
1	1	1	1	1	1	1	1	1	1
1	1	1	2	2	3	1	3	3	1
1	1	1	1	1	4	1	1	1	1
1	1	1	2	1	1	1	1	1	1
1	1	1	2	1	1	1	1	1	1
1	1	1	1	1	1	1	1	1	1
1	1	1	1	1	1	1	1	1	1
1	1	1	1	1	1	1	1	1	1
1	1	1	2	1	4	1	1	1	1
1	1	1	1	1	1	1	1	1	1
2	1	1	2	1	1	1	1	1	1
1	1	1	2	1	1	1	1	1	1
1	1	1	1	1	1	1	1	1	1
1	1	1	2	1	3	1	3	1	1
2	1	1	1	1	2	1	1	1	3
1	2	1	3	1	2	2	1	2	2
1	1	1	3	1	1	1	1	1	1
1	2	3	2	4	1	2	1	1	3
1	1	1	2	1	3	2	1	1	1

1	1	1	1	1	3	4	3	1	1
2	1	1	2	3	4	4	4	1	4
1	1	1	1	1	1	1	1	1	1
1	1	1	1	1	1	1	1	1	1
1	1	1	1	1	3	3	4	3	1
1	3	1	3	2	4	4	4	4	4
1	1	1	1	3	1	1	1	1	1
1	1	1	1	3	1	1	1	1	1
1	2	1	2	1	2	1	1	2	1
1	2	3	3	2	2	1	1	2	1
1	1	1	1	1	2	2	1	1	2
1	1	1	2	1	4	4	4	4	4
1	2	1	2	2	2	3	3	3	1
1	1	2	2	2	4	4	4	4	4
1	2	1	2	1	1	1	1	1	1
2	1	1	1	1	3	3	1	1	1
1	1	1	1	1	2	2	2	2	2
1	1	1	4	1	1	1	1	1	1
1	1	1	4	1	1	1	1	1	1
1	1	1	3	1	1	1	1	1	1
1	1	1	2	1	1	1	1	1	1
1	1	1	1	1	2	1	2	1	1
1	1	1	2	1	3	3	3	2	3
1	1	1	1	1	1	1	1	1	1
1	1	1	4	1	1	1	1	1	1
2	2	2	2	2	1	1	1	1	1
1	1	1	2	1	1	1	1	1	1
1	1	1	1	1	2	3	2	3	3
2	2	2	2	2	1	1	1	1	1
1	2	1	2	1	2	2	1	1	1
1	1	1	1	1	4	4	4	4	4
2	2	3	4	1	4	1	1	3	1
1	1	1	1	1	1	1	1	1	1
1	1	1	1	1	4	4	4	4	4
2	2	2	2	2	3	3	3	2	3
1	1	1	2	1	1	2	2	1	3
1	1	1	3	1	3	3	1	1	3
1	1	1	2	1	1	1	1	1	1
1	1	1	1	1	1	1	1	1	1
2	2	2	2	2	1	1	1	1	1
4	4	4	4	4	1	1	1	1	1
3	3	3	3	3	1	1	1	1	1
2	1	2	1	2	2	2	2	2	2
2	2	2	2	2	4	4	4	4	4
1	1	1	2	1	4	4	1	1	1
1	1	1	2	1	4	3	3	3	3
1	1	1	2	1	3	3	3	3	3

1	1	1	1	1	1	1	1	1	1
1	1	1	1	1	4	4	4	4	4
1	1	1	1	1	4	2	1	1	4
1	1	1	1	1	4	1	1	1	1
1	1	1	4	1	4	4	3	1	1
1	1	1	1	1	1	1	1	1	3
1	1	1	1	1	3	1	3	1	1
1	1	1	1	1	3	1	3	1	1
1	1	1	2	1	4	1	3	1	1
1	1	1	4	1	4	1	3	1	3
2	1	1	1	1	3	1	1	1	1
1	1	1	3	1	1	1	1	1	1
1	1	1	3	1	4	4	4	2	4
1	1	1	1	1	1	1	1	1	1
1	1	1	1	1	1	1	1	1	1
1	1	1	1	1	1	1	1	1	1
1	1	1	1	1	1	1	1	1	1
1	1	1	1	1	3	1	1	1	1
1	1	1	1	1	1	1	1	1	1
1	1	1	1	1	3	1	1	1	1
1	1	1	1	1	3	1	3	1	1
1	1	1	1	1	1	1	3	1	1
1	1	1	2	1	1	1	1	1	1
2	1	1	2	1	3	2	2	1	3
1	1	1	1	1	1	1	1	1	1
2	1	1	1	1	3	3	3	3	1
1	1	1	5	1	2	2	1	1	1
1	1	1	2	1	3	1	1	1	1
1	1	1	1	1	1	1	1	1	1
1	1	1	1	1	4	4	1	3	4
1	1	1	1	1	1	1	1	1	1
1	1	1	1	1	3	1	1	1	1
1	1	1	1	1	4	1	1	1	1
1	1	1	1	1	3	1	1	1	1
1	1	1	1	1	1	1	1	1	1
1	1	1	1	1	1	1	1	1	1
1	1	1	1	1	3	3	1	3	1
1	1	1	1	1	1	1	1	1	1
1	1	1	1	1	3	3	1	1	1
1	1	1	1	1	3	3	1	1	1
1	1	1	1	1	1	1	1	1	1
1	1	1	1	1	1	1	1	1	1
1	1	1	2	1	1	1	1	1	1
1	1	1	1	1	4	3	4	4	3
1	2	1	2	1	3	1	1	1	1
2	2	1	3	1	4	1	3	1	2
2	2	1	3	2	1	1	1	1	1

2	1	1	5	1	2	1	3	1	2
1	1	1	1	1	1	1	1	1	1
2	1	1	2	1	2	1	4	2	1
1	1	1	2	1	1	1	1	1	1
1	1	1	2	1	1	1	1	1	1
1	1	1	4	1	1	1	1	3	3
2	2	2	2	1	1	1	1	1	1
1	1	1	1	1	1	1	1	1	1
1	1	1	3	1	3	1	3	1	1
1	1	1	1	1	1	1	1	1	1
1	1	1	3	1	1	1	1	1	1
1	1	1	2	2	3	1	1	1	1
1	1	1	1	1	1	2	2	1	2
1	1	1	1	1	1	1	1	1	3
1	1	1	1	1	1	1	1	1	1
1	1	1	2	1	1	1	1	1	1
1	1	1	3	1	1	1	1	1	1
1	1	1	2	1	1	1	1	1	1
1	1	3	3	1	1	1	1	1	1
3	1	1	3	1	4	1	4	1	1
1	1	1	3	1	3	1	1	1	1
1	1	1	1	1	4	1	2	1	3
1	1	1	2	1	3	1	1	1	1
2	1	1	1	1	3	1	1	1	1
3	2	1	5	3	1	1	1	1	1
1	1	1	3	1	1	1	1	1	1
1	1	1	3	1	3	1	1	1	1
1	1	1	5	1	1	1	1	1	2
1	1	1	1	1	1	1	1	1	1
1	1	1	3	1	1	1	1	1	1
1	1	1	2	1	1	1	1	1	1
1	1	1	1	2	1	1	1	1	1
1	1	1	3	1	1	1	1	1	1
1	1	1	3	2	2	1	1	1	2
1	1	1	1	1	3	1	1	1	1
1	1	1	2	1	1	1	1	1	1
1	1	1	4	1	1	1	1	1	1
1	1	1	4	1	2	1	1	1	1
1	1	1	1	1	2	2	2	1	1
1	1	1	3	1	4	1	1	1	1
2	1	1	1	1	3	3	1	3	3
3	1	1	2	1	4	2	3	4	3
1	1	1	2	1	1	1	1	1	1
1	1	1	1	1	1	1	1	1	1
1	1	1	3	1	1	1	1	1	1
1	1	1	3	1	3	1	1	1	1
1	1	1	2	1	3	1	1	1	1

1	1	1	3	1	1	1	1	1	1
1	2	2	3	3	1	1	1	1	1
2	1	1	1	1	3	1	1	1	1
1	1	1	3	1	3	1	1	1	1
2	1	1	3	1	1	1	1	1	1
3	1	1	2	1	1	1	3	1	1
1	1	1	1	1	1	1	1	1	1
2	1	1	2	1	3	1	1	1	1
1	1	1	1	1	1	1	1	1	1
1	1	1	1	1	3	1	1	1	1
1	1	1	1	1	2	1	1	1	1
1	1	1	1	1	1	1	1	1	1
1	1	1	1	1	1	1	1	1	1
1	1	1	1	1	1	1	1	1	1
1	1	1	1	1	2	1	1	1	1
1	1	1	1	1	1	1	1	1	1
3	1	1	1	1					
1	1	1	1	1	1	1	1	1	1
1	1	1	1	1					
1	1	1	3	1	1	1	1	1	1
1	1	1	1	1	2	1	1	1	4
2	1	1	3	2	1	1	4	3	1
3	1	1	4	4	1	1	1	1	1
2	1	1	2	1	3	1	2	1	1
1	1	1	3	1	1	1	1	1	1
1	1	1	2	1	1	1	2	1	1
1	1	1	4	1	1	1	1	1	1
3	1	1		1	4	4	4	4	2
1	1	1	2	1	1	1	1	1	1
1	1	2	4	2	1	1	3	4	1
1	1	1	3	1	1	2	1	1	1
1	1	1	1	1					
1	1	1		1	3	3	3	1	4
3	3	2	4	3	2	2	1	1	4
1	1	1	1	1	1	1	1	1	1
1	1	1	3	1	4	4	4	1	4
1	1	1	1	1	1	1	1	1	1
1	1	1	1	1	4	1	1	1	1
1	1	1	1	1	3	2	2	1	1
2	1	1	1	1	2	2	1	1	1
1	1	1	2	1	1	1	1	1	1
1	1	1	1	1	1	1	1	1	1
1	1	1	1	1	1	1	1	1	1
1	1	1	1	1	1	1	1	1	1
1	1	1		1	1	1	1	1	1
1	2	1	2	1	1	4	4	1	4
1	1	1	2	1	3	1	1	1	1

1	1	1	1	1	1	1	1	1	1
1	1	1	1	1	1	3	3	1	1
1	1	1	1	1	1	1	1	1	1
1	1	1	1	2	3	3	3	3	1
1	1	1	2	1	3	3	1	1	1
1	1	1	2	1	4	1	4	1	1
1	2	1	1	1	1	1	1	1	1
2	1	1	3	1	1	1	1	1	1
1	1	1	1	1	1	1	1	1	1
1	1	1	1	1	4	1	1	1	1
1	1	1	1	1	3	1	1	1	1
1	1	1	1	1	1	1	1	1	1
1	1	1	1	1	1	1	1	1	1
1	1	1	1	1	4	4	4	4	1
1	1	1	1	1	1	1	1	1	1
1	1	1	3	1	4	1	1	1	4
1	1	1	1	1	4	1	1	1	1
1	1	1	1	1	4	4	4	4	4
1	1	1	1	1	1	1	1	1	1
1	1	1	1	1	1	1	1	1	1
1	1	2	1	2	3	3	3	3	1
2	2	1	1	1					
1	1	1	2	3	1	1	1	1	1
1	1	1	1	1					
1	1	1	1	1	1	1	1	1	1
1	1	1	2	1	3	3	3	3	4
1	1	1	1	1	1	1	1	1	1
1	1	1	2	1	4	4	4	3	4
2	1	1	3	1	3	1	4	4	1
1	1	1	1	1	3	4	4	4	4
1	1	1	1	1	1	1	1	1	1
1	1	1	1	1	1	1	1	1	1
1	1	1	1	1	3	3	1	1	1
1	1	1	1	1	3	1	1	1	1
2	1	3	1	3	1	1	1	1	1
3	1	1	1	1	4	4	4	2	4
1	1	1	1	1	1	1	1	1	1
1	1	1	2	1	3	3	3	3	3
1	1	1	2	1	3	3	3	3	3
1	1	1	3	1	1	1	1	1	1
1	2	4	3	3	3	3	1	1	1
1	1	1	1	1	1	1	1	1	1
1	1	1	1	1	3	4	3	3	3
1	1	3	2	3	1	1	1	1	1
2	3	1	1	1	3	1	1	1	1
1	1	1	1	1	1	1	1	1	1
1	1	1	3	3	2	2	2	2	2

1	1	1	3	1	1	1	1	1	3
1	1	1	3	1	1	1	1	1	1
1	1	1	3	1	1	1	1	1	1
1	1	1	1	1	1	1	1	1	1
1	1	1	1	1	4	4	1	4	1
1	1	1	3	1	3	3	3	3	1
1	1	1	3	1	1	1	1	1	1
2	1	1	1	1	3	3	3	3	3
1	1	1	1	2	1	1	1	1	1
1	1	1	3	1	1	1	1	3	1
1	1	1	1	1	1	1	1	1	1
1	1	1	1	2	1	1	1	1	1
1	1	2	2	2	2	3	2	2	2
1	1	1	2	1	1	1	3	1	1
1	1	1	2	1	3	3	1	1	3
1	1	1	1	1	2	2	2	2	1
3	3	3	3	3	3	3	3	3	3
1	1	1	2	1	1	1	1	1	1
1	2	1	1	1	4	4	4	4	4
1	2	1	1	1	4	4	4	4	4
1	1	1	1	1	1	1	1	1	2
1	1	1	1	1	1	1	1	1	1
1	1	1	1	1	3	3	1	1	1
1	1	1	1	1	1	1	1	1	1
1	1	1	1	1	1	1	1	1	1
1	1	1	3	1	4	1	3	1	1
3	1	2	1	2	1	2	4	4	1
1	1	1	1	1	4	1	4	4	1
1	1	1	1	1	4	1	1	3	1
3	1	1	1	1	4	1	1	1	1
1	1	1	1	1	1	1	1	1	1
1	1	1	3	1	4	1	4	3	3
1	1	1	1	1	4	1	4	1	1
1	1	1	3	1	4	3	1	1	1
1	2	1	2	1	4	1	1	1	1
1	1	1	1	1	1	1	1	1	1
1	1	1	1	1	4	1	3	1	1
1	1	1	2	1	1	1	1	1	1
1	1	1	1	1	3	1	1	1	1
1	1	1	5	1	4	4	1	1	1
1	1	1	1	1	1	1	1	1	1
1	1	1	1	1	3	1	3	3	1
1	1	1	1	1	1	1	1	1	1
1	1	1	1	1	1	1	1	1	1
2	1	1	1	1	1	1	1	1	1
1	1	1	1	1	1	1	1	1	1
1	1	1	1	1	1	1	1	1	1

1	1	1	1	1	3	1	1	1	1
1	1	1	1	1	1	1	1	1	1
1	1	1	1	1	1	1	1	1	1
1	1	1	1	1	4	1	1	1	1
1	1	1	1	1	4	4	1	1	4
1	1	1	1	1	1	1	1	1	1
2	1	1	1	1	1	1	1	1	1
1	1	1	1	1	3	1	1	1	1
1	1	1	2	1	1	1	1	1	1
1	1	1	1	1	3	1	1	1	1
1	1	1	1	1	3	1	1	1	1
1	1	1	2	1	3	1	1	1	1
1	1	1	2	1	3	1	1	1	1
1	1	2	3	2	3	1	3	1	1
1	1	1	5	3	1	1	4	1	2
1	1	1	1	1	1	1	1	1	1
1	1	1	3	1	4	4	4	4	1
1	1	1	1	1	4	4	4	4	4
1	1	1	2	1	2	1	1	1	1
1	1	1	1	1	4	4	4	1	1
1	1	1	1	1	1	1	1	1	4
1	1	1	2	1	1	1	1	1	1
1	1	1	1	1	1	1	3	1	1
1	1	1	2	1	1	1	1	1	1
1	1	1	2	1	1	1	1	1	1
1	1	1	5	1	3	1	1	1	1
1	1	1	2	1	1	1	1	1	1
1	1	1	1	1	3	1	1	1	1
2	1	1	2	1	4	4	4	3	3
1	1	1	4	1	3	1	1	1	1
1	1	1	4	1	1	1	1	1	1
1	1	1	4	1	3	2	3	1	2
1	1	1	1	1	3	1	1	1	1
1	1	1	2	1	1	1	1	1	1
1	1	1	2	1	3	1	1	1	1
1	1	3	1	2	1	1	1	3	3
1	1	1	2	1	1	1	1	1	1
1	1	1	1	1	1	1	1	1	1
1	1	1	3	1	1	1	1	3	3
1	1	1	1	1	4	4	1	1	1
1	1	1	2	1	2	1	1	1	1
1	1	1	4	1	2	3	1	1	3
1	1	1	1	1					
2	1	1	3	1	3	3	3	1	3
1	1	1	3	1	1	1	1	1	1
1	1	1	1	1	3	1	1	1	1
1	1	1	2	1	3	1	4	1	1

1	1	1	4	1	1	1	1	1	1
1	1	2	3	2	2	1	1	1	2
1	1	1	1	1	3	3	3	3	1
1	1	3	1	3	1	1	1	1	1
1	1	1	3	1	4	3	4	1	3
1	1	1	1	2	4	3	1	3	3
3	1	1	1	1	3	1	1	3	3
1	1	1	1	1	1	1	1	3	3
1	1	1	1	2	4	3	1	3	3
1	1	1	1	1					
1	1	1	1	1	1	1	1	1	1
1	1	1	1	1	1	1	1	1	1
1	1	1	1	1	1	1	1	3	3
1	1	1	1	1	1	1	1	1	1
1	1	1	1	1					
1	1	1	1	1	1	1	1	1	1
1	1	1	1	2	4	1	1	1	1
1	1	1	1	1	1	1	1	1	1
1	1	1	1	1	1	1	1	1	1
2	1	1	3	1					
2	1	1	1	1					
1	1	1	1	1	1	1			1
2	1	1	1	1	1	1	1	1	1
1	1	1	4	1			1	1	1
3	1	3	4	3	4	1	3	4	4
1	1	1	4	1	1	1	1	1	1
1	2	2	2	2	1	1	2	3	1
1	1	1	2	1					
3	1	1		3	1	1	3	1	4
1	1	1	2	1	1	1	1	1	1
1	1	1	3	1	1	1	1	1	1
1	1	4	4	3	3	3	4	4	2
1	2	1	3	1	1	1	1	1	1
1	1	1	4	1	3	1	3	1	1
1	1	1	2	1	1	1	1	1	1
1	1	1	2	1	1	1	1	1	1
1	1	1	2	1	1	2	1	1	1
1	1	1	1	1	1	1	1	1	1
1	1	1	2	1					
1	1	1	1	1	4	1	1	1	1
1	1	1	1	1	1	1	1	1	1
1	1	1	3	1	1	1	1	1	1
1	1	1	1	3	2	1	3	1	1
3	1	1	1	1	4	4	4	4	4
1	1	1	1	1	1	1	1	1	1
1	1	1	1	1	2	1	1	1	1
1	1	1	1	1	2	1	1	1	1

1	1	1	1	1	3	1	3	1	1
1	1	1	1	1					
1	1	1	1	1	1	1	1	1	1
1	1	1	2	1	1	1	1	1	1
1	1	1	2	1	3	1	3	1	1
1	1	1	3	1	3	4	3	1	1
1	1	1	1	1	3	1	1	1	1
1	1	1	1	1	1	1	1	1	1
1	1	2	2	1	4	4	4	1	1
1	1	1	1	1	4	1	4	4	1
1	1	1	1	1	1	1	1	1	1
1	1	1	1	1	3	1	2	1	1
1	1	1	1	1	1	1	1	1	1
1	1	1	1	1	3	1	1	1	1
1	1	1	2	1	1	1	1	1	1
1	1	1	1	1	1	1	1	1	1
1	1	1	1	1	1	1	1	1	1
1	1	1	1	1					
1	1	1	2	1	2	1	1	1	1
1	1	1	1	1	1	1	1	1	1
1	1	1	1	1	4	1	1	1	3
1	1	1	5	1	1	1	1	1	1
1	1	1	1	1	1	1	1	1	1
1	1	1	1	1	1	1	2	1	1
4	1	1	1	1	1	1	1	1	1
1	1	1	1	1	1	1	3	1	1
1	2	1	3	1	3	1	1	3	1
1	1	1	2	1	1	3	1	1	1
1	1	2	2	2	1	1	1	1	1
1	1	1	1	1	1	1	1	1	1
1	2	1	3	3	1	1	1	1	1
1	1	1	2	1	4	4	4	4	4
1	1	1	2	1	1	1	1	1	1
1	1	1	2	1	1	1	1	1	1
1	1	1	3	2	1	1	1	1	1
1	1	1	3	2	1	1	1	1	1
2	1	3		1	1	1	1	1	1
1	1	1	1	1	1	1	1	1	1
1	1	2	1	1	2	3	2	2	3
2	1	1	1	1	4	4	4	1	1
1	1	1	1	1	4	4	4	4	1
1	1	1	2	1	3	3	1	1	2
2	1	1	2	1	1	1	1	1	1
1	1	1	1	1	3	3	3	1	1
1	1	1	1	1	1	1	1	1	1
1	1	3	3	2	1	1	1	1	1
1	1	1	1	2	1	1	1	1	1

1	1	1	1	1					
1	1	1	2	2	1	1	1	1	1
1	1	1	1	1	1	1	1	1	1
1	1	1	1	1	1	1	1	1	1
1	1	1	1	1	3	3	1	1	1
1	1	1	1	1	1	1	1	1	1
1	1	1	1	1	4	4	3	3	2
1	1	1	4	3					
1	1	1	1	1	3	3	3	3	3
1	1	1	1	1	1	1	1	1	1
1	2	1		1	4	3	3	1	1
1	1	1	2	1	3	1	3	1	1
1	1	1	2	1	1	2	2	1	1
1	1	1	1	1	2	1	1	1	1
1	1	1	3	1	4	3	3	3	1
1	1	1	2	1	1	1	1	1	1
2	1	1	1	1	4	3	1	1	1
2	1	1	1	1	4	3	1	1	1
1	1	1	1	1	4	1	4	4	3
3	3	3	1	3	4	4	4	3	4
1	1	1	1	1	4	1	1	1	1
1	1	1	2	1	3	1	1	1	1
1	1	1	1	1	3	1	1	1	1
1	1	1	1	1	4	1	4	3	1
1	1	1	2	1	1	1	1	1	1
1	1	1	1	1	3	1	1	1	1
1	1	1	1	1	3	1	1	1	1
1	1	1	1	1	2	1	1	1	1
1	1	1		1	1	1	1	1	1
1	1	1	1	1	3	4	4	4	3
1	1	1	5	1	4	4	4	4	1
2	1	1	3	1	3	1	4	4	1
1	1	2	1	1	4	4	1	2	1
1	1	1	5	1	3	1	1	1	1
1	1	1	1	1	3	3	3	3	3
1	1	1	1	1	3	1	1	1	1
1	1	2	1	1	4	4	4	1	1
1	1	1	1	1	1	1	1	1	1
1	1	1		1	1	2	1	3	1
1	1	1	1	1	4	4	3	1	3
1	1	1	3	1	4	4	1	1	4
1	1	1	2	1	3	3	2	2	3
1	1	1	1	1	3	3	1	1	1
1	1	1	1	1	1	1	1	1	1
1	1	1	1	1	1	1	1	1	1
1	1	1	1	1	1	1	1	1	1
1	1	1	1	1	3	1	1	1	3

1	1	1	1	1	3	1	4	1	1
1	1	1	1	1	1	1	1	1	1
1	1	1	1	1	2	1	1	1	1
1	1	1	5	1	4	1	4	1	1
1	1	1	1	1	3	1	1	1	1
1	1	1	3	3	1	1	1	1	1
1	1	1	2	1	2	1	1	1	1
1	1	1	5	1	4	3	3	3	1
4	1	1	1	1	1	1	1	1	1
1	1	1	1	1	1	1	1	1	1
1	2	1	3	1	2	2	3	1	1
1	1	1	1	1	1	1	3	1	1
1	1	1	1	1	1	1	3	1	1
1	1	1	2	1	1	1	1	1	1
1	1	1	1	1	1	1	1	1	1
1	1	1	1	1	1	1	1	1	1
1	1	1	4	3	4	3	4	1	4
2	1	1	5	1	1	1	3	1	1
1	1	1	2	1	1	1	1	1	1
1	1	1	2	1	1	1	1	1	1
1		1	1	1	1	1	1	1	1
1	1	1	1	1	3	1	1	1	1
1	1	1	3	1	3	2	1	1	1
1	1	1	1	1	1	1	1	1	1
1	1	1	2	1	1	1	1	1	2
1	2	1	1	1	3	1	1	1	1
1	1	1	1	1	1	1	1	1	1
1	1	1	2	1	1	1	1	1	1
1	1	1	4	1	1	2	1	1	1
1	1	1	2	1	1	1	1	1	2
1	1	1	2	1	3	2	3	3	1
1	1	1	2	1	1	1	1	1	1
1	1	1	2	1	3	1	1	3	1
1	1	1	1	1					
1	1	1	1	1	4	1	4	4	3
1	1	1	1	1	3	1	3	3	1
1	1	1	1	1					
1	1	1	1	1	1	1	1	1	1
1	1	1	1	1	1	1	1	1	1
1	1	1	2	1	3	1	3	1	1
2	1	1	2	1					
1	1	1	1	1					
1	1	1	1	1					
2	1	1	2	1	1	1	1	1	1
1	1	1	1	1	1	1	1	1	1
1	1	1	1	1	1	1	1	1	1
1	1	1	2	1	1	1	2	1	1

1	3	1	3	1	4	4	3	3	4
1	1	1	4	1	3	1	3	3	1
3	1	2		1	4	1	3	3	2
1	1	1	1	1	1	1	1	1	1
1	3	1	2	1					
1	1	1	2	1					
1	1	1	4	2	1	1	4	1	4
1	1	1	1	1	3	1	1	1	1
1	1	1	2	1	4	1	1	1	1
1	1	1	1	1	1	1	1	1	1
1	1	1	1	1	4	1	1	1	1
	2	1	1		3	3	1	3	1
1	1	1	1	2					
1	1	1	1	1	1	1	1	1	1
1	1	1	1	2	4	1	1	1	1
1	1	1	1	1	3	1	1	1	1
1	1	1	1	1	1	1	1	1	1
1	1	1	1	1	4	1	4	1	1
1	1	1	3	1	4	1	1	4	1
1	1	1	1	1	4	3	1	1	1
1	1	1	2	1	3	1	1	1	1
1	1	1	2	1	3	1	1	1	1
1	1	1	1	1	1	1	1	1	1
1	1	1	1	1	4	4	1	1	1
1	1	1	1	1	4	4	1	1	1
1	1	1	1	1	3	1	1	1	1
1	1	1	1	1	1	1	1	1	1
1	1	1	1	1	1	1	1	1	1
1	1	1	1	1	4	1	4	3	1
1	1	1	1	1	4	1	1	1	1
1	1	1	1	1	4	1	1	1	1
1	1	1	1	1	1	1	1	1	1
1	1	1	1	1	3	1	3	1	3
1	1	1	2	1	2	1	3	1	1
1	1	1	2	1	2	1	3	1	1
1	1	1	2	1	3	1	3	1	3
1	2	1		1	4	1	1	1	1
1	1	1	1	1	1	1	1	1	1
1	1	1	2	1	4	1	3	1	1
3	1	1	1	1	1	1	1	1	1
1	1	1	1	1	1	1	1	1	1
1	1	1	2	1					
1	1	1	1	1	1	1	1	1	1
1	1	1	1	1		1	1	1	1
1	1	1	1	1	3	3	2	2	1
1	1	1	1	1					
1	1	1	2	1	1	1	2	4	4

1	1	1	1	1	4	4	4	4	1
1	1	1	1	1					
1	1	1	2	1	3	3	1	1	1
2	2	2	2	2					
1	1	1	1	1					
1	1	1	3	1	3	1	3	1	1
1	1	1	2	1					
1	1	1	1	1					
1	1	1	2	2	3	3	3	3	3
1	1	1	1	1	1	1	1	1	1
1	1	1	1	1	1	4	4	4	4
1	1	1	2	1	1	1	1	1	1
1	1	1	1	1	3	3	3	3	1
1	1	1	1	1	1	1	1	1	1
1	1	1	3	1	3	1	2	1	1
2	2	1	1	2	3	3	3	3	2
2	1	1	3	1	3	2	2	2	2
1	1	1	4	1	2	3	3	3	3
1	1	1	3	1	4	4	4	4	4
1	2	2	2	1	4	2	4	1	3
1	1	1	1	1	3	2	3	1	1
1	1	1	2	1	1	1	1	1	1
1	1	1	1	1	2	2	2	2	2
1	1	1	1	1	3	3	3	1	1
1	1	1	1		1	1	1	1	1
1	1	1	1	1	1	1	1	1	1
1	1	1	1	1	1	1	1	1	1
1	1	1	3	1	1	1	1	1	1
1	1	1	1	1	3	3	3	3	1
1	1	1	1	1	1	1	1	1	1
1	1	1	2	1	1	1	1	1	1
1	1	1	1	1	3	3	3	1	1
1	1	1	1	1	1	1	1	1	1
1	1	1	1	3					
1	1	1	1	1	1	1	1	1	1
1	1	1	1	1	1	1	1	1	1
1	1	1	1	1	1	1	1	1	1
2	2	2	2	2	1	1	2	2	2
2	2	2	2	2	1	1	1	1	1
1	1	1	1	2	1	1	1	1	1
1	1	1	1	1	1	1	1	1	1
1	1	1	2	1	1	1	1	1	1
1	1	1	1	1	1	1	1	2	1
1	1	1	1	1	2	2	2	2	2
			1	1	4	4	4	3	3
1	1	1	1	1	1	1	1	1	1
1	1	1	1	1	1	1	1	1	1

1	1	1	1	1	1	1	1	1	1
1	1	1	1	1	1	1	1	1	1
1	1	1	1	1	1	1	1	1	1
1	2	1	2	1	1	1	1	1	1
2	2	2	2	2	1	1	1	1	1
1	1	1	1	1	1	1	1	1	1
1	1	1	1	1	1	2	2	2	2
2	2	2	2	2	1	1	1	1	1
3	3	3	4	1					
3	1	3	3	4	1	1	1	1	1
2	2	2	2	2	1	1	1	2	2
2	1	1	1	1	3	1	1	1	1
3	2	2	3	2	2	2	2	3	1
1	1	1	1	1	4	4	4	4	4
2	2	1	2	1					
1	1	1	3	1	4	2	2	1	1
1	5	1	3	3	4	4	3	3	1
1	1	1	1	1	4	4	4	4	4
1	1	1	1	1	3	3	3	1	1
1	1	1	2	1	4	4	4	4	4
1	1	1	3	1	4	3	1	1	1
1	1	1	3	1	3	3	1	1	1
2	2	1	1	1	4	4	4	4	4
2	2	1	1	1	4	4	4	4	4
2	2	1	1	1	4	1	1	4	1
1	2	1	2	1	4	4	4	4	4
1	1	1	2	1	4	4	4	4	4
1	1	1	3	1	4	1	3	1	1
1	1	1	4	1	4	1	1	1	1
1	1	1	1	1	3	1	1	1	1
1	1	1	5	1	4	4	4	1	1
1	1	1	1	1	4	1	4	4	1
1	1	1		1	4	1	4	4	4
1	1	1	2	1	4	1	1	1	1
1	1	1	3	1	3	1	1	1	1
1	1	1	1	1	4	4	4	4	4
1	1	1	1	1	4	4	4	4	4
1	1	1	1	1	4	1	1	1	1
1	1	1	1	1	4	1	1	1	1
1	1	1	5	1	4	1	1	1	2
1	1	1	5	1	1	1	1	1	1
1	1	1	1	1	4	1	1	1	1
1	1	1	2	1	4	4	4	4	4
1	1	1	2	1	3	1	1	1	1
1	1	1	2	1	3	1	1	1	1
1	1	1	1	1	4	4	4	4	4
1	1	1	1	1	4	4	4	4	4

1	1	1	1	1	3	1	1	1	1
1	1	1	1	1	4	1	1	1	1
1	1	1	1	1	3	1	1	1	1
1	1	1	2	1	3	2	3	3	2
2	1	1	1	2	1	2	1	2	1
1	1	1	5	1	1	1	1	1	1
1	1	1	1	1	1	1	1	1	1
1	1	1	1	1	3	1	1	1	1
1	1	1	1	1	4	1	1	1	1
1	1	1	1	1	3	3	3	3	1
1	1	1	1	1	1	1	1	1	1
1	2	1	1	1	4	1	1	1	1
2	1	1	1	1	1	1	1	1	1
1	1	1	1	1	3	3	1	3	1
1	1	1	1	1	3	1	1	1	1
1	1	1	1	1	4	4	4	4	4
1	1	1	1	1	2	1	1	1	1
1	1	1		1	1	1	1	1	1
1	1	1	5	1	1	1	1	1	1
1	1	1		1	1	1	1	1	1
1	1	1	1	1	3	1	1	1	1
1	1	1	1	1	1	1	1	1	1
1	1	1	1	1	4	1	1	1	1
1	1	1	1	2	4	4	4	4	4
1	1	1	1	1	1	1	1	1	1
1	1	1	1	1	4	3	1	1	1
1	1	1	1	1	1	1	1	1	1
1	1	1	1	1	3	1	1	1	1
1	1	1	1	1	3	1	1	1	1
1	1	1	1	1	3	1	1	1	1
1	1	1	1	1	1	1	1	1	1
1	1	1	1	1	4	1	1	1	1
2	1	1	5	1	1	1	1	1	1
1	1	1	1	1					
1	1	1	1	1	3	1	1	1	1
1	1	1	5	1	4	1	1	1	1
1	1	1	1	1	1	1	1	1	1
1	1	1	1	1	1	1	1	1	1
1	1	1	1	1	1	2	3	1	1
1	1	1	1	1	3	3	3	3	3
1	1	1	1	1	4	1	4	1	3
2	1	1	5	1	3	1	3	3	4
1	1	1	2	1	3	1	3	1	1
1	1	1	1	1	1	1	1	1	1
2	2	1	1	1	1	1	2	2	3
1	1	1	5	1	4	4	4	1	4
1	2	1	5	1	1	1	1	1	1

1	1	1	1	1	1	1	1	1	1
1	1	1	3	1	3	1	3	1	1
1	1	1	5	1	3	1	1	1	1
1	1	1	1	1	4	4	4	1	4
1	1	1	5	1	3	1	1	1	1
1	1	1	5	1	3	1	1	1	1
1	1	1	1	1	1	1	1	1	1
1	1	1	3	1	3	1	1	1	1
1	1	1	1	1	1	2	1	1	1
1	1	1	5	1	1	1	3	1	1
1	1	1	1	1	4	4	4	1	1
1	1	1	5	1	1	1	4	2	1
1	1	1	1	1	3	1	3	1	1
1	1	1	1	1	1	1	1	1	1
1	1	1	1	1	1	1	2	1	1
1	1	1	2	1	1	1	3	1	1
1	1	1	1	1	1	1	1	1	1
1	1	1	1	1	4	1	4	4	4
1	1	1	1	1	4	1	4	4	4
1	1	1	5	1	1	1	1	1	1
1	1	1	1	1	2	2	1	1	1
1	1	1	1	1	1	1	1	1	1
1	1	1	1	1	4	4	4	4	4
1	1	1	4	2	1	1	1	1	1
1	1	1	5	1	1	1	1	1	1
1	2	1	2	1	1	1	1	1	1
1	1	1	2	1	1	1	1	1	1
1	1	1	2	1	4	1	4	1	1
1	1	1	2	1	4	1	1	1	1
1	1	1	3	1	2	1	1	1	3
1	2	1	4	1	1	1	1	1	1
1	1	1	2	1	3	1	3	1	1
1	1	1	2	1	1	1	1	1	1
1	1	1	4	1	1	1	1	1	1
1	1	1	1	1	3	1	1	1	1
	1	1	4	1	3	3	3	3	1
1	1	1	5	1	1	1	1	1	1
1	1	1	1	1	3	1	3	3	3
1	1	1	1	1	2	1	1	1	1
1	1	1	2	1					
1	1	1	1	1	3	1	1	1	1
1	1	1	2	1	3	1	1	2	1
1	1	1	2	1	3	1	3	1	1
1	1	1	1	1	1	1	1	1	1
1	1	1	5	1	1	1	1	1	2
1	1	1	5	1	3	3	3	3	1
1	1	1	1	1	4	3	1	1	1

1	1	1	5	1	1	1	1	1	1
1	1	1	1	1	3	1	1	1	1
1	1	1	1	1	3	3	1	1	1
1	1	1	1	1	1	1	1	1	1
1	1	1	2	1	4	4	4	1	1
1	1	1	3	1	3	3	3	1	2
1	1	1	3	2	3	1	4	1	3
1	1	1	2	1	4	4	4	4	2
2	1	1	1	1	3	3	1	1	3
2	1	1	1	1	3	3	3	3	3
1	1	1	1	1					
1	1	1	1	1	1	1	1	1	1
1	1	1	1	1	1				
1	1	1	1	1	3	1	1	3	1
1	1	1	1	1					
1	1	1	1	1					
2	1	1	1	2					
1	1	1	1	1	1	1	1	1	1
1	1	1	1	1					
2	2	1	2	1					
1	1	1	1	1	1	1	1	1	1
1	1	1		1					
1	1	1	1	1	1	1	1	1	1
1	1	1	1	1	1	1	1	1	1
1	2	2	1	1					
1	1	1	1	1	1	1	1	1	1
1	1	1	1	1					
1	1	1	1	1	1	1	1	1	1
1	1	1	1	1					
1	1	1	1	1	1	1	1	1	1
1	1	1	1	1	1	1	1	1	1
1	1	1	1	1	2	1	1	1	1
1	1	1	1	1					
1	1	1	1	1					
1	1	1	1	1	1	1	1	1	1
2	1	1	1	1	3	1	1	1	1
2	1	1	2	1	4	4	4	4	4
1	1	1	2	1					
1	1	1	1	1	1	1	1	1	1
1	1	1	1	1	1	1	1	1	1
1	1	1	1	1					
1	2	2	2	1	2	2	3	3	1
1	1	1	1	1	1	1	1	1	1
1	1	1	3	1					
1	1	1	4	1	4	1	2	1	1
1	1	1	4	1	1	1	1	1	1
1	1	1	3	1					

3	1	1	4	1					
1	1	1	3	1	1	2	3	1	1
3	1	1	3	1	3	3	4	4	4
1	1	1	2	1	1	1	1	1	1
1	1	1	2	1	2	2	3	1	2
1	3	1	5	1	1	1	1	1	1
1	1	1	4	1					
1	1	1	1	1					
2	1	1	2	1					
1	1	1	1	1					
1	1	1	3	1	1	1	1	1	1
1	1	1	4	1	4	3	4	1	1
1	1	1	1	1	1	1	1	1	1
2	2	1	1	1	1	1	2	1	2
3	3	4		4	4	4	4	4	4
1	1	1	1	1	1	1	3	1	1
3	1	1	3	1					
1	1	1	2	1	2	3	2	1	1
3	1	1		1	4	4	4	4	4
1	1	1	2	1					
1	1	1	2	1					
1	1	1	1	1					
1	1	2	1	1	3	1	1	1	3
1	1	1	3	1	4	1	1	3	3
1	1	1	4	1					
1	1	1	2	1	1	1	1	1	1
1	1	1	1	1	1	1	1	1	1
1	1	1	5	1	4	4	3	3	1
1	1	1	1	1	1	1	1	1	1
1	1	1	1	1	4	1	1	1	1
1	1	1	3	1	1	1	1	1	1
1	1	1	1	1	1	1	1	1	1
1	1	1	1	2	1	1	1	1	1
1	1	1	1	1	1	1	1	1	1
1	1	1	1	1	4	1	1	1	1
1	1	1	1	1	3	1	1	1	1
1	1	1	1	1	4	4	4	4	1
1	1	1	1	1	4	1	3	1	1
1	1	1	1	1	1	1	1	1	1
2	1	1	1	1	3	1	1	1	1
1	1	1	1	1	1	1	1	1	1
1	1	1	3	1	4	1	1	1	1
1	1	1	5	1	3	1	1	1	1
1	1	1	1	1	1	1	1	1	1
1	1	1	1	1	1	1	1	1	1
1	1	1	2	1	3	1	1	1	1
1	1	1	1	1	3	1	1	1	1

1	1	1	3	1	3	1	1	1	1
3	1	1	1	1	3	1	1	1	1
2	1	1	1	1	1	1	1	1	1
1	1	1	1	1	2	1	1	1	1
1	1	1	1	1					
1	1	1	2	1	3	1	1	1	1
1	1	1	1	1	4	1	1	1	1
1	1	1	1	1	4	1	4	1	1
1	1	1	1	1	4	1	1	1	1
1	1	1	1	1	1	1	1	1	1
1	1	1	1	1	4	1	1	1	1
1	1	1	1	1	4	4	4	1	1
1	1	1	1	1		1	1	1	1
1	1	1	1	1	4	1	1	1	1
1	1	1	1	1	3	1	1	1	1
1	1	1	2	1	1	1	1	1	1
1	1	1	1	1	4	1	1	1	1
1	1	1	1	1	1	1	1	1	1
1	1	1	1	1	1	1	1	1	1
4	4	1	1	1	1	1	1	1	1
3	4	1	1	1	3	1	1	1	1
1	1	1	1	1	2	1	1	1	1
1	1	1	1	1					
1	1	1	2	1	1	1	1	1	1
1	1	1	1	1	1	1	1	1	1
1	1	1	2	1	3	1	1	1	1
1	1	1	1	1	4	1	1	1	1
1	1	1	1	1	4	1	3	1	3
1	1	1	2	1	1	1	1	1	1
1	1	1	1	1	1	1	1	1	1
3	2	1	2	1	4	1	3	1	1
1	1	1	1	1	1	1	1	1	1
1	1	1	1	1	1	1	1	1	1
1	1	1	1	1	1	1	1	1	1
1	1	1	1	1	1	1	1	1	1
2	1	1	2	1	3	1	2	1	1
2	2	1	2		1	1	1	1	1
2	2	2	1	2	4	4	4	4	4
1	1	1	5	1	2	3	1	1	1
1	1	1	1	1	1	1	1	1	1

bringgf	hap12mo	prohjob	takeearn	forc2lea	notmon	hap12	slapped	pushed	hit
1	0	1	1	1	1	0	1	1	1
1	0	1	1	1	1	0	1	1	1
1	0	1	1	1	1	0	1	1	1
1	0	1	1	1	1	0	1	1	1
1	0	1	1	1	1	0	1	1	1
1	0	1	1	1	1	0	1	1	1
1	0	1	1	1	1	0	1	1	1
1	0	1	1	1	1	0	1	1	1
1	0	1	1	1	1	0	1	1	1
1	0	1	1	1	1	0	1	1	1
1	0	1				0	3	1	1
1	1	1		1	1	0	3	2	2
1	0	1	1	1	1	0	1	1	1
1	0	1	1	1	1	0	1	1	1
3	1	1	1	1	1	0	1	1	1
1	0	1	1	1	1	0	1	1	1
1	0	1	1	1	1	0	1	1	1
1	0		1			0	1	1	1
1	1	4		3	1	1	3	3	3
1	0	1	1	1	1	0	1	1	1
1	0	1	1	1	1	0	1	1	1
1	0	1	1	1	1	0	3	3	3
1	0	1	1	1	1	0	1	1	1
1	0	1	1	1	1	0	1	1	1
3	1	1	1		3	1	1	1	1
1	0	4	1	1	1	0	1	1	1
2	0	1	1		1	0	1	1	1
1	0	2	1	1	1	0	3	1	3
1	0	1	1	1	1	0	1	1	1
3	1	3	3	1	1	0	1	1	1
1		1	1	1	1		1	1	1
1	0	1	1	1	1	0	1	1	1
3	0					0	1	1	1
1	0	2	1			0	1	1	1
1	0	1	1	1	1	0		1	1
1	0	1	1			0	3	1	1
3	0	3	3	3	4	1	3	3	3
1	0	1	1	1	1	0	1	1	1
1	0	1	1			0	1	1	1
1	0	1	1			0	1	1	1
1	0					0	1	1	1
1	0	1	1			0	1	1	1
1	0	1	1	1	1	0	1	1	1
1	0	1	1	1	1	0	1	1	1
						0			

1	0	1	1			0	1	1	1
1	0	1	1	1	1	0	1	1	1
1	0	4	1	1	1	0	1	1	1
1	0	1	1	1	1	0	1	1	1
1	0	1	1	1	1	0	1	1	1
1	0	1	1	1	1	0	3	1	1
1	0	1	1			0	1	1	1
1	0	1	1			0	1	1	1
1	0	1	1			0	1	1	1
1	1	1	1	1	1	0	3	1	3
1	0	1	1			0	1	1	1
1	0	1	1	1	1	0	1	1	1
1	0	1	1	1	1	0	1	1	1
1	1	1	1			0	1	1	1
1	0	1	1	1	1	0	1	1	1
1	1	1	1	1	1	0	3	4	1
1	0					0	1	1	1
1	0	4	1		1	0	1	1	1
1	0	1	1	1	1	0	1	1	1
1	0					0	1	1	1
1	0	1	1	1	1	0	2	2	1
1							1	1	1
1		1	1	1	1		1	1	1
1	0	1	1	1	1	0	1	1	1
1	0	1	1	1	1	0	1	1	1
1	0	1	1	1	1	0	1	1	1
1	0	1	2	1	1	0	1	1	1
3	0	1	1	1	1	0	1	1	1
1	0	1	1	1	1	0	1	1	1
1	0	1	1	1	1	0	1	3	3
1	0	1	1	1	1	0	1	1	1
1	0	1	1		1	0	1	1	1
1	0	1	1	2	2	0	1	1	1
1		1	1	1	1	0	1	1	1
1	0	1	1	1	1	0	1	1	1
1	0	1	1	1	1	0	1	1	1
1	0	1	1	1	1	0	1	1	1
4	0	4	4	4	4	0	4	4	4
1	0	1	1	1	1	0	1	1	1
1	0	1	1	2	1		1	1	1
1	0	1	1	1	1	0	1	1	1
1	0	1	1	1	1	0	1	1	1
3	1	3	3	2	2	1	2	3	2
1		1	1	1	1		1	1	1
1	0	1	1	1	1	0	2	3	2
1		1	1	1	1		1	1	1

2	1	1	1	1	1	0	1	1	1
1	0	1	1	1	1	0	1	1	1
		2	2	2	2	1	1	1	1
1	1	1	1	1	1	0	2	2	1
1	1	1	1	1	1	0	3	3	1
1	0	1	1	1	1	0	1	1	1
1	0	1	1	1	1	0	1	1	3
1	1	1	1	1	1	0	1	1	1
3	1	1	1	1	1	0	1	1	1
1	0	1	1	1	1	0	1	1	1
1	1	1	1	1	1	0	1	1	1
1	1	1	1	1	1	0	3	1	3
1	0	1	1	1	1	0	2	1	2
1	0	1	1	1	1	0	1	1	1
1	0	1	1	1	1	0	1	1	1
1	0	1	1	1	1	0	1	1	1
1	1	1	1	1	1	0	2	1	2
1	1	1	1	1	1	0	4	3	4
1	1	1	1	1	1	0	3	1	1
4	1	1	1	4	4	1	4	4	3
1	0	1	1	1	1	0	1	2	1
1	1	1	1	1	1	0	1	1	1
1	1	1	1	1	1	0	1	1	1
1	0	1	1	1	1	0	1	1	1
1	1	1	1	1	1	0	4	1	3
3	0	1	1	1	2	0	1	1	1
1	0	1	1	1	1	0	1	1	1
4	1	1	1	1	4	1	3	1	4
4	1	1	1	1	4	1	3	1	4
1	0	1	1	1	1	0	1	1	1
1	0	2	1			1	1	1	1
1	0	1	1		1	0	1	1	1
2	1	1	1	1	1	0	1	1	1
1	0	1	1	1	1	0	1	1	1
1	0	1	1			0	1	1	1
1	1	3	3	1	1	0	1	1	1
1	0	1	1	1	1	0	1	1	1
1	0	1	1	1	1	0	1	1	1
1	0	1	1	1	1	0	1	1	1
3	1	1	2	3	3	1	3	1	4
1	0	1	1	1	1	0	1	1	1
2	1	1	1	1	1	0	2	1	2
1	0	1	1	1	1	0	2	1	2
1	0	1	1	1	1	0	1	1	1
1	0	1	1	1	1	0	1	1	1
1	0	2	1	2	1	0	1	1	1
1	1	2	2	2	2	0	1	1	1

1	1	1	1	1	1	0	1	1	1
1	0	1	1	1	1	0	1	1	1
1	0	1	1	1	3	0	1	1	1
1	0	1	1	1	1	0	1	1	1
1	0	1	1	1	1	0	1	1	1
1	0	1	1	1	1	0	1	1	1
1	1	1	1	1	1	0	1	1	1
1	0	1	1	1	1	0	1	1	2
1	1	1	1	1	1	0	1	1	1
1	1	1	1	1	1	0	1	1	1
1	1	1	1	1	1	0	1	1	1
1	0	1	1	1	1	0	1	1	1
1	0	1	1	1	1	0	1	1	1
1	0	3	3	3	1	0	2	1	1
4	0	1	1	1	1	0	4	4	4
1	0	1	1	1	1	0	1	1	1
1	0	1	1	1	1	0	1	1	1
1	0	1	1	1	1	0	1	1	1
1	1	1	1	1	1	0	1	1	1
1	1	1	1	1	1	0	1	1	1
1	0	1	1	1	1	0	1	1	1
1	1	1	1	1	1	0	1	1	1
1	0	1					1	1	1
2	0	1	1	1	1	0	1	1	1
1	0	1	1	1	3	1	1	1	1
1	0	1	1	1	1	0	1	1	1
1	0	1	1	1	1	0	1	1	1
1	0	1	1			0	1	1	1
1	0	1	1	1	1	0	1	1	1
1	0	2	1	1	3	1	1	1	1
1	0	1	1	3	4	0	1	1	1
1	0	1	1			0	1	1	1
1	0	1	1			0	1	1	1
1	0	1	1	1	1	0	1	1	1
1	0	1	2	1	1	1	1	1	1
1	0	1					1	1	1
1	1	1	1	1	1	0	1	1	1
1	0	1	1	1	1	0	1	1	1
3	1	1	2	2	2	0	3	1	4
	0					0			
1	1	4	1	1	1	0	4	3	3
1	0	3	1	1	1	0	1	2	1
1	0	1	1	1	1	0	1	1	1
1	0	1	1			0	1	1	1
1	1	4	1	1	1	0	3	1	3
1	0	1	1	1	1	0	1	1	1

1	0	1	1	1	1	0	1	1	1
1	0	4	1	1	1	0	1	1	1
							1		
1	1	1	1	1	1	0	1	1	1
1	0	1		1	1	0	1	1	1
1	0	1	1	1	1	0	1	1	1
1	0	1	1	1	2	1	1	1	1
1	0	1	1	1	1	0	1	1	1
1	0	1	1	1	1	0	1	1	1
1	1	2	1	1	1	1	1	1	1
1	0	1				0	1	1	1
1	0	1	1			0	1	1	1
1	0	1	1	1	1	0	1	1	1
1	0	4	1	1	1	0	3	1	1
1	1	1	1	4	4	1	4	4	4
1	0	1	1	1	1	0	1	1	1
1	0	1	1	1	1	0	1	1	1
1	0	1	1	1	1	0	1	1	1
1	1	1	1	1	1	0	1	1	1
1	0	1	1	1	1	0	1	1	1
1	0	1	1			0	1	1	1
1	0	1	1	1	1	0	1	1	1
1	0	1	1	1	1	0	1	1	1
1	1	1	1	4	4	1	4	4	4
1	0	1	1	1	1	0	1	1	1
1	0	1	1	1	1	0	1	1	1
1	0	1	1	1	1	0	1	1	1
1	0	1		1	1	0	1	1	1
1	0	1	1	1	1	0	1	1	1
1	1	1	1	1	1	0	4	4	4
1	0	1	1	1	1	0	1	1	1
1	0	1		1	4	1	1	1	1
1	0	1	1			0	4	1	1
1	0	1	1	1	1	0	1	1	1
1	0	1	1	1	1	0	1	1	1
1	0	1	1	1	1	0	1	1	1
1	1	1	1			1	1	1	1
1	1	1	1	1	1	1	1	1	1
1	1	1	1	1	1	0	1	1	1
4	1	2	1	1	4	1	1	1	1
4	1	1	1	4	4	1	4	3	3
1	0	4	1	1	1	0	1	1	1
1	0	1	1	1	1	0	1	1	1
1	0	1		1	1	0	1	1	1

		1	1	1	1	0	1	1	1
4	1	1		1	1	1	4	4	4
1	0	1	1	1	1	0	1	1	1
1	0	1	1	1	1	0	1	1	1
1	0	2	1	1	1	0	1	1	1
1	0	1	1	1	1	0	1	1	1
1	0	1	1			0	1	1	1
1	0	1	1			0	1	1	1
1	1	1	1	1	1	0	1	1	1
1	1	1	1	1	1	0	3	1	1
4	1	1	1	1	1	0	4	3	3
1	0	1	1	1	1	0	1	1	1
1	0	3	1			1	1	1	1
1	0	1	1	1	4	1	1	1	1
1	0	1	1	1	1	0	1	1	1
1	0	1	1	1	1	0	1	1	1
1	0	1	1	1	1	0	1	1	1
1	0	1	1			0	1	1	1
1	0	1	1			0	1	1	1
1	0	1				0	1	1	1
1	0	1	1	1	1	0	1	1	1
1	0	3	1			0	1	1	1
1	1	1	1	1	1	0	1	1	1
4	0	3	1	1	4	1	4	1	4
4	0	1	3	3	1	0	3	3	3
1	0	1	1	1	1	0	1	1	1
1	0	1	1			0	2	1	1
1	1	1	1	1	4	1	1	1	1
1	0	1	1	1	1	0	1	1	1
1	0	4	1	1	1	0	1	1	1
1	0	1	1	1	1	0	1	1	1
1	0	1	1	1	1	0	1	1	1
1	0	1	1	1	1	0	1	1	1
2	1	1	1	1	1	0	2	2	1
1	0	1				0	1	1	1
1	0	1	1	1	1	0	1	1	1
1	0	1	1	2	1	0	1	2	1
2	0	1	1	1	1	0	1	1	1
1	1	1	1	1	1	0	4	4	4
1	0					0	2	2	2
1	0	1	1	1	1	0	1	1	1
1	0	2	2	1	1	0	2	2	2
1	0	1	1	1	1	0	1	1	1
1	0	1	1	1	1	0	1	1	1
1	0	1	1	1	1	0	1	1	1
1	0	1	1	1	1	0	1	1	1
1	0	1	1			0	1	2	1

1	0	1	1	1	1	0	1	1	1
1	0	1	1			0	1	1	1
1	0	1	1	1	1	0	1	1	1
1	0	1	1			0	1	1	1
	0					0			
1	0	1	1	1	1	0	1	1	1
1	0	1	1	1	1	0	1	1	1
1	0	1	1	4	4	0	1	1	1
1	0	1	1	1	1	0	1	1	1
3	1	4	3	3	3	1	1	1	1
1	0	1	1	1	1	0	1	1	1
1	0	4	2	4	4	0	2	1	1
1	0	1				0	1	1	1
1	0	4	4	1	1	0	1	1	1
1	0	4	4	1	1	0	1	1	1
1	1	4	1	1	1	0	1	1	1
1	1	3	1	1	2	1	2	1	1
1	0	1	1	1		0	1	1	1
1	0	1	1	1	1	0	4	3	2
1	0	1	1	1	1	0	1	1	1
1	0	1	1	1	1	0	1	1	1
1	1	3	4	1	1	0	1	3	1
4	1	4	3	3	4	1	4	4	4
1	0	1	1	1	1	0	1	1	1
1	0	1	1	1	1	0	1	2	2
1	1	3	1	4	4	1	1	4	1
1	0	1	1	1	1	0	1	1	1
1	0	1	1	1	1	0	1	1	1
1	0	1	1	1	1	0	1	1	1
1	1	3	1	1	1	1	1	2	2
1	0	1	1	1	1	0	4	4	3
1	0	1	1	1	2	1	1	1	1
1	0	1	1	1	1	0	1	1	1
1	0	1	1	1	1	0	1	1	1
1	0	1	1	1	1	0	1	1	1
3	1	4	1	3	1	0	1	3	1
1	0	1	1	1	1	0	1	1	1
1	0	1	1	1	1	0	1	1	1
1	0	1	1			0	1	1	1
1	0	1	1	1	1	0	1	1	1
1	0	1	1	1	1	0	1	1	1
2	0	3	1	1	1	0	3	1	3
1	0	1	1	1	1	0	1	1	1
1	0	1	1	1	1	0	1	3	3
1	0	1	1	1	1	0	1	1	1
1	0	3	1	1	1	0	3	3	3
3	1	4	2	1	1	1	1	1	1

3	1	4	2	1	1	1	1	1	1
1	0	1	1	1	1	0	1	1	1
3	1	1	1	1	1		2	3	2
1	0	2	1	1	1	0	1	3	1
1	0	1	1	1	1	0	1	1	1
1	0	1	1	1	1	0	1	1	1
1	0	1	4	1	4	1	1	1	1
1	0	1	4	1	4	1	1	1	1
1	0	1	1	1	1	0	1	1	1
1	0	1	1	1	1	0	1	1	1
1	0	2	1	1	3	1	1	1	1
1	0	3		1	1	0	1	1	1
3	1	3	1	4	4	1	4	4	4
2	0	1	3	1	1	0	1	1	1
1	0	4	4	1	4	1	1	1	1
1	0	3	1		1	0	1	1	1
2	1	1	1			0	1	1	1
1	0	3	1	1	1	0	1	1	1
4	1	2	1	4	4	1	1	3	1
2	0	1	1	1	1	0	4	4	4
1	0	1	1	1	1	0	1	1	1
1	1	1	1	1	1	0	1	1	1
1	0	1	1	1	1	0	1	1	1
1	0	1		1	1	0	1	1	1
1	0	2	1	1	1	0	1	1	1
1	0	1	1	1	1	0	1	1	1
1	0	1	1	1	1	0	1	1	1
1	1	1	1	1	1	0	1	1	1
1	1	4	1	1	1	1	2	2	1
1	0	4	1	1	1	0	1	1	1
1	0	1	1	1	1	0	1	1	1
1	0	1	1	1	1	0	1	1	1
1	0	2	1	1	1	0	1	2	1
1	1	1	1	1	1	0	3	3	3
1	0	4	1	1	1	0	1	1	1
1	0	4		1	1	0	3	1	1
1	0	1	1	1	1	0	1	1	1
1		1	1	1	1		1	1	1
1		1	1	1	1	0	1	1	1
1	0	1	1	1	1	0	1	1	1
1	0	1	1	1	1	0	1	1	1
2	0	2	1	1	1	0	1	1	1
1	1	1	1	2	1	1	1	2	1
1	0	1	1	1	1	0	1	1	1
1	0	1	1	1	1	0	1	1	1
1	0	1	1	1	1	0	1	1	1

1	0	1	1	1	1	0	1	1	1
1	0	1	1	1	1	0	1	2	1
1	0	1	2	1	1	0	1	1	1
1	0	1	1	1	1	0	1	1	1
4	0	1	1	1	1	0	1	1	1
2	0	1	1	1	1	0	1	1	1
1	0	1	1	1	1	0	1	1	1
1	0	1	1	1	1	0	1	1	1
1	0	3	1	1	1	1	3	3	1
3	0	3	3	3	3	0	3	3	3
1	0	2	2	3	3	1	2	3	2
1		1	1	1	1	0	1	1	1
4	1	1	1	1	3	1	1	1	3
1	1	1	1	1	1	0	1	1	1
1	1	1	1	1	1	0	1	1	1
2	0	1	1	1	2	1	1	1	3
1	0	1	1	1	1	0	1	1	1
1	0	1	1	1	1	0	1	1	1
1	1	1	1	1	1	0	1	1	4
1	0	1	1	1	1	0	1	1	1
1	1	1	1	4	4	1	4	4	4
4	1	4	1	1	4	1	4	4	4
4	0	1	1	1	4	1	4	4	4
1	1	1	1	1	4	1	1	4	4
1	0	1	1	1	1	0	2	1	1
1	0	1	1	1	1	0	1	1	1
3	1	1	1	1	4	1	4	4	4
1	0	4	3	3	1	1	1	1	2
1	0	1	1	1	1	0	1	1	1
1	1	1	1	1	1	0	1	1	1
1	0	4	1	1	1	1	1	1	1
1	0	1	1	1	1	0	1	1	1
1	0	1	1	1	1	0	1	1	1
1	0	1	1	1	1	0	1	1	1
1		1	1	1	1	0	1	1	1
1	1	1	1	1	1	0	3	3	1
1	0	1	1	1	2	1	2	1	1
1	0	1	1	1	1	0	1	1	1
1	0	1	1	1	1	0	1	1	1
3	1	2	1	1	1	0	2	1	2
1	0	1	1	1	1	0	1	1	1
1	0	4	1	1	4	1	4	4	4
3	0	1	1	1	1	0	2	3	3
1	0	1	1	1	1	0	1	1	1
1	0	2	1	1	1	0	1	1	1
1	0	3	1	1	1	0	1	1	1
1	0	2	1	1	1	0	1	1	1

1	0	1	1	1	1	0	1	1	1
1	0	1	1	1	1	0	1	1	1
1	0	1	1	1	4	1	1	3	1
3	1	1	1	3	1	1	3	1	1
1	0	1	1	1	1	0	2	2	2
1	0	1	1	1	1	0	4	1	1
1	0	1	1	1	1	0	1	1	1
1	0	1	1	1	1	0	1	1	1
1	0	1	1	1	1	0	1	1	1
1	0	1	1	1	1	0	1	1	1
1	0	1	1	1	1	0	1	1	1
4	1	4	1	4	4	1	4	4	4
1	0	1	1	1	1	0	1	1	1
1	0	1	1	1	1	0	1	1	1
1	0	1	1	1	1	0	1	1	1
1	0	1	1	1	1	0	1	1	1
1	0	1	1	1	4	1	2	2	1
1	1	4	1	1	1	0	4	4	4
1	0	1	1	1	1	0	3	1	1
1	0	3	1	1	1	0	1	1	1
1	1	4	1	1	4	1	3	2	2
1	0	1	1	1	1	0	1	1	1
1	1	1	1	1	1	0	1	1	1
1	0	1	1	1	1	0	1	1	1
1	0	1	1	1	1	0	1	1	1
1	0	1	1	1	1	0	1	1	1
1	0	3	3	1	1	0	3	3	3
4	0	2	3	4	4	0	4	4	4
1	1	1	1	1	1	0	3	2	1
1	0	1	1	1	1	0	1	1	1
1	0	1	1	1	2	1	1	1	1
1	0	1	1			0	1	1	1
1	0	1	1			0	1	1	1
1	0	1	1		1	0	1	1	1
1	0	4	1	1	2	1	1	1	1
1	1	4	1	1	4	1	1	4	4
1	0	1	1	1	1	0	4	1	1
3	1	4	4	4	2	1	4	4	4
4	0	1	1	1	3	0	3	3	1
1	1	1	1		1	0	1	1	1
1	0	4	1	1	1	0	1	1	1
1	0	1	1	1	1	0	1	1	1
1	1	4	1	1	1	1	4	4	4
1	1	1	1	1	1	0	1	1	1
4	0	4	1	1	1	0	1	3	4
1	0	1	1	1	1	0	1	1	1
1	0	3	1	1	1	0	1	1	1

1	1	1	1	1	1	0	2	2	2
1	0	1	1	1	1	0	1	1	1
1	0	1	1	1	1	0	1	1	1
1	0	1	1	1	2	0	2	1	1
1	0	4	1	1	1	0	2	2	2
1	0	1	1	1	1	0	2	2	1
1	0	1	1	1	4	1	1	1	1
1	0	1	1	1	1	0	1	1	1
1	1	1	1	1	1	0	1	1	1
1	0	4	1	1	1	0	1	1	1
1	0	1	1	1	1	0	1	1	1
1	0	4	1	1	1	0	4	4	4
1	0	1	1	1	1	0	1	1	1
1	0	1	1	1	1	0	1	1	1
1	0	1	1			0	4	1	1
1	0	1	1	1	1	0	1	1	1
2	1	4	3	1	3	1	1	1	4
1	0	1	1	1	1	0	1	1	1
1	1	1	1	1	1	0	1	1	1
1	0	1	1	1	4	1	1	1	1
1	0	1		1	2	0	1	1	1
1	0	1	1			0	1	1	1
1	0	1	1	1	1	0	1	1	1
1	0	1	1	1	1	0	1	1	1
3	0	1	1	1	1	0	1	1	1
1	1	4	1	1	1	0	1	3	4
1	0	1	1	1	1	0	1	1	1
1	0	1	1	1	1	0	1	1	1
1	1	1	1			0	1	1	1
1	1	1	1		1	0	1	1	1
1	1	1	3	1	2	1	1	1	1
1	0	1	1	1	1	0	1	1	1
1	0	4	1	1	1	0	1	1	1
1	0	3	1	1	1	0	1	1	1
1	1	1	1		1	1	1	1	1
1	0	1	1	1	1	0	1	1	1
1	0	1	1	1	1	0	1	1	1
1	0	1	1	1	1	0	1	1	1
1	1	1		1	1	1	3	3	3
1	1	1		1	1	0	1	1	1
1	1	1		1	1	0	1	1	1
4	1	1	1	4	4	1	4	3	4
1	0	1	1	1	1	0	1	1	1
1	1	4		1	1	0	1	1	1
1	0	1	1	1	1	0	1	1	1
1	0	1	1	1	1	0	2	1	1
1	0	1			1	0	1	1	1

1	0	1	1	1	1	0	1	1	1
1	0	1		1	1	0	1	1	1
1	0	1	1	1	4	1	1	1	1
1	0	1	1	1	1	0	1	1	1
1	0	1	1	1	1	0	1	1	1
1	0	1	1	1	1	0	1	1	1
1	0	4	4	1	4	1	4	4	4
1	1	4	1	1	1	0	1	1	1
1	0	1	1	1	1	0	3	3	3
1	0	1	1	1	4	1	1	1	1
1	0	1	1	1	1	0	1	1	1
1	1	4	1	1	1	0	1	1	1
1	0	1	1	1	1	0	1	1	1
1	0	1	1	1	1	0	1	1	1
1	0	1	1	1	1	0	1	1	1
1	0	1	1	1	1	0	4	4	4
1	0	1	1	1	1	0	1	1	1
1	0	1		1	1	0	3	1	3
1	0	1	1	1	1	0	1	1	1
1	0	1	1	1	1	0	1	1	1
1	0	1	1	1	1	0	2	1	1
1	0	1	1	1	1	0	1	1	1
1	0	1	1	1	1	0	3	1	1
1	0	1			3	1	3	1	1
1	0	1	1	1	1	0	1	1	1
4	0	1	1		4	0	1	1	1
1	1	1	1	1	1	0	1	1	1
1	1	1	1			0	3	3	1
1	0	1	1	1	1	0	1	1	1
1	0	1	1	1	1	0	1	1	1
1	0					0	1	1	1
1	0	1	1			0	1	1	1
1	0	1	1	1	1	0	1	1	1
3	1	4	1	3	4	1	4	3	4
1	0	1	1	1	1	0	1	1	1
1	0	1	1		1	1	1	1	1
1	0	1	1	1	1	0	1	1	1
1	0	2	1	1	2	0	3	2	1
1	0	1	1	1	1	0	1	1	1
1	0	1	1	1	3	1	1	1	1
1	0	4	1	1	3	1	1	1	1
1	0	1	1			0	1	1	1
4	0	1	1		2	0	1	1	1
1	1	1	1	1	2	1	3	2	1
1	0	1	1	1	1	0	1	1	1
3	0	1	1			0	3	3	3

3	0	2	1	2	2	0	2	2	2
1	0	4	1	1	1	0	4	1	1
1	0		1	1	1	0	1	1	1
4	1	4	1	3	1	0	1	2	1
1	0	1	1	1	1	0	1	1	1
1	0	1	1	1	1	0	1	1	1
1	0	1	1	1	4	1	2	1	1
1	0	1	1	1	1	0	1	1	1
1	0	1	1	1	1	0	1	1	1
1	0	1	1	1	1	0	1	2	1
1	0	1	1	1	1	0	1	1	1
1	0	1	1			0	1	1	1
1	0	1	1	2	1	0	1	3	1
1	0	1	1	1	1	0	1	1	1
1	0	1	1	1	1	0	1	1	1
4	1	4	1	1	1	1	4	4	4
1	0	1	1	1	1	0	1	1	1
1	0	1	1	1	1	0	1	1	1
3	1	4	1	1	1	0	1	3	1
1	0	1	1	1	4	0	1	1	1
1	0	1	1	1	1	0	1	1	1
1	0	1	1			0	1	1	1
1	1	1	1	1	1	0	1	2	2
1	0	1	1	1	1	0	1	2	1
3	1	4	1	1	1	0	1	1	1
1	1	1	1			0	1	2	1
1	0	4	1	1	1	0	1	2	1
1	0	1	1	1	1	0	1	1	1
1	0	1	1	1	1	0	2	1	1
4	1	1	1	1	1	0	4	4	4
1	0	4	1	1	1	0	1	1	1
1	0	4	1	1	1	1	1	1	1
1	0	4	1	1	1	0	1	4	1
1	0	1	1			0	1	1	1
1	0	1	1	1	1	0	1	1	1
1	0	1	1	1	1	0	2	3	2
1	0	1	1			0	1	1	1
4	1	1	1	4	4	1	4	4	1
1	0	1	1	1	1	0	1	1	1
1	0	4	1			0	1	1	1
1	0	1	1	1	1	0	3	1	3
1	0	4	1	1	1	0	3	3	3
1	0	4	1	1	1	0	1	1	1
1	0	1	1			0	1	1	1
1	0	1	1			0	1	1	1
3	1	3	1	3	1	1	4	4	3

1	0	1	1	1	1	0	1	2	1
1	0	1	1	1	1	0	1	1	1
1	0	1	1	1	1	0	1	1	1
1	1	1	1	1	1	0	3	3	1
1	0	1	1	1	1	0	1	1	1
1	0	1	1	1	1	0	1	1	1
1	0	1	1	1	1	0	1	1	1
1	1	3	1	1	4	1	1	2	1
1	0	1	1	1	2	0	1	1	1
1	0	1	1	1	1	0	3	1	3
1	0	1	1	1	1	0	1	1	1
1	0	1	1	1	1	0	1	1	1
1	0	1	1	1	1	0	1	1	1
3	0	1		1	1	0	3	3	3
1	0	1	1	1	1	0	1	1	1
1	0	1	1	1	1	0	1	1	1
1	0	1	1	1	1	0	1	1	1
1	0	1	1	1	1	0	1	1	1
1	0	1	1	1	1	0	1	1	1
1	0	1	1	2	1	0	2	1	1
1	0	1	1	1	1	0	1	1	1
1	0	1	1	1	1	0	1	1	1
1	0	1	1	1	1	0	1	1	1
1	0	1	1	1	1	0	1	1	1
1	0	3	1	1	1	0	1	1	1
4	1	3	2	4	3	1	3	1	1
	0					0			
1	0	2	1	1	1	0	1	1	1
4	0	2	1	1	3		4	4	4
1	0			1	1	0	1	1	1
1	0	1	1	1	1	0	3	1	2
1	0	1	1	1	1	0	1	1	1
1	0	4	1	1	1	0	1	1	1
1	0	1	1			0	1	1	1
1	0	1	1	2	3	0	3	3	1
1	0	1	1	1	1	0	1	1	1
1	0	1	1	1	1	0	1	1	1
1	0	1	1	1	1	0	1	1	1
1	0	1	1	1	1	0	1	1	1
1	0	1	1	1	1	0	1	1	1
1	1	3	1	1		1	1	1	1
1	0	1	1	1	1	0	1	1	1
1	0	1	1	1	1	0	1	1	1
1	0	1	1	1	1	0	3	2	2
1	0	1	1	1	1	0	1	1	1
1	0	1	1	1	1	0	4	3	4
1	0	1	1	1	1	0	1	1	1

1	0	1	1	1	1	0	1	1	1
1	0	1	1	2	4	1	1	1	1
3	1	4	1	1	1	1	1	1	1
1	0	2	1	1	1	0	1	1	1
4	1	4	1	4	4	1	4	4	4
3	1	4	1	1	4	1	1	1	1
1	1	3	1	2	1	0	4	3	1
1	1	3	1	2	1	0	4	3	1
1	0	4	1	1	1	0	1	1	1
1	0	1	1	1	1	0	1	1	1
1	0	1	1	1	1	0	1	1	1
1	0	1	1	1	1	0	1	1	1
1	0	1	1	1	1	0	1	3	1
1	0	1	1	1	1	0	1	2	2
1	0	1	1	1	1	0	1	1	1
1	0	1	1	1	1	0	1	1	1
1	0	4	1	1	3	1	1	1	1
1	1	3	3	1	4	1	1	1	1
1	0	1		1	1	0	3	1	1
1	0	1	1	1	1	0	1	1	1
4	1	2	3	1	4	1	3	3	3
4	1	1	1	1	3	1	3	3	3
1	0	1	1	1	1	0	1	1	1
1	0	1	1	1	1	0	1	1	1
1	0	1	1	1	1	0	1	1	1
3	1	2	2	3	3	1	4	3	4
1	1	1	1	1	1	0	1	2	1
1	0	1	1	1	1	0	1	1	1
1	0	1	1	1	1	0	1	1	1
1	0	1	1	1	1	0	1	1	1
3	1	1	4	4	1	1	1	1	1
1	0	1	1	1	1	0	1	1	1
1	0	1	1	1	1	0	1	1	1
1	0	1	1	1	1	0	1	1	1
1	0	4	3	3	3	0	2	1	1
1	0	4	3	3	3	0	2	1	1
1	0	1	1			0	1	1	1
1	0	1	1			0	1	1	1
1	0	4	3	1	1	0	1	1	1
1	1	1	1	1	3	1	1	1	1
1	0	4	1	1	1	0	1	1	1
1	0	1	1	1	1	0	3	3	3
4	1	4	4	4	2	1	3	3	2
1	0	1	1	1	1	0	1	1	1
1	0	4	1	1	1	0	1	2	1
1	0	1	4	1	4	0	1	1	1
1	0	1	1	1	1	0	1	1	1

1	0	4	1	1	1	0	1	3	1
1	0	1	1	1	1	0	1	1	1
1	0	1	1	1	1	0	1	1	1
1	0	1	1	1	1	0	1	3	1
1	0	4	1	1	1	0	1	1	1
3	1	2	2	2	2	1	1	1	1
3	1	2	2	2	2	1	1	1	1
1	1	1	1	1	1	0	1	1	1
1	1	1	3	1	1	0	1	3	3
1	0	1	1	1	1	0	1	1	1
4	1	3	1	1	4	1	3	3	4
4	1	3	1	1	4	1	3	3	4
1	0	1	1	1	1	0	1	1	1
1	0	1	1	3	3	1	3	1	1
4	1	4	4	4	4	1	4	4	4
1	0	4	1	1	3	1	1	3	3
1	1	1	2	1	1	0	1	1	1
1	1	2	1	1	1	0	4	1	2
1	0	1	1	1	1	0	1	1	1
1	1	1	1	4	1	1	4	4	2
1	0	1	1	1	1	0	1	1	1
1	1	1	1	1	1	0	1	3	1
1	0	1	1	1	1	0	1	1	1
1	0	4	1	1	1	0	1	1	1
1	0	4	1	1	1	0	1	1	1
1		1	1	1	1	0	1	1	1
1	0	4	3	3	2	0	3	3	2
1	0	1	1	1	1	0	1	2	1
1	0	4	1	2	2	0	2	2	3
1	0	4	1	1	1	1	1	1	1
1	0	1	1	1	1	0	1	1	1
1	0	1	1	1	1	0	1	1	1
1	0	1	1	1	1	0	1	1	1
1	0	1	1	1	1	0	1	1	1
1	0	2	1	1	1	1	1	2	1
1	0	1	1	1	1	0	1	1	1
1	0	1	1	1	1	0	1	1	1
1	0	1	1	1	1	0	1	1	1
1	0	1	1	1		0	1	1	1
2	1	1	1	1	1	0	1	1	1
1	0	1	1	1	1	0	1	1	1
1	0	4	1	2	1	0	1	1	2
1	0	1	1	1	1	0	1	1	1
1	0	1	1	1	1	0	1	1	1
1	0	1	1	1	1	0	1	1	1
1	0	4	1	1	1	0	1	1	1
1	0	1	1	1	1	0	1	1	1

1	0	4	1	1	1	0	1	1	1
1	0	2	1	1	1	1	1	1	1
1	0	1	3	1	1	0	1	1	1
3	1	1	1	1	1	0	1	1	1
1	0	1	1	1	1	0	1	1	1
1	0	1	1	1	1	0	1	1	1
1	0	1	1	1	1	0	1	1	1
1	0	4	4	1	1	1	1	3	1
1	0	4	3	1	1	0	1	1	1
3	0	1	1	1	1	0	1	3	4
3	0	3	3	3	3	0	3	3	3
1	0	1	1	1	1	0	1	1	1
1	0	1	1	1	1	0	1	1	1
1	0	1	1	1	1	0	3	3	3
4	1	4	4	4	4	1	4	4	4
1	0	1	1	1	1	0	1	1	1
1	0	1	1	1	1	0	1	1	3
2	0	1	1	1	1	0	2	2	2
1	1	3	2	1	1	1	1	1	1
1	0	1	1	1	1	0	1	1	1
1	0	1	1	1	1		1	1	1
1	0	1	1	1	1	0	1	1	1
1	0	1	1	1	1	0	1	1	1
1	0	1	1	1	3	0	3	3	3
1	0	1	1	1	1	0	1	1	1
1	0	1	1	1	1	0	1	1	1
1	0	1	1	1	1	0	1	1	1
1	0	1	1	1	1	0	1	1	1
1	0	1	1	1	1	0	1	1	1
1	0	1	1	1	1	0	1	1	1
4	1	4	4	4	4	1	4	4	4
1	0	1	1	1	1	0	1	1	1
1	1	4	4	1	1	1	1	1	1
1	0	1	1	1	1	0	1	1	1
3	0	1	1	1	1	0	1	1	1
4	0	1	1	1	1	0	1	1	1
1	0	1	1	1	1	0	1	1	1
1	0	1	1	1	1	0	1	1	1
1	0	1	1	1	1	0	1	1	1
1	0	1	1	1	1	0	1	1	1
1		1	1	1	1		1	1	1
1		1	1	1	1	0	1	1	1
3	1				1	0	4	3	3
1	0	1	1	1	1	0	1	1	1
1	0	1	1	1	1	0	3	3	3
1	0	1	1	1	1	0	1	1	1
1	0	1	1	2	1	0	1	1	1

1	0	1	1	1	1	0	1	1	1
3	0	3	3	2	2	0	3	3	1
3	1	1	1	1	1	0	1	1	1
3	0	1	1	1	1	0	1	1	1
1	0	1	1	1	1	0	1	1	1
1	0	3	1	1	1	0	1	1	1
3	1	1	4	1	3	0	3	3	3
4	0	4	4	4	4	0	4	4	4
1	0	4	4	1	1	0	1	3	3
1	1	4	1	1	4	1	1	1	1
4	1	1	3	4	4	1	4	4	4
1	0	1	3	1	1	0	2	1	2
1	1	1	1	1	1	0	3	3	3
2	0	1	1	1	1	0	1	4	4
1	1	1	1	1	1	0	1	1	1
3	0	1	1	4	3	0	4	1	4
1	0	1	1	1	1	0	4	1	4
1	0	1	1	1	1	0	1	1	1
1	0	1	1	1	1	0	1	1	1
1	0	1	1	1	1	0	1	1	1
1	0	1	1	1	3	1	4	1	1
4	1	1	1	1	1	0	1	3	1
1	1	4	1	1	1	1	1	1	1
1	0	4	1	1	1	1	1	1	1
1	1	1	1	1	1	0	1	1	1
1	0	1	1			0	1	1	1
1	0	4	1	1	1	0	1	3	1
2	0	1	1	1	1	0	1	2	1
4	1	4	4	4	4	1	4	4	4
1	0	1	1	3	3	0	1	3	3
4	0	4	4	4	4	1	4	4	4
1	1	1	1	1	1	0	1	1	1
1	1	1	1	1	1	0	1	1	1
1	0	1	1	1	1	0	1	1	1
3	0	4	4	1	4	0	1	4	4
3	1	2	1	1	1	1	3	3	1
1	1	1	1	1	1	0	4	4	4
1	0	1	1	1		0	1	1	1
1	0	1	1	1	1	0	1	1	1
1	1	1	1	1	1	0	1	1	1
3	1	4	1	1	4	1	4	3	4
1	1	4	1	1	1	1	4	3	3
1	0	1	1	1	1	0	1	1	1
1	1	1	1	1	1	0	4	3	3
1	0	4	1	4	4	0	4	1	4
1	1	4	2	1	1	0	4	4	4
1	0	1	1	1	1	0	2	2	2

2	0	1	1	1	1	0	1	1	1
1	0	4	1	1	1	0	2	1	1
1	0	1	1	1	1	0	2	1	1
1	1	1	2	1	1	1	1	1	1
1	0	1	1	1	1	0	1	1	1
1	0	1	1	1	1	0	3	1	3
1	0	1	1	1	1	0	1	1	1
1	0	1	1	1	1	0	1	1	1
1	0	1	1	3	4	1	2	2	3
1	0	1	1		1	0	1	1	1
3	0	1	1	1	1	0	1	1	1
1	0	2	1	1	1	0	1	1	1
1	0	1	1	1	1	0	3	3	3
1	0	1	1	1	1	0	3	3	3
1	0	1	1	1	1	0	1	1	1
1	0	1	1	1	1	0	1	1	1
1	1	1	1	1	1	0	1	1	1
1	0	1	1	1	1	0	1	1	1
1	0	1	1	1	1	0	1	1	1
1	0	1	1	1	1	0	1	1	1
1	0	2	1	1	1	0	3	1	1
1	0	4	1	1	1	0	1	3	1
1	1	1	1	1	1	0	2	3	2
1	0	1	1	1	1	0	1	1	3
1	0	1	1	1	1	0	1	1	1
1	0	1	1	1	1	0	3	3	3
1	0	1	1	1	1	0	1	1	1
1	0	1	1	1	1	0	1	1	1
1	0	1	1	1	1	0	3	3	3
3	1	1	1	1	2	1	4	3	3
2	1	1	2	3	1	1	4	4	2
1	1	1	1	1	1	1	1	4	4
1	0	1	1	1	1	0	1	1	1
1	0	1	1	1	1	0	1	1	1
1	0	2	1	3	1	1	1	3	1
1	0	1	1	1	1	0	1	1	1
1	0	4	1	1	1	0	1	1	1
1	0	1	1	1	1	0	1	1	1
1	0	1	1	1	1	0	1	1	1
1	0	1	1	1	1	0	2	2	2
1	0	1	1	1	1	0	2	2	2
1	0	1	1	1	1	0	1	1	1
1	0	3	1	1	1	0	1	1	1
1	0	4	1	1	1	0	3	3	3
1	0	1	1	1	1	0	1	1	1
3	0	4	1	1	1	0	3	3	3
1	0	1	1	1	1	0	2	1	2

1	1	4	1	1	1	0	3	3	3
1	1	4	1	1	1	0	3	3	3
1	0	2	1	1	1	0	3	1	1
1	0	1	1	1	1	0	2	1	3
1	0	1	1	1	1	0	1	1	1
1	0	1	1	1	4	0	4	4	4
1	0	1	1	1	1	0	3	3	3
1	1	1	1	1	1	0	3	1	1
4	1	1	1	1	1	0	4	4	4
1	1	1	1	1	1	0	4	4	4
1	0	1	1	1	1	0	4	2	1
1	0	1	1	1	1	0	2	1	1
2	1	2	3	4	4	0	1	2	3
	1	1	1	1	1	0	3	1	3
	1	1	1	1	1	0	3	1	3
1	0	1	1	1	1	0	1	1	1
1	0	1	1	1	1	0	1	1	1
1	0	3	2	1	1	1	1	1	1
1	0	1	1	1	1	0	1	1	1
1	0	1	1	1	1	0	1	1	1
1	0	1	1	1	1	0	1	1	1
1	0	1	1	1	1	0	1	1	1
1	0	1	1	1	1	0	1	1	1
1	0	1	1	1	1	0	1	1	1
1	1	4	4	1	4	1	4	4	3
1	0	1	1	1	1	0	1	1	1
1	0	1	1	1	1	0	1	1	1
1	0	1	1	1	1	0	1	1	1
2	1	1	1	1	3	0	2	2	2
1	1	1	1	1	1	0	2	1	1
1	1	1	1	1	3	1	1	1	1
4	1	1	1	1	4	1	1	1	1
1	0	1	1	1	1	0	3	1	1
1	0	4	1	1	1	1	1	2	1
1	0	1	1	1	1	0	1	1	1
1	1	1	1	1	2	1	1	1	1
1	0	1	1	1	1	0	1	1	1
1	0	1	1	1	1	0	1	1	1
1	1	1	1	1	1	0	1	1	1
1	1	3	1	1	1	0	3	3	1
3	1	3	1	2	4	1	3	1	3
1	1	1	1	1	3	1	2	1	1
1	1	1	1	1	3	1	3	1	1
1	1	1	1	1	1	0	1	1	1
1	1	1	1	1	1	0	1	1	1
1	1	1	1	1	1	0	1	1	1
1	0	4	1	1	4	1	2	3	1

1	0	4	1	1	4	1	2	3	1
1	0	1	1	1	1	0	1	1	1
1	0	1	1	1	1	0	3	3	3
1	1	1	2	1	1	1	2	2	3
1	0	1	1	1	1	0	1	1	1
1	0	1	4	1	1	0	1	1	1
1	0	1	1	1	1	0	1	1	1
1	0	1	1	1	1	0	1	1	1
1	0	1	1	4	1	1	4	4	4
1	0	1	1	1	1	0	1	1	1
1	0	1	4	1	1	0	1	1	1
1	0	1	3	1	3	0	1	1	1
1	0	4	1	1	1	0	1	1	1
1	0	1	1	1	1	0	1	1	1
4	1	4	4	1	4	1	4	4	4
1	0	1	1	1	1	0	1	1	1
1	0	1	1	1	1	0	1	1	1
1	0	1	1	1	1	0	1	1	1
1	1	1	1	1	1	0	1	1	1
1	1	1	2	3	4	1	4	1	4
1	1	1	1	1	1	0	1	1	1
1	0	1	1	1	1	0	1	1	1
1	0	1	1	1	1	0	1	1	1
1	0	4		1	1	0	1	1	1
4	0	4	4	4	4	0	4	4	4
1	0	4	1	1	1	0	3	3	3
1	0	1	1	1	1	0	1	1	1
1	0	1	1	1	1	0	1	1	1
4	1	2	2	2	1	1	2	1	2
1	1	2	2	1	2	1	2	3	4
1	0	1	1	1	1	0	1	2	4
1	0	1	1	1	1	0	1	1	1
1	1	1	1	1	3	1	3	1	3
1	0	4	1	1	1	0	2	3	1
1	1	1	1	1	1	0	2	1	2
1	0	1	1	1	3	1	1	1	1
1	1	2	1	1	1	1	1	1	1
1	0	1	1	1	1	0	1	1	1
1	0	1	1	1	1	0	1	1	1
1	0	1	1	1	1	0	4	3	4
1	0	1	1	1	1	0	1	1	1
1	0	1	1	1	1	0	1	1	1
1	0	1	1	1	1	0	1	1	1
3	1	1	1	1	1	0	1	1	1
1	0	3		1	1	0	1	1	1
1	0	1	1	1	1	0	1	1	1
1	0	4	2	3	3	1	1	1	1

1	0	1	1	1	1	0	1	1	1
1	1	1	1	1	1	0	1	1	1
1	0	1	1	1	1	0	1	1	1
1	0	3	1	1	1	0	1	1	1
1	1	1	1	1	1	0	1	1	1
1	1	1	1	1	1	0	3	1	3
1	0	1	1	1	1	0	1	1	1
4	1	1	1	1	1	0	1	2	3
1	0	1	1	1	1	0	1	1	1
1	0	1	1			0	2	2	2
3	1	3	3	3	3	1	3	3	3
1	1	1	1	1	3	1	3	2	3
1	0	1	1	1	1	0	1	1	1
1	0	1	1	1	1	0	1	1	1
1	0	4	1	1	1	0	1	1	1
1	1	1	1	1	1	0	1	1	1
1	0	1	1	1	1	0	1	1	1
2	0	3	1	1	3	0	3	3	3
1	0	1	1	1	1	0	1	1	1
1	0	1	1	1	1	0	1	1	1
1	0	4	1	1	1	0	1	1	1
1	0	1	1	1	1	0	1	1	1
1	0	1	1	1	1	0	1	1	1
1	0		1	1	1	0	1	1	1
1	0	1	1	1	1	0	1	1	1
3	0	2	3	2	1	0	1	1	3
3	0	1	1	1	1	0	1	1	1
1	0	1	1	1	1	0	1	1	1
1	0	1	1	1	1	0	1	2	3
1	0	1		1	1	0	1	1	1
1	0	1	1			0	1	1	1
3	0	1	1			0	3	3	3
1	0	1	1	1	1	0	1	1	1
1	0	1	1	1	1	0	1	1	1
1	1	1	1	1	1	0	1	1	1
1	1	1	1	1		0	1	1	1
1	1	1	1	1	1	0	1	1	1
1	0	1	1	1	1	0	1	1	1
1	0	4	1	1	1	0	1	1	1
1	0	1	1	1	1	0	1	1	1
1	0	1	1	1	1	0	3	1	1
1	0	1	1	1	2	1	1	1	1
1	0	4	1	2	4	0	1	1	1
1	0	1	1	1	1	0	1	2	1
3	0	4	1	3	1	1	3	2	3
1	0	1	1	1	1	0	1	1	1
1	0	1	1			0	1	1	1

1	0	1	1	1	1	0	1	1	1
1	0	1	1	1	3	1	3	3	3
1	0	1	1	1	1	0	1	1	1
1	0	4	1	1	1	0	1	1	1
1	0	1	3	1	1	0	3	1	3
2	1	1	1	1	1	0	1	1	1
1	1	4	4	1	1	0	2	1	2
1	0	1	1		1	0	1	1	1
1	0	1	1	1	1	0	1	1	1
1	0	1	1	1	1	0	1	1	1
1	0	1		1	2	1	1	1	1
1	0	1	1	1	1	0	1	1	1
1	0	1	1	1	1	0	1	1	1
1	0	1	1			0	1	1	1
1	0	1	1	1	1	0	1	1	1
1	1	4	1	1	1	1	1	1	1
1	0	1	1	1	1	0	1	1	1
1	0	1	1	1	1	0	1	1	1
1	0	1	1	1	1	0	1	1	1
1	0	4		1	1	0	1	1	1
1	0	4		1	1	0	1	1	1
1	0	1	1	1	1	0	1	1	1
1	1	1	1	1	1	0	2	1	1
1	0	1	1	1	1	0	1	1	1
1	0	1	1	1	1	0	1	1	1
1	0	1	1	1	1	0	1	1	1
1	0	4	1	1	1	0	1	1	1
1	0	1	1	1	1	0	1	1	1
1	1	4	1	1	1	1	1	1	1
1	0	1	1	1	1	0	1	1	1
1	0	1	1	1	1	0	4	4	4
1	0	1	1	1	1	0	1	1	1
1	0	1	1	1	4	1	1	1	1
1	1	4	4	1	4	1	4	4	4
1	0	1	1	1	1	0	3	3	4
1	0	1	1	1	1	0	1	1	1
1	0	1	1	1	1	0	1	1	1
1	0	1	1	1	1	0	1	1	1
1	0	1	1	2	1	1	1	4	1
1	0	1	1	1	1	0	1	1	1
1	0	1	1	1	1	0	1	1	1
1	0	1	1	1	1	0	1	1	1
3	1	1	1	1	1	1	1	1	1
1	0	1	1	1	1	0	1	1	1
1	0	1	1	1	1	0	1	1	1
1	0	1	1	4	1	0	3	4	1
1	0	1		1	1	0	1	1	1

1	0	1	1	1	1	0	1	1	1
1	1	4	1	1	4	1	1	1	1
1	0	4	1	1	1	0	1	1	1
1	0		1	1	1	0	1	1	1
1	0	1	1	1	1	0	1	1	1
1	0	1	1	1	1	0	1	1	1
1	0	1	1	1	2	1	1	1	1
1	0	1		1	1	0	1	1	1
1	0	1	1	1	1	0	1	3	1
1	0	1	1	1	1	0	1	1	1
3	1	1	1	1	3	1	3	2	2
1	0	1	1	1	1	0	1	1	1
1	0	1	1	1	4	1	1	1	1
1	0	1	1	1	1	0	1	1	1
1	0	1	1	1	1	0	1	1	1
1	0	4	1	1	1	0	1	3	1
1	0	1	1	1	1	0	1	1	1
1	1	1	1	1	1	0	1	1	1
1	0	1	1			0	1	1	1
1	0	4	4	1	1	0	1	1	1
1	1	4	1	1	1	0	4	4	4
1	1	1	1	1	2	1	1	1	1
1	0	2	1	1	1	0	1	1	1
1	0	2	1	1	1	0	1	1	1
1	0	1	1	1	1	0	1	1	1
1	1	1	1	1	1	0	1	1	1
1	0	1	1	1	1	0	1	1	1
1	0	1	1	1	1	0	1	1	1
1	0	1	1	1	1	0	1	1	1
1	0	1	1	1	1	0	1	1	1
1	0	1	1	1	1	0	1	1	1
1	0	1	1	1	1	0	1	1	1
1	0	1	1	1	1	0	1	1	1
1	0	3	1	1	1	0	1	1	1
1	0	4	1	1	1	0	1	1	1
1	0	4	1	1	1	0	1	1	1
1	0	1	1	1	1	0	1	1	1
1	0	1	1	1	1	0	1	1	1
1	0	1	1	1	1	0	1	1	1
1	0	1	1	1	1	0	1	1	1
1	0	1	1	1	1	0	1	1	1
1	0	1	1	1	1	0	1	1	1
1	0	1	1	1	1	0	1	1	1
1	0	1	1	1	1	0	3	3	1
1	0	1	1	1	1	0	1	2	1
1	0	1	1	1	1	0	1	1	1
1	0	1	1	1	1	0	1	1	1

1	1	1	1	1	1	0	1	1	1
1	0	4	1	1	1	0	1	1	1
1	1	1	1	1	3	0	3	1	3
1	1	1	1	1	1	0	1	1	1
1	0	1	1	1	1	0	1	1	1
1	0	1	1	1	1	0	1	1	1
1	0	1	1	1	1	0	4	1	1
1	0	1	1	1	1	0	1	1	1
1	0	1	1	1	1	0	1	1	1
1	0	1	1	1	1	0	1	1	1
1	1	1	1	1	1	0	1	1	1
1	0	1	1	1	1	0	4	4	4
1	0	4	1	1	1	0	4	1	1
1	0	1	1	1	3	1	1	1	1
1	0	1	1	1	1	0	2	1	1
2	1	4	1	1	2	1	1	2	1
1	0	1	1	1	1	0	1	1	1
3	1	1	1	3	1	1	3	3	3
1	0	1	1	1	1	0	1	1	1
1	0	1	1	1	4	1	1	1	1
1	1	4	4	1	4	1	1	1	1

1	0	1	1	1	1	0	1	1	1
1	0	3	1	1	1	0	1	1	1
1	1	1	1	1	4	1	1	1	1
1	0	4	1	1	1	0	1	1	1
1	1	4	4	1	1	0	1	3	1
1	0	4	2	1	1	0	2	1	3
1	0	1	1	1	1	0	1	1	1
1	0	1	1	1	1	0	1	1	1
1	0	1	1	1	1	0	1	1	1
1	0	4	1	1	1	1	1	1	1
1	1	4	1	1	1	0	4	1	1

1	0	4	1	1	1	0	1	1	1
1	0	1	1	1	1	0	2	1	2
1	0	1	1	1	1	0	1	1	1
4	1	4	1	1	4	1	1	1	1
1	0	1	1	1	2	0	1	1	1
4	1	4	4	4	4	1	4	4	4
1	1	1	1	1	1	0	1	1	1
1	0	1	1	1	1	0	1	1	1
1	0	1	1	1	3	0	1	1	1
1	0	1	1	1	1	0	1	1	1
1	0	1	1	1		0	1	1	1
1	0	4	2	1	1	0	4	1	3
1	0	1	1	1	1	0	1	1	1

1	0	1	1	1	1	0	1	1	1
1	0	1	1	1	1	0	1	1	1
1	0	1	1	1	1	0	1	1	1
1	0	1	1	1	1	0	1	1	1
1	0	1	1	1	1	0	1	1	1
1	0	1	1	1	1	0	1	2	1
2	1	3	3	3	4	1	4	4	4
1	0	1	1	1	1	0	1	1	1
3	0	1	1	1	1	0	2	2	1
1	0	1	1	1	1	0	2	2	2
1	0	1				0	1	1	1
1	0	1	1	1	1	0	1	1	1
	0	1	1	1	1	0	1	1	1
1	0	1	1	1	1	0	1	1	1
1	0	1	1	1	1	0	1	1	1
1	0	1	2	1	3	1	3	2	1
1	0	1	1	1	1	0	2	2	2
1	0	1	1	1	1	0	1	1	1
1	0	1	1	4	1	0	2	2	3
1	0	1	1	1	1	0	1	1	1
1	0	1	1	1	1	0	1	1	1
1	0	3	1	1	1	0	1	1	1
1	0	1	1	1	1	0	1	1	1
1	0	1	1	1	1	0	1	1	1
1	0	1	1	1	1	0	1	1	1
1	0	1	1	1	1	0	2	1	1
4	1	4	1	1	1	1	1	1	1
1	0	1	1	1	1	0	1	1	1
1	0	1	1	1	1	0	1	1	1
1	0	1	1	1	1	0	1	1	1
1	0	1	1	1	1	0	1	1	1
1	1	1	1	1	3	1	3	3	3
1	0	1	1	1	1	0	1	1	1
1	0	3	1	1	1	0	2	2	1
1	0	1	1	1	1	0	1	1	1
1	1	1	1	1	3	1	4	4	1
1	0	4	1	1	1	0	1	1	1
1	0	4	1	1	1	0	1	1	1
1	0	1	1	1	1	0	1	1	1
1	0	4	1	1	1	0	1	1	1
1	0	1	1	1	1	0	1	1	1
1	0	1	1	1	1	0	1	1	1
1	0	1	1	1	1	1	1	1	1
1	0	1	1	1	1	0	1	1	1
1	0	1	1	1	1	0	1	1	1
1	0	1	1	1	1	0	1	1	1
1	0	3	3	2	2	0	1	1	1

1	0	3	3	2	2	0	1	1	1
1	0	4	4	1	1	0	1	1	1
3	0	4	4	1	4	1	1	1	1
1	0	4	1	1	1	0	2	1	1
1	0	1	1	1	4	0	1	1	1
4	0	1	1	2	1	0	3	3	3
3	0	4	1	4	4	0	4	4	4
1	0	2	1	1	1	0	1	1	1
1	0	1	1	1	1	0	1	1	1
1	0	1	4	1	1	0	3	3	3
1	0	1	1	1	1	0	2	1	1
1	0	1	1	1	1	0	2	1	1
1	0	1	1	1	1	0	1	1	1
1	0	2	1	1		0	1	1	1
3	0	1	3	4	4	0	4	4	4
1	0	1	2	1	1	1	2	3	3
1	0	1	1	1	1	0	1	1	1
1	0	1	1	1	1	0	1	1	1
1	0	1	1	1	1	0	3	2	1
1	0	1	1	1	1	0	1	1	1
1	1	1	1	1	1	0	1	1	1
1	0	1	1	1	1	0	1	1	1
1	0	3	1	1	1	0	1	3	3
1	0	1	1	1	1	0	1	1	1
1	1	3	1	3	4	1	4	4	4
1	0	4	1	1	3	1	1	1	1
1	0	1	1	1	1	0	2	2	2
1	0	1	1	1	1	0	2	1	1
1	0	1	1	1	1	0	2	1	1
1		1	1	1	1	0	1	1	1
1	0	1	1	1	1	0	1	2	1
1	1	3	1	1	4	1	1	3	1
3	0	1	1	1	3	1	1	1	1
1	0	2	1	1	1	0	1	1	1
1	0	1	1	1	1	0	1	1	1
4	1	4		4	4	1	4	4	4
1	1	1	1	1	1	0	3	3	1
1	0	1	1	2	1	0	1	1	1
1	1	3	4	4	1	1	1	1	1
1	1	1	1	3	3	1	3	3	3
1	0	1	1	1	1	0	1	1	1
1	0	1	1		2	0	1	1	1
3	0	1	3			0	1	1	1
1	0	1	1	3	3	0	3	3	1
1	0	1	1	3	3	0	3	3	1
1	0	1	1			0	1	1	1
1	0	3	1	1	1	0	1	1	1

1	0	1	1	1	1	0	1	1	1
1	0	1	1	1	1	0	1	1	1
3	1	1	1	1	1	0	2	2	1
2	1	1	1	1	1	0	1	1	1
3	1	1	1	1	1	1	1	1	1
3	1	1	1	1	1	1	1	1	1
1	0	2	1	1	1	0	4	3	3
1	0	2	1	1	1	0	4	3	3
1	0	2	1	1	1	0	4	3	3
1	0	1	1	1	1	0	1	1	1
1	0	1	1	1	1	0	1	1	1
1	0	4	1	1	1	0	1	1	1
1	1	1	1	4	3	1	3	3	3
1	0	1	1	1	1	0	1	1	1
2	0	1	1	1	1	0	1	1	1
1	0	1	1	1	1	0	1	1	1
3	1	1	1	1	1	0	1	1	1
1	0	1	1	1	1	0	1	1	1
1	0	1	1	1	1	0	1	1	1
1	1	1	1	1	1	0	1	1	1
4	0	4	4	4	4	0	1	1	1
1	0	1	1	1	1	0	1	1	1
1	0	1	1	1	1	0	1	1	1
2	0	2	2	2	2	1	2	2	2
1	0	1	1	1	1	0	1	1	1
1	0	1	1	1	1	0	1	1	1
1		1	1	1	1	0	1	1	1
1	0	1	1	1	1	0	1	1	1
2	0	1	1	1	1	0	1	2	1
2	1	1	2	2	2	0	1	1	1
1	0	1	1	1	1	0	1	1	1
1	0	1	1	1	1	0	1	1	1
4	1	1	1	1	1	0	1	1	1
1	1	4	4	1	1	1	1	1	3
1	0	1	2	1	1	0	1	1	1
1	1	1	1	1	1	0	1	1	1
1	0	1	1	1	1	0	1	1	1
1	1	1	1	1	1	1	1	1	2
3	0	3	3	3	3	0	3	3	3
1				1	1		1	1	1
3	0	1	1	1	1	0	2	2	1
1	1	1	1	1	1	1	2	2	2
1	1	1	1	1	1	0	2	1	1
1	1	1	1	1	1	0	1	1	1
3	1	1	1	1	1	0	1	1	1
1	0	1	1	1	1	0	1	1	1

1	0	1	1	1	1	0	1	1	1
1	0	1	1	1	3	1	1	1	1
1	0	1	1	1	1	0	1	2	2
4		4	4	4	4	0	4	4	4
1		1	1	1	1	0	1	1	1
1		1	1	1	1		1	2	1
1	0	1	1	1	1	0	1	1	1
1	0	1	1	1	1	0	1	1	1
1	0	1	1	1	1	0	1	1	1
1	1	1	3	1	1	0	3	3	3
1	1	3	1	1	1	0	2	2	2
1	0	1	1	1	1	0	1	1	1
2	0	1	1	1	1	0	1	1	1
1	0	1	1	1	1	0	1	1	1
1		1	1	1	1	0	1	1	1
3	1	1	1	1	1	1	1	1	1
2	1	1	1	1	1	0	2	2	2
1	0	1	1	1	2	1	2	1	1
1	0	1	1	1	1	0	1	2	1
		1	1	1	1		1	1	1
1	0	1	1	1	1	0	1	1	3
1	0	1	1	1	1	0	1	1	1
1	1	1	1	1	2	1	3	3	3
1	0	1	1	1	1	0	1	1	1
1	0	1	1	1	1	0	1	1	1
2	0	1	1	1	1	0	1	1	1
1	0	1	1	1	1	0	1	1	1
1	0	1	1	1	1	0	1	1	1
1	0	1	1	1	1	0	1	1	1
3	0	1	1	1	1	0	1	1	1
1	0	3	1	1	1	0	1	1	1
1	0	1	1	1	1	0	2	2	2
2		1	1	2	1		1	1	1
1	0	1	1	1	1	0	1	1	1
1	0	1	1	2	1	0	1	1	1
4	1	1	1	1	1	1	1	1	1
1	0	1	1	1	1	0	2	2	2
1	0	1	1	1	1	0	1	1	1
1	0	2	1	1	1	0	1	1	1
1	1	1	1	1	1	0	1	1	1
1	1	2	2	2	2	1	1	1	1
1	1	1	1	1	1	1	1	1	1
1	0	1	1	3	3	0	1	1	1
1	0	1	4	3	1	1	1	4	4
1	0	1	1	1	1	0	1	1	1
1	1	4	1	1	4	1	4	4	4
1	1	1	1	1	1	0	4	1	1

1	0	1	1	1	1	0	1	1	1
3	0	4	3	4	4	0	4	1	4
4	1	1	1	1	4	1	4	4	4
1	0	1	1	1	1	0	1	1	1
1	0	1	1	1	1	0	1	1	1
3	1	1	1	1	1	0	4	4	3
1	1	1	1	1	1	0	3	3	1
4	0	1	1	1	1	0	1	1	1
4	1	1	1	4	4	1	4	4	4
1	0	1	1	1	1	0	1	1	1
1	1	1	1	3	4	1	1	1	1
1	1	1	1	3	4	1	1	1	1
1	1	4	1	1	1	0	4	4	4
1	1	4	1	1	1	0	4	4	4
1	0	1	1	1	3	1	4	4	4
4	1	4	4	4	4	1	4	4	4
1	1	1	1	1	4	1	1	4	4
1	1	1	1	1	4	1	1	2	2
1	0	1	1	1	1	0	1	1	1
1	0	1	4	1	4	0	4	3	2
1	1	4	4	4	4	1	4	4	4
1	1	4	1	1	1	1	4	4	4
2	0	1	1	1	3	0	3	1	3
1	1	1	1	1	1	0	2	1	1
1	0	4	1	1	1	0	3	1	1
2	1	4	1	1	4	1	4	4	4
1	0	1	1	1	1	0	1	1	1
1	0	4	1	1	4	1	1	1	1
4	1	4	4	4	4	1	4	4	4
4	1	4	4	4	4	1	4	4	4
1	1	1	1	1	1	0	2	2	2
1	0	1	1	1	1	0	1	1	1
1	0	1	1	1	4	1	1	1	1
1	0	2	1	1	4	1	4	4	4
1	0	1	1	1	3	0	1	1	1
4	1	1	1	1	3	0	3	3	3
4	0	4	1	4	4	0	4	4	4
1	1	1	1	1	1	0	1	1	1
4	1	1	1	1	4	1	1	3	3
1	0	3	3	1	1	0	1	1	1
1	0	3	3	1	1	0	1	1	1
1	0	1	1	1	1	0	1	1	1
1	0	1	1	1	1	0	2	2	2
1	0	1	1	1	1	0	1	1	1
3	0	1	1	1	3	0	4	1	1
1	0	1	1	1	1	0	2	2	2
1	0		1	1	1	0	1	1	2

1	0	1	1	1	1	0	1	1	1
1	0	1	1	1	1	0	1	1	1
1	0	1	1	1	1	0	2	2	2
1	0	1	1	1	1	0	1	1	1
1	0	1	1	1	1	0	1	1	1
1	0	1	1	1	1	0	1	1	1
1	0	2	1	1	1	0	1	1	1
1	0	1	1	1	1	0	1	1	1
1	0	1	1	1	1	0	1	1	1
1	0	1	1	1	1	0	1	3	1
1	0	1	1	1	1	0	1	3	1
1	0	1	1	1	1	0	1	1	1
1	0	1	1	1	1	0	1	1	1
4	1	1	1	4	3	1	1	4	4
1	0	1	1	1	1	0	3	3	3
1	0	1	1	1	1	0	3	3	3
1	0	1	1	2	1	0	2	2	2
1	0	1	1	1	1	0	1	1	1
1	0	2	1	1	1	0	1	1	1
1	0	1	1	1	1	0	1	1	1
1	0	1	1	1	1	0	1	1	1
1	0	1	1	1	1	0	1	1	1
1	0	1	1	1	1	0	2	2	2
1	0	1	1	1	1	0	3	3	3
1	0	1	1	1	1	0	1	1	1
1	0	1	1	1	1	0	1	1	1
1	0	1	1	1	1	0	3	3	3
1	0	1	1	1	1	0	1	1	1
1	0	1	1	1	1	0	2	1	1
1	0	1	1	1	1	0	1	1	1
1	0	1	1	1	1	0	1	1	1
1	0	1	1	1	1	0	1	1	1
1	0	1	1	1	1	0	1	1	1
1	0	1	1	1	1	0	1	1	1
4	0	1	1	4	4	0	4	4	4
1	0	1	1	1	1	0	1	1	1
1	0	1	1	1	1	0	3	3	1
1	0	1	1	1	1	0	3	1	1
1	0	1	1	1	1	0	1	1	1
1	0	1	1	1	1	0	1	1	1
1	1	1	1	1	1	0	1	1	1
1	0	1	1			0	1	1	1
1	1	1	1	1	2	1	1	1	1
1	0	4	1	3	1	0	1	1	1
1	0	1	1	1	1	0	1	1	1
1	0	1	1	1	1	0	1	1	1
1	0	1	1	1	1	0	1	1	1

3	1	1	1	1	1	0	1	3	3
1	0	4	1	1	4	0	1	1	1
1	0	3	1	1	1	0	1	1	1
1	0	4	1	1	1	0	1	1	1
1	1	1	1	1	4	1	4	4	4
2	1	3	1	1	3	0	4	4	2
1	0	1	1	1	1	0	1	1	1
1	0	1	1	1	1	0	1	2	2
4	1	4	1	1	4	1	1	1	1
1	1	1	1	1	2	1	1	3	1
4	1	3	4	4	4	1	4	4	1
1	0	3	1	1	1	0	1	1	1
1	1	4	1	1	1	0	1	1	1
4	1	1	1	3	4	1	1	1	1
1	1	4	1	1	4	1	4	4	4
1	0	1	1	1	1	0	1	1	1
1	0	1	1	1	1	0	1	1	1
4	1	3	3	4	4	1	4	4	4
1	0	1	1	1	1	0	1	1	1
1	0	1	1	1	1	0	1	1	1
1	0	1	1	1	4	1	1	1	1
1	0	1	1	1	1	0	1	1	1
1	0	1	1	1	1	0	1	1	1
1	0	1	1	1	1	0	1	1	1
1	0	1	1	1	1	0	1	1	1
1	0	1	1	1	1	0	1	1	1
1	1	1	1	1	3	1	3	1	1
1	1	1	3	3	4	1	3	3	3
2	1	1	3	1	4	1	1	1	1
2	1	1	3	1	4	1	1	1	1
3	1	1	1	2	3	1	3	3	3
3	1	1	1	2	3	1	3	3	3
1	0	1	1	2	4	1	1	3	1
1	0	1	1	1	1	0	1	1	1
1	0	1	1	1	1	0	1	1	1
1	0	1	1	1	1	0	1	1	1
3	1	3	3	3	3	1	3	3	3
1	0	3	1	1	1	0	1	1	1
1	0	1	1	1	1	0	1	1	1
3	1	1	1	1	4	1	1	1	1
1	1	1	1	1	1	0	1	1	1
1	0	1	1	1	1	0	1	1	1
1	1	1	1	1	1	0	1	1	1
1	0	1	2	2	1	0	1	1	1
1	0	1	1	1	1	0	1	1	1
1	0	1	1	1	1	0	1	1	1
1	0	1	1	1	1	0	1	1	1

1	1	2	1	1	1	0	4	4	4
1	1	3	1	1	1	0	3	3	3
4	1	4	4	4	4	1	4	4	4
1	0	1	1	1	2	0	1	2	2
1	0	1	1	1	1	0	1	1	1
1	1	3	1	1	1	1	1	1	1
1	0	1	1	1	1	0	1	1	1
1	0	1	1	1	2	1	2	1	2
3	1	1	1	1	1	0	3	3	1
3	1	1	1	1	1	0	1	1	1
1	0	1	1	1	1	0	1	1	1
1	0	1	1	1	1	0	3	1	3
1	0	1	1	1	1	0	1	1	1
1	0	1	1	1	1	0	1	1	1
2	1	1	1	1	1	1	1	1	1
1	0	1	1	1	2	0	1	1	1
1	0	1	1	1	1	0	1	1	1
3	1	1	3	1	1	1	1	3	2
1	0	1	1	1	1	0	1	1	1
1	0	1	1	1	1	0	1	1	1
1	0	4	1	1	1	0	1	1	1
1	0	1		1	1	0	1	1	1
1	0	2	1	1	2	0	1	1	1
1	0	1	1	1	1	0	1	1	1
3	1	1	1	1	4	0	1	1	1
1	0	1	1	1	1	0	1	1	1
1	0	1	1	1	1	0	1	1	1
1	0	1	1	1	1	0	1	1	1
1	0	4	1	1	1	0	1	1	1
1	0	1	1	1	1	0	1	1	1
1	0	3	1	1	1	0	1	1	1
1	0	1	1	1	1	0	1	1	1
1	0	1	1	1	1	0	1	1	1
1	0	1	1	1	1	0	1	1	1
3	0	3	1	1	3	0	1	3	1
1	0	1	1	1	1	0	1	1	1
1	0	1	1		1	0	1	1	1
1	0	1	1	1	1	0	1	1	1
1	0	1	1	1	1	0	1	1	1
1	0	1	3	2	1	0	1	1	1
4	0	4	4	1	4	0	4	4	4
1	0	1	1	1	1	0	1	1	1
1	0	1	1	1	1	0	1	1	1
1	0	1	1	1	1	0	1	1	1
1	0	1	1	1	1	0	1	1	1
1	0	1	1	1	1	0	1	1	1
1	0	1	1	1	4	1	1	1	1

3	1	1	3	4	4	1	4	4	4
1	0	1	1	1	1	0	1	1	1
1	0	1	1	1	1	0	1	1	1
1	0	1	1	1	1	0	1	1	1
1	0	1	1	1	1	0	1	1	1
1	0	1	1	1	1	0	1	1	1
1	0	1	1	1	1	0	1	1	1
1	0	1	1	1	4	1	1	1	1
1	0	1	1	1	1	0	1	1	1
1	0	1	1	1	1	0	1	1	1
1	0	1	1	1	1	0	1	1	1
1	0	1	1	1	1	0	1	1	1
1	0	1	1	1	1	0	1	1	1
1	0	1	1	1	1	0	1	1	1
1	0	1	1	1	1	0	4	1	4
1	0	4		1	1	0	3	3	3
1	0	1	1	1	1	0	1	1	1
1	0	1	1	1	1	0	1	1	1
1	0	1	1	1	1	0	1	1	1
1	0	1	1	1	1	0	1	1	1
1	0	1	1	1	1	0	1	1	1
1	0	1	1	1	1	0	1	1	1
1	0	1	1	1	1	0	1	1	1
1	0	1	1	1	1	0	1	1	1
1	0	1	1	1	1	0	1	1	1
1	0	1	1	1	1	0	2	1	1
1	0	1	1	1	1	0	1	1	1
1	0	1	1	1	1	0	2	2	2
1	1	1	1	1	1	0	1	1	1
1	0	1	1	1	1	0	1	1	1
1	0	1	1	1	1	0	1	2	2
1	0	1	1	1	1	0	1	1	1
1	0	1	1	1	1	0	1	1	1
4	1	1	1	1	2	1	1	1	2
1	0	1	1	1	1	0	3	1	1
1	0	1	1	1	1	0	3	1	1
2	1	4	1	4	3	1	4	4	3
1	0	2	1	1	1	0	1	1	1
1	0	1	1	1	1	0	3	3	3
1	1	1	2	2	3	1	3	2	3
1	0	1	1	1	1	0	1	1	1
2	0	1	1	1	3	1	2	2	2
1	0	1	1	1	1	0	1	1	2
3	0	1	1	1	1	0	2	1	3
1	0	1	1	1	1	0	3	1	1
1	0	1	1	1	1	0	4	2	1
1	0	1	1	1	1	0	1	1	1

4	1	4	3	4	4	1	4	4	4
1	0	1	1	1	1	0	1	1	1
1	0	4	1	1	1	0	1	1	1
1	0	1	1	1	1	0	3	3	3
1	0	1	1	1	1	0	3	3	3
1	0	1	1	1	1	0	1	1	1
1	0	1	1	1	1	0	1	1	1
1	0	2	1	1	1	0	2	1	2
1	1	1	1	1	1	1	1	1	1
1	1	1	1	1	1	0	3	3	1
1	0	1	1	1	1	0	1	1	1
1	0	4	1	1	1	0	1	1	1
1	0	1	1	1	1	0	3	1	1
1	0	1	1	1	1	0	1	3	1
1	0	1	1	1	1	0	1	1	1
1	0	1	1	1	1	0	1	1	1
1	0	1	1	1	1	0	1	1	1
1	0	1	1	1	1	0	2	1	1
1	0	1	1	1	1	0	1	1	1
1	1	4	1	1	1	0	1	1	1
1	0	1	1	1	1	0	1	1	1
1	0	1	1	1	2	1	1	1	1
1	0	1	1	1	1	0	1	1	1
1	0	1	1	1	1	0	1	1	1
1	0	1	1	1	1	0	1	1	1
1	1	1	1	1	1	0	1	1	1
1	0	3	1	1	1	0	1	1	1
1	0	1	1	1	1	0	1	1	1
1	0	1	1	1	1	0	1	1	1
1	1	1	1	1	1	0	1	1	1
1	0	4	1	1	4	0	4	1	1
1	0	3	1	1	1	0	1	1	1
1	0	1	1	1	1	0	1	1	1
2	1	1	1	1	1	0	1	1	1
1	0	1	1	1	1	0	1	1	1
1	1	1	1	1	1	0	1	1	1
1	0	1	1	1	1	0	1	1	1
1	0	1	1	1	1	0	1	1	1
1	0	3	1	1	1	0	1	1	1
1	0	1	1	1	1	0	1	1	1
1	0	1	1	1	1	0	1	1	1
1	1	1	1	1	1	0	1	1	1
4	0	4	1	1	4	0	4	4	4
1	1	2	2	1	4	1	1	1	1
1	0	1	1	1	1	0	1	1	1
1	0	1	1	1	1	0	1	1	1

1	0	4		1	3	1	1	1	1
1	0	1	1	1	1	0	1	1	1
1	0	2	1	1	1	0	1	1	2
1	0	1	1	1	1	0	1	1	1
1	0	1	1	1	1	0	1	1	1
1	0	1	1	1	1	0	1	1	3
1	0	1	1	1	1	0	1	1	3
1	0	1	1	1	1	0	1	1	1
1	0	4	1	1	1	0	1	1	1
1	0	1	1	1	1	0	1	1	1
1	0	1	1	1	1	0	1	3	1
1	0	1	1	1	1	0	3	1	1
1	1	1	1	1	1	0	1	2	2
1	1	1	1	1	1	0	3	1	4
1	0	1	1	1	1	0	1	1	1
1	0	1	1	1	1	0	1	1	1
1	0	1	4	1	1	0	1	3	1
1	1	1	1	1	1	0	1	1	1
1	0	1	1	1	1	0	1	1	1
2	1	3	1	3	4	1	3	3	2
1	0	1	1	1	1	0	1	1	1
1	0	1	1	1	1	0	1	1	1
1	0	1	1	1	1	0	1	1	1
1	0	1	1	1	1	0	1	1	1
1	0	1	1	1	1	0	1	3	3
3	1	4	1	1	1	0	3	3	3
1	0	1	1	1	1	0	1	1	1
1	0	1	1	1	1	0	1	1	1
1	0	1	1		1	0	1	1	1
1	0	1	1	1	1	0	1	1	1
1	0	1	1	1	1	0	1	1	1
1	0	1	1	1	1	0	1	1	1
1	0	1	1	1	1	0	1	1	1
3	0	3	3	3	3	0	1	1	1
1	0	1	1	1	1	0	1	1	1
1	0	1	1	1	1	0	1	1	1
1	0	1	1	1	1	0	1	1	1
1	0	1	1	1	1	0	1	1	1
	0	1	1	1	1	0	2	3	1
1	0	1	1	1	1	0	3	1	1
1	0	1	1	1	1	0	1	1	1
1	1	1	1	1	1	0	2	2	1
3	1	2	1	1	1	1	1	2	1
1	0	1	1	1	1	0	1	1	1
1	0	1	1	1	1	0	1	1	1
1	0	1	1	1	1	0	1	1	1
3	1	1	1	3	3	1	3	3	1

1	0	4	4	4	4	1	2	4	2
1	0	1	1	1	1	0	1	1	1
1	0	1	1	1	1	0	1	1	1
1	0	1	1	1	1	0	1	1	1
1	0	4	1	1	4	0	1	1	1
1	1	1	1	2	2	1	3	3	3
1	0	1	1	1	1	0	1	1	1
1	0	4	3	1	4	1	1	1	1
3	0	3	3	3	1	0	3	3	3
1	1	1	1	1	3	1	3	3	3
1	0	3	1	1	1	0	1	1	1
1	0	4	1	1	1	0	1	1	1
1	0	1	1	2	1	0	3	3	3
1	0	1	1	1	1	0	1	1	1
3	1	2	2	1	2	1	4	2	2
4	1	3	1	1	3	1	1	3	1
2	0	1	1	1	1	0	1	1	1
1	0	1	1	1	1	0	1	1	1
1	0	1	1	1	1	0	3	1	1
1	0	4	1	1	1	0	1	3	2
1	0	1	1	1	1	0	1	1	1
1	0	3	1	1	1	0	1	1	1
1	0	1	4	1	1	0	1	1	1
1	0	1	1	1	1	0	2	1	3
2	1	1	4	1	4	1	1	4	4
1	0	1	1	1	1	0	1	1	1
1	0	1	1	1	1	0	1	1	1
1	0	1	1	1	1	0	3	3	3
1	0	1	1	1	1	0	1	1	1
4	1	4	1	4	4	1	1	4	1
1	0	1	1	1	1	0	1	1	1
1	1	1	1	1	1	0	2	2	1
1	0	1	1	1	1	0	3	1	1
3	0	4	1	1	1	0	1	1	1
1	0	3	1	1	1	0	3	1	1
1	0	1	2	1	1	0	1	1	1
4	1	2	2	4	4	1	4	4	4
1	0	1	1	1	1	0	1	1	1
1	0	1	1	1	1	0	1	1	1
3	0	4	1	1	4	1	3	1	1
1	1	1	1	1	3	1	2	2	2
1	0	1	1	1	1	0	1	1	1
1		1	1	1	1		1	1	1
1		1	1	1	1		1	1	1

1	0	1	1	1	1	0	1	1	1
2	0	1	1	1	1	0	1	1	1
1	0	1	1	1	1	0	1	1	1
3	0	2	2	2	2	0	1	1	1
2	1	1	1	1	1	0	1	1	1
3	0	1	1	1	1	1	1	1	2
4	0	4	4	4	4	0	1	1	1
1	0	1	1	1	1	0	1	1	1
1	0	1	1	1	1	0	2	2	2
1	1	1	1	1	1	0	1	1	1
1	1	2	1	1	1	0	3	1	1
1	0	1	1	1	1	0	1	1	1
1	0	1	1	1	3	0	3	3	3
2		1	1	1	2		1	1	1
1		1	2	2	1		1	1	1
1	0	1	1	1	1	0	1	1	1
2	1	2	3	3	3	1	1	1	1
1	0	4	1	1	1	0	1	1	1
1	0	1	1	1	1	0	1	1	2
4	0	4	4	4	4	0	4	4	4
1	0	1	1	1	1	0	1	1	1
3	0	1	1	1	1	0	1	1	1
1	0	1	1	1	1	0	1	1	1
1		1	1	1	1		1	1	1
1	0	1	1	1	1	1	1	1	1
1	0	1	1	1	1	0	1	1	1
1	0	1	1	1	1	0	1	1	1
1	0	2	3	2	3	0	2	1	2
2	1	1	2	2	1	1	1	1	1
1	0	1	1	1	1	0	1	1	1
4	1	1	1	1	1	0	1	1	1
4	1	1	1	1	1	0	1	1	1
4	0	1	1	1	1	0	1	1	1
1	0	3	1	1	1	0	1	1	1
1	0	3	1	1	1	0	1	1	1
1	0	1	1	1	1	0	1	1	1
3	1	1	1	1	1	1	1	1	1
2	0	2	1	1	1	0	1	1	1
1	0	1	1	1	1	0	1	1	1
1	1	1	1	3	4	1	1	1	1
4	0	4	4	4	4	0	4	4	4
1	0	4	1	1	1	0	1	1	1
1	0	1	1	1	4	0	4	4	4
2	1	4	1	1	3	1	1	1	1
2	1	4	1	1	3	1	1	1	1
4	1	4	4	1	1	1	4	4	4

4	1	4	4	1	1	1	4	4	4
1	0	4	4	1	1	0	4	4	3
1	0	4	4	1	1	0	4	4	3
4	0	1	1	1	2	0	1	1	1
4	0	1	1	1	2	0	1	1	1
1	1	1	1	1	1	0	1	1	1
1	0	1	1	1	4	1	1	2	1
1	0	1	1	1	1	0	1	1	4
1	0	1	1	1	1	0	1	1	1
1	0	1	1	1	1	0	1	1	1
1	0	1	1	1	1	0	1	1	1
4	1	1	1	4	4	1	1	1	1
1	1	1	1	1	1	0	1	1	1
3	1	1	1	1	3	0	4	3	3
1	0	1	1	1	1	0	1	1	1
1	0	1	1	1	1	0	2	1	1
1	0	1	1	1	1	0	1	1	3
1	0	1	1	1	1	0	1	1	1
1	0	1	1	1	1	0	1	1	1
1	0	3	2	1	1	0	1	1	4
1	0	1	1	1	1	0	4	1	1
1	1	1	1	1	3	0	1	1	3
1	0	1	1	1	1	0	1	1	1
1	0	3	1	1	1	0	1	1	1
1	0	1	1	1	1	0	1	1	1
1	0	1	1	1	3	0	1	1	1
1	0	4	1	2	1	0	1	1	1
1	0	1	1	1	1	0	3	3	3
1	0	1	1	1	1	0	1	1	1
1	0	1	1	1	1	0	1	1	1
1	0	1	1	1	1	0	1	1	1
1	0	1	1	1	1	0	3	1	3
1	0	1	1	1	1	0	1	1	1
1	0	1	1	1	1	0	3	1	1
1	0	1	1	1	1	0	1	1	1
1	0	1	1	1	1	0	1	1	1
1	0		1	1	1	0	1	1	1
3	0	2	1	1	1	0	3	1	1
1	0	1	2	1	1	0	1	2	3
1	0	1	1	1	1	0	1	1	1
1	0	1	1	1	1	0	1	1	1
1	0	1	1	1	1	0	1	1	1
1	0	1	1	1	1	0	1	1	1
1	0	1	1	1	1	0	1	1	1
1	0	1	1	1	1	0	1	1	1
2	0	1	1	1	2	0	2	1	1
1	0	1	1	1	1	0	1	1	1

1	0	1	1	1	1	0	1	1	1
1	0	4	1	1	1	0	1	1	1
1	0	1	1	1	1	0	1	1	1
1	0	2	1	1	1	0	1	1	1
1	0	1	1	1	1	0	1	1	1
1	0	1	1	1	1	0	1	1	1
4	0	4	4	4	4	0	4	4	4
1	0	1	1	1	1	0	1	1	1
1	0	1	1	1	1	0	1	1	1
1	0	4	1	3	4	1	4	4	4
1	0	1	1	1	1	0	1	1	1
1	0	1	1	1	1	0	1	3	3
1	0	1	1	1	1	0	1	1	1
1	0	1	1	1	1	0	2	1	1
1	0	1	1	1	1	0	1	1	1
1	0	1	1	1	1	0	1	1	1
1	0	1	1	1	1	0	1	1	1
1	0	1	1	1	1	0	1	1	1
1	0	1	1	1	1	0	1	1	1
1	0	1	1	1	1	0	1	1	1
1	0	4	1	1	1	0	3	1	1
1	0	1	1	1	1	0	1	1	1
1	0	2	2	1	1	0	1	1	1
1	0	1	1	1	1	0	1	1	1
1	0	3	3	1	3	0	4	3	4
4	1	4	1	1	4	1	1	1	1
1	0	2	2	1	1	1	1	1	1
1	0	1	1	1	1	0	1	1	1
3	0	1	1	1	1	0	3	1	1
1	0	1	1	1	1	0	1	1	1
1	0	1	1	1	1	0	1	1	1
1	0	1	1	1	1	0	1	1	1
1	0	1	1	1	1	1	1	1	1
1	0	4	2	1	1	0	1	1	1
1	1	1	1	1	1	0	1	2	1
1	0	1	1	1	1	0	1	1	1
1	0	1	1	1	1	0	1	1	1
1	0	1	1	1	1	0	2	1	1
1	0	1	1	1	4	1	1	1	2
1	1	3	1	1	3	1	1	2	1
1	1	1	2	1	3	1	2	2	3
1	1	1	1	1	2	1	2	2	2
1	0	1	1	1	1	0	1	1	1
1	0	1	1	1	1	0	1	1	1
1	0	1	1	1	1	0	4	4	4
1	1	1	1	1	1	0	4	4	4
1	1	4	1	1	1	1	1	1	1

1	0	1	1	1	1	0	1	1	1
4	1	3	4	3	4	1	4	4	4
1	0	1	1	1	1	0	1	1	1
4	1	1	1	1	4	1	4	4	1
3	1	1	1	1	1	0	4	4	4
1	0	1	1	1	1	0	1	1	1
1	0	1	1	1	1	0	1	1	1
1	0	2	1	1	1	0	1	1	1
1	1			1	1	1	1	1	1
1	0	4	1	1	1	0	1	1	1
1	1	1	1	1	1	0	1	1	1
2	0	1	1	2	1	0	1	3	2
2	1	1	1	1	2	0	1	2	1
1	0	1	1	1	1	0	1	1	1
1	0	1	1	1	1	0	1	2	1
1	0	1	1	1	1	0	1	1	1
1	0	1	1	1	1	0	1	1	1
1	1	1	1	1	1	1	1	1	1

3	0	1	1	1	3	1	1	3	3
1	0	4	1	1	4	1	1	1	1
2	0	1	1	1	1	0	1	2	3
1	1	1	3	1	3	1	1	4	4
3	0	3	1	1	3	0	3	3	3
1	0	1	1	1	1	0	1	1	1
1	0	1	1	1	1	0	1	2	2
3	0	1	1	1	1	0	1	1	1
3	0	1	1	1	1	0	1	1	1
1	0	1	1	1	3	1	1	1	1
1	0	1	1	1	4	0	1	1	1
1	0	1	1	1	1	0	1	1	1
4	1	2	1	1	4	1	3	4	3
1	0	3	1	1	1	0	1	1	1
1	0	1	1	1	1	0	1	1	1
1	0	1	1	1	1	0	1	1	1
1	0	1	1	1	1	0	1	1	1
1	0	1	1	1	1	0	1	1	1
1	0	1	1	1	1	0	3	3	3
4	0	1	1	1	3	1	1	1	1
1	0	1	1	1	1	0	1	1	1
4	0	4	3	4	4	0	4	4	4
3	1	1	1	1	1	0	4	1	4
1	0	1	1	1	1	0	1	1	1
1	0	1	1	1	1	0	1	2	1
1	0	1	1	1	1	0	1	1	1

1	0	1	1	1	1	0	1	1	1

3	1	1			1	0	4	3	3
1	0	1	1	1	1	0	1	1	1
2	0	1	1	2	3	0	3	3	1
3	0	1	1	1	4	1	1	1	1
1	0	1	1	1	1	0	1	1	1
1	0	1	1	1	1	0	3	1	1
1	0	1	1	1	1	0	1	1	1
	0	1	3	3	4	0	3	4	4
1	0	1	1	1	1	0	2	1	1
3	0	1	1	1	1	0	3	3	3
1	0	1	1	1	1	0	1	1	1
1	0	1	1	1	1	0	1	1	1
1	0	1	1	1	1	0	1	1	1
1	0	1		1	1	0	1	1	1
1	0	1	1	1	1	0	1	1	1
1	0	1	1	1	1	0	1	1	1
1	0	1	1	1	1	0	1	1	1
1	0	1	1	1	1	0	1	1	1
1	0	1	1	1	1	0	1	1	1
1	0	1	4	1	1	0	1	1	1
1	0	1	1	1	1	0	1	1	1
1	0	1	1	1	1	0	1	1	1
1	0	4	1	1	1	0	1	1	1
1	0	1	1	1	1	0	1	1	1
1	0	1	1	1	1	0	1	1	1
1	1	1	1	1	1	0	3	1	1
1	0	1	1	1	1	0	1	1	1
1	0	1	1		3	1	1	1	1
1	0	1	1	1	1	0	1	1	1
1	0	1	1	1	1	0	1	1	1
1	0	1	1	1	1	0	1	1	1
4	1	1	1	4	1	1	3	4	4
3	0	1	1	1	2	0	3	3	4
	0	1	1	1	1	0	1	1	1
1	0	1	1	1	1	0	1	1	1
	0	1	1	1	1	0	1	1	1
2	1	1	1	1	3	1	1	3	3
1	0	1	1	1	1	0	1	1	1
1	0	1	1	1	4	0	1	1	1
1	0	1	1	1	1	0	1	1	1
1	0	1	1	1	1	0	1	4	3
1	0	1	1	1	1	0	2	1	1
1	0	1	1	1	1	0	1	1	1
1	1	1	4	1	1	1	3	3	3
1	0	1	1	1	1	0	1	1	1

1	0	1	1	1	1	0	3	1	1
1	0	1	1	1	1	0	1	1	1
1	0	1	1	1	1	0	1	1	1
1	0	1	1	1	1	0	1	1	1
2	0	1	1	1	1	0	2	2	1
1	0	3	3	2	4	1	2	3	1
1	0	1	1	1	1	0	1	1	1
3	1	1	1	1	1	0	1	1	1
1	0	4	1	1	1	0	2	3	1
2	0	4	1	1	1	0	1	3	1
1	0	1	1	1	1	0	1	1	1
1	0	1	1	1	1	0	1	1	1
1	0	1	1	1	1	0	1	1	1
1	0	1	1	1	1	0	1	1	1
1	1	4	1	1	1	1	1	1	3
1	0	1	1	1	1	0	1	1	1
1	0	4	1	1	1	0	4	1	1
1	0	1	1	1	1	0	1	1	1
1	0	1	1	1	1	0	1	1	1
1	0	1	4	1	3	1	1	1	1
1	0	4	1	1	4	1	1	1	1
4	0	4	4	1	1	1	4	4	4
1	1	1	1	1	3	1	1	1	1
1	0	4	1	1	1	0	1	1	1
1	0	1	1	1	1	0	1	1	1
1	0	1	1	1	1	0	1	1	1
1	0	1	1	1	1	0	1	1	1
1	0	1	1	1	1	0	1	1	1
1	0	1	1	1	1	0	3	3	1
1	0	1	1	1	1	0	3	3	3
1	0	1	1	1	1	0	1	1	1
1	0	4	1	1	1	0	1	1	1
1	0	4	1	1	1	0	1	1	1
1	0	1	1	1	1	0	1	1	1
1	0	1	1	1	1	0	1	1	1
1	0	1	1	1	1	0	1	1	1
4	1	1	1	4	3	1	3	3	3
1	0	1	1	1	1	0	1	1	1
1	0	1	1	1	1	0	1	1	1
1	0	1		1	1	0	1	1	1
1	0	1	1	1	1	0	1	1	1
1	0	1	1	1	1	0	1	1	1
2	0	2	1	1	1	0	1	1	1
2	1	1	1	1	1	0	2	3	1
1	0	1	1	1	1	0	1	1	1
1	1	1	1	2	3	1	2	2	3
3	0	3	1	1	3	1	2	3	3

1	0	1	1	1	3	0	2	3	1
1	1	4	4	4	4	1	1	3	3
1	0	1	1	1	1	0	1	1	1
1	0	1	1	1	1	0	1	1	1
2	0	4	1	1	3	0	2	4	2
4	1	2	3	3	3	1	4	4	4
1	0	1	1	1	1	0	1	1	1
1	0	1	1	1	1	0	1	1	1
1	1	1	1	1	1	0	2	2	1
1	0	1	1	2	2	0	3	3	3
1	0	1	1	1	1	0	1	1	1
4	1	4	4	4	3	1	4	4	4
3	1	1	1	4	4	1	3	3	3
4	1	1	1	1	4	1	4	4	4
1	0	1	1	1	1	0	1	1	1
1	0	3	3	1	1	0	1	3	1
2	1	1	2	2	3	1	1	1	1
1	0	1	1	1	1	0	1	1	1
1	0	1	1	1	1	0	1	1	1
1	0	1	1	1	1	0	1	1	1
1	0	1	1	1	1	0	1	1	1
1	0	1	1	1	1	0	1	2	1
3	0	1	3	3	3	1	4	3	1
1		1	1	1	1		1	1	1
1	0	1	1	1	1	0	1	1	1
1	0	1	1	1	1	0	1	1	1
1	0	2	1	1	1	0	1	1	1
3	0	2	2	2	2	0	2	2	3
1	0	1	1	1	1	0	1	1	1
1	0	1	1	1	1	0	1	1	1
4	1	4	1	1	1	0	4	4	4
4	0	4	1	1	4	0	4	4	4
1	0	1	1	1	1	0	1	1	1
4	0	1	1	1	1	0	1	1	1
3	0	1	1	1	1	0	1	1	1
3	1	1	1	1	1	0	1	1	1
3	1	4	1	1	1	0	1	1	1
1	0	1	1	1	1	0	1	1	1
1	0	1	1	1	1	0	1	1	1
1	0	1	1	1	1	0	1	1	1
1	0	1	1	1	1	0	3	3	3
1	0	1	1	1	1	0	1	1	1
2	0	1	1	1	1	0	1	1	1
4	0	1	1	1	1	0	1	1	1
1	1	1	1	1	1	0	4	4	4
1		1	3	3	3	0	2	2	2
3	0	4	4	1	1	0	1	4	2

1	0	1	1	1	1	0	1	1	1
4	1	3	3	3	3	1	1	1	1
4	1	4	4	1	4	1	2	2	1
1	0	1	1	1	1	0	1	1	1
1	1	1	1	1	4	1	1	1	1
3	1	1	1	1	1	1	4	4	4
4	0	1	1	1	1	0	1	1	1
4	0	1	1	1	1	0	1	1	1
1	1	1	1	1	1	0	1	1	1
3	1	4	1	1	1	0	3	1	3
1	0	1	1	1	1	0	3	3	1
1	0	1	1	1	1	0	1	1	1
3	1	2	4	4	4	1	4	4	4
1	0	1	1	1	1	0	1	1	1
1	0	1	1	1	1	0	1	1	1
1	0	1	1	1	1	0	4	4	4
1	0	1	1	1	1	0	4	4	4
1	0	1	1	1	1	0	3	1	3
1	0	2	1	1	1	0	4	1	3
1	0	1	1	1	1	0	1	1	1
1	0	1	1	1	1	0	3	1	1
1	0	1	1	1	1	0	1	1	1
1	0	1	1	1	1	0	1	1	1
1	0	1	1	1	1	0	3	1	1
1	0	1	1	1	1	0	1	1	1
2	0	1	1	1	1	0	1	1	1
1	0	1	1	1	3	0	2	2	2
2	0	1	1	1	1	0	1	1	1
1	0	1	1	1	1	0	1	1	1
1	1	4	1	1	4	1	4	4	4
1	0	1	1	1	1	0	1	1	1
1	0	1	1	1	1	0	1	1	1
1	0	2	1	1	1	0	2	1	1
1	0	1	1	1	1	0	1	1	1
1	0	1	1	1	1	0	1	1	1
1	0	1	1	1	1	0	1	1	1
1	0	1	1	1	1	0	1	1	1
1	0	1	1	1	1	0	1	1	1
1	0	1	1	1	1	0	1	1	1
1	0	1	1	1	1	0	1	1	1
1	0	1	1	1	1	0	1	1	1
1	0	1	1	1	1	0	1	1	1
1	0	1	1	1	1	0	2	2	2
1	1	1	2	2	4	1	4	3	4
1	0	1	1	1	1	0	1	1	1
1	0	4	1	1	1	0	1	1	4
1	0	2	2	1	1	0	2	1	1

1	0	1	1	1	1	0	1	1	1
1	0	1	1	1	1	0	1	1	1
1	0	1	1	1	1	0	2	4	4
1	0	1	1	1	1	0	1	1	1
1	0	1	1	1	1	0	1	2	1
1	0	4	4	1	1	0	1	3	1
1	0	4	1	2	2	0	2	1	1
1	0	1	1	1	1	0	1	1	1
1	0	1	1	1	1	0	2	1	1
1	0	1	1	1	1	0	1	1	1
1	0	3	1	1	1	0	1	1	1
1	0	1	1	1	1	0	1	1	1
1	0	1	1	1	1	0	2	2	2
1	0	1	1	1	1	0	1	1	1
1	0	1	1	1	1	0	1	1	1
1	0	1	1	1	1	0	1	1	1
1	0	1	1	1	1	0	1	1	1
1	0	1	1	1	1	0	1	1	1
1	0	1	1	1	1	0	1	1	1
1	0	4	1	1	4	0	1	3	1
1	0	1	1	1	1	0	2	1	1
1	0	1	1	1	1	0	4	4	4
1	0	1	1	1	1	0	1	1	1
1	0	1	1	1	1	0	1	1	1
1	0	1	1		1	0	1	1	1
1	0	1	1	1	1	0	1	1	1
1	0	1	1	1	1	0	1	2	1
1	0	1	1	1	1	0	1	1	1
1	0	1	1	1	1	0	1	1	1
2	0	1	1	1	1	0	1	1	1
1	0	1	1	1	1	0	1	1	1
1	0	4	1	1	1	0	1	1	1
1	0	1	1	1	1	0	1	1	1
3	1	1	2	1	3	1	1	1	1
1	0	1	1	1	1	0	1	1	1
1	0	1	1	1	1	0	1	1	1
1	0	1	1	1	1	0	1	1	1
1	0	1	1	1	1	0	1	1	1
1	0	1	1	1	1	0	1	1	1
1	0	1	4	1	1	1	1	1	1
1	0	1	1	2	4	0	3	3	3
4	0	1	1	2	4	0	3	3	4
1	0	1	1	1	1	0	1	1	1
1	0	1	1	1	1	0	1	1	1
1	0	1	1	1	1	0	1	1	1
1	0	1	1	1	1	0	1	1	1
1	0	4	1	3	1	0	1	1	1

1	0	1	1	1	1	0	1	3	1
1	0	1	1	1	1	0	1	1	1
1	0	1	1	1	1	0	1	1	1
1	0	1	1	1	1	0	1	1	1
1	0	1	1	1	1	0	1	1	1
1	0	1	1	1	1	0	1	1	1
1	0	4	1	1	1	0	1	1	1
1	0	1	1	1	1	0	1	1	1
1	0	4	1	1	1	0	1	1	1
1	0	1	1	1	1	0	1	1	1
1	0	1	1	1	1	0	1	1	1
1	0	1	1	1	1	0	1	1	1
1	0	1	1	1	1	0	1	1	1
1	0	1		1	1	0	1	1	1
1	0	1		1	1	0	1	1	1
1	0	1	1	1	1	0	1	1	1
						0			
1	0	1	1	1	1	0	1	1	1
		1	1	1	1	0	1	1	1
1	0	2	1	1	1	0	1	1	1
1	0	1	1	1	1	0	2	1	1
1	1	4	1	1	4	1	4	3	4
1	0	1	1	1	1	0	1	1	1
1	0	1	1	1	1	0	2	1	1
1	0	1	1	1	1	0	1	1	1
1	0	1	1	1	1	0	1	1	1
1	0	1	1	1	1	0	1	1	1
4	1	1	1	4	4	1	3	4	4
1	0	1	1	1	1	0	1	1	1
1	0	4	1	1	1	1	2	3	2
1	0	1	1	1	1	0	1	1	1
	0	1	1	1	1	0	1	1	1
1	0	1	1	1	1	0	1	1	1
2	1	1	2	4	4	1	4	3	1
1	0	1	1	1	1	0	1	1	1
4	0	1	1	4	4	0	4	4	4
1	0	4	1	1	1	0	1	1	1
1	0	1	1	1	1	0	1	1	1
3	1	1	1	1	1	0	1	2	1
2	0	4	1	1	4	0	1	3	1
1	0	1	1	1	1	0	1	1	1
1	0	1	1	1	1	0	1	1	1
1	0	3	1	1	1	0	1	1	1
1	0	1	1	1	1	0	1	1	1
1	0	1	1	1	3	0	1	1	1
4	0	4	1	4	4	0	3	2	1
1	0	1	1	1	1	0	1	1	1

1	0	1	1	1	1	0	1	1	1
4	1	4	1	1	3	1	3	3	3
1	0	1	1	1	1	0	1	1	1
4	0	1	1	1	1	0	2	3	3
1	0	1	1	1	1	0	1	1	1
1	0	1	1	1	1	0	3	1	1
1	0	1	1	1	1	0	1	1	1
1	0	4	1	1	1	0	1	1	1
1	0	1	1	1	1	0	1	1	1
1	0	1	1	1	1	0	1	1	1
1	0	1	1	1	1	0	1	1	1
1	0	1	1	1	1	0	1	1	1
1	0	1	1	1	1	0	1	1	1
4	1	1	1	2	3	0	1	1	1
1	0	1	1	1	1	0	1	1	1
1	0	4	1	4	1	0	4	4	1
1	0	1	1	1	1	0	1	1	1
1	1	1	1	4	4	1	4	4	4
1	0	1	1	1	1	0	1	1	3
1	0	1	1	1	1	0	1	1	1
3	1	1	1	3	1	1	3	3	3
		1	1	1	1	0	1	1	1
1	0	4	1	1	4	0	1	1	1
		1	1	1	1	0	1	1	1
1	0	1	1	1	1	0	1	1	1
1	0	4		1	1		3	3	3
1	0	1	1	1	1	0	1	1	1
3	0	3	3	3	3	0	3	3	3
1	1	1	1	1	1	0	4	4	4
4	0	1	1	1	1	0	1	1	1
1	0	1	1	1	1	0	1	1	1
1	0	1	1	1	1	0	2	1	1
1	0	1	1	2	3	0	1	1	1
1	0	1	1	1	1	0	1	1	1
1	0	1	1	1	1	0	1	1	1
4	0	4	1	4	4	0	4	4	3
1	0	1	1	1	1	0	1	1	1
3	1	3	3	3	3	1	1	1	1
3	1	3	3	3	3	1	1	1	1
1	0	1	1	1	1	0	1	1	1
1	0	1	1	1	1	0	3	3	2
1	0	1	1	1	2	0	1	2	3
4	1	1	1	1	1	0	2	3	3
1	0	4	1	1	1	0	1	1	1
1	0	1	1	1	1	0	1	1	1
1	0	1	1	1	1	0	1	1	1
2	1	4	4	4	4	0	1	1	1

3	1	2	1	1	2	1	1	1	1
1	0	1	1	1	1	0	1	1	1
1	0	1	1	1	1	0	1	1	1
1	0	1	1	1	1	0	1	1	1
1	0	1		4	1	0	4	4	4
1	0	1	1	1	4	1	1	3	3
1	0	1	1	1	1	0	1	1	1
1	0	4	1	1	2	0	4	4	1
1	0	1	1	1	1	0	1	1	1
1	0	1	1	1	1	0	1	1	1
1	1	1	1	1	1	1	2	2	2
1	0	1	1	1	1	0	1	1	1
2	0	2	2	2	2	0	3	3	3
1	0	1	1	1	1	0	1	1	1
1	0	3	4	1	1	0	1	1	1
1	0	1	1	1	1	0	1	1	1
3	1	1	1	1	1	0	1	1	1
1	0	1	1	1	1	0	1	1	1
4	0	1	1	1	3	1	4	4	4
4	0	1	1	1	3	1	4	4	4
1	1	4	1	1	1	0	1	1	1
1		1	1	1	1		1	1	1
1	0	4	1	1	1	0	1	3	3
1	0	1	1	1	1		1	1	1
1	0	1	1	1	1	0	1	1	1
2	1	1	1	1	1	0	3	3	1
1	0	4	4	1	4	0	1	4	4
1	0	1	1	1	1	0	1	1	1
1	0	1	4	4	4	1	3	3	4
1	0	1	1	1	1	0	1	1	1
1	0	1	1	1	1	0	1	1	1
1	1	1	1	1	1	0	1	1	1
4	0	4	1	4	1	0	1	1	1
1	1	1	1	1	3	1	1	1	1
2	0	1	1	1	4	0	3	3	1
1	0	1	1	1	1	0	1	1	1
1	0	1	1	1	1	0	2	2	2
1	0	1	1	1	1	0	1	1	1
1	0	1	1	1	1	0	1	1	1
1	0	1	1	1	1	0	4	4	4
1	0	1	1	1	1	0	1	1	1
2	0	2	1	1	1	0	1	1	1
1	0	1	1	1	1	0	1	1	1
1	0	4	1	1	1	0	1	1	1
1	0	2	1	1	1	0	1	1	1
1	0	1	1	1	1	0	1	1	1
1	0	1	1	1	1	0	3	3	3

1	0	1	1	1	1	0	1	1	1
1	0	1	1	1	1	0	1	1	1
1	0	2	1	1	1	0	1	1	1
3	0	2	1	1	1	0	4	1	1
4	0	4	1	1	4	0	4	4	4
1	0	1	1	1	1	0	1	1	1
1	0	1	1	1	1	0	3	1	2
1	0	1	1	1	1	0	1	1	1
1	0	1	1	1	1	0	1	1	1
1	0	1	1	1	1	0	1	1	1
1	0	1	1	1	1	0	1	1	1
1	0	3	1	1	3	0	1	1	1
1	0	3	1	1	3	0	1	1	1
1	0	1	1	1	1	0	1	2	1
1	0	1	1	1	1	0	1	3	1
1	0	1	1	1	1	0	1	1	1
1	1	1	1	1	1	0	4	4	4
1	0	1	1	1	1	0	4	4	4
1	0	1	1	1	1	0	2	2	1
1	1	1	1	4	4	1	1	4	1
1	0	4	1	1	1	0	1	1	1
1	0	1	1	1	1	0			
1	0	1	1	1	1	0	1	1	1
1	0	1	1	1	1	0	1	1	1
1	0	1	1	1	1	0	1	1	1
1	0	1	1	1	1	0	1	1	2
1	0	4	1	1	1	0	1	1	1
1	0	1	1	1	1	0	1	1	1
1	1	3	3	2	3	0	1	3	4
1	0	1	1	1	1	0	1	1	1
3	0	1	1	1	1	0	1	1	1
1	1	1		1	3	1	1	3	1
1	1	1	1	1	1	0	1	1	1
1	0	1	1	1	1	0	1	1	1
1	0	1	3	1	1	0	1	1	1
2	0	1	1	1	1	0	1	1	1
3	1	1	1	1	4	1	1	1	1
1	0	1	1	1	1	0	1	1	1
1	0	1	1	1	1	0	1	1	2
4	0	2	1	1	4	0	1	1	3
1	0	1	1	1	4	0	1	1	1
1	0	4	1	1	1	0	1	1	1
							3	4	4
4	0	4	1	3	4	0	4	4	4
1	0	1	1	1	1	0	1	1	1
1	1	1	1	1	1	0	2	1	1
1	0	4	1	1	1	0	1	1	1

1	0	1	1	1	1	0	1	1	1
1	0	1	1	1	1	0	1	1	1
1	1	1	1	1	1	0	1	2	1
1	0	1	1	1	1	0	1	1	1
3	0	1	2	1	4	0	4	3	4
1	0	1	1	1	4	0	1	4	4
1	0	1	1	1	1	0	2	1	3
1	0	1	1	1	1	0	1	1	1
1	0	1	1	1	4	0	1	4	4

1	0	1	1	1	1	0	1	1	1
1	0	1	1	1	1	0	1	1	1
1	0	1	1	1	1	0	1	1	1
1	0	1	1	1	1	0	1	1	1
	0	1	1	1	1	0	4	4	4
1	0	1	1			0	1	1	1
1	0	1	1	1	1	0	4	3	3
1	0	1	1	1	1	0	1	1	1
1	0	1	1	1	1	0	1	1	1
		1	2	3	2	0	2	4	2
	0	3	1	1	1	0	3	3	4
		1	1	1	1	0	1	1	1
1	0	1	1	1	1	0	1	1	1
1	0	1	1	1	1	0	1	1	1
1	1	4	4	3	4	1	4	4	4
1	0	1	1	1	1	0	1	1	1
1	0	3	1	1	1	0	3	3	4
		4		1	1	0	3	1	3
1	0	4	1	1	1	0	1	1	1
1	0	1	1	1	1	0	1	1	1
4	1	1	1			0	1	1	1
1	0	2	1	1	1	0	4	4	4
1	0	1	1	1	1	0	1	1	1
1	0	1	1	1	1	0	4	4	2
1	0	1	1	1	1	0	1	1	1
1	0	4	1	1	1	1	1	1	1
1	0	1	1	1	1	0	1	1	1
1	0	1	1	1	1	0	1	1	1
	0	1	1	2	1	0	2	2	2
1	0	1	1	1	1	0	1	1	1
1	0	1	1	1	1	0	1	1	1
1	0	1	1	1	1	0	1	1	1
1	1	1	1	1	1	0	1	1	1
4	0	1	4	1	1	0	4	4	4
1	0	1	1	1	1	0	1	1	1
1	0	1	1	1	1	0	1	1	1
1	0	1	1	1	1	0	1	1	1

1	0	4	2	1	1	0	1	1	1
1	0	1	1	1	1	0	1	4	1
1	0	1	1	1	1	0	1	1	1
3	0	1	1	1	1	0	1	1	1
4	0	1	1	3	4	0	1	1	1
1	0	1	1	1	1	0	1	1	1
1	0	1	1	1	1	0	1	1	1
4	0	1	1	2	1	0	1	1	1
1	0	1	1	1	1	0	1	1	1
1	0	1	1	1	1	0	1	1	1
1	0	1	1	1	1	0	1	1	1
1	0	1	1	1	1	0	1	1	1
1	0	1	1	1	1	0	1	1	1
1	0	4	3	1	1	0	3	1	1
1	0	1	1	1	1	0	1	1	1
1	0	1	1	1	1	0	1	1	1
1	0	4		1	1	0	2	2	2
1	0	1	1	1	1	0	1	1	1
1	0	1	1	1	1	0	1	1	1
1	0	1	1	1	1	0	1	1	1
1	0	1	1	1	1	0	1	1	1
1	0	1	1	1	1	0	1	1	1
1	0	1	1	1	1	0	1	1	1
1	0	1	1	1	1	0	1	1	1
3	1	1	1	1	1	0	3	3	1
1	0	4	1	1	1	0	1	3	1
1	0	1	1	1	1	0	1	1	1
1	0	3	1	1	1	0	1	1	1
1	0	1	1	1	1	0	4	4	4
4	1	4	4	1	1	1	4	4	4
1	0	1	1	1	1	0	1	1	1
1	0	1	1	1	1	0	1	1	1
1	0	1	1	1	1	0	1	1	1
1	0	1	1	1	1	0	1	1	1
4	0	1	1	1	1	0	1	1	1
1	0	1	1	1	1	0	1	1	1
1	0	1	1	1	1	0	3	3	3
1	0								
1	0	1	1	4	4	1	4	4	4
1	0	1	1	1	1	0	1	1	1
1	0	4	3	1	1	0	1	2	1
1	0	1	1	1	1	0	2	2	1
1	0	1	1	1	1	0	2	1	1
1	0	4	4	4	4	0	3	3	3
1	0	1	1	1	1	0	1	1	1

		2					4	4	4
1	0	1	1	1	1	0	1	1	1
1	0	1	1	1	1	0	1	1	1
1	0	1	1	1	1	0	1	1	1
1	0	1	1	1	1	0	1	1	3
1	1	1	1	1	1	0	1	1	1
2	0	1	1	1	1	0	1	1	1
3	0	1	1	1	1	0	3	3	3
1	0	2	1	1	1	0	1	1	1
1	0	1	1	1	1	0	1	4	4
1	0	3	1	1	1	0	2	2	2
1	0	1	1	1	1	0	1	1	1
1	0	1	1	1	1	0	1	1	1
1	0	1	1	1	1	0	1	1	1
1	0	1	1	1	1	0	1	1	1
1	0	4	1	1	1	0	1	1	1
1	0	4	1	1	1	0	1	1	1
1	0	1	1	1	1	0	1	1	1
3	1	4	4	1	4	1	4	4	4
1	0	1	1	1	1	0	1	3	1
1	0	1	1	1	1	0	1	1	1
1	0	1	1	1	1	0	1	1	1
2	0	1	1	1	1	0	1	1	4
1	0	1	1	1	1	0	1	1	1
1	0	1	1	4	1	0	3	3	3
1	0	1	1	1	1	0	2	1	1
1	0	1	1	1	1	0	2	2	2
1	0	1	1	1	1	0	1	1	1
2	0	3	4	4	3	0	3	4	4
1	1	1	1	3	1	0	1	1	1
4	0	4	4	4	4	0	4	4	4
1	0	1	1	1	1	0	3	1	3
1	0	1	1	1	1	0	1	1	1
1	0	1	1	1	1	0	1	1	1
1	0	1	1	1	1	0	1	1	1
1	0	1	1	4	4	0	4	4	4
1	0	1	1	1	1	0	1	1	1
4	0	1	1	1	1	0	1	1	1
1	1	1	1	4	1	1	3	3	4
4	0	1	1	1	4	0	4	2	4
4	0	1	1	1	3	0	3	1	3
4	0	1	1	1	1	0	4	3	3
1	0	1	1	1	1	0	1	1	1
1	0	1	1	1	1	0	1	1	1
1	0	1	1	1	1	0	3	1	1
3	0	1	1	1	1	0	3	3	3

1	0	4	1	1	1	0	2	1	4
1	0	1	1	1	1	0	1	1	1
1	1	1	1	1	1	0	1	1	1
2	0	1	1	1	1	0	1	1	1
1	0	1	2	1	3	1	1	1	1
1	0	1	1	1	1	0	1	1	1
1	0	1	1	1	1	0	3	1	1
1	1	1	1	1	1	0	3	3	3
4	0	1	1	2	1	0	1	1	1
1	0	1	1	1	1	0	1	1	1
1	0	4	4	1	1	0	4	1	1
1	0	1	1	1	1	0	1	1	3
1	0	1	1	1	1	0	1	1	3
1	0	1	1	1	1	0	1	1	1
1	0	2	1	1	1	0	1	1	1
1	0	2	1	1	1	0	1	1	1
3	0	1	1	1	1	0	4	4	4
1	0	1	1	1	1	0	2	2	1
1	0	1	1	1	1	0	1	1	1
4	0	4	4	1	1	0	1	1	1
1	0	4		1	1	0	1	1	1
1	0	1	1	1	1	0	1	1	1
2	0	1	1	1	1	0	1	1	1
1	0	1	1	1	1	0	1	1	1
1	0	1		1	2	0	1	1	1
1	0	2	1	2	1	0	3	1	3
1	0	1	1	1	1	0	1	1	1
1	0	1	1	1	1	0	1	1	1
1	0	1	1	1	1	0	1	1	1
3	0	1	1	1	1	0	1	1	1
1	0	1	1	1	4	0	3	3	2
1	0	3	1	1	1	0	1	1	1
1	0	4	1	3	1	0	1	1	3
1	0	1	1	1	1	0	1	1	1
1	1	4	2	1	1	1	4	4	4
1	0	1	1	1	1	0	1	1	1
	0	1		1	1	0	1	1	1
1	0	1	1	1	1	0	1	1	1
1	0	4	1	1	1	0	1	1	1
1	0	1	1	1	1	0	1	1	1
1	0	1	1	1	1	0	4	4	3
1	0	1	1	1	1	0	1	1	1
		1	1	1	1	0	1	1	1
2	0	1	1	1	1	0	2	2	2
1	0	1	1	1	1	0	1	1	1
1	0	1	1	1	1	0	1	1	1
1	0	1		1	1	0	1	1	1

2	1	4	3	3	4	1	4	4	3
2	1	2	1	1	4	1	3	2	1
3	1	1	1	1	4	1	4	4	1
1	0	1	1	1	1	0	1	1	1
1	0	1		1	1	0	1	1	1
		1	1	1	2	0	2	1	1
4	1	4	4	4	4	1	1	4	1
3	0	1	1	1	3	0	1	1	1
2	0	1	1	1	1	0	2	1	1
1	0	1	1	1	1	0	1	1	1
1	0	1	1	1	1	0	3	1	1
3	0	1	1	2	4	0	1	1	1
1	0	1	1	1	1	0	1	1	1
1	0	1	1	1	1	0	1	1	1
1	0	4	1	1	4	0	3	3	3
1	0	1	1	1	1	0	1	1	1
1	0	1	1	1	1	0	3	1	1
1	0	4	1	1	1	0	4	1	1
1	0	1	1	1	1	0	1	4	1
1	0	1	1	1	1	0	1	3	1
1	0	1	1	1	1	0	1	1	1
1	0	1	1	1	1	0	1	1	1
1	0	1	1	1	1	0	1	1	1
1	0	1	1	1	1	0	1	1	1
1	0	1	1	1	1	0	3	1	1
1	0	1	1	1	1	0	1	1	1
1	0	1	1	1	1	0	1	1	1
4	1	4	1	1	4	1	3	4	4
1	0	1	1	1	1	0	1	1	1
1	0	1	1	1	1	0	1	1	1
2	0	1	1	1	1	0	1	1	1
1	0	4	1	1	1	0	3	4	3
1	0	1	2	1	4	0	1	1	1
1	0	1	2	1	4	0	1	1	1
1	0	1	3	1	1	0	1	1	1
3	0	1	1	1	1	0	3	1	1
1	0	1	1	1	1	0	1	1	1
1	1	1	1	1	1	0	3	1	1
1	0	1	1	1	1	0	1	2	1
1	0	3	1	1	1	0	1	1	1
1	0	1	1	1	1	0	2	1	1
1	0	1	1	1	1	0	1	1	1
1	0	1	1	1	1	0	1	1	1
1	0	1	1	1	1	0	3	1	1
	0	1	1	1	1	0	1	1	1
4	0	4				0	4	4	4

1	0	4	1	4	4	0	4	4	4
		1	1	1	1	0	1	1	1
2	0	1	1	1	1	0	1	1	1
	0	1	1	1	4	0	1	1	1
	0	1	1	1	1	0	1	1	1
1	0		1	1	3	0			
	0								
3	1	1	1	1	1	0	1	1	1
1	0	1	1	1	1	0	1	1	1
4	1	2	4	1	3	1	1	1	1
1	0	1	1	1	3	0	1	1	1
1	0	1	1	1	1	0	1	3	3
1	0	1	1	1	1	0	1	1	1
1	0	2	1	1	1	0	1	1	1
1	0	1	1	3	2	0	2	2	1
3	1	1	1	1	1	0	1	1	1
3	1	1	1	1	1	0	1	1	1
4	1	4	4	4	1	0	3	4	4
2	0	1	4	2	4	0	1	1	2
1	0	1	1	4	1	0	1	1	1
1	0	1	1	1	1	0	1	1	1
3	1	1	1	1	1	1	1	1	1
1	0	1	1	1	1	0	1	1	1
1	0	1	1	1	1	0	1	1	1
1	0	1	1	1	1	0	1	1	1
3	0	1	1	1	1	0	1	1	1
1	0	1	1	1	1	0	1	1	1
1	0	1	1	1	1	0	1	1	1
1	0					0			
1	0	1	1	1	1	0	1	1	1
1	0	1	1	1	1	0	1	1	1
1	0	1	1	1	1	0	1	1	1
1	0	1	1	1	1	0	1	1	1
1	0	1	1	1	1	0	1	1	1
1	0	1	1	1	1	0	1	1	1
2	1	1	1	1	1	0	1	1	1
1	0	1	1	1	1	0	1	1	1
1	0	1	1	1	2		1	1	1
1	0	1	1	1	1	0	1	1	1
1	0	1	1	1	1	0	1	1	1
1	0	1	1	1	1	0	1	1	1
2	0	2	1	1	1	0	2	1	3
3	0	1	1	1	1	0	1	1	1
1	0	1	1	1	1	0	1	1	1
1	0	1	1	1	1	0	1	1	1

1	0	1	1	1	1	0	1	1	1
1		1	1	1	1		1	1	1
1	0	1	1	1	1	0	1	1	1
1	0	1	1	1	1	0	2	2	3
1	0	1	1	1	1	0	1	1	1
1	0	1	1	1	1	0	1	1	1
2	1	1	1	1	1	0	3	2	2
1	0	1	1	1	2	0	2	2	2
		1	1	1	1	0			
1	0	2	3	4	3	0	3	3	3
2	0	2	2	2	2	1	1	2	1
1	0	2	3	1	1	0	1	1	1
1	0	1	1	1	1	0	1	1	1
4	1	1	1	1	1	0	1	1	1
							1	1	1
1	0	1	1	1	1	0	1	1	1
4	0	1	1	1	4	0	4	4	4
4	0	1	1	4	4	0	3	3	4
3	0	1	1	1	1	0	1	1	1
4	0	4	4	4	4	0	4	4	4
1	1	1	1	1	1	0	1	1	1
1	0	1	1	1	1	0	1	1	1
1	0	1	1	1	1	0	1	1	3
1	0	1	1	1	1	0	1	1	3
1	0	1	1	1	1	0	1	1	1
4	1	4	4	1	4	1	4	4	4
4	0	1	1	1	1	0	1	1	1
1	0	1	1	1	1	0	4	4	3
1	0	1	1	1	1	0	1	1	1
2	0	1	1	1	1	0		1	1
1	0	1	1	1	1	0	2	1	1
1	0	4	1	1	1	0	1	1	1
1	0	1	1	1	1	0	1	1	1
1	0						1	1	1
1	0	1	1	1	1	0	1	1	1
4	0	4	1	1	4	0	4	4	4
4	0	4	1	1	4	0	4	4	4
3	0	1	1	1	3	0	1	1	1
3	0	1	1	1	3	0	1	1	1
1	0	1	1	1	1	0	2	1	2
1	0	1	1	1	1	0	3	1	2
1	0	1	1	1	1	0	4	3	1
4	1	1	1	4	4	1	4	4	4
3	0	1	1	1	2	0	1	1	1
3	0	1	1	1	2	0	1	1	1
4	0	1	1	1	1	0	4	4	4
4	0	1	1	1	1	0	4	4	4

1	0	1	1	1	1	0	2	1	2
1	0	1	1	1	1	0	4	4	4
1	0	1	1	1	1	0	1	1	3
3	0	1	1	1	1	0	1	1	2
2	0				1	0	2	2	3
1	0	1	1	1	1	0	1	1	1
1	0	1	1	1	3	0	1	1	1
1	0	1	1	1	1	0	1	1	1
1	0	1	1	1	1	0	1	1	1
1	0	1	1	1	1	0	1	1	1
1	0	1	1	1	1	0	1	1	1
4	0	1	1	1	1	0	4	1	1
1	0	2	1	1	1	0	1	1	1
4	0	1	1	1	1	0	3	3	3
1	0	1	1	1	1	0	1	1	1
4	0	1	1	1	1	0	1	4	4
1	0	1	1	1	1	0	1	1	1
1	0	1	1	1	1	0	1	1	1
1	0	1	1	1	1	0	1	1	1
1	0	1	1	1	1	0	1	1	1
1	0	1	1	1	1	0	3	1	1
1	0	1	1	1	1	0	1	1	1
1	0	1	1	1	1	0	1	1	1
2	0	1	1	4	4	0	4	4	4
1	0	1	1	1	1	0	1	1	1
1	1	1	1	1	1	0	1	2	1
1	0	1	1	1	1	0	1	1	1
1	0	4	1	1	1	0	1	1	1
1	0	1	1	1	1	0	1	1	1
1	0	1	1	1	1	0	1	1	1
1	0	1	1	1	1	0	1	1	1
1	0	4	1	1	1	0	1	1	1
1	0	1	1	1	1	0	1	1	1

1	0	1	1	1	1	0	1	1	1
1	0	1	1	3	4	0	4	1	1
1	0	1	1	1	1	0	1	1	1
1	0	1	1	1	1	0	1	1	1
1	0	1	1	1	1	0	1	2	1
3	0	1	1	1	1	0	4	4	3
1	0	1	1	1	1	0	4	4	4
3	0	1	1	1	1	0	3	1	3
3	0	1	1	3	4	0	3	1	3
1	0	1	1	1	1	0	1	1	1
1	1	3	1	1	3	1	1	1	1
4	0	1	4	1	1	0	1	1	4
1	0	1	1	1	1	0	1	1	1

1	0	1	1	1	1	0	1	1	1
1	0	1	1	1	1	0	2	1	1
3	0	1	1	1	1	0	1	1	1
1	0	1	4	1	4	0	1	1	1
1	0	1	1	1	1	0	1	1	1
1	0	1	1	1	1	0	1	1	1
1	0	1	1	1	1	0	1	1	1
1	0	1	1	1	1	0	1	1	1
1	0	4	1	4	4	0	1	1	1
1	0	1	1	1	1	0	1	1	1
1	0	1	1	1	1	0	4	4	4
1	0	1	1	1	1	0	1	3	3
1	0	1	1	1	1	0	2	4	2
1	0	1	1	1	1	0	1	1	1
1	0	1	1	1	1	0	1	1	1
1	0	1	1	1	1	0	1	1	1
1	0	1	1	1	1	0	1	1	1
4	0	1	1	1	1	0	1	1	1
4	0	1	1	1	1	0	1	1	1
1	0	1	1	1	1	0	1	1	1
1	0	1	1	1	1	0	2	3	1
1	0	1	1	1	1	0	1	1	1
4	1	1	4	1	1	0	4	4	4
1	0	1	1	1	1	0	1	1	1
1	0	1	1	1	1	0	1	1	1
1	0	1	3	1	1	0	1	1	1
1	0	1	1	1	1	0	1	1	1
3	1	1	1	1	1	0	1	1	1
2	0	1	1	1	1	0	1	1	1
2	0	1	1	1	1	0	1	1	1
1	0	1	1	1	1	0	1	1	1
1	0	1	1	1	1	0	1	2	1
1	0	1	1	1	1	0	1	1	1
1	0	1	1	1	1	0	1	1	1
3	0	1	1	1	1	0	1	3	3
4	0			1	3	0	1	2	1
1	0	1	1	1	1	0	1	1	1
3	0	1	1	1	1	0	1	1	1
1	0	1	1	1	1	0	1	1	1
1	0			1	1	0	2	2	2
1	0		1	1	1	1	1	1	1
1	0			1	1	0	1	1	1
1	0	1	1	1	1	0	1	1	1
1	0	1	1	1	1	0	1	1	1
2	1	1	1	3	1	0	1	2	3
1	0	1	1	1	1	0	3	3	3

1	0	1	1	1	1	0	1	1	1
1	0	1	1	2	3	0	4	4	4
1	0	1	1	1	1	0	2	2	2
1	0	1	1	1	1	0	1	1	1
4	0	1	1	4	4	0	3	1	1
1	0	4	1	3	4	0	1	1	1
4	0	1	1	3	4	0	3	3	3
1	0	1	1	1	1	0	4	4	4
1	0	1	1	1	1	0	1	1	1
3	0	1	1	1	3	0	4	4	4
	0	1	1	1	1	0	1	1	1
1	0					0	1	1	1
	0	1	1	1	1	0	1	1	1
1	0	1	1	1	1	0	3	1	1
	0	1	1	1	1	0	4	4	4
		4	3	1	1	0	4	4	4
		1	1	1	1	0	4	4	4
1	0	1	1	1	1	0	1	1	1
		1	1	1	1	0	1	1	1
1	0	1	1	1	1	0	1	1	1
	0	4	4	4	4	0	4	4	4
1	0	1	1	1	1	0	1	1	1
1	0	1	1	1	1	0	1	1	1
1	0					0	1	1	1
	0	1	1	1	1	0	1	1	1
1	0	1	1	1	1	0	1	1	1
	0	4	1	1	4	0	4	2	4
1	0	1	1	1	1	0	1	1	1
1	0	1	1	1	1	0	4	4	4
1	0	1	1	1	1	0	1	1	1
	0				1	0	1	1	1
1	0	1	1	1	1	0	1	1	1
1	0	1	1	1	1	0	3	3	3
4	0	4	3	4	4	1	4	4	4
1	0	1	1	1	1	0	1	1	1
1	0	1	1	1	1	0	1	1	1
		1	1	1	1	0	1	1	1
1	0	1	2	1	1	0	1	2	2
1	0	1	1	1	1	0	1	1	1
3	0	1	1	1	1	0	4	3	3
1	0	1	1	1	1	0	4	3	3
1	0	1	1	1	1	0	1	1	1

	0	1	1	1	1	0	1	1	1
1	0	1	1	1	1	0	2	3	1
4	0	3	2	3	3	0	4	4	4
1	0	1	1	1	1	0	1	1	1
1	0	1	1	1	1	0	1	1	2
1	0	1	1	1	1	0	1	1	1
1	0	1	1	1	1	0	1	1	1
1	0	1	1	1	1	0	1	1	1
	0	1	1	1	1	0	1	1	1
1	0	1	1	1	1	0	1	1	1
1	0	1	1	1	1	0	1	1	1
4	0	1	1	4	4	1	4	4	4
1	0	1	1	1	1	0	1	1	1
1	0	1	1	1	1	0	1	1	1
4	0	4	4	4	4	0	4	4	4
4	0	1	1	1	1	0	1	4	2
1	0	1	1	1	1	0	4	3	3
1	0	1	1	1	1	0	1	1	1
1	0	4	4	1	4	0	4	4	4
		1	1	1	2	0	1	1	1
		1	1	1	1	0	1	1	1
	0		1	4	4	1	4	4	4
1	0	1	3	1	3	0	3	2	2
1	0	1	1	1	2	0	2	1	1
		1	1	1	4	0	1	1	1
1	0	1	1	1	1	0	1	1	1
1	0	1	1	1	1	0	1	1	1
4	0	1	1	1	1	0	4	4	4
1	0	1	1	1	1	0	1	1	1
1	0	1	1	1	1	0	1	1	1
1	0	1	1	1	1	0	1	1	1
1	0	1	1	1	1	0	1	1	1
1	0	1	1	1	1	0	1	1	1
1	0	1	1	1	1	0	1	1	1
1	0	1	1	1	1	0	1	1	1
1	0	1	1	1	1	0	1	1	3
3	0	1	1	3	3	0	1	1	1
1	0	1	1	1	1	0	1	1	1
1	0	1	1	1	1	0	1	1	1
1	0	1	1	1	1	0	1	1	1
1	0						3		
1	0	1	1	1	1	0	3	1	1
1	0	1	1	1	1	0	3	1	1
1	0	1	1	1	1	0	1	1	1
1	0	1	1	1	1	0	1	1	1
1	0	1	1	1	1	0	1	1	1
1	0	1	1	1	1	0	1	1	1

1	0	1	1	1	1	0	1	1	1
1	1	1	1	1	1	0	1	1	1
1	0	1	1	1	1	0	1	1	1
1	0						1	1	1
		1	1	1	1	0	1	1	1
1	0	1	1	1	1	0	1	1	1
1	0	1	1	1	1	0	1	1	1
1	0	1	1	1	4	0	4	4	4
1	0	1	1	1	1	0	1	1	1
1	0	4	1	1	1	0	1	1	1
1	0	1	1	1	1	0	2	1	1
1	0	1	1	1	1	0	4	1	1
1	0	1	1	1	1	0	1	1	1
1	0	1	1	1	1	0	1	1	1
1	0	1	1	1	1	0	1	1	1
1	0	1	1	1	1	0	1	1	1
1	0	1	1	1	1	0	3	1	1
1	0	1	1	1	1	0	1	2	1
1	0	1	1	1	1	0	1	1	1
1	0	1	1	1	1	0	1	1	1
1	0	1	1	1	1	0	1	1	1
1	0	4	2	1	1	0	1	1	1
1	0	1	1	1	1	0	1	1	1
1	0	1	1	1	1	0	1	1	1
1	1	1	1	1	1	0	3	1	1
1	0	1	1	1	1	0	1	1	1
1	0	4	4	1	4	0	3	1	3
1	0	1	1	1	1	0	1	1	1
1	0	1	1	1	1	0	1	1	1
1	0	3	3	1	1	1	1	1	1
1	0		1	1	1	0	1	1	1
1	0	1	1	1	1	0	1	1	1
1	0	1	1	1	1	0	1	1	1
1	0	1	1	1	1	0	1	1	1
1	0	1	1	1	1	0	1	1	1
1	0	1	1	1	1	0	1	1	1
4	0	1	1	1	4	1	4	4	4
1	0	1	1	1	1	0	1	2	1
1	0		1	1	1	0	1	2	1

kick	gun	vq148	hap12m	force	sexex	exforce	vq183	nothusb	manyforc
1	1	0		1	1	1	0	0	0
1	1	0		1	1	1	0	0	0
1	1	0		1	1	1	0	0	0
1	1	0		2	1	1	0	1	1
1	1	0		1	1	1	0	0	0
1	1	0		1	1	1	0	0	0
1	1	0		1	1	1	0	0	0
1	1	0		1	1	1	0	0	0
1	1	0		1	1	1	0	0	0
1	1	0		1	1	1	0	0	0
1	1	0		1	1	1	0	2	2
3	2	1	1	1	4	4	1	0	0
1	1	0		1	1	1	0	0	0
1	1	0		1	1	1	0	0	0
1	1	0		1	1	1	0	0	0
1	1	0		1	1	1	0	0	0
1	1	0		1	1	1	0	0	0
1	1	0		1	1	1	0	0	0
4	1	1	1	1	1	1	0	1	0
1	1	0		1	1	1	0	0	0
1	1	0		2	2	1	1	0	0
1	1	1	1	1	1	1	1	0	0
1	1	0		1	1	1	0	0	0
1	1	0		1	1	1	0	0	0
1	1	0		1	1	1	0	0	0
1	1	0		1	1	1	0	0	0
1	1	0		1	1	1	0	0	0
1	1	1	1	1	1	1	0	0	0
1	1	0		1	1	1	0	0	0
1	1	0		1	1	1	0	0	0
1	1	0		1	1	1	0	0	0
1	1	0		1	1	1	0	0	0
1	1	0		1	1	1	0	0	0
1	1	0		1	1	1	0	0	0
1	1	0		1	1	1	0	0	0
1	1	1	0	1	1	1	0	0	2
3	4	1	1	1	1	2	0	0	0
1	1	0		1	1	1	0	0	0
1	1	0		1	1	1	0	0	0
1	1	0		1	1	1	0	0	0
1	1	0		1	1	1	0	0	0
1	1	0		1	1	1	0	0	0
1	1	0		1	1	1	0	0	0
1	1	0		1	1	1	0	0	0
		9							
		9							

1	1	0		1	1	1	0	0	1
1	1	0		1	1	1	0	0	0
1	1	0		1	1	1	0	0	0
1	1	0		1	1	1	0	0	0
1	1	0		1	1	1	0	0	0
1	1	0		1	1	1	0	0	0
1	1	0		1	1	1	0	0	2
1	1	0		1	1	1	0	0	2
1	1	0		1	1	1	0	2	2
1	1	1	1	1	1	1	0	0	1
1	1	0		1	1	1	0	0	0
1	1	0		1	1	1	0	0	0
1	1	0		1	1	1	0	0	0
1	1	0		1	1	1	0	0	0
1	1	0		1	1	1	0	0	0
1	1	0		3	1	1	0	0	1
1	1	0		1	1	1	0	0	0
1	1	0		1	1	1	0	0	0
1	1	0		1	1	1	0	0	0
1	1	0		1	1	1	0	0	0
1	1	1	0	1	1	1	0	0	0
1	1	0		1	1	1	0		
1	1	9		2	2	1		0	0
1	1	0		1	1	1	0	0	0
1	1	0		3	1	1	1	0	0
1	1	0		1	1	1	0	0	0
1	1	0		1	1	1	0	0	0
1	1	0		1	1	1	0	0	0
1	1	0		1	1	1	0	0	0
1	1	0		4	1	1	1	0	0
1	1	0		1	1	1	0	0	0
1	1	0		1	1	1	0	1	0
1	1	0		1	1	1	0		
		9							
1	1	1	1	1	1	1	0	0	0
1	1	0		1	1	1	0	0	0
1	1	0		1	1	1	0	0	0
1	1	0		1	1	1	0	0	0
4	4	0		1	1	1	0	0	0
1	1	0		1	1	1	0	0	0
1	1	9		1	1	2		0	0
1	1	0		1	1	1	0	0	0
1	1	0		1	1	1	0	0	0
2	3	1	1	4	4	4	1	2	1
1	1	9		1	1	1		1	0
2	3	0		1	1	1	1	1	2
1	1	0		1	1	1	0	0	0

1	1	0		1	1	1	0	0	0
1	1	0		1	1	1	0	0	0
1	1	0		1	1	1	0		
1	1	1	1	4	3	1	1	0	0
1	1	1	1	3	4	1	1	0	0
1	1	0		1	1	1	0	0	0
1	1	0		1	1	1	0	0	0
1	1	0		1	4	1	1	0	0
1	1	0		3	4	1	1	0	0
1	1	0		1	1	1	0	0	0
1	1	0		1	1	1	0	0	0
1	1	1	1	1	1				
1	1	1	1				0		
1	1	0		1	1	1	0	0	0
1	1	0		2	2	2	1	1	1
1	1	0		1	1	1	0	0	0
1	1	1	0	1	1	1	0	0	0
1	1	1	1	4	4	1	1	0	0
1	1	1	0	1	3	1	1	0	0
4	4	1	1	1	1	1	1	0	0
1	1	1	1	1	1	1	0	0	0
1	1	0		1	1	1	0	0	0
1	1	0		1	3	1	1	0	0
1	1	0		1	1		0	0	
1	1	1	1	4	4	1	1	1	1
1	1	0		3	3	3	1	0	0
1	1	0		1	1	1	0	0	0
1	1	1	1	1	1	1	0	0	0
1	1	1	1	1	1	1	0	0	0
1	1	0		1	1	1	0	0	0
1	1	0		1			0	0	0
1	1	0		1	1	1	0	0	0
1	1	0		1	1	1	0	0	0
1	1	0		1	1	1	0	0	0
1	1	0		1	1	1	0	0	0
1	1	0		3	3	1	1	0	0
1	1	0		1	1	1	0	0	0
1	1	0		1	1	1	0	0	0
1	1	0		1	1	1	0	0	0
3	2	1	1	1	1	1	0	0	0
1	1	0		1	1	1	0	0	0
1	1	1	1	1	1	1	0	0	0
2	1	1	1	1	1	1	0		
1	1	0		1	1	1	0	0	0
1	1	0		1	1	1	0	0	0
1	1	0		1	1	1	0	0	0
1	1	0		1	1	1	1	0	0

1	1	0		1	3	1	0	0	0
1	1	0		1	1	1	0	0	0
1	1	0		1	1	1	0	0	0
1	1	0		1	1	1	0	0	0
1	1	0		1	1	1	0	2	1
1	1	0		1	1	1	0	0	0
1	1	0		1	1	1	0	0	0
2	3	1	0	1	1	1	0	0	0
1	1	0		1	1	1	0	0	0
1	1	0		1	1	1	0	0	0
1	1	0		2	1	1	1	1	1
1	1	0		2	1	1	1	0	0
1	1	0		1	1	1	0	0	0
1	1	0		1	1	1	0	0	0
4	4	1	0	1	1	1	1	2	2
1	1	0		1	1	1	0	0	0
1	1	0		1	1	1	0	0	0
1	1	0		1	1	1	0	0	0
1	1	0		1	1	1	0	0	0
1	1	0		1	1	1	0	0	0
1	1	0		1	1	2	1	0	0
1	1	0		1	1	1	0	0	0
1	1	0					0	0	
1	1	0		3	3	3	1	0	0
1	1	0		1	1	1	0	0	0
1	1	0		1	1	1	0	0	0
1	1	0		3	1	1	0	0	0
1	1	0		1	1	1	0	0	0
1	1	0		1	1	1	0	0	0
1	1	0		1	1	1	0	0	0
1	1	0		1	1	1	0	0	0
1	1	0		1	1		0	0	0
1	1	0		1	1	1	0	0	0
1	1	0		1	1	1	0	0	0
		9						0	0
1	1	0		3	2	1	1	0	1
1	1	0		1			0	0	0
1	1	0		3	3	1	1	0	0
1	1	0		1	1	1	0	0	0
3	1	1	1	1	1	1	0	0	0
		0					0	0	0
1	1	1	1	1	1	1	0	0	0
3	1	1	1	3	1	1	0	0	0
1	1	0		3	1	1	0	0	0
1	1	0		1	1	1	0	0	0
1	1	1	0	1	1	1	0	0	0
1	1	0		1	1	1	0	0	0

1	1	0		1	1	1	0	0	0
1	1	0		1	1	1	0	0	0
1	1	0		1	1		0	0	0
1	1	0		1	1	1	0	0	0
1	1	0		1	1	1	0	0	
1	1	0		1	1	1	0	0	0
1	1	0		1	1	1	0	0	0
1	1	0		1	1	1	0	0	0
1	1	0		1	1	1	0	0	0
1	1	0		1	1	1	0	0	0
1	1	0		1	1	1	0	0	0
1	1	0		1	1	1	0	0	0
1	1	0		1	1	1	0	0	0
		9						0	0
1	1	0		1	1	1	0	0	0
4	4	1	1	1	1	1	0	0	0
1	1	0		1	1	1	0	2	1
1	1	0		1	1	1	0	1	1
1	1	0		1	1	1	0	0	0
1	1	0		1	1	1	0	0	0
3	3	0		3	3	3	0	0	0
1	1	0		1	1	1	0	0	0
1	1	0		1	1	1	0	0	0
2	2	0		1	1	1	0	0	0
4	4	1	1	1	1	1	0	0	0
1	1	0		1	1	1	0	0	0
1	1	0		1	1	1	0	0	0
1	1	0		1	1	1	0	0	0
1	1	0		1			0	0	0
1	1	0		1	1	1	0	0	0
1	1	0		1	1	1	0	0	0
		0					0	0	0
1	1	0		1	1	1	0	0	0
		9						0	0
1	1	0		1	1	1	0	0	0
1	2	1	0	1	1	1	1	0	0
1	1	0		1	3	1	1	0	1
1	1	0		1	1	1	0	0	0
1	1	0		1	1	1	0	0	2
1	1	1	1	1	1	1	0		
1	1	0		2	1	1	1	0	0
1	1	0		2	1	1	0	0	0
1	1	0		1	1	1	0	0	2
3	1	1	1	3	3	1	1	0	0
1	1	0		1	1	1	0	0	0
1	1	0		1	1	1	0	1	2
1	1	0		1	1	1	0	0	0

1	1	0		1	1	1	0	0	0
4	3	1	1	1	2	1	1	0	2
1	1	0		1	1	1	0	0	0
1	1	0		1	1	1	0	0	0
1	1	0		1	1	1	0	0	0
1	1	0		1	1	1	0	0	0
1	1	0		1			0	0	0
1	1	0		3	1		0	2	0
1	1	0		1	1	1	0	0	0
1	1	1	1	1	1	1	0	0	0
1	1	1	1	1	1	1	0	0	0
1	1	0		1	1	1	0	0	0
1	1	0		1	1	1	0	0	0
1	1	0		4	4	4	1	0	0
1	1	0		1	1	1	0	0	0
1	1	0		1	1	1	0	0	0
1	1	0		1	1	1	0	0	0
1	1	0		1	1		0	0	0
1	1	0		1	1		0	0	0
1	1	0		1	1	1	0	0	0
1	1	0		1	1	1	0	0	0
1	3	0		1	1	1	0	0	0
1	1	0		1	4	1	0	0	0
4	1	1	1	4	4	4	1	0	0
3	1	1	1	1	3	1	1	0	0
1	1	0		1	1	1	0	0	0
1	1	0		4	1	1	0	2	2
1	1	0		1	1	1	0	2	2
1	1	0		1	1	1	0	0	0
1	1	0		1	1	1	0	0	0
1	1	0		1	1	1	0	0	0
1	1	0		1	1	1	0	0	1
1	1	0		1	1	1	0	0	1
1	1	0		1	1	1	0	0	0
1	1	0		4	4	1	0	0	0
1	1	0		1	1	1	0	0	0
1	1	0		1	1	1	0	0	0
1	1	0		1	1	1	0	0	0
4	1	1	0	4	4	1	1	0	0
2	1	0		1	1	3	1	2	2
1	1	0		1	1	1	0	0	0
1	1	0		1	1	1	0	0	0
1	1	0		1	1	1	0	0	0
1	1	0		1	1	1	0	0	0
1	1	0		1	1	1	0	0	0
1	1	0		1	1	1	0	0	0
1	1	0		1	1	1	0	1	0

1	1	0		1	1	1	0	0	0
1	1	0		1			0	0	0
1	1	0		1	1	1	0	0	0
1	1	0		1	1	1	0	0	0
		0					0	0	0
1	1	0		1	1	1	0	0	0
1	1	0		1	1	1	0	0	0
1	1	0		1	1	1	0	0	0
1	1	0		1	1	1	0	0	0
1	1	0		1	1	1	0	0	0
1	1	0		1	1	1	0	0	0
2	1	0		3	1	1	1		
1	1	0		1	1	1	0	0	0
1	1	0		1	1	1	0	0	0
1	1	0		1	1	1	0	0	0
1	1	0		1	1	1	0	0	1
1	1	1	1	1	1	1	0	0	0
1	1	0		1	1	1	0	1	
1	1	0		1	1	1	0	0	2
1	1	0		1	1	1	0	0	0
1	1	0		1	1	1	0	0	0
1	1	0		1	3	1	0	0	0
1	1	1	1	1	1	1	0	0	0
1	1	0		1	1	1	0	0	0
1	1	0		1	1	1	0	0	2
1	3	1	1	4	4	2	1	1	2
1	1	0		1	1	1	0	0	0
1	1	0		1	1	1	0	0	0
1	1	0		1	1	1	0	0	0
2	1	1	1	1	1	1	0	0	0
1	1	1	0	1	1	1	0	0	0
1	1	0		1			0	0	0
1	1	0		1	1	1	0	0	0
1	1	0		1	1	1	0	0	0
1	1	0		1	1	1	0	2	1
3	1	1	1	1	4	1	1	0	0
1	1	0		2	1	1	0	0	0
1	1	0		3	1	1	0	0	0
1	1	0		1	1	1	0	0	2
1	1	0		1	1	1	0	0	0
1	1	0		1	1	1	0	0	0
3	1	1	0	1	1	1	0	0	0
1	1	0		1	1	1	0	0	0
1	1	1	1	1	1	1	0	0	0
1	1	0		1	1	1	0	0	2
3	3	0		1	1	1	0	0	0
1	1	0		1	1	1	0	0	0

1	1	0		1	1	1	0	0	0
1	1	0		1	1	1	0	0	0
3	2	1	1	2	2	1	1	1	2
1	1	1	0	1	3	1	0	0	0
1	1	0		1	1	1	0	0	0
1	1	0		1	1	1	0	0	0
1	1	0		1	1	1	0	0	0
1	1	0		1	1	1	0	0	0
1	1	0		1	1	1	0	0	0
1	1	0		1	1	1	0	0	0
1	1	0		1	1	1	0	0	0
1	1	0		1	1	1	0	0	0
1	1	1	1	1	1	1	0	0	0
1	1	0		1	1	1	0	0	0
1	1	0		1	1	1	0	0	0
1	1	0		1	1	2	0	0	1
1	1	0		1	1	1	0	0	0
1	1	0		1	1	1	0	0	0
1	1	1	1	1	3	1	0	0	0
4	1	1	0	1	1	1	0	0	0
1	1	0		1	1	1	0	0	0
1	1	0		1	1	1	0	0	0
1	1	0		1	1	1	0	0	0
1	1	0		1	1	1	0	0	0
1	1	0		1	1	1	0	0	0
1	1	0		1	1	1	0	0	0
		9						0	0
1	1	0		1	1	1	0	0	0
1	1	0		1	1	1	0	0	0
1	1	1	0	1	1	1	0	0	0
1	1	0		3	3	1	1	0	0
1	1	0		1	1	1	0	0	0
1	1	0		1	1	1	0	0	0
1	1	0		1	1	1	0	0	0
3	3	0		1	1	1	0	1	0
1	1	0		3	3	3	1	0	0
1	1	1	1	3	3	3	1	0	0
1	1	0		1	1	1	0	0	0
1	1	0		1	1	1	0	0	0
1	1	9		1	1	1	0	0	0
1	1	0		1	1	1	0	0	0
1	1	0		1	1	1	0	0	0
1	1	0		1	1	1	0	0	0
2	2	1	1	4	4	3	0	2	0
1	1	0		1	1	1	0		0
1	1	0		1	1	1	0	0	0
1	1	0		1	1	1	0	0	0

1	1	0		4	1	2	0	1	0
2	1	0		1	2	1	0	0	0
1	1	0		1	1	1	0	0	0
1	1	0		1	1	1	0		
1	1	0		1	1	1	0	0	0
1	1	0		1	1	1		0	0
1	1	0		1	1	1	0	0	0
1	1	0		1	1	1	0	0	0
2	1	1	1	1	1	1	0	0	0
3	2	0		3	3	3	0	1	1
2	1	1	1	1	1	1	0	1	1
1	1	0		1	1	1	0	0	0
3	1	1	1	1	1	1	0	0	0
1	1	0		1	3	1	1	0	0
1	1	0		1	1	1	0	0	0
1	1	1	0	4	1	1	1	0	
1	1	0		1					
1	1	0		1	1	1	0	0	
4	1	1	1	1	1	1	0	0	0
1	1	0		1	1	1	0	0	0
1	1	1	1	1	1	1	0	0	0
4	1	1	1	3	1	1	1	0	0
4	4	0		1	1	1	0	0	0
1	1	1	1	1	1	1	0	0	0
1	1	0		1				0	0
4	1	0		1	1	1	0	0	0
4	1	1	1	3	4	1	1	0	0
1	1	1	1	1	1	1	1	0	0
1	1	0		1	1	1	0	0	0
1	1	0		1	1	1	0	0	0
1	1	0		2	3	1	1	0	0
1	1	0		1	1	1	0	0	0
1	1	0		1	1	1	0		
1	1	0		1	1	1	0	0	0
1	1	0		1	1	1	0	0	0
1	1	1	0	2	1	1	1	0	0
1	1	1	1	1	1	1	0	0	0
1	1	0		1	1	1	0	0	0
1	1	0		1	1	1	0	0	0
2	1	1	0	4	4	1	1	2	0
1	1	0		1	1	1	0		
4	1	1	1	1	1	1	0	0	0
4	2	0		3	2	2	0	0	0
1	1	0		1	1	1	0	0	0
1	1	0		1	1	1	0	0	0
1	1	0		1	1	1	0	0	0
1	1	0		1	1	1	0	0	0

1	1	0		1	1	1	0	0	0
1	1	0		1	1	1	0		
1	1	1	1	1	1	1	0	0	0
1	1	1	1	1	1	1	0	0	0
2	2	1	1	1	1	1	0	0	0
1	1	0		1	1	1	0	0	0
1	1	0		1	1	1	0	0	0
1	1	0		1	1	1	0	0	0
1	1	0		1	1	1	0	0	0
1	1	0		1	1	1	0	0	0
1	1	0		1	1	1	0	0	0
4	4	1	1	1	1	1	0	0	0
1	1	0		1	1	1	0	0	0
1	1	0		1	1	1	0	0	0
1	1	0		1	1	1	0	0	0
1	1	0		1	1	1	0	0	0
1	2	1	0	1	1	2	1	0	0
1	1	1	1	1	1	1	0	0	0
2	1	1	1	1	1	1	0	0	0
1	1	0			1	1	0	0	0
1	1	1	1	3	3	1	1	0	0
1	1	0		1	1	1	0	0	0
1	1	0		1	1	1	0	0	0
1	1	0		1	1	1	0	0	0
1	1	0		1	1	1	0	0	0
1	1	0		1	1	1	0	0	0
3	1	1	1	1	1	1	0	0	0
4	1	1	0	3	3	1	1	0	0
1	1	0		1	1	1	0	0	0
1	1	0		1	1	1	0	0	0
1	1	0		1	1	1	0	0	0
1	1	0		1	1	1	0	0	0
1	1	0		1	1	1	0	0	0
1	1	0		1	1	1	0	0	0
1	1	0		3	1	1	1	0	0
4	1	0		1	1	1	0	0	2
1	1	1	0	1	1	1	0	0	0
4	4	1	1	3	3	3	1	0	0
1	1	0		1	1	1	0	0	0
1	1	0		1	1	1	0	0	0
1	1	0		1	1	1	0	0	0
1	1	0		4	4	1	1	1	0
1	1	1	1	4	4	1	1	1	0
1	1	0		1	1	1	0	0	0
4	1	0		4	1	1	0	2	
1	1	0		3	1	1	0	0	0
1	1	0		1	1	1	0	0	0

1	1	1	1	2	1	2	1	0	0
1	1	0		1	1	1	0	0	0
1	1	0		1	1	1	0	0	0
1	1	0		3	2	1	1	0	0
2	1	1	1	1	1	1	0	0	0
1	1	1	0	1	1	1	0	0	0
1	1	0		1	1	1	0	0	0
3	1	0		1	1	1	0	0	0
1	1	0		1	1	1	0	0	0
1	1	0		1	1	1	0	0	0
1	1	0		1	1	1	0	0	0
1	1	1	0	4	1	1	1	0	0
1	1	0		1	1	1	0	2	2
1	1	0		1	1	1	0	0	0
1	1	0		1	1	1	0	0	0
1	1	0		1	1	1	0	0	0
4	1	1	0	1	1	1	0	0	0
1	1	0		1	1	1	0	0	0
1	1	0		1	1	1	0	0	0
1	1	0		1	1	1	0	0	0
1	1	0		3	3	2	1	0	0
1	1	0		1	1	1	0	0	0
1	1	0		1	1	1	0	0	0
1	1	0		1	1	1	0	0	0
1	1	0		1	1	1	0	0	0
1	1	1	1	3	3	1	1	0	0
1	1	0		1	1	1	0	0	0
1	1	0		3	3	1	1	0	0
1	1	0					0	0	0
1	1	0		1	1	1	0	0	0
3	1	1	0	3	3	1	1	0	0
1	1	0		1	1	1	0	0	0
1	1	0		3	1	1	0	1	
1	1	0		2	2	1	1	0	0
1	1	0		1	1	1	0	0	0
1	1	0		1	1	1	0	0	0
1	1	0		1	1	1	0	0	0
1	1	0		1	1	1	0	0	0
3	1	1	1	1	1	1	0	0	0
1	1	0		1	1	1	0	0	0
1	1	0		1	1	1	0	0	0
4	3	1	1	1	1	1	0	0	0
2	1	0		1	1	1	0	0	0
1	1	0		1	1	1	0	0	0
1	1	0		1	2	2	1	0	0
1	1	0		1	1	1	0	0	0
1	1	0		1	1	1	0	0	0

1	1	0		1	1	1	0	0	0
1	1	0		1	1	1	0	0	0
1	1	0		1	1	1	0	0	0
1	1	0		1	1	1	0	0	0
1	1	0		1	1	1	0	0	0
1	1	0		1	1	1	0	0	0
4	4	1	1	1	1	1	0	0	0
1	1	0		1	1	1	0	0	0
3	3	1	0	1	1	1	0	0	0
1	1	0		1	1	1	0	0	0
1	1	0		1	1	1	0	0	0
1	1	0		1	1	1	0	0	0
1	1	0		1	1	1	0	0	0
		9						2	1
1	1	0		1	1	1	0	0	0
1	1	0		1	1	1	0	0	0
4	1	1	1	1	4	1	0	0	0
1	1	0		1	1	1	0	0	0
1	1	0		1	1	1	0	0	0
1	1	0		1	1	1	0	0	0
1	1	0		1	1	1	0	0	2
1	1	0		1	1	1	0	0	0
1	1	0		1	1	1	0	0	0
2	1	1	0	2	1	3	1	0	2
1	1	0		1	1	1	0	0	0
1	1	0		1	1	1	0	0	0
1	1	0		1	1	1	0	0	2
1	1	0		1	2	1	0	0	2
1	1	1	1	2	3	1	1	0	0
1	1	0		1	1	1	0	0	0
1	1	0		1	1	1	0	0	0
1	1	0		1	1	1	0	0	0
1	1	0		2	1	1	0	0	1
1	1	0		1	2	1	0	1	2
4	4	1	1	1	1	1	0	0	2
1	1	0		1	1	1	0	0	0
1	1	0		1	1	1	0	0	1
1	1	0		1	1	1	0	0	0
1	1	0		2	1	1	1	0	0
1	1	0		1	1	1	0	0	0
1	1	0		3	2	1	1	0	0
1	1	0		1	1	1	0	0	0
1	1	0		1	1	1	0	0	0
1	1	0		1	1	1	0	0	0
1	1	1	1	1	1	1	0	0	0
1	1	0		1	1	1	0	0	0
3	1	0		1	1	1	0	0	0

2	1	0		1	1	1	0	0	0
1	1	1	0	1	1	1	0	0	0
1	1	0		1	1	1	0	0	0
1	1	1	1	1	1	1	0	0	0
1	1	0		1	1	1	0	0	0
1	1	0		1	1	1	0	0	0
1	1	1	0	1	1	1	0	0	0
1	1	0		1	1	1	0	0	2
1	1	0		1	1	1	0	0	0
1	1	0		1	1	1	0	0	0
1	1	0		1	1	1	0	0	0
1	1	0		1	1	1	0	0	0
1	1	0		1	3	1	1	0	0
1	1	0		1	1	1	0	0	0
1	1	0		1	1	1	0	0	0
4	1	1	1	4	4	1	1	0	0
		9						0	0
1	1	0		1	1	1	0	0	0
1	1	0		1	1	1	0	0	0
1	1	1	0	1	3	1	1	0	0
1	1	0		1	1	1	0	0	0
1	1	0		1	1	1	0	0	0
1	1	0		1			0	0	0
2	1	1	0	1	1	1	0	0	0
1	1	1	0	1	1	1	0	0	0
1	1	0		1	1	1	0	0	0
1	2	1	0	1	1	1	0	0	0
1	2	0		1	1	1	0	0	0
1	1	0		1	1	1	0	0	0
1	1	0		1	1	1	0	0	0
4	1	1	1	1	1	1	0	0	0
1	1	0		1	1	1	0	0	0
1	1	0		1	1	1	0	0	0
1	3	0		4	1	1	0	0	0
1	1	0		1	1	1	0	0	0
1	1	0		1	1	1	0	0	0
1	2	1	0	1	1	1	0	0	0
1	1	0		1	1	1	0	0	0
4	1	1	1	1	1	1	0	0	0
1	1	0		1	1	1	0	0	0
1	1	0		1	4	1	0	0	0
2	1	0		1	1	1	0	0	0
1	1	1	1	1	1	1	1	0	0
1	1	0		1	1	1	0	0	0
1	1	0		2	2	1	0	0	0
1	1	0		1	1		0	0	0
4	1	1	1	1	1	1	0	0	0

1	1	0		1	1	1	0	0	1
1	1	0		1	1	1	0	0	0
1	1	0		1	1	1	0	0	0
1	1	0		1	1	1	0	0	0
1	1	0		1	1	1	0	0	0
1	1	0		1	1	1	0	0	0
1	1	0		1	1	1	0	0	0
1	1	1	1	1	1	1	0	0	0
1	1	0		1	1	1	0	0	0
3	1	1	1	3	3	1	1	0	0
1	1	0		1	1	1	0	0	0
1	1	0		1	1	1	0	0	0
1	1	0		1	1	1	0	0	0
3	1	0		2	3	2	1	0	0
1	1	0		1	1	1	0	0	0
1	1	0		1	1	1	0	0	0
1	1	0		1	1	1	0	0	0
1	1	0		1	1	1	0	0	0
1	1	0		1	1	1	0	2	2
1	2	0		1	1	1	0	0	0
1	1	0		1	1	1	0	0	0
1	1	0		1	1	1	0	0	0
1	1	0		1	1	1	0	0	0
1	1	0		1	1	1	0	0	0
1	1	0		1	1	1	0	0	0
1	1	1	1	4	4	3	1	1	2
		0		1	1	1	0	0	0
1	1	0		1	4	1	1	0	0
4	4	0		1	4	1	0	0	0
1	1	0		1	1	1	0	0	0
1	1	0		1	1	1	0	1	0
1	1	0		1	1	1	0	0	0
1	1	0		1	1	1	0	0	0
1	1	0		1	1	1	0	0	0
1	1	1	0	1	1	1	0	0	0
1	1	0		1	1	1	0	0	0
1	1	0		1	1	1	0	0	0
2	1	1	0	1	1	1	0	0	0
1	1	0		1	1	1	0	0	0
1	1	0		1	1	1	0	0	0
1	1	0		3	3	3	1	2	0
1	1	0		1	1	1	0	0	0
1	1	0		1	3	1	1	0	0
2	1	1	0	1	1	1	0	0	0
1	1	0		1	1	1	0	0	0
1	1	1	0	1	1	1	0	0	0
1	1	0		1	1	1	0	0	0

1	1	0		1	1	1	0	0	0
1	1	0		1	1	1	0	0	0
1	1	0		1	2	1	0	0	0
1	1	0		1		1	0	0	0
4	4	1	1	4	4	4	1	0	0
1	1	1	1	1	1	1	0	0	0
1	3	1	1	1	1	1	0	0	0
1	3	1	1	1	1	1	0	0	0
1	1	0		1	1	1	0	0	0
1	1	0		1	1	1	0	0	0
1	1	0		1	1	1	0	0	0
1	1	0		1	1	1	0	0	0
1	1	0		1	1	1	0	0	0
1	1	1	0	1	1	1	0	0	0
1	1	0		1	1	1	0	0	0
1	1	0		1	1	1	0	0	0
1	1	0		1	1	1	0	0	0
1	1	0		1	1	1	0	0	0
1	1	1	0	1	1	1	0	0	0
1	1	0		3	1	1	0	0	0
1	1	0		1	1	1	0	0	0
1	1	1	1	1	3	1	1	0	2
1	1	0		1	1	1	0	0	0
1	1	0		1	1	1	0	0	0
1	1	0		1	1	1	0	0	0
3	4	1	1	4	1	1	0	0	0
1	1	1	0	1	1	1	0	0	0
1	1	0		1	1	1	0	1	2
1	1	0		1	1	1	0	0	0
1	1	0		1	1	1	0	0	0
1	1	0		1	1	1	0	0	0
1	1	0		1	1	1	0	0	0
1	1	0		1	1	1	0	0	0
1	1	0		1	1	1	0	0	0
1	1	0		3	1	1	0	0	0
1	1	0		3	1	1	0	0	0
1	1	0		1	1	1	0	0	0
1	1	0		1	1	1	0	0	0
1	1	0		1	1	1	0	0	0
1	1	0		1	1	1	0	0	0
1	1	0		1	1	1	0	0	0
1	1	1	0	1	1	1	0	0	0
3	3	1	1	1	1	1	0	1	1
1	1	0		1	1	1	0	0	0
1	1	1	0	1	1	1	0	0	0
1	1	0		1	1	1	0	0	0
1	1	0		1	1	1	0	0	0

1	1	0		1	1	1	0	0	0
1	1	0		3		3	1	0	0
1	1	0		1	1	1	0	0	0
1	1	0		1	1	1	0	0	0
1	1	0		1	1	1	0	0	0
1	1	0		1	1	1	0	0	0
1	1	0		1	1	1	0	0	0
1	1	0		1	1	3	1	0	0
3	1	1	1	1	1	1	0	0	0
1	1	1	1	1	1	1	0	0	0
3	3	1	1	1	1	1	0	2	2
3	3	1	1	1	1	1	0	2	2
1	1	0		1	1	1	0	0	0
1	1	0		4	1	4	1	0	0
4	4	1	1	4	4	4	1	0	0
1	1	1	1	1	1	1	0	0	0
1	1	0		1	1	1	0	1	2
4	1	1	1	1		1	0	0	0
3	1	0		1	1	1	0	0	0
2	1	1	1	3	4	1	1	0	0
1	1	0		1	3	1	1	0	0
1	1	0		1	1	1	0	0	0
1	1	0		1	1	1	0	0	0
1	1	0		1	1	1	0		
1	1	0		1	1	1	0		
1	1	0		1	1	1	0	0	0
3	3	0		1	1	1	0	0	0
1	1	0		3	1	1	0	0	0
2	1	1	1	1	1	1	0	0	0
1	1	0		1	1	1	0	0	0
1	1	0		1	1	1	0	0	0
1	1	0		1	1	1	0	0	0
1	1	0		1	1	1	0	0	0
1	1	0		1	1	1	0	0	0
1	1	1	0	1	1	1	0	0	0
1	1	0		3	1	2	1	0	0
1	1	0		3	1	2	1	0	0
1	1	0		1	1	1	0	0	0
1	1	0		1	1	1	0	0	0
1	1	0		1	1	1	0	0	2
1	1	0		1	1	1	0	0	0
1	1	0		1	1	1	0	0	0
1	1	0		1	2	2	0	0	0
1	1	0		1	1	1	0	0	0
1	1	0		1	1	1	0	0	0
1	1	0		1	1	1	0	0	0
1	1	0		1	1	1	0	0	0

1	1	0		1	1	1	0	0	2
1	1	0		1	1	1	0	0	0
1	1	0		1	3	1	0	0	1
1	1	0		1	1	1	0		
1	1	0		1	1	1	0	0	0
1	1	0		1	1	1	0	0	0
1	1	0		3	1	1	0	0	0
1	1	0		3	1	1	1	0	0
1	1	0		3	1	1	0	0	0
3	1	1	0	1	1	1	0	0	0
3	3	1	1	1	1	1	0	0	0
1	1	0		1	1	1	0	0	0
1	1	0		1	1	1	0	0	0
3	3	0		3	3	3	0	2	
4	4	1	1	4	4	4	1	2	
1	1	0		1	1	1	0	0	0
3	1	0		1	1	1	0	0	0
2	2	0		1	1	1	0	0	0
1	1	0		1	1	1	0	0	0
1	1	0		1	1	1	0	0	0
1	1	9		1	1	1	0	0	0
1	1	0		1	1	1	0	0	0
1	1	0		1	1	1	0	0	0
1	1	1	0	1	1	1	0	0	0
1	2	0		1	1	1	0	0	0
1	1	0		1	1	1	0	0	0
1	1	0		1	1	1	0	0	0
1	1	0		1	1	1	0	0	0
1	1	0		1	1	1	0	1	
1	1	0		1	1	1	0	0	0
4	4	1	1						
1	1	0		2	1	1	0	0	0
1	1	1	0	4	1	1	0	0	0
1	1	0		1	1	1	0	0	0
1	1	0		1	1	1	0	0	0
1	1	0		1	1	1	0	0	0
1	1	0		1	1	1	0	0	0
1	1	0		2	3	3	0	0	0
1	1	0		1	1	1	0	0	0
1	1	0		1	1	1	0	0	0
1	1	0		1	1	1	0	0	0
1	1	9		1	1	1		0	0
3	3	0		1	1	1	0	0	0
1	1	0		1	1	1	0	0	0
3	3	1	0	1	1	1	0	0	0
1	1	0		1	1	1	0	0	0
1	1	0		1	1	1	0	0	0

1	1	0		1	1	1	0	0	0
1	1	0		3	3	2	0	0	0
1	1	0		1	1	1	0		
1	1	0		1	1	1	0	0	0
1	1	0		1	1	1	0		
1	1	0		1	1	1	0	0	0
3	1	1	1	1	1	1	0	0	0
4	4	0		4	4	4	1	0	0
3	3	0		3	1	1	0	0	0
1	1	0		1	3	1	1	0	0
4	4	1	1	4	1	1	1		
1	1	1	0	1	1	1	0		
1	1	1	1	3	4	1	1	0	0
4	4	0		1	1	1	0	0	0
1	1	0		1	1	1	0	0	0
1	1	1	0	1	1	1	0		
1	1	0		4	1	1	0	0	0
1	1	0		1	1	1	0	1	1
1	1	0		2	2	2	1	0	
1	1	0		1	1	1	0	0	0
1	1	1	1	2	1	1	1	0	0
1	1	0		1	1	1	0	0	0
1	1	0		1	1	1	0	0	0
1	1	0		1	3	1	1	0	0
1	1	0		1	1	1	0	0	0
1	1	0		1	1	1	0	0	0
1	1	0		1	1	1	0	0	0
1	1	0		1	1	1	0	0	0
4	4	1	1	1	1	1	0	0	0
3	3	0		3	3	1	0	0	0
2	1	1	1	3	1	3	1	0	0
1	1	0		1	1	1	0	0	0
1	1	0		1	1	1	0	0	0
1	1	0		1	3	1	1	0	0
4	4	0		1	1	1	0	0	0
3	2	0		1			0	0	
4	4	1	1	1					
1	1	0		1	1	1	0	2	2
1	1	0		1	1	1	0	0	0
1	1	0		1	3	1	1	0	0
1	1	1	1	1	4	1	1	0	0
1	1	1	1	1	4	1	1	0	0
1	1	0		1	1	1	0	0	0
1	1	1	0	1	4	1	1	0	0
1	1	1	0	1	1	1	0	0	0
4	1	1	1	1	1	1	0	0	0
1	1	1	0	1	1	1	0	0	0

1	1	0		3	1	1	0	0	0
1	3	1	0	1	1	1	0	0	0
1	1	0		1	3	1	1	0	0
1	1	1	1	1	1	1	0	0	0
1	1	0		1	1	1	0	0	0
1	1	0		1	1	1	0	0	0
1	1	0		1	1	1	0	0	0
1	1	0		1	1	1	0	0	0
3	1	1	1	1	1	1	0	0	0
1	1	0		1	1	1	0	0	0
1	1	0		1	1	1	0	0	0
1	1	0		1	1	1	0	0	0
3	3	1	1	1	1	1	0	1	0
3	1	1	1	1	1	1	0	0	0
1	1	0		3	1	1	1	0	0
1	1	0		1	1	1	0	0	0
1	1	0		1	1	1	0	0	0
1	1	0		1	1	1	0	0	0
2	1	0		1	1	1	0	0	0
2	1	0		1	1	1	0	0	0
1	1	0		1	1	1	0	0	0
1	1	0		1	1	1	0	0	0
2	1	1	1	1	1	1	0	0	0
3	1	1	0	1	1	1	0	0	0
1	1	0		1	1	1	0	0	0
1	1	1	1	1	1	1	0	0	0
1	1	0		1	1	1	0	0	0
1	1	0		1	1	1	0	0	0
3	1	1	1	1	1	1	0	0	0
4	4	1	1	1	1	1	0	0	0
2	3	1	1	1	1	1	0	0	0
4	1	1	1	1	1	1	0	0	0
1	1	0		1	1	1	0	0	0
1	1	0		1	1	1	0	0	0
1	1	0		1	1	1	0	0	0
1	1	0		1	1	1	0	0	0
1	1	0		1	1	1	0	0	0
1	1	0		2	1	1	0	0	0
1	1	0		1	1	1	0	0	0
2	2	1	1	1	1	1	0	0	0
2	2	1	1	1	1	1	0	0	0
1	1	0		2	1	1	0	0	0
1	1	0		1	1	1	0	0	0
1	1	1	0	1	1	1	0	0	0
1	1	0		1	1	1	0	0	0
3	1	1	1	1	1	1	0	0	0
1	1	0		1	1	1	0	0	0

3	3	1	1	3	1	1	0	0	0
3	3	1	1	3	1	1	0	0	0
2	1	0		1	1	1	0	0	0
1	1	1	1	1	1	1	0	0	0
1	1	0		1	1	1	0	0	0
4	1	1	0	4	1	1	1	0	0
3	1	1	1	1	1	1	0	0	0
1	1	0		1	1	1	0	0	0
4	4	0		1	1	1	0	0	0
4	4	1	1	1	1	1	0	0	0
1	1	1	1	1	1	1	0	0	0
1	1	1	0	1	1	1	0	0	0
4	3	1	0	3	1	4	1	0	1
3	1	1	1	2	3	3	1	0	0
3	1	1	1	2	3	3	1	0	0
1	1	0		1	1	1	0	0	0
1	1	0		4	4	1	1	0	0
1	1	0		3	1	1	1	0	0
1	1	0		1	1	1	0	0	0
1	1	0		3	1	1	1	0	0
1	1	0		1	1	1	0	0	0
1	1	0		3	3	1	1	0	0
1	1	0		1	1	1	0	0	0
1	1	0		1	1	1	0	0	0
3	4	1	1	4	3	3	1	0	0
1	1	0		3	1	1	1	0	0
1	1	0		1	1	1	0	0	0
1	1	0		1	1	1	0	0	0
2	1	0		1	1	1	0	0	0
1	1	1	0	1	1	1	0	0	0
1	1	0		4	3	1	1	0	0
1	1	0		1	1	1	0	2	2
1	1	1	0	1	1	1	0	0	0
1	1	1	1	1	4	1	1	0	0
1	1	0		1	1	1	0	0	0
1	1	0		1	1	1	0	0	0
1	1	0		1	1	1	0	0	0
1	1	0		1	1	1	0	0	0
1	1	0		3	3	1	1	0	0
2	1	0		1	1	1	0	0	0
3	3	1	1	3	1	1	1	0	0
1	1	1	1	3	3	1	1	0	0
1	1	1	1	3	3	1	1	0	0
1	1	0		1	1	1	0	0	0
1	1	0		1	1	1	0	0	0
1	1	0		1	1	1	0	0	0
1	1	1	1	1	3	4	1	0	0

1	1	1	1	1	3	4	1	0	0
1	1	0		1	1	1	0	0	0
1	1	1	1	3	1	1	0	0	0
1	1	1	1	2	1	3	1	2	2
1	1	0		1	1	1	0	0	0
1	1	0		1	1	1	0	0	0
1	1	0		1	1	1	0	0	0
1	1	0		1	1	1	0	0	0
3	1	1	1	1	1	1	0	0	0
1	1	0		1	1	2	1	0	0
1	1	0		1	1	1	0	0	0
1	1	0		1	3	1	1	0	0
1	1	0		3	1	1	1	0	2
1	1	0		1	1	1	0	0	0
1	1	1	1	3	3	4	1	0	2
1	1	0		1	1	1	0	0	0
1	1	0		1	1	1	0	0	1
1	1	0		1	1	1	0	0	0
1	1	0		3	2	1	1	0	0
1	1	1	1	4	1	1	1	0	0
1	1	0		1	1	1	0	0	0
1	1	0		1	1	1	0	0	2
1	1	0		1	1	1	0	0	0
1	1	0		1	1	1	0	0	0
4	4	1	0	1	1	1	0	0	0
1	3	1	0	1	1	1	0	0	0
1	1	0		1	1	1	0	0	0
1	1	0		1	1	1	0	0	0
2	1	1	1	1	1	1	0	0	0
3	1	1	1	3	2	1	1	0	0
4	1	1	0	1	3	1	0	0	1
1	1	0		1	1	1	0	0	0
1	1	1	0	3	3	1	1	0	0
1	1	1	0	1	2	1	1	0	0
2	1	1	0	1	1	1	0	0	0
1	1	0		1	1	1	0	0	1
1	1	0		1	1	1	0	0	0
1	1	0		1	1	1	0	0	0
1	1	0		1	1	1	0	0	0
4	1	1	1	1	1	1	0	0	0
1	1	0		1	1	1	0	0	0
1	1	0		1	1	1	0	0	0
1	2	0		1	1	1	0	0	0
1	1	0		2	2	1	1	1	1
1	1	0		1	1	1	0	0	0
1	1	0		2	2	1	0	0	0
1	1	0		2	3	1	1	0	0

1	1	0		1	1	1	0	0	0
1	1	0		1	1	1	0	0	0
1	1	0		1	1	1	0	0	0
1	1	0		1	1	1	0	0	0
1	1	0		1	1	1	0	0	0
3	1	1	0	3	4	1	1	0	0
1	1	0		1	1	1	0	0	0
3	1	0		1	1	1	0	0	0
1	1	0		1	1	1	0	0	0
1	1	1	0	1	1	1	0	0	0
3	1	1	1	1	1	1	0	0	0
1	1	1	0	1	1	1	0	0	0
1	1	0		2	2	3	1	0	0
1	1	0		1	1	1	0	0	0
1	1	0		1	1	1	0	0	0
1	1	0		4	3	1	1	0	0
1	1	0		1	1	1	0	0	0
3	1	1	0	3	1	1	0	0	0
1	1	0		1	1	1	0	2	2
1	1	0		1	1	1	0	0	0
1	1	0		1	1	1	0	0	0
1	1	0		1	1	1	0	0	0
1	1	0		1	1	1	0	0	0
1	1	0		3	3	1	0	0	0
1	1	0		1	1	1	0	0	0
3	1	0		3	1	1	0	0	0
3	1	0		1	1	1	0	0	0
1	1	0		1	1	1	0	0	0
3	1	1	1	2	2	1	1	0	0
1	1	0		1		1	0	2	2
1	1	0		1	1	1	0	0	0
3	1	1	1	1	1	2	1	0	0
1	1	0		1	1	1	0	0	0
1	1	0		1	1	1	0	0	0
1	1	0		1	1	1	0	0	0
1	1	0		2	1	1	1	0	0
1	1	0		1	1	1	0	0	0
1	1	0		1	1	1	0	0	1
1	1	0		2	1	1	0	0	0
1	1	0		1	1	1	0	0	0
1	1	0		3	1	1	0	0	0
1	1	0		1	1	1	0	0	0
1	1	0		1	1	1	0	0	0
2	1	1	1	3	1	1	1	0	0
3	1	1	1	1	1	1	0	0	0
1	1	0		1	1	1	0	0	0
1	1	0		1	1	1	0	0	0

1	1	0		1	1	1	0	0	0
1	1	1	1	1	1	1	0	0	0
1	1	0		1	1	1	0	0	0
1	1	0		1	1	1	0	0	0
1	1	1	0	1	1	1	0	0	0
1	1	0		3	3	1	1	0	0
2	1	1	0	1	1	1	0	1	1
1	1	0		1	1	2	1	0	0
1	1	0		1	1	1	0	0	0
1	1	0		1	1	1	0	0	0
1	1	0		2	2	2	1	0	0
1	1	0		1	1	1	0	0	0
1	1	0		1	1	1	0	0	0
1	1	0		1	1	1	0	0	0
1	1	0		1	2	1	0	0	0
1	1	0		1	1	1	0	0	0
1	1	0		1	1	1	0	0	0
1	1	0		1	1	1	0	0	0
1	1	0		1	1	1	0	0	0
1	1	0		1	1	1	0	0	0
1	1	0		1	1	1	0	0	0
1	1	0		1	1	1	0	2	0
1	1	0		1	1	1	0	0	0
1	1	0		1	1	1	0	0	0
1	1	0		1	1	1	0	0	0
1	1	0		1	1	1	0	0	0
1	1	0		4	4	1	0	0	0
1	1	0		1	1	1	0	0	0
1	1	0		1	1	1	0	0	0
1	1	0		1	1	1	0	0	0
1	1	1	0	1	1	1	0	0	0
1	1	0		1	1	1	0		
1	1	0		1	1	1	0	0	0
4	4	1	1	1	1	1	0	0	0
3	1	1	1	1	1	1	0	0	0
1	1	0		1	1	1	0	0	0
1	1	0		1	1	1	0	0	0
1	1	0		1	1	1	0	0	0
1	1	1	1	1	1	1	0	0	0
1	1	0		1	1	1	0	2	2
1	1	0		1	1	1	0	0	0
1	1	0		1	1	1	0	0	0
1	1	0		1	1	1	0	0	2
1	1	0		1	1	1	0	0	0
1	1	0		1	1	1	0	0	0
1	1	0		1	1	1	0	0	0
1	1	0		1	1	1	0	0	0

1	1	0		1	1	1	0	2	1
4	1	1	1	1	1	1	0	0	0
1	1	0		1	1	1	0	0	1
1	1	0		1	1	1	0	0	0
1	1	0		1	1	1	0	0	0
1	1	0		1	1	1	0	1	0
1	1	0		1	1	1	0	0	1
1	1	0		1	1	1	0	0	0
1	1	0		1	1	1	0	0	2
1	1	0		2	1	1	1	0	1
3	1	1	1	3	3	1	1	2	0
1	1	0		1	1	1	0	0	0
1	1	0		1	1	1	0	0	0
1	2	0		1	1	1	0	1	0
1	1	0		1	1	1	0	0	0
1	1	0		1	1	1	0	0	0
1	1	0		1	1	1	0	0	0
1	3	1	0	1	1	1	0	0	0
1	1	0		2	2	1	0	0	0
1	1	0		4	1	1	0	0	0
4	1	1	1	1	4	1	0	1	
1	1	0		3	2	1	1	0	0
1	1	0		1	1	1	0	0	0
1	1	0		1	1	1	0	0	0
1	1	0		1	1	1	0	0	0
1	1	0		1	1	1	0	0	0
1	1	0		1	1	1	0	0	1
1	1	0		1	1	1	0	0	1
1	1	0		1	1	1	0	0	0
1	1	0		1	1	1	0	0	0
1	1	0		1	1	1	0	0	0
1	1	0		1	1	1	0	2	2
1	1	0		1	1	1	0	0	0
1	1	0		1	1	1	0	0	0
1	1	0		1	1	1	0	0	0
1	1	0		1	1	1	0	0	0
1	1	0		1	1	1	0	0	0
1	1	0		1	1	1	0	0	0
1	1	0		1	1	1	0	0	0
1	1	0		1	1	1	0	0	0
1	1	0		1	1	1	0	0	0
1	1	0		1	1	1	0	0	0
1	1	0		1	1	1	0	0	0
1	1	1	0	1	1	1	0	0	0
1	1	1	1	1	1	1	0	0	0
1	1	0		1	1	1	0	0	0
1	1	0		1	1	1	0	0	0

1	1	0		1	1	1	0	0	0
1	1	0		1	1	1	0	0	0
1	1	1	1	1	1	1	0	0	0
1	1	0		1	1	1	0	0	0
1	1	0		1	1	1	0	0	0
1	1	0		1	1	1	0	0	0
1	1	0		1	1	1	0	0	0
1	1	0		1	1	1	0	0	0
1	1	0		1	1	1	0	0	0
1	1	0		1	1	1	0	1	1
1	1	0		1	1	1	0	0	1
1	1	1	1	1	1	1	0	0	0
1	1	0		1	1	1	0	0	0
1	1	0		1	1	1	0	0	0
1	1	0		1	3	1	0	0	0
1	1	1	1	1	1	1	0	0	0
1	1	0		1	1	1	0	0	0
3	1	1	1	1	1	1	1	0	0
1	1	0		1	1	1	0	0	0
1	1	0		1	1	1	0	0	0
1	1	0		1	1	1	0	0	0
		9						0	0
1	1	0		1	1	1	0	0	0
1	1	0		1	1	1	0	0	0
1	1	0		1	1	1	0	2	0
1	1	0		1	1	1	0	0	0
1	1	1	0	1	1	1	0	0	0
2	2	1	1	4	1	1	1	0	0
1	1	0		1	1	1	0	0	0
1	1	0		1	1	1	0	0	0
1	1	0		1	1	1	0	2	1
1	1	0		1	1	1	0	0	0
1	1	0		1	1	1	0	0	0
		9						0	0
1	1	0		1	1	1	0	0	0
1	1	1	1	1	1	1	0	0	0
1	1	0		1	1	1	0	0	0
1	1	0		4	4	1	1	0	0
1	1	0		1	1	1	0	0	1
4	2	1	1	4	4	4	1	0	0
1	1	0		1	1	1	0	0	0
1	1	0		1	1	1	0	0	0
1	1	0		1	1	1	0	2	0
1	1	0		1	1	1	0	0	0
1	1	0		1	1	1	1	0	0
1	1	1	0	1	1	1	0	0	0
1	1	0		1	1	1	0	0	0

1	1	0		1	1	1	0	0	0
1	1	0		1	1	1	0	0	0
1	1	0		1	1	1	0	0	0
1	1	0		1	1	1	0	0	0
1	1	0		1	1	1	0	0	0
1	1	0		1	1	1	0	0	0
4	1	1	1	1	4	4	1	0	0
1	1	0		1	1	1	0	0	0
2	1	0		1	1	1	0	1	1
2	1	0		1	1	1	0	0	0
1	1	0		1	1	1	0	0	0
1	1	0		1	1	1	0	0	0
1	1	0		1	1	1	0	1	0
1	1	0		1	1	1	0	0	0
1	1	0		1	1	1	0	0	0
2	1	1	1	1	1	1	0	1	2
2	1	0		1	1	1	0	0	0
1	1	0		1	1	1	0	0	0
3	2	1	1	1	1	1	1	0	0
1	1	0		1	1	1	0	0	0
1	1	0		3	1	1	0	0	0
1	1	0		3	1	1	0	2	1
1	1	0		1	3	1	0	0	0
1	1	0		1	1	3	1	0	0
1	1	0		1	1	1	0	0	0
1	1	0		1	1	1	0	0	0
1	1	0		1	1	1	0	0	0
1	1	0		1	1	1	0	0	0
1	1	0		4	4	1	1	0	0
1	1	0		4	4	1	1	0	0
1	1	0		1	1	1	0	0	0
1	1	1	0	1	1	1	0	0	0
1	1	0		1	1	1	0	0	0
1	1	1	0	4	3	1	0	0	0
1	1	0		1	1	1	0	0	0
1	1	1	1	1	1	1	0	0	0
1	1	0		1	1	1	0	0	0
1	1	0		1	1	1	0	0	0
1	1	0		1	1	1	0	0	0
1	1	0		1	1	1	0	0	0
1	1	0		1	1	1	0	0	2
1	1	0		1	1	1	0	0	0
1	1	0		4	4	3	1	0	0
1	1	0		1	1	1	0	0	0
1	1	0		3	3	3	0	2	0
1	1	0		3	3	3	0	2	0
1	1	0		1	1	1	0	0	0

1	1	0		1	1	1	0	0	0
1	1	0		1	1	1	0	0	0
1	1	0		1	4	1	0	0	0
1	1	0		1	1	1	0	0	0
1	1	0		1	3	3	0	0	0
2	3	1	0	1	4	1	1	0	0
4	4	0		1	1	1	0	0	0
1	1	0		1	1	1	0	0	0
1	1	0		1	1	1	0	0	2
1	1	1	0	1	1	1	0	0	2
1	1	0		1	1	1	0	0	0
1	1	0		1	1	1	0	0	0
1	1	0		1	1	1	0	0	0
1	1	0		1	1	2	0	0	0
4	1	1	0	3	3	1	1	2	2
1	1	1	0	2	2	1	1	1	1
1	1	0		1	1	1	0	0	0
1	1	0		1	1	1	0	0	0
1	1	0		1	1	1	0	0	0
1	1	0		1	1	1	0	0	0
1	1	0		1	1	1	0	0	0
1	1	0		1	3	1	1	0	0
1	1	1	0	1	3	1	1	0	0
1	1	0		1	1	1	0	0	0
4	1	1	1	1	1	1	0	0	0
1	1	0		1	1	1	0	0	0
3	3	0		1	1	1	0	0	0
1	1	0		1	1	1	0	0	0
1	1	0		1	1	1	0	0	0
1	1	0		1	1	1	0	1	1
2	1	1	0	2	2	1	1	0	2
1	1	0		1	1	1	0	0	2
1	1	0		3	1	1	0	0	0
1	1	0		1	1	1	0	0	0
1	1	0		1	1	1	0	0	0
4	4	1	1	1	4	4	1	0	0
1	1	1	0	1	1	1	0	1	2
1	1	0		1	1	1	0	1	0
1	1	0		1	1	1	0	0	0
3	1	1	1	1	1	1	0	0	1
1	1	0		1	1	1	0	0	0
1	1	0		1	1	1	0	2	1
1	1	0		3	1	3	1	0	0
3	2	1	1	3	3	1	1	0	1
3	2	1	1	3	3	1	1	0	1
1	1	0		1	1	1	0	0	0
1	1	0		1	1	1	0	0	0

1	1	0		1	1	1	0	0	0
1	1	0		1	1	1	0	1	1
1	1	1	1	2	2	1	1	2	2
1	1	0		1	1	1	0	0	0
1	1	0		1	1	1	0	0	0
1	1	0		1	1	1	0	0	0
3	2	1	1	3	3	1	1	1	2
3	2	1	1	3	3	1	1	1	2
3	2	1	1	3	3	1	1	1	2
1	1	0		1	1	1	0	0	0
1	1	0		1	1	1	0	0	1
1	1	0		1	1	1	0	0	0
3	1	1	1	3	1	1	1	0	0
1	1	0		1	1	1	0	0	0
1	1	0		1	1	1	0	0	0
1	1	0		1	1	1	0	0	0
1	1	0		1	1	1	0		
1	1	0		1	1	1	0	0	0
1	1	0		1	1	1	0	0	0
1	1	0		1	1	1	0	0	0
1	1	0		1	1	1	0	0	0
1	1	0		1	1	1	0	0	0
1	1	0		1	1	1	0	0	0
2	2	1	0	1	1	1	0		
1	1	0		1	1	1	0	0	0
1	1	0		1	1	1	0	0	0
1	1	9		1	1	1		0	0
1	1	0		1	1	1	0	0	0
2	1	0		3	1	1	0	0	0
2	2	0		1	1	1	1	0	0
1	1	0		1	1	1	0	0	0
1	1	0		1	1	1	0	0	0
1	1	0		1	1	1	0	0	0
1	1	0		4	1	1	1	0	0
1	1	0		1	1	1	0	0	0
1	1	0		1	1	1	0	0	0
1	1	0		1	1	1	0	0	0
1	2	1	1	1	1	1	0	0	0
3	3	0		3	3	3	0	0	0
1	1	9		1	1	1		0	0
1	1	0		1	1	1	0	0	0
2	2	1	1	1	1	1	0	0	0
1	1	0		1	1	1	0	0	0
1	2	0		1	1	1	0	0	0
1	1	0		1	1	1	0	0	0
		9						0	0
1	1	0		1	1	1	0	0	0

1	1	0		1	1	1	0	0	0
1	1	0		3	1	1	0	0	0
1	1	0		1	1	1	0	0	0
4	4	9		1	1	1		0	0
1	1	0		1	1	1	0	0	0
1	1	9		1	1	1	0	0	0
1	1	0		1	1	1	0	0	0
1	1	0		1	1	1	0	0	0
1	1	0		1	1	1	0	0	0
3	1	0		1	1	1	0	0	0
3	1	0		1	1	1	0	0	0
1	1	0		1	1	1	0	0	0
2	2	0		1	1	1	0	0	0
1	1	0		1	1	1	0	0	0
1	1	0		1	1	1	0	0	0
2	2	1	1	1	1	1	0	0	0
2	2	1	0	3	3	1	0	0	0
1	1	0		1	1	1	0	0	0
2	1	0		2	1	2	0	0	0
1	1	9		1	1	1		0	0
1	1	1	0	1	1	1	0	0	0
1	1	0		1	1	1	0	0	0
3	1	1	0	3	3	3	1	0	0
2	1	0		1	1	1	0	0	0
1	1	0		1	1	1	0	0	0
1	1	0		1	1	1	0	0	0
1	1	0		1	2	2	0	0	0
1	1	9		1	1	1		0	0
1	1	0		1	1	1	0	0	0
1	1	0		1	1	1	0	0	0
1	1	0		3	1	1	0	0	0
2	2	0		1	1	1	0		
1	1	9		1	1	1	0	0	0
1	1	0		1	1	1	0	0	0
1	1	9		1	1	1		0	0
1	1	0		1	1	1	0		
1	1	1	0	1	1	1	0	0	0
1	1	9		1	1	1		0	0
1	1	0		1	1	1	0	0	0
1	1	0		1	1	1	0	0	0
1	1	1	1	1	1	1	0	0	0
1	1	1	1	1	1	1	0	0	0
1	1	0		1	1	1	0	1	1
4	1	1	1	4	4	4	0	0	0
1	1	0		1	1	1	0	0	0
4	1	1	1	4	4	1	1	0	0
1	1	1	0	1	1	1	0	0	0

1	1	0		1	3	1	1	0	0
1	1	1	0	1	1	1	0	0	0
4	1	1	1	4	1	1	1	0	0
1	1	0		1	1	1	0	0	0
1	1	0		1	1	1	0	0	0
3	1	1	1	3	4	1	1	0	0
1	1	1	0	1	4	1	1	0	0
1	1	0		1	1	1	0	0	
4	1	1	1	1	1	1	0	0	0
1	1	0		1	1	1	0	0	0
1	1	0		2	1	1	1	0	2
1	1	0		2	1	1	1	0	2
4	4	1	1	1	1	1	0	0	0
4	4	1	1	1	1	1	0	0	0
1	1	1	0	1	1	1	0	0	0
1	1	1	1	4	3	1	1	0	0
4	1	1	1	1	1	1	0	0	0
2	1	1	1	4	1	1	1	0	2
1	1	0		1	1	1	0	0	0
2	2	1	0	4	3	3	1	0	0
4	2	1	1	1	1	1	0	0	0
1	1	1	1	4	3	1	1	0	0
1	1	1	0	1	1	1	0	0	0
1	1	1	0	1	4	1	1	0	0
1	1	1	0	1	1	1	0	0	0
4	4	1	1	1	1	1	0	0	0
1	1	0		1	1	1	0	0	0
1	1	0		1	1	1	0	0	0
4	4	0		4	1	1	1	0	0
4	4	0		4	1	1	1	0	0
1	1	1	0	3	4	1	1	0	0
1	1	0		1	4	1	1	0	0
1	1	0		1	1	1	0	0	1
4	4	1	1	1	1	1	0	0	0
4	4	0		3	1	1	1	0	0
1	1	1	0	3	4	1	1	2	2
4	4	1	0	4	1	1	0	0	0
1	1	0		1	4	1	1	0	0
3	3	1	1	4	4	4	1	0	0
1	1	0		1	1	1	0	0	0
1	1	0		1	1	1	0	0	0
1	1	0		1	1	1	0	0	0
2	2	1	0	1	1	1	0	0	0
1	1	0		1	1	1	0	1	1
1	1	1	0	1	1	1	0	0	0
2	1	0		1	1	1	0	0	0
1	1	1	0	1	1	1	0	0	0

1	1	0		1	1	1	0	0	0
1	1	0		1	1	1	0	0	0
2	2	1	0	1	1	1	0	0	0
1	1	0		1	1	1	0	1	1
1	1	0		3	1	1	0	0	0
1	1	0		1	1	1	0	0	0
1	1	0		1	1	1	0	0	0
1	1	0		1	1	1	0	0	0
1	1	0		2	1	1	0	0	0
1	1	0		1	1	1	0	0	0
1	1	0		1	1	1	0	0	0
1	1	0		1	1	1	0	0	0
1	1	0		1	1	1	0	0	0
3	1	1	1	1	1	1	0	1	1
3	1	1	1	1	1	1	0	0	0
3	1	1	1	1	1	1	0	0	0
2	2	1	1	1	1	1	0	0	0
1	1	0		1	1	1	0	0	0
1	1	0		1	1	1	0	0	0
1	1	0		1	1	1	0	0	0
1	1	0		1	1	1	0	0	0
1	1	0		1	1	1	0	0	0
2	2	0		1	1	1	0	0	0
3	3	0		1	1	1	0	0	0
1	1	0		1	1	1	0	0	0
1	1	0		1	1	1	0	0	0
3	1	0		1	1	1	0	0	0
1	1	0		1	1	1	0	0	0
1	1	0		1	1	1	0	0	0
1	1	0		1	1	1	0	0	0
1	1	0		1	1	1	0	0	0
1	1	0		1	1	1	0	0	0
1	1	0		1	1	1	0	0	0
1	1	0		1	1	1	0	0	0
4	1	1	0	4	4	1	1	0	0
1	1	0		1	1	1	0	0	0
1	1	1	0	1	1	1	0	0	0
1	1	0		3	3	1	1	0	0
1	1	0		1	1	1	0	0	0
1	1	0		1	1	1	0	0	0
1	1	0		1	1	1	0	0	0
1	1	0		1	1	1	0	0	0
1	1	0		3	1	1	1	0	0
1	1	0		1	1	1	0	0	0
1	1	0		1	1	1	0	0	0
1	1	0		1	1	1	0	0	0
1	1	0		1	1	1	0	0	0

3	1	0		1	1	1	0	0	0
1	1	0		1	1	1	0	2	2
1	1	0		3	1	1	0	0	0
1	1	0		1	1	1	0	0	0
4	4	0		4	3	4	1	0	2
2	3	0		1	3	4	1	2	1
1	1	0		1	1	1	0	0	0
1	1	1	0	1	1	1	0	0	0
1	1	0		4	4	1	1	0	0
1	1	1	1	1	1	1	0	1	0
1	1	1	1	4	4	4	1	0	0
1	1	0		3	1	1	0	2	2
1	1	0		3	1	1	1	0	0
1	1	0		1	1	1	0	0	0
4	4	1	1	4	1	4	1	0	0
1	1	0		1	1	1	0	0	0
1	1	0		1	1	1	0	0	0
4	4	1	1	1	1	1	0	0	0
1	1	0		1	1	1	0	0	0
1	1	0		1	1	1	0	0	0
1	1	0		3	3	1	1	0	0
1	1	0		1	1	1	0	0	0
1	1	0		1	1	1	0	0	0
1	1	0		1	1	1	0	0	0
1	1	0		1	1	1	0	0	0
1	1	0		1	1	1	0	0	0
2	1	1	1	1	3	1	1	0	0
2	1	1	1	3	1	2	1	1	0
1	4	1	1	1	1	1	0	0	0
1	4	1	1	1	1	1	0	0	0
3	3	1	1	1	1	1	0	0	0
3	3	1	1	1	1	1	0	0	0
1	1	0		1	1	1	0	0	0
1	1	0		1	1	1	0	0	0
1	1	0		1	1	1	0	0	0
1	1	0		1	1	1	0	0	0
3	3	0		3	3	3	1	1	1
1	1	0		1	1	1	0	0	0
1	1	0		1	1	1	0	0	0
1	1	0		3	3	1	1	0	0
1	1	0		1	1	1	0	0	0
1	1	0		3	3	1	1	0	0
1	1	0		1	1	1	0	0	0
1	1	0		1	1	1	0	0	0
1	1	0		1	1	1	0	0	0
1	1	0		1	1	1	0	0	0
1	1	0		1	1	1	0	0	0

4	1	0		4	1	1	0	0	0
3	3	1	1	3	1	1	0	0	0
4	2	1	1	4	4	4	1	0	0
3	1	1	0	2	1	1	0	0	
1	1	0		1	1	1	0	0	0
1	1	0		1	1	1	0	2	0
1	1	0		1	1	1	0	0	0
1	1	1	1	1	1	1	0	0	0
1	1	1	1	4	1	1	1	0	0
1	1	0		1	1	1	0	0	0
1	1	0		1	1	1	0	0	0
1	1	1	0	3	3	1	1	0	0
1	1	0		1	1	1	0	0	0
1	1	0		1	1	1	0	0	0
3	1	1	1	2	2	1	1	0	0
1	1	0		3	1	1	0	0	0
1	1	0		1	1	1	0	0	0
1	1	1	1	2	2	1	1	0	0
1	1	0		1	1	1	0	0	0
1	1	0		1	1	1	0	0	0
1	1	0		1	1	1	0	0	0
1	1	0		2	2	1	1	1	1
1	1	0		1	1	1	0	0	0
1	1	0		1	1	1	0	1	0
1	1	0		1	1	1	0	0	0
1	1	0		1	1	1	0	0	0
1	1	0		1	1	1	0	0	0
1	1	0		1	1	1	0	0	0
1	1	0		4	4	1	1	0	0
1	1	0		1	1	1	0	0	0
1	1	0		3	1	1	0	0	0
1	1	0		3	3	1	1	0	0
1	1	0		1	1	1	0	0	0
1	1	0		1	1	1	0	0	0
3	1	0		3	1	1	1	0	0
1	1	0		1	1	1	0	0	0
1	1	0		1	1	1	0	0	0
1	1	0		1	1	1	0	0	0
1	1	0		1	1	1	0	0	0
1	1	0		1	1	1	0	0	0
4	4	0		4	4	1	0	0	0
1	1	0		3	1	1	0	0	0
1	1	0		1	1	1	0	0	0
3	1	0		3	1	1	0	0	0
1	1	0		1	1	1	0	0	0
1	1	0		1	1	1	0	0	0
1	1	0		3	2	1	0	0	0

4	1	1	1	3	3	3	1	0	0
1	1	0		1	1	1	0	0	0
1	1	0		1	1	1	0	0	0
1	1	0		1	1	1	0	0	0
1	1	0		1	1	1	0	0	0
1	1	0		1	1	1	0	0	0
1	1	0		3	4	4	1	1	2
1	1	0		4	4	1	1	0	0
1	1	0		1	1	1	0	0	0
1	1	0		1	1	1	0	0	0
1	1	0		1	1	1	0	0	0
1	1	0		1	1	1	0	0	0
1	1	0		1	1	1	0	0	0
1	1	0		1	1	1	0	0	0
4	1	1	0	1	1	1	0	0	0
3	3	1	0	3	3	3	0	2	2
1	1	0		1	1	1	0		0
1	1	0		1	1	1	0	0	0
1	1	0		1	1	1	0	0	0
1	1	0		1	1	1	0	1	1
1	1	0		1	1	1	0	0	0
1	1	0		1	1	1	0	0	0
1	1	0		1	1	1	0	0	0
1	1	0		1	1	1	0	0	0
1	1	0		1	1	1	0	0	0
1	3	0		1	1	1	0	0	0
1	1	0		1	1	1	0	0	0
1	1	0		1	1	1	0	0	1
1	2	1	1	2	1	1	0	0	1
1	1	0		1	1	2	1	0	0
2	2	1	0	1	1	1	0	0	0
1	1	0		1	1	1	0	0	0
1	1	0		1	1	1	0	0	0
3	1	1	1	1	1	1	0	0	0
1	1	0		1	1	1	0	0	0
1	1	0		1	1	1	0	0	2
4	3	1	1	1	2	1	1	0	0
1	1	0		1	1	1	0	0	0
1	1	1	0	1	1	1	0	0	0
1	1	1	0	2	1	1	0	0	0
1	1	0		1	1	1	0	0	0
2	1	1	0	1	1	1	0	1	0
1	2	1	1	1	1	1	1	0	0
1	1	0		1	1	1	0	0	0
1	1	0		1	1	1	0	0	0
4	1	0		1	1	2	0	0	0
4	1	0		1	1	1	0	0	0

3	3	1	1	1	3	1	1	0	0
1	1	0		1	1	1	0	0	0
1	1	0		1	1	1	0	0	0
1	1	1	0	3	1	1	0	0	0
1	1	1	0	3	1	1	0	0	0
1	1	0		1	4	1	1	0	0
1	1	0		1	1	1	0	0	0
1	1	1	0	1	1	1	0	0	0
1	1	0		1	1	1	0	0	0
3	1	1	0	1	1	1	0	0	0
1	1	0		1	1	1	0	0	0
1	1	0		1	1	1	0	0	0
1	1	0		1	1	1	0	0	0
1	1	0		1	1	1	0	0	0
1	1	0		1	1	1	0	0	0
1	1	0		3	3	1	0	0	0
1	1	0		1	3	1	0	0	0
1	1	0		1	1	1	0	0	0
1	1	0		1	1	1	0	2	2
1	1	0		1	4	1	1	0	0
1	1	0		1	1	1	0	0	0
1	1	0		2	3	1	1	0	0
1	1	0		4	1	1	0	0	0
1	1	0		1	1	1	0	0	0
1	1	0		1	1	1	0	1	0
1	1	0		1	1	1	0	0	0
1	1	0		1	1	1	0	0	0
		9						0	0
1	1	0		1	1	1	0	0	0
1	1	0		1	1	1	0	0	0
1	1	0		1	1	1	0	0	0
1	1	0		1	1	1	0	0	0
1	1	0		1	1	1	0	0	0
1	1	0		1	1	1	0	0	0
2	1	1	1	1	1	1	0	0	0
1	1	0		1	1	1	0	0	0
1	1	0		3	3	3	1	1	1
1	1	0		1	1	1	0	0	0
1	1	0		1	1	1	0	0	0
1	1	0		1	1	1	0	0	0
1	1	0		1	1	1	0	2	2
1	1	0		1	1	1	0	0	0
1	1	0		1	1	1	0	0	0
1	1	1	0	1	1	1	0	0	0
1	1	0		4	4	1	1	0	0
1	1	0		1	2	1	0	0	0
1	1	0		1	1	1	0	0	0

1	1	0		1	1	1	0	0	1
1	1	0		1	1	1	0	0	0
1	1	1	0	1	1	1	0	0	0
1	1	0		1	1	1	0	0	0
1	1	0		1	1	1	0	0	0
1	1	1	0	1	1	1	0	0	0
1	1	1	0	1	1	1	0	0	0
1	1	0		3	1	1	0	0	0
1	1	0		1	1	1	0	0	0
1	1	0		1	1	1	0	0	0
1	1	1	0	1	1	1	0	0	0
1	1	0		2	1	1	0	0	0
2	1	1	0	1	1	1	0	0	0
4	1	1	1	1	1	1	0	0	0
1	1	0		4	2	1	1	0	0
1	1	0		1	1	1	0	0	0
1	1	0		4	1	1	0	2	2
1	1	0		1	1	1	0	0	
1	1	0		3	3	1	1	0	0
3	1	1	1	3	3	3	1	1	0
1	1	0		1	1	1	0	0	0
1	1	0		1	1	1	0	0	0
1	1	0		1	1	1	0	0	0
1	1	0		1	1	1	0	0	0
1	1	0		1	1	1	0	0	0
3	4	1	1	3	4	4	1	0	0
1	1	0		1	1	1	0	0	0
1	1	0		1	1	1	0	0	2
1	1	0		1	1	1	0	0	0
1	1	0		1	1	1	0	0	0
1	1	0		1	1	1	0	0	0
1	1	0		1	1	1	0	0	0
1	1	0		1	1	1	0	0	0
1	1	0		1	1	1	0	0	0
1	1	0		1	1	1	0	0	0
1	1	0		1	1	1	0	0	
1	1	0		1	1	1	0	0	0
1	1	0		1	1	1	0	0	0
1	1	0		1	1	1	0	1	1
1	1	0		1	1	1	0	0	0
1	1	0		1	1	1	0	0	0
1	1	1	1	1	2	1	1	1	2
1	2	1	1	2	2	1	0	0	0
1	1	0		1	1	1	0	0	0
1	1	0		1	1	1	0	0	2
1	1	0		1	1	1	0	0	0
1	1	0		1	3	1	1	2	2

4	4	1	1	1	1	1	0	0	0
1	1	0		1	1	1	0	0	0
1	1	0		1	1	1	0	0	0
1	1	0		1	1	1	0	0	0
1	1	0		1	1	1	0	0	0
		0					0	1	0
3	1	1	1	1	1	1	0	0	0
1	2	0		1	1	1	0	0	0
1	1	0		1	1	1	0	0	0
3	3	1	0	1	1	1	0	0	0
1	1	1	0	3	3	1	1	0	0
1	1	0		1	3	1	0	0	0
1	1	0		1	1	1	0	0	0
1	1	1	0	1	2	1	1	1	1
1	1	0		1	1	1	0	0	0
2	2	1	1	1	1	1	0	0	0
		9							
3	3	1	1	1	1	1	0	0	0
1	1	0		2	3	3	1	0	1
1	1	0		2	1	1	1	0	0
1	1	0		1	1	1	0	0	0
3	1	0		1	1	1	0	0	0
1	1	0		1	1	1	0	0	0
1	1	0		1	1	1	0	0	0
1	1	0		1	1	1	0	0	0
1	1	1	0	1	1	1	0	1	
4	2	1	1	1	1		0	2	2
1	1	0		2	1	1	0	0	0
1	1	0		1	1	1	0	0	0
1	1	1	1	3	1	1	1	0	2
1	1	0		1	1	1	0	0	0
1	1	0		1	3	1	0	0	0
1	1	0		1	1	1	0	0	0
1	1	0		1	1	1	0	0	0
1	1	0		1	1	1	0	0	0
1	1	0		1	1	1	0	0	0
1	1	0		1	1	1	0	0	0
1	1	0		1	1	1	0	1	1
4	4	1	1	4	4	4	1	0	0
1	1	0		1	1	1	0	0	0
1	1	0		1	1	1	0	0	0
1	1	1	1	4	3	1	1	0	0
1	2	1	1	3	3	1	1	2	2
1	1	0		1	1	1	0	0	0
1	1	9		1	1	1		0	0
	1	0		1	1	1	0	0	0
1	1	9		1	1	1		0	0

1	1	0		1	1	1	0	0	0
1	1	0		1	1	1	0	0	0
1	1	0		1	1	1	0	0	0
		9						0	0
1	1	0		1	1	1	0	0	0
1	1	0		1	1	1	0	0	0
1	2	1	1	1	1	1	0	0	0
1	1	0		1	1	1	0	0	0
1	1	0		1	1	1	0	0	0
2	2	1	0	1	1	1	0	0	0
1	1	0		4	1	1	1	0	0
1	1	1	1	1	1	1	1	0	0
1	1	0		1	1	1	0	0	0
3	3	0		4	4	4	0	0	0
1	2	9		1	1	1		0	0
1	1	9		1	1	1		0	0
1	1	0		1	1	1	0	0	0
1	1	0		1	1	1	0	0	0
1	1	0		1	1	1	0		
2	2	1	1	1	1	1	0	0	0
4	4	0		4	4	4	1	0	0
1	1	0		1	1	1	0	0	0
1	1	0		1	1	1	0		0
1	1	0		3	1	3	1	0	0
1	1	9		1	1	1		0	0
1	1	0		1	1	1	0	0	0
1	1	0		1	1	1	0	0	0
1	1	0		1	1	1	0	0	0
1	3	0		2	3	2	0	1	1
1	1	0		1	1	1	0	0	0
1	1	0		1	1	1	0	0	0
1	1	0		1	1	1	0	2	
1	1	0		1	1	1	0	0	0
1	1	0		1	1	1	0	0	0
1	1	0		1	1	1	0	0	0
1	1	0		1	1	1	0	1	
1	1	0		1	1	1		0	0
1	1	1	0	1	1	1	0	0	0
1	1	0		1	1	1	0	0	0
1	1	0		1	1	1		0	0
1	1	0		3	4	1	1	0	0
4	4	0		1	1	1	0	0	0
1	1	0		1	1	1	0	0	
4	1	1	0	1	3	1	1	0	0
1	1	0		1	1	1	0	0	2
1	1	0		1	1	1	0	0	2
4	1	1	1	1	1	1	0	0	0

4	1	1	1	1	1	1	0	0	0
1	1	0		1	1	1	0	0	0
1	1	0		1	1	1	0	0	0
1	1	0		1	1	1	0	0	0
1	1	0		1	1	1	0	0	0
1	1	0		1	4	1	1	0	0
1	1	0		1	1	1	0	0	
4	4	0		1	1	1	0	0	0
1	1	0		2	3	1	1	0	0
1	1	0		1	1	1	0	0	0
1	1	0		1	1	1	0	0	0
1	1	0		1	1	1	0	0	0
1	1	0		1	3	1	1	0	0
3	1	1	1	3	4	1	1	0	0
1	1	0		1	1	1	0	0	0
1	1	1	0	3	1	1	1	0	0
1	1	0		1	1	1	0	0	0
1	1	0		1					
1	1	0		1	1	1	0	0	0
4	1	1	0	1	1	1	0	0	2
1	1	0		1	1	1	0	0	0
1	1	1	0	1	3	1	1	0	0
1	1	0		1	1	1	0	0	0
1	1	0		1	1	1	0	0	0
1	1	0		1	1	1	0	0	0
1	1	0		1	1	1	0	0	0
1	1	0		1	1	1	0	0	0
3	1	0		1	1	1	0	0	0
1	1	0		1	1	1	0	0	0
1	1	0		1	1	1	0	0	0
1	1	0		1	1	1	0	0	0
1	1	1	0	3	1	1	0	0	0
1	1	0		1	1	1	0	0	0
1	1	0		1	1	1	0	0	0
1	1	0		1	1	1	0	0	0
1	1	0		1	1	1	0	0	0
1	1	0		1	1	1	0	0	0
1	1	1	0	1	1	1	0	0	0
1	1	1	1	1	1	1	0	0	0
1	1	0		1	1	1	0	0	0
1	1	0		1	1	1	0	0	0
1	1	0		1	1	1	0	0	0
1	1	0		1	1	1	0	0	0
1	1	0		1	1	1	0	0	0
1	1	0		1	1	1	0	0	0
1	1	1	0	1	1	1	0	0	0
1	1	0		1	1	1	0	0	0

1	1	0		1	1	1	0	0	0
1	1	0		1	1	1	0	0	0
1	1	0		1	1	1	0	0	0
1	1	0		1	1	1	0	0	0
1	1	0		1	1	1	0	0	0
1	1	0		1	1	1	0	0	0
4	4	1	0	4	4	4	1	0	0
1	1	0		1	1	1	0	0	0
1	1	0		3	1	1	0	0	0
4	4	1	1	1	1	1	0	1	0
1	1	0		1	1	1	0	0	0
3	1	0		4	4	1	0	0	0
1	1	0		1	1	1	0	0	0
1	1	1	0	2	1	1	1	0	0
1	1	0		1	1	1	0	0	0
1	1	0		1	1	1	0	0	0
1	1	0		1	1	1	0	0	0
1	1	0		1	1	1	0	0	0
1	1	0		1	3	1	1	0	0
1	1	0		1	3	1	1	0	0
1	1	1	1	1	4	1	1	0	0
1	1	0		1	1	1	0	0	0
1	1	0		1	1	1	0	0	0
1	1	0		1	1	1	0	0	0
4	3	1	0	1	1	1	1	0	0
1	1	0		4	4	1	1	2	2
1	1	0		4	1	1	0	0	0
1	1	0		1	1	1	0	2	2
1	1	1	0	1	1	1	0	0	0
1	1	0		1	1	1	0	0	0
1	1	0		1	1	1	0	0	0
1	1	0		1	1	1	0	0	0
1	1	0		1	1	1	0	0	0
2	1	1	0	1	1	1	0	0	0
1	1	1	0	1	1	1	0	0	0
1	1	0		1	1	1	0	0	0
1	1	0		1	1	1	0	0	0
1	1	0		2	1	1	1	0	0
1	1	0		3	2	1	0	0	0
1	1	0		1	1	1	0	0	0
3	1	1	1	2	3	1	1	1	0
2	1	1	1	1	1	1	1	0	0
1	1	0		1	1	1	0	0	0
1	1	0		1	1	1	0	0	0
3	4	0		1	1	1	0	0	0
4	1	1	1	1	1	1	0	0	0
1	1	0		1	1	1	0	0	0

1	1	0		1	1	1	0	0	0
4	4	1	1	4	4	4	1	0	0
1	1	0		1	1	1	0	0	2
4	1	1	1	4	3	1	1	0	0
4	1	1	1	1	1	1	0	0	0
1	1	0		1	1	1	0	0	0
1	1	0		1	1	1	0	0	0
1	1	0		2	1	1	0	0	0
1	1	0		1	1	1	0	0	0
1	1	0		1	1	1	0	0	0
1	1	0		1	1	1	0	0	1
2	2	0		1	1	1	0	0	0
1	1	0		3	1	1	0	0	0
1	1	0		1	1	1	0	0	0
1	1	0		1	1	4	1	0	0
1	1	0		1	1	1	0	0	0
1	1	0		1	1	1	0	0	0
1	1	0		1	1	2	1	0	0
		9						0	0
2	1	1	1	3	1	1	1	0	0
1	1	0		4	4	1	0	0	0
3	1	0		1	1	1	0	0	0
3	1	1	1	3	3	3	1	0	0
1	1	1	0	3	3	1	1	0	0
1	1	0		1	1	1	0	0	0
1	1	1	0	1	1	1	0	0	0
1	1	0		1	1	1	0	0	0
1	1	0		3	1	1	0	0	0
1	1	0		3	1	1	0	0	0
1	1	0		3	1	1	0	2	0
1	1	0		2	1	1	1	0	0
3	2	1	1	3	1	3	1	1	0
1	1	0		1	1	1	0	0	0
1	1	0		1	1	1	0	0	0
1	1	0		1	1	1	0	0	0
1	1	0		1	1	1	0	0	0
1	1	0		1	1	1	0	0	0
3	1	1	0	3	1	1	0	0	0
1	1	0		1	1	1	0	0	0
1	1	0		1	1	1	0	0	0
4	4	0		3	3	1	0	0	0
4	1	1	1	1	1	1	0	0	0
1	1	0		1	1	1	0	0	0
1	1	0		1	1	1	0	0	0
1	1	0		1	1	1	0	0	0
		9						0	0
1	1	0		1	1	1	0	0	0

3	1	1	0	3	2	3	1	0	0
1	1	0		1	1	1	0	0	0
1	1	1	0	1	1	1	0	0	0
1	1	1	0	2	1	1	1	0	0
1	1	0		1	1	1	0	0	0
1	1	0		2	1	1	0	0	0
1	1	0		1	1	1	0	0	0
4	1	1	0	1	1	3	1	0	0
1	1	0		1	1	1	0	0	0
3	1	0		1	1	1	0	0	0
1	1	0		1	1	1	0	0	0
1	1	0		1	1	1	0	0	0
1	1	0		1	1	1	0	0	0
1	1	0		1	1	1	0	0	0
1	1	0		1	1	1	0	0	0
1	1	0		1	1	1	0	0	0
1	1	0		1	1	1	0	0	0
1	1	0		1	1	1	0	0	
1	1	0		1	1	1	0	0	0
1	1	0		1	1	1	0	0	0
		9						0	0
1	1	0		1	1	1	0	0	0
1	1	0		1	1	1	0	0	0
1	1	0		1	1	1	0	0	0
1	1	0		1	1	1	0	0	0
1	1	0		1	1	1	0	0	0
1	1	1	0	1	1	1	1	0	0
1	1	0		1	1	1	0	0	0
1	1	0		1	1	1	1	0	0
1	1	0		1	1	1	0	0	0
1	1	0		3	1	1	0	0	0
1	1	0		1	1	1	0	0	0
3	4	1	1	1	1	1	0	0	0
2	4	0		4	1	1	0	0	0
1	1	0		1	1	1	0	2	1
1	1	0		1	1	1	0	0	0
1	1	0		1	1	1	0	0	0
2	2	1	1	1	2	3	1	2	0
1	1	0		1	1	1	0	0	0
1	1	0		1	1	1	0	0	0
1	1	0		1	1	1	0	0	0
		9						0	0
1	1	0		1	3	1	0	0	0
1	1	0		1	1	1	0	0	0
1	1	0		1	1	1	0	0	0
1	1	1	1	1	1	1	0	0	0
1	1	0		1	1	1	0	0	0

1	1	1	0	1	1	1	0	0	0
1	1	0		1	1	1	0	0	0
1	1	0		1	1	1	0	0	0
1	1	0		1	1	1	0	0	0
2	1	1	0	1	1	1	0	0	0
1	1	0		3	3	1	1	2	2
1	1	0		1	1	1	0	0	0
1	1	0		1	1	1	0	0	0
1	1	1	0	1	1	1	0	0	0
1	1	1	0	1	1	1	0	0	0
1	1	0		1	1	1	0	0	0
1	1	0		1	1	1	0	0	0
1	1	0		1	1	1	0	0	0
1	1	0		1	1	1	0	0	0
1	1	0		1	1	1	0	0	0
1	1	0		1	1	1	0	0	0
1	1	0		1	1	1	0	0	0
1	1	0		1	1	1	0	0	0
1	1	0		1	1	1	0	0	0
1	1	0		1	1	1	0	0	0
1	1	0		1	1	1	0	0	0
4	1	1	0	4	4	1	1	0	0
1	1	0		3	1	1	1	0	0
1	1	0		1	1	1	0	0	0
1	1	0		1	1	1	0	0	0
1	1	0		3	1	1	0	0	0
1	1	0		1	3	1	0	0	0
1	1	0		1	1	1	0	0	0
1	1	0		1	1	1	0	0	0
1	2	1	0	3	3	1	1	0	0
1	1	0		1	1	1	0	0	0
1	1	0		1	1		0	0	0
1	1	0		1	1		0	0	0
1	1	0		1	1	1	0	0	0
1	1	0		1	1	1	0	0	0
1	1	0		1	1	1	0	0	0
1	1	1	1	4	1	3	1	0	0
1	1	0		1	1	1	0	0	0
1	1	0		1	1	1	0	2	2
1	1	0		1	1	1	0	0	0
1	1	0		1	1	1	0	0	0
1	1	0		1	1	1	0	0	0
1	1	0		1	1	1	0	0	0
1	1	0		1	2	1	1	2	1
1	1	0		1	1	1	0	0	0
4	1	1	1	1	2	1	1	1	2
1	1	0		3	1	1	1	2	0

1	1	1	0	1	1	2	1	0	0
3	1	1	1	4	4	2	0	0	0
1	1	0		1	1	1	0	0	0
1	1	0		1	1	1	0	0	0
1	1	1	0	3	3	1	0	0	0
2	1	1	1	3	4	2	1	1	1
1	1	0		1	1	1	0	0	0
1	1	0		1	1	1	0	0	0
1	1	0		1	1	1	0	0	0
1	1	1	0	3	3	1	1	1	1
1	1	0		1	1	3	0	0	0
4	4	1	1	1	1	1	0	0	0
2	1	1	1	4	4	2	1	2	2
4	4	1	1	1	2	2	1	1	0
1	1	0		1	1	1	0	0	0
1	1	0		1	1	1	0	0	0
1	1	0		1	1	1	0	0	0
1	1	0		1	1	1	0	0	0
1	1	0		1	1	1	0	0	0
1	1	0		1	1	1	0	0	0
1	1	0		1	1	1	0	0	0
1	1	0		1	1	1	0	0	0
3	3	1	1	3	3	3	1	0	0
1	1	0		1	1	1	0	0	0
1	1	0		1	1	1	0	0	0
1	1	0		1	1	1	0	0	0
1	1	0		1	1	1	0	0	0
3	3	0		2	2	2	0	0	0
1	1	0		1	1	1	0	1	
1	1	0		1	1	1	0	0	0
4	4	1	1	3	1	1	0	0	0
4	1	0		4	4	1	0	2	2
1	1	0		1	1	1	0	0	0
1	1	0		1	1	1	0	2	
1	1	0		1	1	1	0	0	0
1	1	0		1	1	1	0	0	0
1	1	0		3	1	1	1	0	0
1	1	0		1	1	1	0	0	0
1	1	0		1	1	1	0	2	0
1	1	0		1	1	1	0	0	0
3	3	1	1	1	1	1	0		
1	1	0		1	1	1	0		
1	1	0		1	1	1	0	0	0
1	1	0		1	1	1	0	0	0
4	1	0		1	1	1	0	1	0
2	1	1	0	4	1	1	0	0	0
3	1	1	0	3	1	1	0	0	0

2	1	0		1	1	1	0	0	0
1	1	0		1	1	1	0	0	0
1	1	1	1	1	1	1	1	0	0
4	1	0		1	1	1	0	0	0
1	1	0		1	1	1	0	0	0
4	1	1	1	4	1	1	1	0	
1	1	0		2	1	1	0	0	0
1	1	0		2	1	1	0	0	0
1	1	0		1	3	1	1	0	0
1	1	1	0	1	1	1	0	0	0
1	1	0		1	1	1	0	0	0
1	1	0		1	4	1	1	0	0
3	1	1	0	4	4	4	1	0	0
1	1	0		1	1	1	0	0	0
1	1	0		1	1	1	0	0	0
4	4	0		1	1	1	0	0	0
4	4	0		1	1	1	0	0	0
1	1	0		1	1	1	0	0	0
3	1	0		1	1	1	0	0	0
1	1	0		1	1	1	0	0	0
1	1	0		3	1	1	0	0	0
1	1	0		1	1	1	0	0	0
1	1	0		1	1	1	0	0	0
1	1	0		1	1	1	0	0	0
1	1	0		1	1	1	0	0	0
1	1	0		1	1	1	0		0
2	1	1	0	1	1	1	0	0	0
1	1	0		3	1	1	0	0	0
1	1	0		3	1	1	0	0	0
1	1	1	1	3	1	1	1	0	0
1	1	0		1	1	1	0	0	0
1	1	0		1	1	1	0	0	0
1	1	0		3	1	1	0	0	0
1	1	0		1	1	1	0	0	0
1	1	0		1	1	1	0		
1	1	0		1	1	1	0	0	0
1	1	0		1	1	1	0	0	0
1	1	0		1	1	1	0	0	0
1	1	0		4	1	1	0	0	0
1	1	0		4	1	1	0	0	0
1	1	0		1	1	1	0	0	0
1	1	0		1	1	1	0	0	0
1	1	1	0	1	3	1	1	0	0
3	4	1	1	4	4	1	1	0	0
1	1	0		1	1	1	0	0	0
1	1	1	0	1	1	3	0	0	0
1	1	1	1	2	1	1	1	1	1

1	1	0		1	1	1	0	0	0
1	1	0		1	3	1	0	0	0
3	1	1	0	1	1	1	0	0	0
1	1	0		1	1	1	0	0	0
1	1	0		1	1	1	0	0	0
1	4	0		1	1	1	0	0	0
2	1	1	0	1	1	1	0	0	0
1	1	0		1	1	1	0	0	0
1	1	1	0	1	1	1	0	0	0
1	1	0		1	1	1	0	0	0
1	1	0		1	1	1	0	0	0
1	1	0		1	1	1	0	0	0
2	2	1	1	3	1	1	1	1	
1	1	9		1	4	1	1	0	0
1	1	0		1	1	1	0	0	0
1	1	0		1	1	1	0	2	2
1	1	0		1	1	1	0	0	0
1	1	0		1	1	1	0	0	0
1	1	0		1	1	1	0	0	1
1	1	0		4	4	2	0	0	0
1	1	1	0	1	1	1	0	0	0
4	1	1	0	1	1	1	0	0	0
1	1	0		1	1	1	0	0	0
1	1	0		1	1	1	0	0	0
1	1	0		1	1	1	0	0	0
1	1	0		1	1	1	0	0	0
1	1	0		1	1	1	0	0	0
1	1	0		1	1	1	0	0	0
1	1	0		1	1	1	0	0	0
2	1	1	0	1	1	1	0	0	0
1	1	0		1	1	1	0	0	0
1	1	0		1	1	1	0	0	0
1	1	0		3	1	1	0	0	0
1	1	0		3	3	1	1	0	0
1	1	0		1	1	1	0	0	0
1	1	0		1	1	1	0	0	0
1	1	0		1	1	1	0	0	0
1	1	0		1	1	1	0	0	0
1	1	0		1	1	1	0	0	0
1	1	0		4	1	1	0	0	0
3	1	1	0	4	1	1	1	0	0
3	1	1	0	4	3	2	1	0	0
1	1	0		1	1	1	0	0	0
1	1	0		1	1	1	0	0	0
1	1	0		1	1	1	0	0	0
1	1	0		1	1	1	0	0	0
1	1	0		1	1	1	0	0	0

1	1	1	0	1	1	1	0	0	0
1	1	0		1	1	1	0	0	0
1	1	0		1	1	3	1	0	0
1	1	0		1	1	1	0	0	0
1	1	0		1	1	1	0	0	0
1	1	0		1	1	1	0	0	0
1	1	0		3	2	1	0	0	0
1	1	0		1	1	1	0	0	0
1	1	0		1	1	1	0	0	0
1	1	0		1	1	1	0	0	0
1	1	0		1	1	1	0	0	0
1	1	0		1	1	1	0	0	0
1	1	0		1	1	1	0	0	0
1	1	0		1	1	1	0	0	0
1	1	0		1	1	1	0	0	0
1	1	0		1	1	1	0	0	0
		0						0	0
1	1	0		1	1	1	0	0	0
1	1	0		1	1	1	0	0	0
1	1	0		1	1	1	0	0	1
1	1	0		1	1	1	0	0	0
3	1	1	1	2	3	1	1	1	0
1	1	0		3	1	1	1	0	0
1	1	0		1	1	1	0	0	0
1	1	0		1	1	1	0	0	2
1	1	0		3	3	1	1	0	2
1	1	0		1	1	1	0	0	0
4	4	1	1	1	4	1	1	0	0
1	1	0		1	1	1	0	0	0
3	1	1	0	2	1	1	0	1	0
1	1	0		1	1	1	0	0	0
1	1	0		1	1	1	0	0	0
1	1	0		1	1	1	0	0	0
4	4	0		1	1	3	1	1	2
1	1	0		1	1	1	0	0	0
4	4	0		3	3	1	0	0	0
1	1	0		1	1	1	0	0	0
1	1	0		1	1	1	0	0	0
1	1	0		1	1	2	1	0	0
1	1	1	0	3	4	1	1	0	0
1	1	0		1	1	1	0	0	0
1	1	0		1	1	1	0	0	0
1	1	0		1	1	1	0	0	0
1	1	0		1	1	1	0	0	0
1	1	0		1	1	1	0	0	0
2	1	0		1	1	1	0	0	0
1	1	0		1	1	1	0	0	0

1	1	0		1	1	1	0	0	0
3	3	1	0	3	4	1	1	0	0
1	1	0		1	1	1	0	0	0
1	1	1	0	1	1	1	0	0	0
1	1	0		1	1	1	0	0	0
1	1	1	0	1	3	1	1	0	0
1	1	0		1	1	1	0	0	0
1	1	0		3	3	1	0	0	0
1	1	0		1	1	1	0	0	0
1	1	0		1	1	1	0	0	0
1	1	0		1	1	1	0	0	0
1	1	0		1	1	1	0	0	0
1	1	0		1	1	1	0	0	0
1	1	0		1	1	1	0	0	0
1	1	0		1	1	1	0	0	0
1	4	1	0	3	4	4	1	2	2
1	1	0		1	1	1	0	0	0
4	1	1	1	1	1	1	0	0	0
1	1	0		1	1	1	0	0	0
1	1	0		1	1	1	0	0	0
3	3	0		1	1	1	0	0	0
1	1	0		1	1	1	0	0	0
4	1	0		3	1	3	1	2	2
1	1	0		1	1	1	0	0	0
1	1	0		1	1	1	0	0	0
3	1	0		1	1	1	0		
1	1	0		1	1	1	0	0	0
1	1	0		3	3	2	0	1	1
4	4	1	0	1	1	1	0	0	0
1	1	0		1	1	1	0	0	0
1	2	0		1	1	1	0	0	0
1	1	0		1	1	1	0	0	0
1	1	0		1	1	1	0	0	0
1	1	0		1	1	1	0	0	
1	1	0		1	1	1	0	2	2
2	3	1	0	4	4	1	1	2	
1	1	0		1	1	1	0	0	0
1	1	0		3	3	3	1	0	0
1	1	0		3	3	3	1	0	0
1	1	0		1	1	1	0	0	0
1	1	1	0	2	3	1	1	2	2
2	3	0		1	2	2	0	0	0
1	1	1	0	1	1	1	0	0	0
1	1	0		2	1	1	0	0	0
1	1	0		1	1	1	0	0	0
1	1	0		1	1	1	0	0	0
1	1	0		1	1	1	0	0	0

1	1	0		1	1	1	0	0	0
1	1	0		1	1	1	0	0	0
1	1	0		1	1	1	0	0	0
1	1	0		1	1	1	0	0	0
1	1	0		1	4	1	0	0	0
1	2	1	0	1	1	1	0	0	2
1	1	0		1	1	1	0	0	0
1	1	1	0	2	1	1	1	0	0
1	1	0		4	1	1	0	2	
1	1	0		1	1	1	0	0	0
2	2	1	1	1	1	1	0	0	0
1	1	0		4	1	1	0	2	
3	3	0		3	3	3	1	1	0
1	1	0		4	1	1	1	0	0
1	1	0		4	1	1	0	0	0
1	1	0		1	1	1	0	0	0
1	1	0		1	1	1	0	0	0
1	1	0		1	2	1	0	0	0
3	3	1	1	3	2	2	0	0	0
3	3	1	1	3	2	2	0	0	0
1	1	0		1	1	1	0	0	0
1	1	0		1	1	1	0	0	0
3	1	0		1	1	1	0	0	0
1	1	0		1	1	1	0	0	0
1	1	0		1	1	1	0	0	0
1	3	1	0	3	1	1	1	0	0
4	2	1	0	4	1	1	1	0	0
4	4	1	0	1	1	1	0	0	0
1	1	1	1	1	1	1	0	0	0
1	1	0		1	1	1	0	0	0
4	4	1	0	1	1	1	0	0	0
1	1	0		1	3	1	1	0	0
1	1	0		1	1	1	0	0	2
1	1	0		1	4	1	1	0	0
3	3	0		1	3	1	0	0	0
1	1	0		1	1	1	0	0	0
2	1	0		1	1	1	0	0	0
1	1	0		1	1	1	0	0	0
1	1	0		1	1	1	0	0	0
4	4	0		1	1	1	0	0	0
1	1	0		1	1	1	0	2	0
1	1	0		3	1	1	0	0	0
1	1	0		1	1	1	0	0	0
1	1	0		1	1	1	0	0	0
1	1	0		1	1	1	0	0	0
1	1	0		1	1	1	0	0	0
1	1	0		1	1	1	0	0	0

1	1	0		1	1	1	0	0	0
1	1	0		1	1	1	0	0	0
1	1	0		1	1	1	0	0	0
1	1	0		4	1	1	0	0	0
4	1	1	0		1	1	0	0	0
1	1	0		1	1	1	0	0	0
1	1	0		1	1	1	0	0	0
1	1	0		1	1	1	0	0	0
1	1	0		1	1	1	0	0	0
1	1	0		1	1	1	0	0	0
1	1	0		1	1	1	0	0	0
1	1	0		3	1	1	0	0	0
1	1	0		3	1	1	0	0	0
1	1	0		1	1	1	0	0	0
1	1	0		1	3	1	0	0	0
1	1	0		1	1	1	0	0	0
4	1	1	1	4	4	3	1	0	0
4	1	0		4	4	1	0	0	0
1	1	1	0	3	3	1	1	0	0
1	4	1	0	1	1	1	0	0	0
1	1	0		3	3	1	0	0	0
		0		1	1	1	0	0	0
1	1	0		1	3	1	0	0	0
1	1	0		1	1	1	0	0	0
1	1	0		1	1	1	0	0	0
1	1	1	0	1	1	1	0	0	0
1	1	0		1	1	1	0	0	0
1	1	0		1	1	1	0	0	0
2	2	1	0	3	3	1	1	0	0
1	1	0		1	1	1	0	0	0
1	1	0		1	1	1	0	0	0
1	1	1	0	1	1	1	1	0	0
1	1	0		1	1	1	0	0	0
1	1	0		1	1	1	0	0	0
1	1	0		1	1	1	0	0	0
2	1	0		2	1	1	0	0	0
1	1	0		1	3	3	1	1	1
1	1	0		1	1	1	0	0	0
1	2	0		2	1	1	0	0	0
1	1	0		2	1	1	0	0	0
1	1	0		1	1	1	0	0	1
1	1	0		1	1	1	0	0	0
3	3	1	0	4	3	1	1	0	0
4	1	1	0	3	1	1	0	0	0
1	1	0		1	1	1	0	0	0
1	1	0		2	1	1	1	0	0
3	1	1	0	1	1	1	0	0	0

1	1	0		1	1	2	1	0	
2	1	0		4	4	1	0	0	0
1	1	1	1	1	1	1	0	0	0
1	1	0		4	4	1	1	0	0
2	1	0		3	4	4	1	0	0
4	4	1	0	1	1	1	1	0	0
1	1	1	0	1	1	1	0	0	0
1	1	0		1	1	1	0	0	0
4	4	1	0	1	1	1	1	0	0
		9		1	1	1	0	0	0
1	1	0		1	1	1	0	0	0
1	1	0		1	1	1	0	0	0
1	1	0		1	1	1	0	0	0
1	1	0		1	1	1	0	0	0
4	4	1	0	1	1	1	0	0	0
1	1	0		1	1	1	0	0	0
3	3	1	0	1	1	1	0	2	0
1	1	0		1	1	1	0	0	0
1	1	0		1	1	1	0	0	0
1	1	1	0					0	1
3	1	0		3	3	1	0	0	0
1	1	0		1	1	1	0	0	0
1	1	0		1	1	1	0	0	0
1	1	0		1	1	1	0	0	0
1	4	1	1	1	3	1	1	0	1
1	1	0		1	1	1	0	0	0
4	2	1	0	1	1	1	0	1	1
3	1	0		1	1	1	0	0	0
1	1	0		4	4	1	1	0	0
1	1	0		1	1	1	0	0	1
1	1	0		1	1	1	0	0	0
4	1	0		4	4	4	1	2	0
1	1	0		1	1	1	0	0	0
1	1	0		1	2	4	1	0	0
1	1	0		1	1	1	0	0	0
1	1	0		1	1	1	0	0	1
1	1	0		1	1	1	0	0	0
1	1	0		1	1	1	0	0	0
2	1	0		1	1	1	0	0	0
1	1	0		1	1	1	0	0	0
1	1	0		1	1	1	0	0	0
1	1	0		1	1	1	0	0	0
1	1	0		3	3	1	1	0	0
4	1	0		1	1	1	0	0	0
1	1	0		1	1	1	0	0	0
1	1	0		1	1	1	0	0	0
1	1	0		1	1	1	0	0	0

1	1	0		1	1	1	0	0	0
		8						0	0
1	1	0		1	1	1	0	0	0
1	1	0		1	1	1	0	1	1
1	1	0		1	1	1	0	0	0
1	1	0		2	2	2	1	1	0
1	1	0		1	1	1	0	0	0
1	1	0		1	1	1	0	0	0
1	3	0		1	1	1	0	1	0
1	1	0		1	1	1	0	0	0
1	1	0		1	1	1	0	0	0
1	1	0		1	1	1	0	0	0
1	1	0		1	1	1	0	0	0
1	1	0		1	1	1	0	0	0
1	1	0		3	3	1	0	2	2
1	1	0		1	1	1	0	0	0
1	1	0		1	1	1	0	0	0
		9						0	0
2	1	0		1	1	1	0	0	0
1	1	0		1	1	1	0	0	0
1	1	0		2	1	1	0	0	0
1	1	0		1	1	1	0	0	0
1	1	0		1	3	1	0	0	0
1	1	0		1	1	1	0	0	0
1	1	0		1	1	1	0	0	0
1	1	0		1	1	1	0	0	0
1	1	1	0	1	1	1	0	0	0
1	1	0		4	1	1	0	0	0
1	1	0		1	1	1	0	0	0
1	1	0		1	1	1	0	0	0
1	2	0		1	1	1	0	0	0
4	4	1	1	1	1	1	0	0	0
1	1	0		1	1	1	0	0	0
1	1	0		1	1	1	0	0	0
1	1	0		1	1	1	0	0	0
1	1	0		1	1	1	0	0	0
1	1	0		1	1	1	0	0	0
1	1	0		1	1	1	0	0	0
3	1	1	0	3	1	1	1	0	0
		0	0	3	3	4	0	0	0
4	4	1	1	3	3	2	1	0	0
1	1	0		1	1	1	0	0	0
1	1	1	0	1	1	1	0	0	0
1	1	0		1	1	1	0	0	0
1	1	0		1	1	1	0		
4	4	0		1	1	1	0	0	0
1	1	0		1	1	1	0	0	0

4	4	0		1	1	1		0	0
1	1	0		1	1	1	0	0	0
1	1	0		1	1	1	0	0	0
1	1	0		1	1	1	0	0	0
1	1	1	0	1	1	1	0	0	0
1	1	0		1	1	1	0	0	0
1	1	0		1	1	1	0	0	0
		9							
3	3	1	0	4	1	1	0	2	0
1	1	0		1	1	1	0	2	2
4	1	0		1	1	1	0	0	0
2	1	0		1	1	1	0	2	2
1	1	0		1	1	1	0	0	0
1	1	0		1	1	1	0	0	0
1	1	0		3	4	1	1	0	0
1	1	0		1	1	1	0	0	
1	1	0		1	1	1	0	0	0
1	1	0		1	1	1	0	0	0
1	1	0		2	1	1	0	0	0
4	4	1	1	3	3	1	1	0	0
1	1	0		4	1	1	1	0	0
1	1	0		1					
1	2	0		1	1	1	0	0	0
1	1	0		1	1	1	0	0	0
1	1	0		1	1	1	0	0	0
3	1	0		1	1	1	0	0	2
1	1	0		1	1	1	0	0	0
1	1	0		3	1	1	0	0	0
1	1	0		1	1	1	0	0	0
4	4	1	0	1	1	1	0	0	0
1	1	0		1	1	1	0	0	0
1	4	0		4	1	1	0	0	0
1	1	0		3	1	1	0	0	0
1	1	0		1	1	1	0	0	0
1	1	0		1	1	1	0	0	0
1	1	0		1	1	1	0	0	0
4	4	0		1	1	1	0	0	0
1	1	0		1	1	1	0	0	0
1	1	0		1	1	1	0	0	0
1	4	1	1	1	1	1	0	0	0
4	1	0		4	1	1	1	2	0
2	1	0		4	3	1	1	0	0
3	1	1	0	1	1	1	0	0	0
1	1	0		1	1	1	0	0	0
1	1	0		1	1	1	0	0	0
1	1	0		1	1	1	0	0	0
3	1	1	0	1	1	1	0	0	0

1	1	1	0	1	1	1	0	1	
1	1	0		1	1	1	0	0	0
1	1	0		1	1	1	0	0	0
1	1	0		1	1	1	0	0	0
1	1	0		4	3	2	1	0	1
1	1	0		1	1	1	0	0	0
1	1	0		1	1	1	0	0	0
1	1	1	1	4	4	1	1	0	0
1	1	0		1	1	1	0	1	0
1	1	0		1	3	1	0	0	0
4	1	1	0	4	1	1	1	0	0
3	1	0		1	1	1	0	0	0
3	1	0		1	1	1	0	0	0
1	1	0		1	1	1	0	0	0
1	1	0		4	4	1	0	0	0
1	1	0		4	4	1	0	0	0
1	1	0		4	4	4	0	0	0
1	1	0		4	3	1	0	0	0
1	1	0		1	1	1	0	0	0
1	1	0		1	1	1	0	0	0
1	1	0		1	1	1	0	0	0
1	1	0		1	1	1	0	0	0
1	1	0		1	1	1	0	0	0
1	1	0		3	1	1	0	0	0
1	1	0		1	1	1	0	0	0
3	1	1	0	1	1	1	0	0	0
1	1	0		1	1	1	0	0	0
1	1	0		1	1	1	0	0	0
1	1	0		3	2	1	1	0	0
1	1	0		2	2	1	0	0	0
2	2	1	0	1	1	1	0	0	0
1	1	0		2	2	1	0	0	0
3	3	0		1	1	1	1	0	2
1	1	0		1	1	1	0	0	0
4	4	1	1	1	1	1	0	0	0
1	1	0		1	1	1	0	0	0
1	1	0		1	1	1	0	0	0
1	1	0		1	1	1	0	0	0
1	1	0		1	1	1	0	0	0
1	1	0		1	1	1	0	0	0
1	1	0		1	1	1	0	0	0
1	1	0		1	1	1	0	0	0
1	1	0		1	1	1	0	0	0
2	1	0		1	1	1	0	0	0
1	1	0		1	1	1	0	0	0
1	1	0		1	1	1	0	0	0
1	1	0		1	1	1	0	0	0

4	4	1	1	1	3	3	1	0	2
1	1	1	0	1	1	1	0	0	0
4	4	1	0	1	1	1	0	2	2
1	1	0		1	1	1	0	0	0
1	1	0		1	1	1	0	0	0
2	1	1	0	1	1	1	0	0	0
3	1	1	1	4	4	4	1	0	0
1	1	0		1	1	1	0	0	0
1	1	0		3	3	1	0	0	0
1	1	0		1	1	1	0	0	0
1	1	1	0	1	1	1	0	0	0
1	1	0		1	1	1	0	0	0
		9						0	0
1	1	0		1	1	1	0	0	0
1	1	0		1	1	4	1	0	0
3	3	1	0	1	1	1	0	0	0
1	1	0		1	1	1	0	0	0
1	1	0		1	1	1	0	0	0
1	1	0		1	1	1	0	0	0
1	1	0		1	1	1	0	0	0
1	1	0		1	1	1	0	0	0
1	1	0		1	1	1	0	0	0
1	1	0		1	1	1	0	0	0
1	1	0		1	1	1	0	0	0
1	1	0		1	1	1	0	0	0
1	1	0		1	1	1	1	0	0
1	1	0		1	1	1	0	0	0
1	1	0		1	1	1	0	0	0
1	1	1	1	1	1	1	0	0	2
1	1	0		1	1	1	0	0	0
1	1	0		1	1	1	0	0	0
1	1	0		1	1	1	0	0	0
3	1	1	0	1	1	1	0	0	0
1	1	0		3	3	1	0	0	0
1	1	0		3	3	1	0	0	0
1	1	0		1	2	1	0	0	0
1	1	0		1	2	1	1	0	0
1	1	0		1	1	1	0	0	0
1	1	0		3	1	1	1	0	0
1	1	0		1	1	1	0	0	0
1	1	0		1	3	1	0		0
1	1	0		1	1	1	0	0	0
1	1	0		3	1	1	0	0	0
1	1	0		1	1	1	0	0	0
1	2	0		1	1	1	0		
1	1	0		1	1	1	0	0	0
4	4	1	0	1	1	1	0	0	0

4	4	1	0	1	1	1	0	0	0
1	1	0		1	1	1	0	0	0
1	1	0		3	1	3	0	0	0
1	1	0		1	1	1	0	0	0
1	1	0		1	1	1	0	0	0
		8				1	0	0	0
		9						0	0
		9							
1	1	0		1	1	1	0	0	0
1	1	0		1	1	1	0	0	0
1	1	0		1	2	1	0	0	0
1	1	0		1	1	1	0	0	0
1	1	1	0	1	1	1	0	0	0
1	1	0		1	1	1	0	0	0
1	1	0					0	0	0
1	1	1	0	1	1	1	0	0	0
1	1	0		1	1	1	0	0	0
1	1	0		1	1	1	0	0	0
4	4	1	0	1	1	1	0	2	2
2	1	1	0	3	1	1	0	1	1
1	1	0		1	1	1	0	0	0
1	1	0		1	1	1	0	0	0
1	1	0		1	1	1	0	0	0
1	1	0		1	1	1	0	0	0
1	1	0		1	1	1	0	0	0
1	1	0		1	1	1	0		
1	1	0		1	1	1	0	0	0
1	1	0		1	1	1	0	0	0
1	1	0		1	3	1	0	0	0
		0					0	0	0
1	1	0		1	1	1	0	0	0
1	1	0		1	1	1	0	0	0
1	1	0		1	1	1	0	0	0
		9					0		
1	1	0		1	1	1	0	0	0
1	1	0		1	1	1	0	0	0
1	1	0		1	1	1	0	0	0
1	1	9		1	1	1		0	0
1	1	0		1	1	1	0	0	0
2	1	0		1	2	1	0	0	0
1	1	0		1	1	1	0	0	0
1	1	0		1	1	1	0	0	0
1	1	0		1	1	1	0	0	0
3	3	0		1	1	1	1	0	0
1	1	0					0	0	0
1	1	0		1	1	1	0	0	0
1	1	0		1	1	1	0	0	0

1	1	0		1	1	1	0	0	0
1	1	9		1	1	1		0	0
1	1	0		1	1	1	0	0	0
1	1	0		1	1	1	0	0	0
1	1	0		1	1	1	0	0	0
1	1	9		1	1	1		0	0
2	2	1	1	1	1	1		0	0
2	2	0		2	2	2	0	0	0
		0						0	0
3	3	0		3	3	3	0	2	1
1	1	1	1	1	1	1	0	0	0
1	1	0		1	1	1	0	0	0
1	1	0		1	1	1	0	0	0
1	1	0		1	1	1	0	0	0
1	2	1	0	3	3	1	1	0	0
1	1	0		1	1	1	0	0	0
4	4	1	0	1	4	1	1	0	0
4	1	1	0	1	1	1	0	0	0
1	1	0		1	1	1	0	0	0
2	1	1	0	1	1	1	0	0	0
1	1	0		4	4	1	1	0	0
1	1	0		1	1	1	0	0	0
3	3	0		1	1	1	0	0	0
3	3	0		1	1	1	0	0	0
1	1	0		2	1	1	0	0	0
4	4	0		4	4	4	0	0	0
1	1	0		1	1	1	0	0	0
1	3	1	0	1	4	1	1	0	0
1	1	0		1	3	1	1	0	0
1	1	0		1	1	1	0	0	0
1	1	0		1	4	1	1	0	0
1	1	0		1	1	1	0	0	0
1	1	0		1	1	1	0	0	0
1	1	0		1	1	1	0	0	0
1	1	0		1	2	1	1	0	0
4	1	0		4	4	1	0	0	0
4	1	0		4	4	1	0	0	0
1	1	0		1	1	1	0	0	0
1	1	0		1	1	1	0	0	0
1	1	0		1	1	1	0	0	0
1	1	0		1	1	1	0	0	0
1	1	1	0	1	1	1	0	0	0
4	4	1	1	1	1	1	0	0	0
1	1	0		3	1	1	0	0	0
1	1	0		3	1	1	0	0	0
4	4	1	0	1	1	1	0	0	0
4	4	1	0	1	1	1	0	0	0

1	1	0		1	1	1	0	0	
4	1	0		1	1	1	0	0	0
1	1	0		1	1	1	0	0	0
2	1	0		3	3	1	0	0	0
2	1	1	0	1	1	2	1		
1	1	0		1	1	1	0	0	0
1	1	0		1	1	1	0	0	0
1	1	0		1	1	1	0	0	0
1	1	0		1	1	1	0	0	0
1	1	0		1	1	1	0	0	0
1	1	0		1	1	1	0	0	0
1	1	0		1	1	1	0	0	0
1	1	0		1	1	1	0	0	0
1	1	1	0	3	1	1	0	0	0
1	1	0		1	1	1	0	0	0
4	4	1	0	1	1	1	0	0	0
1	1	0		1	1	1	0	0	0
1	1	0		1	1	1	0	0	0
1	1	0		1	1	1	0	0	0
1	1	0		1	1	1	0	0	0
1	1	0		1	1	1	0	0	0
1	1	0		3	1	1	0	0	0
1	1	0		1	1	1	0	0	0
4	4	0		4	1	1	0	2	0
1	1	0		1	1	1	0	0	0
1	1	0		1	1	1	0	0	0
1	1	0		1	1	1	0	0	0
1	1	0		1	1	1	0	0	0
1	1	0		1	1	1	0	0	0
1	1	0		2	1	1	0	0	0
1	1	0		1	1	1	0	0	0
1	1	0		1	1	1	0	0	0
1	1	0		1	1	1	0	0	0
		9						0	0
1	1	0		1	1	1	0	0	0
1	1	0		1	1	1	0	0	0
1	1	0		1	2	1	0	0	0
1	1	0		1	3	1	0	0	0
1	1	0		1	3	1	0	0	0
1	1	0		3	3	1	0	0	0
1	1	1	0	4	3	1	1	0	0
3	1	1	0	1	1	1	0	0	0
3	1	1	0	3	3	1	1	0	0
1	1	0		1	1	1	0	0	0
1	1	0		1	1	1	0	0	0
4	1	1	0	1	4	1	1	0	2
1	1	0		1	1	1	0	0	0

1	1	0		1	1	1	0	0	0
1	1	1	0	1	1	1	0	0	0
1	1	0		1	1	1	0	0	0
1	1	0		1	1	1	0	0	1
1	1	0		1	1	1	0	0	0
1	1	0		1	1	1	0	0	0
1	1	0		1	1	1	0	0	0
1	1	0		1	1	1	0	0	0
1	1	0		1	1	1	0	0	0
1	1	0		3	1	1	0	0	0
1	1	1	0	3	1	1	1	0	0
3	1	0		1	1	1	0	0	0
1	1	1	0	1	1	1	0	0	0
1	1	0		1	1	1	0	0	0
1	1	0		1	3	1	0	0	0
1	1	0		1	3	1	0	0	0
1	1	0		1	1	1	0	0	0
1	1	0		4	4	4	0	0	0
1	1	0		4	4	4	0	0	0
1	1	0		1	1	1	0	0	0
1	1	1	0	1	1	1	0	0	0
1	1	0		3	1	1	0	0	0
1	2	1	1	1	1	1	0	0	0
1	1	0		1	1	1	0	0	0
1	1	0		1	1	1	0	0	0
1	1	0		1	1	1	0	0	0
1	1	0		1	1	1	0	0	0
1	1	0		1	1	1	0	0	0
1	1	0		1	1	1	0	0	0
1	1	0		1	1	1	0	0	0
1	1	0		1	1	1	0	0	0
1	1	0		1	1	1	0	0	0
1	1	0		1	1	1	0	0	0
1	1	0		1	1	1	0	0	0
2	1	0		1	1	1	0	0	0
3	1	0		3	3	1	0	0	0
1	1	0		1	1	1	0	0	0
1	1	0		2	2	1	0	0	0
1	1	0		1	1	1	0	0	0
		9				1	0	0	0
1	1	0		1	1	1	0	0	0
1	1	0		1	1	2	0	0	0
1	1	0		1	1	1	0	0	0
1	1	0		1	1	1	0	0	0
1	1	0		1	1	1	0	0	0
3	1	1	1	1	1	1	0	0	0
3	1	0		1	1	1	1	0	0

1	1	0		1	1	1	0	0	0
3	1	0		2	3	3	1	0	0
2	1	1	0	1	1	1	0	0	0
1	1	0		1	1	1	0	0	0
1	1	0		4	2	1	0	2	2
1	1	0		1	1	1	0	0	0
3	1	1	0	1	1	1	0	0	0
4	1	1	0	4	4	1	1	0	0
1	1	0		1	3	3	1	0	0
4	4	1	0	1	1	1	1	0	0
1	1	0		1	1	1	0	0	0
1	1	0		1	1	1	0	0	0
1	1	0		1	1	1	0	0	0
1	1	0		1	1	1	0	0	0
4	4	1	0	1	1	1	0	0	0
1	4	0		1	1	1	0	0	0
4	1	0		1	1	1	0	1	1
1	1	0		1	1	1	0	0	0
		9						0	0
1	1	0		1	1	1	0	0	0
1	1	0		1	1	1	0	0	0
4	4	0		1	4	1	0	0	0
1	1	0		1	1	1	0	0	0
1	1	0		1	1	1	0	0	0
		9							
1	1	0		1	1	1	0	0	0
1	1	0		1	1	1	0	0	0
1	1	0		1	1	1	0	0	0
4	1	0		1	1	1	0	0	0
1	1	0		1	1	1	0	0	0
4	4	1	0	4	4	4	0	1	0
1	1	0		1	1	1	0	0	0
		0					0	0	0
1	1	0		1	1	1	0	0	0
1	1	0		1	1	1	0	0	0
1	1	1	0	1	1	1	0	0	0
4	4	1	1	1	4	1	1	0	2
		9						0	0
1	1	0		1	1	1	0	0	0
1	2	0		1	1	1	0	0	0
1	1	0		1	1	1	0	0	0
2	1	0		1	1	1	0		
1	1	0		1	1	1	0	0	0
3	1	1	0	4	4	1	1	0	0
4	1	1	0	1	1	1	0	0	0
1	1	0		1	1	1	0	0	0
		9						0	2

1	1	0		1	1	1	0	0	0
1	1	0		1	2	1	1	1	1
4	3	1	0	3	4	1	0	0	2
1	1	0			1	1	0	0	0
1	1	0		1	3	2	0	0	2
1	1	0		1	1	1	0	0	0
1	1	0		1	1	1	0	0	0
1	1	0		1	1	1	0	0	0
1	1	0		1	1	1	0		
1	1	0		1	1	1	0	0	0
1	1	0		1	1	1	0	0	0
4	4	0		4	4	4	0	0	0
1	1	0		1	1	1	0	0	0
1	1	0		1	1	1	0	0	0
4	4	0		4	4	4	0	2	2
3	1	1	0	1	1	1	0	1	2
4	2	1	0	4	4	3	1	0	0
1	1	1	0	1	1	1	1	0	0
1	4	0		1	4	4	0	2	2
1	1	0		1	1	1	0	0	0
1	1	0		1	1	1	0	0	0
4	4	1	0	4	1	1	0	0	0
1	1	0		4	4	4	0	2	2
1	1	0		3	3	2	0	0	0
1	3	0		1	1	1	0	0	0
1	1	0		1	1	1	0	0	0
1	1	0		1	1	1	0	0	0
4	1	0		1	1	1	0	0	0
1	1	0		1	1	1	0	0	0
1	1	0		1	2	1	0	0	0
1	1	0		1	1	1	0	0	0
1	1	0		1	1	1	0	0	0
1	1	0		4	1	1	0	0	0
1	1	0		1	1	1	0	0	0
1	1	0		1	1	1	0	0	0
1	1	0		1	1	1	0	0	0
3	1	1	0	1	1	1	0	0	0
1	1	0		1	1	1	0	0	0
1	1	0		1	1	1	0	0	0
1	1	0		1	1	1	0	0	0
		9						0	0
1	1	0		1	1	1	0	0	0
1	1	0		1	1	1	0	0	0
1	1	0		1	1	1	0	0	0
1	1	0		1	1	1	0	0	0
1	1	0		1	1	1	0	0	0
1	1	0		1	1	1	0	0	0

1	1	0		1	1	1	0	0	0
1	1	0		1	1	1	0	0	0
1	1	0		1	1	1	0	0	0
1	1	0		1	1	1	0	0	0
1	1	0		1	1	1	0	0	0
1	1	0		1	1	1	0	0	0
1	1	0		1	1	1	0	0	0
4	1	0		1	1	1	0	0	0
1	1	0		1	1	1	0	1	
1	1	0		1	1	1	0	0	0
1	1	0		1	1	1	0	0	0
1	1	1	0	1	1	1	0	0	0
1	1	0		1	1	1	0	0	0
1	1	0		1	1	1	0	0	0
1	1	0		1	3	1	0	0	0
1	1	0		1	1	1	0	0	0
1	1	0		1	1	1	0	0	0
1	1	0		1	1	1	0	0	0
1	1	0		1	1	1	0	0	0
1	1	0		1	1	1	0	0	0
1	1	0		1	1	1	0	0	0
1	1	0		1	1	1	0	0	0
		9						0	0
1	1	0		3	3	1	0	0	0
1	1	0		1	1	1	0	0	0
1	1	0		1	1	1	0	0	0
1	1	0		1	1	1	0	0	0
1	1	1	0	4	1	1	0	0	0
1	1	0		1	1	1	0	0	0
1	1	0		1	1	1	0	0	0
1	1	0		3	1	1	0	0	0
1	1	0		1	1	1	0	0	0
1	1	0		1	1	1	0	0	0
1	1	0		1	1	1	0	0	0
1	1	0		1	1	1	0	2	0
1	1	0		4	1	1	0	0	2
1	1	0		1	1	1	0	0	0
4	1	1	1	1	1	1	0	0	0
1	1	0		1	1	1	0	0	0
1	1	0		1	1	1	0	0	0

prison	war	injury	death	murder	unnatdth	murdstr	torture	carjack	kidnap
0	0	0	0	0	0	0	0	0	0
0	0	0	0	0	0	0	0	0	0
0	0	0	0	0	0	0	0	0	0
0	0	0	0	0	0	0	0	0	0
0	0	0	0	0	0	0	0	0	0
0	0	0	0	0	0	0	0	0	0
0	0	0	0	0	0	0	0	0	0
0	0	0	0	0	0	0	0	0	0
0	0	0	0	0	0	0	0	0	0
0	0	0	0	0	0	0	0	0	0
0	0	0	0	0	1	0	0	0	0
1	0	1	0	0	1	0	0	0	0
0	0	0	0	0	0	0	0	0	0
0	0	0	0	0	0	0	0	0	0
0	0	0	0	0	0	0	0	0	0
0	0	0	0	0	0	0	0	0	0
0	0	0	0	0	0	0	0	0	0
0	0	1	1	0	1	0	0	0	0
0	0	0	1	0	1	0	0	0	0
0	0	0	0	0	0	0	0	0	0
0	0	0	0	0	0	0	0	0	0
0	0	1	0	0	1	0	0	0	0
0	0	0	0	0	1	1	0	0	0
0	0	0	0	0	0	0	0	0	0
0	0	1	0	0	1	0	0	0	0
0	0	0	0	0	0	0	0	0	0
0	0	0	0	0	0	0	0	0	0
0	1	0	0	0	0	0	0	0	0
0	0	0	0	1	1	0	0	1	0
0	0	1	1	0	1	0	0	0	0
0	0	0	0	0	0	0	0	0	0
0	0	0	0	0	0	0	0	0	0
0	0	0	0	0	1	0	0	0	0
0	0	1	1	1	1	0	0	1	0
0	0	0	0	0	0	0	0	0	0
0	0	0	0	0	1	0	0	0	0
0		0	0	0	0	0	1	0	0
0	0	1	0	0	0	1	0	0	0
1	0	0	0	0	0	0	0	0	0
0	0	0	0	0	1	0	0	0	0
0	0	0	0	0	0	0	0	0	0
0	0	0	0	0	1	0	0	0	0
0	0	0	0	0	0	1	0	0	0
0	0	0	0	0	0	0	0	0	0
0	0	1	0	0	0	0	0	0	0
0	0	0	0	0	1	0	0	0	0

0	0	1	0	1	1	0	0	0	0
0	0	0	0	1	1	1	0	0	0
0	0	0	0	0	0	0	0	0	0
0	0	0	0	0	0	0	0	0	0
0	0	0	0	0	0	0	0	0	0
0	0	0	0	0	0	0	0	0	0
0	0	0	0	0	0	0	0	0	0
0	0	0	0	0	0	0	0	0	0
0	0	0	0	1	0	0	1	0	0
0	0	1	1	0	0	0	0	0	0
0	0	0	0	0	1	0	0	0	0
0	0	0	0	0	0	0	0	0	0
0	0	0	0	0	0	0	0	0	0
0	0	1	0	0	0	0	0	0	0
0	0	0	0	0	0	0	0	0	0
0	0	1	1	0	1	0	0	0	0
0	0	0	0	0	0	0	0	0	0
0	0	0	0	0	0	0	0	0	0
0	0	0	0	0	0	0	0	0	0
0	0	1	0	0	0	0	0	0	0
0	0	0	0	0	1	0	0	0	0
0	0	0	0	0	0	0	0	0	0
0	0	0	0	0	0	0	0	0	0
0	0	0	0	0	0	0	0	0	0
0	0	0	0	0	0	0	0	0	0
0	0	0	0	0	0	0	0	0	0
0	0	0	0	0	0	0	0	0	0
0	0	0	0	0	0	0	0	1	0
0	0	0	0	0	0	0	0	0	0
0	0	0	0	0	0	0	0	0	0
0	0	0	0	0	0	0	0	0	0
0	0	0	0	0	0	0	0	0	0
0	0	0	0	0	0	0	0	0	0
0	0	0	0	0	0	0	0	0	0
0	0	0	0	0	0	0	0	0	0
0	0	0	0	0	0	0	0	0	0
0	0	0	0	0	0	0	0	0	0
0	0	0	0	0	0	0	0	0	0
0	0	0	0	0	0	0	0	0	0
0	0	0	0	0	0	0	0	0	0
0	0	0	0	0	0	0	0	0	0
0	0	0	0	0	0	0	0	0	0
0	0	0	0	1	0	0	1	0	0
0	0	0	0	0	0	0	0	0	0
0	0	0	0	0	0	0	0	0	0
0	0	0	0	0	0	0	0	1	0
0	0	0	0	0	0	0	0	0	0

0	0	0	0	0	0	0	0	0	0
0	0	0	0	0	0	0	0	0	0
0	0	0	0	0	0	0	0	1	0
0	0	0	0	1	1	0	0	0	0
0	0	0	0	1	1	0	0	0	0
0	0	1	0	0	1	0	0	0	0
0	0	1	0	0	0	0	1	0	0
0	0	0	0	1	1	0	0	0	0
0	0	0	0	0	1	0	0	0	0
0	0	0	0	0	0	0	0	0	0
0	0	0	0	0	1	0	0	0	0
0	0	0	0	0	0	0	0	0	0
0	0	0	0	0	0	0	0	0	0
0	0	0	0	0	0	0	0	0	0
0	0	0	0	0	0	0	0	0	0
0	0	0	0	0	0	0	0	0	0
0	0	0	0	0	1	0	0	0	0
0	0	0	0	0	0	0	0	0	0
0	0	0	0	0	1	0	0	0	0
0	0	0	0	0	0	0	0	0	0
0	0	1	0	0	0	0	0	0	0
0	0	0	0	0	0	0	0	0	0
0	0	0	0	0	0	0	0	0	0
0	0	0	0	0	0	0	0	0	0
0	0	0	0	0	0	0	0	0	0
0	0	0	0	0	0	0	0	0	0
0	0	0	0	0	0	0	0	0	0
0	0	0	0	0	0	1	0	0	0
0	0	0	0	0	0	1	0	0	0
0	0	0	0	0	0	0	0	0	0
0	0	0	0	0	0	0	0	0	0
0	0	0	0	0	0	0	0	0	0
0	0	1	0	0	0	0	0	0	0
0	0	0	0	0	0	0	0	0	0
0	0	0	0	0	0	0	0	0	0
0	0	0	0	0	0	0	0	0	0
0	0	0	0	0	0	0	0	0	0
0	0	0	0	0	0	0	0	0	0
0	0	0	0	0	0	0	0	0	0
0	0	0	0	0	0	0	0	0	0
0	0	0	0	0	0	0	0	0	0
0	0	0	0	0	0	0	0	0	0
0	0	0	0	0	0	0	0	0	0
0	0	0	0	0	0	0	0	0	0
0	0	0	0	0	0	0	0	0	0
0	0	0	0	0	0	0	0	0	0
0	0	0	0	0	0	0	0	0	0

0	1	0	0	0	1	0	0	0	0
0	1	1	0	0	1	0	0	0	0
0	0	0	0	0	0	0	0	0	0
0	0	0	0	0	0	0	0	0	0
0	0	1	1	0	0	0	0	0	0
0	0	1	0	0	0	0	0	0	0
0	0	0	0	0	0	0	0	0	0
0	0	0	0	0	0	0	0	0	0
0	0	0	0	0	0	0	0	0	0
0	0	0	0	0	0	0	0	0	0
0	0	0	0	0	0	0	0	0	0
0	0	0	0	0	0	0	0	0	0
0	0	0	0	0	0	0	0	0	0
0	0	0	0	0	0	0	0	0	0
0	0	0	0	0	1	0	0	0	0
0	1	0	0	0	1	0	0	0	0
0	0	0	0	0	1	0	0	0	0
0	0	0	0	0	0	0	0	0	0
0	0	0	0	0	0	0	0	0	0
0	0	0	0	0	0	0	0	0	0
0	0	0	0	0	0	0	0	0	0
0	0	0	0	0	0	0	0	0	0
0	0	0	0	0	1	0	0	0	0
0	0	0	0	0	0	0	0	0	0
0	0	0	0	0	1	0	0	0	0
0	0	0	0	0	0	0	0	0	0
0	0	0	0	0	0	0	0	0	0
0	0	0	0	0	1	0	0	0	0
0	0	0	0	0	0	0	0	0	0
0	0	1	0	0	0	0	0	0	0
0	0	0	0	0	0	0	0	0	0
0	0	0	0	0	0	0	0	0	0
0	0	0	0	0	0	0	0	0	0
0	0	0	0	0	0	0	0	0	0
0	0	0	0	0	0	0	0	0	0
0	0	1	0	0	0	0	0	0	0
0	0	0	0	0	0	0	0		0
0	0	0	0	0	0	0	0	0	0
0	0	0	0	0	1	0	0	0	0
0	0	0	0	0	0	0	0	0	0
0	0	0	0	0	0	0	0	0	0
0	0	0	0	0	0	0	0	0	0
0	0	0	0	0	0	0	0	0	0
0	0	0	0	0	0	0	0	0	0
0	0	0	0	0	0	0	0	0	0
1	0	1	0	0	0	0	0	0	0
0	0	0	0	0	0	0	0	0	0

0	0	0	0	0	0	0	0	0	0
0	0	0	0	0	0	0	0	0	0
0	0	0	0	0	0	0	0	0	0
0	0	0	0	0	0	0	0	0	0
0	0	0	0	0	0	0	0	0	0
0	0	0	0	0	1	0	0	0	0
0	0	0	0	0	0	0	0	0	0
0	0	0	0	0	0	0	0	0	0
0	0	0	0	0	1	0	0	0	0
0	0	0	0	0	1	0	0	0	0
0	0	1	0	0	1	0	0	0	0
0	0	0	0	0	0	0	0	0	0
0	0	0	0	0	0	0	0	0	0
0	0	0	1	0	0	0	0	0	0
0	0	0	0	0	0	0	0	0	0
0	0	0	0	0	0	0	0	0	0
0	0	0	0	0	0	0	0	0	0
0	0	0	0	0	0	0	0	0	0
0	0	0	0	0	0	0	0	0	0
0	0	0	0	0	0	0	0	0	0
0	0	0	0	0	0	0	0	0	0
0	0	0	0	0	0	0	0	0	0
0	0	0	0	0	0	0	0	0	0
0	0	0	0	0	0	0	0	0	0
0	0	0	0	0	0	0	0	0	0
0	0	0	0	0	0	0	0	0	0
0	0	0	0	0	0	0	0	0	0
0	0	0	0	0	0	0	0	0	0
0	0	0	0	0	0	0	0	0	0
0	0	0	0	0	0	0	0	0	0
0	0	0	0	0	0	0	1	0	0
0	0	0	0	0	0	0	0	0	0
0	0	0	0	0	0	0	0	0	0
0	0	0	0	0	0	0	0	0	0
0	0	0	0	0	1	0	0	0	0
0	0	0	1	0	0	0	1	1	0
0	0	0	1	0	1	0	0	0	0
0	0	0	0	0	1	0	1	1	0
0	0	0	0	0	0	0	0	0	0
0	0	0	0	0	0	0	0	0	0
0	0	0	0	0	0	0	0	0	0
0	0	0	0	0	0	0	0	0	0
0	1	0	0	0	0	0	0	0	0
0	0	1	1	0	1	0	1	0	0
0	0	0	0	0	0	0	0	0	0
0	0	0	0	0	0	0	0	0	0
0	0	0	0	0	0	0	0	0	0

0	0	1	0	0	0	0	0	0	0
0	0	0	0	0	0	0	1	0	0
0	0	0	0	0	1	0	0	0	0
0	0	0	0	0	0	0	0	0	0
0	0	0	0	0	0	0	0	0	0
0	0	0	0	0	0	0	0	0	0
0		0	0	0	0	0	0	0	0
0		0	0	0	0	0	0	0	0
0	0	0	0	0	0	0	0	0	0
0	0	0	0	0	0	0	0	0	0
0	0	0	0	0	0	0	0	0	0
0	0	0	0	0	0	0	0	0	0
0	0	0	0	0	0	0	0	0	0
0	0	0	0	0	0	0	0	0	0
0		0	0	0	0	0	0	0	0
0		0	0	0	0	0	0	0	0
0	0	0	0	0	1	0	0	0	0
0		0	0	0	1	0	0	0	0
0		0	0	0	1	0	0	0	0
0	0	0	0	0	0	0	0	0	0
0	0	0	0	0	0	0	0	0	0
0		1	1	1	1	1	0	0	0
1		1	0	0	1	0	0	0	0
0	0	0	1	0	1	0	1	0	0
0	0	0	0	0	0	0	1	0	0
0		0	0	0	0	0	0	0	0
0		0	0	0	0	0	0	0	0
0	0	0	0	0	0	0	0	0	0
0	0	0	1	0	0	0	0	0	0
0	1	0	0	1	0	0	0	0	0
0	0	1	1	0	0	0	0	0	0
0	0	0	0	0	1	0	0	0	0
0	0	0	0	0	1	0	0	0	0
0	0	0	0	0	0	0	0	0	0
0		0	0	0	0	0	0	0	0
1	0	0	0	0	0	0	0	0	0
0	0	0	0	0	0	0	0	0	0
0	0	0	0	0	0	0	0	0	0
0	0	0	0	0	1	0	0	0	0
0	0	1	0	0	0	0	0	0	0
0	0	0	0	0	0	0	0	0	0
0	0	0	0	0	0	0	0	0	0
0	0	0	0	0	0	0	0	0	0
0	0	0	0	0	0	0	0	0	0
0	0	0	0	0	0	0	0	0	0
0	0	0	0	0	0	0	0	0	0
0	0	0	0	1	0	0	0	0	0

0	0	0	0	0	0	0	0	0	0
0	0	0	0	0	0	0	0	0	0
0	0	0	0	0	0	0	0	0	0
0	0	0	0	0	1	1	0	0	0
0	0	0	0	0	0	0	0	0	0
0	0	0	0	0	0	0	0	0	0
0	0	1	1	0	1	0	0	0	0
0	0	0	0	0	0	0	0	0	0
0	0	0	0	0	0	0	0	0	0
1	0	0	0	0	0	0	0	0	1
0	0	0	0	0	0	0	0	0	0
0	0	0	0	0	1	0	1	0	0
0	0	0	0	0	0	0	0	0	0
0	0	0	0	0	0	0	0	0	0
0	0	0	0	0	0	0	0	0	0
0	0	1	1	0	0	0	0	0	0
0	0	0	0	1	1	0	1	0	0
0	0	0	0	0	0	0	0	0	0
0	0	0	0	0	0	0	0	0	0
0	0	0	0	0	1	0	0	0	0
0	0	0	0	0	0	0	0	0	0
0	0	0	0	0	1	0	0	0	0
0	0	0	0	0	0	0	0	0	0
0	0	0	0	0	0	0	0	0	0
0	0	0	0	0	0	0	1	0	0
0	0	1	0	0	1	0	0	0	0
0	0	0	0	0	0	0	0	0	0
0	0	0	0	0	0	0	0	0	0
0	0	0	0	0	0	0	0	0	0
0	0	0	0	0	1	0	0	0	0
0	0	1	0	0	0	0	0	0	0
0	0	0	0	1	1	0	0	0	0
0	0	0	0	0	0	0	0	0	0
0	0	0	0	0	1	0	0	0	0
0	0	0	0	0	1	0	0	0	0
0	0	0	0	0	1	0	0	0	0
0	0	0	0	0	1	0	0	0	0
0	0	0	0	0	0	0	0	0	0
0	0	0	0	0	0	0	0	0	0
0	0	0	0	0	1	0	0	0	0
0	0	0	0	0	1	0	0	0	0
0	0	0	0	0	0	0	0	0	0
0	0	0	0	0	1	0	0	0	0
0	0	0	0	0	0	0	0	0	0
0	0	0	0	0	0	0	0	0	0
0	0	0	0	0	0	0	0	0	0
0	0	0	0	0	0	0	0	0	0

0	0	0	0	0	0	0	0	0	0
0	0	0	0	0	1	0	0	0	0
0	0	0	0	0	0	0	0	0	0
0	0	1	1	1	0	0	0	0	0
0	0	0	0	0	1	0	0	0	0
0	0	0	0	0	0	0	0	0	0
0	0	0	0	0	0	0	0	0	0
0	0	0	0	0	0	0	0	0	0
0	0	0	0	0	0	0	0	0	0
0	0	0	0	0	0	0	0	0	0
0	0	0	0	0	0	0	0	0	0
0	0	0	0	0	0	0	0	0	0
0	0	0	0	0	0	0	0	0	0
0	0	0	0	0	1	0	0	0	0
0	0	0	0	0	0	0	0	0	0
0	0	0	1	0	0	0	0	0	0
0	0	1	0	1	0	0	0	0	0
0	0	0	0	0	1	0	0	0	0
0	0	0	0	0	0	0	0	0	0
0	0	0	0	0	0	0	0	0	0
0	0	1	0	0	1	0	0	0	0
0	0	0	0	0	0	0	0	0	0
0	0	0	0	1	1	0	0	0	0
0	0	1	0	0	0	0	0	0	0
0	0	1	1	0	0	0	0	0	0
0	0	0	0	0	0	0	0	0	0
0	0	1	1	0	1	0	0	0	0
0	0	0	0	0	1	0	0	0	0
0	0	0	0	0	1	0	0	0	0
0	0	0	0	0	0	0	0	0	0
0	1	0	0	1	1	0	0	0	0
0	0	0	0	0	0	0	0	0	0
0	0	0	0	0	0	0	0	0	0
0	0	0	0	0	1	0	0	0	0
0	0	0	0	0	0	0	0	0	0
0	0	0	0	1	1	0	0	0	0
0	0	0	0	0	1	0	0	0	0
1	0	0	0	0	0	0	0	0	0
0	0	0	0	0	0	0	0	0	0
0	0	0	0	0	0	0	0	0	0
0	0	0	0	0	0	0	0	0	0
0	0	0	0	0	0	0	0	0	0
0	0	0	0	0	0	0	0	0	0
0	0	0	0	0	0	0	0	0	0
0	0	0	0	0	0	0	1	1	0
0	0	0	0	0	0	0	0	0	0
1	0	0	1	0	0	0	0	0	0

0	0	0	0	0	0	0	0	0	0
0	0	0	0	0	0	0	0	0	0
0	0	0	0	0	0	0	0	0	0
0	0	0	0	0	0	0	0	0	0
0	0	0	0	0	0	0	0	0	0
0	0	0	0	0	0	0	0	0	0
0	0	0	0	0	0	0	0	0	0
0	0	0	0	0	0	0	0	0	0
0	0	0	0	0	0	0	0	0	0
1	0	1	1	1	0	1	0	0	0
0	0	0	0	0	0	0	0	0	0
0	0	0	0	0	0	0	0	0	0
0	0	0	0	0	0	0	0	0	0
0	0	0	0	0	1	0	0	0	0
0	0	0	0	1	1	0	0	0	0
0	0	0	0	0	0	0	0	0	0
0	0	0	0	0	0	0	0	0	0
0	0	0	0	0	0	0	0	0	0
0	0	0	0	0	0	0	0	0	0
0	0	0	0	0	0	0	0	0	0
0	0	0	0	0	0	0	1	0	0
0	0	0	0	0	0	0	0	0	0
0	0	0	0	0	0	0	0	0	0
0	0	0	0	0	0	0	0	0	0
0	0	0	0	0	0	0	0	0	0
0	0	0	0	0	0	0	0	0	0
0	0	0	0	0	1	0	0	0	0
0	0	0	0	0	0	0	1	0	0
0	0	0	0	0	0	0	0	0	0
0	0	0	0	1	1	0	0	0	0
0	0	0	0	0	1	0	0	0	0
0	0	0	0	0	0	0	0	0	0
0	0	0	0	0	0	0	0	0	0
0	0	0	0	0	0	0	0	0	0
0	0	0	0	0	0	0	0	0	0
0	0	0	0	0	0	0	0	0	0
0	0	0	0	0	0	0	0	0	0
0	0	0	0	0	0	0	0	0	0
0	0	0	0	0	0	0	0	0	0
0	0	0	0	0	0	0	0	0	0
0	0	0	0	0	0	0	0	0	0
0	0	0	0	0	0	0	0	0	0
0	0	0	0	0	0	0	0	0	0
0	0	0	0	0	0	0	0	0	0
0	0	0	0	0	0	0	0	0	0
0	0	0	0	0	0	0	0	0	0
0	0	1	0	0	0	0	0	0	0

0	0	0	0	0	0	0	0	0	0
0	0	0	1	1	1	0	0	0	0
0	0	0	0	0	0	0	0	0	0
0	1	0	0	0	0	0	0	0	0
0	0	0	0	0	0	0	0	0	0
0	0	0	0	0	0	0	0	0	0
0	0	0	0	0	0	0	0	0	0
0	0	0	0	0	0	0	0	0	0
0	0	0	0	0	0	0	0	0	0
0	0	0	0	0	0	0	0	0	0
0	0	0	0	0	0	0	0	0	0
0	0	0	0	0	0	0	0	0	0
0	0	0	1	0	0	0	0	0	0
0	0	0	0	0	0	0	0	0	0
0	0	0	0	0	0	0	0	0	0
0	0	0	0	0	0	0	0	0	0
0	1	0	0	0	0	0	0	0	0
0	0	0	0	0	0	0	0	0	0
0	0	0	0	0	0	0	0	0	0
0	0	0	0	0	0	0	0	0	0
0	0	0	0	0	1	0	0	0	0
0	0	0	0	1	1	0	1	0	0
0	0	0	0	0	1	0	0	0	0
0	0	0	0	0	0	0	0	0	0
0	0	0	0	0	0	0	0	0	0
0	0	0	0	0	0	0	0	0	0
1	1	0	0	0	1	1	1	1	1
0	0	0	0	0	1	0	0	0	0
0	0	0	0	0	0	0	0	0	0
0	0	0	0	0	0	0	0	0	0
0	0	0	0	1	1	0	0	1	0
0	0	0	0	0	0	0	0	0	0
0	0	0	0	0	0	0	0	0	0
0	0	0	0	0	1	0	0	0	0
0	0	0	0	0	0	0	0	0	0
0	1	1	1	0	0	0	0	0	0
0	0	0	0	0	0	1	0	0	0
0	0	0	0	0	0	0	0	0	0
0	0	0	0	0	0	0	0	0	0
0	0	0	0	0	0	0	0	0	0
0	0	0	0	0	1	0	0	0	0
0	0	1	1	0	0	0	0	0	0
0	0	0	0	0	0	0	0	0	0
0	0	0	1	0	0	0	0	0	0
0	0	0	0	0	0	0	0	0	0
0	0	0	0	0	0	0	0	0	0
0	0	0	0	0	1	0	0	0	0

0	0	0	0	0	0	0	0	0	0
0	0	0	0	0	0	0	0	0	0
0	0	0	1	0	1	0	0	1	0
0	0	0	0	0	1	0	0	0	0
0	0	0	0	0	1	0	1	0	0
0	0	0	0	0	1	0	0	0	0
0	0	0	1	0	1	0	0	0	0
0	0	1	0	0	0	0	0	0	0
0	0	0	0	0	0	0	0	0	0
0	0	0	0	0	1	0	0	0	0
0	0	0	0	0	0	0	0	0	0
0	0	0	0	0	0	0	0	0	0
0	0	1	1	0	0	0	0	0	0
0	0	0	0	0	0	0	0	0	0
0	0	1	0	0	0	0	0	0	0
0	0	0	0	0	0	0	0	0	0
0	0	0	0	0	0	0	1	0	0
0	0	0	0	0	0	0	0	0	0
0	1	0	1	0	0	1	0	0	0
0	0	0	0	0	0	0	0	0	0
0	0	0	0	0	1	0	0	0	0
0	0	0	0	0	1	0	0	0	0
0	0	1	0	0	0	0	0	0	0
0	0	0	0	0	0	0	0	0	0
0	0	0	0	0	0	0	0	0	0
0	0	0	0	0	0	0	0	0	0
0	0	0	0	0	0	0	0	0	0
0	0	0	0	0	1	0	0	0	0
0	0	0	0	0	1	0	1	0	0
0	0	0	0	0	1	0	0	0	0
0	0	0	0	0	1	0	0	1	0
0	0	0	0	0	0	0	0	0	0
0	0	0	0	0	0	0	0	0	0
0	0	1	1	0	0	0	0	0	0
0	0	0	0	0	1	0	0	0	0
0	0	0	1	0	0	0	0	0	0
0	0	0	0	0	0	0	0	0	0
0	0	0	0	0	0	1	0	0	0
0	0	0	0	0	0	0	0	0	0
0	0	0	0	0	0	0	0	0	0
0	0	0	0	0	0	0	0	0	0
0	0	0	0	0	0	0	0	0	0
0	0	0	0	0	0	0	0	0	0
0	0	0	0	0	1	1	0	0	0
0	0	0	0	0	1	0	0	0	0
0	0	0	0	0	0	0	1	0	0
0	0	0	0	0	0	0	0	0	0

0	0	0	0	0	0	0	0	0	0
0	0	0	0	0	0	0	0	0	0
0	0	0	0	0	0	0	0	0	0
0	0	0	0	0	0	0	0	0	0
0	0	0	0	0	0	0	0	0	0
0	0	0	0	0	0	0	0	0	0
0	0	0	0	0	0	0	1	0	0
0	0	0	0	0	0	0	0	1	0
0	0	0	0	0	0	0	0	0	0
0	0	0	0	0	0	0	0	0	0
0	0	0	0	0	0	0	0	0	0
0	0	0	0	0	0	0	0	0	0
0	0	1	0	0	0	0	0	0	0
0	0	0	0	0	0	0	0	0	0
0	0	0	0	0	0	0	0	0	0
0	0	0	0	0	0	0	0	0	0
0	0	0	0	0	0	0	1	0	0
0	0	0	0	0	0	0	0	0	0
0	0	0	0	0	0	0	0	0	0
0	0	0	0	0	0	0	0	0	0
0	0	0	0	0	0	0	0	0	0
0	0	0	0	0	0	0	1	0	0
0	0	0	0	0	0	0	0	0	0
0	0	0	0	0	0	0	1	0	0
0	0	0	0	1	1	0	1	1	0
0	0	0	0	1	1	0	0	0	0
0	0	0	0	0	0	0	0	0	0
0	0	0	0	0	0	0	0	0	0
0	0	1	1	0	1	0	1	0	0
0	0	0	1	0	1	0	0	0	0
0	0	1	0	0	0	0	0	0	0
0	0	0	0	0	0	1	0	0	0
0	0	0	0	0	0	0	0	0	0
0	0	0	1	1	1	0	0	1	0
1	0	1	0	0	1	1	1	0	0
0	0	0	0	0	0	0	0	0	0
0	0	1	1	1	1	0	0	0	0
0	0	0	0	0	0	0	0	0	0
0	0	0	0	1	1	0	1	0	0
0	0	0	0	0	0	0	1	0	0
0	0	0	0	0	1	0	0	0	0
0	0	1	0	0	0	0	0	0	0
0	0	0	0	0	0	0	0	0	0
0	0	1	0	0	0	0	0	1	0
0	0	0	0	0	0	0	0	0	0
0	0	1	1	0	1	0	0	0	0
0	0	0	1	0	1	0	0	1	0

0	0	0	0	0	0	0	1	0	0
0	0	0	0	0	0	0	0	0	0
0	0	0	0	0	0	0	0	0	0
0	0	0	0	0	0	1	0	0	0
0	0	0	0	0	0	0	0	0	0
0	0	0	0	1	1	0	0	0	0
0	0	0	0	0	0	0	0	0	0
0	0	0	0	0	0	0	0	0	0
0	0	0	0	0	0	0	0	0	0
0	0	0	0	0	0	0	0	0	0
0	0	0	0	1	0	0	0	0	0
0		0	0	0	0	0	0	0	0
0	0	0	0	0	0	0	0	0	0
0		0	0	0	0	0	1	1	0
0	0	0	0	0	0	0	0	0	0
0	0	1	0	0	0	0	0	0	0
0	0	0	0	0	0	0	0	0	0
0		0	0	0	0	0	0	0	0
0		0	0	0	0	0	0	0	0
0	0	1	1	0	1	0	0	0	0
1	1	1	1	0	0	0	1	0	0
0	0	0	0	0	0	0	0	0	0
0		0	0	0	0	0	0	0	0
0	0	0	0	0	0	0	0	0	0
0	0	0	0	0	0	0	0	0	0
0	0	0	1	0	0	0	0	0	0
0		1	0	1	1	1	1	0	0
0		0	0	0	1	0	0	0	0
0	0	0	0	0	0	0	0	0	0
0	0	0	0	0	0	0	0	0	0
0	0	0	0	0	0	0	0	0	0
0	0	0	0	0	0	0	0	0	0
0	0	0	0	0	0	0	0	0	0
0		0	0	0	0	0	0	0	0
0	0	0	0	0	0	0	0	0	0
0	0	0	0	0	0	0	0	0	0
0		0	0	0	0	0	0	0	0
0	0	0	0	0	0	0	0	1	0
0	0	0	0	0	0	0	0	0	0
0	0	0	0	0	0	0	0	0	0
0	0	0	0	0	0	0	0	0	0
0	0	0	0	0	1	0	0	0	0
0	1	1	1	0	0	0	0	0	0
0	0	0	0	0	0	0	0	0	0
0		0	0	0	0	0	0	0	0
0		0	0	0	0	0	0	0	0
0	0	0	0	1	0	0	0	0	0

0	0	0	1	1	1	0	0	0	0
0	0	0	0	0	0	0	0	0	0
0	0	0	0	0	0	0	0	0	0
0		0	1	0	1	0	0	0	0
0	0	0	0	0	0	0	0	0	0
0	0	0	0	0	0	0	0	0	0
0	0	0	0	0	0	0	0	0	0
0		1	1	0	0	1	1	1	0
0		0	0	0	0	0	0	0	0
0		0	0	0	0	0	0	0	0
0	0	1	1	0	0	0	0	0	0
0	0	0	0	0	0	0	0	0	0
0	0	0	0	0	0	0	0	0	0
0	0	0	0	1	1	1	0	1	0
0	0	0	0	0	0	0	0	0	0
0	0	0	0	0	0	0	0	0	0
0	0	0	0	0	0	0	0	0	0
0	0	0	0	0	0	0	0	0	0
0	1	0	1	0	0	0	0	1	0
0	0	0	0	0	0	0	0	0	0
0	0	0	0	0	0	0	0	0	0
0	0	0	0	0	0	0	0	0	0
0	0	0	0	0	0	0	0	0	0
0	0	0	0	0	0	0	0	0	0
0	0	0	0	0	0	0	0	0	0
1	0	0	0	0	0	1	1	0	0
0	0	0	0	0	0	0	0	0	0
0	0	0	0	0	0	0	0	0	0
0	0	0	1	0	0	0	0	0	0
0	0	0	0	0	0	0	0	0	0
0	0	0	0	0	0	0	0	0	0
0	0	0	0	0	0	0	0	0	0
0	0	0	0	0	0	0	0	0	0
0	0	0	0	0	1	0	0	0	0
0	0	0	0	0	0	0	0	0	0
0	0	0	0	0	1	0	0	0	0
0	0	0	0	0	0	0	0	0	0
0	0	0	0	0	0	0	0	0	0
0	0	0	0	1	0	0	1	0	0
0	0	0	0	0	0	0	0	0	0
0	0	0	1	1	1	0	0	0	0
0	0	0	0	0	0	0	0	0	0
0	0	1	0	0	1	0	1	0	0
0	0	0	0	0	1	0	0	0	0
0	0	0	0	0	0	0	0	0	0
0	0	0	0	0	0	0	0	0	0
0	0	0	0	0	0	0	0	0	0

0	0	0	0	0	1	0	0	0	0
0	0	1	0	1	1	0	1	0	0
0	0	0	0	0	0	0	0	0	0
0	1	0	0	1	0	0	0	0	0
0	0	0	0	0	0	0	0	0	0
0	0	0	1		1	0	0	0	0
0	0	0	1	0	0	0	1	0	0
0	0	0	1	0	0	0	1	0	0
0	0	0	0	0	0	0	0	0	0
0	0	0	0	0	0	0	0	0	0
0	0	0	0	0	1	1	0	0	0
0	0	0	0	0	1	0	0	0	0
0	0	0	0	0	0	1	0	0	0
0	0	0	0	0	1	0	0	0	0
0	0	0	0	0	0	0	0	0	0
0	0	0	0	0	0	0	0	0	0
1	0	0	0	0	1	0	0	0	0
0	0	0	0	0	0	0	0	0	0
0	0	1	0	0	0	0	0	0	0
0	0	0	0	0	0	0	0	0	0
0	0	0	1	0	0	0	0	0	0
0	0	0	0	0	0	0	1	0	0
0	0	0	0	0	0	0	0	0	0
0	0	0	0	0	0	0	0	0	0
0	0	0	0	0	1	0	0	0	0
0	0	0	0	0	0	0	0	0	0
0	0	0	1	0	1	1	0	0	0
0	0	1	0	1	1	0	0	1	0
0	0	0	0	0	1	0	0	0	0
0	0	1	0	0	1	0	0	0	0
0	0	0	0	0	0	0	0	0	0
0	0	1	0	0	0	0	0	0	0
0	0	1	0	0	0	0	0	0	0
0	0	0	0	0	0	0	0	0	0
0	0	0	0	0	1	0	0	0	0
0	0	0	0	0	1	0	0	0	0
0	0	0	0	0	0	0	0	0	0
0	0	0	0	0	0	0	0	0	0
0	0	0	0	1	0	0	1	0	0
0	0	0	0	0	0	0	0	0	0
0	0	0	0	0	0	0	0	0	0
0	0	0	0	0	0	0	0	0	0
0	0	0	0	0	0	0	0	0	0
1	0	0	0	0	0	0	0	0	0
0	0	0	0	1	1	0	0	0	0
0	0	0	0	0	0	0	0	0	0
0	0	0	0	0	0	0	0	0	0

0	0	0	0	0	0	0	0	0	0
0	0	0	0	0	0	0	0	0	0
0	0	0	0	0	0	0	0	0	0
0	0	0	0	0	1	0	0	0	0
0	0	0	0	0	1	0	0	0	0
0	0	0	0	0	0	0	0	0	0
0	0	0	0	0	0	0	0	0	0
0	1	0	0	0	1	0	0	0	0
0	0	0	0	0	1	0	0	0	0
0	0	0	0	0	0	0	1	0	0
0	0	0	0	0	0	0	0	0	0
0	0	0	0	0	0	0	0	0	0
0	0	0	0	0	0	0	0	0	0
0	0	0	0	0	1	0	0	0	0
0	0	0	0	0	1	1	1	0	0
0	0	1	1	1	1	0	0	0	0
0	0	0	0	0	1	0	1	0	0
0	0	0	0	0	1	0	0	0	0
0	0	0	1	1	1	0	0	0	0
0	0	0	0	0	0	0	0	0	0
0	0	0	0	0	1	0	0	0	0
0	0	0	1	1	1	0	0	0	0
0	0	0	1	1	1	0	0	0	0
0	0	0	0	0	0	0	0	0	0
0	0	0	0	0	0	0	0	0	0
0	0	0	0	0	1	0	0	0	0
0	0	0	0	0	0	0	0	0	0
0	0	0	0	1	0	0	0	0	0
0	0	0	0	0	1	0	0	0	0
0	0	0	0	0	0	0	0	0	0
0	0	0	0	0	0	0	0	0	0
0	0	0	1	0	0	0	0	0	0
0	0	0	0	1	1	0	0	0	0
0	0	0	0	1	1	0	0	0	0
0	0	1	1	0	1	0	0	0	0
0	0	0	0	1	0	0	0	0	0
0	0	0	0	1	0	0	0	0	0
0	0	0	0	0	0	0	0	0	0
0	0	0	1	0	1	0	0	0	0
0	0	0	0	1	0	0	0	0	0
0	0	1	0	0	1	0	1	0	0
0	0	0	0	1	0	0	0	1	0
0	0	1	0	1	1	0	0	0	0
0	0	0	0	1	1	0	0	0	0
0	0	0	0	0	0	0	0	0	0
0	0	0	0	0	0	0	0	0	0
0	0	0	0	1	1	0	0	0	0

0	0	0	0	0	0	0	0	0	0
0	0	0	0	0	1	0	0	0	0
0	0	0	0	0	1	0	0	0	0
0	0	0	0	0	0	0	0	0	0
0	0	0	0	0	0	0	0	0	0
0	0	0	0	0	0	0	0	0	0
0	0	0	0	0	0	0	0	0	0
0	0	0	0	0	0	0	0	0	0
0	0	0	0	0	0	0	0	0	0
0	0	0	0	0	0	0	0	0	0
0	0	0	0	0	0	0	0	0	0
0	0	0	0	0	0	0	0	0	0
0	0	0	0	0	0	0	1	1	0
0	0	0	0	0	0	0	0	0	0
0	0	0	0	0	0	0	0	0	0
0	0	0	0	0	0	0	0	0	0
0	0	0	0	0	0	0	0	0	0
0	0	0	0	0	0	0	0	0	0
0	0	0	0	0	0	0	0	0	0
0	0	0	0	0	1	0	0	0	0
0	0	0	0	0	0	0	0	0	0
0	0	0	0	0	0	0	0	0	0
0	0	0	0	0	0	0	0	0	0
0	0	0	0	0	0	0	1	0	0
0	0	0	0	0	0	0	0	0	0
0	0	0	0	0	0	0	0	0	0
0	0	0	0	0	0	0	0	0	0
0	0	0	0	0	0	0	0	0	0
0	0	0	0	0	0	0	0	0	0
0	0	0	0	0	0	0	0	1	0
0	0	0	0	0	0	0	0	0	0
0	0	0	0	0	1	1	1	0	0
0	0	0	0	0	0	0	0	0	0
0	0	0	0	0	0	0	0	0	0
0	0	0	0	0	0	0	0	0	0
0	0	0	0	0	0	0	0	0	0
0	0	0	0	0	0	0	0	0	0
0	0	0	0	0	0	0	0	0	0
0	0	0	0	0	0	0	0	0	0
0	0	0	0	0	0	0	0	0	0
0	0	0	0	0	0	0	0	0	0
0	0	0	0	0	0	0	0	0	0
0	0	0	0	0	0	0	0	0	0
0	0	0	0	0	0	0	0	0	0
0	0	0	0	0	0	0	0	0	0
0	0	0	0	0	0	0	0	1	0
0	0	0	0	0	0	0	0	0	0

0	0	0	0	0	0	0	0	0	0
0	0	1	1	0	0	0	0	0	0
0	0	0	0	0	0	0	0	0	0
0	0	0	0	0	1	0	0	1	0
0	0	0	0	0	0	0	0	1	0
0	0	0	0	0	0	0	0	0	0
0	0	0	0	0	0	0	0	0	0
0	0	0	0	0	0	0	0	0	0
1	0	0	0	0	0	0	0	0	0
0	0	0	0	0	1	0	0	0	0
0	0	1	0	0	0	0	0	0	0
0	0	0	0	0	0	0	0	0	0
0	0	0	0	0	0	0	0	0	0
0	0	0	0	0	0	0	0	0	0
0	0	0	0	1	1	0	0	0	0
0	0	0	0	0	0	0	1	0	0
0	0	0	0	0	0	0	0	0	0
0	0	0	0	0	0	0	0	0	0
0	0	0	0	0	0	0	0	0	0
0	0	0	0	0	1	0	0	0	0
0	0	0	0	0	0	0	0	0	0
0	0	0	0	0	0	0	0	0	0
0	0	0	0	0	1	0	0	0	0
0	0	0	0	0	1	0	0	0	0
0	0	0	0	0	0	0	0	0	0
0	0	0	0	0	1	0	0	0	0
0	0	0	0	0	0	0	0	0	0
0	0	0	0	0	0	1	1	0	0
0	0	0	0	0	0	0	1	0	0
0	0	0	0	0	0	0	0	0	0
0	0	0	0	0	0	0	1	0	0
0	0	0	0	0	0	0	0	0	0
0	0	0	0	0	0	0	0	0	0
0	0	0	0	0	0	0	0	0	0
0	0	1	0	1	0	0	0	0	0
0	0	0	0	0	0	0	0	0	0
0	0	0	0	0	0	0	0	0	0
0	0	0	0	0	0	0	0	0	0
0	0	0	0	0	0	0	0	0	0
0	0	0	0	0	0	0	0	0	0
0	0	0	0	1	0	0	0	0	0
0	0	0	0	0	1	0	0	0	0
0	0	0	0	0	0	0	0	0	0
0	0	0	0	0	1	0	0	0	0
0	0	0	0	0	0	0	0	0	0
0	0	0	0	0	0	0	1	0	0
0	1	0	0	0	0	0	0	0	0

0	0	0	0	0	0	0	0	0	0
0	0	0	0	0	1	0	0	0	0
0		0	0	0	0	0	0	0	0
0	0	0	0	0	0	0	0	0	0
0	0	0	0	0	0	0	0	0	0
0	0	0	0	0	0	0	0	0	0
0	0	0	0	0	0	0	0	0	0
0	0	0	1	0	0	0	0	0	0
0	0	0	0	0	0	0	0	0	0
0	0	0	0	0	0	0	0	0	0
0	0	0	0	0	0	0	0	0	0
0	0	0	0	0	0	0	0	0	0
0	0	0	0	0	0	0	0	0	0
0	0	0	0	0	0	0	0	0	0
0	0	0	0	0	0	0	0	0	0
0	0	0	0	0	0	0	0	0	0
0	0	0	1	0	0	0	0	0	0
0	0	0	0	0	0	0	0	0	0
0	0	0	0	0	0	0	0	0	0
0	0	0	0	0	0	0	0	0	0
0	0	0	0	0	0	0	0	0	0
0	0	0	0	0	0	0	0	0	0
0	0	0	0	0	0	0	0	0	0
0	0	0	0	0	0	0	0	0	0
0	0	0	0	0	0	0	0	0	0
0	0	0	0	0	0	0	0	0	0
0	0	0	0	0	0	0	0	0	0
0	0	0	0	0	0	0	0	0	0
0	0	0	0	0	0	0	0	0	0
0	0	0	0	0	0	0	0	0	0
0	0	0	0	0	0	0	0	0	0
0	0	0	0	0	0	0	1	0	0
0	0	0	0	0	0	0	0	0	0
0	0	1	1	0	1	0	0	0	0
0	0	0	0	0	0	0	1	0	0
0	0	0	0	0	0	0	0	0	0
0	0	0	0	0	0	0	0	0	0
0	0	0	0	0	0	0	0	0	0
0	0	0	0	0	0	0	0	0	0
0	0	0	0	0	0	0	0	0	0
0	0	0	0	0	0	0	0	0	0
0	0	0	0	0	0	0	0	0	0
0	0	0	0	0	0	0	0	0	0
0	0	0	0	0	0	0	0	0	0
0	0	0	0	0	0	0	0	0	0
0	0	0	0	0	0	0	0	0	0
0	0	0	0	0	0	0	0	0	0

0	0	0	0	0	0	0	0	0	0
0	0	0	0	0	0	0	0	0	0
0	0	0	0	0	0	0	0	0	0
0	0	0	0	0	0	0	0	0	0
0	0	0	0	0	0	0	0	0	0
0	0	0	0	0	0	0	0	0	0
0	0	0	0	0	0	0	0	0	0
0	0	0	0	0	0	0	0	0	0
0	0	0	0	0	0	0	0	0	0
0	0	0	0	0	0	0	0	0	0
0	0	0	0	0	0	0	0	0	0
0	0	0	0	0	0	0	0	0	0
1	0	1	1	1	0	0	0	1	1
0	0	0	0	0	0	0	0	0	0
0	0	0	0	0	0	0	0	0	0
0	0	0	0	0	0	0	0	0	0
0	0	0	0	0	1	0	0	0	0
0	0	1	1	0	1	0	1	0	0
0	0	0	0	0	1	0	0	0	0
0	0	1	1	1	0	0	0	0	0
0	0	0	0	0	0	0	0	0	0
0	0	0	0	0	0	0	0	0	0
0	0	0	0	0	0	0	0	0	0
0	0	0	0	0	0	0	0	0	0
0	0	1	0	0	1	0	1	0	0
0	0	0	0	0	0	0	0	0	0
0	0	0	0	0	0	0	0	0	0
0	0	0	0	0	0	0	0	0	0
0	0	1	0	0	0	0	0	0	0
0	0	0	0	0	0	0	0	0	0
0	0	0	0	0	0	0	0	0	0
0	0	1	1	0	0	0	0	0	0
0	0	0	1	0	0	0	0	0	0
0	0	0	0	0	0	0	0	0	0
0	0	0	0	0	0	0	0	0	0
0	0	0	0	0	1	0	0	0	0
0	1	0	0	0	1	0	0	0	0
0	0	0	1	0	0	0	0	0	0
0	0	0	0	0	0	0	0	0	0
1	0	0	1	1	0	0	0	0	0
0	0	0	0	0	0	0	1	0	0
0	0	0	0	0	0	0	0	0	0
0	0	0	0	0	0	0	0	0	0
0	0	0	0	0	1	0	0	0	0
0	0	0	1	0	0	0	0	0	0
0	0	1	1	0	0	0	0	0	0
0	0	0	0	0	0	0	0	0	0

0	0	0	0	0	0	0	0	0	0
0	0	0	0	0	1	0	0	0	0
0	0	0	0	0	0	0	0	0	0
0	0	0	1	0	1	0	0	0	0
0	0	0	0	0	0	0	0	0	0
0	0	0	0	0	0	0	0	0	0
0	0	0	0	0	0	0	0	0	0
0	0	0	0	0	0	0	0	0	0
0	0	0	0	0	1	0	0	0	0
0	0	0	0	0	1	0	1	0	0
0	0	0	1	1	1	0	1	0	0
0	0	0	0	0	0	0	1	0	0
0	0	0	0	0	1	0	0	0	0
0	0	0	0	0	0	0	0	0	0
0	0	0	0	0	0	0	0	0	0
0	0	0	0	0	0	0	0	0	0
0	0	0	1	0	0	0	0	0	0
0	0	0	0	0	0	0	0	0	0
0	0	1	1	0	1	0	1	0	0
0	0	0	0	1	1	0	0	0	0
0	0	1	1	0	1	0	0	0	0
0	0	0	0	0	0	0	0	0	0
0	0	0	1	0	0	0	0	0	0
0	0	0	0	0	0	0	0	0	0
0	0	1	1	0	1	0	0	0	0
0	0	0	0	0	0	0	0	0	0
0	0	0	0	0	0	0	0	0	0
0	0	0	0	0	0	0	0	0	0
0	1	1	0	0	0	0	1	0	0
0	1	0	0	0	1	0	1	0	0
0	0	0	1	1	1	0	0	0	0
0	0	0	0	0	0	0	0	0	0
0	0	1	1	1	0	0	0	0	0
0	0	0	0	0	0	0	0	0	0
0	0	0	0	0	0	0	1	0	0
0	0	0	0	0	1	0	0	0	0
0	0	1	0	0	1	0	0	0	0
0	0	0	0	0	1	0	0	0	0
0	1	0	0	0	0	0	0	0	0
0	0	0	0	0	0	0	0	0	0
0	0	0	0	0	0	0	0	0	0
0	0	0	0	0	0	0	0	0	0
0	0	0	0	0	0	0	0	0	0
0	0	0	0	0	1	0	0	0	0
0	0	0	0	0	0	0	0	0	0
0	0	0	0	0	1	0	0	0	0
0	0	0	0	0	1	0	0	0	0

0	0	0	0	0	0	0	0	0	0
0	1	0	0	0	0	0	0	0	0
0	0	0	0	0	0	0	0	0	0
0	0	0	0	0	0	0	0	0	0
0	0	0	0	0	0	0	0	0	0
0	0	0	0	1	0	0	0	0	0
0	0	0	0	0	0	0	0	0	0
0	0	0	0	0	0	0	0	0	0
0	0	0	0	0	0	0	0	0	0
0	0	0	0	0	0	0	0	0	0
0	0	0	0	0	0	0	0	0	0
0	0	0	0	0	0	0	0	0	0
0	0	0	0	0	0	0	0	0	0
0	0	0	0	0	0	0	0	0	0
0	0	0	0	1	0	0	0	0	0
0	0		0	0	0	0	0	0	0
0	0	0	0	0	0	0	0	0	0
0	0	1	0	0	0	0	0	0	0
0	0	0	0	0	1	0	0	0	0
0	0	1	0	0	1	0	0	0	0
0	0	0	0	0	0	0	0	0	0
0	0	0	0	0	0	0	0	0	0
0	0	0	0	1	0	1	0	0	0
0	0	0	0	0	0	0	0	0	0
0	0	0	0	0	0	0	0	0	0
0	0	0	0	0	0	0	0	0	0
0	0	0	0	0	0	0	0	0	0
0	0	0	0	0	0	0	0	0	0
0	0	0	0	0	1	0	0	0	0
0	0	0	0	0	0	0	0	0	0
0	0	0	0	0	1	0	1	0	0
0	0	0	0	0	0	0	0	0	0
0	0	0	0	0	0	0	0	0	0
0	0	0	0	0	0	0	0	0	0
0	0	0	0	0	0	0	0	0	0
0	0	0	0	0	0	0	0	0	0
0	0	0	0	0	0	0	0	0	0
0	0	0	0	0	0	0	0	0	0
0	0	0	0	0	0	0	0	0	0
0	0	0	0	0	1	0	0	0	0
0	0	0	0	0	0	0	0	0	0
0	0	0	0	0	0	0	0	0	0
0	0	0	0	0	0	0	1	0	0
0	0	0	0	0	0	0	0	0	0
0	0	0	0	0	0	0	0	0	0
0	0	0	0	0	0	0	0	0	0
0	0	0	0	0	0	0	0	0	0

0	0	0	0	0	1	0	0	0	0
0	0	0	0	0	1	0	0	0	0
0	0	0	0	0	1	0	0	0	0
0	0	0	0	0	0	0	0	0	0
0	0	0	1	0	0	0	0	0	0
0	0	0	0	0	0	0	1	0	0
0	0	0	0	0	0	0	0	0	0
0	0	0	0	0	1	0	0	0	0
0	0	0	0	0	0	0	0	0	0
0	0	1	0	0	0	0	0	0	0
0	0	0	0	0	0	0	0	0	0
0	0	0	0	0	0	0	0	0	0
0	0	0	0	0	0	0	0	0	0
0	0	0	0	0	0	0	0	0	0
0	0	0	0	0	0	0	0	0	0
0	0	0	0	0	0	0	0	0	0
0	0	0	0	0	0	0	0	0	0
0	0	0	0	0	0	0	0	0	0
0	0	0	0	0	0	0	0	0	0
0	0	0	0	0	0	0	0	0	0
0	0	0	0	0	0	0	0	0	0
0	0	0	1	0	0	0	1	1	0
0	0	0	0	0	1	0	1	1	0
0	0	0	0	0	0	0	0	0	0
0	0	0	0	0	0	0	0	0	0
0	0	0	0	0	0	0	0	0	0
0	0	0	0	0	0	0	0	0	0
0	0	0	0	0	0	0	0	0	0
0	0	0	0	0	0	0	0	0	0
0	0	0	0	0	0	0	0	0	0
0	0	0	0	0	0	0	0	0	0
0	0	0	0	0	0	0	0	0	0
0	0	0	0	0	0	0	0	0	0
0	0	0	0	0	0	0	0	0	0
0	0	0	0	0	0	0	0	0	0
0	0	0	1	0	0	0	0	0	0
0	0	0	0	0	0	0	0	0	0
0	0	0	0	0	0	0	0	0	0
0	0	0	0	0	0	0	1	1	0
0	0	0	0	0	0	0	0	0	0
0	0	0	0	0	0	0	0	0	0
0	0	0	0	0	0	1	0	0	0
0	1	0	0	0	0	0	0	0	0
0	0	0	0	0	0	0	0	0	0
0	0	0	1	0	0	0	0	1	0
0	0	0	0	0	1	0	1	0	0
0	0	0	0	0	0	0	0	0	0

0	0	1	0	0	0	0	0	0	0
1	0	0	0	1	1	0	0	0	0
0	0	0	1	0	1	0	0	0	0
0	0	0	0	0	0	0	0	0	0
0	0	0	0	0	0	1	0	0	0
0	0	0	0	0	0	0	0	0	0
0	0	0	1	0	0	0	1	0	0
0	0	0	0	0	0	0	0	0	0
0	0	0	0	0	0	0	0	0	0
0	0	0	0	0	1	0	0	0	0
0	0	1	0	0	1	1	1	0	0
0	0	0	0	0	0	0	0	0	0
0	0	0	0	0	0	0	0	0	0
0		0	0	0	0	0	0	0	0
0		0	0	0	0	0	0	0	0
0	0	0	0	0	0	0	0	0	0
0	0	0	0	0	0	0	0	0	0
0		0	0	0	0	0	0	0	0
0		1	0	1	1	0	0	0	0
0	0	0	0	0	0	0	0	0	0
0	0	0	0	0	0	0	0	0	0
0	0	1	0	0	1	0	0	0	0
0	0	1	0	0	1	0	0	0	0
0	0	0	0	0	0	0	0	0	0
0	1	1	1	1	1	1	1	0	0
0		0	0	0	0	0	0	0	0
0	0	0	0	0	0	0	0	0	0
0	0	0	0	0	0	0	0	0	0
0	0	0	0	0	1	0	0	0	0
0	0	0	0	0	1	0	0	0	0
0	0	0	0	0	0	0	0	0	0
0	0	0	0	0	0	0	0	0	0
0	0	0	0	0	1	0	0	0	0
0		1	0	0	1	0	0	0	0
0		0	0	0	1	0	0	0	0
0		0	0	0	1	0	0	0	0
0		0	0	0	1	0	0	0	0
0		0	0	0	0	0	0	0	0
0	0	0	0	0	1	0	1	0	0
0	0	0	0	0	1	0	0	0	0
0	0	0	0	0	0	0	0	0	0
0	0	0	0	0	0	0	0	0	0
0	0	0	0	0	0	0	0	0	0
0	0	0	0	0	0	0	0	0	0
0	0	0	1	0	0	0	0	0	0
0	0	0	0	0	0	0	0	0	0
0	0	0	0	0	0	0	0	0	0

0	0	0	0	0	0	0	0	0	0
0	0	0	0	0	0	0	0	0	0
0	0	0	0	0	0	0	0	0	0
0	0	0	0	0	0	0	0	0	0
0		0	0	0	0	0	0	0	0
0		0	0	0	0	0	0	0	0
0	0	0	0	1	0	0	0	0	0
0	0	0	0	0	0	0	0	0	0
0	0	0	1	0	0	0	0	0	0
0	0	0	0	0	0	0	0	0	0
0	0	0	0	0	0	0	0	1	0
0	0	0	0	0	1	1	0	0	0
0	0	0	0	0	0	0	0	0	0
0		0	0	0	1	0	0	0	0
0		0	0	0	0	0	0	0	0
0	0	0	0	0	1	0	0	0	0
0	0	0	0	0	0	0	0	0	0
0	0	0	0	0	0	0	0	0	0
0	0	0	0	0	0	0	0	0	0
0		0	0	0	0	0	0	0	0
0	1	0	0	1	0	0	0	0	0
0	0	1	1	0	0	0	1	0	0
0	0	0	0	0	0	0	0	0	0
0	0	0	1	0	0	0	0	0	0
0	0	0	0	0	0	0	0	0	0
0	0	0	0	0	0	0	0	0	0
0	0	0	1	0	0	0	0	0	0
0		0	0	0	0	0	1	0	0
0		1	0	0	1	0	0	0	0
0	0	0	0	0	0	0	0	0	0
0	0	0	0	0	0	0	0	0	0
0	0	0	0	1	1	0	0	0	0
0	0	0	0	0	0	0	0	0	0
0	0	0	0	0	1	0	0	0	0
0	0	0	0	0	0	0	0	0	0
0	0	0	0	0	1	0	0	0	0
0		0	0	0	0	0	0	0	0
0		1	1	0	1	0	1	0	0
0		1	0	0	1	0	0	0	0
0	0	1	0	0	1	0	0	0	0
0	0	0	0	0	0	0	0	0	0
0		0	0	0	0	0	0	0	0
0		0	1	1	1	0	0	0	0
0	0	0	0	0	0	0	0	0	0
0		0	0	0	1	0	0	0	0
0	0	0	0	0	0	0	0	0	0
0		0	0	0	0	0	1	0	0

0	0	0	0	0	0	0	0	0	0
0	0	0	0	0	0	0	0	0	0
0	0	0	0	0	0	0	0	0	0
0	0	0	0	0	0	0	0	0	0
0	0	0	0	0	0	0	0	0	0
0	0	0	0	0	1	0	0	0	0
0	1	0	1	0	1	1	0	0	0
0	0	0	0	0	0	0	0	0	0
0	0	0	0	0	0	0	0	0	0
0	0	1	0	0	0	0	0	0	0
0	0	0	1	0	1	0	0	0	0
0	0	0	0	0	0	0	0	0	0
0	0	0	0	0	0	0	0	0	0
0	0	0	0	0	0	0	0	0	0
0	0	0	0	0	0	0	1	0	0
0	0	0	0	0	0	0	0	0	0
0	0	0	0	0	0	0	0	0	0
0	0	0	0	0	0	0	0	0	0
0	0	0	0	0	0	0	0	0	0
0	0	0	0	0	0	0	0	0	0
0	0	0	0	0	0	0	0	0	0
0	0	0	0	0	1	0	0	0	0
0	0	0	0	0	0	0	0	0	0
0	0	1	1	0	0	0	0	0	0
0	0	0	0	0	0	0	0	0	0
0	0	0	0	0	0	0	0	0	0
0	0	0	0	0	0	0	0	0	0
0	0	0	0	0	1	0	0	0	0
0	0	0	1	1	1	0	0	0	0
0	0	0	1	1	1	0	0	0	0
0	0	0	0	0	0	0	1	0	0
0	0	0	0	0	1	0	0	0	0
0	0	0	0	0	0	0	0	0	0
0	0	0	0	0	1	0	0	0	0
0	0	1	1	0	1	0	0	0	0
0	0	1	1	1	1	0	0	0	0
0	0	0	0	0	0	0	0	0	0
0	0	0	0	0	0	0	0	0	0
0	0	0	0	0	0	0	0	0	0
0	0	0	0	1	1	0	1	0	0
0	0	1	1	0	0	0	1	0	0
0	0	0	0	0	0	0	0	0	0
0	0	1	1	1	1	0	1	1	0
0	0	0	0	0	0	0	0	0	0
0	0	0	0	0	0	0	0	0	0
0	0	0	0	0	0	0	0	0	0
0	0	0	0	0	0	0	0	0	0

0	0	0	0	0	0	0	0	0	0
0	0	0	0	1	1	0	1	1	0
0	0	1	1	1	1	0	1	0	0
0	0	0	0	0	0	0	0	0	0
0	0	0	0	0	0	0	0	0	0
0	0	1	1	0	1	1	1	0	0
0	0	0	0	0	0	0	1	0	0
0	0	0	0	0	0	0	0	0	0
0	0	0	0	0	0	0	0	0	0
0	0	0	0	0	1	0	1	1	0
0	0	0	0	1	1	0	0	0	0
0	0	0	0	1	1	0	0	0	0
0	0	1	1	1	0	0	0	0	0
0	0	0	0	1	0	0	0	0	0
0	0	1	0	0	1	0	0	0	0
1	0	1	1	1	1	0	0	0	0
0	0	0	0	0	0	0	0	0	0
0	0	0	0	0	0	0	0	0	0
0	0	0	0	0	0	0	0	0	0
0	0	0	0	0	0	0	0	0	0
0	0	0	0	0	1	0	0	0	0
0	0	0	0	0	0	0	0	0	0
0	0	0	0	0	1	0	0	0	0
0	0	0	0	0	0	0	0	0	0
1	0	0	0	0	1	0	0	0	0
0	0	0	0	1	1	0	0	0	0
0	0	0	0	0	0	0	0	0	0
0	0	0	0	0	0	0	0	0	0
0	0	0	0	0	0	0	0	0	0
0	0	0	0	0	0	0	0	0	0
0	0	1	0	0	0	0	0	0	0
1	0	0	0	1	1	0	1	0	0
0	0	0	0	0	1	0	0	0	0
0	0	0	1	0	0	0	0	0	0
0	0	0	0	0	1	0	0	0	0
0	0	1	1	1	1	0	1	0	0
1	0	0	0	1	1	0	0	0	0
0	0	0	0	0	0	0	0	0	0
0	0	0	0	0	0	0	0	0	0
0	0	0	0	0	0	0	0	0	0
0	0	0	1	1	1	0	0	0	0
0	0	0	1	1	1	0	0	0	0
0	0	0	0	0	0	0	0	0	0
0	0	0	0	0	1	0	0	0	0
0	0	0	0	0	1	0	0	0	0
0	0	0	0	1	1	0	0	0	0
0	0	0	0	0	1	0	0	0	0

0	0	0	0	0	1	0	0	0	0
0	0	1	0	0	0	0	0	0	0
0	0	0	0	0	1	0	0	0	0
0	0	1	0	0	1	0	0	0	0
0	0	0	0	0	0	0	0	0	0
0	0	0	0	0	0	0	0	0	0
0	0	0	0	0	0	0	0	0	0
0	0	0	0	0	0	0	0	0	0
0	0	0	0	0	0	0	0	0	0
0	0	1	0	1	1	0	0	1	0
0	0	0	0	0	1	0	0	0	0
0	0	0	0	0	0	0	0	0	0
0	0	0	1	0	1	0	0	0	0
0	0	0	0	0	0	0	0	0	0
0	0	0	0	1	1	0	0	0	0
0	0	0	0	0	1	0	0	0	0
0	0	0	0	0	0	0	0	0	0
0	0	0	0	0	0	0	0	0	0
0	0	0	0	0	0	0	0	0	0
0	0	0	0	0	0	0	0	0	0
0	0	0	0	0	1	0	0	0	0
0	0	1	1	0	0	0	1	1	0
0	0	0	0	0	0	0	0	0	0
0	0	1	1	0	0	0	0	0	0
0	0	0	0	1	1	0	0	0	0
0	0	0	0	0	0	0	0	0	0
0	0	0	0	0	0	0	0	0	0
0	0	0	0	0	1	0	0	0	0
0	0	0	0	0	0	0	0	0	0
0	0	0	0	0	0	0	0	0	0
0	0	0	0	0	0	0	0	0	0
0	0	0	0	0	0	0	0	0	0
0	0	0	0	0	0	0	0	0	0
0	0	0	0	0	1	0	0	0	0
0	0	0	0	0	0	0	0	0	0
0	0	0	0	0	0	0	0	0	0
0	0	0	0	0	0	0	0	0	0
0	0	0	0	0	0	0	0	0	0
0	0	0	0	0	0	0	0	1	0
0	0	0	0	0	0	0	0	0	0
0	0	0	0	0	0	0	0	0	0
0	0	0	0	0	0	0	0	0	0
0	0	0	0	0	0	0	0	0	0
0	0	0	0	0	0	0	0	0	0
0	0	0	0	0	0	0	0	0	0
0	0	0	0	0	0	0	0	0	0
0	0	0	0	0	0	0	0	0	0

0	0	0	0	0	0	0	0	0	0
1	0	0	0	0	0	0	0	0	0
0	0	0	0	0	0	0	0	0	0
0	0	0	0	0	0	0	0	0	0
0	0	0	0	0	0	0	0	0	0
0	0	0	0	0	0	0	0	0	0
0	0	0	0	0	0	0	0	0	0
0	0	0	0	0	0	0	1	0	0
0	0	0	0	0	0	0	0	0	0
0	0	0	0	0	0	0	0	0	0
0	0	0	0	0	1	0	0	0	0
0	0	0	0	0	0	0	0	0	0
0	0	0	0	0	0	0	0	0	0
0	0	0	0	0	0	0	0	1	0
0	0	0	0	0	0	0	0	0	0
0	0	0	0	0	0	0	0	0	0
0	0	0	1	1	1	0	1	1	0
0	0	0	0	0	0	0	0	0	0
0	0	0	0	0	0	0	0	0	0
0	0	0	0	0	0	0	0	0	0
0	0	0	1	1	0	1	0	0	0
0	0	0	0	0	0	0	0	0	0
0	0	0	0	0	1	0	0	0	0
0	0	0	0	0	0	0	0	0	0
0	0	0	0	0	0	0	0	0	0
0	0	0	0	0	0	0	0	0	0
0	0	0	0	0	0	0	0	0	0
0	0	0	0	0	0	0	0	0	0
0	0	0	0	0	0	0	0	0	0
0	0	0	0	0	0	0	0	0	0
0	0	0	0	0	0	0	1	0	0
0	0	0	0	0	0	0	0	0	0
0	0	0	0	0	0	0	0	0	0
0	0	0	0	0	0	0	0	0	0
0	0	0	0	0	0	0	0	0	0
0	0	0	0	0	0	0	0	0	0
0	0	0	0	0	0	0	0	0	0
0	0	0	0	0	0	0	0	0	0
0	0	0	0	0	0	0	0	0	0
0	0	0	0	0	0	0	0	0	0
0	0	0	0	0	0	0	0	0	0
1	1	1	1	1	1	1	1	1	1
0	0	0	0	0	1	0	0	0	0
0	0	0	0	0	1	0	1	0	0
0	0	0	0	0	0	0	0	0	0
0	0	0	0	0	0	0	0	0	0
0	0	0	0	0	0	0	0	0	0

0	0	0	0	0	0	0	0	0	0
0	0	1	0	0	0	0	1	0	0
0	0	0	0	0	0	0	0	0	0
0	0	0	0	0	0	0	0	0	0
0	0	0	0	1	0	0	0	0	0
0	0	1	0	1	1	0	0	0	0
0	0	0	0	1	1	0	0	0	0
0	0	0	0	0	0	0	0	0	0
0	0	1	1	1	0	0	0	0	0
0	0	0	0	0	0	0	0	0	0
0	0	0	0	0	0	0	1	0	0
0	0	0	0	0	0	0	1	0	0
0	0	1	1	0	0	0	0	0	0
0	0	1	1	0	0	0	0	0	0
0	0	0	0	0	0	0	0	0	0
0	0	0	0	0	1	0	0	0	0
0	0	0	0	1	0	0	0	0	0
0	0	0	0	0	0	0	0	1	0
0	0	0	0	1	1	0	0	0	0
0	0	0	0	0	0	0	1	0	0
0	0	0	0	0	0	0	0	0	0
0	0	0	0	1	1	0	0	0	0
0	0	0	0	0	0	0	0	0	0
0	0	1	0	1	1	0	0	0	0
0	0	0	0	0	0	0	0	0	0
0	0	0	0	0	0	0	0	0	0
0	0	0	0	0	0	0	0	0	0
0	0	0	0	0	0	0	0	0	0
0	0	0	1	0	0	0	0	0	0
0	0	0	1	0	0	0	0	0	0
0	0	0	0	0	1	0	0	0	0
0	0	0	0	0	1	0	0	0	0
0	0	0	1	0	1	0	0	0	0
0	0	0	0	0	0	0	0	0	0
0	0	0	0	0	0	0	0	1	0
0	0	1	0	0	1	1	0	0	0
0	0	0	0	0	1	0	1	0	0
0	0	0	0	0	1	0	0	0	0
0	0	0	0	0	1	0	0	0	0
0	0	0	0	0	0	0	0	0	0
0	0	0	0	0	0	0	0	0	0
0	0	0	0	0	1	0	0	0	0
0	0	0	0	0	0	0	0	0	0
0	0	1	0	0	0	0	0	0	0
0	0	0	0	0	0	0	0	0	0
0	0	0	0	0	0	0	0	0	0
0	0	0	0	0	1	0	1	0	0

0	0	0	0	0	0	0	0	0	0
0	0	0	0	0	0	0	0	0	0
0	0	0	0	0	0	0	0	0	0
0	0	0	0	0	0	0	0	0	0
0	0	0	0	0	0	0	0	0	0
0	0	0	0	0	0	0	0	0	0
0	0	0	0	0	0	0	0	1	0
0	0	0	0	0	0	0	0	0	0
0	0	0	0	0	0	0	0	0	0
0	0	0	0	0	0	0	0	0	0
0	0	0	0	0	0	0	0	0	0
0	0	0	0	0	0	0	0	0	0
0	0	0	0	0	0	0	0	0	0
0	0	0	0	0	0	0	0	0	0
0	0	0	0	0	0	0	0	0	0
0	0	0	0	0	0	0	0	0	0
0	0	0	0	0	0	0	0	0	0
0	0	0	0	0	0	0	0	0	0
0	0	0	0	0	0	0	0	0	0
0	0	0	0	0	0	0	0	0	0
0	0	0	0	0	0	0	0	0	0
0	0	0	0	0	0	0	0	1	0
0	0	0	0	0	0	0	0	0	0
0	0	1	0	0	0	0	0	0	0
0	0	0	0	0	0	0	0	0	0
0	0	0	0	0	0	0	0	0	0
0	0	0	0	0	0	0	0	0	0
0	0	0	0	0	0	0	0	0	0
0	0	0	0	0	0	0	0	0	0
0	0	0	0	0	0	0	0	0	0
0	0	0	0	0	0	0	0	0	0
0	0	0	0	0	0	0	0	0	0
0	0	0	0	0	0	0	0	0	0
0	0	0	0	0	0	0	0	0	0
0	0	1	1	0	1	0	0	0	0
0	1	1	0	0	1	0	0	0	0
0	0	0	0	0	0	0	0	0	0
0	0	0	0	0	1	0	0	0	0
0	0	1	0	0	0	0	0	0	0
0	0	1	0	0	0	0	0	0	0
0	0	0	1	0	1	0	0	0	0
0	0	0	0	0	0	0	0	0	0
0	0	0	0	0	1	0	0	0	0
0	0	1	1	0	1	0	0	0	0
0	0	0	0	0	0	1	0	0	0
0	0	0	0	0	0	1	0	0	0
0	0	0	0	0	0	0	0	0	0

0	1	0	0	0	0	0	0	0	0
0	0	0	1	0	0	0	0	0	0
0	0	1	1	0	0	1	0	1	0
0	0	0	0	0	0	0	0	0	0
0	0	1	0	0	0	0	0	0	0
0	0	0	0	0	0	0	0	0	0
0	1	0	1	0	1	0	0	0	0
0	0	0	1	0	1	0	0	0	0
0	0	0	1	1	1	0	0	0	0
0	0	0	1	0	1	0	1	0	0
0	0	1	1	0	0	0	1	0	0
0	0	0	0	0	0	0	0	0	0
0	1	1	0	1	1	1	0	0	0
0	1	0	0	0	0	0	0	0	0
0	0	1	1	0	0	0	1	0	0
0	1	0	0	0	1	0	0	0	0
0	0	0	0	0	0	0	0	0	0
0	0	0	0	0	0	0	1	0	0
0	0	0	0	0	0	0	0	0	0
0	0	0	0	0	1	0	0	0	0
0	0	0	0	0	1	0	0	0	0
0	0	0	0	1	1	1	0	0	0
0	0	0	0	1	1	1	0	0	0
0	0	0	1	0	1	1	1	0	0
0	0	0	1	0	1	1	1	0	0
0	0	0	0	0	0	0	0	0	0
0	0	1	1	0	1	0	0	0	0
0	1	0	1	0	0	0	0	0	0
0	0	0	1	0	0	0	0	0	0
0	0	0	1	0	0	0	0	0	0
0	0	0	0	0	0	0	0	0	0
0	0	0	0	0	0	0	0	0	0
0	0	0	0	0	0	0	0	0	0
0	0	0	0	0	0	0	0	0	0
0	0	0	0	0	0	0	0	0	0
0	0	0	0	0	0	0	0	0	0
0	0	0	0	0	0	0	0	0	0
0	0	0	0	0	0	0	0	0	0
0	0	0	0	0	0	0	0	0	0
0	0	0	0	0	0	0	0	0	0
0	0	0	0	0	0	0	0	0	0
0	0	0	0	0	0	0	0	0	0
0	0	0	0	0	0	0	0	0	0
0	0	0	0	0	0	0	0	0	0
0	0	0	0	0	1	0	0	0	0
0	0	0	0	0	0	0	0	0	0
0	0	0	1	0	0	0	0	0	0

0	0	0	0	0	0	0	0	0	0
0	0	0	0	0	0	0	0	0	0
0	0	0	0	0	0	0	0	0	0
0	0	0	0	0	1	0	0	0	0
0	0	0	0	0	1	0	0	0	0
0	0	0	1	0	1	0	0	1	0
0	0	1	0	1	1	1	0	1	0
1	1	0	0	0	0	0	0	0	0
0	0	0	0	0	0	0	0	0	0
0	0	0	0	0	0	1	0	1	0
0	0	0	0	0	0	0	0	0	0
0	0	0	0	0	0	0	0	0	0
0	0	0	0	0	0	0	0	0	0
0	0	0	0	0	0	0	0	0	0
0	0	0	0	0	1	0	0	0	0
0	0	0	0	0	0	0	0	0	0
0	0	1	0	0	0	0	0	0	0
0	0	0	0	0	0	0	0	0	0
0	0	0	0	0	1	0	0	0	0
0	0	0	0	0	1	0	0	0	0
0	1	0	1	0	1	0	0	0	0
0	0	0	0	0	1	0	0	0	0
0	0	0	0	0	1	0	0	0	0
0	0	0	0	0	0	0	0	0	0
0	0	0	0	0	0	0	0	0	0
0	0	0	0	0	0	0	0	0	0
0	0	1	0	0	0	0	0	0	0
0	0	0	0	0	0	0	0	0	0
0	0	0	0	0	0	1	0	0	0
0	0	0	0	0	0	0	0	0	0
0	0	0	0	0	0	0	0	0	0
0	0	0	1	0	1	0	1	0	0
0	0	0	0	0	0	0	0	0	0
0	0	0	1	0	1	0	0	0	0
0	0	0	0	0	0	0	0	0	0
0	0	0	0	0	1	0	0	0	0
0	0	0	0	0	0	0	0	0	0
0	0	0	0	0	0	0	0	0	0
0	0	0	0	0	0	0	0	0	0
0	0	0	0	0	0	0	0	0	0
0	0	0	0	0	0	0	0	0	0
0	0	0	0	0	0	0	0	0	0
0	0	0	1	0	0	0	0	0	0
0	1	0	0	0	0	0	0	0	0
0	0	0	0	0	0	0	0	0	0
0	0	0	0	0	1	0	0	0	0
0	0	0	0	0	1	0	0	0	0

0	0	0	0	0	1	0	0	0	0
0	0	0	0	0	0	0	0	0	0
0	0	0	0	1	1	0	0	0	0
0	0	0	0	0	0	0	0	0	0
0	0	0	0	0	0	0	0	0	0
0	0	0	0	0	0	0	0	0	0
0	0	0	0	0	0	0	0	0	0
0	0	0	0	0	0	0	1	0	0
0	0	0	0	0	0	0	0	0	0
0	0	1	0	0	0	0	0	0	0
0	0	0	0	0	0	0	0	0	0
0	0	0	0	0	0	0	0	0	0
0	0	0	0	0	0	0	0	0	0
0	0	0	0	0	0	0	0	0	0
0	0	0	0	0	0	0	0	0	0
0	0	0	0	0	0	0	0	0	0
0	0	0	0	0	0	0	0	1	0
0	0	0	0	0	0	0	0	0	0
0	0	0	0	0	0	0	0	0	0
0	0	0	0	0	0	0	0	0	0
0	0	0	0	0	0	0	0	0	0
0	0	0	0	1	1	0	0	1	0
0	0	0	0	0	0	0	0	0	0
0	0	0	0	1	1	0	0	0	0
0	0	0	0	0	0	0	0	0	0
0	0	0	1	0	0	0	1	0	0
0	0	1	0	1	1	0	0	0	0
0	0	0	0	0	1	0	0	0	0
0	0	0	1	0	1	0	0	0	0
0	0	0	0	0	1	0	0	0	0
0	0	0	0	0	1	0	0	0	0
0	0	0	0	0	0	0	0	0	0
0	0	0	0	0	0	0	0	0	0
0	0	0	0	0	1	1	1	0	0
0	0	0	1	0	0	0	0	0	0
0	1	0	0	0	0	1	0	0	0
0	0	0	0	0	0	0	1	0	0
0	0	0	0	0	0	0	0	0	0
0	0	0	0	0	0	1	0	1	0
0	1	0	0	0	0	0	0	0	0
0	0	0	0	0	1	0	0	0	0
0	0	0	0	1	1	0	1	0	0
0	0	0	0	0	0	0	0	0	0
0	0	0	0	0	0	0	0	0	0
0	0	0	0	0	0	0	0	0	0
0	0	1	1	0	0	0	0	0	0
0	0	0	0	0	0	0	0	1	0

0		0	0	0	1	0	0	0	0
0	0	0	0	0	0	0	0	0	0
0		0	0	0	0	0	0	0	0
0	0	0	0	0	0	0	0	0	0
0	0	0	0	0	0	0	0	0	0
0	0	0	0	0	1	0	0	0	0
0		0	0	1	1	0	0	0	0
0	0	0	0	0	0	0	0	0	0
0	0	0	0	1	1	1	0	1	0
0	1	0	0	0	0	0	0	1	0
0	1	0	0	0	0	0	0	0	0
0	0	0	0	1	0	0	0	0	0
0	0	0	0	0	0	0	0	0	0
0		0	0	0	0	0	0	0	0
0	0	0	0	0	0	0	0	0	0
0		0	0	0	1	0	0	0	0
0	0	1	0	0	0	0	0	0	0
0	0	0	0	0	0	0	0	0	0
0	0	0	0	0	0	0	0	0	0
0	0	1	0	0	0	0	0	0	0
0		0	1	0	0	0	0	0	0
0		0	0	1	1	0	0	0	0
0	0	0	0	0	0	0	0	0	0
0	0	0	0	0	0	0	0	0	0
0	0	0	0	0	0	0	0	0	0
0	0	0	0	0	0	0	0	0	0
0		0	0	0	0	0	0	0	0
0	0	0	0	0	0	0	0	1	0
0	0	0	0	1	0	0	0	0	0
0	0	0	0	0	0	0	0	0	0
0	0	0	0	0	0	0	0	0	0
0	0	0	0	0	0	0	0	0	0
0		0	0	0	0	0	0	0	0
0	1	0	0	0	0	0	0	0	0
0	1	0	0	0	0	0	0	0	0
0	1	0	0	1	1	0	0	0	0
0	0	0	0	0	0	0	0	0	0
0	0	0	0	0	0	0	0	0	0
0		0	0	0	0	0	0	0	0
0	0	1	0	0	1	0	0	0	0
0		1	0	0	0	0	0	0	0
0		0	0	0	0	0	0	0	0
0	0	0	0	0	1	0	0	0	0
0	0	0	0	0	0	0	0	0	0
0	1	0	0	0	1	1	0	0	0
0	0	0	0	0	0	0	0	0	0
0	0	0	0	0	1	0	0	0	0

0	1	1	1	1	1	0	0	0	0
0	0	0	0	0	0	0	0	0	0
0	0	0	0	0	0	0	0	0	0
0	0	0	0	0	0	0	0	0	0
0	0	0	0	0	0	0	0	0	0
0	1	0	0	0	0	0	0	0	0
0	1	0	0	0	0	0	0	0	0
0		0	0	0	0	0	0	0	0
0		0	0	0	0	0	0	0	0
0	0	0	0	0	0	0	0	0	0
0		0	0	1	0	0	0	0	0
0		0	0	0	1	1	0	0	0
0	0	0	0	0	0	0	0	0	0
0	0	0	0	0	0	0	0	0	0
0	0	0	0	0	1	0	0	0	0
0	0	0	0	0	0	0	0	0	0
0		0	0	0	0	0	0	0	0
0		0	1	0	0	0	1		0
0	0	0	0	1	1	1	1	0	0
0	0	0	0	0	0	0	0	0	0
0	0	0	0	0	0	0	0	0	0
0	0	0	0	0	0	0	0	0	0
0	0	0	0	0	0	0	0	0	0
0	0	1	0	1	1	0	0	0	0
0	0	0	1	1	1	0	0	0	0
0	0	0	1	1	1	1	1	0	0
0	1	0	1	0	1	0	0	0	0
0	0	0	0	0	0	0	0	1	0
0	0	0	1	0	0	0	0	0	0
0	0	0	0	0	0	0	0	0	0
0	0	0	0	0	0	0	0	0	0
0	0	0	0	0	0	0	0	0	0
0	0	0	0	0	0	0	0	0	0
0	0	0	0	0	0	0	0	0	0
0	0	0	0	0	0	0	0	0	0
0	0	0	0	0	0	0	0	0	0
0	0	0	0	0	0	0	0	0	0
0	0	0	0	0	0	0	0	0	0
0	0	0	0	0	0	0	0	0	0
0	0	0	0	0	0	0	0	0	0
0	0	0	0	0	0	0	0	0	0
0	0	0	0	1	1	0	0	1	0
0	0	1	0	1	1	0	0	0	0
0	0	0	0	0	0	0	0	0	0
0	0	0	0	1	1	0	0	0	0
0	0	0	0	0	0	0	0	0	0
0	0	1	0	0	1	0	0	0	0

0	0	0	1	1	1	0	0	0	0
0	0	0	0	0	0	0	0	0	0
0	0	0	0	1	1	0	0	0	0
0	0	1	1	0	1	0	0	0	0
0	0	0	0	0	0	0	0	0	0
0	0	0	0	1	1	0	0	0	0
0	0	0	0	0	0	0	0	0	0
0	0	0	0	0	0	0	0	0	0
0	0	0	0	0	0	0	0	0	0
0	0	0	0	0	0	0	0	0	0
0	0	0	0	1	1	1	0	0	0
0	0	0	0	0	1	0	0	0	0
0	0	0	1	0	0	0	0	0	0
0	0	0	0	0	1	0	0	0	0
0	0	0	0	0	0	0	0	0	0
0	0	0	0	0	0	0	0	0	0
0	0	0	1	0	1	1	0	0	0
0	0	0	0	0	0	0	0	0	0
0	0	0	0	1	1	0	1	0	0
0	0	1	0	0	0	0	0	0	0
0	0	0	0	0	0	0	0	0	0
0	0	0	0	0	1	0	0	0	0
0	0	0	0	0	0	0	0	0	0
0	0	1	1	0	0	0	0	0	0
0	0	0	0	0	0	0	0	0	0
0	0	0	0	0	1	0	0	0	0
0	0	1	1	0	1	0	0	0	0
0	0	0	0	0	1	0	0	0	0
0	0	0	0	0	0	0	0	0	0
0	0	0	0	0	0	0	0	0	0
0	0	0	0	1	1	0	0	0	0
0	0	0	0	0	0	0	0	0	0
0	0	0	0	0	0	0	0	0	0
0	0	0	1	0	1	0	0	1	0
0	0	0	0	0	0	0	0	0	0
0	0	0	0	0	1	0	0	0	0
0	0	0	0	0	1	0	0	0	0
0	0	0	1	0	0	0	1	0	0
0	0	0	0	0	0	0	0	0	0
0	1	1	0	0	0	0	0	0	0
0	0	0	0	0	0	0	0	0	0
0	0	1	1		1	0	0	1	0
0	0	1	0	0	1	0	0	0	0
0	0	0	0	0	0	0	0	0	0
0	0	0	0	0	0	0	0	0	0
0	0	0	0	0	0	0	0	0	0
0	0	0	0	0	0	0	0	0	0

0	0	0	0	0	0	0	0	0	0
0	0	0	0	0	0	0	0	0	0
0	0	0	0	0	0	0	0	1	0
0	0	0	0	0	0	0	0	0	0
0	0	0	0	0	0	0	0	0	0
0	0	0	0	0	0	0	0	0	0
0	0	0	0	0	0	0	0	0	0
0	0	0	0	0	0	0	0	0	0
0	0	0	0	0	0	0	0	0	0
0	0	0	0	0	0	0	0	0	0
0	0	0	0	0	0	0	0	0	0
0	0	0	0	0	1	0	0	0	0
1	0	0	0	0	0	0	0	0	0
0	0	0	0	0	0	0	0	0	0
0	0	0	0	0	0	0	0	0	0
0	0	0	0	0	0	0	0	0	0
0	0	0	0	0	0	0	0	0	0
0	0	0	0	0	0	0	0	0	0
0	0	0	0	0	0	0	0	0	0
0	0	0	0	0	0	0	0	0	0
0	0	0	0	0	0	0	0	0	0
0	0	0	0	0	0	0	0	0	0
1	0	0	0	0	0	0	0	0	0
0	0	0	0	0	0	0	0	1	0
0	0	0	0	0	0	0	0	0	0
0	0	0	0	0	0	0	0	0	0
0	0	0	0	0	0	0	0	0	0
0	0	0	1	0	0	0	0	1	0
0	0	1	1	1	0	0	0	0	0
0	0	0	0	0	0	0	0	0	0
0	0	0	0	0	0	0	0	0	0
0	0	0	0	0	0	0	0	1	0
0	0	0	0	0	0	0	0	0	0
0	0	0	0	0	0	0	0	0	0
0	0	0	0	0	0	0	0	0	0
0	0	0	0	0	0	0	0	0	0
0	0	0	0	0	0	0	0	0	0
0	0	0	0	0	0	0	0	0	0
0	0	0	0	0	0	0	0	0	0
0	0	0	0	0	0	0	0	0	0
0	0	0	1	1	1	0	0	0	0
0	0	1	0	0	0	0	1	0	0
0	0	0	0	0	0	0	0	0	0
0	0	0	0	0	1	0	0	0	0
0	0	0	0	0	0	0	0	0	0
0	0	0	0	0	0	0	0	0	0
0	0	0	0	0	0	0	1	1	0

0	0	0	0	0	0	0	1	1	0
0	0	0	1	0	0	0	0	0	0
0	0	0	1	0	0	0	0	0	0
0	0	0	0	0	0	0	0	0	0
0	0	0	0	0	0	0	0	0	0
0	0	0	0	0	1	0	0	0	0
0	0	0	0	0	0	0	0	0	0
0	0	0	0	0	0	0	0	0	0
0	0	0	0	0	1	0	0	0	0
0	0	0	0	0	0	0	0	0	0
0	0	0	0	0	0	0	0	0	0
0	0	0	0	0	0	0	0	0	0
0	0	0	0	0	1	0	0	0	0
0	0	1	1	1	1	0	0	0	0
0	0	0	0	0	0	0	0	0	0
0	0	0	0	0	0	0	0	0	0
0	1	0	0	1	1	1	0	0	0
0	0	0	0	0	0	0	0	0	0
0	0	0	0	0	0	0	0	0	0
0	0	1	1	1	0	1	0	0	0
0	0	0	1	1	0	0	0	0	0
0	0	0	1	0	1	0	0	0	0
0	0	0	0	0	0	0	0	0	0
0	0	0	0	0	0	0	0	0	0
0	0	0	0	0	0	0	0	0	0
0	0	0	0	0	0	0	0	0	0
0	0	0	0	0	0	0	0	0	0
0	0	0	0	0	0	0	0	0	0
0	0	0	0	0	0	0	0	0	0
0	0	0	0	0	0	0	0	0	0
0	0	0	0	0	0	0	0	0	0
0	0	0	0	0	1	0	0	0	0
0	0	0	0	0	0	0	0	0	0
0	0	0	0	0	0	0	0	0	0
0	1	0	0	0	1	0	0	0	0
0	0	0	0	0	0	0	0	0	0
0	0	0	0	0	0	0	0	0	0
0	0	0	0	0	0	0	0	0	0
0	0	0	0	0	1	0	0	0	0
0	0	0	0	0	0	0	0	0	0
0	0	0	0	0	0	0	0	0	0
0	0	0	0	0	0	0	0	0	0
0	0	0	0	0	0	0	0	0	0
0	0	0	0	0	0	0	0	0	0
0	0	0	0	0	0	0	0	0	0
0	0	0	0	0	0	0	0	0	0
0	0	0	0	0	0	0	0	0	0

0	0	0	0	0	0	0	0	0	0
0	0	0	0	0	0	0	0	0	0
0	0	0	0	0	0	0	0	0	0
0	0	0	0	0	0	0	0	0	0
0	0	0	0	0	0	0	0	0	0
0	0	0	0	0	1	0	0	0	0
1	0	1	1	0	1	0	1	0	0
0	0	0	0	0	0	0	0	0	0
0	0	0	0	0	0	0	0	0	0
0	0	0	0	0	0	0	0	0	0
0	0	0	0	0	0	0	0	0	0
0	0	1	1	0	1	0	0	0	0
0	0	0	0	0	0	0	0	0	0
0	0	0	0	0	0	0	0	0	0
0	0	0	0	0	0	0	0	0	0
0	0	0	0	0	0	0	0	0	0
0	0	0	0	0	0	0	0	0	0
0	0	0	0	0	0	0	0	0	0
0	0	0	1	0	1	0	0	0	0
0	0	0	0	0	0	0	0	0	0
0	0	0	0	0	0	0	0	0	0
0	0	0	0	0	0	0	0	0	0
0	0	0	0	0	0	0	0	0	0
0	1	1	1	0	1	0	0	0	0
0	0	0	1	1	0	0	1	0	0
0	0	0	0	0	1	0	0	0	0
1	0	0	0	0	1	0	0	0	0
0	0	0	0	0	0	1	1	0	0
0	0	0	0	0	0	0	1	0	0
0	0	0	0	0	0	0	0	0	0
0	1	0	0	0	1	0	0	0	0
0	0	0	0	0	0	0	0	0	0
0	0	0	0	0	0	0	0	0	0
0	0	0	0	0	0	0	0	0	0
0	0	0	0	0	0	0	0	0	0
0	1	0	0	1	0	0	0	0	0
0	1	0	0	1	0	0	0	0	0
0	0	0	1	0	0	0	0	0	0
0	1	0	0	1	1	0	0	0	0
0	0	0	0	0	0	0	0	0	0
0	0	1	0	0	0	0	0	0	0
0	0	0	0	0	1	0	1	0	0
0	0	0	0	0	0	0	0	0	0
0	0	0	0	0	0	0	0	0	0
0	0	0	0	1	0	0	0	0	0
0	0	0	0	0	0	0	0	0	0
0	0	0	0	0	0	0	0	0	0

0	0	0	0	0	0	0	0	0	0
0	0	1	0	0	0	0	1	0	0
0	0	1	1	0	0	0	0	0	0
0	0	1	0	0	1	0	0	0	0
0	0	0	0	0	1	0	0	0	0
0	0	0	0	0	0	0	0	0	0
0	0	0	0	0	0	0	0	0	0
0	0	0	0	0	0	0	0	0	0
0	0	0	0	0	1	0	1	0	0
0	0	0	0	0	0	0	0	0	0
0	0	0	0	0	1	0	0	0	0
0	0	0	0	0	0	0	0	0	0
0	0	0	0	0	0	1	0	0	0
0	0	1	0	0	1	0	0	0	0
0	0	0	0	0	0	0	0	0	0
0	0	0	0	0	0	0	0	0	0
0	0	0	0	0	1	0	0	0	0
0	0	0	0	0	1	0	0	0	0
0	0	0	0	0	0	0	0	0	0
0	0	0	0	0	1	0	0	0	0
0	0	0	0	0	0	0	0	0	0
0	0	0	0	1	0	0	0	0	0
0	0	0	0	0	1	0	0	0	0
0	1	1	1	0	0	0	1	0	0
0	0	0	0	0	0	0	0	0	0
0	0	0	0	0	1	0	0	0	0
0	0	0	0	0	0	0	0	0	0
0	0	0	0	0	1	0	0	0	0
0	0	0	0	0	0	0	0	0	0
0	0	1	0	0	0	0	0	0	0
0	1	0	0	0	0	0	0	0	0
0	1	0	1	0	1	0	1	0	0
0	0	0	0	0	0	0	0	0	0
0	0	0	0	0	0	0	0	0	0
0	0	0	0	0	0	0	0	0	0
0	0	0	0	0	0	0	0	0	0
0	0	0	1	0	1	0	0	0	0
0	0	0	0	0	0	0	0	0	0
0	0	0	0	0	1	0	0	0	0
0	0	0	0	0	0	0	0	0	0
0	0	0	1	0	0	0	0	0	0
0	0	0	0	0	0	0	0	0	0
0	1	0	0	0	0	0	0	0	0
0	0	0	0	0	1	0	0	0	0
0	0	0	0	0	0	0	0	0	0
0	1	0	0	0	0	0	0	0	0
0	0	0	0	0	1	0	0	0	0

0	0	0	0	0	0	0	0	0	0
1	0	0	0	0	0	0	0	0	0
0	0	0	0	0	0	0	0	0	0
0	0	0	0	0	0	0	0	0	0
0	0	0	0	0	0	0	0	0	0
0	0	1	1	0	1	0	0	1	0
0	0	0	0	0	0	0	0	0	0
0	0	0	0	0	0	0	0	0	0
0	0	0	0	0	0	0	0	0	0
0	0	0	0	0	0	0	0	0	0
0	0	0	0	0	0	0	0	0	0
0	0	1	1	0	1	0	0	1	0
0	0	0	0	0	0	0	0	0	0
0	0	0	0	0	0	0	0	0	0
0	0	1	0	0	1	0	0	1	0
0	0	0	0	0	0	0	0	0	0
0	0	0	0	0	0	0	0	0	0
0	0	0	0	0	0	0	0	0	0
0	0	0	0	0	0	0	0	0	0
0	0	0	0	0	0	0	0	0	0
0	0	0	0	0	0	0	0	0	0
0	0	0	0	0	0	0	0	0	0
0	0	1	1	0	0	0	0	0	0
0	0	0	1	0	0	0	1	0	0
0	0	0	0	0	0	0	0	0	0
0	0	0	0	0	0	0	0	0	0
1	0	0	0	1	1	1	1	1	0
1	0	0	0	0	0	0	0	0	0
1	0	0	1	0	1	0	1	0	0
0	0	0	0	0	0	0	0	0	0
0	0	0	0	0	1	0	0	0	0
0	0	0	0	0	0	0	0	0	0
0	0	0	1	0	0	1	1	0	0
0	1	1	1	1	1	1	1	1	0
0	0	0	0	0	1	0	1	0	0
0	0	1	0	0	1	0	0	1	0
0	0	0	0	0	0	0	0	0	0
0	0	0	0	0	0	0	0	0	0
0	0	0	0	0	0	0	0	0	0
0	0	0	0	0	0	0	0	0	0
0	0	0	0	0	0	1	0	0	0
0	0	0	0	0	0	0	0	0	0
0		0	1	1	0	0	0	0	0
0	0	1	0	0	0	0	0	0	0
0		0	0	0	0	0	0	0	0
0		0	0	0	0	0	1	0	0
0	0	0	0	0	1	1	0	0	0

0	0	0	1	1	0	0	0	0	0
0	0	0	0	0	1	0	0	0	0
0	0	0	0	1	1	0	0	0	0
0	1	0	0	0	0	0	0	0	0
0	1	0	1	1	0	0	1	0	0
0		0	0	1	1	0	0	0	0
0	0	1	0	0	0	0	0	0	0
0	0	0	0	0	1	0	0	0	0
0	0	0	0	0	0	0	0	0	0
0	0	1	0	0	1	1	1	1	1
0	0	0	0	0	0	0	0	0	0
0	0	0	0	0	0	0	0	0	0
0	0	0	0	0	0	0	0	0	0
0	0	0	0	0	0	0	0	0	0
0	0	0	0	1	1	0	0	0	0
0	0	0	0	0	0	0	0	0	0
0	0	0	0	0	0	0	0	0	0
0	0	0	0	0	0	0	0	0	0
0		0	0	0	0	0	0	0	0
0	0	0	0	0	0	0	0	0	0
0	1	0	0	0	0	0	0	0	0
0	0	0	1	0	0	0	0	0	0
0	1	1	0	0	0	0	0	0	0
0	0	0	0	0	1	0	0	0	0
0		0	0	0	0	0	0	0	0
0		0	0	0	1	0	1	0	0
0		0	0	0	1	0	0	0	0
0		0	0	0	0	0	0	0	0
0	0	0	0	0	0	0	0	0	0
0		0	0	0	1	0	0		
0	0	0	0	0	0	0	0	0	0
0	0	0	1	0	0	0	0	0	0
0	0	0	1	0	0	0	0	0	0
0	0	0	0	0	0	0	0	0	0
0	0	0	0	0	0	0	0	0	0
0	0	0	1	0	0	0	0	0	0
0	0	0	0	0	0	0	0	0	0
0	0	0	0	0	0	0	0	0	0
0	0	0	0	0	0	0	0	0	0
0	0	0	0	0	0	0	0	0	0
0	0	0	0	0	0	0	0	0	0
0	0	0	0	1	1	1	0	0	0
0	0	0	0	0	0	0	0	0	0
0	0	1	0	0	0	0	0	0	0
0	0	0	0	0	1	0	0	0	0
1	1	1	1	1	1	1	1	1	1
0	0	0	0	0	0	0	0	0	0

0	1	0	0	1	0	0	0	0	0
0	0	0	0	0	0	0	0	0	0
0	0	0	1	0	0	0	0	0	0
0	0	0	1	0	0	0	0	0	0
0	0	0	0	0	1	0	0	0	0
0	1	1	1	0	1	0	0	0	0
0	0	0	0	0	0	0	0	0	0
0	0	0	0	0	0	0	0	0	0
0	0	1	0	1	1	0	0	1	0
1	1	1	1	1	1	0	0	1	0
0	0	0	0	0	0	0	0	0	0
0	1	0	0	0	0	0	0	0	0
0	0	1	0	0	1	0	1	0	0
0	0	0	0	0	1	0	1	0	0
0	0	1	0	0	1	0	0	0	0
0	0	0	0	0	1	0	0	0	0
0	0	0	0	0	0	0	0	0	0
0	0	1	0	0	0	0	1	0	0
0	0	1	0	0	0	0	1	0	0
0	0	1	0	0	1	0	0	0	0
0	0	0	0	0	0	0	0	0	0
0	0	1	0	0	1	0	0	0	0
0	0	0	0	0	1	0	0	0	0
0	0	0	0	0	0	0	0	0	0
0	0	0	0	0	0	0	0	0	0
0	0	0	0	0	0	0	0	0	0
1	1	1	1	0	1	0	1	0	0
0	0	0	0	0	0	0	0	0	0
1	1	0	0	0	0	0	0	0	1
0	0	0	0	0	0	0	0	0	0
0	0	1	0	0	1	1	1	1	0
0	1	0	0	1	0	0	0	0	1
0	0	0	0	0	0	0	0	0	0
0	0	0	0	0	0	0	0	0	0
0	0	0	0	0	0	0	0	0	0
0	0	0	0	0	1	0	0	0	0
0	0	0	0	0	0	0	1	0	0
0	0	0	0	0	1	0	0	0	0
0	0	0	0	0	1	0	0	0	0
0	0	0	1	0	0	0	0	0	0
0	0	0	0	0	0	0	0	0	0
0	0	0	0	0	0	0	0	0	0
0	0	0	0	0	0	0	0	0	0
0	0	0	0	0	0	0	0	1	0
0	0	0	0	0	0	0	1	0	0
0	0	0	0	0	0	0	0	0	0
0	0	0	0	0	0	0	0	0	0

0	0	0	0	0	0	0	0	0	0
0	0	0	0	0	0	0	0	0	0
0	0	0	0	0	0	0	0	0	0
0	0	0	0	0	0	0	0	0	0
0	0	0	0	1	1	0	0	0	0
0	0	0	0	0	0	0	0	0	0
0	0	0	0	0	1	0	0	0	0
0	0	0	0	0	1	0	0	0	0
0	0	0	0	0	1	0	0	0	0
0	0	1	1	1	1	0	0	0	0
0	0	0	0	0	0	0	0	0	0
0	0	0	0	0	1	0	0	0	0
0	0	0	1	1	1	0	0	0	0
0	0	0	0	0	1	0	0	0	0
0	0	0	0	0	0	0	0	0	0
0	0	0	0	0	0	0	0	0	0
0	0	0	0	0	0	0	0	0	0
0	0	0	0	0	0	0	0	0	0
0	0	0	0	0	0	0	0	0	0
0	1	0	0	0	0	0	0	0	0
0	0	0	0	0	0	0	0	0	0
0	0	0	0	0	0	0	0	0	0
0	0	0	0	0	0	0	0	0	0
0	0	0	0	0	0	0	0	0	0
0	0	0	0	0	0	0	0	0	0
0	0	0	0	0	0	0	0	0	0
0	0	1	0	0	0	0	0	0	0
0	0	0	0	0	0	0	0	0	0
0	1	0	0	0	0	0	0	0	0
0	0	0	0	0	0	0	1	0	0
0	0	0	0	0	0	0	0	0	0
0	1	0	0	0	0	0	0	0	0
0	0	0	0	0	0	0	0	0	0
0	0	0	0	0	0	0	0	0	0
0	0	0	0	0	0	0	0	0	0
0	0	0	0	0	0	0	0	0	0
0	0	0	0	0	0	0	0	0	0
0	0	0	0	0	0	0	0	0	0
0	0	0	0	0	0	0	0	0	0
0	0	0	0	0	0	0	0	0	0
0	0	0	0	0	0	0	0	0	0
0	0	0	0	0	0	0	0	0	0
0	0	0	0	0	1	0	0	0	0
0	1	1	1	0	1	0	1	0	0
0	0	0	1	0	0	0	0	0	0
0	0	0	0	0	0	0	0	0	0
0	1	0	0	0	1	0	0	0	0

0	1	0	1	0	1	0	0	0	0
0	1	1	0	0	1	0	0	0	0
0	1	1	1	0	0	0	1	0	0
0	0	0	1	0	0	0	1	0	0
0	0	0	0	0	1	0	1	0	0
0	1	0	0	0	0	0	1	0	0
0	0	0	0	0	0	0	0	0	0
0	0	0	0	0	1	0	0	0	0
0	0	0	0	0	0	0	0	0	0
0	0	0	0	0	0	0	0	0	0
0	0	0	0	0	0	0	0	0	0
0	0	0	1	0	1	0	0	0	0
0	0	0	0	0	0	0	1	0	0
0	0	0	0	0	0	0	0	0	0
0	0	0	0	0	0	0	0	0	0
0	0	0	0	0	0	0	0	0	0
0	0	0	0	0	0	0	0	0	0
0	1	0	0	0	0	0	0	0	0
0	0	0	0	0	0	0	0	0	0
0	0	0	0	0	0	0	0	0	0
0	1	0	1	0	0	0	0	0	0
0	1	0	0	0	0	0	1	0	0
0	0	0	1	0	0	0	0	0	0
0	0	0	0	0	0	0	0	0	0
0	0	0	0	0	1	0	0	0	0
0	0	0	0	0	1	0	0	0	0
0	0	0	0	0	1	0	0	0	0
0	0	0	0	0	1	0	0	0	0
0	0	0	0	0	0	0	0	0	0
0	0	0	0	0	1	0	0	0	0
0	0	1	0	0	0	0	0	0	0
0	0	0	0	0	0	0	0	0	0
0	0	0	0	0	0	0	0	0	0
0	0	0	0	0	1	0	0	0	0
0	0	0	0	0	0	0	0	0	0
0	0	0	0	0	0	0	0	0	0
0	0	0	0	0	1	0	0	0	0
0	0	0	0	0	0	0	0	0	0
0	0	0	0	0	1	0	0	0	0
0	0	1	1	0	0	0	0	0	0
0	0	0	0	0	0	0	0	0	0
0	0	0	0	0	1	0	1	0	0
0	0	0	0	0	1	0	0	0	0
0	0	0	0	0	0	0	0	0	0
0	0	0	0	0	0	0	0	0	0
0	1	0	0	0	0	0	0	0	0
1	0	0	1	0	0	0	0	0	0

0	0	0	0	0	1	0	0	0	0
0	1	0	0	0	0	0	0	0	0
0	0	1	0	0	0	0	0	0	0
0	0	0	1	0	0	0	0	0	0
0	1	0	0	0	0	0	1	0	0
0	0	0	0	0	0	0	0	0	0
0	0	0	0	0	0	0	0	0	0
0	0	1	1	0	0	0	0	1	0
0	0	0	1	0	0	0	0	0	0
0	0	0	0	0	0	0	0	0	0
0	0	0	0	0	0	0	0	0	0
0	0	0	0	0	0	0	0	0	0
0	0	0	1	0	0	0	0	0	0
0	0	0	0	0	0	0	0	0	0
0	0	0	0	0	0	0	0	0	0
0	0	0	0	0	0	0	0	0	0
0	0	0	1	0	0	0	1	1	0
0	0	0	1	0	0	0	0	0	0
0	0	0	0	0	0	0	0	0	0
0	0	0	1	0	0	0	0	1	0
0	0	0	0	0	1	0	0	0	0
0	1	0	1	0	0	0	1	0	0
0	0	0	1	0	0	0	0	0	0
0	0	1	0	0	0	0	1	0	0
0	0	0	0	0	0	0	0	0	0
0	0	0	0	0	0	0	0	0	0
0	0	0	0	0	1	0	0	0	0
0	1	0	0	1	0	0	1	0	0
0	1	0	0	1	1	1	1	0	0
0	0	1	1	0	1	1	1	0	0
0	1	0	1	0	0	0	0	0	0
0	0	0	0	0	0	0	0	0	0
0	0	0	0	0	0	0	0	0	0
0	0	1	1	1	1	1	1	0	0
0	0	0	0	0	0	0	0	0	0
0	0	0	0	0	0	0	0	0	0
0	0	0	0	0	0	0	0	0	0
0	0	0	0	0	0	0	0	0	0
0	1	0	0	0	1	0	0	0	0
0	1	0	1	0	1	0	0	0	0
0	1	0	0	1	1	0	0	0	0
0	1	0	0	1	1	1	0	0	0
0		0	0	0	0	0	0	0	0
0	0	1	0	0	0	0	0	0	0
0	0	0	0	0	0	0	0	0	0
0	1	1	0	1	1	0	0	1	0
0	1	1	1	1	0	0	0	1	0

0	1	0	0	0	0	0	0	0	0
0	1	0	0	0	1	0	1	0	1
0	1	0	1	0	0	0	0	0	0
0	0	0	1	0	0	0	0	0	0
0	1	0	0	0	0	0	0	0	0
0	0	0	0	0	0	0	0	0	0
0		0	0	0	1	0	0	0	0
0	0	0	0	0	0	0	0	0	0
0	0	0	0	0	0	0	0	0	0
0	0	0	0	0	0	0	0	0	0
0	1	0	0	0	0	0	0	0	0
0		0	0	1	0	0	0	0	0
0	1	0	1	0	0	0	1	0	0
0	0	0	0	0	0	0	0	0	0
0	0	0	0	0	0	0	0	0	0
0	0	0	1	0	1	1	0	0	0
0	0	0	1	0	0	0	0	0	0
0	1	1	0	0	0	0	0	0	0
0	1	0	0	0	1	0	0	0	0
0	0	0	0	0	0	0	0	0	0
0	0	0	0	0	0	0	0	0	0
0	0	0	0	0	0	0	0	0	0
0	0	0	0	0		0	0	0	0
0	0	0	0	0	0	0	0	0	0
0	0	1	0	0	0	0	0	0	0
0	1	0	1	0	0	0	1	0	0
0	0	0	0	0	0	0	0	0	0
0	0	0	1	0	0	0	0	0	0
0	0	0	1	0	0	0	0	0	0
0	0	0	0	0	0	0	0	0	0
0	0	0	0	0	0	0	0	0	0
0	0	0	0	0	0	0	0	0	0
0	0	0	0	0	0	0	0	0	0
0	1	1	0	1	1	0	0	0	0
0	0	0	0	0	0	0	0	0	0
0	1	0	0	1	0	1	0	0	0
0	0	0	0	0	0	0	0	0	0
0	0	0	0	0	0	0	0	0	0
0	0	0	0	0	0	0	0	0	0
0	0	1	1	0	1	0	0	0	0
1	0	1	0	1	1	0	0	0	0
0	0	0	0	0	0	0	0	0	0
0	0	1	0	0	0	0	1	0	0
0	0	0	0	0	1	0	0	0	0
0	0	0	0	0	0	0	0	0	0
0	0	0	0	0	0	0	0	0	0
0	0	0	0	0	0	1	0	0	0

0	0	0	0	0	0	0	0	1	0
0	0	0	0	0	0	0	0	0	0
0	0	0	0	0	0	0	0	0	0
0	0	0	0	0	0	0	0	0	0
1	0	1	0	0	0	0	0	0	0
0	0	0	0	0	1	0	0	0	0
0	0	0	0	0	0	0	0	0	0
0	1	1	1	1	1	1	0	0	0
0	0	0	0	0	0	0	0	0	0
0	0	0	0	0	0	0	0	0	0
0	0	0	0	0	0	0	0	0	0
0	0	0	0	0	0	0	0	0	0
0	0	0	0	0	0	0	0	0	0
0	0	0	0	0	0	0	0	0	0
0	0	0	0	1	0	0	0	0	0
0	0	0	0	0	0	0	0	0	0
0	0	0	0	0	0	0	0	0	0
0	0	0	0	0	0	0	1	0	0
0	0	0	0	0	0	0	0	0	0
0	0	0	0	0	0	0	0	0	0
0	0	0	0	0	0	1	0	0	0
0	0	0	0	0	0	0	0	0	0
0	0	0	0	0	0	0	0	0	0
0	0	0	0	0	0	0	0	0	0
0	0	0	0	0	1	0	0	0	0
0	0	0	0	1	1	0	0	0	0
0	0	1	0	0	0	0	1	0	1
0	0	0	0	0	0	0	0	0	0
0	0	0	0	0	0	0	0	0	0
0	0	0	0	0	0	0	0	0	0
0	0	0	0	0	0	0	0	0	0
0	0	1	0	1	1	0	0	0	0
0	0	1	1	0	0	0	1	0	0
0	0	1	1	0	1	0	0	0	0
0	0	0	0	1	0	1	0	1	0
0	1	0	1	1	1	0	0	0	0
0	1	0	0	0	0	0	0	0	0
0	0	1	0	0	0	0	0	0	0
0	1	0	0	0	0	0	0	0	0
0	0	0	0	0	0	0	0	0	0
0	0	0	0	0	0	0	0	0	0
0	0	0	1	0	0	0	0	0	0
0	0	0	0	0	0	0	0	0	0
0	0	0	0	0	0	0	0	0	0
0	0	0	0	0	0	0	0	0	0
0	0	0	0	0	0	0	0	0	0
0	0	0	0	0	0	0	0	0	0

0	0	0	0	0	0	0	0	0	0
0	0	0	0	0	0	0	0	0	0
0	0	0	0	0	0	0	0	0	0
0	0	0	0	0	0	0	0	0	0
0	0	0	0	0	0	0	0	0	0
0	1	0	0	0	0	0	0	0	0
0	0	0	0	0	0	0	0	0	0
0	0	0	0	0	0	0	0	0	0
0	0	0	0	0	0	0	0	0	0
0	1	0	0	0	0	0	0	0	0
0	1	0	0	0	0	0	0	0	0
0	1	0	0	0	0	0	0	0	0
0	1	0	0	0	0	0	0	0	0
0	1	0	0	0	1	0	0	0	0
0	1	1	0	0	1	0	0	0	0
0	0	0	0	0	0	0	0	0	0
0	0	0	0	0	0	0	0	0	0
0	0	0	0	0	0	0	0	0	0
0	0	0	0	0	1	0	0	0	0
0	1	1	1	0	1	0	0	0	0
0	1	0	1	0	0	0	0	0	0
0	1	0	0	0	0	0	0	0	0
0	0	1	1	0	1	0	0	0	0
0	0	0	0	0	0	0	0	0	0
0	0	0	0	0	0	0	0	0	0
0	0	1	0	0	0	0	0	0	0
0	0	1	0	0	1	0	0	0	0
0	1	0	0	1	1	1	0	0	0
0	1	0	1	1	1	0	1	0	0
0	0	0	0	0	0	0	0	0	0
0	0	0	0	0	0	0	0	0	0
0	0	0	0	0	1	0	0	0	0
0	0	0	0	0	1	0	0	0	0
0	1	0	0	0	0	0	0	0	0
0	0	1	1	0	1	0	0		0
0	0	0	0	0	0	0	0	0	0
0	0	0	0	0	0	0	0	0	0
0	1	0	0	0	0	0	0	0	0
0	0	0	0	0	0	0	0	0	0
0	0	0	0	0	0	0	0	0	0
0	0	0	0	0	1	0	0	0	0
0	0	0	0	0	0	0	0	0	0
0	0	0	0	0	0	0	1	0	0
0	0	0	0	0	0	0	0	0	0
0	0	0	0	0	0	0	0	0	0
0	1	0	0	0	1	0	0	0	0
0	0	1	1	0	0	0	0	0	0

0	0	0	0	0	0	1	0	0	0
0	0	0	1	0	0	0	0	0	0
0	0	0	0	0	0	0	0	0	0
0	1	0	0	0	0	0	0	0	0
0	0	0	0	0	0	0	0	0	0
0	0	0	0	0	1	0	1	0	0
0	0	0	0	0	1	0	0	0	0
0	0	0	0	0	0	0	0	0	0
0	0	0	0	0	1	0	1	0	0
0	0	0	0	0	0	0	0	0	0
0	0	1	1	0	0	0	0	0	0
0	0	0	0	0	0	0	0	0	0
0	0	0	0	0	0	0	0	0	0
0	0	0	0	0	0	0	0	0	0
0	0	0	0	0	0	0	0	0	0
0	0	0	0	0	0	0	0	0	0
0	0	0	0	0	0	0	0	0	0
1	0	0	1	0	0	0	0	0	0
0	0	0	0	0	0	0	0	0	0
0	1	0	1	0	1	0	0	0	0
0	0	0	0	0	0	0	0	0	0
0	0	0	0	0	0	0	0	0	0
0	1	0	0	0	1	0	0	0	0
0	1	0	0	0	1	0	1	1	0
1	0	0	0	0	1	0	1	0	0
0	1	0	0	0	1	0	1	1	0
0	1	0	0	0	0	0	1	0	0
1	0	0	0	0	0	0	0	0	0
0	0	0	0	0	1	0	1	0	0
1	1	0	0	1	1	0	0	0	0
0	0	0	1	0	0	0	0	0	0
0	1	1	1	0	0	0	1	1	0
1	1	0	0	1	0	1	0	0	0
0	0	0	1	0	0	0	1	0	0
0	0	0	0	1	1	0	0	0	0
0	1	0	1	0	1	0	0	0	0
0	0	0	1	1	1	1	1	0	0
0	0	0	0	0	0	0	0	0	0
1	0	0	1	0	0	0	0	1	0
0	1	0	0	0	0	0	0	0	0
0	1	0	0	0	0	0	0	0	0
0		1	0	0	1	0	0	0	0
0	0	0	0	0	0	0	0	0	0
0	1	0	0	0	0	0	0	0	0
0	0	1	1	0	1	0	0	0	0
0	1	0	0	0	0	0	0	0	0
0	1	0	1	0	0	0	0	0	0

0	1	0	0	0	0	0	0	0	0
0	0	0	1	0	0	0	0	0	0
0		0	1	1	1	0	0	0	0
0	0	1	1	1	1	0	0	0	0
0	1	0	0	0	0	0	0	0	0
0		0	0	0	1	0	0	0	0
0	1	0	1	0	1	0	0	0	0
0	1	1	0	0	0	0	0	0	0
0	1	1	0	0	0	0	0	0	0
0	0	0	0	0	0	0	0	0	0
0	0	0	0	0	0	0	0	0	0
0	0	0	0	0	0	0	0	0	0
0	1	0	0	0	0	0	0	0	0
0	0	0	0	0	0	0	0	0	0
0	0	0	0	0	0	0	0	0	0
0	0	0	0	0	0	0	0	0	0
0	0	0	0	0	0	0	0	0	0
0	0	0	0	0	0	0	0	0	0
0	0	0	0	0	0	0	0	1	0
0	0	0	0	0	0	0	0	0	0
0	0	0	0	0	0	0	0	0	0
1	1	0	0	0	0	0	0	0	0
0	0	1	0	0	0	0	0	0	0
0	0	0	0	0	0	0	0	0	0
0	0	0	0	0	0	0	0	0	0
0	0	0	0	0	0	0	0	0	0
1	0	1	0	0	1	0	0	0	0
0	0	0	0	0	1	0	0	0	0
0	0	0	0	0	0	0	0	0	0
0	0	0	0	0	1	0	1	0	0
0	0	1	0	0	1	0	0	0	0
0	0	0	0	0	0	0	0	0	0
0	0	0	0	0	0	0	0	0	0
0	0	0	0	0	0	0	0	0	0
0	0	0	0	0	0	0	0	0	0
0	0	0	0	0	0	0	0	0	0
1	1	0	1	1	1	0	1	0	1
0	0	0	0	0	0	0	0	0	0
0	0	0	0	0	1	0	0	0	0
0	0	0	0	0	0	0	0	0	0
0	0	0	0	0	0	0	1	0	0
0	0	0	0	0	1	0	0	0	0
0	1	0	1	1	1	0	1	0	0
0	0	0	0	0	0	0	0	0	0
0	0	0	1	1	0	0	0	0	0
0	0	0	0	0	0	0	0	0	0
0	0	1	0	0	1	0	0	1	0

0	0	0	0	0	0	0	0	0	0
0	0	0	0	0	0	0	0	0	0
0	0	0	0	0	0	0	0	0	0
0	0	0	0	0	0	0	0	0	0
0	0	0	0	0	1	0	0	0	0
0	0	0	0	0	0	0	0	0	0
0	0	0	0	0	0	0	0	0	0
0	0	0	0	0	0	0	0	0	0
0	0	0	1	0	0	0	0	0	0
0	0	0	0	0	0	0	0	0	0
0	0	0	0	0	0	0	0	0	0
1	0	0	0	0	0	0	0	0	0
0	0	0	0	0	0	0	0	0	0
0	0	0	0	0	0	0	0	0	0
0	0	0	1	1	1	0	0	0	0
0	0	0	0	0	0	0	0	0	0
0	0	0	0	0	0	0	0	0	0
0	0	0	0	0	0	0	0	0	0
0	0	0	0	0	0	0	0	0	0
0	1	0	0	0	0	0	1	0	0
0	0	0	0	0	0	0	0	0	0
0	0	0	0	0	0	0	0	0	0
0	0	1	1	0	1	0	0	0	0
0	0	0	0	0	0	0	0	0	0
0	0	1	1	0	0	0	0	0	0
0	0	0	0	0	0	0	0	0	0
0	1	0	0	0	0	0	0	0	0
0	1	0	0	0	0	0	0	0	0
0	0	0	0	0	0	0	0	0	0
0	0	0	0	0	0	0	0	0	0
0	1	0	1	0	0	0	0	0	0
0	0	0	0	0	0	0	0	0	0
0	0	0	0	0	0	0	0	0	0
0	1	0	0	0	0	0	0	0	0
0	0	0	0	0	0	0	0	0	0
0	0	0	0	0	0	0	0	0	0
0	1	0	0	0	0	0	0	0	0
0	0	0	0	0	0	0	0	0	0
0	0	0	0	0	0	0	0	0	0
0	0	1	0	0	0	0	0	0	0
0	0	1	0	1	0	0	1	0	0
0	0	0	0	0	0	0	0	0	0
0	0	0	0	0	0	0	0	0	0
0	0	0	0	0	0	0	0	0	0
1	0	0	0	0	0	0	0	0	0
0	0	0	0	0	0	0	0	0	0
0	1	0	0	0	0	0	0	0	0

0	1	1	1	0	0	0	0	0	0
0	1	0	1	0	1	0	1	0	0
0	1	0	1	0	1	1	0	0	0
0	0	1	1	1	0	0	0	0	0
0	1	0	1	0	0	0	1	1	0
0	0	0	0	0	0	0	0	0	0
0	0	0	0	0	0	0	0	0	0
0	1	0	0	1	1	1	1	0	0
1	1	0	1	1	1	1	0	0	0
0	1	0	0	0	1	0	0	0	0
1	1	1	0	0	0	0	0	0	0
0	1	1	1	1	1	0	0	0	0
0	1	1	1	1	1	0	0	0	0
0	1	1	0	1	1	0	0	0	0
0	0	0	0	0	0	1	0	0	0
0	0	0	0	0	0	1	0	0	0
0	0	0	0	0	0	0	0	0	0
0	1	1	1	1	1	1	1	0	0
0	1	0	1	1	0	1	0	0	0
0	1	0	1	0	1	0	0	0	0
0	0	0	0	0	0	0	0	0	0
0	1	0	0	0	0	0	0	0	0
0	0	0	0	0	1	0	0	0	0
0	1	0	1	0	0	0	0	0	0
0	0	0	0	0	0	0	0	0	0
0	1	0	0	1	1	0	0	0	0
0	1	0	1	0	0	0	0	0	0
0	0	0	0	0	1	0	0	0	0
0	0	0	0	0	1	0	0	0	0
0	0	0	0	0	1	0	0	0	0
0	1	0	0	0	0	0	0	0	0
0	0	0	0	0	1	0	0	0	0
0	0	0	0	1	1	0	1	0	0
0	0	0	0	0	0	0	0	0	0
0	0	0	1	0	0	0	0	0	0
0	0	0	0	0	0	0	0	0	0
0	0	0	0	0	0	0	0	0	0
0	0	1	1	0	0	0	0	0	0
0	0	0	1	0	0	0	0	0	0
0	0	1	1	0	1	0	1	0	0
0	0	0	1	0	0	0	1	1	0
0	0	0	0	0	0	0	0	0	0
0	0	0	1	0	0	0	0	0	0
0	0	0	0	0	0	0	0	1	0
0	0	0	0	0	0	0	0	0	0
0	0	0	0	0	0	0	0	0	0
0	0	0	0	0	0	0	0	0	0

1	1	1	1	1	0	0	1	1	0
1	1	1	1	0	0	0	1	0	0
0	1	0	1	0	1	0	1	0	0
0	1	1	1	1	1	0	0	0	0
0	1	0	1	0	1	0	0	0	0
0	1	0	0	0	0	0	0	0	0
0	0	1	0	0	0	0	1	1	0
0	0	0	0	0	0	0	0	0	0
0	1	0	0	0	0	0	0	0	0
0	1	0	1	1	1	0	0	0	0
0	1	1	1	0	1	0	0	0	0
0		0	0	0	1	0	0	0	0
0	0	0	0	0	0	0	0	0	0
0		0	0	1	1	0	0	0	0
0	0	0	1	0	0	0	0	0	0
0	1	0	0	0	0	0	0	0	0
0	1	0	0	0	0	0	0	0	0
0	0	0	0	0	0	0	0	0	0
0	0	0	0	0	0	0	0	0	0
0	0	0	0	0	0	0	0	0	0
0		0	1	0	0	0	0	0	0
0	1	0	1	0	0	0	0	0	0
0	0	0	0	0	0	0	0	0	0
0	1	0	0	0	0	0	0	0	0
0	1	0	0	0	0	0	0	0	0
0	0	1	0	0	1	0	0	0	0
0	0	0	1	0	0	0	0	0	0
0	1	0	1	0	0	0	0	0	0
0	1	1	1	0	1	0	0	0	0
0	1	0	0	0	0	0	1	0	0
0	1	0	1	1	1	0	0	0	0
0	1	1	0	0	0	0	0	0	0
0	1	0	0	0	0	0	0	0	0
0	0	0	0	0	0	0	0	0	0
0	0	0	0	0	0	0	0	0	0
0	1	0	1	1	1	0	0	0	0
0	0	0	1	0	1	0	0	0	0
0	0	0	0	0	0	0	0	0	0
0		1	0	1	1	0	1		
0	0	0	0	0	0	0	0	0	0
0	0	0	0	0	0	0	0	0	0
0	0	0	0	0	0	0	0	0	0
0	0	0	0	0	0	0	0	0	0
0	0	0	0	0	0	0	0	0	0
0	0	0	1	0	0	0	0	0	0
0	0	0	0	0	0	0	0	0	0
0	0	0	0	0	0	0	0	1	0

1	1	1	1	1	1	1	1	1	1
0	0	0	0	0	0	0	1	0	0
0	0	0	0	0	1	1	0	0	0
0	0	0	0	0	0	0	0	0	0
0	0	0	0	0	0	0	0	0	0
0	0	1	0	0	1	0	0	0	0
0	0	0	0	0	0	0	0	0	0
0	1	0	1	0	0	0	0	0	0
0	0	0	0	0	1	0	0	0	0
0	0	0	0	0	0	0	0	0	0
0	0	0	0	0	0	0	0	0	0
0	0	0	1	1	1	0	0	0	0
0	1	0	0	1	1	1	0	0	0
0	0	0	0	0	0	0	0	0	0
0	0	1	0		1	0	0	0	0
0	0	0	0	0	0	0	0	0	0
0	0	0	0	0	0	0	0	0	0
0	1	0	0	0	0	0	0	0	0
0	0	0	0	0	0	0	0	0	0
0	1	0	1	0	1	0	0	0	0
0	1	1	1	1	1	0	1	0	0
0	0	0	0	0	0	0	0	0	0
0	0	0	0	0	0	0	0	0	0
0	0	0	0	0	0	0	0	0	0
0	0	0	0	1	1	0	1	0	0
0	0	0	0	0	0	0	1	0	0
1	1	1	0	1	1	0	0	0	0
0	0	0	0	0	0	0	0	0	0
0	0	0	0	1	1	0	1	0	0
0	1	0	0	1	1	1	1	0	0
0	0	0	0	0	0	0	0	0	0
0	0	0	0	0	0	1	0	0	0
0	0	0	1	0	0	1	1	1	0
0	0	0	0	0	0	0	0	0	0
0	0	0	0	0	0	0	0	0	0
0	0	0	0	0	0	0	0	0	0
0	0	0	0	0	0	0	0	0	0
0	0	0	0	0	0	0	0	0	0
0	0	0	0	0	0	0	0	0	0
0	0	0	0	0	0	0	0	0	0
0	0	0	0	0	0	0	0	0	0
0	0	0	0	0	0	0	0	0	0
0	0	0	0	0	0	0	0	0	0
0	0	0	0	0	0	0	0	0	0
0	0	0	0	0	0	0	0	1	0
1	0	0	1	0	0	0	0	1	0
0	0	0	0	0	0	0	0	0	0

0	1	1	1	1	1	1	1	1	1
0	0	0	0	0	0	0	0	0	0
0	0	0	0	0	0	0	0	0	0
0	0	0	0	0	1	1	0	0	0
0	0	0	0	0	0	0	0	0	0
0	0	0	0	0	0	0	0	0	0
0	0	0	0	0	0	0	0	0	0
0	0	0	0	0	0	0	0	0	0
0	0	0	0	0	0	0	0	0	0
0	0	0	0	0	0	0	0	0	0
0	0	0	0	0	0	0	0	0	0
0	0	0	0	0	1	0	0	0	0
		0	0	0	0	0	0	0	0
0	0	0	0	0	0	0	0	1	0
0	0	0	0	0	0	0	0	0	0
0	0	0	0	1	0	0	0	0	0
0	0	1	0	1	1	0	0	0	0
0	0	0	0	0	0	0	0	0	0
0	0	0	0	0	0	0	0	0	0
0	0	0	0	1	1	0	0	0	0
0	0	0	0	0	1	0	0	0	0
0	0	1	0	1	1	0	0	0	0
0	0	0	0	0	0	0	0	0	0
0	0	0	0	0	0	0	0	0	0
0	0	1	0	0	0	0	0	0	0
0	0	0	0	0	0	0	1	0	0
0	0	0	0	0	0	0	0	0	0
0	0	0	1	0	1	0	0	0	0
0	0	1	0	0	1	0	0	0	0
0	0	0	0	0	0	0	0	0	0
0	0	0	0	0	1	0	0	0	0
0	0	0	0	0	0	0	0	0	0
0	0	0	0	0	0	0	0	0	0
0	0	0	0	0	0	0	0	0	0
0	0	0	0	0	1	0	0	0	0
0	0	1	0	0	1	0	0	0	0
0	0	1	0	0	1	0	0	0	0
0	0	1	0	0	1	0	0	0	0
0	0	1	0	0	1	0	0	0	0
0	0	0	0	0	1	0	0	0	0
0	0	0	0	0	0	0	0	0	0
0	0	0	1	1	1	0	0	0	0
0	0	1	1	1	1	0	0	0	0
0	0	0	1	0	0	0	1	0	0
0	0	0	1	0	0	0	1	0	0
0	0	0	0	0	0	0	0	0	0
0	0	0	0	0	0	0	0	0	0

0	0	0	0	0	1	0	0	0	0
0	0	0	0	0	0	0	0	0	0
0	0	0	0	0	1	0	0	0	0
0	0	0	0	0	0	0	0	0	0
0	0	0	0	0	0	0	1	0	0
0		0	1	0	0	0	0	0	0
0	1	0	0	0	0	0	0	0	0
0	1	0	0	0	0	0	0	0	0
0	1	0	0	0	0	0	0	0	0
0	0	0	0	0	0	0	0	0	0
0	1	0	0	0	0	0	0	0	0
0	1	0	0	0	0	0	0	0	0
0	0	0	0	0	0	0	0	0	0
0	0	0	0	0	0	0	0	0	0
0	1	0	0	0	0	0	0	0	0
0	0	0	0	0	0	0	0	0	0
0	0	0	0	0	0	0	0	0	0
0	0	0	0	0	0	0	0	0	0
0	0	0	0	0	0	0	0	0	0
0	1	0	0	0	0	0	0	0	0
0	0	0	0	0	0	0	0	0	0
0	1	0	0	0	0	0	0	0	0
0	1	0	0	0	0	0	0	0	0
0	1	0	0	0	0	0	0	0	0
0	0	0	0	0	0	0	0	0	0
0	0	0	0	1	0	0	0	0	0
0	1	0	0	0	0	0	0	0	0
0	0	0	0	0	0	0	0	0	0
0	1	0	0	0	0	0	0	0	0
0	1	0	0	0	0	0	0	0	0
0	1	0	0	0	0	0	0	0	0
0	1	0	0	0	0	0	0	0	0
0	0	0	0	0	0	0	0	0	0
0	0	0	0	0	0	0	0	0	0
0	1	0	0	0	0	0	0	0	0
0	1	0	1	1	0	0	0	0	0
0	1	0	1	1	1	0	0	0	0
0	1	0	1	0	1	0	0	0	0
0	1	0	1	0	1	0	0	0	0
0	1	0	0	0	0	0	0	0	0
0	0	1	1	0	0	0	0	0	0
0	1	0	0	0	0	0	0	0	0
0	1	1	1	0	1	0	0	0	0
0	0	0	0	0	0	0	0	0	0
0	1	0	0	1	1	0	0	0	0
0	0	0	0	0	1	0	1	0	0
0	0	0	1	0	0	0	0	0	0

0	0	1	1	0	1	0	0	0	0
0	1	0	0	1	1	0	0	0	0
0	1	0	0	1	0	0	0	0	0
0	1	0	1	0	1	0	0	0	0
0	1	1	1	0	0	0	0	0	0
0	1	1	1	0	0	0	0	0	0
0	0	0	0	0	0	0	0	0	0
0	1	0	0	0	0	0	0	0	0
0	0	1	0	0	0	0	0	0	0
0	1	0	0	1	1	0	0	0	0
0	0	0	0	0	1	0	0	0	0
0	1	0	1	0	1	0	1	0	0
0	1	0	0	0	0	0	0	0	0
0	0	0	0	0	0	0	0	0	0
0	1	0	0	1	1	0	0	0	0
0	1	0	1	0	0	0	0	0	0
0	0	0	0	0	0	0	0	0	0
0	0	0	0	0	0	0	0	0	0
0	0	0	0	0	0	0	0	0	0
0	1	0	1	0	1	0	0	0	0
0	0	0	0	0	0	0	0	0	0
0	0	0	0	0	0	0	0	0	0
0	1	0	0	0	0	0	0	0	0
0	0	0	0	0	0	0	0	0	0
1	0	0	1	1	0	0	1	0	1
0	0	0	0	0	0	0	0	0	0
0	0	1	0	1	1	0	0	0	0
0	1	0	0	0	0	0	0	0	0
0	0	0	0	0	1	0	0	0	0
0	0	0	0	0	0	0	0	0	0
0	0	1	1	0	0	0	0	0	0
0	1	0	1	0	0	1	0	0	0
0	0	0	0	0	0	0	0	0	0
0	1	0	0	0	1	0	0	0	0
0	0	0	0	0	1	0	0	0	0
0	1	1	1	1	0	0	1		0
0	0	0	0	0	0	0	0	0	0
0	0	0	0	0	1	0	0	0	0
0	1	0	1	0	0	0	0	0	0
0	0	0	0	0	0	0	0	0	0
0	1	0	0	0	0	0	0	0	0
0	0	0	0	0	0	0	1	0	0
0	0	0	0	0	0	0	0	0	0
0	1	0	0	0	0	0	0	0	0
0	0	0	0	0	1	0	0	0	0
0	1	0	0	0	0	0	0	0	0
0	0	0	0	0	0	0	0	0	0

0	1	1	0	0	0	0	0	0	0
0	0	0	0	0	0	0	0	0	0
0	1	0	1	0	1	0	0	0	0
0	0	0	0	0	0	0	0	0	0
0	0	0	0	0	0	0	0	0	0
0	1	0	0	0	0	0	0	0	0
0	0	0	0	0	0	0	0	0	0
0	0	0	1	0	0	0	0	0	0
0	0	1	1	0	0	1	1	1	0
0	1	0	1	0	1	0	0	0	0
0	0	0	0	0	0	1	0	1	0
0	0	0	0	0	0	0	0	0	0
0	0	0	0	0	0	0	0	0	0
0	0	0	1	0	0	0	0	0	0
0	0	0	0	0	0	0	0	0	0
0	0	0	0	0	0	0	1	1	0
0	0	0	1	0	0	0	1	0	0
0	0	0	0	0	0	0	0	0	0
0	0	0	0	0	0	0	0	0	0
0	0	1	1	0	0	0	0	0	0
0	0	0	0	0	0	0	0	0	0
0	1	1	1	0	0	0	1	1	0
0	0	0	0	0	0	0	0	0	0
0	0	0	0	0	0	0	0	0	0
	0	0	0	0	0	0	0	0	0
0	0	0	0	0	0	0	0	0	0
0	0	0	0	0	0	1	0	1	0
0	0	0	0	0	0	0	0	0	0
0	0	0	0	0	0	0	0	0	0
0	0	0	0	0	0	0	0	0	0
0	0	0	0	0	0	0	0	0	0
0	0	0	0	0	0	0	0	0	0
1	0	1	1	0	0	1	0	1	0
0	0	0	0	0	0	0	0	0	0
0	0	0	0	0	0	0	0	0	0
0	0	0	0	0	0	0	0	0	0
0	1	1	1	0	0	0	1	1	0
0	0	0	0	0	0	0	0	0	0
0	0	0	0	0	0	0	0	0	0
0	0	0	0	0	0	0	0	0	0
0	0	0	0	0	0	0	0	0	0
0	0	0	0	0	0	0	0	0	0
0	0	1	1	0	0	0	0	0	0
1	1	0	0	1	0	0	1	0	1
0	1	1	1	1	0	0	1	0	1
1	0	0	0	0	1	0	0	1	0
0	1	0	1	0	0	0	0	0	0

0	0	1	1	0	1	0	1	1	0
0	1	0	1	0	0	0	0	1	0
0	0	0	0	1	1	0	1	0	0
0	0	0	0	0	1	0	0	0	0
0	1	0	0	0	0	0	0	0	0
0	1	1	1	0	0	0	1	0	0
0	1	1	1	0	1	0	0	0	0
0	0	0	0	0	0	0	0	0	0
1	0	1	0	0	0	0	0	1	0
0	0	0	0	0	0	0	0	0	0
0	0	0	1	0	0	1	0	0	0
0	0	0	0	0	0	0	0	0	0
0	0	0	0	0	1	0	0	0	0
0	0	0	0	0	0	0	0	0	0
1	0	1	0	0	0	0	1	0	0
0	0	1	1	0	0	0	0	0	0
1	1	0	1	1	1	1	1	0	0
0	0	0	0	0	0	0	0	0	0
0	1	0	1	0	0	0	1	0	0
0	0	0	0	0	0	0	1	0	0
0	0	0	0	0	0	0	1	0	0
0	0	0	0	0	0	0	0	0	0
0	0	0	0	0	1	0	0	0	0
0	1	0	0	0	1	0	0	1	0
0	0	1	1	0	0	0	1	1	0
0	1	0	0	0	0	0	0	0	0
0	1	0	0	0	0	0	0	0	0
0		1	1		1	0	0	0	0
0	1	0	0	0	0	0	0	0	0
0	1	0	0	0	0	0	0	0	0
0		0	1	0	0	0	0	0	0
0	0	0	0	0	0	0	0	0	0
0	1	0	0	0	0	0	0	0	0
0	1	0	0	0	0	0	0	0	0
0	1	0	0	0	0	0	0	0	0
0		0	0	0	0	0	0	0	0
0	1	0	1	0	0	0	0	0	0
0		0	0	1	1	0	0	0	0
0	0	0	0	0	0	0	0	0	0
0	1	0	0	0	1	0	0	0	0
0	1	0	0	1	0	0	0	0	0
0		0	0	0	0	0	0	0	0
0		0	0	0	1	1	0	0	0
0	1	0	0	0	0	0	0	0	0
0	1	0	0	0	0	0	0	0	0
0	1	0	0	0	0	0	0	0	0
0	1	0	0	1	1	0	0	0	0

0	0	0	0	0	0	0	0	0	0
0	1	0	0	1	0	0	0	0	0
0	0	0	0	0	0	0	0	0	0
0	1	0	1	0	1	1	0	1	0
0	1	0	0	0	0	0	0	0	0
0	1	0	0	0	0	0	0	0	0
0		0	0	0	0	0	0	0	0
0	0	0	0	0	0	0	0	0	0
0	1	0	1	0	0	0	0	0	0
0	1	0	0	0	0	0	0	0	0
0	0	0	0	0	0	0	0	0	0
0	0	0	0	0	0	0	0	0	0
0		1	1	0	0	0	0	0	0
0		0	1	0	0	0	0	0	0
0		0	1	1	0	0	0	1	0
0	1	0	0	0	0	0	0	0	0
0		0	0	0	0	0	0	0	0
0	1	0	1	0	0	0	0	0	0
0		0	0	0	0	0	0	0	0
0	1	0	0	0	0	0	0	0	0
0	1	0	0	0	0	0	0	0	0
0	1	0	0	1	1	0	1	0	0
0		0	0	1	1	0	0	0	0
0	1	0	0	0	0	0	0	0	0
0	0	0	0	0	0	0	0	0	0
0	1	0	0	0	0	0	0	0	0
0	0	0	0	0	0	0	0	0	0
0	1	0	1	0	1	0	0	0	0
0	1	0	0	0	0	0	0	0	0
0	1	0	0	0	0	0	0	0	0
0	0	0	1	0	0	0	0	0	0
0	0	0	1	0	0	0	0	0	0
0	1	1	0	1	1	0	0	0	0
0	1	0	1	1	1	0	0	0	0
0	1	0	1	0	0	0	0	0	0
0	1	0	0	1	1	0	0	0	0
0	0	0	0	0	0	0	0	0	0
0	0	0	0	0	0	0	0	0	0
0	0	0	0	0	0	0	0	0	0
0	0	0	0	0	0	0	0	0	0

bother	ete	cheer	good	mind	depress	effort	hope	failure	fear
0	0	1	1	2	1	1	1	1	0
0	0	0	0	0	0	0	0	0	0
0	0	0	0	0	0	0	0	0	0
0	1	1	1	1	1	1	1	1	1
0	0	0	0	0	0	0	0	0	0
0	0	0	0	0	0	0	0	0	0
0	0	0	0	0	0	0	0	0	0
0	0	0	0	0	0	0	0	0	0
0	0	0	0	0	0	0	0	0	0
0	0	0	0	0	0	0	0	0	0
0	0	0	0	0	0	0	0	0	0
0	0	0	0	1	3	3	0	3	3
0	0	0	0	0	0	0	0	0	0
0	0	0	0	0	0	0	0	0	0
0	0	0	0	0	0	0	0	0	0
0	2	0	0	1	1	1	1	1	1
0	0	0	0	0	0	0	0	0	0
0	2	0	3	1	1	2	0	0	1
1	0	3	3	0	0	3	3	3	0
0	0	0	0	0	0	0	1	0	0
0	0	1	1	1	0	1	1	0	1
3	0	1	0	3	3	2	0	3	1
1	0	0	3	1	0	0	3	1	0
0	0	0	3	0	0	0	3	0	0
1	0	0	3	0	0	3	3	0	1
0	3	0	3	0	0	0	0	0	0
1	1	2	3	3	0	3	3	0	0
3	1	3	1	3	1	3	3	3	3
3	3	3	0	3	3	2	3	3	0
3	3	3	0	1	3	0	3	3	3
0	0	0	0	0	0	0	0	0	0
0	3	0	3	0	0	0	3	0	0
1	1	0	2	2	1	0	0	3	1
3	2	0	1	1	2	2	2	0	0
0	0	0	0	0	0	0	3	0	0
0	0	0	3	0	0	0	3	0	0
0	0	0	3	1	2	3	1	2	0
0	0	0	3	1	0	0	3	0	1
3	0	0	3	0	0	0	3	1	0
0	3	0	3	1	1	0	3	2	0
0	0	0	3	0	0	3	3	0	0
0	0	0	3	0	1	1	3	0	0
0	0	1	3	1	1	2	0	0	1
3	0	3	3	0	3	3	3	3	3
0	0	0	2	0	0	1	1	0	1
0	0	0	3	2	1	0	3	0	0

0	3	0	0	0	0	0	0	0	1
0	0	1	3	0	2	0	2	3	0
0	1	0	3	0	0	2	3	0	0
0	0	0	0	0	0	1	1	0	0
0	0	0	3	0	0	0	3	0	0
0	0	0	0	0	0	0	0	0	1
0	3	3	0	1	0	3	3	3	3
0	3	3	0	1	0	3	3	3	3
3	3	3	0	2	3	3	2	3	3
1	0	0	3	0	0	2	0	3	0
0	0	0	3	0	0	0	0	0	0
0	0	0	3	1	0	3	3	1	0
0	1	1	3	1	1	1	1	0	0
0	1	2	3	0	2	2	3	0	0
0	1	0	0	0	0	0	0	0	0
0	2	0	0	0	2	0	3	0	0
0	0	0	3	0	0	0	3	1	0
0	0	0	3	0	0	3	3	0	0
0	0	0	3	0	0	0	3	0	0
3	3	0	2	2	0	3	3	0	0
0	0	1	3	0	1	1	1	1	0
0	0	0	0	0	0	0	0	0	0
0	0	0	0	0	0	0	0	1	0
0	0	0	0	0	0	0	0	0	0
0	0	0	0	0	0	0	0	0	0
0	1	1	1	0	1	1	1	1	0
0	0	0	0	1	0	1	0	1	0
1	1	1	1	1	1	1	1	1	1
0	0	0	0	0	0	0	0	0	0
0	0	0	0	0	0	0	0	0	0
0	0	0	0	0	0	0	0	0	0
0	0	0	0	0	0	0	0	0	0
0	0	0	0	0	0	0	0	0	0
1	0	1	1	1	1	1	1	1	1
0	1	0	1	0	0	0	0	0	0
1	1	1	0	0	0	0	0	0	0
0	1	0	1	0	1	0	1	0	0
3	1	1	1	1	1	1	1	1	1
1	1	1	1	1	1	1	1	1	3
3	1	1	1	1	0	0	0	0	0
0	1	0	0	0	0	1	0	0	1
0	0	0	0	0	1	1	1	1	1
0	0	0	0	0	0	0	0	0	0
0	1	3	1	0	1	1	0	0	0
0	0	0	1	1	0	1	0	0	1
0	0	0	0	0	0	0	1	1	1
0	0	0	0	1	1	0	0	0	0

1	1	1	1	1	1	1	1	1	1
0	0	0	0	0	0	0	0	0	0
1	1	1	1	1	1	1	1	1	1
2	0	1	2	0	1	0	3	0	0
2	0	1	1	1	2	0	3	0	2
1	0	1	0	1	3	0	1	1	0
1	0	1	1	3	1	0	1	3	1
0	0	0	3	0	0	0	2	0	0
2	0	2	0	2	2	0	2	0	2
2	0	1	2	0	0	0	3	0	0
0	0	0	3	0	0	0	2	0	0
0	0	0	0	0	0	0	3	0	0
0	1	3	0	1	3	3	3	1	0
0	0	0	0	0	0	0	3	0	0
3	0	1	0	1	3	0	2	1	1
0	0	0	1	0	0	0	3	0	0
2	0	0	0	3	3	0	0	2	0
2	0	1	2	2	3	2	0	1	2
2	0	1	1	2	2	0	0	3	2
0	0	0	1	1	3	0	1	3	0
0	0	0	3	0	0	1	3	0	0
0	0	0	0	0	0	0	3	0	1
0	0	0	2	0	1	0	2	0	0
0	0	0	0	0	0	0	2	0	0
0	0	0	3	0	0	0	3	0	0
0	0	0	3	3	3	0	3	1	0
0	0	0	0	0	0	0	0	0	0
1	0	1	1	0	3	1	0	1	1
1	0	1	1	0	3	1	0	1	1
0	0	0	0	0	0	0	0	0	0
0	0	0	0	0	0	0	0	0	0
0	0	0	0	0	0	0	0	0	0
1	0	1	0	0	0	0	0	0	0
1	1	0	0	0	0	0	0	0	0
1	1	1	1	1	1	0	0	0	0
0	0	0	0	0	0	0	1	3	0
0	0	0	0	0	0	0	0	0	0
0	0	0	0	0	0	0	0	0	0
0	0	0	0	0	0	0	0	0	0
1	1	1	1	1	1	1	0	0	0
1	0	1	1	1	1	1	1	3	3
0	0	0	1	1	0	1	3	0	0
1	0	0	0	0	0	1	1	0	0
0	0	0	0	0	0	0	0	0	0
0	0	0	0	0	0	0	0	0	0
1	1	1	1	3	1	0	0	1	3
1	3	3	0	1	1	1	1	1	1

0	0	0	3	0	0	0	3	0	0
0	0	0	3	0	0	0	3	0	0
0	0	0	0	0	0	0	0	0	0
0	0	0	3	0	0	0	3	1	0
0	2	1	3	1	1	1	3	1	0
0	1	0	3	0	0	0	3	0	0
0	0	0	1	3	0	0	3	3	0
0	0	0	0	0	0	0	0	0	0
0	0	0	3	0	0	3	3	0	0
0	0	0	3	0	0	3	3	0	0
0	0	0	3	0	0	0	0	0	0
0	0	0	0	0	0	0	0	0	0
0	0	0	0	0	0	0	0	0	1
1	0	0	0	0	0	0	0	0	0
0	0	0	0	0	0	0	0	0	0
0	0	0	0	0	0	0	0	0	0
0	0	0	3	0	0	2	3	0	0
0	0	0	3	0	0	0	3	0	0
0	0	0	3	0	1	0	1	3	1
0	0	0	3	0	1	0	1	3	1
0	0	0	3	0	0	3	2	0	0
0	0	0	3	0	0	3	3	0	0
0	0	0	0	0	0	0	3	0	0
1	0	1	2	1	2	0	1	1	2
0	0	0	3	0	0	3	3	0	0
0	0	0	3	0	0	3	3	0	0
0	0	0	3	0	0	3	3	0	0
0	1	2	2	1	0	0	3	0	0
0	0	0	3	0	0	3	3	0	0
1	1	1	2	1	0	2	0	0	1
0	0	0	3	0	0	3	3	0	0
0	0	0	3	0	0	3	3	1	0
0	0	0	3	0	0	3	3	0	0
0	0	0	3	0	0	0	3	0	0
0	0	0	0	0	0	0	0	0	0
0	0	0	3	0	0	3	3	0	0
0	0	0	2	0	0	3	2	0	1
0	0	1	1	1	1	2	1	1	1
0	0	0	3	0	1	0	3	0	0
1	1	1	2	1	1	3	3	1	0
0	0	0	3	0	0	3	3	0	0
0	0	0	3	0	0	3	3	0	0
1	1	1	2	2	1	1	2	1	1
0	0	0	3	0	0	3	3	0	0
0	0	0	3	0	0	1	3	0	0
1	0	0	1	0	0	1	1	1	0
0	0	0	3	0	0	3	3	0	0

0	0	0	3	0	0	3	3	0	0
1	2	0	1	1	2	1	2	0	0
0	0	0	0	0	0	3	3	0	0
0	0	0	2	0	0	3	3	0	0
0	0	0	3	0	0	2	3	1	0
0	0	0	3	0	0	2	3	0	0
0	0	0	3	0	0	3	3	0	0
0	1	0	1	0	1	1	3	0	1
0	0	0	3	0	0	3	2	0	0
2	1	0	1	0	0	1	1	0	0
0	0	0	3	0	0	3	1	0	0
0	0	0	3	0	0	3	3	0	0
0	0	0	3	0	0	3	3	0	0
0	0	0	3	0	0	3	2	0	0
0	1	2	3	0	0	0	0	0	0
1	1	1	1	1	1	1	1	1	1
0	0	0	0	0	0	0	0	0	0
0	0	0	0	0	0	0	0	0	0
0	0	0	0	0	0	0	0	0	0
0	0	0	0	0	0	0	0	0	0
0	0	0	0	0	0	0	0	1	1
0	0	0	0	0	0	0	0	0	0
0	2	0	3	0	0	0	1	0	0
0	0	0	0	0	0	0	0	0	0
1	1	1	1	1	1	1	1	1	1
0	0	0	0	0	0	0	0	0	0
0	0	0	0	0	0	0	1	1	1
0	0	0	0	0	0	0	0	0	0
0	0	0	0	0	0	0	0	0	0
0	0	0	0	0	0	0	0	0	0
3	3	3	3	1	1	1	3	3	3
0	0	0	0	0	0	0	0	0	0
0	0	0	0	0	0	0	0	0	0
1	0	0	0	0	0	0	0	0	0
0	0	1	2	0	1	0	2	0	0
0	0	3	3	0	0	0	3	3	3
0	0	0	3	0	0	0	3	0	0
1	0	0	3	0	0	0	3	0	0
2	2	1	2	0	1	1	2	1	1
1	0	0	1	2	2	2	0	1	1
0	0	0	3	0	0	0	3	0	0
0	0	0	3	3	0	3	3	0	0
3	1	2	1	1	2	1	2	1	0
3	0	2	0	3	3	3	1	1	3
1	0	0	3	0	0	0	0	1	0
0	0	0	3	0	0	0	3	0	0
1	0	1	2	0	1	0	2	1	1

0	0	0	3	0	0	0	3	0	0
0	0	0	3	0	0	0	3	0	0
0	0	0	3	0	0	0	0	0	0
0	0	0	3	0	0	1	3	0	0
0	0	0	0	0	0	0	0	0	0
0	0	0	3	0	0	2	3	0	0
0	0	0	3	0	0	2	2	0	0
0	0	0	1	0	0	0	3	0	0
0	0	0	3	0	0	0	0	2	0
0	0	0	3	0	0	0	1	0	0
3	1	1	1	1	1	1	1	1	0
0	0	0	3	0	0	1	3	0	0
0	0	0	3	0	0	0	3	0	0
0	3	1	1	1	1	3	3	0	1
0	0	1	1	2	1	2	1	1	2
0	1	0	1	0	0	0	0	0	0
0	0	0	3	0	0	1	3	0	0
0	1	1	1	2	0	1	2	3	0
0	1	1	1	2	0	1	2	3	0
0	0	0	3	0	0	0	3	0	0
0	0	0	3	0	0	0	3	0	0
0	1	1	0	2	2	1	3	0	1
0	0	0	0	1	2	1	1	1	0
3	0	3	0	1	3	1	0	3	0
3	3	3	0	3	1	3	3	1	1
0	0	0	0	1	0	0	1	0	0
0	0	1	1	0	0	0	3	0	1
3	1	1	0	1	1	1	1	0	0
0	3	0	1	0	3	3	3	0	0
0	0	0	0	0	0	0	0	0	0
0	0	0	0	0	0	0	0	0	0
0	1	0	1	0	0	3	3	0	0
0	1	0	1	0	0	3	3	0	0
0	0	0	0	0	0	0	0	0	0
3	3	3	3	3	0	3	0	3	3
0	0	0	0	0	0	1	1	3	3
0	1	1	1	1	1	0	0	0	0
0	0	0	0	0	0	0	0	0	0
0	0	0	0	0	0	0	0	0	0
0	0	0	0	0	0	0	0	0	0
0	0	0	0	0	0	0	0	0	0
1	1	1	1	1	1	2	2	2	2
0	0	0	0	0	0	0	0	0	0
0	0	0	0	0	0	0	0	0	0
0	0	1	0	1	1	0	0	1	1
1	1	2	1	1	1	1	1	0	0
3	3	0	0	0	0	0	3	0	0

0	0	0	0	0	0	0	0	0	0
0	1	0	1	0	0	2	3	0	0
0	0	0	0	0	0	0	0	0	0
0	0	0	2	0	0	3	3	0	0
1	0	0	1	0	0	0	1	0	1
0	0	0	0	0	1	1	1	3	0
0	0	0	2	1	0	0	1	0	0
0	0	0	3	0	0	0	3	0	0
0	3	0	3	1	0	3	3	0	0
0	0	0	3	0	0	0	3	0	0
0	0	0	3	0	0	0	3	0	0
2	3	2	0	0	1	1	1	0	3
0	0	0	3	0	0	0	3	0	0
1	1	2	0	0	0	0	1	1	0
1	1	2	0	0	0	0	1	1	0
0	3	0	2	0	0	0	3	0	0
3	2	3	0	1	3	1	3	0	1
3	1	1	1	1	1	1	1	1	1
0	0	0	2	0	0	3	3	1	0
3	1	0	1	0	1	0	1	0	0
0	0	0	3	0	0	0	0	2	1
0	1	0	2	0	0	2	3	1	3
0	3	0	3	0	0	0	3	0	0
1	1	0	0	0	1	0	1	1	0
2	0	0	2	1	0	1	2	2	0
3	3	1	0	2	3	3	1	3	0
0	0	0	3	1	0	0	3	0	0
0	0	0	3	0	0	1	3	0	0
0	0	0	0	0	0	0	0	0	0
0	0	0	3	0	0	0	3	0	0
0	0	0	0	0	1	0	0	0	0
0	0	0	3	0	0	3	3	1	0
1	0	1	3	0	0	0	3	0	0
0	1	0	1	0	1	0	1	0	0
1	0	2	1	0	2	0	0	0	1
0	3	0	3	2	1	0	3	2	0
0	0	0	3	1	0	0	3	0	0
0	0	0	1	0	2	0	0	0	0
1	1	1	3	1	0	1	2	3	0
0	1	1	3	1	0	2	3	0	0
0	0	0	2	0	0	0	0	0	0
1	0	0	3	0	0	3	3	0	0
0	0	0	3	0	1	0	3	0	0
0	0	0	3	1	0	1	0	0	0
2	0	0	0	0	1	0	0	0	0
0	1	0	1	1	0	1	1	2	0
0	0	0	3	0	0	0	3	0	0

0	0	0	3	0	0	0	3	0	0
0	0	0	2	0	0	2	2	0	0
1	0	2	0	1	0	2	1	1	0
3	3	3	0	3	3	0	0	3	0
2	2	0	2	2	1	0	3	0	0
0	0	0	0	1	0	0	3	0	0
0	0	0	3	0	0	0	0	0	0
0	0	0	3	0	0	0	0	0	0
1	2	0	3	2	3	1	2	1	2
3	0	0	0	0	0	0	0	0	0
0	0	0	3	2	0	0	3	3	0
3	0	0	3	0	2	0	3	3	0
3	3	0	0	0	3	1	0	3	1
2	1	1	3	1	1	1	1	1	0
0	0	0	3	0	0	0	3	0	0
0	0	0	0	0	0	0	1	1	1
0	0	0	3	0	1	1	3	0	0
0	0	0	3	0	0	3	3	0	0
0	1	1	1	2	3	2	2	3	1
0	0	0	0	0	0	0	0	0	0
0	0	0	3	0	0	0	3	0	0
0	0	0	3	0	0	3	3	0	0
0	0	0	3	0	2	0	3	0	0
1	2	0	3	2	0	2	3	0	0
1	2	1	3	0	0	0	3	0	0
0	0	0	0	0	0	0	3	0	0
0	0	0	3	0	0	0	3	0	0
0	0	0	3	0	0	0	3	0	0
1	1	2	0	2	2	2	0	2	1
0	0	0	3	0	0	0	3	0	0
0	3	3	0	3	0	0	2	0	0
0	0	1	1	1	1	1	1	1	1
2	0	0	0	0	0	0	0	0	0
0	0	0	0	0	0	0	0	0	0
1	0	0	0	0	0	0	0	0	0
0	0	0	0	0	0	0	0	0	0
0	0	0	0	0	0	1	1	1	1
0	0	1	0	1	0	0	0	1	0
0	0	0	0	0	0	0	0	0	0
0	0	0	0	0	0	1	1	0	0
3	2	2	0	0	0	0	1	0	0
1	1	0	1	1	0	1	1	0	1
0	0	0	0	0	0	0	0	0	0
1	1	3	3	1	1	1	1	1	1
0	0	0	0	0	0	1	1	1	1
0	0	0	0	0	0	0	0	0	0
0	1	0	1	0	1	0	1	0	1

0	0	0	0	0	0	0	0	0	0
0	0	0	0	0	1	0	0	1	0
1	1	1	1	1	1	1	1	0	1
0	0	0	0	0	1	1	1	1	1
0	0	0	0	0	0	0	0	0	0
0	0	0	0	0	0	0	0	0	0
1	1	1	0	1	1	0	0	1	1
0	0	0	0	1	1	0	0	1	1
1	0	0	0	0	0	0	0	0	0
2	2	2	0	2	1	1	0	2	1
2	2	2	0	2	2	2	0	2	2
0	1	0	1	0	1	0	1	0	1
1	1	0	0	1	1	3	0	0	0
0	0	0	2	0	0	0	2	0	0
0	0	0	2	0	0	0	3	0	0
0	0	3	1	3	0	1	0	3	1
0	0	0	3	0	0	3	3	0	0
0	0	0	1	0	0	0	3	0	0
0	0	0	3	0	0	0	3	0	0
0	0	0	3	0	0	1	1	1	0
1	0	0	0	0	0	0	1	0	0
1	0	0	0	1	1	1	1	1	1
0	0	0	0	1	1	1	1	3	0
1	1	1	1	1	1	1	1	0	0
0	0	1	0	2	2	0	1	0	0
1	1	0	0	0	0	1	3	0	1
2	0	3	0	2	3	0	0	2	3
0	1	2	0	1	1	0	0	0	1
0	0	0	0	0	0	1	3	0	0
0	0	0	2	0	0	0	2	0	0
0	1	0	3	3	0	3	0	3	0
0	0	0	0	0	0	0	0	0	0
0	0	0	0	0	0	0	0	0	0
0	0	0	0	0	0	0	0	0	0
0	0	0	0	0	0	0	1	0	0
1	1	1	1	0	0	0	0	0	0
0	0	0	0	0	0	0	3	1	0
0	0	0	0	0	0	0	1	0	0
0	0	0	0	0	0	0	0	0	0
0	0	0	0	0	0	0	0	0	0
0	0	0	0	0	0	0	0	0	0
0	0	1	0	1	1	1	0	0	0
0	0	0	0	0	0	0	0	0	0
0	0	0	0	0	0	0	0	0	0
0	0	0	1	0	0	0	0	0	0
0	1	0	0	0	0	0	0	0	0
0	0	0	0	0	0	0	0	0	0

0	0	0	0	0	0	0	0	0	0
0	0	0	0	0	0	0	0	0	0
0	1	1	0	0	1	3	0	1	1
2	0	1	3	0	1	1	0	0	0
0	0	1	1	1	1	1	1	1	1
0	0	0	0	0	0	0	0	0	0
0	0	0	0	0	0	0	0	0	0
0	0	0	0	0	0	0	0	0	0
0	0	0	0	0	0	0	0	0	0
0	0	0	0	0	0	0	0	0	0
0	0	0	0	0	0	0	0	0	0
1	1	1	1	1	1	1	1	1	1
0	0	1	0	0	0	0	0	0	0
0	0	0	3	0	0	0	3	0	0
0	0	0	1	0	2	0	1	1	0
0	0	0	2	0	0	2	3	0	0
3	0	3	0	3	3	3	3	3	0
3	0	0	0	3	0	1	3	0	0
2	0	1	3	1	3	3	0	1	1
0	0	0	0	0	0	0	0	0	0
0	0	0	3	0	0	2	3	0	0
0	0	2	1	0	0	2	2	0	0
0	0	0	0	0	0	0	0	0	0
0	0	0	0	0	0	0	0	0	0
0	0	0	3	3	0	0	3	2	0
0	0	0	0	0	0	0	0	0	0
3	1	0	3	1	0	2	2	1	2
0	0	0	3	0	0	0	0	0	0
0	0	0	3	0	0	1	3	0	0
0	1	1	2	1	1	0	2	1	1
1	0	0	0	0	0	0	1	0	0
0	0	0	0	0	0	0	0	0	0
0	0	0	0	0	0	0	0	0	0
0	0	0	3	0	0	0	3	0	0
0	2	0	0	0	0	3	3	1	0
0	0	1	1	0	0	2	3	0	0
0	0	0	3	0	0	3	3	2	0
1	1	0	0	1	1	1	1	1	1
0	0	0	0	0	0	0	0	0	0
0	0	0	3	0	0	0	3	0	0
0	0	0	3	0	1	0	3	0	0
0	0	0	3	0	0	0	3	0	0
1	1	1	3	1	1	1	3	1	1
0	0	1	1	1	1	1	1	0	0
1	2	2	2	2	2	2	1	1	1
0	0	0	3	0	0	3	3	0	0
0	0	0	3	0	0	3	3	0	0

0	0	0	3	0	0	3	3	0	0
0	0	0	3	0	0	0	3	0	0
0	0	0	3	0	0	3	3	0	0
0	0	0	3	1	0	3	3	0	0
0	0	0	3	0	0	3	3	0	1
0	0	0	3	0	0	3	3	0	0
0	0	0	3	0	0	3	3	0	0
0	0	0	3	0	0	3	3	0	0
0	0	0	3	0	0	3	3	0	0
0	0	0	3	0	0	3	3	0	0
0	0	1	2	2	1	1	1	2	1
1	0	0	3	0	0	3	3	0	0
0	0	0	3	0	0	3	3	0	0
0	0	0	3	0	0	3	3	0	0
0	0	0	2	0	0	3	2	0	1
0	0	0	3	0	0	3	3	0	0
0	0	0	2	0	1	0	1	2	0
0	0	0	3	0	0	3	3	0	0
0	0	0	3	0	0	3	3	0	0
0	0	0	2	0	0	2	2	0	0
0	0	0	3	0	0	3	3	0	0
0	0	0	3	0	0	3	3	0	0
1	1	1	2	1	1	2	3	1	0
0	0	0	0	0	0	3	1	0	0
0	0	0	2	0	0	3	3	0	0
1	2	1	2	1	1	3	3	1	0
1	0	0	1	0	2	0	3	0	0
0	0	0	3	0	0	3	3	0	0
0	0	0	3	0	0	3	3	1	0
0	0	0	3	0	0	2	3	1	0
0	0	0	3	0	1	3	3	0	0
0	0	0	3	0	0	3	3	0	0
1	1	1	2	1	1	1	1	0	1
1	0	0	1	0	0	1	1	3	0
0	0	0	3	0	0	3	3	0	0
0	0	0	3	0	0	3	3	0	0
0	0	0	3	0	0	3	3	0	0
0	0	0	3	0	0	3	3	0	0
0	0	0	3	0	0	3	3	0	0
1	1	1	3	0	0	3	3	0	0
0	0	0	3	0	0	3	3	0	0
1	1	1	2	2	1	2	1	0	1
0	0	0	3	0	0	3	3	0	0
0	0	0	0	1	1	3	3	0	0
0	0	0	3	0	0	3	3	0	0
0	0	0	0	0	0	0	0	0	0
0	0	0	0	0	0	0	0	0	0

0	0	0	0	0	0	0	0	0	0
0	0	0	0	0	0	0	0	0	0
3	3	3	3	3	3	3	3	3	3
0	0	0	0	0	0	0	0	0	0
0	0	0	3	0	0	0	0	0	0
0	0	0	3	0	0	0	0	0	0
1	1	1	3	1	1	3	1	1	1
0	0	0	0	0	0	0	0	0	0
0	0	0	0	0	0	0	0	0	0
0	0	0	0	0	0	0	0	0	0
0	0	0	0	0	0	0	0	0	0
0	0	0	0	0	0	0	0	0	0
0	1	1	0	0	1	0	1	1	0
0	0	0	0	0	1	1	1	1	3
1	1	0	0	0	0	0	0	0	0
0	0	0	0	0	0	0	0	0	0
1	1	0	0	0	0	1	1	1	1
0	0	0	0	0	0	0	0	1	1
0	0	0	0	0	0	0	0	0	0
0	0	0	0	0	0	0	0	0	0
0	0	0	3	0	0	0	3	0	0
0	0	0	0	0	0	0	0	0	0
0	0	0	0	0	0	0	0	0	0
0	1	1	2	1	1	1	2	0	0
0	1	0	0	2	3	1	0	3	0
0	0	0	3	0	0	0	3	0	0
0	0	0	3	0	0	0	3	0	0
0	0	0	3	0	0	0	3	3	0
0	0	0	3	0	0	0	3	0	0
0	0	0	1	0	0	1	2	1	1
0	0	0	1	0	0	0	1	0	0
0	1	1	1	0	0	0	0	0	0
2	0	0	3	1	0	0	2	0	2
3	3	2	2	2	3	3	1	3	3
3	3	0	1	3	3	0	2	2	2
1	0	1	1	1	1	1	1	0	0
3	0	3	1	2	2	0	3	0	0
0	1	0	3	0	0	0	3	0	0
1	1	0	2	0	1	1	3	1	0
0	0	3	3	0	0	0	3	0	0
0	0	0	3	0	0	0	3	0	0
0	0	0	3	3	0	2	3	3	0
0	1	0	2	1	1	0	0	0	1
0	0	0	3	0	0	0	1	0	0
0	0	2	2	0	0	0	3	0	0
0	0	0	3	0	0	0	3	0	0
1	3	3	0	1	3	3	3	3	3

0	0	0	0	0	1	3	1	0	0
0	0	0	0	0	0	0	0	0	0
3	1	2	0	1	1	0	0	1	2
0	0	0	2	0	0	1	3	0	0
0	0	0	0	0	0	0	0	0	0
0	0	0	3	0	0	3	3	0	0
0	0	0	0	0	0	0	0	0	0
3	0	3	0	3	3	0	0	3	3
0	0	0	3	0	0	3	0	0	0
0	0	3	3	0	0	3	3	0	0
0	0	0	3	0	0	0	3	0	0
0	0	0	3	0	0	1	1	0	0
0	0	0	0	0	0	0	0	0	0
0	2	1	0	1	0	0	1	1	0
0	0	0	3	0	0	0	3	0	0
0	0	0	3	3	1	1	3	1	0
1	1	1	3	1	1	3	1	1	1
0	0	0	0	0	0	0	1	0	0
0	0	0	3	0	3	0	0	0	0
0	0	0	3	2	0	0	0	0	0
0	0	0	1	1	0	3	3	0	0
1	1	2	1	2	1	1	2	0	0
0	0	1	1	1	3	1	1	2	0
0	0	0	1	0	0	0	3	0	0
0	0	0	3	0	0	0	0	0	0
0	0	0	3	0	0	0	3	0	0
1	1	1	0	2	2	2	0	3	2
0	1	0	3	2	1	3	2	2	1
0	0	0	3	0	0	3	3	0	0
1	0	2	0	1	3	0	3	0	0
0	0	0	1	0	0	3	1	0	0
1	1	1	0	2	1	0	0	0	0
0	0	3	3	0	0	0	0	0	0
0	0	1	0	2	2	0	1	2	0
0	0	0	3	0	0	0	3	0	0
0	0	0	3	0	0	0	3	0	0
0	0	1	2	0	0	0	2	1	0
0	0	0	3	0	0	0	3	0	0
0	0	0	1	0	0	1	3	0	0
0	0	0	0	0	0	0	0	0	0
0	0	0	0	0	0	0	0	0	0
0	0	0	3	0	0	3	3	0	0
0	3	0	2	3	0	3	3	0	0
0	1	0	3	0	0	2	3	0	0
0	0	0	0	1	0	0	1	0	0
0	0	0	3	0	0	1	1	0	0
0	0	0	0	0	0	0	0	0	0

0	0	0	3	3	0	1	3	0	0
0	0	0	3	0	0	3	3	0	0
0	0	0	3	0	0	3	3	0	0
0	0	1	3	1	0	0	3	1	0
0	0	0	0	0	0	0	0	0	0
0	0	0	3	0	0	0	3	0	0
0	0	0	0	0	0	0	0	0	0
0	1	1	1	3	3	0	0	1	2
0	0	0	3	1	3	1	1	1	0
1	1	1	0	1	0	0	3	1	0
0	0	0	3	0	0	3	3	0	0
1	1	1	2	1	1	1	0	1	1
0	0	0	0	0	0	0	0	0	0
0	0	0	0	0	0	0	0	0	0
0	0	0	0	0	0	0	0	0	0
0	0	0	0	0	0	0	0	0	0
0	0	0	0	0	1	0	1	0	1
0	0	0	0	0	0	0	0	0	0
3	3	3	3	3	3	0	3	3	3
1	0	0	1	0	1	1	1	1	0
0	0	0	0	0	1	1	1	1	1
0	0	0	0	0	0	0	0	0	0
0	0	0	0	0	0	0	0	0	0
0	0	0	0	0	0	0	1	1	0
0	0	0	0	0	0	0	0	0	0
3	3	1	1	1	1	0	0	3	3
0	0	0	0	0	0	0	0	0	0
0	0	0	0	0	0	0	0	0	0
0	0	0	0	0	0	0	0	0	0
3	0	3	0	3	3	1	2	2	2
0	0	0	0	0	0	0	0	0	0
0	0	0	0	0	0	0	0	0	0
0	0	0	0	0	0	0	0	0	0
1	0	0	3	0	0	1	3	0	0
0	0	0	0	0	0	0	0	0	0
2	0	0	3	0	0	0	2	0	0
2	3	1	3	0	0	2	3	0	0
0	0	1	2	0	1	0	0	1	0
0	0	0	1	1	1	1	1	1	0
0	1	0	3	0	1	2	2	0	0
3	2	2	3	3	3	3	3	3	1
0	0	0	0	0	0	0	0	0	0
0	0	0	3	0	0	3	3	0	0
1	0	0	3	1	1	3	3	0	0
0	0	0	2	0	0	0	2	0	0
1	0	0	3	0	0	1	1	0	0
0	0	0	3	0	0	3	3	0	0

0	3	3	0	0	2	0	0	0	0
3	3	3	3	3	3	0	0	3	3
0	0	0	3	0	0	0	0	0	0
0	0	0	3	0	0	0	1	0	0
0	3	1	2	0	1	3	2	0	3
0	0	0	3	3	0	3	2	1	0
0	1	0	1	0	2	0	3	0	0
0	1	0	1	0	2	0	3	0	0
0	0	1	1	1	1	1	1	2	0
0	0	0	3	0	0	0	3	0	0
3	0	0	2	1	1	2	2	2	0
0	1	1	2	1	0	3	0	0	0
0	0	0	3	0	0	3	3	1	0
0	0	0	3	0	0	1	1	0	0
0	0	0	0	0	0	0	0	0	0
0	0	0	1	0	0	1	1	0	0
1	0	0	3	0	0	0	3	0	0
0	0	0	3	0	0	0	3	0	0
0	0	0	3	0	0	2	2	0	0
0	0	3	3	0	0	3	3	0	0
0	2	3	0	2	2	0	3	3	0
1	2	0	3	3	2	2	2	2	0
1	0	1	1	2	1	0	1	0	1
0	0	0	0	0	0	0	0	0	0
0	1	0	3	0	0	0	3	0	0
0	0	0	0	0	0	0	0	0	0
2	0	0	1	1	0	3	3	0	0
0	0	0	0	0	0	0	0	0	0
1	2	0	3	1	2	0	3	0	0
1	2	3	0	2	3	0	2	2	2
0	0	0	3	0	0	0	3	0	0
0	1	1	3	1	1	2	3	0	1
0	1	1	3	1	1	2	3	0	1
1	0	0	3	0	0	0	3	0	0
0	0	1	3	0	0	1	3	0	1
0	0	1	3	0	0	1	3	0	1
0	3	0	3	0	0	0	0	0	0
0	3	0	3	0	0	0	0	0	0
0	1	1	1	0	0	3	3	0	0
0	0	1	2	1	1	1	1	1	1
0	0	0	1	0	0	1	0	0	0
0	1	3	0	3	0	0	2	2	0
0	0	0	3	0	0	0	3	0	0
0	0	0	0	0	0	0	0	0	0
0	0	0	3	0	0	0	3	0	0
0	0	0	3	0	0	0	3	0	0
0	0	0	3	0	0	0	0	0	0

3	0	1	3	1	3	3	3	0	0
1	1	1	1	1	1	1	3	0	0
0	1	1	3	1	1	3	3	1	0
3	0	3	0	3	0	0	0	3	0
0	0	0	3	0	0	3	3	1	0
0	0	0	3	0	0	0	3	0	0
0	0	0	3	0	0	0	3	0	0
0	0	0	3	0	0	1	3	0	0
3	3	3	3	3	3	0	0	3	3
1	1	2	0	1	2	1	1	0	0
0	0	0	0	1	2	2	0	1	0
0	0	0	0	1	2	2	0	1	0
0	0	0	1	0	0	0	0	0	0
3	0	1	1	1	2	1	2	1	1
0	0	0	3	0	0	0	0	0	0
2	2	0	3	3	0	3	3	0	0
0	0	1	1	1	2	3	3	2	1
0	0	0	3	0	0	3	3	1	0
0	0	0	0	0	0	0	1	0	1
3	3	0	0	0	3	3	0	3	3
0	0	0	3	0	0	0	3	1	0
0	3	0	1	1	2	2	0	3	0
1	1	1	3	0	0	1	3	0	0
1	0	1	1	1	0	0	1	1	0
1	0	1	1	1	0	0	1	1	0
2	2	1	0	2	2	1	0	3	0
2	1	1	2	1	1	0	1	0	1
0	1	0	0	0	1	1	0	1	0
3	2	3	0	2	3	3	0	3	2
0	0	0	3	0	0	0	3	0	0
0	0	2	2	2	0	0	3	0	0
0	0	0	3	0	0	0	3	0	0
0	0	0	3	0	0	3	3	0	0
1	0	0	2	0	1	1	3	0	0
0	1	0	3	0	0	0	3	1	0
3	1	1	2	3	2	2	2	0	1
3	1	1	2	3	2	2	2	0	1
0	0	0	3	0	0	0	3	0	0
0	0	2	1	3	0	0	1	2	0
0	3	0	3	0	0	0	3	0	0
0	0	0	3	3	0	3	3	0	0
0	0	0	0	1	0	0	3	0	0
2	3	3	0	0	3	0	3	0	0
3	2	3	1	0	0	1	2	0	0
0	0	0	1	1	0	0	1	0	0
0	3	0	1	2	0	2	2	1	0
0	3	0	0	0	0	0	3	0	0

0	0	0	3	0	0	0	3	2	0
0	0	0	3	0	0	0	3	0	0
3	0	0	1	3	0	0	3	0	0
1	1	0	1	0	0	0	0	1	1
0	0	0	0	0	0	0	0	0	0
0	0	0	0	0	0	0	0	0	0
0	0	0	0	0	0	0	0	0	0
1	1	0	3	1	1	1	1	0	0
0	0	0	1	1	1	1	0	0	0
0	0	0	0	0	0	0	0	1	0
1	1	1	1	1	1	1	1	1	1
1	1	1	1	1	1	1	1	1	1
1	1	1	1	1	1	1	1	1	1
1	1	1	1	1	1	1	1	1	1
1	1	1	1	1	1	1	1	1	1
0	0	1	0	1	0	1	0	0	0
2	0	1	1	1	1	1	1	1	1
0	1	0	1	0	0	1	1	0	1
1	1	1	1	1	0	0	0	0	1
0	0	0	1	1	1	0	1	3	1
0	0	0	0	0	0	0	0	0	0
0	0	0	1	1	1	1	1	0	1
0	0	0	0	1	0	1	0	0	0
1	1	1	1	1	0	0	0	0	0
0	0	0	0	0	0	0	0	0	0
0	0	0	0	0	0	0	0	0	0
0	1	0	1	0	1	0	0	0	0
1	1	1	1	1	1	1	1	1	1
0	0	0	0	0	0	0	0	0	0
0	0	1	3	0	0	0	0	0	0
0	1	1	1	1	1	1	1	1	1
3	1	0	0	1	1	0	0	0	0
1	1	0	0	1	1	1	1	0	0
0	0	0	0	0	0	0	0	0	0
0	0	0	0	0	0	0	0	0	0
0	0	0	0	0	0	0	0	0	0
0	1	0	1	1	0	0	0	0	0
3	2	0	0	0	0	0	0	0	0
0	0	0	3	0	0	0	3	0	0
1	1	1	1	1	1	1	1	1	1
0	0	0	0	0	0	0	0	0	0
0	0	0	0	0	0	0	0	0	0
0	0	0	0	0	0	0	0	0	0
1	1	1	0	0	1	1	0	1	0
0	0	0	0	0	0	0	0	1	0
3	1	1	1	1	1	0	0	0	0
0	0	0	0	0	0	0	0	0	0

0	0	0	1	0	0	0	1	0	0
0	2	2	0	2	1	1	0	1	1
0	0	1	1	1	1	1	1	1	1
0	0	0	0	0	0	0	1	1	1
0	0	0	0	0	0	0	0	0	0
0	0	0	0	0	0	0	0	0	0
1	1	1	0	0	1	0	0	0	0
3	3	0	0	0	0	1	0	0	1
1	1	1	0	0	0	0	0	0	0
3	0	3	0	2	3	2	1	0	2
0	0	0	1	0	1	0	2	3	0
0	0	1	1	2	3	0	3	1	2
2	0	0	1	0	2	0	0	0	0
1	1	1	1	1	1	1	1	0	1
2	0	2	0	2	3	0	2	0	2
0	0	0	0	1	3	0	0	1	0
0	0	1	1	1	0	1	3	1	0
0	0	2	2	0	2	0	0	1	2
0	3	1	0	0	3	0	0	3	3
0	0	0	3	0	0	0	3	0	0
0	1	1	1	1	0	1	3	0	0
1	1	1	1	0	0	0	3	0	0
0	0	0	3	0	0	0	0	0	0
0	0	0	2	2	0	0	3	0	0
0	0	0	2	0	0	0	2	0	0
0	0	0	3	0	0	0	1	0	0
0	0	0	3	1	0	0	3	0	1
1	0	1	1	3	3	0	1	3	0
3	0	1	0	1	3	1	1	1	0
1	0	3	1	1	1	1	1	1	1
0	0	1	1	1	3	0	1	3	0
1	0	0	0	0	0	0	0	0	0
1	0	0	0	0	0	0	0	0	0
3	1	3	0	2	3	1	2	0	0
0	0	0	1	0	0	0	0	0	0
0	0	0	3	3	3	1	2	1	0
0	0	0	1	0	3	0	3	1	0
0	0	0	3	0	0	3	3	0	0
0	0	0	3	0	0	3	3	0	0
0	0	0	2	0	0	0	2	0	0
0	0	0	2	0	0	0	0	0	0
2	0	2	0	3	2	0	2	0	0
3	0	0	3	1	0	0	0	1	3
0	0	0	2	2	2	0	2	0	0
0	0	0	3	0	0	3	2	0	0
0	1	1	3	1	1	3	3	0	3
0	0	0	2	3	0	0	0	0	2

0	0	0	0	0	0	0	0	0	0
0	0	0	3	1	1	0	1	2	0
0	0	0	0	3	0	0	3	0	0
0	0	0	1	0	0	1	1	1	1
0	0	0	0	0	0	0	0	0	0
0	0	0	0	0	0	0	0	0	0
0	0	0	0	0	0	0	0	0	0
0	0	0	0	0	0	0	0	0	0
0	1	1	1	1	1	1	3	0	1
0	0	0	0	0	0	0	3	0	0
0	0	0	0	0	0	0	0	0	0
0	0	0	0	0	0	0	0	0	0
1	0	1	1	1	1	1	1	1	1
1	1	1	1	1	1	1	1	1	1
0	1	0	0	1	2	1	3	1	0
0	0	0	0	0	0	0	3	0	0
0	0	0	3	0	1	1	3	0	0
0	0	0	1	0	0	0	0	0	0
1	1	1	0	0	1	0	1	1	1
1	1	1	0	0	1	0	1	1	1
1	1	1	0	0	0	0	0	0	0
1	1	1	1	1	1	1	1	1	1
0	0	0	0	0	0	0	0	0	0
1	3	1	1	3	1	0	1	1	1
0	0	0	0	0	0	0	0	0	0
0	1	0	0	0	0	0	0	0	0
0	0	0	0	0	0	0	0	0	0
0	0	0	1	0	0	0	1	0	0
0	1	1	1	1	1	1	1	1	1
1	1	3	3	1	1	0	0	0	1
1	1	1	1	1	1	0	0	1	1
0	0	0	3	0	0	1	0	1	1
0	0	0	0	0	0	0	1	0	0
0	1	0	1	1	2	0	3	0	3
0	0	0	1	1	1	1	1	1	1
0	0	0	3	0	0	2	0	0	0
0	0	0	0	0	0	0	0	0	0
0	1	0	0	0	0	0	0	0	0
0	0	0	0	0	0	0	0	0	0
0	0	0	1	0	0	0	1	0	1
0	0	0	1	0	0	0	1	0	1
0	0	0	0	0	1	0	0	0	0
0	0	0	1	0	0	0	1	0	0
0	0	0	0	0	0	0	0	0	0
0	0	0	0	0	0	0	0	0	0
0	0	0	0	0	0	0	0	0	0
0	0	0	0	0	0	0	0	0	0

0	0	1	1	1	1	1	1	1	1
0	0	1	1	1	1	1	1	1	1
0	0	0	0	1	0	1	3	0	0
0	0	0	0	0	0	1	3	3	0
0	0	0	1	0	0	1	1	0	0
0	0	0	0	0	0	0	0	0	0
0	0	0	0	0	0	0	0	0	0
0	0	0	0	0	0	0	0	0	0
0	0	0	1	0	0	0	1	0	0
1	0	0	1	0	1	0	1	0	1
0	0	0	1	3	0	3	1	0	0
0	0	0	1	1	0	1	1	0	0
1	1	1	1	1	1	0	0	0	0
1	1	1	1	1	1	1	1	1	1
1	1	1	1	1	1	1	1	1	1
1	0	0	0	1	0	0	0	0	0
0	0	0	3	0	0	0	3	0	0
0	0	0	3	2	0	3	3	1	1
1	1	1	2	2	1	2	3	2	2
0	0	0	0	0	0	0	1	0	0
0	0	1	0	0	1	0	0	0	0
0	0	0	3	0	0	0	3	0	0
0	0	0	0	0	0	1	0	0	1
0	0	0	3	0	0	1	3	2	0
1	1	1	1	1	1	1	1	1	1
0	2	0	3	2	0	0	3	3	0
0	0	1	3	1	0	0	3	0	0
0	0	0	0	0	0	0	0	0	0
0	0	0	0	0	1	0	0	0	0
0	0	0	3	0	0	3	3	0	0
0	1	0	3	1	2	3	3	1	0
0	0	0	3	0	0	0	0	0	0
1	1	1	1	1	1	0	1	1	0
1	0	1	0	2	0	2	3	0	0
1	1	1	1	3	1	1	1	1	0
0	0	0	3	0	0	0	3	2	0
0	0	0	3	0	0	0	3	0	0
1	0	0	0	1	0	3	3	0	1
0	1	0	2	0	0	2	3	0	0
0	0	0	0	0	0	1	3	1	0
1	0	3	0	0	0	1	0	0	1
0	0	0	3	0	0	0	3	0	0
0	0	0	3	0	0	0	3	0	0
0	0	0	3	0	0	2	3	0	0
0	0	0	3	0	0	0	3	0	0
0	0	0	0	0	0	0	0	0	0
0	0	0	2	0	0	0	3	0	0

0	0	0	2	0	0	0	3	0	0
3	3	1	1	1	3	0	0	3	0
1	1	1	1	1	1	1	2	0	1
0	1	1	1	1	1	0	1	1	1
0	0	0	3	0	0	0	0	0	0
0	0	0	0	0	0	3	3	0	0
0	0	0	0	0	0	0	0	0	0
0	0	0	0	0	0	0	0	0	0
0	1	1	3	0	2	2	3	2	1
1	1	1	0	1	1	1	3	0	1
0	3	0	1	3	2	0	2	0	3
1	0	0	3	1	1	1	3	1	0
0	0	0	0	0	0	0	0	0	0
1	1	0	0	0	0	0	1	0	0
1	1	1	1	1	0	1	1	1	1
0	0	0	0	0	0	0	0	0	0
0	0	0	0	0	0	0	0	0	0
0	0	0	3	0	0	0	3	0	0
2	0	2	3	1	2	0	3	3	0
2	0	0	2	2	2	2	0	3	2
1	3	0	2	0	2	1	0	1	1
3	0	0	0	3	0	3	1	2	2
0	0	0	3	0	0	0	3	0	0
0	0	0	0	0	0	0	0	0	0
0	0	0	1	1	1	1	0	0	1
1	1	2	3	1	1	1	3	1	0
0	0	0	1	0	0	3	1	0	0
0	2	3	3	0	0	0	3	0	0
0	0	3	3	2	3	0	3	2	0
0	0	0	3	0	3	0	3	0	0
0	0	0	3	2	0	0	3	0	0
0	0	0	0	0	0	0	0	0	0
0	1	0	3	2	0	2	0	2	0
1	0	3	1	0	0	1	3	0	0
0	0	3	1	0	0	1	0	0	1
0	0	0	2	0	0	1	3	0	0
0	0	0	0	0	0	1	3	0	0
0	2	2	3	1	2	3	3	0	0
0	1	0	3	3	0	3	1	0	0
0	2	1	3	1	1	1	1	1	2
0	0	0	3	0	0	3	1	0	0
0	0	0	3	0	0	3	0	0	0
0	0	0	3	0	0	1	3	0	0
0	0	0	3	1	1	0	3	1	0
0	0	0	3	0	0	3	3	0	0
2	0	1	2	1	1	1	0	0	0
0	0	0	3	1	0	3	3	0	0

0	0	0	1	0	0	3	3	0	0
0	0	0	3	0	0	3	3	0	0
0	0	0	3	0	0	1	3	0	0
0	0	0	3	0	0	3	3	0	0
0	0	0	3	0	0	3	3	0	0
1	0	0	3	0	0	1	3	0	0
0	0	0	3	0	0	2	3	0	0
1	1	1	2	1	0	0	2	0	0
0	0	0	3	0	0	3	3	0	0
0	0	0	3	0	0	3	3	0	0
0	0	0	3	0	0	3	1	0	0
0	0	0	3	0	0	3	3	0	0
0	0	0	3	0	0	2	3	0	0
0	0	0	3	0	0	3	3	0	0
0	0	0	3	0	0	3	3	0	0
0	0	0	3	0	0	2	2	0	0
0	0	0	3	0	0	3	3	0	0
1	1	1	2	1	1	2	1	1	0
0	0	0	3	1	1	0	2	0	1
0	1	0	3	0	0	3	3	0	0
1	0	0	1	0	0	0	1	1	0
1	0	2	1	1	1	2	1	0	0
1	2	2	1	1	1	2	2	1	1
0	0	0	3	0	0	3	3	0	0
0	0	0	3	0	0	3	3	0	0
1	1	0	2	1	0	2	1	0	0
0	0	0	2	1	0	2	3	0	0
0	0	0	3	0	0	3	3	0	0
0	0	0	3	0	0	2	3	0	0
0	0	0	2	0	1	1	3	0	0
1	1	1	1	2	2	0	0	1	0
0	0	0	3	0	0	3	1	0	0
0	0	0	3	0	0	2	3	0	0
0	0	0	3	1	1	3	3	0	0
0	0	0	3	0	0	3	3	0	0
1	0	1	1	1	1	1	1	0	1
0	0	0	3	0	0	3	3	0	0
0	0	0	3	0	0	3	3	0	0
0	0	0	3	0	0	3	3	0	0
0	0	0	2	0	0	2	2	0	0
0	0	0	3	0	0	0	3	0	0
0	0	0	3	1	0	2	3	0	0
1	1	1	1	0	0	3	3	0	0
0	1	1	2	1	0	3	1	0	0
0	0	0	3	0	0	3	3	0	0
0	0	0	2	1	0	2	2	0	0
0	0	0	3	0	0	0	3	0	0

0	0	0	2	0	1	1	2	1	0
1	1	0	2	1	2	0	0	1	0
0	0	0	3	1	1	0	3	0	0
1	0	0	1	0	0	1	3	0	0
1	1	1	0	1	1	3	0	0	0
0	1	2	2	1	2	0	1	3	2
3	0	0	1	1	1	1	0	3	0
0	0	0	3	0	0	3	3	0	0
1	0	1	2	1	1	2	3	0	0
0	0	0	3	0	0	3	3	0	0
0	0	0	3	0	0	3	2	0	0
0	0	0	0	0	0	0	0	0	0
0	0	0	0	0	0	0	0	0	0
0	0	0	0	0	0	0	0	0	0
0	0	0	0	0	0	0	0	0	0
0	0	0	0	0	0	0	0	0	0
0	0	0	0	0	0	0	0	0	1
0	0	0	0	0	0	0	0	0	0
0	0	0	0	0	0	0	0	0	0
3	3	3	3	3	3	3	3	3	3
3	3	3	3	3	3	3	3	3	3
1	1	1	1	3	0	2	3	2	2
1	1	1	0	1	3	1	1	1	1
0	0	0	0	0	0	0	0	0	0
0	0	0	0	0	0	0	0	0	0
0	0	0	0	0	0	0	0	0	0
0	3	1	1	1	1	1	1	0	1
0	0	0	0	0	0	0	0	0	0
0	0	0	0	0	0	0	0	0	0
0	0	1	1	1	1	0	0	0	0
0	0	0	0	0	0	0	0	0	0
0	0	0	0	0	0	0	0	0	0
1	1	1	1	1	1	1	1	1	1
0	3	3	1	3	3	3	1	1	1
0	0	0	0	0	0	0	0	0	0
1	1	1	1	1	0	0	1	0	0
0	0	0	0	0	0	0	0	0	0
0	0	0	3	0	0	0	0	0	0
0	0	3	0	3	3	3	3	0	3
0	0	0	3	2	1	0	3	0	1
0	0	0	3	0	0	3	0	0	0
0	0	0	3	0	0	0	3	0	0
0	0	0	3	0	0	0	3	0	0
0	0	2	0	0	0	0	1	2	3
0	0	0	0	0	0	0	0	0	0
3	0	0	0	3	3	3	0	3	0
0	0	0	3	0	0	0	3	0	0

0	2	2	1	2	0	2	0	2	2
0	0	0	3	0	0	0	3	0	0
2	0	2	0	1	2	0	1	0	3
0	0	0	2	0	0	0	3	0	0
0	0	0	3	0	0	0	0	0	0
0	0	0	3	0	0	1	2	0	0
2	2	2	1	2	2	2	1	0	0
0	0	0	3	0	0	0	3	0	0
0	0	1	3	0	0	0	3	0	0
0	0	0	3	0	0	0	3	0	1
1	0	1	2	1	1	1	2	0	1
0	0	0	3	0	0	1	3	0	0
0	0	0	0	0	0	0	0	0	1
0	0	0	0	0	0	0	0	0	0
0	0	0	0	0	0	0	0	0	0
0	0	0	3	3	3	3	3	0	0
0	0	0	1	0	0	1	3	0	0
0	0	0	3	1	1	2	1	2	0
0	0	0	1	1	0	1	2	0	0
1	3	3	0	1	1	0	1	3	3
3	1	1	1	1	1	1	1	1	1
1	1	1	3	3	2	2	0	2	0
1	0	0	3	0	0	3	3	0	0
0	0	0	3	0	0	3	3	0	0
0	0	0	3	0	0	0	3	0	0
3	1	0	1	1	1	3	2	1	0
0	0	0	2	0	0	1	3	0	0
0	0	0	2	0	0	1	3	0	0
0	0	0	1	0	0	2	3	0	0
0	0	0	1	0	0	2	3	0	0
0	0	0	3	0	3	1	3	0	0
2	1	0	0	0	1	0	3	0	0
0	0	0	3	0	0	0	3	0	0
0	0	0	3	0	0	3	3	0	0
0	0	0	3	0	0	1	1	0	1
0	0	0	3	0	0	1	1	0	1
1	1	0	1	0	0	0	0	0	0
1	0	0	0	0	0	0	2	0	0
0	0	0	3	3	0	1	3	0	1
0	0	0	3	0	0	1	3	0	0
0	0	0	3	0	0	0	3	0	0
0	0	0	3	0	0	0	3	0	0
0	0	0	0	0	0	0	0	0	0
0	0	0	0	0	0	0	0	0	0
0	0	0	0	0	0	3	3	0	0
0	0	0	3	0	0	0	3	0	0
0	0	0	3	0	0	3	3	0	0

0	0	0	3	0	0	0	3	0	0
0	0	0	0	0	0	0	0	0	0
1	0	0	3	0	0	1	3	0	0
0	0	0	3	0	0	0	3	0	0
0	0	0	2	0	0	1	1	0	0
0	0	0	2	1	0	0	3	0	0
0	0	0	1	0	0	3	3	0	0
0	0	0	0	0	0	0	3	0	0
3	1	1	1	1	1	1	1	1	1
1	1	1	0	1	3	1	0	1	1
0	0	0	3	0	0	0	3	0	0
0	0	0	3	0	0	0	3	0	0
3	1	0	0	0	0	0	0	0	0
0	1	1	0	1	2	3	2	1	0
0	0	0	1	0	1	0	0	0	0
0	0	0	3	0	0	3	3	0	0
0	0	0	3	0	0	3	3	0	0
0	0	1	0	0	0	0	0	0	0
0	0	0	0	0	0	0	0	0	0
0	1	1	1	2	1	0	1	2	0
3	3	3	0	3	3	0	0	3	0
3	1	1	0	3	1	1	0	3	0
0	0	0	3	0	0	3	3	0	0
0	0	0	3	0	0	1	3	0	0
0	0	0	3	0	0	1	3	0	0
3	1	1	0	3	1	1	3	3	3
0	0	1	0	0	0	3	0	3	0
0	0	1	1	1	1	1	0	1	0
0	0	0	1	3	1	0	3	0	0
0	0	0	3	0	0	0	3	0	0
0	0	0	0	0	0	0	0	0	0
0	0	0	3	0	0	0	3	0	0
0	0	0	0	0	0	0	0	0	0
0	0	0	3	0	0	0	3	0	0
0	0	0	0	0	0	0	0	0	0
0	1	0	0	0	0	1	3	0	0
0	0	0	1	0	0	2	3	0	0
2	1	1	1	1	3	1	1	2	0
0	2	2	1	3	2	1	0	3	2
1	1	0	0	0	1	0	1	0	0
0	0	0	0	0	0	0	0	0	0
0	1	0	0	1	1	0	1	0	1
0	1	1	1	1	1	1	0	1	1
0	0	0	0	0	0	0	0	0	0
0	1	1	0	0	0	0	2	0	2
0	0	0	3	2	0	1	3	1	0
1	0	1	0	1	1	1	3	0	1

0	0	1	2	1	0	1	0	0	1
1	2	0	0	0	0	0	0	0	0
0	0	0	0	0	0	0	0	0	0
0	0	0	0	0	0	1	0	1	0
0	1	0	0	0	1	0	0	0	0
0	0	1	1	0	1	0	0	0	1
3	3	3	0	3	3	0	2	3	3
0	0	0	0	0	0	0	0	0	0
1	1	1	1	1	1	1	1	1	1
0	0	0	0	0	0	0	0	0	0
3	3	3	3	3	3	3	3	3	3
0	0	0	0	0	0	0	0	0	0
0	0	0	1	0	0	0	0	0	1
0	0	0	0	0	0	0	0	0	0
0	0	0	0	0	0	0	0	1	0
0	0	0	2	0	0	0	2	0	0
0	0	0	0	0	0	0	0	0	0
0	0	0	1	2	1	2	1	1	1
1	0	1	3	0	0	0	0	1	0
1	0	0	0	0	1	3	3	1	1
0	0	0	0	0	0	0	0	0	0
3	3	1	2	0	1	0	3	2	3
0	0	0	3	0	0	0	3	2	0
0	0	0	0	0	0	1	0	0	0
0	1	2	1	1	1	2	1	2	3
1	0	1	1	2	0	0	1	0	0
0	0	0	0	0	0	0	3	0	0
0	0	0	2	0	0	1	2	1	0
0	0	0	3	0	0	0	2	0	0
0	0	0	3	0	0	0	2	0	0
0	0	0	0	0	0	0	2	0	0
1	1	1	1	1	1	1	1	0	0
0	0	1	2	0	1	0	1	0	0
0	0	0	3	0	3	0	0	0	0
0	0	0	3	0	0	0	3	0	0
2	3	1	2	3	1	2	3	0	0
0	0	0	0	0	0	0	3	1	0
0	0	0	0	0	0	0	3	1	0
0	0	0	3	0	0	0	0	0	0
1	2	0	3	0	3	1	3	0	0
0	0	0	3	0	0	0	3	0	0
0	0	0	0	0	0	0	0	0	0
3	3	3	2	3	0	3	3	1	0
0	1	1	2	1	0	1	2	1	0
1	1	0	0	1	0	1	1	1	1
1	1	0	0	1	0	1	1	1	1
1	0	1	0	1	0	0	1	1	1

1	0	1	0	1	0	0	1	1	1
0	0	0	3	3	1	3	1	0	0
2	3	1	0	3	3	0	1	3	3
0	0	0	0	0	0	0	1	0	0
0	0	0	0	0	1	1	1	0	0
3	3	3	0	3	3	3	0	3	3
0	0	1	1	1	0	1	1	0	0
1	0	0	3	1	1	2	3	0	0
0	0	0	1	1	1	0	1	0	0
0	0	0	3	0	0	3	3	1	0
0	0	0	0	0	0	0	0	0	1
0	0	0	0	0	0	0	0	0	1
0	0	0	3	0	0	3	3	0	0
2	0	0	3	0	0	0	3	0	0
3	1	3	0	0	0	0	0	2	0
3	0	3	1	1	0	2	2	2	1
0	0	0	0	1	0	1	2	0	1
1	0	1	2	1	0	1	2	1	1
0	1	0	0	0	0	0	0	0	0
0	0	0	3	0	0	0	3	0	0
0	0	0	3	0	0	0	2	0	0
3	3	1	1	0	0	0	2	2	0
0	1	0	3	1	0	3	3	0	0
0	0	0	3	0	0	3	3	0	0
2	0	0	2	0	1	1	3	3	1
0	0	0	3	0	0	0	3	0	0
0	1	2	1	1	1	1	1	1	0
0	1	1	1	1	2	1	2	1	0
0	1	1	1	1	2	1	2	1	0
0	0	0	3	0	0	1	0	0	0
3	0	0	2	2	2	0	1	2	0
3	3	3	2	2	1	1	0	0	0
3	3	0	0	3	3	3	0	2	0
1	0	1	0	1	3	1	3	0	0
0	0	0	3	0	0	0	3	0	0
0	3	0	1	3	3	0	2	3	0
0	0	0	3	0	0	0	3	0	0
1	1	1	1	2	1	2	1	1	2
0	0	0	3	0	0	0	0	0	0
3	0	3	3	3	3	0	0	3	3
0	0	1	3	0	1	1	0	0	0
2	1	2	2	0	1	1	3	0	0
1	1	2	3	0	1	3	1	1	0
3	0	3	1	3	3	3	1	0	3
3	0	3	1	3	3	3	1	0	3
1	0	0	1	0	1	1	3	0	0
0	1	0	3	1	1	0	3	1	0

0	0	0	3	0	0	0	3	0	0
2	1	2	0	2	3	2	1	2	1
0	0	0	2	0	0	0	1	3	1
0	0	0	3	0	0	0	3	0	0
0	0	0	3	0	0	0	3	0	0
0	0	0	3	0	0	0	3	0	0
0	0	0	0	0	0	0	0	0	0
0	0	0	0	0	0	0	0	0	0
0	0	0	0	0	0	0	0	0	0
0	0	0	3	0	0	0	3	0	0
1	0	0	3	0	1	0	3	2	0
0	0	1	1	2	1	0	1	1	1
1	0	0	3	1	3	3	3	1	0
0	0	0	3	3	0	1	3	0	0
1	1	1	3	0	1	3	3	0	0
1	1	0	3	0	0	3	3	0	0
0	0	0	0	1	1	0	0	1	1
0	0	0	0	0	0	0	0	0	0
0	0	0	0	0	0	0	0	0	0
1	1	1	1	1	1	0	1	0	1
0	0	0	3	0	0	0	3	0	0
1	3	1	1	1	1	1	1	1	1
0	0	0	0	0	0	0	3	0	0
1	1	1	1	1	1	1	1	1	1
0	0	0	0	0	0	0	0	0	2
0	0	0	0	0	0	0	0	0	0
0	0	0	0	0	0	0	0	0	0
0	0	0	0	0	0	0	0	0	0
0	0	0	0	0	0	0	0	0	0
0	0	0	0	0	0	0	0	0	0
0	0	1	0	0	0	0	0	0	0
0	0	0	0	0	0	0	1	1	1
0	0	0	0	0	0	0	0	0	0
1	1	1	1	2	1	1	1	0	1
0	0	0	0	0	0	0	0	0	0
0	0	0	0	0	0	0	0	0	0
0	0	0	0	0	0	0	0	0	0
0	0	0	0	1	1	1	1	1	1
3	1	1	1	1	1	1	1	1	1
3	0	0	0	0	0	0	0	0	0
0	0	0	0	0	0	0	0	0	0
0	0	0	0	0	0	0	0	1	1
0	0	0	0	0	1	0	0	0	0
0	0	1	0	0	0	1	0	1	0
0	0	0	1	1	0	0	1	0	1
0	0	0	0	0	0	0	0	1	0
0	0	0	0	0	0	0	0	0	0

0	0	0	0	0	1	1	0	0	0
1	1	1	1	1	1	0	0	0	0
1	1	1	0	0	0	0	0	0	0
0	1	0	0	1	0	1	0	1	0
0	0	1	1	1	1	1	1	1	1
0	0	0	0	0	0	0	0	0	0
0	0	1	0	1	0	1	0	1	0
3	1	1	1	1	1	1	1	1	1
3	0	0	0	0	0	0	0	1	0
0	0	1	0	0	0	1	1	0	1
0	0	0	0	0	0	0	0	0	0
0	0	0	0	0	0	0	0	0	0
0	0	0	0	0	0	0	0	0	0
1	1	1	0	1	0	1	1	0	1
0	0	0	0	0	0	0	0	0	0
0	1	0	1	1	1	0	1	1	0
0	0	0	0	0	0	0	0	0	0
0	1	1	0	1	1	0	1	0	1
0	0	1	1	1	1	1	1	1	1
1	1	1	1	1	1	1	1	1	0
0	2	0	2	2	0	2	2	0	1
1	1	1	0	0	0	0	0	1	0
1	1	1	0	0	1	0	0	0	0
0	0	0	0	0	0	0	0	0	0
0	0	0	3	0	0	0	3	0	0
0	1	0	1	0	1	0	1	0	0
0	1	0	1	1	1	0	0	0	0
0	1	1	1	1	1	1	0	1	1
0	0	0	0	0	0	0	1	1	0
3	1	1	1	1	1	1	1	1	1
0	0	0	0	0	0	0	0	1	1
0	0	0	0	1	1	1	1	1	1
0	0	0	0	0	0	0	0	0	0
0	0	0	0	0	0	0	0	0	0
1	0	0	0	0	0	0	0	0	0
0	0	0	1	1	1	1	1	1	1
2	0	0	0	0	0	0	0	0	0
0	1	0	1	0	1	0	1	0	1
0	0	0	0	0	0	0	0	0	0
2	0	1	0	0	1	0	0	0	1
0	0	0	0	0	0	1	1	0	0
0	0	0	1	1	0	0	1	0	1
2	1	1	1	1	1	1	0	0	0
1	1	1	1	1	0	0	1	0	0
0	1	1	1	1	0	1	0	1	0
0	0	1	2	1	1	0	2	0	0
0	0	0	1	0	0	3	3	0	0

2	0	2	0	2	2	0	0	0	0
0	1	3	1	1	1	1	3	1	0
0	1	0	1	0	1	0	1	1	0
0	0	1	2	1	1	1	3	0	2
0	0	0	2	0	1	0	2	0	0
2	0	2	1	1	2	0	3	3	2
0	0	0	2	0	0	0	2	0	0
0	0	0	3	0	0	3	1	0	0
3	2	2	0	3	3	0	0	2	3
0	0	1	1	1	1	1	1	1	1
1	0	0	3	1	0	1	3	0	0
1	0	0	3	1	0	1	3	0	0
0	3	1	0	1	1	1	3	0	1
0	3	1	0	1	1	1	3	0	1
1	1	1	0	0	0	0	3	0	1
0	0	0	2	2	1	0	3	0	0
1	1	0	1	0	0	0	0	0	0
0	1	1	0	1	1	1	1	1	1
3	0	2	0	2	3	2	2	0	0
0	0	0	3	1	2	3	3	0	0
0	1	0	3	0	0	0	0	0	1
0	0	2	1	3	3	0	2	0	0
0	0	0	3	0	0	0	1	0	0
0	0	3	2	2	2	0	3	0	0
0	0	0	1	1	0	0	0	1	0
0	1	1	1	1	1	1	1	1	1
0	0	0	3	1	0	0	3	1	0
0	2	0	1	0	3	1	1	2	0
0	0	0	0	1	0	1	1	1	1
0	0	0	0	1	0	1	1	1	1
3	2	3	0	3	3	2	2	3	3
0	0	0	3	0	0	0	3	0	1
0	0	0	3	0	0	0	2	0	0
0	0	0	0	0	0	0	0	0	0
1	0	0	0	0	0	1	3	0	0
0	0	0	3	2	1	2	2	3	0
0	0	0	3	0	0	1	3	0	0
0	0	0	2	1	0	0	2	0	0
2	1	3	0	2	3	0	2	0	2
0	0	0	0	0	1	1	1	3	0
0	0	0	0	0	1	1	1	3	0
0	0	0	0	0	2	0	0	0	0
0	1	1	1	1	1	1	1	1	1
0	1	0	0	0	0	0	0	0	0
0	0	0	0	0	0	0	0	0	0
0	0	0	1	0	0	0	1	0	0
0	1	0	0	0	0	0	0	0	0

1	1	1	1	1	1	1	1	1	1
0	0	0	0	0	0	0	1	0	0
1	1	1	0	3	1	0	1	1	3
0	0	0	0	0	0	0	1	0	0
0	0	0	0	0	0	0	1	0	0
1	1	0	0	1	3	1	1	1	0
0	0	0	0	0	0	0	1	0	0
0	0	0	0	0	0	0	0	0	0
0	0	0	0	0	0	0	3	1	0
0	0	0	3	0	0	0	0	0	0
0	0	0	3	0	0	0	0	0	0
0	0	0	0	0	0	0	0	0	0
0	0	0	0	0	0	0	0	0	0
1	1	3	3	3	3	1	0	3	0
1	0	0	1	0	0	1	1	1	1
1	0	0	1	0	0	1	1	1	1
0	0	0	1	0	0	0	1	0	0
0	0	0	0	0	0	0	0	0	0
0	0	0	0	0	0	0	0	0	0
0	0	0	1	0	0	0	1	1	1
0	1	0	1	1	0	1	3	0	0
0	0	0	0	0	0	0	0	0	0
0	0	0	0	0	0	0	0	0	0
1	1	0	1	0	1	1	0	1	1
1	1	1	1	0	0	1	1	0	0
1	1	1	1	0	0	1	1	0	0
0	0	0	3	0	0	0	1	0	1
0	0	0	0	0	0	0	0	0	0
1	1	1	0	0	1	0	1	0	0
0	0	0	1	1	0	1	1	0	0
0	0	0	1	0	0	0	3	0	0
0	0	0	1	0	0	1	1	0	0
0	0	0	0	0	0	0	1	0	0
0	0	0	0	0	0	0	0	0	0
0	0	0	3	0	1	0	3	0	0
0	0	0	3	0	0	0	3	0	2
0	0	0	0	0	0	0	0	0	0
0	0	0	3	0	0	0	3	0	0
0	0	0	2	0	0	2	2	0	0
0	0	0	2	0	0	2	2	0	0
0	0	0	2	3	0	2	2	0	0
0	0	0	3	0	0	0	3	0	0
0	1	0	3	0	0	0	3	0	0
2	1	1	2	2	2	1	3	0	2
0	1	1	1	0	0	0	0	0	1
0	1	1	1	0	0	0	0	0	1
0	0	0	1	0	0	0	0	1	1

2	0	2	2	2	2	0	2	2	0
0	1	0	3	3	3	1	3	3	0
0	0	0	0	0	0	0	0	0	0
0	0	0	0	1	1	1	1	1	1
0	1	1	1	1	1	1	1	1	1
0	1	3	3	1	3	2	1	0	1
0	0	0	3	0	0	0	3	0	0
0	1	0	0	0	0	0	0	1	1
3	0	0	0	3	3	3	1	3	2
2	2	2	0	2	2	2	0	2	2
3	3	3	2	3	3	3	2	3	3
0	0	0	0	0	0	0	0	0	0
1	1	0	3	0	1	2	3	0	1
3	3	3	0	3	3	0	0	3	0
0	1	1	1	1	3	1	1	1	1
0	0	0	3	0	0	0	3	0	0
0	1	1	0	0	0	0	0	0	0
1	3	1	3	1	1	3	3	3	3
0	0	0	1	0	0	0	0	0	0
0	0	0	3	1	1	0	2	0	0
0	0	0	3	0	0	0	3	0	0
0	0	0	3	3	3	0	3	3	0
0	0	0	3	3	3	0	3	3	0
0	1	2	3	3	1	0	3	1	1
0	1	2	3	3	1	0	3	1	1
0	0	0	0	0	0	0	1	0	0
0	1	0	3	1	0	0	3	1	0
1	2	1	0	2	3	3	0	3	2
1	0	1	3	1	1	0	0	0	1
1	0	1	3	1	1	0	0	0	1
1	3	1	0	0	3	0	0	0	1
1	3	1	0	0	3	0	0	0	1
0	0	0	3	1	0	2	3	0	0
1	0	0	0	0	0	0	3	1	3
1	1	1	1	0	0	0	3	0	0
0	0	0	3	2	1	0	0	2	0
0	1	1	1	1	1	1	1	1	1
0	1	0	0	1	0	0	0	1	0
1	0	0	0	0	0	0	0	0	0
0	0	1	3	0	1	1	3	0	0
3	3	1	0	1	3	0	0	2	0
0	0	0	3	0	3	3	3	0	0
0	0	0	3	0	0	3	3	0	0
1	0	0	2	1	1	2	3	0	0
0	0	1	3	0	0	0	2	0	0
1	1	1	2	1	1	2	3	0	0
0	0	0	3	0	0	3	3	0	0

0	0	0	3	0	0	3	3	0	0
1	1	2	2	1	0	2	3	1	0
3	3	3	0	3	3	0	0	3	0
0	0	1	0	2	2	0	1	2	3
0	0	0	3	0	0	3	2	0	0
0	0	0	3	0	0	3	3	0	0
0	0	0	3	0	1	3	1	0	0
0	0	0	3	0	0	3	3	0	0
0	0	0	3	0	0	3	3	0	0
1	2	2	0	2	1	0	0	3	0
0	0	0	3	0	0	2	3	0	0
3	1	0	2	1	1	1	1	1	1
1	0	1	2	1	0	2	3	0	0
0	0	0	3	0	0	3	1	0	0
0	0	0	2	0	0	0	2	0	0
1	0	1	1	0	0	3	3	0	0
1	1	1	1	2	1	3	2	0	1
0	0	0	3	0	0	3	3	0	0
0	0	0	3	0	0	3	3	0	0
0	0	0	3	0	0	3	3	0	0
0	0	0	2	0	0	3	3	0	1
0	0	0	2	1	0	0	3	1	0
3	0	1	1	1	3	1	1	1	0
1	1	1	2	0	0	2	2	0	0
1	1	0	3	0	0	3	3	0	0
1	1	0	2	1	0	2	3	0	0
0	0	0	3	0	0	3	3	0	0
0	0	0	1	0	1	3	3	0	0
0	0	0	2	0	0	3	3	0	0
0	0	0	3	0	0	3	3	0	0
1	0	1	2	1	0	2	3	0	0
0	1	0	0	0	0	3	2	1	0
0	0	0	1	1	1	1	3	0	0
1	1	0	3	1	1	3	1	0	0
0	0	0	3	0	0	3	3	0	0
0	0	0	3	0	0	3	3	0	0
3	2	2	1	1	2	0	0	3	1
0	0	0	3	0	0	3	3	0	0
0	0	1	2	1	1	2	2	0	1
0	0	0	2	1	1	1	0	2	0
0	0	0	3	0	0	3	3	0	0
0	1	1	1	0	0	1	2	1	0
1	0	1	2	1	0	2	3	0	0
0	0	0	3	0	0	3	3	0	0
0	0	0	3	0	0	3	3	0	0
0	0	0	3	0	0	3	3	0	0
0	1	1	1	0	1	1	1	1	1

0	0	1	2	1	1	1	2	1	0
0	0	0	0	0	0	0	0	0	0
1	1	1	3	1	1	1	3	1	0
0	1	2	1	0	1	1	0	0	0
0	0	0	0	0	0	0	0	0	0
0	0	0	0	0	0	0	0	0	0
0	0	0	0	0	0	0	0	1	1
0	0	0	0	0	0	0	0	0	0
0	0	0	0	0	0	0	0	0	0
0	0	0	1	0	0	1	0	1	1
0	0	0	0	0	0	0	0	0	0
0	0	0	0	0	0	0	0	1	1
0	0	0	0	0	0	0	0	0	0
0	0	0	0	0	1	1	0	0	0
0	0	0	0	0	0	0	0	0	0
0	0	0	0	0	0	0	0	0	0
0	0	0	0	0	0	0	0	0	0
0	0	0	0	0	0	0	0	0	0
3	1	1	1	1	3	1	3	3	3
3	3	3	3	3	3	3	3	3	3
0	0	0	0	0	1	1	1	1	1
0	0	0	3	0	0	1	0	0	0
0	0	0	3	0	0	0	1	0	0
1	0	1	0	0	1	3	1	0	0
0	0	0	3	0	0	0	3	0	0
1	0	1	2	0	0	1	3	0	0
0	0	1	1	0	1	0	2	1	0
1	0	1	1	1	1	0	1	1	1
1	1	1	0	1	1	1	0	0	1
0	1	1	2	1	1	1	1	2	1
1	0	0	1	1	0	0	3	0	1
0	0	0	1	1	0	1	3	0	0
0	0	2	2	0	0	0	3	0	0
0	0	3	0	1	3	2	1	1	0
0	0	0	3	0	0	0	3	0	0
0	1	1	2	0	0	0	3	0	0
1	0	0	3	0	0	0	3	0	0
1	0	0	3	0	0	0	2	0	0
0	0	0	3	0	0	1	3	0	0
2	0	2	1	2	2	0	0	0	2
0	0	0	1	0	0	1	3	0	0
1	1	1	2	0	1	0	2	0	1
0	0	0	3	0	0	0	3	0	2
0	0	0	3	0	0	0	3	0	0
0	0	0	3	0	0	0	3	0	0
0	0	0	3	0	0	0	3	0	0
0	0	0	3	0	0	0	3	0	0

0	0	0	0	2	3	3	0	3	3
0	0	0	3	0	0	0	3	0	0
0	1	0	0	0	0	0	2	1	0
1	0	0	0	0	0	3	3	0	0
1	0	0	0	0	0	3	3	0	0
0	0	1	1	0	0	3	3	0	3
1	1	1	3	2	3	1	1	2	1
0	0	0	3	0	0	0	3	0	0
0	0	2	0	2	2	0	0	0	0
1	0	0	3	0	0	3	3	0	0
0	0	0	0	0	0	0	0	0	0
0	0	0	2	0	0	1	3	0	0
0	0	0	2	0	0	1	3	0	0
0	0	0	1	1	0	1	2	0	0
0	0	0	0	0	0	0	0	0	0
0	1	0	3	0	0	3	1	0	0
0	0	0	0	0	0	0	0	0	0
0	0	0	3	0	0	1	3	0	0
3	0	3	0	3	3	0	0	0	0
0	0	0	3	0	0	3	3	0	0
0	0	0	0	1	0	1	2	0	0
0	0	0	1	3	0	2	2	0	0
0	0	0	0	0	0	0	0	0	0
0	1	0	3	0	0	0	3	0	0
0	0	0	3	0	0	1	3	0	0
0	0	0	3	0	0	0	3	0	0
0	0	1	0	1	1	1	2	1	0
0	0	0	0	0	0	0	0	0	0
0	0	0	3	0	0	1	1	0	0
0	0	0	3	0	0	0	3	0	0
0	0	0	3	0	0	0	3	0	0
0	0	0	0	0	0	0	0	0	0
0	0	0	3	0	0	0	0	0	0
0	0	0	0	0	0	3	3	0	0
0	0	0	0	0	1	0	3	0	0
0	0	0	0	0	0	0	3	0	0
1	3	2	1	1	3	1	0	2	2
0	0	0	1	0	0	1	3	0	0
0	0	0	1	2	0	0	1	0	0
0	0	1	3	0	0	1	0	0	0
0	0	0	3	1	2	1	0	0	0
0	0	0	0	0	1	0	1	0	0
0	0	0	3	0	0	0	3	0	0
0	0	0	3	0	0	0	3	1	0
0	0	0	3	0	0	3	3	0	0
0	0	1	1	1	0	3	0	1	0
3	0	3	0	0	3	0	0	3	0

1	1	1	0	1	3	1	3	0	0
0	0	0	3	0	0	3	3	0	0
0	0	0	3	1	0	1	3	0	0
0	0	0	3	0	0	3	3	0	0
0	0	0	3	0	0	3	3	0	0
0	3	0	3	2	0	3	3	0	0
0	3	0	3	2	0	3	3	0	0
0	0	0	3	0	0	0	3	0	0
0	1	0	3	0	0	3	2	0	0
0	0	0	0	0	0	0	0	0	0
0	0	0	0	0	0	0	0	0	0
0	1	0	1	2	1	0	0	0	2
0	0	0	3	0	0	1	3	0	0
0	0	0	3	0	0	0	3	0	0
0	0	0	0	0	0	0	0	0	0
0	0	0	0	0	0	0	0	0	0
0	3	1	1	1	3	0	1	1	2
0	1	0	3	1	1	3	1	1	1
3	3	3	3	3	3	0	0	3	3
0	0	1	1	1	0	1	3	1	1
0	0	0	0	0	0	0	0	0	1
0	0	0	0	0	0	0	0	0	0
0	0	0	0	0	0	0	0	0	0
3	3	3	3	3	3	3	3	3	3
3	0	3	3	3	3	3	3	3	3
3	3	3	0	3	3	3	0	3	3
2	2	2	0	2	2	2	0	2	2
0	0	0	0	0	0	0	0	0	0
0	0	0	0	0	0	0	0	0	0
0	0	0	0	0	0	0	2	0	0
0	1	1	1	1	1	0	1	1	0
0	0	0	0	0	0	0	0	0	0
0	0	0	0	0	0	0	0	0	0
0	0	0	0	0	0	0	0	0	0
0	0	0	0	0	0	0	0	0	0
3	3	3	1	0	3	3	2	3	3
0	0	0	0	0	0	0	0	0	0
1	0	1	0	1	0	1	1	3	0
3	0	0	0	0	0	0	0	0	0
0	0	0	0	0	0	0	0	0	0
0	0	1	3	0	1	1	3	0	0
1	0	0	0	0	0	0	2	0	0
1	0	0	3	0	1	0	3	0	0
0	0	0	3	0	0	3	3	0	0
0	0	0	3	1	3	3	3	0	1
0	0	0	3	0	0	0	3	0	0
1	0	0	0	0	0	1	1	2	0

1	0	0	1	1	1	1	1	1	1
0	1	0	3	0	0	1	3	0	0
0	0	0	2	0	0	1	3	1	2
2	0	0	3	0	0	0	2	0	0
0	1	0	1	0	1	0	1	2	1
2	2	1	2	1	3	0	0	2	2
0	0	0	0	0	0	0	0	0	0
1	0	2	1	0	1	1	2	1	0
0	0	0	3	0	0	0	3	0	0
1	0	0	1	1	1	0	1	1	1
0	0	1	3	3	3	2	0	2	0
0	0	3	2	0	0	0	0	3	0
1	0	0	3	1	3	3	3	3	0
0	0	0	1	0	0	0	0	0	0
1	1	2	1	0	0	1	0	1	0
0	0	0	3	0	0	0	3	0	0
0	2	1	1	1	1	3	3	0	0
0	0	0	3	0	0	0	3	0	0
3	3	2	0	3	3	3	1	3	2
0	0	0	3	0	0	2	1	1	0
0	0	0	0	0	0	2	3	0	0
0	0	0	1	1	1	0	0	0	1
0	0	0	3	0	0	0	0	0	0
0	0	0	3	2	3	2	3	0	0
1	0	1	0	1	0	0	1	1	1
0	3	0	3	0	2	2	1	0	3
0	0	0	0	0	0	0	0	0	0
0	0	0	3	0	0	0	3	0	0
1	2	2	2	0	0	0	1	0	0
1	1	1	1	1	1	0	2	1	0
2	0	0	3	0	0	1	3	1	0
3	3	3	0	3	3	3	0	3	3
0	1	2	3	1	2	3	1	2	1
0	0	0	3	0	0	0	3	0	0
1	1	2	3	2	0	0	1	1	1
3	0	3	0	3	2	0	0	3	0
0	0	0	0	0	0	0	0	0	0
3	0	1	3	2	3	0	0	3	0
0	3	3	0	0	0	0	0	0	0
0	2	1	3	0	0	3	1	2	1
0	0	0	0	0	0	0	0	0	0
2	0	0	3	0	0	0	3	0	0
2	1	1	0	1	2	2	0	1	1
3	1	0	0	0	0	0	0	0	0
0	0	0	0	0	0	0	0	0	0
0	0	0	0	0	0	0	0	0	0
0	1	0	0	1	0	1	0	1	0

0	0	0	0	0	0	0	1	0	1
0	0	0	0	0	0	0	0	0	0
0	0	0	0	0	0	0	0	0	1
0	0	0	0	0	0	0	0	0	0
0	0	0	0	0	0	0	0	0	0
0	0	0	0	0	0	0	0	0	0
0	0	0	0	1	0	0	1	1	1
1	1	1	1	1	1	1	1	1	1
0	0	0	0	0	0	0	0	0	0
0	0	0	0	0	0	0	0	0	0
1	1	1	1	0	0	1	0	0	0
0	0	0	0	0	1	0	0	1	0
0	0	0	0	0	0	0	0	0	0
0	0	0	0	0	0	1	0	1	0
0	0	0	0	0	1	1	1	1	1
0	0	0	0	1	0	0	0	0	0
0	0	0	2	0	0	0	2	0	0
0	0	0	0	1	1	0	1	0	1
3	1	1	1	1	2	2	0	0	0
0	0	0	0	0	0	0	0	0	0
0	0	0	0	0	0	0	0	0	0
0	0	0	0	1	0	1	0	1	0
0	0	0	0	0	0	0	1	1	1
0	0	0	0	0	0	0	0	0	0
0	1	0	1	1	0	0	0	0	0
0	0	0	0	0	0	0	0	0	0
0	0	0	0	0	0	0	0	0	0
0	0	0	0	0	0	0	0	3	1
2	2	2	0	3	3	2	1	2	2
0	0	0	0	0	0	0	0	0	0
0	0	0	0	0	0	0	0	0	0
1	1	0	0	0	0	0	0	0	0
2	0	0	0	0	0	0	0	0	0
0	0	1	1	1	1	1	1	1	1
0	0	0	0	0	0	0	0	1	0
0	0	0	1	3	1	0	0	0	0
0	0	0	0	0	0	0	1	0	0
1	1	1	1	1	1	0	0	0	0
1	1	0	0	0	0	0	0	0	0
0	0	0	0	0	0	0	0	0	0
3	0	3	0	3	2	3	0	2	2
3	0	1	1	1	1	0	1	1	0
0	0	1	0	1	3	0	1	3	3
3	0	3	0	2	3	0	1	0	0
0	1	1	1	2	3	1	1	2	0
0	1	1	1	2	3	1	1	2	0
1	1	0	1	0	1	1	1	3	3

1	1	0	1	0	1	1	1	3	3
0	1	0	0	0	0	0	3	1	1
0	1	0	0	0	0	0	3	1	1
0	0	0	3	0	0	0	0	0	0
0	0	0	3	0	0	0	0	0	0
0	0	0	1	1	0	0	3	0	0
1	0	0	1	3	0	3	0	1	0
0	1	1	1	3	0	0	0	1	0
0	0	2	1	1	2	3	1	3	3
0	1	0	2	1	3	1	3	1	0
1	0	0	0	0	0	0	3	0	0
0	0	0	1	1	3	1	2	1	0
0	0	0	2	2	0	0	3	0	0
0	0	2	0	2	3	0	0	3	3
0	0	0	0	1	3	1	1	3	1
0	0	0	3	2	2	0	3	0	0
1	3	1	0	1	2	0	0	3	1
0	0	0	1	1	3	1	1	0	0
0	0	0	0	3	3	1	0	3	0
0	1	1	1	2	0	1	0	1	0
0	2	0	0	0	0	1	1	0	0
1	1	1	0	3	2	3	0	2	3
1	0	1	1	1	1	0	2	0	1
1	1	0	1	1	0	1	1	0	1
0	0	0	0	0	0	0	0	0	0
0	1	1	1	1	1	1	2	0	0
0	0	0	3	0	0	0	1	1	0
0	0	0	1	0	0	0	1	0	0
0	0	0	0	0	0	0	3	0	0
0	0	0	1	0	0	1	1	0	0
0	0	0	1	0	0	1	3	0	0
1	1	0	1	1	0	1	1	1	3
0	0	0	0	0	0	0	0	0	0
1	0	0	0	0	0	0	0	0	0
0	0	0	1	0	0	3	1	0	0
0	0	0	0	0	0	0	0	0	0
0	0	0	0	0	0	0	1	0	0
0	0	1	0	1	0	1	0	1	0
1	0	0	2	0	0	1	3	1	1
0	0	0	1	1	0	1	1	1	1
1	0	0	0	0	0	0	0	0	0
1	0	0	0	0	0	0	1	0	0
0	0	0	1	0	0	0	0	0	0
1	0	1	0	0	0	0	0	1	0
1	1	1	0	1	1	1	3	0	0
0	0	0	1	0	0	1	1	0	0
0	0	0	0	0	0	0	0	0	0

1	0	0	0	0	0	0	0	0	0
0	0	0	0	0	0	0	0	0	0
0	0	0	0	0	0	0	0	0	0
1	0	0	1	1	1	0	2	1	1
0	0	0	0	0	0	1	1	0	0
0	0	0	0	1	0	0	1	3	0
2	1	1	0	1	3	1	0	3	3
0	0	0	0	0	0	1	0	0	0
0	0	0	0	0	0	0	0	0	0
1	1	1	1	1	1	1	1	1	1
1	1	1	1	1	1	1	1	1	1
0	0	0	2	0	2	1	1	0	0
0	0	0	3	0	0	0	3	0	0
0	0	0	3	0	1	1	0	1	1
1	0	1	0	0	0	0	0	0	0
0	0	0	3	1	1	0	3	0	0
0	0	0	0	0	0	0	0	0	0
0	0	0	0	0	0	0	0	0	0
0	0	0	3	0	0	0	3	0	0
1	3	3	1	1	1	3	2	3	0
0	0	0	3	0	0	1	2	0	0
0	0	0	3	0	0	0	3	0	0
0	0	0	0	0	0	0	0	0	0
0	0	0	3	0	0	3	3	0	0
0	1	1	0	0	1	0	0	0	0
0	1	2	2	3	2	0	2	1	0
0	0	0	3	2	2	2	3	2	0
2	0	1	3	1	1	1	3	1	1
0	1	0	1	3	0	0	3	0	0
0	0	0	2	0	0	0	3	0	0
3	0	2	3	3	0	3	3	3	0
0	0	0	0	0	0	0	0	0	0
0	0	0	0	0	0	0	0	0	0
0	0	1	2	1	0	2	1	1	0
0	0	0	3	0	0	2	3	0	0
0	0	0	3	0	0	0	3	0	0
0	0	0	3	0	0	0	3	0	0
0	1	1	1	1	1	0	0	0	0
0	0	0	3	0	1	0	3	0	0
0	0	0	0	0	1	0	0	0	0
1	2	1	2	1	1	0	2	0	1
0	1	0	3	0	1	0	1	0	1
0	0	0	0	0	0	0	0	0	0
0	0	0	0	0	0	0	0	0	0
0	0	0	0	0	0	0	0	0	0
0	1	0	3	0	2	0	0	0	0
1	0	0	0	0	0	0	0	1	0

0	0	0	3	0	0	0	0	3	0
0	1	1	1	3	3	3	0	1	3
0	0	1	3	0	2	1	3	0	0
0	0	0	3	0	1	0	3	0	0
1	0	3	1	0	0	1	3	0	0
0	0	0	3	0	0	3	3	0	0
0	0	0	3	0	0	3	3	0	0
0	0	0	3	0	0	3	3	0	0
0	0	1	3	1	0	3	2	0	0
0	0	0	1	0	0	1	3	0	0
0	0	0	3	0	0	3	3	0	0
0	0	0	3	0	0	3	3	0	0
0	0	0	3	0	0	3	3	0	0
0	0	0	3	0	0	3	3	0	0
0	0	0	3	0	0	3	1	0	0
0	0	0	3	0	0	3	3	0	0
0	0	0	3	0	0	3	3	0	0
0	0	0	3	0	0	1	1	1	0
0	0	0	3	0	0	2	3	0	0
0	1	2	0	3	2	0	1	1	1
3	0	3	2	3	3	3	1	3	1
0	0	0	3	0	0	3	3	0	0
0	0	0	3	0	0	3	3	0	0
1	0	1	1	1	0	3	2	3	1
0	0	0	3	0	0	3	3	0	0
0	3	1	2	1	2	1	1	3	2
0	0	0	3	0	0	3	3	0	0
3	0	3	1	0	0	1	1	0	0
0	0	0	3	0	0	0	3	0	0
0	0	0	3	0	0	3	3	0	0
0	0	0	3	0	0	3	1	0	0
3	0	1	1	1	0	1	0	0	0
0	0	0	3	0	0	3	3	0	0
0	0	1	2	0	1	3	3	0	0
1	0	0	2	1	0	2	3	0	0
0	0	0	3	0	0	3	3	0	0
3	0	1	2	0	0	3	3	3	0
0	0	0	3	1	1	2	0	1	1
0	1	1	2	1	1	2	1	2	0
1	1	1	3	0	0	3	1	0	0
1	1	1	2	1	1	2	2	2	1
1	0	1	1	1	0	1	1	0	0
0	0	0	3	1	0	3	3	0	0
0	0	0	3	2	0	3	2	0	0
0	0	0	3	0	0	3	2	3	0
1	1	1	2	1	1	2	2	1	1
1	0	0	2	1	0	2	2	0	0

0	0	0	3	0	0	3	2	0	0
0	0	0	3	0	0	3	3	0	0
1	1	0	1	1	1	2	1	0	0
3	0	0	2	0	2	3	3	0	0
0	0	0	0	0	0	0	0	0	0
1	1	3	1	0	0	0	3	0	0
0	0	0	0	1	1	1	1	1	1
3	0	1	1	3	1	1	1	1	1
3	3	1	3	3	3	3	3	3	3
0	0	0	1	0	0	3	0	0	0
0	0	0	3	0	0	0	0	0	0
3	3	0	1	3	3	3	0	1	0
0	0	0	0	0	0	0	0	0	0
0	1	0	1	0	0	0	0	0	0
0	0	0	0	0	0	0	0	0	0
0	0	0	0	0	0	0	0	0	0
0	0	0	0	0	0	0	0	0	3
0	0	0	0	0	0	0	0	0	0
0	0	0	0	0	0	0	1	1	0
0	0	0	3	0	0	0	0	0	0
0	0	0	0	0	0	0	0	0	0
0	0	0	0	0	0	0	0	0	0
1	1	1	1	1	1	1	0	0	0
3	3	3	3	3	1	1	1	3	2
0	0	0	0	0	0	0	0	0	0
0	0	0	0	0	0	0	1	1	1
3	2	3	1	1	3	1	1	1	1
2	0	0	0	0	0	0	0	0	0
1	3	2	0	0	3	3	3	0	2
0	0	0	0	0	0	0	3	0	0
0	0	0	3	0	0	0	3	0	0
0	0	0	0	0	0	0	0	0	0
0	0	1	2	0	0	0	0	0	0
3	3	3	0	3	3	3	0	0	0
0	0	0	3	0	0	0	3	0	0
0	0	0	3	1	0	0	3	3	0
0	0	0	3	0	0	1	3	0	2
3	1	3	0	1	3	3	3	3	3
0	0	0	3	0	0	0	3	0	0
3	3	3	0	3	3	1	1	3	3
0	0	0	3	0	0	0	3	0	0
0	0	0	3	0	0	1	3	0	0
1	0	1	0	2	2	0	0	1	2
0	0	0	0	0	0	0	0	0	0
0	0	0	3	3	0	0	0	0	0
0	0	0	1	1	0	1	2	0	0
0	0	0	3	0	0	0	3	0	0

0	0	0	0	0	0	3	3	0	2
0	0	1	3	0	1	1	3	0	0
2	0	0	3	3	0	1	3	2	1
0	0	0	3	1	0	1	2	0	0
0	0	0	3	0	0	0	3	0	0
1	1	1	0	0	1	0	0	1	0
0	0	0	3	0	0	3	3	0	0
0	0	0	3	0	0	0	3	0	0
0	0	0	0	0	0	0	0	0	0
0	3	0	3	1	1	1	3	1	0
0	0	0	0	0	0	0	0	0	0
0	0	0	3	0	0	0	3	0	0
0	0	0	1	0	0	1	2	0	0
0	0	0	0	0	0	0	0	0	0
3	1	1	0	2	1	0	0	1	3
0	0	0	3	0	0	0	3	0	0
0	0	0	0	0	0	0	0	0	0
0	0	0	3	0	0	0	2	1	0
0	1	0	1	1	0	1	2	0	0
1	0	0	2	2	1	1	3	1	0
1	0	3	0	1	1	0	0	1	0
0	0	0	0	0	0	0	0	0	0
0	0	0	0	0	0	0	0	0	0
0	0	0	0	0	0	0	0	0	0
0	1	1	3	3	1	0	0	2	2
0	0	0	1	0	1	1	0	0	0
0	0	0	3	0	1	1	1	1	0
0	1	1	0	1	0	0	0	0	0
0	1	3	3	0	0	0	0	0	0
1	0	0	3	0	0	3	3	0	0
3	0	0	3	1	1	1	1	1	1
0	0	0	0	0	0	0	0	0	0
0	0	0	0	0	0	0	0	0	0
0	0	0	0	0	0	0	0	0	0
0	1	0	0	0	0	0	0	0	0
0	0	0	0	0	0	0	0	0	0
2	2	2	2	2	2	2	1	2	1
0	0	0	0	0	0	0	0	0	0
0	0	0	0	0	0	0	0	0	0
0	0	0	0	0	0	0	0	0	0
0	0	0	0	0	0	0	0	0	1
0	0	0	3	1	0	2	3	1	0
0	0	0	3	1	0	0	3	0	0
0	0	0	2	0	0	0	0	0	0
3	3	1	3	3	1	2	3	0	0
0	0	0	0	0	0	0	0	0	0
2	0	0	2	1	3	1	2	0	0

0	0	0	2	0	1	0	1	0	0
1	1	1	1	1	3	3	1	0	0
0	0	0	0	0	0	0	0	0	0
0	0	0	0	0	0	0	0	0	0
0	2	0	3	0	0	0	1	2	0
2	1	0	0	1	2	0	1	2	0
0	0	0	3	0	0	0	3	0	0
0	0	0	3	0	0	0	3	0	0
1	0	0	0	0	0	2	1	0	0
2	0	0	0	0	0	0	0	0	0
1	0	1	2	1	0	1	2	0	1
0	0	0	3	0	0	0	3	0	0
3	3	3	3	3	3	3	0	3	3
3	1	3	0	3	1	3	0	3	3
0	0	0	1	1	0	0	0	1	0
1	0	1	3	0	0	1	3	0	0
0	0	0	3	0	0	0	3	0	0
3	1	2	0	1	2	1	2	2	0
3	1	2	0	1	2	1	2	2	0
0	0	0	0	0	0	0	0	0	0
0	1	2	3	1	2	3	1	2	3
0	0	0	3	0	0	0	2	0	0
0	0	1	0	0	1	1	1	1	1
0	0	0	0	0	0	0	0	0	0
0	0	0	3	0	0	0	0	0	0
0	0	0	0	0	1	1	1	1	1
1	3	3	0	3	3	3	2	3	3
0	2	0	0	0	0	1	3	0	0
0	0	0	0	0	0	0	0	1	1
0	0	0	0	0	0	0	0	0	0
0	1	1	1	1	1	1	1	1	1
0	0	1	3	0	0	0	0	0	0
0	0	1	0	0	0	0	0	0	0
3	1	1	1	1	1	1	1	1	1
1	1	1	1	1	1	1	1	1	1
0	0	0	2	2	0	0	0	0	0
1	1	3	0	0	0	0	0	0	0
1	1	1	1	1	1	0	0	0	0
0	0	0	0	0	0	0	0	1	1
0	0	1	0	1	0	1	1	1	1
0	0	0	0	1	1	1	0	0	0
0	1	1	1	1	0	1	0	1	1
1	1	1	1	1	1	1	1	1	1
0	0	0	1	1	1	1	0	1	3
0	1	0	0	0	0	0	0	0	0
2	0	1	0	0	0	0	0	0	0
3	1	1	0	0	1	0	0	0	0

0	0	0	0	0	0	0	0	0	0
1	1	1	1	1	1	1	1	1	1
0	0	0	1	0	1	1	1	1	1
0	1	0	1	1	1	1	3	1	0
2	0	2	0	2	2	0	2	0	0
0	2	0	0	1	3	2	1	0	0
0	0	0	1	0	0	3	3	0	0
0	0	0	1	0	0	3	3	0	0
0	1	3	0	2	3	1	2	0	2
0	0	0	3	3	0	0	2	0	0
0	0	0	0	0	0	1	0	1	0
0	0	2	0	1	1	3	1	2	0
2	0	2	0	2	1	3	0	3	3
0	0	0	1	0	0	0	3	0	0
0	0	0	0	0	0	0	0	0	0
0	1	0	1	1	0	0	1	0	0
0	1	0	1	1	0	0	1	0	0
0	0	0	0	0	0	0	0	0	0
0	0	0	0	0	0	0	0	0	0
1	1	1	1	1	1	1	1	1	1
0	0	1	1	0	0	0	1	0	0
0	0	1	1	0	1	0	3	1	0
0	0	0	0	0	0	0	0	0	0
0	0	0	0	0	0	0	0	0	1
0	0	0	1	0	0	1	0	0	0
0	3	0	1	1	0	1	1	0	0
2	0	0	0	0	0	0	0	0	2
1	1	1	0	0	1	1	1	1	1
0	0	0	0	1	0	0	1	1	1
3	1	1	1	1	1	1	0	3	3
1	0	0	0	0	0	0	0	0	0
0	1	0	3	0	0	0	3	0	0
3	2	0	0	0	1	0	0	0	0
1	1	1	1	1	1	0	1	0	0
0	0	0	0	0	0	0	0	0	0
0	0	0	0	0	0	0	1	1	1
0	0	0	1	1	1	1	1	1	2
0	0	0	0	0	0	0	0	0	0
0	0	0	0	0	0	0	0	0	0
0	0	0	0	0	0	0	0	0	0
0	0	0	1	1	0	0	1	0	0
0	0	0	1	3	0	3	3	0	0
0	0	0	3	0	0	0	3	0	0
0	3	1	1	1	3	3	0	3	0
0	0	0	0	0	0	0	0	0	0
2	0	1	3	1	1	1	2	0	0
0	0	0	0	0	0	0	0	0	0

0	0	0	0	0	0	0	0	0	0
0	0	0	3	0	0	0	3	0	0
0	0	0	3	0	1	0	3	0	0
1	1	1	1	0	0	0	0	0	0
0	1	1	0	0	1	1	0	3	1
0	0	0	0	0	0	0	0	1	1
0	0	0	0	0	0	0	0	0	0
0	0	0	3	0	0	0	3	0	0
0	0	0	3	1	2	0	0	3	0
0	2	0	2	0	0	0	2	0	1
0	0	0	0	0	0	0	0	0	0
0	0	0	0	1	1	1	0	1	0
0	1	0	0	0	0	0	0	0	1
0	0	0	3	0	0	3	3	0	0
0	1	0	1	0	0	3	3	0	0
0	0	0	2	0	2	2	3	0	0
0	0	0	3	0	0	3	3	0	0
0	0	0	3	0	0	3	3	0	0
1	1	1	2	1	1	2	1	1	0
0	0	2	0	3	3	0	0	3	1
0	0	0	3	0	0	3	3	0	0
0	0	0	3	0	0	3	1	0	0
0	1	0	2	1	0	2	3	1	0
0	0	0	3	0	0	3	3	0	0
0	1	0	2	1	1	2	1	0	0
0	0	0	2	1	0	0	2	0	1
1	2	2	1	1	1	1	1	1	1
0	0	0	2	0	0	2	2	0	0
0	0	0	3	0	0	3	3	0	0
0	0	0	3	0	0	3	3	1	0
0	0	0	3	0	0	1	3	0	0
0	1	0	3	0	3	0	0	0	0
0	0	0	3	0	0	3	3	0	0
0	0	0	2	1	0	3	3	0	0
0	0	0	3	0	0	3	3	0	0
1	1	0	2	1	0	2	2	0	0
0	0	0	3	0	0	3	3	0	0
0	0	0	2	1	0	3	3	0	0
1	0	0	3	1	0	3	3	0	0
1	0	0	3	1	0	3	1	1	0
1	0	0	1	0	1	0	0	1	1
2	0	1	2	1	1	2	2	1	0
0	0	0	3	0	0	3	3	0	0
0	0	0	1	0	0	1	3	0	0
0	0	0	3	0	0	3	3	0	0
0	0	0	3	0	0	3	3	0	0
1	0	0	2	1	0	2	2	0	0

1	0	1	1	1	1	0	0	0	1
0	0	0	0	0	0	3	1	0	0
0	0	0	3	0	0	3	3	0	0
0	0	0	3	0	0	3	1	0	0
0	0	0	0	0	0	1	3	0	0
0	0	2	3	0	0	0	1	0	0
1	0	0	0	0	0	0	0	0	0
1	0	1	0	2	3	3	2	3	1
0	0	0	0	0	0	0	0	0	0
0	0	0	0	0	0	0	0	0	0
0	0	0	0	0	0	0	0	0	0
0	0	0	0	0	0	0	0	0	0
1	1	1	1	1	1	1	1	1	1
0	0	0	3	0	0	0	0	0	0
0	0	0	0	0	0	0	0	0	0
0	0	0	0	0	0	0	0	0	0
1	1	1	1	1	1	1	1	1	1
0	0	0	0	0	0	0	0	0	0
1	1	1	1	1	1	1	1	1	1
0	0	0	3	0	0	0	3	0	0
0	1	0	0	0	0	0	0	0	1
0	0	1	2	1	0	2	3	0	1
0	0	0	3	0	1	0	3	0	0
1	0	3	1	2	2	2	0	0	0
0	0	0	3	1	0	0	1	0	0
0	0	0	3	0	0	0	3	0	0
0	0	3	0	3	3	3	3	0	3
3	1	3	1	3	2	3	1	3	3
0	2	3	2	0	3	3	1	2	3
3	0	3	0	1	3	0	3	0	0
0	0	0	3	0	0	0	3	0	0
0	0	0	3	0	0	0	3	0	0
0	0	0	3	0	0	0	3	0	0
0	3	3	0	3	3	3	0	3	0
0	0	0	2	1	1	0	3	1	0
1	3	1	0	1	1	3	3	0	0
0	3	3	1	1	1	1	1	1	1
0	0	0	0	0	0	0	0	0	0
0	0	0	3	3	1	0	3	0	0
2	0	1	0	1	1	1	2	1	0
0	0	0	3	0	0	3	3	0	0
0	0	0	3	0	0	0	3	0	0
1	1	1	1	1	3	0	0	0	0
3	3	3	0	3	3	0	3	3	3
3	3	0	3	3	0	3	3	3	0
0	0	0	3	0	0	1	3	0	0
0	0	0	3	0	0	3	3	0	0

0	0	0	3	0	0	3	3	1	0
2	0	0	3	3	0	3	3	0	0
0	0	0	3	0	0	1	3	0	0
0	0	0	3	0	0	0	3	0	0
0	0	0	3	0	0	3	3	0	0
0	0	0	0	0	0	0	0	0	0
0	0	0	2	2	0	2	2	1	0
1	1	3	1	3	1	0	1	1	2
0	0	0	3	0	0	1	3	0	0
0	0	0	0	0	0	0	0	0	0
0	0	0	3	0	0	3	3	0	0
0	0	0	0	1	0	1	3	1	0
1	0	0	3	1	0	1	3	0	0
0	3	3	0	1	3	0	1	0	0
0	0	0	0	0	0	0	0	0	0
0	0	0	3	0	0	3	3	1	0
0	0	0	0	0	0	0	0	0	0
0	0	0	0	0	0	0	0	0	0
0	0	0	3	0	0	0	3	0	0
0	0	0	0	0	0	0	0	0	0
0	0	0	0	0	0	0	1	0	3
0	0	0	0	1	0	0	0	1	0
0	0	0	0	0	0	0	0	0	0
0	0	0	0	0	0	0	0	0	0
1	0	0	1	0	2	1	0	3	3
0	0	0	0	0	0	0	0	0	0
0	1	0	0	0	0	0	1	0	1
0	0	0	3	3	1	0	0	1	0
1	2	0	0	0	2	1	1	1	1
0	1	1	0	0	0	0	0	0	0
0	0	2	2	0	0	0	0	0	0
0	0	0	0	0	0	0	0	0	0
0	0	0	0	0	0	0	0	0	0
0	1	1	3	1	3	3	3	1	0
0	0	0	0	0	0	1	1	0	0
1	3	3	0	3	2	2	1	3	3
0	0	0	1	0	1	1	1	1	0
0	0	0	3	0	0	0	3	0	0
0	0	0	3	0	0	0	3	0	0
1	0	0	3	2	0	0	3	0	1
2	1	1	0	0	2	0	1	0	0
1	0	1	0	1	2	1	0	1	2
3	3	3	0	3	3	0	0	3	3
0	3	1	1	0	0	0	1	1	0
0	0	0	1	3	1	3	3	0	0
0	0	0	0	0	0	0	1	0	0
0	0	0	3	0	0	0	3	0	0

0	0	0	3	0	0	0	3	0	0
0	0	0	3	0	0	0	3	0	0
0	0	0	3	0	0	0	3	0	0
0	0	0	3	0	0	0	3	0	0
0	0	0	0	0	0	0	0	0	0
3	0	0	0	3	0	0	3	0	0
0	0	0	3	0	0	0	0	0	0
1	3	1	0	1	1	1	1	1	1
0	1	3	1	1	1	1	1	1	1
3	0	1	1	1	0	0	0	0	0
0	0	0	0	0	0	0	0	0	0
0	1	3	1	1	1	1	1	1	1
0	0	0	0	0	0	0	0	0	0
1	1	1	0	0	1	0	0	1	0
1	1	1	0	0	0	0	0	0	0
1	0	1	0	0	0	0	0	0	0
0	0	0	0	0	0	0	0	0	0
1	1	0	0	0	0	0	0	0	0
0	0	0	0	0	0	0	0	1	1
0	0	0	0	0	0	0	0	1	1
3	1	1	0	0	0	0	0	0	0
0	0	0	0	0	0	0	0	0	0
1	3	0	1	0	0	1	1	0	0
0	0	0	0	0	0	0	0	0	0
0	0	0	0	0	1	1	0	0	1
0	0	0	1	0	1	2	2	0	0
0	0	0	1	1	1	0	1	1	1
0	0	0	1	0	0	0	3	0	0
0	1	3	0	1	3	3	1	3	1
0	1	1	1	3	1	0	0	1	1
1	1	1	1	0	0	0	3	0	1
2	0	2	0	2	2	0	1	0	2
0	0	0	1	0	3	1	1	1	1
2	1	2	0	2	3	0	0	2	3
0	1	1	3	0	0	3	2	0	0
0	1	0	3	0	0	1	3	1	0
1	1	1	1	1	1	1	1	0	1
0	0	0	1	1	1	1	3	0	0
0	1	0	1	0	0	0	0	0	0
1	1	1	1	1	1	1	1	0	0
1	0	0	1	1	1	1	1	1	0
1	1	1	0	1	1	3	0	1	3
0	0	0	0	0	0	0	0	0	0
1	1	0	1	1	1	1	1	1	1
1	1	1	1	1	1	1	1	1	1
0	0	0	0	0	0	0	0	0	0
1	0	0	0	0	1	1	1	0	1

0	0	0	0	0	1	0	0	0	0
0	0	0	0	0	0	0	0	0	0
0	1	0	0	0	0	0	1	0	0
0	0	0	0	0	0	0	0	0	0
0	0	0	0	0	0	0	0	0	0
0	0	0	1	0	0	0	0	0	0
0	0	0	0	0	0	0	0	0	0
0	0	0	0	0	0	0	0	0	0
0	0	0	0	0	0	0	0	0	0
0	0	0	1	0	0	0	1	0	0
0	0	0	1	0	0	0	1	0	0
1	1	1	1	1	1	1	1	1	1
1	1	1	1	1	1	1	1	1	1
1	3	1	3	2	2	1	1	1	1
0	0	0	3	0	0	0	3	0	0
0	0	0	0	0	0	0	0	0	0
3	3	3	0	2	3	1	1	2	0
2	1	0	3	0	0	2	3	0	0
0	0	0	3	0	0	0	3	0	0
0	0	0	3	2	1	0	1	2	1
2	0	2	3	1	0	2	3	3	2
0	0	1	0	0	0	0	1	0	1
0	2	0	0	1	0	0	3	0	1
0	0	0	0	0	0	0	0	0	0
0	0	0	3	1	0	0	3	0	0
0	0	0	3	0	0	3	3	0	0
0	0	0	3	3	1	0	1	3	2
0	0	1	3	0	0	0	0	1	0
0	0	0	3	0	2	1	3	0	1
0	0	0	3	0	0	3	3	0	0
0	0	0	3	0	0	3	3	0	0
0	0	0	3	0	0	2	3	0	0
0	0	1	1	0	0	1	1	0	0
0	0	0	3	0	0	3	3	0	0
0	0	0	3	0	0	3	3	0	0
1	1	0	0	0	0	2	2	0	0
1	0	0	2	0	0	2	2	0	0
0	0	0	3	0	0	1	3	0	0
0	0	0	3	0	0	2	2	0	0
0	0	0	3	0	0	3	3	0	0
0	0	2	3	0	0	3	0	3	0
2	0	0	2	0	0	2	2	1	0
0	0	2	3	2	0	2	2	0	0
0	0	0	3	0	0	3	3	0	0
0	0	0	3	0	0	3	3	0	0
0	0	0	3	0	0	3	1	0	0
0	0	0	3	0	0	2	3	0	0

0	0	0	2	0	1	3	2	0	0
1	0	1	2	2	1	1	2	0	1
0	0	0	3	0	0	1	0	0	0
0	0	0	3	1	1	3	3	0	0
0	3	0	3	0	0	3	3	0	0
0	1	3	2	0	0	0	1	0	0
2	0	0	3	0	0	0	0	1	0
3	0	0	3	0	0	0	3	0	0
0	1	3	2	0	0	0	1	0	0
0	0	0	0	0	0	0	0	0	0
1	1	1	3	3	3	3	1	1	3
0	0	0	0	0	0	0	0	0	0
3	0	0	3	0	0	0	3	0	0
0	0	0	0	0	0	0	0	0	0
0	0	0	0	0	0	0	0	0	0
0	0	0	0	0	0	0	0	0	0
0	0	0	0	0	0	0	0	0	0
0	0	0	0	0	0	0	0	0	0
0	0	0	0	0	0	0	0	0	0
0	0	0	3	0	0	0	3	0	0
0	2	0	3	0	0	0	3	0	0
0	0	0	3	0	0	0	3	0	0
3	1	0	1	3	3	0	3	0	0
3	0	3	0	3	3	3	1	0	3
0	0	0	3	1	3	3	0	3	3
0	0	0	3	0	0	0	3	0	0
1	3	3	0	3	3	3	2	2	2
0	0	0	3	0	1	0	2	0	0
0	2	0	3	0	0	1	3	0	0
3	2	3	0	2	2	2	0	0	2
0	1	0	3	1	0	1	3	0	0
3	3	3	0	0	3	3	0	3	3
0	0	0	3	0	0	0	3	0	0
0	3	3	0	3	3	3	0	0	0
1	1	0	2	0	0	0	2	0	0
3	0	0	2	0	0	0	3	0	0
0	0	0	0	0	0	0	3	0	0
0	0	0	3	0	0	0	3	0	0
0	0	0	1	3	3	1	1	3	0
0	0	3	0	0	0	3	3	0	0
0	0	0	1	0	0	0	3	0	0
0	0	0	2	1	0	2	3	0	0
0	0	0	3	0	1	3	0	0	0
0	0	0	3	0	0	1	3	0	0
0	0	0	3	0	3	3	3	0	0
0	0	0	0	0	0	0	0	0	0
0	2	0	3	3	3	0	0	0	0

0	0	0	0	0	0	0	0	0	0
0	0	0	1	0	0	1	1	0	0
2	3	0	0	3	2	3	1	3	1
0	1	1	1	1	0	3	3	0	0
0	3	1	0	1	1	0	3	0	0
3	0	0	1	3	0	2	0	2	0
0	0	0	0	0	0	0	0	0	0
0	0	0	3	0	0	3	3	0	0
0	0	0	3	0	0	0	0	0	0
0	0	0	0	0	0	0	0	0	0
0	0	0	3	0	0	0	3	0	0
0	0	0	0	0	0	0	0	0	0
0	0	3	3	0	0	1	3	0	0
0	0	0	0	0	0	0	0	0	0
0	0	0	0	0	0	0	0	0	0
0	1	0	0	0	0	0	0	0	0
0	0	0	0	0	0	0	0	0	0
0	1	0	3	0	1	0	1	0	1
3	3	2	0	3	3	3	3	3	3
0	0	0	0	0	0	0	0	0	0
1	0	0	1	2	1	0	0	0	0
0	0	0	0	0	0	0	0	0	0
0	0	0	0	0	0	0	0	0	0
0	0	0	0	0	0	0	0	1	0
0	0	0	0	0	0	0	0	0	0
0	0	0	3	0	0	3	3	0	0
0	0	0	1	1	0	0	0	0	0
0	1	2	0	2	3	2	0	3	0
0	0	0	3	0	0	0	3	0	0
0	0	0	1	2	1	0	1	0	0
0	0	0	1	1	0	0	0	0	0
0	0	0	3	0	0	0	3	0	0
0	0	0	3	0	0	0	3	0	0
0	0	0	3	0	0	0	3	0	0
0	0	0	3	0	0	0	3	0	0
0	0	0	3	0	0	0	3	0	0
0	2	2	2	2	3	2	2	1	3
0	0	0	0	0	0	0	3	0	0
1	0	1	1	1	1	3	3	3	1
2	3	0	3	1	1	3	1	0	0
0	1	1	1	1	1	2	1	1	2
0	0	0	2	0	0	2	3	0	0
0	0	1	3	0	1	0	1	0	0
0	0	0	0	0	0	0	0	0	0
1	1	1	1	1	0	0	0	0	0
1	0	0	1	1	0	1	0	1	0
1	1	0	3	1	1	0	0	0	0

0	0	0	0	0	0	0	0	0	0
0	0	0	1	1	0	0	0	0	0
0	0	0	0	0	0	0	0	0	0
0	0	0	1	1	0	1	0	1	0
3	1	1	0	1	1	1	1	0	0
0	0	0	0	0	0	0	0	0	0
1	1	1	1	1	1	1	0	1	0
1	1	1	1	1	1	1	1	1	1
0	0	0	0	0	1	1	1	0	0
1	0	0	0	0	0	0	0	0	0
1	1	1	1	1	0	0	0	3	0
3	0	1	1	1	1	1	1	1	1
1	1	1	1	0	0	1	1	1	0
0	0	0	0	0	0	0	0	0	0
3	2	2	0	2	3	0	0	0	2
0	0	0	3	3	0	3	3	1	0
0	3	1	1	3	0	3	0	1	0
0	3	1	1	3	0	3	0	1	0
1	1	0	0	0	0	1	1	0	0
0	1	0	1	1	3	3	1	1	1
0	3	0	3	0	1	0	3	3	1
0	1	1	0	1	3	1	1	1	0
1	3	1	3	1	3	3	3	0	1
1	1	0	0	0	0	0	1	0	0
3	0	0	3	0	0	0	3	0	2
0	0	0	0	1	0	0	1	1	1
1	1	1	1	1	1	1	1	1	1
0	0	0	0	0	0	0	1	0	0
0	0	0	1	0	0	1	1	0	0
0	0	0	1	1	0	1	3	0	0
0	0	0	0	2	3	0	1	1	0
0	1	0	1	1	1	1	1	1	0
0	0	0	1	0	1	1	0	1	1
0	1	1	1	0	1	0	1	0	1
0	0	1	0	1	0	1	0	1	0
1	1	0	0	1	1	1	1	1	1
1	1	0	1	0	0	0	1	0	0
0	0	0	0	0	0	1	1	0	0
0	1	3	0	1	3	1	0	0	0
0	0	0	0	0	0	0	0	1	3
0	0	0	0	0	0	0	0	0	0
0	0	0	0	0	0	0	0	0	0
1	0	0	1	2	0	1	1	0	0
0	0	0	0	0	0	0	0	0	0
0	0	0	0	0	0	0	0	0	0
0	0	0	0	0	0	0	0	0	0
0	0	1	1	2	0	0	1	1	1

3	1	0	1	1	0	1	1	1	0
0	0	0	3	0	0	0	3	0	0
3	0	3	1	1	1	3	0	0	1
0	0	0	3	3	3	3	1	1	0
1	2	0	3	0	0	1	2	1	0
0	0	0	0	0	0	1	1	0	0
1	1	1	1	1	0	1	0	1	1
0	1	3	3	2	2	0	3	3	3
3	3	0	2	0	1	0	2	3	2
0	0	0	3	0	0	0	3	0	0
0	0	0	0	0	2	0	1	1	0
0	0	0	3	0	0	0	3	0	0
0	0	0	3	0	0	0	3	0	0
0	0	3	3	1	1	0	3	1	0
3	3	3	0	3	3	3	2	3	3
3	3	3	0	3	3	3	2	3	3
3	3	3	0	3	3	3	0	3	3
1	3	3	0	3	3	3	0	3	3
0	1	2	1	2	1	0	2	1	0
2	0	0	2	2	1	1	2	1	1
0	0	0	3	0	0	3	0	0	0
1	0	0	1	1	1	1	1	1	1
0	0	0	3	0	0	3	2	0	0
0	0	2	3	1	1	3	1	2	1
0	1	0	2	0	1	2	0	1	0
0	0	0	3	0	0	1	3	0	0
0	0	0	3	1	0	3	3	0	0
0	0	0	3	2	0	0	1	0	0
0	0	1	3	1	0	0	3	0	0
0	0	0	3	0	0	3	2	0	0
1	0	0	1	1	0	0	3	3	0
0	0	0	3	1	0	3	3	0	0
0	0	1	1	1	1	1	1	1	1
0	0	0	0	0	0	0	0	0	0
0	0	0	0	0	0	0	0	0	0
0	0	0	0	0	0	0	0	0	0
0	0	0	0	0	0	0	0	0	0
0	0	0	0	0	0	0	0	0	0
0	0	0	0	0	0	0	0	0	0
0	1	1	1	0	1	1	3	0	0
1	1	1	1	1	1	1	1	1	1
0	0	0	0	0	0	0	0	0	0
1	1	1	1	1	1	1	0	0	0
0	3	3	0	0	0	3	2	0	0
0	3	0	3	0	0	0	0	0	0
0	0	0	3	0	0	0	3	0	0
0	0	0	3	0	0	0	3	0	0

2	1	1	0	1	1	0	0	1	1
0	0	0	3	0	0	0	0	0	0
0	3	1	0	1	3	1	1	3	0
0	0	0	0	0	0	0	3	0	0
1	1	1	0	1	1	0	2	0	1
0	0	0	3	0	0	0	3	0	0
0	0	0	3	3	3	3	3	1	3
0	0	0	1	0	0	1	3	0	0
0	0	0	1	0	0	3	3	0	0
0	0	0	3	0	0	0	3	0	0
2	1	1	0	1	1	0	0	1	3
1	2	0	0	1	3	0	0	3	0
0	0	0	0	0	0	0	0	0	0
1	3	2	0	3	2	0	1	1	0
0	1	1	1	3	3	3	3	1	0
0	0	0	3	0	0	3	3	3	0
0	0	0	0	0	0	0	0	0	0
0	0	0	0	0	0	0	0	0	0
0	0	0	0	0	0	0	0	0	0
2	0	0	3	0	0	0	1	0	0
0	0	0	1	0	0	0	0	1	1
0	0	0	3	0	0	0	0	1	1
0	0	0	0	0	0	0	0	0	0
0	0	0	3	0	0	0	3	0	0
0	0	0	3	0	0	0	3	0	0
0	0	0	0	0	0	3	3	0	0
0	0	0	0	0	0	0	0	0	0
3	3	2	0	2	1	1	2	0	0
2	3	2	0	2	0	1	3	0	0
0	0	0	0	0	0	0	0	0	0
0	0	0	3	1	0	0	0	0	0
3	3	3	0	3	0	1	0	0	0
0	0	0	0	0	0	0	0	0	0
0	0	0	3	0	0	3	3	0	0
0	0	0	3	0	0	3	3	0	0
3	3	3	0	3	3	0	0	1	3
0	0	0	0	0	0	0	0	0	0
0	0	0	0	0	0	0	0	0	0
0	1	0	0	1	3	1	0	3	0
0	0	0	0	0	0	1	0	0	1
0	0	0	0	0	0	0	0	0	0
0	1	0	1	0	0	0	1	0	1
0	0	0	0	0	0	0	0	0	0
1	1	1	0	0	0	0	0	0	0
3	0	0	0	0	3	0	3	0	3
0	0	0	0	0	0	0	0	0	0
2	0	0	0	0	2	0	2	0	0

1	3	3	2	1	2	3	3	0	0
0	0	0	0	0	0	0	1	0	0
1	0	0	0	0	0	0	0	0	0
0	0	0	0	0	0	0	0	0	0
0	0	0	0	0	0	0	0	0	0
0	2	0	3	0	0	1	3	0	0
0	3	0	1	0	2	0	0	0	0
1	0	1	3	1	0	3	3	0	0
0	0	0	3	0	0	0	3	0	0
1	0	0	3	0	0	3	3	0	0
0	0	0	3	0	0	0	1	0	0
0	0	0	3	0	0	1	3	0	0
0	0	0	2	0	0	1	1	1	0
0	1	1	0	1	2	1	0	1	0
0	3	0	3	0	3	1	3	0	0
0	0	0	0	0	0	0	0	0	0
0	0	0	3	0	0	0	3	0	0
0	0	0	3	0	0	0	3	0	0
0	0	0	3	0	0	0	3	0	0
3	0	1	1	0	3	1	2	1	0
1	0	1	3	0	1	3	3	0	0
0	0	0	3	0	0	0	3	0	0
0	0	0	3	0	0	0	3	0	0
0	0	0	1	1	0	0	1	0	0
1	0	1	3	0	1	1	3	0	2
0	0	0	0	0	0	1	0	0	0
0	1	0	2	1	0	2	2	0	0
0	0	0	3	0	0	0	3	0	0
0	1	0	3	1	0	0	2	3	0
0	0	0	0	0	0	0	0	0	0
0	0	0	3	0	0	0	3	0	0
1	1	1	1	1	1	1	1	1	1
3	1	1	1	1	0	0	0	1	1
0	0	0	0	0	0	0	0	0	0
0	0	0	0	0	0	0	0	0	0
0	0	0	0	0	0	0	0	0	0
0	0	0	0	0	0	0	0	0	0
0	0	0	0	0	0	0	0	0	0
0	1	2	1	1	1	0	0	0	1
0	1	1	1	1	1	1	1	1	1
0	0	1	2	2	2	2	1	1	2
0	0	0	2	0	0	0	2	0	0
0	0	0	0	0	0	0	0	0	0
0	1	0	1	0	0	0	0	0	0
3	1	1	1	1	1	1	1	1	1
0	1	0	1	0	1	0	1	0	0
0	0	0	0	0	0	0	0	0	0

0	0	0	0	0	0	0	1	1	1
0	1	0	1	0	1	1	0	1	0
1	1	1	1	1	1	1	1	1	1
1	1	1	1	1	1	1	1	1	0
0	1	1	1	1	1	1	1	0	0
0	1	0	0	1	1	1	1	1	1
1	0	1	0	1	1	0	1	0	0
1	0	0	0	0	0	0	0	0	3
0	0	0	2	0	0	0	2	0	0
2	1	1	0	1	1	1	0	1	0
0	0	0	0	0	0	0	0	0	0
1	0	0	0	1	1	3	0	3	0
1	0	0	1	0	0	0	0	0	1
1	1	1	1	1	1	1	1	1	1
0	0	0	0	0	0	0	0	0	0
0	0	2	0	0	1	0	1	0	2
3	2	3	0	2	1	0	2	0	0
0	0	0	0	0	1	1	1	1	1
0	0	0	0	1	3	0	1	1	0
0	0	0	2	0	0	0	2	0	0
2	0	2	0	2	3	0	2	0	2
0	0	0	2	1	0	0	3	0	0
0	0	0	3	0	1	1	0	1	1
0	0	0	3	0	1	1	0	1	1
1	1	1	1	1	1	1	1	1	1
3	1	3	0	3	3	1	1	3	0
1	1	1	1	1	1	0	0	1	0
1	0	0	2	2	1	1	2	0	2
0	0	0	2	1	1	0	2	0	1
0	0	0	3	0	0	1	3	3	0
0	0	2	0	2	2	2	2	0	0
0	0	0	0	1	0	1	3	1	1
1	3	2	0	0	0	1	1	1	1
1	1	3	1	1	1	1	0	0	2
0	0	0	3	0	0	1	2	0	0
0	1	3	1	1	3	3	1	1	1
0	1	3	1	1	3	3	1	1	1
0	0	0	1	0	0	2	3	3	2
0	0	0	1	0	0	2	3	3	2
0	0	0	3	1	0	0	2	0	0
0	0	0	3	1	0	0	1	0	0
0	0	0	3	0	2	1	2	0	3
3	2	3	0	3	3	0	0	0	3
0	1	0	0	0	1	1	1	0	0
0	1	0	0	0	1	1	1	0	0
1	0	1	1	3	1	0	0	0	3
1	0	1	1	3	1	0	0	0	3

0	0	0	1	0	0	2	1	0	0
0	0	0	3	0	0	3	3	1	0
0	1	2	1	0	0	2	0	3	0
0	0	0	0	1	3	0	3	3	0
0	0	0	0	0	0	0	3	0	0
0	1	0	3	0	0	1	2	0	0
0	1	0	1	0	0	0	1	0	0
1	1	1	1	1	1	1	1	1	1
0	0	0	1	0	0	0	1	0	0
0	0	0	0	0	0	0	0	0	0
0	0	0	1	0	0	0	3	0	0
1	0	0	0	0	0	0	1	0	0
0	0	0	0	0	0	0	0	0	0
0	0	0	0	0	0	0	0	0	0
1	0	0	0	1	1	0	1	0	0
0	0	0	0	0	0	0	1	0	0
0	0	0	0	0	0	0	1	1	1
0	0	0	0	0	0	0	0	0	0
0	0	0	0	0	0	0	0	0	0
0	0	0	0	0	0	0	0	0	0
0	0	0	0	0	0	0	0	0	0
0	0	0	1	1	0	0	1	1	1
1	1	0	1	0	1	0	1	1	1
1	1	0	1	1	1	1	1	1	1
0	0	0	0	0	0	1	1	0	0
1	1	0	1	0	0	3	1	1	0
0	0	0	1	0	0	0	3	0	0
3	0	0	0	0	0	0	3	0	0
1	0	1	1	1	1	1	1	1	1
0	0	0	1	0	1	0	1	0	0
0	1	0	1	1	1	0	1	0	0
0	0	0	0	0	0	0	0	0	0
0	0	1	0	0	0	0	0	0	0
0	0	0	0	0	0	0	0	0	0
1	0	1	1	0	1	1	1	1	1
2	0	0	3	3	3	0	0	3	0
0	0	0	3	0	0	0	3	0	0
0	0	0	3	0	0	0	3	0	0
0	0	0	3	0	0	0	3	0	0
0	0	0	0	0	0	0	1	0	1
1	1	2	2	0	0	2	2	0	0
0	0	0	1	0	1	0	0	1	0
0	0	0	3	0	0	1	3	1	2
0	1	0	0	0	0	0	0	0	0
0	0	0	1	0	0	0	3	1	0
0	2	0	3	3	3	3	0	1	0
0	0	0	3	3	0	3	3	0	0

0	1	0	3	0	0	0	3	0	0
0	1	1	3	2	0	1	3	0	0
0	1	1	1	0	0	3	2	0	0
0	1	1	0	3	1	0	0	0	0
0	3	0	3	1	2	3	3	2	1
0	3	0	3	1	2	3	3	2	1
0	0	0	0	0	0	0	0	0	0
0	0	0	1	1	1	0	3	0	0
0	3	0	3	1	3	2	3	2	3
0	1	0	3	3	3	3	3	3	3
2	0	0	1	0	2	3	3	0	1
0	0	0	3	0	0	0	3	0	0
1	0	3	1	1	0	1	3	0	0
0	1	0	3	0	0	0	3	0	0
0	0	0	3	0	0	0	3	0	0
0	0	0	3	0	1	0	3	0	0
3	3	3	0	1	1	3	3	2	3
0	0	0	3	0	0	0	3	0	0
0	0	0	3	0	0	0	3	0	0
0	0	0	3	0	1	1	3	0	0
0	0	0	0	0	0	0	1	1	1
0	0	0	3	0	0	0	3	0	0
1	1	3	1	0	0	0	0	1	1
0	0	0	0	0	0	0	0	0	0
1	0	0	0	1	1	1	1	1	3
0	0	0	0	0	0	0	0	0	0
0	0	0	3	0	0	0	0	0	0
0	1	0	0	0	0	1	3	0	0
0	0	0	3	0	0	3	3	0	0
0	0	0	3	0	0	3	3	0	0
2	0	1	1	1	1	1	1	1	1
0	0	0	3	0	0	3	3	0	0
0	0	0	3	0	0	3	3	0	0
0	0	0	3	0	0	3	3	0	0
1	1	1	3	0	0	3	2	1	0
0	0	2	1	1	1	2	3	0	0
0	0	0	3	0	0	3	3	0	1
0	0	0	3	0	0	3	3	0	0
0	1	0	3	0	0	3	3	0	0
0	0	0	2	0	0	3	3	0	2
1	1	0	0	0	1	0	1	0	0
0	1	1	0	2	1	1	2	1	0
0	0	0	2	2	2	2	2	0	0
0	0	0	3	0	0	3	3	0	0
0	0	0	1	0	0	2	0	0	0
0	0	0	2	1	1	3	1	0	0
0	0	0	3	0	0	3	3	0	0

0	0	1	1	0	1	1	0	0	0
0	0	0	2	0	0	2	2	0	0
3	1	1	2	1	1	2	1	0	0
0	0	0	3	0	0	3	3	0	0
1	1	1	2	1	1	2	2	0	0
0	0	1	2	1	1	2	3	0	0
0	0	0	3	0	0	3	3	0	0
3	0	1	1	1	1	0	1	1	1
1	1	1	1	0	1	1	2	0	0
1	1	1	0	1	1	1	1	0	1
0	1	0	0	0	0	0	1	0	0
0	0	0	0	0	0	0	0	0	0
0	0	0	0	0	0	0	0	0	0
0	0	0	0	0	0	0	0	0	0
0	0	0	0	0	0	0	0	0	0
0	0	0	0	0	0	0	0	0	0
3	3	3	3	3	3	3	3	3	3
0	0	0	0	0	0	0	0	0	0
2	1	0	1	0	1	0	0	0	1
3	3	3	3	3	3	3	3	3	3
0	0	0	0	0	0	0	0	0	0
3	3	3	1	3	3	3	0	3	1
0	0	0	0	0	0	0	0	0	0
0	0	0	0	0	0	0	0	0	0
0	0	0	0	0	0	0	0	0	0
0	0	0	0	0	0	0	0	0	0
0	1	0	0	0	0	0	1	0	0
0	0	0	0	0	0	0	0	0	0
0	0	0	0	0	0	0	0	0	0
0	0	0	0	0	0	0	0	0	0
0	0	0	0	0	0	0	0	0	0
0	0	0	0	0	0	0	0	0	0
1	1	1	1	1	3	3	1	1	3
0	0	0	0	0	0	0	0	0	0
0	0	0	0	0	0	0	0	0	0
0	0	0	3	0	0	0	0	0	0
3	3	1	3	3	3	3	3	3	3
0	0	0	0	0	0	1	1	1	1
0	0	0	0	0	0	0	0	0	0
0	0	0	0	0	0	0	0	0	0
0	0	0	0	0	0	0	0	0	0
0	0	0	0	1	0	0	2	0	3
0	0	0	3	0	0	0	3	0	0
3	3	3	0	2	3	3	3	0	0
3	2	2	0	2	3	3	3	0	0
0	0	0	3	3	0	3	3	3	0
1	0	0	3	0	1	0	3	0	0

3	3	3	0	3	3	0	3	0	0
1	1	3	0	3	3	1	0	3	3
3	3	1	2	3	2	2	2	1	1
2	3	3	2	2	2	3	1	0	0
0	1	3	0	1	3	3	0	1	2
0	0	0	3	0	0	0	2	0	0
3	3	3	0	2	3	3	0	0	0
0	0	0	3	0	0	0	2	0	0
0	2	1	2	0	1	1	2	0	0
0	0	0	3	0	0	0	3	0	2
0	0	0	3	3	0	0	3	0	0
3	0	3	0	3	3	3	0	3	3
0	0	0	3	0	1	0	3	0	0
0	0	0	3	0	0	0	0	0	0
3	0	0	0	0	0	0	3	0	0
3	1	1	2	1	1	1	1	0	0
2	0	1	0	2	2	1	3	0	1
0	0	0	3	0	0	0	3	0	0
3	3	3	0	3	3	3	0	3	3
0	0	0	3	0	0	0	3	0	0
0	0	0	3	0	0	0	3	0	0
0	0	0	0	1	1	2	1	1	2
3	1	1	0	3	1	1	3	3	3
0	0	3	3	0	0	0	1	0	0
0	3	0	3	1	1	3	3	2	0
0	0	0	3	0	0	0	3	0	0
0	0	0	3	0	0	0	3	0	0
0	1	0	1	0	3	1	0	1	1
0	1	1	1	1	1	1	1	1	1
3	3	1	1	1	3	1	1	0	1
0	0	0	0	1	0	0	0	0	0
1	1	1	1	1	1	1	1	1	1
0	1	0	3	0	0	1	2	1	0
0	0	0	0	0	0	0	0	0	0
0	0	0	0	0	0	0	0	0	0
0	1	0	0	1	1	0	3	0	0
0	0	0	3	0	0	1	3	1	0
1	0	0	0	1	0	0	0	0	0
0	0	0	1	0	0	0	0	0	0
0	0	0	0	0	0	1	3	0	0
0	0	0	3	0	0	0	3	0	0
1	1	2	0	0	3	0	0	2	2
0	0	2	0	0	0	0	0	1	0
0	0	2	3	0	0	0	3	0	0
0	0	2	3	0	0	0	3	0	0
0	0	0	3	0	0	1	3	0	0
0	1	1	0	0	0	0	2	1	3

0	0	0	1	0	0	0	3	0	0
0	0	0	3	0	0	0	3	0	0
0	0	0	3	0	0	0	3	0	0
0	0	0	3	0	0	1	3	0	0
0	0	0	3	0	0	1	3	0	0
0	0	0	3	0	0	0	3	0	0
0	0	0	0	0	0	0	1	0	0
0	0	0	0	0	0	0	0	0	0
0	0	0	0	0	0	0	0	0	0
0	0	0	0	0	0	0	0	0	0
0	0	0	3	0	0	1	3	0	0
3	0	0	0	0	0	0	0	0	0
3	0	1	0	3	3	0	0	0	1
0	0	1	0	1	0	0	0	0	0
0	0	0	0	0	0	0	1	0	0
0	0	0	3	0	0	0	0	1	0
0	1	1	1	2	0	0	0	3	0
0	0	0	3	0	0	0	3	0	0
2	0	1	0	1	1	0	1	0	0
0	0	0	0	0	0	0	0	0	0
1	1	1	1	1	0	3	3	0	0
0	0	1	3	0	1	1	3	0	0
1	1	0	0	0	1	1	2	0	0
3	1	1	0	1	1	1	1	1	0
1	1	3	0	1	1	0	0	0	1
0	0	0	3	0	0	0	3	0	0
0	0	0	0	0	0	0	0	0	0
1	3	1	0	3	1	1	0	3	3
0	3	0	3	0	0	1	3	0	0
0	0	0	3	0	0	1	3	0	0
0	3	0	0	0	0	0	0	0	0
0	0	0	0	0	0	0	0	0	0
0	1	0	1	1	0	0	3	0	0
0	3	3	1	0	0	3	3	0	0
1	0	1	0	3	3	0	0	1	0
0	0	0	3	0	0	3	3	0	0
1	1	0	0	1	1	0	1	0	1
1	1	1	1	1	1	0	3	0	1
0	0	0	0	1	0	0	3	0	0
0	0	0	0	0	0	0	0	0	0

slep	happy	talkless	lonely	unfriend	njoylife	cry	sick	dislike	going
0	0	1	2	1	1	1	1	1	1
0	0	0	0	0	0	0	0	0	0
0	0	0	0	0	0	0	0	0	0
0	1	0	1	0	1	0	1	0	1
0	0	0	0	0	0	0	0	0	0
0	0	0	0	0	0	0	0	0	0
0	0	0	0	0	0	0	0	0	0
0	0	0	0	0	0	0	0	0	0
0	0	0	0	0	0	0	0	0	0
0	0	0	0	0	0	0	0	0	0
0	0	0	0	0	0	0	0	0	0
3	0	0	3	3	0	3	0	3	3
0	0	0	0	0	0	0	0	0	0
0	0	0	0	0	0	0	0	0	0
0	0	0	0	0	0	0	0	0	0
0	1	1	1	0	0	0	0	0	0
0	1	0	0	0	2	0	0	0	0
1	3	0	1	0	3	1	1	0	0
0	3	3	0	0	3	2	0	0	0
0	0	0	0	0	0	0	0	0	0
2	1	0	1	1	1	2	1	1	1
1	0	0	3	0	0	2	1	0	3
0	3	1	1	1	3	0	0	1	0
0	3	0	0	0	0	0	0	0	0
0	3	0	0	0	0	0	0	0	0
0	3	0	0	0	3	0	0	0	0
0	3	1	3	0	2	1	3	0	2
1	0	3	2	1	3	3	1	1	1
3	0	0	0	0	2	2	1	1	0
3	0	3	3	1	0	3	3	0	3
0	0	0	0	0	3	0	0	0	0
0	3	0	0	0	3	0	0	0	0
2	1	0	1	0	0	0	1	1	0
0	0	0	1	1	0	0	0	0	0
0	0	0	0	0	0	0	0	0	0
0	3	0	0	0	3	0	0	0	0
0	0	0	3	1	0	0	2	1	1
0	3	0	0	0	3	0	0	0	0
0	3	0	1	0	3	0	0	0	0
0	3	0	1	0	3	0	0	0	0
0	3	0	0	0	1	0	0	0	0
0	3	1	2	0	3	0	1	0	0
1	3	0	0	0	3	0	0	0	0
3	0	3	3	3	0	0	0	0	3
1	2	0	0	0	2	0	0	0	0
0	3	2	1	0	3	0	0	0	0

0	1	1	1	0	3	0	0	0	0
1	1	0	0	0	1	0	1	0	0
0	3	1	0	1	3	0	1	1	0
0	1	0	1	0	2	0	0	0	0
0	3	0	0	0	3	0	0	0	0
0	3	0	0	0	0	0	0	0	0
1	1	1	1	0	0	1	3	2	3
1	1	1	1	0	0	1	3	2	3
3	0	3	3	1	0	3	1	1	3
1	1	0	2	0	1	0	2	0	0
0	3	0	0	0	1	0	0	0	0
0	3	0	0	0	2	0	0	0	1
0	3	0	0	1	3	1	1	0	1
2	3	2	0	1	3	1	0	1	1
0	1	0	0	0	0	0	0	0	0
1	2	2	0	0	1	0	0	0	0
0	3	0	0	0	3	0	0	0	0
0	3	0	2	0	3	0	0	0	0
0	3	0	0	0	0	0	0	0	0
0	3	3	0	0	1	0	0	0	0
1	1	0	0	0	0	1	0	0	1
0	0	0	0	0	0	0	0	0	0
1	0	0	1	0	1	1	0	1	0
0	3	0	0	0	0	0	0	0	0
0	3	0	0	0	0	0	0	0	0
1	0	1	0	1	0	1	0	1	0
1	0	1	0	1	0	1	0	1	0
1	0	0	0	0	0	0	1	3	1
0	0	0	0	0	0	0	0	0	0
0	3	0	0	0	0	0	0	0	0
0	3	0	0	0	0	1	0	0	1
0	3	0	0	0	0	0	0	0	0
1	1	1	1	1	1	1	1	1	1
1	1	1	1	1	1	1	1	1	1
0	0	0	0	0	0	0	0	1	1
0	0	0	0	0	0	1	1	0	0
0	0	0	0	0	0	0	0	0	0
1	1	1	1	1	1	1	1	1	1
1	3	1	1	1	1	1	1	1	1
0	0	0	0	0	0	0	0	0	0
0	1	0	0	0	0	0	0	0	0
1	1	1	1	1	1	1	1	1	1
0	3	0	0	0	0	0	0	0	0
0	1	0	0	0	1	1	0	0	0
0	1	0	1	1	0	0	0	0	0
1	1	1	1	1	1	1	1	1	1
1	1	1	0	0	0	0	0	0	0

1	1	0	0	1	0	1	1	1	0
0	0	0	0	0	0	0	0	0	0
1	1	1	1	1	1	1	1	1	1
0	2	0	0	0	1	0	0	0	0
2	1	2	3	2	0	0	0	0	0
1	1	0	3	1	1	1	1	1	1
3	0	3	1	1	2	1	1	3	1
0	3	0	0	0	2	0	0	0	0
2	0	2	3	1	0	2	1	2	2
0	3	0	0	0	2	0	0	0	0
0	2	0	0	0	2	0	0	0	0
0	3	0	0	0	3	2	1	0	0
3	0	0	3	0	0	1	0	2	1
0	3	0	0	0	1	0	1	1	0
1	1	1	1	1	0	3	1	1	1
0	3	0	0	0	1	0	0	0	0
2	0	2	2	0	0	0	0	0	0
3	0	3	3	3	0	3	0	2	1
2	0	0	3	3	0	0	0	0	2
1	0	1	3	0	0	3	0	0	1
0	3	0	0	0	3	0	1	0	1
1	3	0	1	0	3	0	0	0	0
0	2	0	0	0	2	0	0	0	0
0	3	0	0	0	1	0	0	0	0
0	3	0	0	0	3	0	0	0	0
0	2	1	1	1	3	3	2	0	2
0	3	0	0	0	3	0	0	0	0
1	0	1	1	0	0	0	0	0	1
1	0	1	1	0	0	0	0	0	1
0	1	0	0	0	0	0	0	0	0
0	1	0	0	0	0	0	0	0	0
0	0	0	0	0	1	0	0	0	0
0	1	1	1	1	1	1	1	1	1
0	3	0	0	0	0	0	0	0	0
0	3	0	0	0	0	0	0	0	0
1	0	3	0	0	3	0	0	0	0
0	3	0	0	0	3	0	0	0	0
0	0	0	0	0	0	0	0	0	0
0	1	0	0	0	0	0	0	0	0
1	1	1	1	1	1	1	0	0	0
0	1	0	1	1	1	1	1	1	1
0	3	0	0	0	3	0	0	0	0
0	0	0	0	0	3	0	0	0	0
0	1	0	0	0	0	0	0	0	0
0	3	0	0	0	0	0	0	0	0
0	0	1	1	3	0	1	3	1	1
1	1	1	3	1	0	1	0	1	1

0	3	0	0	0	3	0	0	0	0
0	3	0	0	0	3	0	0	0	0
0	0	0	0	0	0	0	0	0	0
0	2	1	0	0	0	0	0	0	0
1	2	2	1	0	2	1	1	0	1
0	3	0	0	0	3	0	0	0	0
0	1	0	0	0	0	0	0	0	0
0	0	0	0	0	0	0	0	0	1
0	3	0	0	0	3	0	0	0	0
0	3	0	0	0	3	0	0	0	0
0	3	0	0	0	2	0	0	0	0
0	0	0	0	1	0	0	0	0	0
0	3	0	2	0	0	0	0	0	0
0	0	0	0	0	0	0	0	0	0
0	0	0	0	0	0	0	0	0	0
0	0	0	0	0	3	0	0	0	0
0	3	0	0	0	3	0	0	0	0
0	3	0	0	0	3	0	0	0	0
1	2	0	1	0	0	0	0	1	0
1	2	0	1	0	0	0	0	1	0
0	3	0	0	0	2	0	0	0	0
0	3	0	0	0	3	0	0	0	0
0	3	0	0	0	3	0	0	0	0
2	1	1	2	1	0	2	0	2	1
1	3	0	0	0	3	0	0	0	0
0	3	0	0	0	3	0	0	0	0
0	1	0	0	0	1	0	0	0	0
0	3	0	0	0	3	0	0	0	0
0	3	0	0	0	3	0	0	0	0
1	2	0	0	1	1	0	1	0	1
0	3	0	0	0	3	0	0	0	0
0	3	0	0	0	3	0	0	0	0
0	3	0	0	0	3	0	0	0	0
0	3	0	0	0	3	0	0	0	0
0	0	0	0	0	0	0	0	0	0
0	3	0	0	0	3	0	0	0	0
0	3	0	0	0	3	0	0	0	0
1	2	2	1	1	2	1	1	1	1
1	3	1	0	0	2	0	0	0	0
1	2	1	1	1	2	0	1	1	1
0	3	0	0	0	3	0	0	0	0
0	3	0	0	0	3	0	0	0	0
2	2	1	1	0	2	2	2	0	0
0	3	0	0	0	3	0	0	0	0
0	1	0	0	0	1	0	0	0	0
0	3	0	0	0	1	0	0	0	0
0	3	0	0	0	3	0	0	0	0

0	3	0	0	0	3	0	0	0	0
0	2	0	1	0	1	1	2	0	0
0	3	0	0	0	3	0	0	0	0
0	2	0	0	0	0	0	0	0	0
0	2	0	2	0	2	0	0	0	0
0	3	0	0	0	3	0	0	0	0
0	3	1	0	0	3	0	0	0	0
1	1	1	1	0	1	1	0	0	1
0	3	0	0	0	2	0	0	0	0
0	3	0	0	0	1	0	0	0	0
0	3	0	0	0	3	0	0	0	0
0	3	0	0	0	3	0	0	0	0
0	3	0	0	0	3	0	0	0	0
0	2	0	0	0	2	0	0	0	0
0	3	3	3	0	0	0	0	3	0
1	0	0	1	1	0	0	0	0	0
0	1	0	1	0	0	0	0	0	0
0	0	0	0	0	0	0	0	0	0
0	0	0	0	0	0	0	0	0	0
0	0	1	1	1	1	1	1	1	1
1	1	1	0	1	0	0	0	0	1
0	3	0	0	0	0	0	0	0	0
1	3	0	0	0	3	0	0	0	0
0	0	0	0	0	0	0	0	0	0
1	0	0	1	1	0	0	0	0	0
1	1	2	0	0	0	0	0	0	0
1	1	1	1	1	0	0	0	0	0
0	0	0	0	0	0	0	0	0	0
0	3	0	0	0	0	0	0	0	0
0	0	0	0	0	0	0	0	0	0
3	3	3	3	3	3	3	3	1	3
0	0	0	0	0	0	0	0	0	0
0	0	0	0	0	0	0	0	0	0
0	0	0	0	0	0	0	0	0	0
0	2	0	1	0	2	1	1	0	0
0	1	0	3	0	3	3	0	0	0
0	3	0	0	0	3	0	0	0	0
0	3	0	0	0	3	0	0	0	0
1	2	2	0	0	2	0	0	0	0
1	0	0	1	0	0	0	0	0	0
0	3	0	0	0	3	0	0	0	0
0	3	0	0	0	3	0	0	0	0
0	2	1	0	0	2	0	0	0	0
2	0	3	3	3	0	3	3	3	1
0	3	0	1	0	0	0	0	0	0
0	3	0	0	0	3	0	0	0	0
1	2	2	1	0	2	0	0	0	0

0	3	0	0	0	3	0	0	0	0
0	3	0	0	0	3	0	0	0	0
0	3	0	0	0	3	0	0	0	0
0	3	0	0	0	3	0	0	0	0
0	0	0	0	0	0	0	0	0	0
0	3	0	0	0	0	0	0	0	0
0	3	0	0	0	3	0	0	0	0
0	2	1	0	0	0	0	0	0	0
0	2	0	3	1	1	0	0	0	0
0	3	0	0	0	0	0	0	0	0
1	0	1	1	1	1	1	0	1	1
0	3	0	0	0	3	0	0	0	0
0	3	0	0	0	3	0	0	0	0
1	3	0	0	0	3	0	1	0	1
0	1	0	1	0	0	0	0	0	1
0	0	1	2	0	0	0	0	0	0
0	3	0	0	0	2	0	0	0	0
0	1	0	0	0	0	0	0	0	0
0	1	0	0	0	0	0	0	0	0
0	3	0	0	0	0	0	0	0	0
0	3	0	0	0	0	0	0	0	0
1	3	1	1	1	0	1	1	0	1
1	0	1	3	1	1	3	0	1	1
0	1	0	1	0	0	3	0	0	3
3	0	3	3	3	0	3	0	0	3
0	3	0	0	0	0	0	0	0	0
1	1	0	2	0	0	2	0	0	0
0	0	1	1	1	0	3	0	0	1
0	0	0	0	0	3	0	1	0	0
0	0	0	0	0	0	0	0	0	0
0	0	0	0	0	0	0	0	0	0
0	3	0	0	0	3	0	2	0	0
0	3	0	0	0	3	0	2	0	0
0	0	0	0	0	0	0	0	0	0
0	0	3	3	3	3	3	3	3	3
1	1	1	1	1	0	0	0	0	1
0	0	0	0	0	0	1	0	0	0
0	0	0	1	2	0	2	0	0	1
0	0	0	0	0	0	0	0	0	0
0	0	0	0	0	0	0	0	0	0
0	0	0	0	0	0	0	0	0	0
2	0	0	1	0	0	1	0	0	0
0	0	0	0	0	0	0	0	0	0
0	0	0	0	0	0	0	0	0	0
1	0	0	0	0	0	1	0	0	0
1	2	1	2	0	0	0	0	0	0
0	1	0	0	0	1	0	3	0	0

0	0	0	0	0	0	0	0	0	0
0	3	0	0	0	3	3	0	0	0
0	0	0	0	0	1	0	0	0	0
0	3	1	0	0	1	0	0	0	0
0	0	0	0	0	0	0	0	0	0
0	2	1	3	0	0	0	2	0	0
0	2	0	0	0	2	1	0	0	0
0	3	0	0	0	3	0	0	0	0
0	1	0	0	0	1	0	0	0	0
0	3	0	0	0	0	0	0	0	0
0	3	0	0	0	3	0	0	0	0
3	1	1	1	1	3	0	0	0	1
0	3	0	0	0	3	0	2	0	0
0	1	1	0	0	0	0	0	0	0
0	1	1	0	0	0	0	0	0	0
3	1	1	0	3	1	3	2	0	1
2	2	3	2	0	0	1	1	0	0
1	2	1	1	1	2	0	0	0	0
0	2	0	0	0	2	0	0	0	0
3	1	1	0	0	3	2	0	0	0
0	3	1	0	0	0	0	0	0	0
2	2	2	0	0	1	0	2	0	0
0	3	1	0	0	3	0	0	0	3
0	1	2	1	0	1	0	0	0	0
2	0	3	1	0	0	0	0	0	0
2	1	0	3	1	0	1	2	0	1
0	3	0	0	0	3	0	0	0	0
0	3	0	0	0	3	0	0	0	1
0	0	0	3	0	3	3	3	0	0
0	3	0	0	0	3	0	0	0	0
0	1	0	1	0	2	0	0	0	1
0	3	0	2	0	3	0	0	0	1
0	3	0	0	1	3	0	0	0	0
1	1	0	0	0	1	0	1	0	1
1	0	0	1	0	0	1	1	0	1
1	2	0	0	0	3	0	1	0	0
2	0	1	0	0	0	0	1	0	0
0	2	1	0	0	2	0	0	0	0
1	1	2	3	0	0	1	0	0	0
1	3	0	0	0	3	0	1	1	1
0	3	0	0	0	2	0	0	0	1
0	3	0	1	0	1	0	0	0	0
0	3	0	0	0	3	0	0	0	0
0	1	1	0	0	1	0	0	0	0
0	3	2	0	0	1	0	0	0	0
1	2	1	0	0	1	0	1	2	1
0	3	0	0	0	0	0	0	0	0

0	3	0	0	0	0	0	0	0	0
0	3	0	0	0	2	0	0	0	0
0	3	2	1	0	2	0	0	1	0
3	0	3	3	0	0	0	3	0	3
0	3	1	0	0	3	0	1	0	1
0	2	1	0	0	3	0	0	0	0
0	3	0	0	0	3	0	0	0	0
0	3	0	0	0	3	0	0	0	0
0	3	2	3	0	3	2	0	3	2
0	3	0	0	0	0	0	0	0	0
0	3	0	0	0	3	0	0	0	0
0	3	0	0	0	3	0	0	0	0
2	0	0	3	0	0	1	1	0	1
0	2	0	1	1	3	1	0	1	1
0	3	0	0	0	0	0	0	0	0
1	1	0	1	0	0	0	1	2	1
0	3	1	0	0	3	0	1	0	0
0	3	1	0	0	3	0	0	0	0
1	1	0	1	0	1	2	2	0	2
0	3	0	0	0	1	0	0	0	0
0	3	0	0	0	3	0	0	0	0
0	3	0	0	0	3	0	0	0	0
0	3	0	0	0	3	0	0	0	0
1	2	0	0	0	1	0	2	0	0
0	3	0	2	0	3	0	0	0	0
0	3	0	0	0	0	0	0	0	0
0	3	0	0	0	2	0	3	0	0
0	3	0	0	0	3	0	0	0	0
2	0	2	2	0	0	2	0	0	1
0	3	0	0	0	3	0	0	0	0
0	2	3	0	0	0	3	0	0	3
1	1	1	1	1	1	1	1	1	1
0	0	0	0	0	0	0	0	0	0
0	1	0	0	0	1	1	1	1	1
0	3	0	0	0	0	0	0	0	0
0	0	3	0	0	0	0	0	0	0
1	1	2	0	0	0	2	0	0	0
1	0	1	0	0	0	0	0	0	0
0	0	0	0	0	0	0	0	0	0
1	0	1	0	0	1	0	1	0	0
0	3	0	2	0	0	1	0	0	0
0	1	1	1	0	1	0	1	1	1
0	0	0	0	0	0	0	0	0	0
0	0	0	0	0	0	0	1	1	0
0	0	0	0	0	0	0	0	0	0
0	0	0	1	0	0	0	0	0	0
0	1	0	1	0	1	0	1	1	1

0	3	0	0	0	0	0	0	0	0
0	1	0	0	0	0	0	0	0	0
0	1	0	0	0	0	0	0	0	0
1	1	1	1	1	1	1	1	1	1
0	0	0	0	0	0	0	0	0	0
0	0	0	0	0	0	0	0	0	0
1	1	1	1	1	1	1	1	1	1
0	0	0	0	0	0	0	0	0	0
1	1	0	0	0	0	0	0	0	0
1	0	1	1	1	0	1	1	1	1
2	0	2	1	1	0	2	2	1	1
0	0	0	0	1	0	1	0	0	0
0	3	0	0	0	0	0	0	0	0
0	3	0	0	0	2	0	0	0	0
0	2	0	0	0	2	0	0	0	0
3	0	0	3	0	0	0	1	1	3
0	3	0	0	0	0	0	0	0	0
0	3	0	0	0	0	0	1	0	0
0	3	2	0	0	1	0	0	0	0
0	3	0	0	0	0	1	2	0	0
0	0	0	0	0	1	0	0	0	1
1	3	1	0	0	0	0	0	0	0
1	3	0	1	1	0	0	0	0	0
0	3	0	1	1	1	0	0	0	0
0	3	0	0	0	0	2	0	1	0
0	3	0	0	0	0	0	0	0	0
3	0	2	3	2	0	0	0	0	3
1	0	0	0	1	0	3	1	0	1
0	3	0	0	0	3	0	0	0	0
0	2	0	0	0	2	0	0	0	0
0	2	0	0	0	1	0	1	0	3
0	1	0	0	0	0	0	0	0	0
0	1	0	0	0	0	0	0	0	0
0	3	0	0	0	0	0	0	0	0
0	1	0	0	0	0	0	0	0	0
0	1	0	1	1	0	1	0	1	1
0	0	0	1	1	0	1	1	1	1
0	3	0	0	0	1	0	0	0	0
0	1	0	0	0	0	0	0	0	0
0	1	0	0	0	0	0	0	0	0
0	0	0	0	0	0	0	0	0	0
1	0	0	1	0	0	0	0	0	0
0	3	0	0	0	1	0	1	0	0
0	1	0	0	0	0	0	0	0	0
0	1	0	0	0	0	0	0	0	0
1	3	0	0	0	3	0	3	0	1
0	1	0	0	0	0	0	0	0	0

0	1	1	0	0	0	0	1	2	0
0	3	0	0	0	3	0	0	0	0
1	0	1	1	1	0	1	0	1	1
0	3	0	1	0	0	0	0	1	1
1	1	1	1	1	1	0	0	0	1
0	3	0	0	0	0	0	0	0	0
0	3	0	0	0	0	0	0	0	0
0	3	0	0	0	0	0	0	0	0
0	3	0	0	0	0	0	0	0	0
0	3	0	0	0	0	0	0	0	0
0	3	0	0	0	0	0	0	0	0
1	1	1	1	1	1	3	3	3	3
0	0	0	0	0	0	0	0	0	0
0	3	0	0	0	0	0	0	2	0
1	3	1	0	0	0	0	0	0	0
0	3	0	0	0	1	0	0	0	0
3	0	3	3	3	0	0	1	1	3
0	1	0	0	1	0	0	0	0	0
1	1	1	1	1	1	1	0	0	0
0	0	0	0	0	0	0	0	0	0
0	3	0	0	0	3	0	0	0	3
1	3	0	0	0	3	0	2	0	1
0	3	0	0	0	3	0	0	0	0
0	0	0	0	0	0	0	0	0	1
0	3	0	0	0	3	0	1	0	1
0	0	0	0	0	0	0	0	0	0
1	3	1	1	1	2	2	1	1	1
0	2	0	0	0	2	0	0	0	0
0	3	0	0	0	3	0	0	0	0
1	2	0	0	0	2	0	0	0	0
1	0	0	0	0	0	0	0	0	0
0	0	0	0	0	0	0	0	0	0
0	0	0	0	0	0	0	0	0	0
0	3	3	0	0	3	0	2	0	0
0	3	0	0	3	2	0	0	0	3
2	1	1	2	0	2	2	0	2	2
0	3	1	0	0	3	0	0	0	3
1	0	1	1	0	1	1	0	0	1
0	0	0	0	0	0	0	0	0	0
0	3	0	0	0	3	0	0	0	0
1	3	0	0	0	3	0	0	0	1
0	3	0	0	0	3	0	0	0	0
0	2	1	1	1	3	0	1	3	1
0	0	0	1	1	0	1	1	1	1
2	1	0	2	1	0	1	1	1	1
0	3	0	0	0	1	0	0	0	0
0	3	0	0	0	3	0	0	0	0

0	3	0	0	0	3	0	0	0	0
0	3	0	0	2	3	0	0	0	0
0	3	0	0	0	3	0	0	0	0
0	2	0	0	0	3	0	0	0	0
1	2	0	1	1	2	1	0	0	0
0	3	0	0	0	3	0	0	0	0
0	3	0	0	0	3	0	0	0	0
0	3	0	0	0	3	0	0	0	0
0	3	0	0	0	3	0	0	0	0
0	3	0	0	0	3	0	0	0	0
1	1	1	3	3	3	0	1	1	1
0	3	0	0	0	3	0	0	0	0
0	3	0	0	0	3	0	0	0	0
0	3	0	0	0	3	0	0	0	0
0	2	0	0	0	3	0	0	0	0
0	3	0	0	0	3	0	0	0	0
1	1	1	0	1	2	1	2	2	1
0	3	0	0	0	3	0	0	0	0
0	3	0	0	0	3	0	0	0	0
1	2	0	0	0	3	0	0	0	0
0	3	0	0	0	3	0	0	0	0
0	3	0	0	0	3	0	0	0	0
1	2	1	1	0	2	0	0	0	0
0	3	0	0	0	3	0	0	0	0
0	3	0	1	1	2	0	0	0	0
3	2	2	2	0	1	1	1	0	1
1	0	3	2	0	0	0	0	0	2
0	3	0	0	0	3	0	0	0	0
0	3	0	0	0	2	0	0	0	0
0	2	0	0	0	3	0	0	0	0
0	2	0	0	0	3	0	0	0	0
0	3	0	0	0	3	0	0	0	0
1	1	0	1	0	1	0	1	0	1
1	1	1	0	0	0	0	0	0	0
0	3	0	0	1	3	0	0	0	0
0	3	0	0	0	3	0	0	0	0
0	3	0	0	0	3	0	0	0	0
1	3	0	1	1	3	0	0	0	0
0	3	0	0	0	3	0	1	0	0
0	3	0	0	0	2	0	0	0	0
0	3	0	0	0	3	0	0	0	0
1	2	1	1	0	2	0	0	0	1
0	2	0	0	0	3	0	0	0	0
0	3	0	0	0	3	0	0	0	0
0	3	0	0	0	3	0	0	0	0
0	0	0	0	0	0	0	0	0	0
0	3	0	0	0	0	0	0	0	0

0	3	0	0	0	0	0	0	0	0
0	3	0	0	0	0	0	0	0	0
3	0	0	0	3	3	3	0	3	3
0	0	0	0	0	0	0	0	0	0
0	3	0	0	0	3	0	0	0	0
0	3	0	0	0	3	0	0	0	0
1	0	1	1	1	1	1	1	1	1
0	0	0	0	0	0	0	0	0	0
0	3	0	0	0	0	0	0	0	0
0	3	0	0	0	0	0	0	0	0
0	3	0	0	0	0	0	0	0	0
0	0	0	0	0	0	0	0	0	0
0	0	0	1	2	0	0	0	0	0
2	0	0	0	0	0	0	0	0	0
1	1	1	1	1	1	1	1	0	0
0	3	0	0	0	0	0	0	0	0
1	3	1	1	1	3	1	1	1	1
0	1	0	0	1	0	0	0	0	0
0	3	0	0	0	0	0	0	0	0
0	0	0	0	0	0	0	0	0	0
0	3	0	0	0	0	0	0	0	0
0	0	0	0	0	0	0	0	0	0
0	3	0	0	0	0	0	0	0	0
1	2	1	1	0	2	0	0	0	0
1	0	2	2	0	0	1	0	2	1
0	3	0	0	0	3	0	0	0	0
0	3	0	0	0	3	0	0	0	0
0	3	0	0	0	3	0	0	0	0
0	3	0	0	0	3	0	1	0	0
0	1	0	1	0	1	0	0	0	2
0	2	0	0	0	1	0	0	0	0
1	2	1	0	0	2	0	0	0	1
0	1	0	1	0	1	0	0	0	0
2	0	3	3	1	0	3	0	1	3
1	1	2	0	1	1	1	2	2	2
1	1	1	1	0	1	1	1	0	1
2	0	3	0	0	1	0	3	0	0
0	3	0	0	0	3	0	0	0	0
0	2	1	2	0	3	0	0	0	0
0	3	0	0	0	3	0	0	0	0
0	3	0	0	0	3	0	0	0	0
0	1	3	3	3	0	0	0	3	1
0	1	0	1	0	0	2	1	0	3
0	3	0	0	0	3	0	0	0	0
0	2	0	0	0	2	0	0	0	0
0	3	0	1	0	3	0	1	0	0
3	0	3	3	1	0	3	1	1	1

0	1	0	3	1	2	3	1	1	3
0	0	0	0	0	0	0	0	0	0
1	0	2	3	0	0	0	0	2	1
0	1	0	0	0	0	0	0	0	0
0	0	0	0	0	0	0	0	0	0
0	3	0	0	0	3	0	0	0	0
0	0	0	0	0	0	0	0	0	0
1	0	0	1	0	0	1	1	0	1
0	3	0	0	0	3	0	0	0	0
0	3	0	0	0	3	0	0	0	0
0	3	0	0	0	3	0	0	0	0
0	3	0	3	0	0	0	2	0	2
0	0	0	0	0	0	0	0	0	0
0	1	0	0	1	1	1	1	0	0
0	3	0	0	0	0	0	0	0	1
0	1	1	3	1	3	1	2	1	1
3	1	1	1	1	1	1	1	1	1
0	3	0	0	0	0	0	0	0	2
0	2	3	3	0	0	2	0	0	0
0	3	0	0	0	0	0	1	0	0
0	3	0	0	0	3	2	2	0	0
1	1	1	2	1	2	0	0	0	1
0	3	0	2	0	0	0	2	0	1
0	0	0	0	0	0	0	0	0	0
0	3	0	0	0	0	0	0	0	0
0	3	1	0	0	3	0	0	0	0
0	0	2	2	2	1	1	2	3	1
0	1	3	2	0	1	1	1	0	1
0	1	0	0	0	2	0	0	0	0
1	1	0	1	0	0	0	3	0	0
0	3	0	0	0	3	0	0	0	0
1	3	0	1	1	1	0	0	0	0
0	0	0	0	0	0	0	0	0	0
1	0	0	0	0	1	3	0	0	1
0	3	0	0	0	3	0	0	0	0
0	3	0	0	0	0	0	0	0	0
1	0	0	3	0	0	1	2	0	0
0	3	0	0	0	0	0	0	0	0
0	3	0	0	0	3	0	0	0	0
0	0	0	0	0	0	0	0	0	0
0	0	0	0	0	0	0	0	0	0
0	3	0	0	0	3	0	0	0	0
0	3	1	0	0	3	0	1	0	0
0	3	2	0	0	3	0	0	0	3
0	1	0	1	0	0	0	0	0	0
0	3	0	0	0	0	0	0	0	0
0	0	0	0	0	0	0	0	0	0

0	3	0	0	0	3	0	1	0	0
0	2	1	0	0	3	0	0	0	0
0	2	1	0	0	3	0	0	0	0
0	1	0	0	0	0	0	0	0	2
0	0	0	0	0	0	0	0	0	0
0	3	0	0	0	3	0	0	0	0
0	0	0	0	0	0	0	0	0	0
3	1	0	3	0	1	2	0	0	0
0	3	0	1	0	3	2	0	2	3
0	3	0	0	0	1	0	1	0	1
0	3	0	0	0	3	0	0	0	0
1	0	0	2	0	0	2	0	0	0
0	0	0	0	0	0	0	0	0	0
0	0	0	0	0	0	0	0	0	0
0	0	0	0	0	0	0	0	0	0
0	0	0	0	0	0	0	0	0	0
1	0	1	0	1	0	0	0	0	0
0	0	0	0	0	0	0	0	0	0
3	0	3	3	3	0	3	3	3	3
1	0	1	0	0	0	0	1	0	3
1	1	0	0	0	1	1	0	0	0
0	0	0	0	0	0	0	0	0	0
0	0	0	0	0	1	0	0	0	0
1	0	1	1	2	0	1	0	2	2
0	0	0	0	0	0	0	0	0	0
3	0	1	3	0	0	3	0	3	3
0	0	0	0	0	1	0	0	0	0
0	0	0	0	0	0	0	0	0	0
0	0	0	0	0	0	0	0	0	0
3	0	2	2	1	2	3	3	3	3
0	0	0	0	0	0	0	0	0	1
0	0	0	0	0	0	0	0	0	0
0	0	0	0	0	0	0	1	0	0
0	3	0	0	0	3	0	1	0	0
0	0	0	0	0	0	0	0	0	0
0	2	0	0	0	0	0	0	0	1
0	3	0	0	0	1	0	0	0	1
1	2	1	0	0	1	2	1	0	1
0	3	2	0	0	3	0	0	0	0
0	3	0	1	0	3	0	0	0	1
3	0	3	0	1	0	1	0	3	2
0	0	3	0	1	2	1	0	1	0
0	3	0	0	0	3	0	0	0	0
0	3	0	0	0	3	0	0	0	0
0	3	0	0	0	2	0	0	0	1
0	1	0	0	0	3	0	1	0	0
0	3	0	0	0	3	0	0	0	0

0	0	3	3	0	0	0	3	2	3
3	0	3	3	0	0	3	3	0	3
0	3	0	0	0	0	0	0	0	0
0	3	0	0	3	2	0	0	2	2
1	0	1	3	0	0	3	3	0	0
3	2	1	1	0	3	0	0	0	0
1	0	2	0	1	1	1	0	1	0
1	0	2	0	1	1	1	0	1	0
0	0	0	0	0	0	0	0	0	0
0	3	0	0	0	3	0	0	0	0
0	2	1	1	0	1	1	0	0	2
1	2	0	2	0	1	0	0	0	0
0	3	0	0	0	2	0	0	0	0
0	3	1	0	0	1	0	0	0	0
0	3	0	0	0	0	0	0	0	0
0	0	0	0	0	0	0	0	0	0
0	1	1	0	0	0	1	0	0	3
0	3	0	0	0	0	0	0	0	0
0	3	0	3	0	3	0	0	0	2
0	0	0	0	0	3	0	0	0	0
3	0	3	3	0	0	3	0	0	3
0	2	0	0	0	1	0	1	0	3
0	1	2	0	1	2	1	0	1	0
0	0	0	0	0	0	0	0	0	0
0	3	0	0	0	3	0	0	0	0
0	3	0	0	0	0	0	0	0	0
0	3	0	0	0	3	0	1	0	0
0	1	0	0	0	0	0	0	0	0
1	3	0	0	0	2	0	0	0	0
1	1	2	2	3	1	2	1	3	3
0	3	0	0	0	0	0	0	0	0
1	3	0	0	0	3	0	0	1	1
1	3	0	0	0	3	0	0	1	1
0	3	0	0	0	3	0	0	0	0
0	3	0	0	0	0	0	0	0	0
0	3	0	0	0	0	0	0	0	0
3	3	0	0	0	0	0	0	0	0
3	3	0	0	0	0	0	0	0	0
0	3	0	0	0	0	0	0	0	1
0	3	0	1	1	1	0	0	2	3
0	0	0	0	0	1	0	0	0	0
1	0	3	3	0	0	3	1	0	3
0	3	0	0	0	0	0	3	0	0
0	3	0	0	0	3	0	0	0	0
0	3	0	0	0	0	0	0	0	0
0	3	0	0	0	0	0	0	0	0
0	2	0	0	0	2	0	0	0	1

0	1	0	2	0	1	0	0	0	3
0	0	0	0	0	3	0	1	0	1
0	3	0	0	0	3	0	0	0	1
3	0	2	2	2	0	3	0	0	3
0	3	0	0	0	3	0	0	0	1
0	3	3	3	0	0	0	0	0	0
0	3	3	3	0	0	0	0	0	0
0	3	0	0	0	3	0	0	0	0
3	0	2	3	0	0	3	0	1	3
0	1	2	1	0	1	1	1	0	1
1	0	0	2	2	1	1	0	1	1
1	0	0	2	2	1	1	0	1	1
0	1	1	0	0	1	0	0	0	0
1	2	0	1	0	1	1	0	0	2
0	1	0	0	0	3	0	0	3	0
0	3	0	0	0	3	0	3	2	3
2	1	1	0	1	1	1	0	1	2
0	3	1	0	0	3	0	0	0	1
0	1	2	2	0	0	0	0	0	0
3	0	3	3	0	0	3	2	3	3
0	3	0	0	0	1	0	0	0	3
3	0	3	3	0	0	0	3	0	3
0	2	1	0	0	3	3	0	1	0
2	1	0	1	1	0	1	2	0	1
2	1	0	1	1	0	1	2	0	1
2	0	0	0	0	0	2	1	0	0
2	2	1	1	1	1	1	2	2	1
1	0	1	1	0	0	0	1	0	1
2	0	3	3	0	0	3	2	0	3
0	0	0	0	0	3	0	0	0	0
0	2	1	0	0	2	0	0	0	0
0	3	0	0	0	3	0	2	0	0
0	3	1	0	0	3	0	0	0	0
0	3	1	0	0	3	0	1	0	0
0	3	0	0	0	3	0	1	0	0
1	1	0	2	0	1	3	1	0	3
1	1	0	2	0	1	3	1	0	3
0	3	0	0	0	3	0	0	0	0
0	1	0	1	1	1	2	0	1	0
0	3	0	0	0	1	0	0	0	0
0	3	1	0	0	2	0	2	0	0
0	3	0	0	0	0	0	0	0	0
3	0	0	0	3	0	1	0	0	0
0	2	0	0	0	2	3	0	0	2
0	1	0	0	0	1	0	0	0	1
0	1	0	1	0	1	0	0	0	2
2	2	2	0	0	1	0	2	0	0

1	2	0	0	0	0	0	0	0	0
0	3	0	0	0	3	0	0	0	0
0	3	0	0	0	0	0	3	0	0
0	1	0	1	0	1	1	1	1	1
0	0	0	0	0	0	0	0	0	0
0	0	0	0	0	0	0	0	0	0
0	3	0	0	0	0	0	0	0	0
1	3	1	1	0	0	0	0	0	0
0	3	0	0	0	0	1	1	0	0
1	1	1	1	1	0	1	1	1	1
0	0	1	1	1	1	1	1	0	0
1	1	1	1	1	1	1	1	1	1
1	1	1	1	1	1	1	1	1	1
1	1	1	1	1	1	1	1	1	1
1	1	1	1	1	1	1	0	0	1
0	0	0	0	1	0	1	0	1	0
1	1	1	1	1	1	1	1	1	1
1	1	1	3	1	1	1	1	1	1
3	3	0	0	0	0	1	0	0	1
0	0	1	0	2	0	0	1	1	0
0	0	0	0	0	0	0	0	0	0
1	1	0	0	0	1	1	0	0	0
0	0	0	0	0	0	0	0	0	0
0	3	0	1	0	0	1	0	0	0
0	0	0	0	0	0	0	0	0	0
0	3	0	0	0	0	0	0	0	0
0	0	0	0	0	0	0	0	0	0
1	1	1	1	1	1	1	1	1	1
0	0	1	1	1	1	1	1	1	1
0	0	1	0	1	0	1	0	0	0
1	1	1	1	1	1	1	1	1	1
1	0	0	1	3	0	1	0	0	0
0	3	0	0	0	0	1	0	0	0
0	3	0	0	0	0	0	0	0	0
0	0	0	0	0	0	0	0	0	1
0	0	0	0	0	0	0	0	0	0
0	3	0	0	0	0	0	0	0	0
1	3	0	0	0	0	3	1	0	0
0	3	0	0	0	3	0	0	0	0
1	1	1	1	1	1	1	1	1	1
0	0	0	0	0	0	0	0	0	0
0	0	0	0	0	0	0	0	0	0
0	3	1	1	1	1	2	0	0	0
1	1	1	0	1	0	1	1	1	1
1	0	0	0	0	0	0	0	0	0
0	0	0	0	0	0	0	0	0	0
0	3	0	0	0	0	0	0	0	0

0	3	0	0	0	1	0	0	0	0
0	0	1	2	1	0	2	1	1	1
1	1	1	1	1	1	1	1	1	1
1	1	1	1	1	1	1	1	1	1
0	0	0	0	0	3	1	1	1	1
0	0	0	0	0	0	0	0	1	0
0	1	0	0	0	0	0	0	0	0
1	1	1	1	1	0	1	0	0	0
0	3	0	0	0	0	0	1	0	0
3	0	2	2	3	0	3	0	2	2
1	0	0	3	0	0	3	0	1	1
0	1	2	1	0	0	2	0	0	1
0	2	0	0	0	2	0	0	0	0
0	1	0	1	0	0	0	1	1	0
2	0	0	2	0	0	2	0	0	0
0	0	0	1	0	0	1	0	0	1
3	0	1	3	0	0	0	0	0	3
2	0	3	3	2	0	3	0	1	2
3	0	0	3	0	0	3	0	0	1
0	3	0	0	0	2	0	0	0	0
0	3	0	1	0	3	0	0	0	0
0	3	0	1	1	1	1	1	1	0
0	3	0	0	0	3	0	0	0	0
0	3	0	0	0	3	0	0	0	0
0	2	0	0	1	3	0	0	0	0
0	3	0	0	0	3	0	3	0	0
0	3	0	0	0	1	0	0	0	0
1	0	3	1	1	1	1	0	1	1
1	0	1	3	1	0	3	0	1	1
1	0	0	3	0	2	1	1	1	1
3	0	0	3	0	0	3	0	0	3
0	3	0	1	1	3	0	3	0	0
0	3	0	1	1	3	0	3	0	0
0	0	2	2	3	0	1	1	1	1
0	3	0	0	0	3	0	0	0	0
2	1	0	0	0	1	0	0	0	0
0	1	0	0	0	1	0	0	0	0
0	3	0	0	0	0	0	0	0	0
0	3	0	0	0	0	0	0	0	0
0	2	0	0	0	3	0	0	0	0
0	2	0	0	0	2	0	0	0	0
3	0	3	2	0	0	0	2	0	3
0	1	0	1	0	0	0	0	0	3
0	2	0	0	0	0	0	0	0	0
0	3	1	0	0	1	0	0	0	0
1	0	2	3	3	3	3	0	0	3
0	0	0	1	0	1	0	0	0	1

0	0	0	0	1	3	0	0	1	0
0	3	2	0	0	3	0	3	0	0
0	3	2	0	0	1	0	0	0	0
1	1	1	1	0	0	0	0	0	0
0	1	0	0	0	0	0	0	0	0
0	1	0	0	0	0	0	0	0	0
0	1	0	0	0	0	0	0	0	0
0	1	0	0	0	0	0	0	0	0
1	1	1	1	2	3	0	0	1	1
0	3	0	1	0	3	0	0	0	0
0	3	0	0	0	0	0	0	0	0
0	3	0	0	0	0	0	0	0	0
1	1	1	1	1	1	0	1	0	1
1	1	1	1	1	1	1	1	1	1
1	0	1	1	1	0	0	0	1	1
0	0	0	0	0	0	0	0	0	0
0	3	0	1	0	1	0	0	0	0
0	3	0	0	0	0	0	0	0	0
1	1	0	0	0	0	0	0	0	0
1	1	0	0	0	0	0	0	0	0
0	3	0	0	0	0	0	1	0	0
1	1	0	1	1	1	1	0	2	1
0	3	0	0	0	1	0	0	0	0
1	0	3	0	1	0	0	0	0	0
0	1	0	0	0	0	0	0	0	0
0	3	0	0	0	0	0	0	0	0
0	1	0	0	0	0	0	0	0	0
0	1	0	0	0	3	0	0	0	0
1	2	0	1	0	2	1	1	0	0
3	0	1	1	1	0	1	3	1	1
1	1	1	1	0	1	1	1	3	3
0	1	1	0	1	1	0	0	0	0
0	2	1	0	2	3	0	3	0	0
3	1	0	0	0	1	0	1	0	1
1	0	1	1	1	0	1	1	0	1
0	3	0	0	0	3	0	0	0	0
0	3	0	0	0	3	0	0	0	0
0	2	0	0	0	0	0	2	0	0
0	3	0	0	0	1	0	0	0	0
0	1	0	0	0	1	0	0	0	0
0	1	0	0	0	1	0	0	0	0
0	1	0	0	0	1	0	0	0	0
0	1	0	0	0	0	0	0	0	0
0	1	0	0	0	0	0	0	0	0
0	1	0	0	0	0	0	0	0	0
0	1	0	0	0	0	0	0	0	0
0	3	0	0	0	0	0	0	0	0

1	1	1	1	1	1	0	0	1	1
1	1	1	1	1	1	0	0	1	1
0	3	0	0	0	3	0	0	0	0
0	3	0	0	0	3	0	0	0	0
0	1	0	0	0	3	0	0	0	0
0	3	0	0	0	0	0	0	0	0
0	3	0	0	0	0	0	0	0	0
0	3	0	0	0	0	0	0	0	0
0	1	0	0	0	1	0	0	0	0
1	1	1	1	1	1	1	1	1	1
0	1	1	0	0	3	0	0	0	1
0	1	0	0	0	3	0	0	0	0
0	0	0	0	0	0	0	0	0	0
1	1	1	1	1	1	1	1	1	1
1	1	1	1	1	1	1	1	1	1
0	1	0	0	0	0	0	0	0	0
0	2	0	0	0	2	0	0	0	0
0	3	3	0	0	2	0	1	1	1
2	1	1	2	3	0	1	2	3	2
0	2	0	0	0	1	0	0	0	0
0	0	0	0	0	1	0	0	0	0
0	3	0	0	0	3	0	0	0	0
0	0	0	0	0	0	0	0	0	0
0	3	0	0	2	2	0	0	2	2
1	1	1	1	1	0	0	0	0	1
0	3	0	0	0	3	0	2	0	2
1	3	0	0	1	2	0	1	0	0
0	1	0	0	0	0	0	0	0	0
1	0	1	1	0	0	0	0	0	0
0	3	0	0	0	3	0	0	0	0
0	2	0	1	1	2	1	0	2	2
0	3	0	0	0	0	0	0	0	0
0	0	0	0	0	0	0	0	0	0
0	3	0	0	0	3	0	0	0	0
1	0	1	1	1	1	1	1	0	0
2	2	1	1	3	2	0	0	0	2
0	3	0	0	0	3	0	0	0	0
0	2	3	3	2	2	0	0	0	0
0	2	0	0	0	2	0	0	0	0
0	1	0	0	0	0	0	0	0	0
1	0	0	0	0	0	0	0	0	0
0	3	0	0	0	3	0	0	0	0
0	3	0	0	0	3	0	0	0	0
0	3	0	0	0	3	0	0	0	0
0	3	0	0	0	0	0	0	0	0
0	0	0	0	0	0	0	0	0	0
0	2	0	0	0	1	0	0	0	0

0	2	0	0	0	1	0	0	0	0
2	0	3	3	3	0	3	2	2	2
1	1	1	1	3	1	1	0	0	0
1	1	1	0	0	0	0	1	1	1
0	3	0	0	0	3	0	0	0	0
3	3	0	0	3	0	0	0	0	0
0	0	0	0	0	0	0	0	0	0
0	0	0	0	0	0	0	0	0	0
0	3	0	0	0	3	0	0	2	0
1	3	0	3	0	1	1	0	0	3
3	0	0	1	0	0	0	0	0	3
0	3	0	0	0	3	0	0	0	0
0	1	1	0	0	1	0	0	0	0
0	1	1	0	1	1	0	0	0	0
1	1	1	1	1	0	1	1	1	1
0	0	1	1	0	0	0	0	0	0
0	0	0	0	0	0	0	0	0	0
0	3	0	2	0	3	0	1	0	0
3	1	2	0	3	0	1	0	0	3
2	0	2	3	0	0	2	0	0	2
1	2	0	1	3	0	0	3	0	1
2	1	1	2	0	1	2	1	0	0
0	3	3	1	0	3	1	1	0	0
0	0	0	0	0	0	0	0	0	0
1	1	1	1	1	1	1	1	1	1
1	1	1	1	0	1	0	1	0	0
0	1	0	0	0	0	0	0	0	1
0	3	0	0	0	3	0	0	0	0
0	3	3	2	0	3	2	1	0	0
0	3	0	0	0	3	0	0	0	0
1	3	0	0	0	3	0	2	0	0
0	0	0	0	0	0	0	0	0	0
2	3	0	0	2	3	0	0	1	1
0	2	0	0	0	0	0	0	0	0
0	3	1	0	0	1	0	0	1	0
0	3	0	0	0	3	0	1	0	0
1	1	0	0	0	3	0	1	0	0
0	3	0	0	0	2	0	0	0	0
0	3	0	0	0	3	0	0	0	0
1	2	0	3	1	0	0	0	0	2
0	3	0	0	0	3	0	0	0	0
0	3	0	0	0	3	0	0	0	0
0	3	0	0	0	2	0	0	0	0
0	3	0	1	0	2	0	0	0	0
0	3	0	0	0	3	0	0	0	0
1	2	0	1	1	2	0	1	0	1
1	3	0	0	0	2	0	0	0	0

0	3	0	0	0	3	0	0	0	0
0	3	0	0	0	3	0	0	1	0
0	3	0	0	0	3	0	0	0	0
0	3	0	0	0	3	0	0	0	0
0	3	0	0	0	2	0	0	0	0
2	3	0	0	0	3	0	0	0	0
0	2	0	1	0	2	0	0	0	0
1	2	1	1	0	2	1	0	0	0
0	2	0	0	0	3	0	0	0	0
0	3	0	0	0	3	0	0	0	0
0	3	0	0	0	3	0	0	0	0
0	3	0	0	0	3	0	0	0	0
1	2	0	0	0	2	0	0	0	0
0	3	0	0	0	3	0	0	0	0
0	3	0	0	0	3	0	0	0	0
0	3	0	0	0	2	0	0	0	0
0	3	0	0	0	3	0	0	0	0
1	2	0	1	0	1	0	1	0	1
0	3	0	0	0	3	0	0	0	1
0	3	0	0	0	3	0	1	0	0
0	3	0	0	0	3	0	0	0	0
1	1	0	2	0	1	0	0	0	0
0	1	0	2	2	1	0	0	1	0
0	3	0	0	0	3	0	0	0	0
0	3	0	0	0	3	0	0	0	0
1	2	0	1	0	3	0	1	0	1
2	2	0	1	0	2	0	0	0	0
0	3	0	0	0	3	0	0	0	0
0	3	0	0	0	3	0	0	0	0
0	3	0	0	0	2	0	0	0	0
2	0	1	2	0	0	0	1	1	0
0	3	0	0	0	1	0	0	0	0
0	3	0	0	0	3	0	0	0	0
0	3	0	0	0	3	0	0	0	0
0	3	0	0	0	3	0	0	0	0
1	3	0	0	0	1	0	0	0	0
0	1	0	0	0	3	0	0	0	0
0	3	0	0	0	3	0	0	0	0
0	3	0	0	0	2	0	0	0	0
0	3	0	0	0	3	0	0	0	0
0	3	0	0	0	3	0	0	0	0
0	2	0	0	1	3	0	0	0	0
0	3	0	0	0	3	0	0	0	0
0	1	1	2	0	1	1	1	0	1
0	1	0	0	0	3	0	0	0	0
0	2	0	0	0	0	0	0	0	1
0	2	1	0	1	2	0	0	0	0

0	3	0	0	0	0	0	0	0	0
0	2	0	1	1	2	0	0	0	1
0	3	0	0	1	3	0	0	0	0
1	3	0	0	0	3	0	0	0	0
1	1	0	0	0	1	0	0	0	0
1	2	1	2	1	0	1	0	1	3
0	3	0	3	0	0	3	0	0	3
0	3	0	0	0	0	0	0	0	0
0	3	0	0	0	2	0	0	0	0
0	3	0	0	0	2	0	0	0	0
0	3	0	0	0	3	0	0	0	0
0	0	0	0	0	0	0	0	0	0
0	0	0	0	0	0	0	0	0	0
0	2	0	0	0	0	0	0	0	0
0	0	0	0	0	0	0	0	0	0
0	0	0	0	0	0	0	0	0	0
1	1	1	1	1	1	1	1	0	0
0	0	0	0	0	0	0	0	0	0
0	1	1	0	0	0	0	0	0	0
3	0	3	3	3	0	3	0	3	3
3	0	3	3	3	0	3	0	3	3
2	3	3	2	2	2	2	1	3	2
1	1	2	1	1	1	1	1	1	1
0	0	0	0	0	0	0	0	0	0
0	3	0	0	0	0	0	0	0	0
0	3	0	0	0	0	0	0	0	0
3	3	1	1	3	3	3	2	3	3
0	0	0	0	0	0	0	0	0	0
0	0	0	0	0	0	0	0	0	0
0	0	0	0	0	0	0	0	0	0
0	3	0	0	0	0	0	0	0	0
0	0	0	0	0	0	0	0	0	0
1	1	1	1	1	0	1	1	0	1
1	3	3	1	1	3	1	3	1	1
0	0	0	0	0	0	1	0	0	0
1	1	1	0	0	0	1	1	0	0
0	0	0	0	0	0	0	0	0	0
0	3	0	0	0	3	0	0	0	0
3	0	3	3	3	0	3	3	0	3
0	3	2	0	2	3	3	2	3	1
0	3	0	0	0	3	0	0	0	0
0	3	0	0	0	3	0	0	0	0
0	3	1	0	0	3	0	0	0	0
0	3	0	2	0	0	0	1	0	1
1	3	0	0	0	0	0	3	0	0
3	0	3	3	0	3	3	0	0	0
0	3	0	0	0	3	0	0	0	0

2	1	2	2	0	1	3	3	3	2
0	3	0	0	0	3	0	0	0	0
2	1	1	3	0	1	3	0	0	0
1	3	0	0	0	0	0	0	0	0
0	2	0	0	0	3	0	0	0	0
0	3	0	1	2	1	0	0	1	0
2	1	2	0	0	1	0	2	0	1
0	3	0	0	0	3	0	0	0	0
0	3	0	0	0	3	0	0	0	0
0	3	0	0	0	3	0	0	0	0
2	2	2	1	0	2	0	1	0	1
0	2	0	0	0	3	0	0	0	0
1	1	1	1	0	0	0	3	0	0
0	0	0	0	0	0	0	0	0	0
0	2	0	0	0	1	0	1	0	0
0	3	0	3	0	0	3	3	0	3
0	2	0	0	0	1	0	0	0	0
0	1	0	1	0	2	1	1	0	3
0	3	0	0	0	0	0	1	0	0
3	0	3	3	3	0	3	3	0	3
1	1	1	1	1	1	1	1	1	1
3	2	0	0	2	2	1	1	1	0
0	3	3	0	0	3	0	3	0	1
0	3	0	0	0	0	0	0	0	0
0	3	0	0	0	0	0	0	0	0
2	1	0	2	0	1	2	1	0	0
0	1	0	0	0	2	0	0	0	0
0	1	0	0	0	2	0	0	0	0
0	2	0	0	0	1	0	0	0	0
0	2	0	0	0	1	0	0	0	0
1	3	0	0	0	3	0	0	0	0
1	2	0	3	0	0	0	0	0	0
0	3	0	0	3	3	0	0	0	0
0	2	0	0	0	3	0	0	0	0
1	2	0	0	0	0	0	0	0	0
1	2	0	0	0	0	0	0	0	0
0	3	1	0	0	0	0	1	0	1
0	3	3	2	0	2	0	1	0	0
1	3	0	0	0	2	0	1	3	0
0	3	0	0	0	2	0	0	0	0
0	3	0	0	0	0	0	0	0	0
0	3	0	0	0	0	0	0	0	0
0	0	0	0	0	0	0	0	0	0
0	0	0	0	0	0	0	0	0	0
0	3	0	0	0	3	0	0	0	0
0	0	0	0	0	0	0	0	0	0
0	3	0	0	0	3	0	0	0	0

0	3	0	0	0	3	0	0	0	0
0	0	0	0	0	0	0	0	0	0
0	3	0	0	0	3	0	0	0	0
0	1	0	0	0	0	0	0	0	0
0	1	0	0	0	0	0	0	0	0
0	2	0	0	0	0	0	0	0	0
0	3	0	0	0	3	0	0	0	0
0	3	0	0	0	0	0	0	0	0
1	1	3	1	3	1	3	0	1	1
1	0	2	0	0	0	0	0	0	1
0	3	0	0	0	0	0	0	0	0
0	3	0	0	0	0	0	0	0	0
0	0	0	0	0	0	0	0	0	0
1	0	2	3	3	0	1	0	0	1
0	3	0	0	0	0	0	0	0	0
0	3	0	0	0	3	0	0	0	0
0	3	0	0	0	3	0	0	0	0
0	0	0	1	1	0	2	1	0	0
0	0	0	0	0	0	0	0	0	0
0	2	0	0	1	1	2	1	1	2
3	0	3	3	0	0	3	0	0	3
3	0	3	3	0	0	3	0	0	3
0	3	0	0	0	3	0	0	0	0
0	3	0	0	0	3	0	0	0	0
0	3	0	0	0	1	0	0	0	0
1	0	3	1	0	0	3	3	0	3
0	0	0	3	0	0	3	0	0	3
1	1	0	0	0	0	1	0	0	1
0	3	0	0	0	0	0	0	0	0
0	3	0	0	0	0	0	0	0	0
0	0	0	0	0	0	0	0	0	0
0	0	0	0	0	0	0	1	0	0
0	0	0	0	0	0	0	0	0	0
0	3	0	0	0	0	0	3	0	1
0	0	0	0	0	0	0	0	0	0
0	3	0	0	0	3	0	3	0	0
0	3	0	0	0	0	0	0	0	0
3	0	0	3	1	0	1	0	0	1
1	0	1	2	1	0	1	2	0	3
0	1	0	0	0	0	0	0	0	0
0	0	0	0	0	0	0	0	0	0
1	2	2	1	0	1	1	2	1	2
1	3	2	0	3	1	3	1	1	1
0	0	0	0	0	0	0	0	0	0
2	0	1	3	0	0	3	0	0	0
0	1	3	0	0	3	0	2	0	0
1	0	1	2	0	0	1	0	1	2

0	0	0	0	0	0	0	0	0	0
0	0	1	1	1	1	1	1	1	0
0	0	0	0	0	0	0	0	0	0
1	0	0	0	1	1	0	0	0	0
0	0	0	0	0	0	0	0	0	0
0	3	1	0	0	0	0	0	0	0
3	0	3	3	3	0	3	3	3	3
0	0	0	0	0	0	0	0	0	0
1	1	1	1	1	0	1	0	0	0
0	0	0	0	0	0	0	0	0	0
3	3	3	3	3	0	3	3	3	3
0	0	0	0	0	0	0	0	0	0
0	1	0	0	0	0	0	0	0	0
0	0	0	0	0	0	0	0	0	0
0	1	0	0	0	1	0	0	0	0
0	2	0	0	0	3	0	0	0	0
0	1	1	0	0	1	2	0	0	0
1	1	0	1	0	0	1	0	0	0
1	1	0	1	0	1	3	0	0	0
3	1	0	0	1	1	0	0	0	0
0	0	0	0	0	0	0	0	0	0
1	1	2	0	0	0	0	0	0	0
0	3	0	0	0	3	0	0	0	0
0	3	0	0	0	3	0	0	0	0
1	1	2	0	1	2	3	2	1	0
2	1	0	1	2	1	0	0	0	0
0	3	0	0	0	0	0	0	0	0
0	3	0	0	1	2	0	0	1	1
0	3	0	0	0	1	0	0	0	0
0	3	0	0	0	1	0	0	0	0
1	3	0	1	0	0	0	0	0	1
0	2	0	0	0	1	0	1	0	0
0	1	0	2	0	3	0	1	2	0
0	3	0	0	0	0	0	0	0	1
0	2	0	0	0	3	0	0	0	0
1	2	1	1	1	2	1	2	0	0
0	3	0	0	0	0	0	0	0	0
0	3	0	0	0	0	0	0	0	0
0	3	2	0	0	0	0	0	0	0
3	2	0	3	0	2	0	2	0	2
0	3	3	0	3	3	0	0	0	0
0	3	0	0	0	0	0	2	0	0
1	0	3	3	0	0	1	3	1	3
1	0	1	1	1	0	2	1	0	1
2	0	1	1	1	1	2	1	0	1
2	0	1	1	1	1	2	1	0	1
0	1	1	1	1	1	1	1	1	1

0	1	1	1	1	1	1	1	1	1
0	3	0	0	0	3	0	0	0	2
3	1	2	3	3	1	0	1	0	3
0	1	1	0	0	1	0	0	0	1
0	1	1	1	0	1	0	0	0	0
3	0	3	3	3	0	3	0	3	3
1	1	0	0	1	0	2	1	0	1
0	2	0	1	0	2	0	0	0	0
0	1	0	0	0	1	0	0	0	0
0	3	0	0	0	1	0	0	0	0
0	2	0	0	0	0	0	0	0	0
0	2	0	0	0	0	0	0	0	0
0	3	0	0	0	3	0	0	0	0
0	3	0	0	0	2	0	0	0	0
1	0	0	2	1	0	1	1	0	0
2	0	2	0	0	0	1	1	0	0
1	0	1	2	1	0	0	0	1	1
0	1	1	0	1	1	2	0	1	2
0	0	1	0	2	0	0	0	0	1
0	3	0	0	0	2	0	0	0	0
0	2	0	0	0	2	0	0	0	1
0	1	0	0	0	0	0	0	0	0
0	3	0	0	0	1	0	0	0	3
0	3	0	0	3	0	3	0	0	2
1	1	1	3	1	2	0	0	0	0
0	3	0	0	0	3	0	0	0	0
1	1	1	1	1	1	2	2	1	1
1	1	2	0	0	1	0	1	0	2
1	1	2	0	0	1	0	1	0	2
0	2	0	0	0	2	0	0	0	0
0	2	0	2	0	0	1	0	0	3
0	2	1	0	0	0	3	0	3	3
3	0	3	3	0	0	0	0	0	3
1	3	0	1	0	0	0	0	1	2
0	3	0	0	0	0	0	0	0	0
3	0	1	3	0	0	3	0	0	3
0	3	0	0	0	3	0	0	0	0
0	1	1	2	0	1	2	0	1	1
0	3	0	0	0	0	0	0	0	0
0	3	0	0	0	0	0	0	2	0
0	3	1	0	0	3	0	0	0	1
1	3	1	0	0	3	1	1	3	0
1	3	0	1	0	1	1	0	0	1
1	0	2	3	0	1	2	2	0	1
1	0	2	3	0	1	2	2	0	1
0	2	2	1	1	1	0	0	1	0
0	3	0	0	0	3	0	1	0	1

0	3	0	0	0	2	0	0	0	0
2	0	1	1	1	1	2	0	0	0
0	1	0	0	0	2	0	1	2	1
0	2	1	0	0	2	0	1	0	0
0	3	0	0	0	0	0	0	0	0
0	3	0	0	0	0	0	0	0	0
0	3	0	0	0	0	0	0	0	0
0	3	0	0	0	0	0	0	0	0
0	3	0	0	0	0	0	0	0	0
0	3	0	0	0	3	0	0	0	0
0	3	0	0	0	2	0	0	0	1
1	1	2	0	0	1	0	1	0	1
1	0	0	2	0	2	1	1	0	2
0	3	0	0	0	3	0	0	0	0
0	3	0	0	0	3	0	0	0	2
0	3	0	0	0	3	0	0	0	0
1	0	0	0	1	0	1	0	1	0
0	0	0	0	0	0	0	0	0	0
0	0	0	0	0	0	0	0	0	0
0	3	1	0	0	0	0	0	0	0
0	3	0	0	0	0	0	0	0	0
1	1	1	1	1	1	1	1	1	1
0	3	0	3	0	1	0	0	0	0
1	1	1	1	1	1	1	1	1	1
0	3	0	0	0	0	0	0	0	0
0	0	0	0	0	0	0	0	0	0
0	0	0	0	0	0	0	1	1	0
0	3	1	1	1	1	1	1	1	1
0	0	0	0	0	0	0	0	0	0
0	0	0	0	0	0	0	0	0	0
0	0	0	0	0	0	0	0	0	0
1	1	1	1	1	1	1	1	1	1
0	0	0	0	0	0	0	0	0	0
1	3	0	0	0	0	1	0	0	0
1	0	0	0	0	0	0	0	0	0
0	3	0	0	0	0	0	1	0	0
0	3	0	0	0	0	0	0	0	0
0	0	0	0	0	0	0	0	0	0
1	1	1	1	1	1	1	1	1	1
0	0	0	0	0	0	0	0	0	0
0	0	0	0	0	1	0	1	0	0
1	1	1	0	0	1	1	1	1	1
0	0	0	0	0	0	1	3	1	1
1	0	0	0	0	0	0	0	0	0
0	0	1	0	1	0	1	0	1	0
0	0	0	0	1	0	0	1	0	0
0	0	0	0	0	0	0	0	0	0

0	0	1	0	1	0	1	1	1	0
0	3	1	0	0	0	0	0	0	0
0	1	0	0	0	0	0	0	0	0
0	0	0	0	0	0	0	0	0	0
1	1	1	1	1	1	1	1	1	1
0	0	0	0	0	0	0	0	0	0
1	0	1	0	1	0	1	0	1	0
1	1	1	1	1	1	1	1	1	1
0	3	0	0	0	0	0	0	0	1
1	3	0	0	0	0	0	0	0	0
0	3	0	0	0	0	0	0	0	0
0	3	0	0	0	0	0	0	0	0
0	0	0	0	0	0	0	0	0	0
0	1	0	1	1	0	1	0	1	0
0	0	0	0	0	0	0	0	0	0
1	0	1	0	1	0	1	1	1	0
0	0	0	0	0	0	0	0	0	0
0	1	0	1	0	1	0	1	0	0
1	1	1	1	1	1	1	1	1	1
1	1	0	0	0	0	0	0	0	0
3	3	0	0	0	1	0	0	0	0
1	0	1	0	1	0	1	1	0	1
1	0	0	0	0	0	1	0	0	0
0	3	0	0	0	0	0	0	0	0
0	3	0	0	0	3	0	0	0	0
1	1	1	1	1	1	1	1	1	1
1	1	0	1	0	0	1	1	0	0
0	0	0	0	1	0	1	0	0	0
1	0	0	1	0	1	0	1	0	1
1	1	1	1	1	1	1	1	1	1
0	3	0	1	0	0	0	0	0	0
1	1	1	1	1	1	1	1	0	1
1	0	0	0	0	0	0	0	0	0
0	0	0	0	0	0	0	0	0	0
0	0	0	0	0	0	0	0	0	0
1	1	1	1	1	1	0	1	0	1
0	3	0	0	0	0	0	0	0	0
0	2	0	1	0	0	0	0	0	0
0	0	0	0	0	0	0	0	0	0
0	3	0	0	0	0	3	0	0	0
0	0	0	0	0	0	0	0	0	0
1	1	1	1	1	1	1	1	1	1
0	3	0	0	0	0	0	0	0	0
1	3	0	0	0	0	0	0	0	0
1	1	1	1	1	1	1	1	1	1
0	2	0	0	0	0	0	0	2	1
0	3	0	0	0	1	0	0	0	0

3	0	2	2	0	0	3	0	0	0
1	1	1	3	0	1	0	0	0	1
0	1	0	0	0	0	0	0	0	0
3	1	1	1	2	0	2	0	0	1
0	2	0	0	0	0	0	0	0	0
0	0	2	3	2	0	1	0	2	0
0	2	0	0	0	2	0	0	0	0
0	3	0	0	0	1	0	0	0	0
3	0	3	3	2	0	3	3	2	2
1	1	1	1	1	1	0	0	0	0
0	1	1	0	0	0	0	0	0	1
0	1	1	0	0	0	0	0	0	1
0	3	0	1	1	1	0	0	0	0
0	3	0	1	1	1	0	0	0	0
1	1	1	0	0	0	0	0	3	0
0	2	0	0	0	0	0	0	0	0
2	2	0	2	0	2	0	0	0	0
3	0	0	1	1	1	3	0	1	1
2	0	2	3	3	0	2	0	0	2
0	2	1	0	0	1	0	0	0	0
0	3	1	1	1	1	1	0	0	0
0	1	3	3	3	0	2	0	2	2
0	3	0	0	0	1	0	0	0	0
2	2	0	2	0	1	0	1	0	0
1	0	0	0	0	0	0	1	0	1
0	3	0	1	1	1	1	1	1	2
1	3	0	1	0	2	0	0	0	0
2	0	1	1	0	0	0	2	0	1
1	1	1	1	1	1	1	1	1	1
1	1	1	1	1	1	1	1	1	1
3	0	3	3	1	0	3	0	3	3
0	3	0	0	0	3	0	0	0	0
0	3	1	2	0	1	0	1	0	0
0	3	0	0	1	1	0	0	0	0
0	3	0	0	0	1	0	0	0	0
3	1	0	1	0	1	0	1	2	1
0	3	0	0	0	3	0	1	0	0
0	2	0	0	0	2	1	0	0	0
2	0	3	3	3	0	2	0	2	3
1	0	1	3	1	1	3	1	1	1
1	0	1	3	1	1	3	1	1	1
0	1	0	0	0	1	0	0	0	0
1	1	1	1	1	1	1	0	1	1
0	1	0	0	0	0	0	0	0	0
0	1	0	0	0	0	0	0	0	0
0	1	0	0	0	1	0	2	0	0
1	0	1	0	0	0	0	1	0	0

1	1	1	3	1	1	1	1	1	1
0	3	0	0	0	0	0	0	0	0
1	0	1	1	3	0	1	3	1	3
0	3	0	0	0	3	0	0	0	0
0	3	0	0	0	1	0	0	0	0
3	1	0	0	0	0	0	0	0	0
0	1	0	0	0	0	0	0	0	0
0	1	0	0	0	0	0	0	0	0
0	3	0	0	0	3	0	0	0	3
1	3	0	1	0	3	3	0	0	1
1	3	0	1	0	3	3	0	0	1
0	0	1	1	0	0	0	0	0	0
0	3	0	0	0	0	0	0	0	0
1	0	3	3	3	0	3	0	0	3
1	3	0	0	0	1	0	0	0	0
1	3	0	0	0	1	0	0	0	0
0	1	0	0	0	0	0	0	0	0
0	1	0	0	0	0	0	0	0	0
0	0	0	0	0	0	0	0	0	0
3	1	0	0	0	1	0	0	0	0
0	3	0	0	0	3	0	1	0	0
0	1	1	1	3	1	1	1	1	1
0	0	1	1	0	1	0	1	0	0
1	0	1	1	0	1	1	0	0	0
0	3	0	0	0	3	0	2	0	0
0	3	0	0	0	3	0	2	0	0
0	3	0	1	0	3	0	0	0	0
0	3	0	0	0	0	0	0	0	0
1	1	0	1	1	0	0	0	1	0
0	1	0	0	0	1	0	0	0	0
0	1	0	0	0	1	0	0	0	0
0	3	0	0	0	3	0	0	0	0
1	1	0	0	0	1	0	0	0	0
0	0	0	0	0	0	0	0	0	0
0	3	0	0	0	3	0	0	0	0
0	3	0	0	0	3	0	1	0	0
0	0	0	0	0	0	0	0	0	0
0	3	0	0	0	3	0	0	0	0
0	1	0	0	0	2	0	2	0	0
0	1	0	0	0	2	0	2	0	0
0	2	1	0	0	3	0	0	0	0
0	3	0	0	0	3	0	1	0	0
0	3	0	0	0	3	0	0	0	0
0	3	0	0	0	3	0	1	0	0
1	1	1	1	1	1	1	1	1	0
1	1	1	1	1	1	1	1	1	0
1	0	1	1	1	1	1	1	1	1

2	1	0	2	0	0	2	2	0	2
3	0	0	3	3	0	1	0	3	3
0	1	1	0	1	1	0	0	0	0
1	0	1	1	1	0	1	1	1	1
1	1	1	1	1	1	1	1	1	1
1	1	0	0	0	0	0	1	0	0
0	3	0	3	0	3	0	0	0	0
0	0	0	0	0	1	0	1	0	0
2	0	2	3	0	0	0	1	2	1
2	0	2	2	1	0	0	2	0	2
3	0	3	3	0	0	0	0	3	3
0	0	0	0	0	1	0	1	0	0
2	2	0	1	2	1	0	2	1	1
3	0	3	0	0	0	3	0	0	2
0	0	0	0	0	1	3	1	1	0
0	3	0	0	0	3	0	0	0	0
0	0	0	0	0	0	0	0	0	0
1	1	1	1	1	0	1	0	1	1
0	0	0	0	0	0	0	0	0	0
0	3	0	0	0	2	0	0	0	0
1	3	0	0	0	3	0	0	0	0
0	3	0	0	0	3	0	0	0	0
0	3	0	0	0	3	0	0	0	0
2	3	1	3	3	0	0	2	3	3
2	3	1	3	3	0	0	2	3	3
0	0	0	0	0	0	0	0	0	0
1	3	1	1	0	3	0	0	0	0
2	0	2	3	3	0	0	1	3	3
0	1	0	0	0	0	0	0	0	1
0	1	0	0	0	0	0	0	0	1
3	0	0	1	1	0	0	1	1	3
3	0	0	1	1	0	0	1	1	3
0	3	0	0	0	2	0	1	0	0
0	0	0	0	0	0	0	0	0	0
2	1	0	1	0	3	0	0	1	3
2	2	0	2	0	2	0	0	0	2
1	1	1	1	1	1	1	1	1	1
1	0	0	0	0	0	0	0	0	0
0	0	0	0	0	0	0	0	0	0
0	3	0	0	0	3	0	0	0	0
1	0	0	3	0	0	2	0	0	3
0	3	0	0	0	3	0	0	0	0
0	3	0	0	0	3	0	0	0	0
0	2	0	1	0	2	0	0	0	0
0	2	0	0	0	2	0	0	0	0
1	2	0	1	0	2	0	0	0	0
0	3	0	0	0	3	0	0	0	0

0	3	0	0	0	3	0	0	0	0
1	2	1	1	0	2	0	0	0	0
3	0	3	3	0	0	3	3	0	3
2	0	2	3	3	0	2	0	3	2
0	3	0	0	0	3	0	0	0	0
0	3	0	0	0	3	0	0	0	0
2	1	1	2	0	1	0	0	0	2
0	3	0	0	0	3	0	0	0	0
0	3	0	0	0	0	0	0	0	0
1	0	0	3	1	1	0	0	1	1
0	3	0	0	0	3	0	0	0	0
1	1	1	1	0	1	0	0	0	0
1	2	0	1	0	2	0	0	0	1
0	3	0	0	0	0	0	0	0	0
0	2	0	0	0	2	0	0	0	1
0	3	0	0	0	1	0	0	0	0
1	2	0	1	0	2	0	0	0	1
0	3	0	0	0	3	0	0	0	0
0	3	0	0	0	3	0	0	0	0
0	3	0	0	0	3	0	0	0	0
1	3	0	0	0	3	0	0	0	0
0	2	0	0	0	2	0	0	0	0
0	1	0	0	0	1	0	0	0	0
0	2	0	0	0	2	0	0	0	0
0	3	0	0	0	3	0	0	0	0
0	2	0	1	0	2	1	0	0	0
0	3	0	0	0	3	0	0	0	0
0	3	1	1	0	3	0	0	0	0
0	3	0	0	0	3	0	0	0	0
0	3	0	0	0	0	0	0	0	0
0	2	0	1	0	2	0	1	0	0
0	3	1	0	0	1	0	0	0	0
0	3	0	0	0	3	0	0	1	1
0	3	0	0	0	3	0	0	0	0
0	3	0	0	0	3	0	0	0	0
0	3	0	0	0	3	0	0	0	0
1	0	0	2	2	0	3	2	1	2
0	3	0	0	0	3	0	0	0	0
0	2	0	0	1	2	0	0	0	1
0	2	0	1	1	1	0	0	1	1
0	3	0	0	0	3	0	0	0	0
1	1	3	2	0	1	2	0	0	1
0	2	0	1	0	2	0	0	0	0
0	0	0	0	0	3	0	0	0	0
0	3	0	0	0	3	0	0	0	0
0	3	0	0	0	3	0	0	0	0
1	1	1	1	1	1	1	1	1	1

0	2	0	0	0	2	0	0	0	0
0	0	0	0	0	0	0	0	0	0
0	3	0	0	0	3	0	0	0	0
0	1	1	0	0	0	0	0	0	0
0	0	0	0	0	0	0	0	0	0
0	0	0	0	1	1	1	1	1	1
1	1	1	1	0	0	0	0	0	0
0	0	0	0	0	0	0	0	0	0
0	0	0	0	0	0	0	0	0	0
0	0	0	0	0	0	0	0	0	1
0	0	0	0	0	0	0	0	0	0
1	1	1	1	0	1	0	1	0	1
0	0	0	0	0	0	0	0	0	0
0	0	0	0	0	0	0	0	0	0
0	3	0	0	0	0	0	0	0	0
0	3	0	0	0	0	0	0	0	0
0	3	0	0	0	3	0	0	0	0
0	3	0	0	0	0	0	0	0	0
3	0	3	3	3	3	3	2	0	3
3	0	3	3	3	2	3	2	3	3
3	3	0	0	1	0	1	0	1	0
1	3	0	0	0	3	0	1	0	0
0	3	0	0	3	1	0	0	0	0
0	0	1	0	0	1	1	0	0	0
0	3	0	0	0	3	0	0	0	0
0	3	0	1	0	2	0	0	0	0
0	1	0	0	0	1	0	0	0	0
1	1	0	2	0	1	1	2	0	1
0	2	1	1	0	0	0	1	0	1
1	2	2	3	1	2	2	1	1	1
0	3	0	0	0	3	1	1	0	0
0	3	0	0	0	3	0	0	0	0
0	3	0	0	0	3	0	0	0	0
0	2	0	0	0	0	0	0	0	0
0	3	0	0	0	3	0	0	0	0
0	2	0	0	0	2	0	0	0	0
0	3	0	0	0	3	0	0	0	0
0	2	0	0	0	3	0	0	0	0
0	3	0	3	0	3	0	0	0	0
1	0	2	2	0	0	2	2	0	0
0	2	0	0	0	3	0	0	0	0
1	2	1	1	0	2	0	0	0	0
0	3	0	0	0	3	0	0	1	0
0	3	0	3	0	3	0	3	0	0
0	3	0	0	0	3	0	0	0	0
3	3	0	0	0	3	0	0	0	0
0	3	0	3	0	3	0	0	0	0

2	1	2	2	0	3	2	1	1	3
0	3	0	0	0	0	0	0	0	0
0	3	0	0	0	0	0	0	0	0
1	3	1	0	0	2	0	0	0	0
1	3	1	0	0	2	0	0	0	0
3	3	0	0	0	3	0	0	0	0
3	2	0	1	0	0	0	1	1	1
0	3	0	0	0	0	0	0	0	0
0	0	2	2	0	0	2	2	0	0
0	3	0	0	0	3	0	0	0	0
0	3	0	0	0	3	0	0	0	0
0	1	0	0	0	3	0	0	0	0
0	3	0	0	0	2	0	0	0	0
0	1	0	1	1	1	0	0	0	0
0	0	0	0	0	0	0	0	0	0
0	3	0	0	0	0	0	0	0	1
0	0	0	0	0	0	0	0	0	0
0	2	0	0	0	2	0	0	0	0
3	0	3	2	0	0	2	0	0	3
0	3	0	2	0	3	0	0	0	0
0	1	0	1	0	0	1	1	0	0
0	3	0	1	0	0	0	0	0	0
0	0	0	0	0	0	0	0	0	0
0	3	0	0	0	3	0	1	0	0
0	3	0	0	0	3	0	0	0	0
0	3	0	0	0	0	0	1	0	0
1	0	1	1	1	2	0	0	1	3
0	0	0	0	0	0	0	0	0	0
1	3	0	3	1	1	1	1	1	1
0	3	0	0	0	3	0	0	0	0
0	3	0	0	0	3	0	0	0	0
0	0	0	0	0	0	0	0	0	0
0	3	0	0	0	1	0	2	0	2
0	3	0	0	0	3	0	0	0	0
0	1	1	1	0	1	1	0	0	1
0	3	1	0	0	0	0	0	0	0
1	0	2	2	1	0	1	2	0	2
0	3	0	0	0	1	0	0	0	0
0	2	0	0	0	0	0	0	0	0
1	1	0	1	0	1	0	0	0	1
0	3	0	0	1	0	0	1	0	0
2	3	0	0	0	0	0	2	0	0
0	3	0	0	0	0	0	0	0	0
0	3	0	0	0	0	0	0	0	0
0	3	0	0	0	3	0	0	0	0
0	1	0	0	0	1	0	0	0	0
0	0	3	3	0	0	0	0	0	3

0	3	1	0	0	0	0	0	0	3
0	3	0	0	0	3	0	0	0	0
0	3	0	0	0	2	0	0	0	0
0	3	0	0	0	1	0	0	0	0
0	3	0	0	0	1	0	0	0	0
0	3	0	0	0	1	0	0	0	0
0	3	0	0	0	1	0	0	0	0
0	3	0	0	0	3	0	0	0	0
0	3	2	0	0	2	0	1	0	1
0	0	0	0	0	0	0	0	0	0
0	3	3	1	1	0	0	0	0	1
2	2	0	2	0	0	0	0	0	0
0	3	0	0	0	3	0	0	0	0
0	3	0	1	0	3	3	1	0	0
0	0	0	0	0	0	0	0	0	0
0	0	0	0	0	0	0	0	0	0
2	1	0	0	0	3	0	1	1	2
1	1	1	1	1	2	0	1	0	0
3	0	3	3	3	0	3	3	3	3
1	1	1	3	1	0	3	2	0	0
2	1	2	1	2	1	2	1	1	1
0	0	0	0	0	0	0	0	0	0
0	0	0	0	0	0	0	0	0	0
3	3	3	3	3	0	3	3	3	3
3	0	3	3	3	3	3	3	3	3
3	0	3	3	3	0	3	3	3	3
2	0	2	0	2	1	2	2	2	2
0	0	0	0	0	0	0	0	0	0
0	0	0	0	0	0	0	0	0	0
0	3	0	1	0	3	0	0	0	0
0	0	0	0	0	0	0	0	0	0
0	0	0	0	0	0	0	0	0	0
0	0	0	0	0	0	0	0	0	0
0	0	0	0	0	0	0	1	0	0
0	0	0	0	0	0	0	0	0	0
3	1	1	3	0	0	3	1	3	3
0	0	0	0	0	0	0	0	0	0
0	1	0	0	0	0	0	0	0	0
0	1	1	1	0	1	0	1	0	0
0	3	0	0	0	0	0	0	0	0
1	1	1	0	0	3	0	0	0	2
0	2	0	0	0	1	0	0	0	1
0	3	0	0	0	3	0	0	0	0
0	3	0	0	0	3	0	0	0	0
0	3	0	3	0	1	3	0	0	0
0	3	0	0	0	3	0	0	0	0
0	1	0	0	0	0	0	1	0	0

1	1	1	1	1	1	1	0	0	0
0	3	0	0	0	1	0	0	0	0
0	2	1	1	0	2	0	0	0	1
0	3	0	0	0	2	0	2	0	0
0	1	2	1	0	1	2	1	0	1
3	0	1	3	2	0	3	3	0	3
0	0	0	0	0	0	0	0	0	0
1	2	0	1	2	1	0	1	2	1
0	3	0	0	0	0	0	0	0	0
0	0	0	1	1	2	1	0	0	0
3	0	2	3	0	1	1	1	0	3
0	0	0	3	0	0	0	0	0	0
3	1	0	3	0	0	3	0	0	3
0	0	0	0	0	0	0	0	0	0
0	1	0	0	1	2	1	0	1	2
0	3	0	0	0	0	0	0	0	0
0	3	3	0	1	3	0	1	1	3
0	3	0	0	0	0	0	0	0	0
1	0	0	2	2	0	3	0	0	3
0	3	0	1	0	3	0	0	0	0
0	3	0	0	0	3	0	0	0	0
0	1	2	1	2	1	0	1	0	0
0	3	0	0	0	0	0	0	0	0
3	1	1	3	1	0	1	0	0	2
2	2	1	0	0	1	2	0	0	0
0	3	2	1	0	0	0	0	1	3
1	1	0	1	1	1	0	0	1	1
0	3	0	0	3	3	0	0	0	0
0	1	0	0	0	0	3	0	0	0
1	0	0	1	0	1	0	3	0	0
0	3	0	0	0	3	0	0	0	0
3	0	3	3	3	0	3	3	2	3
2	3	1	2	3	1	2	0	0	0
0	3	0	0	0	3	0	0	0	0
1	1	1	0	0	0	0	1	1	1
0	0	1	3	0	0	2	0	0	3
0	0	0	0	0	0	0	0	0	0
3	1	0	0	0	0	1	0	0	0
0	0	0	0	0	1	0	1	3	3
1	2	0	1	0	2	1	0	0	0
0	3	0	0	0	3	0	0	0	0
0	1	0	1	0	0	0	1	0	0
1	0	0	0	0	1	1	0	0	1
1	3	0	0	0	1	1	0	0	0
0	0	0	0	0	0	0	0	0	0
0	0	0	0	0	0	0	0	0	0
1	0	1	0	1	0	1	0	0	0

0	1	0	1	0	1	0	1	0	1
0	0	0	0	0	0	0	0	0	0
1	0	0	1	1	1	1	1	1	1
0	3	0	0	0	0	0	0	0	0
0	0	0	0	0	0	0	0	0	0
0	0	0	0	0	0	0	0	0	0
1	1	1	1	1	1	1	1	1	1
1	1	1	1	1	1	1	1	1	1
0	0	0	1	1	1	1	1	1	1
0	0	0	0	0	0	0	0	0	0
1	1	2	1	0	0	0	0	0	0
1	1	0	0	0	0	0	0	0	0
0	1	0	0	0	0	0	0	0	0
1	0	1	1	0	0	0	0	0	1
1	1	1	1	1	1	1	1	1	1
0	0	0	0	0	0	0	0	0	1
0	3	0	0	0	2	0	0	0	0
0	0	0	0	0	1	0	0	0	0
0	3	1	1	1	1	0	0	0	0
0	0	0	0	0	0	0	0	0	0
0	0	0	0	0	0	0	0	0	0
0	0	0	0	0	0	0	0	1	0
1	1	1	1	0	1	0	1	1	1
0	0	0	0	0	0	0	0	0	0
0	1	1	0	0	0	0	0	0	0
0	0	0	0	0	0	0	0	0	0
0	1	1	1	0	1	1	1	1	1
2	1	3	1	1	1	1	1	1	1
3	1	3	2	2	0	1	1	1	2
0	0	0	0	0	0	0	0	0	0
0	0	0	0	0	0	0	0	0	0
0	0	0	0	0	0	0	0	0	0
0	0	0	0	0	0	0	0	0	0
1	1	1	1	1	1	1	3	1	1
1	0	1	0	0	0	0	0	0	0
0	1	0	0	0	1	3	0	0	0
0	0	0	0	0	0	0	0	0	0
0	0	0	1	1	1	1	1	1	0
0	0	0	0	0	0	0	0	0	0
0	0	0	0	0	0	0	0	0	0
3	0	3	3	0	0	3	3	0	3
1	2	0	3	1	1	1	1	1	1
3	0	0	3	3	0	3	0	0	3
2	0	2	3	2	0	2	0	2	3
3	0	1	1	0	0	1	1	0	1
3	0	1	1	0	0	1	1	0	1
1	3	0	1	1	0	1	0	3	3

1	3	0	1	1	0	1	0	3	3
1	3	1	0	0	3	1	3	1	0
1	3	1	0	0	3	1	3	1	0
0	3	0	0	0	3	0	3	0	0
0	3	0	0	0	3	0	3	0	0
0	2	0	0	0	2	0	0	0	0
0	3	1	0	0	1	0	0	0	0
0	3	0	1	1	1	1	0	0	3
2	0	2	3	3	0	3	0	2	2
1	0	1	1	0	0	0	2	0	1
0	3	0	0	0	0	0	1	0	1
3	0	0	3	0	0	3	0	0	1
2	2	1	1	0	1	0	1	0	0
2	0	3	2	2	0	2	0	2	2
3	3	1	1	1	0	0	0	0	0
1	3	0	0	0	3	0	0	0	0
3	0	3	3	1	0	3	0	3	3
0	3	0	0	0	3	0	0	0	0
0	3	0	1	0	3	0	0	0	0
3	1	1	0	0	1	0	1	0	1
0	1	1	0	0	0	0	0	0	0
2	0	2	0	1	0	0	0	3	3
1	0	1	2	0	1	1	0	0	3
1	1	1	1	1	1	1	1	1	1
0	1	1	1	0	0	0	0	0	0
0	0	0	0	0	0	0	0	0	0
0	1	0	1	0	3	0	0	0	0
0	1	0	0	0	1	0	0	0	0
0	3	0	0	0	0	0	0	0	0
0	1	0	0	0	1	0	0	0	0
0	3	0	0	0	3	0	0	0	0
0	1	0	1	0	0	3	0	1	1
0	1	0	0	0	0	0	0	0	0
0	1	1	0	0	0	0	0	0	0
0	3	0	0	0	3	0	0	0	0
0	0	0	0	0	0	0	0	0	0
0	3	0	0	0	0	0	0	0	0
1	0	1	1	0	0	0	0	0	1
0	3	0	1	0	1	0	0	0	1
1	1	1	1	0	0	0	0	0	0
0	3	0	1	0	1	1	0	0	1
0	3	0	0	0	0	0	0	0	0
0	3	0	0	0	0	0	0	0	0
0	0	0	0	0	0	0	0	0	0
1	0	0	1	0	0	1	1	0	1
2	0	0	1	1	0	0	0	0	0
0	1	0	0	0	0	0	0	0	0

0	1	0	0	0	0	0	0	0	0
0	3	0	0	0	0	0	1	0	0
0	1	0	0	0	0	0	0	0	0
1	2	0	0	0	3	0	0	0	2
0	1	0	0	0	3	0	0	0	0
0	3	0	1	0	0	0	1	0	1
1	0	1	1	0	0	3	1	3	3
0	1	0	0	0	0	0	0	0	0
3	1	1	1	0	1	0	1	0	1
1	1	1	1	1	1	1	1	1	1
1	1	1	1	1	1	1	1	1	1
1	2	0	1	0	1	0	0	0	0
0	3	0	0	0	3	0	0	0	0
1	3	0	1	0	0	1	1	1	1
0	1	0	0	0	0	0	0	0	0
0	3	0	0	0	3	0	0	0	0
0	0	0	0	0	0	0	0	0	0
0	0	0	0	0	0	0	0	0	0
0	3	0	0	0	3	0	0	0	0
1	3	0	1	0	3	0	0	0	1
0	3	0	0	0	0	0	0	0	0
0	3	0	0	0	3	0	0	0	0
0	0	0	0	0	0	0	0	1	0
0	3	0	0	0	3	0	0	0	0
0	0	1	0	0	0	0	0	0	0
2	0	2	2	0	2	2	0	0	2
2	2	0	0	0	2	2	2	2	0
3	1	0	1	3	1	1	0	1	0
0	1	1	0	1	0	0	3	0	0
0	3	0	0	0	2	0	0	0	0
3	0	2	3	0	0	0	0	0	3
0	0	0	0	0	0	0	0	0	0
0	0	0	0	0	0	0	0	0	1
0	2	1	0	0	1	0	0	0	0
0	2	0	0	0	2	0	0	0	0
0	3	0	0	0	3	0	0	0	0
0	3	0	0	0	3	0	0	0	0
3	2	1	0	0	0	0	0	0	0
0	3	0	0	0	1	0	0	0	0
0	0	0	0	0	1	0	1	0	0
2	1	1	1	1	1	0	1	0	0
1	1	0	1	1	0	0	1	0	1
0	0	0	0	0	0	0	0	0	0
0	0	0	0	0	0	0	0	0	0
0	0	0	0	0	0	0	0	0	0
0	2	0	0	0	2	0	0	0	3
0	1	3	0	2	0	0	0	0	0

0	3	0	0	0	0	0	0	0	0
3	0	0	3	1	1	3	3	1	0
0	3	0	0	0	0	0	0	0	0
0	2	0	0	0	1	0	1	0	0
0	1	0	0	0	1	0	0	0	0
0	3	0	0	0	3	0	0	0	0
0	3	1	0	0	3	0	3	0	1
0	3	0	0	0	3	0	0	0	0
0	2	0	0	0	2	0	0	0	0
0	3	0	0	0	3	0	0	0	0
0	3	0	0	0	3	0	0	0	0
0	3	0	0	0	3	0	1	0	0
0	3	0	0	0	2	0	0	0	0
0	3	0	0	0	0	0	0	0	0
0	3	0	0	0	3	0	0	0	0
0	3	0	0	0	3	0	0	0	0
0	3	0	0	0	3	0	0	0	0
1	2	0	0	0	2	0	1	0	1
0	3	0	0	0	3	0	0	0	0
3	0	1	2	1	0	3	0	1	2
3	1	0	1	1	0	3	3	3	1
0	3	0	0	0	3	0	0	0	0
0	3	0	0	0	3	0	0	0	0
1	1	0	2	1	1	0	0	1	3
0	3	0	0	0	2	0	0	0	0
2	0	1	3	1	0	2	2	1	1
0	3	0	0	0	3	0	0	0	0
0	1	0	1	0	1	1	0	0	0
0	2	0	0	0	3	0	0	0	0
0	3	0	0	0	3	0	0	0	0
1	3	0	0	0	3	0	1	0	0
1	1	0	1	0	1	0	0	1	1
0	3	0	0	0	3	0	0	0	0
0	2	0	1	0	2	0	0	0	0
0	2	0	1	0	2	0	0	0	0
0	3	0	0	0	3	0	0	0	0
1	1	0	0	0	1	0	0	0	0
1	1	1	0	0	0	0	0	0	0
1	2	0	1	2	2	0	1	1	2
0	3	0	0	0	0	0	0	0	0
1	2	1	1	1	1	1	1	1	1
0	3	0	0	0	3	0	0	0	0
0	3	0	0	0	3	0	1	0	0
1	3	0	0	0	3	0	0	0	0
0	3	0	0	0	3	0	0	0	0
1	2	1	1	1	1	1	2	0	1
0	2	1	1	0	2	0	0	0	0

0	2	1	0	0	2	0	0	0	0
1	3	0	0	0	3	0	0	0	1
1	0	0	0	1	1	0	0	0	0
0	3	3	1	0	3	0	0	0	0
0	0	0	0	0	0	0	0	0	0
0	1	2	0	1	1	0	0	0	0
1	1	0	0	0	0	0	0	0	0
1	0	3	1	3	3	3	2	3	0
3	0	1	0	0	3	3	3	3	3
2	3	0	0	0	3	0	0	0	0
0	3	0	0	0	3	0	0	0	0
3	0	3	3	0	1	3	3	1	1
0	0	0	0	0	0	0	0	0	0
0	3	0	0	0	0	0	0	0	0
0	0	0	0	0	0	0	0	0	0
0	3	0	0	0	0	0	0	0	0
0	0	0	0	0	3	0	0	0	0
0	0	0	0	0	0	0	0	0	0
1	1	1	1	1	1	1	1	0	0
0	3	0	0	0	3	0	0	0	0
0	3	0	0	0	3	0	0	0	0
0	3	0	0	0	3	0	0	0	0
0	0	0	0	0	1	1	1	1	0
3	0	3	3	3	3	3	3	3	0
0	3	0	0	0	0	0	0	0	0
0	1	0	0	1	3	0	0	0	0
1	1	1	1	3	1	1	1	1	2
0	1	0	0	0	0	0	0	0	0
1	1	3	1	0	1	2	3	0	1
0	3	0	0	0	2	0	0	0	0
0	3	0	0	0	3	0	0	0	0
0	3	0	0	0	0	0	2	0	0
0	3	0	0	0	2	0	0	0	0
3	0	3	3	3	0	3	3	3	0
0	2	0	0	0	0	0	1	0	0
0	3	3	0	0	3	0	0	0	0
0	2	0	0	0	2	0	0	0	0
3	0	3	3	3	0	1	1	3	3
0	3	0	0	0	3	0	0	0	0
3	1	1	1	1	1	1	1	0	1
0	3	0	0	0	0	0	0	0	0
0	3	0	0	0	1	0	0	0	0
3	3	0	0	1	0	0	3	1	1
0	0	0	0	0	0	0	0	0	0
0	3	0	0	0	0	0	0	0	0
0	2	0	0	0	2	0	2	0	0
0	3	0	0	0	0	0	1	0	0

3	1	1	3	0	3	3	0	0	0
0	2	1	0	0	3	0	1	0	0
3	1	3	1	0	0	0	1	0	1
0	3	0	0	0	3	0	0	0	0
0	3	0	0	0	3	0	0	0	0
0	0	0	0	0	0	0	1	0	0
0	3	0	0	0	3	0	0	0	0
0	3	0	0	0	0	0	0	0	0
0	0	0	0	0	0	0	0	0	0
1	3	1	1	1	0	1	0	0	1
0	3	0	2	0	0	0	0	0	0
0	3	0	0	0	0	0	0	0	0
0	1	0	0	0	2	0	0	0	0
0	0	0	0	0	0	0	0	0	0
1	0	1	2	1	0	3	2	1	1
0	3	0	0	0	0	0	0	0	0
0	0	0	0	0	0	0	0	0	0
1	0	1	1	0	3	3	3	0	1
0	3	0	1	0	0	0	1	0	0
0	0	1	0	0	0	2	2	0	0
1	0	0	1	0	0	0	0	0	3
0	0	0	0	0	0	0	0	0	0
0	0	0	0	0	0	0	0	0	0
0	0	0	0	0	0	0	0	0	0
3	1	3	1	0	0	0	2	1	1
1	2	0	0	0	0	0	1	0	1
2	1	2	2	0	1	0	1	0	1
0	3	0	0	0	0	0	0	0	1
0	2	0	0	0	0	0	0	0	0
1	2	0	0	0	0	0	1	0	1
1	1	0	1	0	1	0	1	0	1
0	0	0	0	0	0	0	0	0	0
0	0	0	0	0	0	0	0	0	0
0	0	0	0	0	0	0	0	0	0
0	0	0	0	0	0	0	0	0	0
0	0	0	0	0	0	1	1	0	0
2	0	2	2	2	1	1	1	0	0
0	0	0	0	0	0	0	0	0	0
0	0	0	0	0	0	0	0	0	0
0	1	0	0	0	1	0	0	0	0
0	1	0	2	0	0	0	0	0	0
0	2	0	0	0	2	0	1	0	0
0	3	0	0	0	3	0	0	0	1
0	3	0	0	0	2	0	0	0	1
3	2	1	2	0	2	1	2	0	0
0	0	0	0	0	0	0	0	0	0
0	2	0	1	0	1	0	2	0	1

0	3	0	1	0	0	0	0	0	0
1	1	1	1	1	0	1	0	1	3
0	0	0	0	0	0	0	0	0	0
0	0	0	0	0	0	0	0	0	0
0	3	0	0	0	0	1	0	0	3
1	1	0	0	0	0	0	1	2	0
0	0	0	0	0	0	0	0	0	0
0	0	0	0	0	0	0	0	0	0
0	3	2	0	2	3	0	0	0	0
0	1	0	0	0	1	0	1	0	0
2	0	1	2	0	1	2	0	0	1
0	3	0	0	0	0	0	0	0	0
3	0	3	3	3	0	3	1	3	3
3	0	3	3	1	0	3	1	1	3
0	0	0	0	1	1	1	1	1	0
0	3	0	1	1	1	0	0	0	0
0	3	0	0	0	0	0	0	0	0
2	2	2	0	2	1	2	1	2	1
2	2	2	0	2	1	2	1	2	1
0	3	0	0	0	3	0	0	0	0
1	2	3	1	2	3	1	3	1	2
0	3	0	0	0	3	0	1	0	0
1	0	1	0	0	0	0	0	0	0
0	0	0	0	0	0	0	0	0	0
0	3	0	0	0	0	0	0	0	3
1	0	1	1	1	1	1	0	0	0
3	1	3	3	3	0	3	0	0	0
0	0	1	0	3	1	1	0	0	0
3	1	1	1	1	1	1	1	1	1
0	3	1	1	0	0	0	0	0	0
1	1	1	1	1	1	1	1	1	1
0	3	3	1	0	0	2	1	0	0
0	0	0	0	0	0	0	0	0	0
1	1	1	1	1	1	0	1	0	0
1	1	1	1	1	1	1	1	1	1
0	3	0	0	0	0	0	0	0	0
1	3	0	0	0	0	0	0	0	0
1	3	0	1	0	0	1	0	0	0
1	1	1	1	0	0	0	0	1	1
1	1	1	1	1	1	1	1	1	1
1	0	0	0	0	0	0	0	0	0
1	1	1	1	1	1	0	0	0	0
0	0	1	1	0	1	1	1	1	1
1	1	1	1	1	1	1	1	1	1
0	3	0	0	0	1	1	0	0	0
1	1	1	1	1	1	1	1	1	0
1	3	0	0	0	0	0	0	0	0

0	0	0	0	0	0	0	0	0	0
1	1	0	0	0	0	0	0	0	0
3	3	1	3	0	1	1	1	0	1
0	1	0	0	0	1	0	0	0	0
0	2	2	2	0	0	0	2	0	0
3	0	0	1	0	0	1	0	0	1
0	3	3	0	0	2	0	0	0	0
0	3	3	0	0	2	0	0	0	0
2	0	3	3	0	0	0	0	0	1
0	2	0	0	0	2	0	0	0	0
0	0	0	2	0	0	0	1	0	3
2	0	3	3	0	0	3	0	0	2
1	0	3	3	2	0	3	0	3	3
0	3	1	0	0	3	0	0	0	0
0	3	0	0	0	1	0	0	0	0
0	1	0	0	0	0	0	0	0	0
0	1	0	0	0	0	0	0	0	0
0	3	0	0	0	0	0	0	0	0
0	3	0	0	0	0	0	0	0	0
1	1	1	1	1	1	1	1	1	1
0	3	0	0	0	0	0	0	0	0
1	3	0	1	0	3	1	0	0	0
0	1	0	1	0	0	0	0	0	0
1	0	1	0	0	0	0	0	0	0
0	1	0	0	0	3	0	0	0	0
0	1	1	0	0	1	0	1	0	1
1	0	0	1	0	0	1	1	0	1
1	1	1	1	1	1	1	1	1	1
0	1	0	1	0	0	0	0	0	0
1	0	0	1	0	0	3	2	0	1
0	1	0	0	0	0	0	0	0	0
3	3	0	0	0	3	0	0	0	0
0	1	0	3	0	0	0	1	0	1
0	1	0	0	0	0	0	0	0	0
0	1	0	0	0	0	0	0	0	0
1	3	0	0	0	0	0	0	0	2
3	0	1	3	2	0	1	0	0	2
0	3	0	0	0	0	0	0	0	0
0	3	0	0	0	0	0	0	0	0
0	3	0	0	0	0	0	0	0	0
0	3	0	0	0	3	0	0	0	0
0	1	0	0	0	3	0	0	0	0
0	3	0	0	0	3	0	0	0	0
2	0	3	0	0	0	0	3	0	3
0	0	0	0	0	0	0	0	0	0
0	3	0	0	0	3	0	0	0	0
0	0	0	0	0	0	0	1	0	0

0	3	1	1	0	3	0	1	0	3
0	3	0	0	0	3	0	0	0	0
1	3	0	1	0	3	0	0	0	0
1	0	0	0	0	0	0	1	0	0
1	0	1	1	0	0	0	0	0	1
1	1	1	0	0	1	1	1	1	1
0	0	0	0	0	0	0	0	0	0
0	3	0	0	0	3	0	0	0	0
0	3	0	0	0	3	0	0	0	0
0	2	0	1	0	2	0	1	0	0
0	0	0	0	0	0	0	0	0	0
0	0	0	0	0	0	0	0	0	0
1	3	3	0	0	0	0	0	0	0
0	1	2	0	3	1	0	1	0	2
0	3	0	0	0	3	0	0	0	0
0	3	0	0	0	3	0	0	0	0
0	3	0	0	0	3	0	0	0	0
0	3	0	0	0	3	0	0	0	0
1	2	0	0	0	2	0	3	0	1
3	0	0	3	1	0	3	3	1	2
0	3	0	0	0	3	0	1	0	0
0	3	0	0	0	3	0	0	0	0
0	2	2	1	0	2	0	2	1	2
0	3	0	0	0	3	0	0	0	0
0	2	0	1	1	2	0	1	0	0
0	2	0	0	0	2	0	0	0	1
2	0	1	1	0	1	0	0	0	0
1	2	1	0	0	2	0	0	0	2
0	3	0	0	0	3	0	0	0	0
0	3	0	0	0	3	0	0	0	0
0	3	0	0	0	3	0	0	0	0
0	3	0	0	0	0	0	0	0	0
0	3	0	0	0	3	0	0	0	0
0	2	0	0	0	3	0	0	0	0
0	3	0	0	0	3	0	0	0	0
0	2	0	0	0	0	0	0	0	0
0	2	0	0	0	0	0	0	0	0
0	3	0	0	0	0	0	0	0	0
0	3	0	0	0	2	0	0	0	0
1	2	0	1	0	0	0	0	0	0
1	1	1	1	1	1	1	0	0	0
1	2	0	0	0	2	0	0	0	1
0	3	0	0	0	3	0	0	0	0
0	3	0	0	0	3	0	0	0	0
0	3	0	0	0	3	0	0	0	0
0	3	0	0	0	3	0	0	0	0
0	2	0	1	0	2	0	1	0	0

0	2	1	1	0	1	0	0	1	0
0	3	0	0	0	3	0	0	0	0
0	3	0	0	0	3	0	0	0	0
0	3	0	0	0	3	0	0	0	0
0	3	0	0	0	3	0	0	0	0
0	3	0	0	0	3	0	0	0	0
0	0	0	0	0	1	0	0	0	0
3	0	3	3	0	0	3	3	3	3
0	0	0	0	0	0	0	0	0	0
0	3	0	0	0	0	0	0	0	0
0	3	0	0	0	0	0	0	0	0
0	3	0	0	0	0	0	0	0	0
1	0	0	0	0	0	0	1	1	1
0	3	0	0	0	3	0	0	0	0
0	3	0	0	0	3	0	0	0	0
0	0	0	0	0	0	0	0	0	0
1	0	1	1	1	1	1	1	1	1
0	0	0	0	0	0	0	0	0	0
1	0	1	1	1	1	0	0	0	0
0	3	0	0	0	3	0	0	0	0
0	1	0	1	0	1	0	1	0	1
1	2	1	0	0	1	2	2	0	0
0	3	0	0	0	2	0	2	0	0
0	2	1	0	2	2	1	0	0	0
0	3	1	0	0	2	0	0	0	0
0	3	0	0	0	3	0	0	0	0
3	0	3	3	0	0	3	3	0	3
3	1	3	2	0	1	2	3	1	3
2	2	1	1	1	2	1	3	1	3
2	0	3	0	1	1	0	2	1	0
0	3	0	0	0	3	0	0	0	0
0	0	0	0	0	0	0	0	0	0
0	3	0	0	0	3	0	0	0	0
3	0	3	3	3	0	3	3	3	3
0	2	1	1	0	2	0	2	0	1
0	1	0	0	0	2	0	0	0	0
1	1	1	1	1	1	3	0	0	0
0	0	0	0	0	0	0	0	0	0
0	3	1	0	1	3	0	0	0	1
3	0	3	3	3	0	3	2	3	3
0	3	0	0	0	3	0	0	0	0
1	3	0	0	0	3	0	0	0	0
2	1	0	1	1	1	0	0	0	1
3	0	3	3	0	0	3	3	1	3
0	3	3	0	3	3	0	0	0	0
0	1	0	0	0	3	0	0	0	0
0	3	0	0	0	0	0	0	0	0

0	3	0	0	0	3	0	0	0	0
0	1	0	0	0	0	0	0	0	2
0	2	0	0	0	1	0	0	0	0
0	0	0	0	0	0	0	0	0	0
0	3	0	0	0	3	0	0	0	0
0	0	0	0	0	0	0	0	0	0
2	3	0	2	1	0	0	0	0	0
1	0	1	3	0	0	1	3	0	1
0	1	0	0	0	2	0	0	0	0
0	0	0	0	0	0	0	0	0	0
0	3	0	0	0	3	0	0	0	0
0	1	1	0	0	0	0	0	0	1
0	3	1	0	0	3	0	0	0	0
3	0	3	3	3	0	0	0	0	0
0	0	0	0	0	0	0	0	0	0
0	1	0	0	0	1	0	1	1	1
0	0	0	0	0	0	0	0	0	0
0	0	0	0	0	0	0	0	0	0
0	3	1	0	0	3	0	0	0	0
0	3	0	3	0	3	0	0	0	0
0	1	1	1	1	1	1	0	1	0
1	0	1	0	0	1	0	1	0	1
0	0	0	0	0	0	0	0	0	0
1	0	1	0	1	3	3	2	0	0
0	1	2	0	0	3	0	0	0	0
0	0	0	0	0	0	0	0	0	0
0	1	0	0	0	0	0	0	0	0
1	0	0	0	0	0	0	0	0	0
0	0	0	0	0	1	1	1	1	1
0	0	0	0	0	0	0	0	0	0
0	3	3	0	0	1	0	0	0	0
1	1	0	1	0	1	0	1	0	2
0	0	0	0	0	0	0	0	0	0
3	2	1	1	1	0	0	1	0	1
0	0	0	0	0	0	1	0	0	1
0	0	3	3	0	0	0	0	0	3
0	1	2	0	0	2	0	0	0	1
0	3	0	0	0	0	0	0	0	0
0	3	0	0	0	0	0	0	0	0
0	3	0	0	0	3	0	0	0	0
0	2	0	2	0	1	0	1	0	1
1	0	0	1	2	1	1	0	1	2
3	0	3	3	0	0	3	3	3	3
0	1	0	1	1	1	1	3	2	1
0	1	3	3	0	3	3	0	0	0
0	3	0	0	0	0	0	0	0	0
0	3	0	0	0	0	0	0	0	0

0	3	0	0	0	0	0	0	0	0
0	3	0	0	0	3	0	0	0	0
0	3	0	0	0	3	0	0	0	0
0	3	0	0	0	0	0	0	0	0
0	3	1	0	0	0	0	0	0	0
0	3	2	3	0	2	0	0	0	0
0	2	0	0	0	2	1	0	0	0
2	1	0	1	1	0	1	2	0	1
1	1	1	1	1	1	1	1	1	0
1	1	1	1	1	0	0	0	0	0
0	0	0	0	0	0	0	0	0	0
1	1	1	1	1	1	1	1	1	0
0	0	0	0	0	0	0	0	0	0
1	1	1	1	0	0	1	1	0	0
0	3	0	0	0	0	1	0	0	0
0	0	0	0	0	0	0	0	0	0
0	0	0	0	0	0	0	0	0	0
0	3	0	0	0	0	0	0	0	0
1	1	1	1	1	1	1	1	1	0
1	1	1	1	1	1	1	1	1	0
0	1	0	0	0	0	0	0	0	0
0	0	0	1	1	1	0	1	0	1
1	1	1	0	1	0	0	0	0	3
0	0	0	0	0	0	0	0	1	1
0	0	1	1	0	0	0	0	0	1
0	2	0	0	0	2	0	0	0	0
1	1	1	1	1	1	1	1	1	0
0	3	0	0	0	0	0	0	0	1
3	0	0	1	0	0	3	0	0	3
1	1	1	1	1	1	0	1	0	0
1	1	1	1	1	1	0	0	0	1
3	0	2	3	0	1	0	3	0	0
3	0	0	3	0	0	3	0	0	0
3	0	2	2	0	0	2	0	0	3
1	1	0	0	3	0	0	0	0	0
0	3	3	0	0	3	0	1	0	0
1	1	1	1	1	1	0	1	0	1
1	1	1	1	1	1	1	1	0	0
1	3	0	0	0	0	0	3	0	0
0	1	0	0	0	0	0	0	0	0
1	1	1	1	1	1	0	0	0	1
3	0	1	1	1	0	1	2	1	1
0	1	0	0	0	0	0	0	0	0
1	0	1	1	0	0	1	1	0	1
1	1	1	1	1	1	1	1	1	1
0	1	0	0	0	0	0	0	0	0
0	1	0	1	0	1	1	1	1	1

0	1	0	0	0	0	0	0	0	0
0	1	0	0	0	0	0	0	0	0
0	1	0	0	0	0	0	0	0	0
0	1	0	0	0	0	0	0	0	0
0	1	1	0	0	0	0	0	0	0
0	3	0	0	0	1	0	0	0	0
0	1	0	0	0	0	0	0	0	0
0	3	0	0	0	0	0	0	0	0
0	3	0	0	0	0	0	0	0	0
0	1	0	1	0	1	0	0	0	0
0	1	0	1	0	1	0	0	0	0
1	1	1	1	1	1	1	1	0	1
1	1	1	1	1	1	1	1	0	1
1	0	3	2	3	0	0	3	0	2
0	3	1	1	0	3	0	1	0	0
0	0	0	0	0	0	0	0	0	0
3	1	0	3	0	0	1	0	0	3
0	2	0	0	0	2	0	0	0	0
1	3	0	0	0	2	0	0	0	0
2	0	0	1	0	1	0	0	0	2
0	2	1	1	1	1	0	0	0	0
0	0	0	0	1	0	1	1	1	0
2	3	0	0	0	3	1	3	0	1
0	0	0	0	0	0	0	0	0	0
0	3	0	0	0	3	0	0	0	0
0	3	2	0	0	3	0	0	0	0
0	3	0	0	0	0	0	0	0	0
0	3	1	1	1	0	0	0	0	0
0	1	1	0	0	1	0	0	0	1
0	3	0	0	0	3	0	0	0	0
0	3	0	0	0	3	0	0	0	0
0	2	0	0	0	2	0	0	0	0
0	1	0	0	0	1	0	0	0	0
0	3	0	0	0	3	0	0	0	0
0	3	0	0	0	2	0	0	0	2
0	2	0	0	0	0	0	3	0	0
0	2	0	0	0	0	0	0	0	0
0	3	0	0	0	3	0	0	0	0
0	2	0	0	0	2	0	0	0	0
0	3	0	0	0	3	0	0	0	0
1	1	1	1	0	1	0	0	0	0
0	2	0	0	0	2	0	0	0	1
2	2	0	2	0	1	0	1	0	2
0	3	0	0	0	2	0	0	0	0
0	3	0	0	0	3	0	0	0	0
0	3	0	0	0	3	0	0	0	0
0	2	0	0	0	3	0	0	0	0

1	1	0	1	0	2	0	0	0	0
2	1	0	2	0	1	1	0	0	1
0	1	0	0	0	3	0	0	0	0
0	3	0	0	0	3	0	0	0	0
1	2	0	0	0	3	0	0	0	0
1	1	1	1	0	1	0	0	0	0
0	3	0	0	0	3	0	0	0	0
0	3	0	0	0	3	0	0	0	0
1	1	1	1	0	1	0	0	0	0
0	0	0	0	0	1	1	1	1	0
1	0	0	0	0	1	1	3	3	1
0	0	0	0	0	0	0	0	0	0
0	3	0	0	0	3	0	0	0	0
0	0	0	0	0	0	0	0	0	0
0	3	0	0	0	0	0	0	0	0
0	0	0	0	0	0	0	0	0	0
0	3	0	0	0	3	0	0	0	0
0	0	0	0	0	0	0	0	0	0
1	1	1	1	1	1	1	1	0	0
0	3	1	0	0	3	0	1	0	0
0	3	0	0	0	3	0	0	0	0
0	3	1	1	3	1	0	0	0	0
0	1	1	3	0	1	1	0	0	0
3	0	3	3	3	0	3	3	0	3
3	0	3	3	3	0	3	3	0	0
0	3	0	0	0	3	0	3	0	3
1	0	3	1	3	0	3	3	1	0
1	3	1	1	0	3	0	0	0	0
0	3	0	2	0	2	0	0	0	0
2	0	1	1	0	0	2	3	0	1
0	3	1	0	0	3	0	0	0	0
3	0	3	0	3	0	3	3	3	3
0	3	0	0	0	3	0	0	0	0
3	0	1	3	3	3	3	3	3	3
0	2	0	0	0	2	0	0	0	0
1	2	0	2	0	2	0	0	0	0
0	3	0	0	0	3	0	2	0	0
0	3	0	0	0	3	0	0	0	0
1	2	1	3	1	2	3	1	1	3
0	3	3	0	0	3	0	0	0	0
0	2	0	0	0	0	0	1	0	0
0	3	0	0	0	0	0	0	0	0
0	3	3	2	0	1	3	0	0	3
0	2	0	0	0	1	0	1	0	0
0	3	0	0	0	3	0	0	3	0
0	0	0	0	0	0	0	0	0	0
0	2	0	0	0	2	0	2	0	0

0	0	0	0	0	0	0	0	0	0
0	1	0	0	0	0	0	3	0	0
3	0	3	2	0	2	2	3	0	2
1	1	0	0	0	1	1	0	0	0
1	0	1	1	0	0	0	3	0	0
2	1	1	1	0	1	0	0	0	1
0	0	0	0	0	0	0	0	0	0
0	3	1	0	0	0	0	3	0	0
0	0	0	0	0	0	0	0	0	0
0	0	0	0	0	0	0	0	0	0
0	3	0	0	0	0	0	3	0	0
0	0	0	0	0	0	0	0	0	0
0	3	0	0	0	3	0	0	0	0
0	0	0	0	0	0	0	0	0	0
0	0	0	0	0	0	0	0	0	0
0	0	0	0	0	0	0	0	0	0
0	0	0	0	0	0	0	0	0	0
0	1	0	0	0	0	0	1	0	1
3	0	3	3	1	2	3	1	1	1
0	0	0	0	0	0	0	0	0	0
0	3	0	0	0	3	0	1	0	0
0	0	0	0	0	0	0	0	0	0
0	0	0	1	1	1	1	0	0	0
1	0	0	1	0	1	0	1	0	3
0	0	0	0	0	0	0	0	0	0
0	1	0	0	0	3	0	0	0	0
0	2	1	1	1	0	2	0	1	0
2	1	0	0	1	1	0	0	1	2
0	3	0	0	0	0	0	0	0	0
0	1	2	2	0	1	1	0	0	0
0	0	0	0	0	0	0	0	0	0
0	3	0	0	0	0	0	0	0	0
0	3	0	0	0	0	0	0	0	0
0	3	0	0	0	0	0	0	0	0
0	3	0	0	0	0	0	0	0	0
0	3	0	0	0	0	0	0	0	0
3	1	2	2	0	1	2	2	0	2
0	3	0	0	0	3	0	0	0	0
1	2	1	1	1	1	1	0	1	1
0	3	0	0	0	0	0	0	0	0
1	0	1	2	0	1	1	2	1	1
0	2	0	1	0	2	0	1	0	0
1	3	1	0	0	2	0	0	0	0
0	0	0	0	0	0	0	0	0	0
0	0	0	0	0	3	1	1	1	0
0	0	0	0	0	0	0	0	0	0
0	3	1	1	0	0	0	1	0	0

0	0	0	0	0	0	0	0	0	0
0	0	0	0	0	0	0	0	0	0
0	0	0	0	0	0	0	0	0	0
1	0	1	0	1	1	0	1	1	0
0	3	0	0	0	0	0	0	0	0
0	0	0	0	0	0	0	0	0	0
0	0	1	1	1	1	1	1	1	1
1	1	1	1	1	1	1	1	1	1
1	1	1	1	1	1	1	0	0	1
0	0	0	0	0	1	0	1	0	1
1	1	1	0	0	0	0	0	0	0
1	1	1	1	1	0	1	0	0	0
0	3	0	0	0	0	1	0	0	0
0	0	0	0	0	0	0	0	0	0
3	0	2	2	0	0	3	2	0	2
3	1	0	0	0	0	0	1	0	0
3	0	1	1	0	0	1	1	0	3
3	0	1	1	0	0	1	1	0	3
1	1	1	1	1	1	1	1	1	0
3	0	0	1	0	2	1	3	0	1
3	1	1	3	1	0	3	3	3	3
3	0	0	1	0	0	0	1	3	3
1	3	3	0	0	3	0	1	0	0
0	3	0	1	3	1	2	1	0	0
0	3	0	0	0	3	0	0	0	0
1	3	0	0	0	0	0	3	1	3
1	1	1	1	1	1	1	1	1	1
0	3	0	0	0	0	0	0	0	0
0	1	0	0	0	1	0	0	0	0
0	3	0	0	0	3	0	0	0	0
1	0	0	1	1	0	0	0	0	1
1	2	1	1	0	1	0	0	0	2
3	0	0	1	1	1	1	1	1	1
1	1	0	0	0	0	0	1	0	0
1	0	0	1	0	0	0	1	0	0
1	1	1	1	1	1	1	1	1	1
0	1	0	0	0	1	0	1	0	0
0	1	0	0	0	1	0	0	0	0
3	0	1	1	3	0	1	3	1	3
0	1	0	0	0	0	0	0	0	0
0	1	0	0	0	0	0	0	0	0
0	1	0	0	0	0	0	0	0	0
1	0	0	1	1	1	1	0	0	1
0	1	0	0	0	0	0	0	0	0
0	1	0	1	0	1	0	0	0	0
0	1	0	0	0	0	0	0	0	0
1	1	1	1	1	1	1	1	1	1

0	0	1	1	0	0	1	3	0	0
1	3	0	0	0	3	0	0	0	0
1	1	3	0	0	0	0	0	0	1
0	0	3	3	3	0	1	3	1	3
1	3	1	0	0	3	0	0	0	1
0	0	0	0	0	0	1	0	0	0
1	0	0	0	0	0	0	0	0	0
3	3	0	0	1	3	0	0	0	0
1	0	1	3	0	2	0	3	1	0
0	3	0	0	0	3	0	1	0	0
0	2	0	1	0	0	0	0	0	0
0	3	0	0	0	3	0	1	0	0
0	3	0	0	0	3	0	1	0	0
1	2	0	1	0	3	0	1	0	1
3	1	2	3	2	1	3	2	1	2
3	1	2	3	2	1	3	2	1	2
3	0	3	3	3	0	3	0	3	3
3	0	3	3	3	0	1	3	1	3
3	1	0	1	2	0	1	0	2	2
3	0	1	2	0	0	1	1	2	1
3	3	0	0	0	0	0	3	0	0
1	1	1	1	0	3	0	0	0	0
0	2	0	0	0	3	0	0	0	0
1	1	0	1	1	1	1	1	1	1
0	2	0	1	0	2	0	0	0	0
0	3	0	0	0	3	0	0	0	0
0	3	0	0	0	1	0	0	0	0
0	3	0	0	2	3	0	0	0	0
0	3	0	0	0	0	1	0	0	0
0	2	0	0	0	2	0	0	0	3
0	3	0	2	0	3	0	0	0	0
0	2	0	0	0	2	0	0	0	0
1	0	1	1	1	0	1	1	1	1
0	3	0	0	0	0	0	0	0	0
0	0	0	0	0	0	0	0	0	0
0	0	0	0	0	0	0	0	0	0
0	0	0	0	0	0	0	0	0	0
0	0	0	0	0	0	0	0	0	0
0	0	0	0	0	0	0	0	0	0
3	2	0	0	0	0	0	3	0	0
1	0	0	1	1	0	1	1	1	1
0	3	0	0	0	0	0	0	0	0
0	0	0	0	0	0	0	0	0	0
0	0	2	0	0	0	0	3	0	3
0	3	0	0	0	0	0	0	0	0
0	3	0	0	0	0	0	0	0	0
0	3	0	0	0	3	0	0	0	0

1	0	1	0	1	3	1	0	1	1
0	3	0	0	0	3	0	0	0	0
3	0	3	1	3	0	3	3	3	3
0	3	0	0	0	0	0	0	0	0
1	0	0	0	0	0	0	0	0	0
0	2	0	0	0	1	0	1	0	0
3	0	0	0	0	1	0	0	1	1
0	3	0	0	0	1	0	0	0	0
0	1	0	0	0	2	0	0	0	0
0	3	0	0	0	0	0	0	0	0
1	0	3	1	3	0	1	3	1	2
3	1	0	2	0	1	0	1	0	0
0	0	0	0	0	0	0	0	0	0
2	1	3	2	0	2	3	2	0	2
1	3	0	1	3	3	3	1	1	1
0	3	0	0	0	3	0	0	0	0
0	0	0	0	0	0	0	0	0	0
0	0	0	0	0	0	0	0	0	0
0	0	0	0	0	0	0	0	0	0
0	3	0	0	0	1	0	0	0	0
1	1	1	0	0	1	0	0	0	0
1	1	1	1	0	1	1	1	0	0
0	0	0	0	0	0	0	0	0	0
0	3	0	0	0	0	0	0	0	0
0	3	0	0	0	0	0	0	0	0
0	3	0	0	0	3	0	0	0	0
0	3	0	0	0	3	0	0	0	0
2	0	2	2	1	0	0	0	0	0
0	0	3	0	0	0	0	3	0	3
0	0	0	0	0	0	0	0	0	0
0	3	0	0	0	0	0	0	0	0
0	0	1	3	0	0	0	3	0	0
0	0	0	0	0	0	0	0	0	0
0	3	0	0	0	3	0	0	0	0
0	3	0	0	0	3	0	0	0	0
3	0	3	3	3	0	3	3	0	3
0	0	0	0	0	0	0	0	0	0
0	0	0	0	0	0	0	0	0	0
3	1	3	2	0	2	3	2	0	3
0	1	0	1	0	0	0	0	0	0
0	0	0	0	0	0	0	0	0	0
1	0	0	0	0	0	0	0	0	1
0	0	0	0	0	0	0	0	0	0
0	0	0	0	0	0	0	0	0	0
3	0	2	0	0	1	3	3	2	3
0	0	0	0	0	0	0	0	0	0
0	0	0	0	0	3	0	0	0	0

1	2	1	2	0	3	3	3	0	1
0	0	0	0	0	0	0	0	0	0
0	0	0	0	0	0	0	0	1	0
0	0	0	0	0	0	0	0	0	0
0	0	0	0	0	0	0	0	0	0
0	2	0	0	0	2	0	0	0	0
1	1	1	1	0	1	0	0	3	1
0	3	0	0	0	0	0	0	0	0
0	3	0	0	0	0	0	0	0	0
0	3	0	0	0	3	0	3	0	0
0	3	0	0	0	0	0	0	0	0
1	3	0	1	0	2	0	1	0	0
0	2	0	0	0	0	0	0	0	0
0	1	2	0	2	0	0	1	2	0
0	2	0	0	0	1	0	1	0	0
0	0	0	0	0	0	0	0	0	0
0	3	0	0	0	0	0	0	0	0
0	3	0	0	0	0	0	0	0	0
0	3	0	0	0	0	0	0	0	0
3	2	3	3	0	1	3	0	0	3
0	3	0	0	0	3	0	1	0	1
0	3	2	0	0	3	0	0	0	0
0	3	0	0	0	0	0	0	0	0
0	1	0	1	0	1	1	0	0	1
0	3	1	1	1	3	0	0	0	0
0	0	0	0	0	0	0	0	1	1
2	1	1	3	1	2	0	2	0	0
0	3	0	0	0	0	0	0	0	0
1	2	0	0	0	0	0	0	0	0
0	0	0	0	0	0	1	1	0	0
0	3	0	0	0	0	0	3	0	0
0	1	0	1	0	0	1	0	0	0
1	1	0	0	3	1	3	1	1	1
0	0	0	0	0	0	0	0	0	0
0	0	0	0	0	0	0	0	0	0
0	0	0	0	0	0	0	0	0	0
0	0	0	0	0	0	0	0	0	0
1	0	0	0	0	0	0	0	0	0
0	1	1	0	0	0	0	0	0	0
0	0	0	0	0	0	0	0	0	0
0	1	2	1	0	2	2	1	1	1
0	1	0	0	0	2	0	0	0	0
0	0	0	1	1	1	0	0	1	0
0	0	0	0	0	0	0	0	0	0
1	1	1	1	1	1	1	1	1	1
0	0	0	0	0	0	0	1	0	1
0	0	0	0	0	0	0	0	0	0

1	0	0	0	1	1	1	0	0	0
1	0	1	0	1	0	0	0	0	0
0	1	1	1	1	1	1	1	1	1
0	1	1	0	0	0	1	1	0	0
0	0	0	0	0	0	0	0	0	0
1	1	1	1	1	1	1	1	1	1
1	0	1	1	0	1	0	1	0	1
0	1	0	1	0	0	0	0	0	1
0	2	0	0	0	2	0	0	0	0
0	1	1	1	1	0	1	1	0	1
0	0	0	0	0	0	0	0	0	0
0	0	1	1	1	1	1	1	1	1
1	0	1	1	3	1	0	0	0	1
1	1	1	1	1	1	1	1	1	1
0	0	0	0	0	0	0	0	0	0
2	0	2	2	0	0	0	0	0	0
3	0	3	3	0	0	1	0	1	2
1	0	0	0	0	0	0	0	0	0
3	0	0	3	0	0	1	2	0	3
2	1	2	2	0	0	2	0	0	0
2	0	0	0	0	2	1	1	0	0
0	2	0	0	0	0	0	2	0	0
0	1	1	1	0	0	1	0	1	1
0	1	1	1	0	0	1	0	1	1
1	3	0	0	1	3	0	1	1	0
1	0	1	3	1	2	3	0	2	1
0	3	0	0	0	1	1	1	1	1
2	0	2	0	0	0	0	0	1	0
0	2	0	3	0	2	0	0	0	0
0	3	0	0	0	3	0	0	0	0
2	0	1	1	0	1	0	0	0	0
0	3	0	0	0	1	0	0	0	1
1	0	1	1	1	1	1	1	1	1
3	3	1	1	1	1	1	1	1	1
1	2	2	0	0	1	1	0	0	0
1	2	0	1	1	0	3	2	1	1
1	2	0	1	1	0	3	2	1	1
3	0	1	1	0	1	2	0	0	0
3	0	1	1	0	1	2	0	0	0
1	3	0	1	0	3	0	0	0	0
0	3	0	0	0	1	0	0	0	0
3	0	3	1	0	0	0	1	0	2
3	0	3	3	2	0	3	0	0	2
1	0	1	3	0	0	0	0	0	0
1	0	1	3	0	0	0	0	0	0
1	0	1	1	1	1	0	1	2	1
1	0	1	1	1	1	0	1	2	1

0	1	0	0	0	3	0	0	0	0
0	3	0	1	0	3	0	0	0	1
0	1	0	0	3	0	2	2	0	0
0	1	2	0	0	0	0	0	0	1
0	0	0	0	0	0	0	0	0	0
0	3	0	0	0	0	0	3	0	0
0	1	0	0	0	1	0	0	0	0
1	1	1	1	1	1	1	1	1	1
0	1	0	0	1	0	0	0	0	0
0	1	0	0	0	0	0	0	0	0
0	3	0	0	0	3	0	0	0	0
0	3	0	0	0	0	0	0	0	0
0	1	0	0	0	0	0	0	0	0
0	0	0	0	0	0	0	0	0	0
1	2	1	1	1	1	1	1	1	1
0	1	0	0	0	0	0	0	0	0
0	1	0	0	0	0	0	0	0	0
0	1	0	0	0	0	0	0	0	0
1	1	0	1	0	1	1	1	1	1
0	1	0	0	0	2	0	0	0	0
0	1	0	0	0	0	0	0	0	0
0	1	0	0	0	1	0	1	1	1
1	1	1	1	1	1	0	0	0	0
1	3	0	1	1	1	1	1	0	1
0	3	0	0	0	3	0	0	0	0
0	1	1	0	0	3	1	0	0	0
0	0	0	0	0	0	0	0	0	0
1	3	0	0	0	0	0	0	0	0
1	1	1	1	1	1	1	1	0	0
0	1	0	0	0	0	0	0	0	0
1	1	0	0	0	0	1	0	1	1
0	3	0	0	0	0	0	1	0	0
0	1	0	1	0	0	0	0	0	0
0	1	0	0	0	0	0	0	0	0
1	1	1	1	1	1	1	1	1	1
3	3	0	2	1	1	2	0	1	3
0	3	0	0	0	3	0	0	0	0
0	3	0	0	0	3	0	0	0	0
0	3	0	0	0	3	0	1	0	0
0	3	0	0	0	1	0	0	0	0
1	2	0	1	0	2	0	0	1	1
0	0	0	0	0	0	1	0	1	0
0	3	0	0	0	3	0	0	0	0
0	0	0	0	0	0	0	0	0	0
2	0	0	0	0	1	0	3	0	0
3	0	0	3	0	3	2	3	0	3
0	3	0	0	0	3	0	0	0	0

0	3	0	0	0	3	0	0	0	0
2	3	0	1	1	3	0	0	0	0
0	3	0	1	1	2	0	0	0	0
0	1	0	0	0	1	0	1	0	0
2	0	2	3	0	0	1	2	0	3
2	0	2	3	0	0	1	2	0	3
0	0	0	0	0	0	0	0	0	0
0	3	0	0	0	3	0	0	0	0
2	3	2	3	2	2	0	3	0	3
3	1	3	3	3	3	1	1	1	3
2	2	0	1	0	2	0	0	0	1
0	3	0	1	0	3	0	1	0	0
0	3	0	0	0	0	0	0	0	0
0	3	0	0	0	3	0	2	0	0
0	3	0	0	0	3	0	0	0	0
0	3	1	0	0	0	0	2	0	0
2	3	2	1	1	0	0	3	1	1
0	3	0	0	0	3	0	0	0	0
0	3	0	0	0	3	0	0	0	0
3	3	1	0	0	3	0	0	0	0
1	3	2	1	1	1	1	1	1	1
0	3	0	0	0	3	0	0	0	0
0	1	0	0	0	0	0	0	0	0
0	0	0	0	0	0	0	0	0	0
1	0	1	1	1	1	1	0	1	1
0	0	0	0	0	0	0	0	0	1
0	3	1	0	0	0	0	3	0	0
3	3	3	0	0	3	0	0	0	0
0	3	0	0	0	3	0	0	0	0
0	3	0	0	0	3	0	0	0	0
1	1	1	1	1	1	1	1	1	1
0	3	0	0	0	3	0	3	0	0
0	3	0	0	0	3	0	0	0	0
0	3	0	0	0	3	0	0	0	0
0	2	0	0	0	2	0	0	0	0
0	2	1	0	2	0	0	1	2	0
0	3	0	0	0	3	0	0	0	0
0	3	1	0	0	3	0	0	1	1
0	3	0	0	0	3	0	1	0	0
0	3	0	0	0	3	0	0	0	0
0	3	0	0	0	3	0	3	0	0
1	2	1	2	2	1	0	0	0	3
0	2	2	0	0	3	0	0	0	2
0	3	0	0	0	3	0	0	0	0
1	3	0	1	0	1	1	0	0	0
2	2	0	1	0	2	0	0	0	1
0	3	0	0	0	3	0	0	0	0

0	3	0	0	0	0	0	0	0	0
0	2	0	0	0	1	1	0	0	0
1	1	0	1	0	1	1	0	0	0
0	3	0	1	0	2	0	1	0	0
3	2	0	1	0	2	1	1	0	0
1	2	0	1	0	2	0	0	0	0
2	3	0	0	0	2	0	0	0	0
1	1	1	1	1	1	0	3	1	1
1	1	1	1	1	1	0	1	0	0
1	0	1	0	1	1	1	1	1	1
0	0	1	0	1	0	0	0	0	0
0	0	0	0	0	0	0	0	0	0
0	0	0	0	0	0	0	0	0	2
0	0	0	0	0	0	0	0	0	0
0	0	0	0	0	0	0	0	0	0
0	0	0	0	0	0	0	0	0	0
3	3	3	3	1	3	3	3	3	3
0	0	0	0	0	0	0	0	0	0
3	1	0	0	0	1	1	1	0	0
3	3	3	3	3	0	3	3	2	1
0	0	0	0	0	0	0	0	0	0
3	3	3	3	3	0	3	3	3	1
0	3	0	0	0	0	0	0	0	0
0	1	1	1	0	0	0	0	0	0
0	0	0	0	0	0	0	0	0	0
0	0	0	0	0	0	0	0	0	0
0	0	1	0	1	0	0	0	0	0
0	0	0	1	1	1	0	1	0	1
0	0	0	0	0	0	0	0	0	0
0	0	0	0	0	0	0	0	0	0
0	0	1	1	1	1	1	1	1	1
0	3	0	0	0	0	0	0	0	0
1	3	0	3	1	1	1	1	1	0
0	0	0	0	0	0	0	0	0	0
0	0	0	0	0	0	0	0	0	0
0	3	0	0	0	3	0	0	0	0
1	3	3	1	1	1	1	3	3	3
1	1	1	0	0	1	1	1	1	1
0	0	0	0	0	0	0	0	0	0
0	0	0	0	0	0	0	0	0	0
0	0	0	0	0	0	0	0	0	0
2	3	0	0	0	0	0	0	0	0
0	3	0	0	0	0	0	0	0	0
3	0	2	3	0	0	0	3	0	2
1	0	2	0	0	0	1	3	0	2
3	0	3	0	0	0	0	3	0	0
0	3	0	0	0	3	0	0	0	0

0	3	0	3	0	3	0	3	0	1
3	0	3	1	1	0	3	1	3	3
2	1	2	1	1	2	0	2	0	1
1	2	1	1	0	2	0	3	0	1
3	0	3	3	0	3	1	0	1	0
0	3	0	0	0	3	0	0	0	0
2	0	3	3	0	0	3	3	0	3
0	3	0	0	0	3	0	0	0	0
0	2	0	0	0	2	0	1	0	0
0	1	0	0	0	0	0	0	0	0
0	3	0	0	0	3	0	0	0	0
3	0	3	3	3	0	1	3	3	3
0	3	0	0	0	3	0	0	0	0
3	3	3	3	3	0	1	3	0	0
0	3	0	0	0	3	0	0	3	1
1	2	1	1	0	2	0	0	0	1
2	2	1	2	0	1	2	3	1	3
0	3	0	0	0	3	0	0	0	0
3	0	3	3	3	0	3	3	3	3
0	3	0	0	0	3	0	0	0	0
0	3	0	0	0	3	0	0	0	0
1	1	1	1	0	1	0	1	0	0
3	0	3	3	3	0	1	1	1	1
0	3	3	0	0	3	0	3	0	0
3	1	3	2	0	0	1	3	0	1
0	0	0	0	0	0	0	1	0	0
0	3	0	0	0	0	2	0	0	0
2	2	2	3	1	2	1	3	0	1
1	1	1	1	0	0	0	0	0	1
0	1	1	1	1	1	1	1	1	1
0	2	0	0	0	1	0	0	0	0
1	3	1	1	1	1	1	1	1	1
0	2	1	1	0	0	0	3	0	0
0	0	0	0	0	0	0	0	0	0
0	0	0	0	0	0	0	0	0	0
0	3	0	0	0	0	0	1	0	0
0	3	0	0	0	3	0	1	0	0
1	3	1	0	1	0	0	1	0	1
0	0	0	0	0	0	0	0	0	0
1	3	0	0	0	3	1	1	1	0
0	1	0	0	0	0	0	0	0	0
2	1	0	2	0	3	3	2	0	2
1	1	1	1	1	1	1	1	2	1
0	3	0	0	0	0	0	0	0	0
0	3	0	0	0	0	0	0	0	0
0	1	0	0	0	3	0	0	0	0
1	0	1	3	0	0	1	3	0	1

0	3	0	0	0	3	0	0	0	0
0	3	0	0	0	0	0	0	0	0
1	3	0	0	0	0	0	0	0	0
0	3	0	0	0	3	0	3	0	0
0	3	0	0	1	3	1	0	0	0
0	3	0	0	0	0	0	1	0	0
0	2	0	0	0	0	0	0	0	0
0	0	0	0	0	0	0	0	0	0
0	0	0	0	0	0	0	0	0	0
0	0	0	0	0	0	0	0	0	0
0	3	0	0	0	3	0	0	0	0
0	0	0	0	0	0	0	0	0	0
2	1	0	0	0	0	0	3	0	1
0	1	2	0	0	0	0	2	0	1
0	2	1	0	0	0	0	0	0	0
1	3	0	0	0	1	1	0	1	1
0	0	0	3	0	1	3	3	0	0
0	3	0	0	0	0	0	0	0	0
1	1	0	2	0	1	2	1	0	0
0	0	0	0	0	0	0	0	0	0
1	2	1	3	1	1	0	1	0	0
1	3	1	0	0	3	0	0	0	0
2	1	0	0	0	0	0	1	0	0
1	0	1	1	1	1	1	1	1	1
3	0	0	1	0	0	0	1	0	2
0	3	0	0	0	0	0	0	0	0
0	0	0	0	0	0	0	0	0	0
3	0	3	3	0	0	3	3	0	0
0	3	0	0	0	3	0	1	0	0
0	3	0	0	0	0	0	0	0	0
0	0	0	0	0	0	0	0	0	0
0	0	0	0	0	0	0	0	0	0
0	1	0	0	0	3	0	1	0	0
0	3	3	0	0	3	0	0	0	0
3	0	1	3	0	0	0	1	0	0
0	3	1	0	0	3	0	0	0	0
1	1	1	1	1	1	1	1	1	1
1	2	0	1	1	0	0	3	0	1
0	3	0	0	0	0	1	0	1	0
0	0	0	0	0	0	0	0	0	0

suicide	endlife	sadhelp	counsel	famhelp	guidteac	helpdoc	agegroup	everrel_di	anynonpar
0	0	0	0	0	0	0	1	1	0
0	0	0	0	0	0	0	1	1	0
0	0	1	0	0	0	0	1	1	0
0	0	0	0	0	0	0	1	1	1
0	0	0	0	0	0	0	1	1	0
0	0	1	0	0	0	0	1	1	0
0	0	0	0	0	0	0	1	1	0
0	0	1	0	0	0	0	1	1	0
0	0	1	0	0	0	0	1	1	0
0	0	0		1	0	0	1	1	0
1	1	1	0	0	0	0	1	1	1
1	0	0	0	0	0	0	1	1	0
0	0	0	0	0	0	0	1	1	0
0	0	1	0	0	0	0	1	1	0
0	0	1	0	1	0	0	1	1	0
0	0	1	0	0	0	1	1	1	0
0	0	0	0	1	0	1	1	1	0
0	0	0	0	0	0	0	1	1	0
1	0	1	0	0	0	0	1	1	1
0	0	1	0	0	0	0	1	1	0
0	0	0	0	0	0	0	1	1	0
0	0	1	0	0	0	0	1	1	0
0	0	1	0	1	0	0	1	1	0
0	0	1	1	1	1	1	1	1	0
0	0	1	1	1	1	0	1	1	0
0	0	1	0	1	0	0	1	1	0
0	0	1	0	0	0	0	1	1	0
0	1	1	1	0	1	0	1	1	0
0	0	1	0	0	0	0	1	1	0
1	1	0	0	0	0	0	1	1	0
0	0	0	0	0	0	0	1	1	0
0	0						1	1	0
0	0	0	0	0	0	0	1	1	0
0	0	0	0	0	0	1	1	1	0
0	0						1	1	0
0	0	0	0	0	0	0	1	1	0
0	0	1	1	1	1	0	1	1	0
0	0	0	0	0	0	0	1	1	0
1	0	0	0	0	0	0	1	1	0
0	0	1	0	0	0	0	1	1	0
0	0	0	0	0	0	0	1	1	0
0	0	0	0	0	0	1	1	1	0
0	0	1	0	0	0	0	1	1	0
0	0	1	0	0	0	0	1	1	0
0	0	0	0	1	0	1	1	1	0
0	0	0	0	0	0	0	1	1	0

0	1	0	0	1	0	0	1	1	0
0	0	0	0	0	0	0	1	1	0
0	0	1	0	0	0	0	1	1	0
0	0	0	0	0	0	0	1	1	0
0	0	0	0	0	0	0	1	1	0
0	0	0	0	0	0	0	1	1	0
1	0	0	0	0	0	0	1	1	0
1	0	0	0	0	0	0	1	1	0
0	0	0	0	0	0	0	1	1	1
0	1	0	0	0	0	0	1	1	0
0	0	1	0	0	0	0	1	1	0
0	0	0	0	0	0	0	1	1	0
0	0	0	0	0	0	1	1	1	0
0	0	0	0	0	0	0	1	1	0
0	0	0	0	0	0	0	1	1	0
0	0	1	0	0	0	0	1	1	0
0	0	0	0	0	0	0	1	1	0
0	0	0	0	0	0	0	1	1	0
0	0	1	1	1	0	1	1	1	0
1	0	0	0	1	0	0	1	1	0
0	0	0	0	0	0	1	1	1	0
0	0	0	0	0	0	0	1	1	0
0	0	0	0	0	0	0	1	1	0
0	0	0	0	0	0	0	1	1	0
0	0	0	0	0	0	0	1	1	0
0	0	0	0	0	0	0	1	1	0
0	0	0	0	0	0	0	1	1	0
1	1	1	0	0	0	0	1	1	0
0	0	0	0	0	0	0	1	1	0
0	0	1	1	1	0	0	1	1	0
0	0	1	1	0	0	0	1	1	0
0	0	1	1	1	1	0	1	1	1
0	0	1	0	0	0	0	1	1	0
1	0	1	0	0	0	0	1	1	0
0	0	1	1	0	1	1	1	1	0
0	0	0	0	0	0	0	1	1	0
0	0	0	0	0	0	0	1	1	0
0	0	1	0	0	0	0	1	1	0
0	0	0	0	0	0	0	1	1	0
0	0	1	0	0	0	0	1	1	0
0	0	0	0	0	0	0	1	1	0
0	0	0	0	0	0	0	1	1	0
0	0	1	1	1	0	0	1	1	0
0	0	1	1	1	0	1	1	1	1
0	0	0	0	0	0	0	1	1	1
0	0	1	0	0	0	0	1	1	1
0	0	0	1	1	1	1	1	1	0

0	0	1	0	0	0	0	1	1	0
0	0	1	0	0	0	0	1	1	0
1	1	1	0	0	0	0	1	1	0
0	0	1	0	0	0	0	1	1	0
0	0	1	0	0	0	0	1	1	0
0	0	1	0	0	0	0	1	1	0
0	0	1	0	0	0	0	1	1	0
0	0	1	0	0	0	0	1	1	0
1	1	1	0	0	0	0	1	1	0
0	0	1	0	0	0	0	1	1	0
0	0	1	0	0	0	0	1	1	0
0	0	0	0	0	0	0	1	1	0
0	0	1	0	0	0	0	1	1	0
0	0	0	0	0	0	0	1	1	0
0	0	1	0	0	0	0	1	1	1
0	0	0	0	0	0	0	1	1	0
0	0	0	0	0	0	0	1	1	0
0	0	1	0	0	0	0	1	1	0
0	0	1	0	0	0	0	1	1	0
1	1	0	0	0	0	0	1	1	0
0	0	0	0	0	0	0	1	1	0
0	0	0	0	0	1	0	1	1	0
0	0	0	0	0	0	0	1	1	0
0	0	0	0	0	0	1	1	1	0
0	0	0	0	1	0	0	1	1	1
0	0	0	0	0	0	0	1	1	0
0	0	0	0	0	0	0	1	1	0
0	0	0	0	0	0	0	1	1	0
0	0	0	0	0	0	0	1	1	0
0	0	0	0	0	0	0	1	1	0
0	0	0	0	0	0	0	1	1	0
0	0	0	0	0	0	0	1	1	0
0	0	0	0	0	0	0	1	1	0
0	0	0	0	0	0	1	1	1	0
0	0	0	0	0	0	0	1	1	0
0	0	0	0	0	0	0	1	1	0
0	0	0	0	0	0	0	1	1	0
0	0	0	0	0	0	0	1	1	0
0	0	0	0	0	0	0	1	1	0
0	0	0	0	0	0	0	1	1	0
0	0	1	0	0	0	0	1	1	0
0	0	0	0	0	0	1	1	1	0
0	0	0	0	0	0	0	1	1	0
0	0	0	0	0	0	0	1	1	0
0	0	0	0	0	0	0	1	1	0
1	1	0	0	0	0	1	1	1	0
0	0	0	0	1	1	1	1	1	0

0	0	1	0	1	0	0	1	1	0
0	0	1	0	1	0	0	1	1	0
0	0	1	0	0	0	0	1	1	0
0	0	1	0	0	0	0	1	1	0
0	0	0	1	1	0	1	1	1	1
0	0	1	1	0	0	0	1	1	0
0	0	1	0	1	0	0	1	1	0
0	0	1	0	0	0	0	1	1	0
0	0	1	0	0	1	0	1	1	0
0	0	1	0	0	1	0	1	1	0
0	0	1	0	1	0	0	1	1	1
0	0	0	0	0	0	0	1	1	0
0	0	1	1	0	0	0	1	1	0
0	0	0	0	0	0	0	1	1	0
0	0	0	0	0	0	0	1	1	1
0	0	1	0	1	0	0	1	1	0
0	0	1	1	0	0	0	1	1	0
0	0	1	0	1	0	0	1	1	0
0	0	0	0	1	0	1	1	1	0
0	0	0	0	1	0	1	1	1	0
0	0	1	0	0	0	0	1	1	0
0	0	1	0	1	0	0	1	1	0
0	0	1	0	1	0	0	1	1	0
0	0	1	0	1	0	0	1	1	0
0	0	0	0	0	0	0	1	1	0
0	0	0	0	0	0	0	1	1	0
0	0	1	0	0	0	0	1	1	0
0	0	1	0	0	0	0	1	1	0
0	0	0	0	0	0	0	1	1	0
0	0	0	1	0	0	0	1	1	0
0	0	0	0	0	0	0	1	1	0
0	0	1	0	0	0	0	1	1	0
0	0	0	0	0	0	0	1	1	0
0	0	0	0	0	0	0	1	1	0
0	0	0	0	0	0	0	1	1	0
0	0	1	0	0	0	0	1	1	0
0	0	0	0	0	0	0	1	1	0
0	0	1	0	0	0	0	1	1	0
0	0	1	0	0	0	0	1	1	0
0	0	1	0	0	0	0	1	1	0
0	0	0	0	0	0	0	1	1	0
0	0	1	0	0	0	0	1	1	0
0	0	1	0	0	0	0	1	1	0
0	0	0	0	0	0	0	1	1	0
0	0	1	0	0	0	0	1	1	0
0	0	1	0	0	0	0	1	1	0
0	0	0	0	0	0	0	1	1	0

0	0	1	0	1	0	0	1	1	0
0	0	1	0	0	0	0	1	1	0
0	0	1	0	0	0	0	1	1	0
0	0	1	0	0	0	0	1	1	0
0	0	0	0	0	0	0	1	1	0
0	0	0	0	0	0	1	1	1	0
0	0	1	0	0	0	0	1	1	0
0	0	0	0	0	0	0	1	1	0
0	0	1	0	0	0	0	1	1	0
0	0	1	0	0	0	1	1	1	0
0	0	1	0	1	0	0	1	1	0
0	0	1	0	0	0	0	1	1	0
0	0	1	0	1	0	1	1	1	0
0	0	1	0	0	0	0	1	1	0
0	0	1	0	0	0	0	1	1	0
0	0	1	0	1	1	0	1	1	0
0	0	1	0	0	0	0	1	1	1
0	0	0	0	0	0	0	1	1	1
0	0	0	0	0	0	0	1	1	0
0	0	0	0	0	0	0	1	1	0
1	1	0	0	0	0	0	1	1	0
0	0	0	0	0	0	0	1	1	0
0	0	0	0	0	0	0	1	1	0
9	0	0	0	0	0	0	1	1	0
0	0	1	0	1	1	0	1	1	0
0	0	0	0	0	0	0	1	1	0
0	0	0	0	0	0	0	1	1	0
0	0	1	1	1	1	0	1	1	0
0	0	0	0	0	0	0	1	1	0
0	0	0	0	0	0	0	1	1	0
0	0	0	0	0	0	0	1	1	0
9	0	0	0	0	0	0	1	1	0
0	0	0	0	0	0	0	1	1	0
0	0	0	0	0	0	0	1	1	0
0	0	1	0	0	0	0	1	1	0
0	0	1	0	1	0	0	1	1	0
0	0	1	0	0	0	0	1	1	0
0	0	1	0	0	0	0	1	1	0
0	0	0	0	0	0	0	1	1	0
0	0	1	0	0	0	0	1	1	0
0	0	1	0	0	0	0	1	1	0
1	0	1	1	0	1	1	1	1	0
0	1	1	0	0	0	0	1	1	0
0	0	1	0	0	0	0	1	1	0
0	0	0	0	0	0	0	1	1	0
0	0	1	0	0	0	0	1	1	1
0	1	1	0	1		0	1	1	0

0	0	0	0	0	0	0	1	1	0
0	0	1	0	1	0	0	1	1	0
0	0	1	0	0	0	0	1	1	0
0	0	1	1	0	0	0	1	1	0
0	0	0	0	0	0	0	1	1	0
0	0	1	0	0	0	1	1	1	0
0	0	1	0	0	0	0	1	1	0
0	0	1	0	0	0	0	1	1	1
0	0	0	0	0	0	1	1	1	0
0	0	0	0	0	0	1	1	1	0
0	0	0	0	0	0	0	1	1	0
0	0	1	0	1	1	0	1	1	0
0	0	0	0	0	0	0	1	1	0
0	0	1	1	0	0	0	1	1	0
0	0	0	0	0	0	0	1	1	0
0	0	0	0	0	0	0	1	1	0
0	0	0	0	0	0	0	1	1	0
0	0	0	0	0	0	0	1	1	0
0	0	0	0	0	0	0	1	1	0
0	0	0	0	0	0	1	1	1	0
0	0	0	0	0	1	1	1	1	0
0	0	1	1	0	0	0	1	1	0
0	0	0	0	0	0	0	1	1	0
0	0	1	0	1	0	0	1	1	0
1	0	0	0	0	0	0	1	1	0
0	0	0	0	0	0	0	1	1	0
0	0	0	0	0	0	0	1	1	1
0	0	0	0	0	0	0	1	1	1
0	0	0	0	0	0	0	1	1	0
0	0	1	0	1	0	0	1	1	0
1	0	1	0	1	0	0	1	1	0
0	0	1	0	0	0	0	1	1	0
0	0	1	0	0	0	0	1	1	0
0	0	0	0	1	0	0	1	1	0
							1	1	0
0	0	0	0	0	0	0	1	1	0
0	0	1	0	0	0	0	1	1	0
1	0	0	0	0	0	0	1	1	0
0	0	1	0	0	0	0	1	1	0
0	0	1	0	1	0	0	1	1	1
0	0	1	0	0	0	0	1	1	0
0	0	1	0	0	0	0	1	1	0
0	0	1	0	0	0	0	1	1	0
0	0	1	0	0	0	0	1	1	0
0	0	0	0	0	0	1	1	1	0
0	0	1	0	0	0	0	1	1	0
0	0	0	0	0	0	1	1	1	1

0	0	0	0	0	0	0	1	1	0
0	0	1	1	0	1	0	1	1	0
0	0	0	0	1	0	0	1	1	0
0	0	1	0	0	0	0	1	1	0
1	0	0	0	1	0	0	1	1	0
0	0	1	1	1	0	0	1	1	0
1	0	1	0	0	0	0	1	1	0
0	0	0	0	0	0	1	1	1	0
0	0	0	0	0	0	0	1	1	0
1	1	1	0	1	0	0	1	1	0
9	0	1	0	0	0	0	1	1	0
0	0	1	0	0	0	0	1	1	0
0	0	0	0	0	1	0	1	1	0
0	0	1	1	1	1	0	1	1	0
0	0	1	1	1	1	0	1	1	0
0	0	0	0	0	0	0	1	1	0
0	0	1	0	0	0	0	1	1	0
1	1	1	1	0	1	1	1	1	1
0	0	1	0	0	0	0	1	1	0
0	0	0	0	0	0	0	1	1	0
0	0	0	0	0	0	0	1	1	0
0	0	1	1	0	1	1	1	1	0
0	0	1	0	0	0	0	1	1	0
0	0	1	0	0	0	0	1	1	0
0	0	1	0	0	0	0	1	1	0
0	0	0	0	0	0	0	1	1	1
0	0	1	0	0	0	0	1	1	0
0	0	0	0	0	0	0	1	1	0
0	0	1	0	1	0	0	1	1	0
0	0	1	0	0	0	0	1	1	0
0	0	1	0	0	0	0	1	1	0
0	0	1	0	1	0	0	1	1	0
0	0	0	0	0	0	0	1	1	0
0	0	1	0	1	0	0	1	1	0
1	0	1	0	0	0	0	1	1	1
0	0	1	1	0	0	1	1	1	0
0	0	1	0	0	0	0	1	1	0
0	0	1	0	0	0	0	1	1	0
0	0	0	0	0	0	0	1	1	0
0	0	0	0	0	0	0	1	1	0
0	0	0	0	0	0	0	1	1	0
0	0	0	0	0	0	0	1	1	0
0	0	0	0	0	0	1	1	1	0
0	0	1	0	1	0	0	1	1	0
0	0	1	0	1	0	0	1	1	0
0	0	0	0	0	0	1	1	1	0
0	0	1	0	0	0	0	1	1	0

0	0	1	0	0	0	0	1	1	0
0	0	0	0	0	0	0	1	1	0
0	0	0	0	1	0	0	1	1	1
0	0	1	0	0	0	0	1	1	0
0	0	1	0	1	0	0	1	1	0
0	0	1	0	1	0	0	1	1	0
0	0	0	1	1	0	0	1	1	0
0	0	0	1	1	0	0	1	1	0
0	0	1	0	1	1	0	1	1	0
0	0	1	1	0	0	0	1	1	0
0	0	1	0	0	0	0	1	1	0
0	0	0	0	0	0	0	1	1	0
0	0	1	0	0	0	0	1	1	0
0	0	0	1	1	0	0	1	1	0
0	0	1	1	1	0	0	1	1	0
0	0	0	1	0	1	1	1	1	0
0	0	0	0	0	0	1	1	1	0
0	0	0	0	0	0	0	1	1	0
1	0	1	0	0	0	0	1	1	0
1	0	1	0	1	0	0	1	1	0
0	0	0	0	0	0	0	1	1	0
0	0	0	0	0	0	0	1	1	0
0	0	1	0	0	0	0	1	1	0
0	0	0	0	0	0	0	1	1	0
0	0	1	0	0	0	1	1	1	0
0	0	1	1	0	0	0	1	1	0
0	0	1	0	0	0	0	1	1	0
0	0	0	0	0	0	0	1	1	0
0	0	0	0	0	0	0	1	1	0
0	0	1	0	0	0	0	1	1	0
0	0	1	0	0	0	1	1	1	0
0	0	1	0	0	0	0	1	1	0
0	0	0	0	0	0	0	1	1	0
0	0	1	1	1	0	0	1	1	0
0	0	1	1	0	0	0	1	1	1
0	0	1	0	0	1	0	1	1	0
0	0	0	0	0	0	0	1	1	0
0	0	0	0	0	0	0	1	1	0
0	0	0	0	1	1	1	1	1	0
1	9	1	1	1	1	1	1	1	0
0	0	1	1	0	0	0	1	1	0
0	0	0	0	0	0	0	1	1	0
0	0	1	1	1	1	1	1	1	0
1	1	1	0	0	0	0	1	1	1
0	0	1	0	0	0	0	1	1	0
0	0	1	0	0	0	0	1	1	0
0	0	0	0	0	0	0	1	1	0

0	0	0	0	0	0	0	1	1	1
0	0	1	1	1	1	1	1	1	0
0	0	1	1	1	1	1	1	1	0
0	0	0	0	0	0	0	1	1	0
0	0	1	0	0	0	0	1	1	0
0	0	0	0	0	0	0	1	1	0
0	0	0	0	0	0	0	1	1	0
0	0	0	0	0	0	0	1	1	0
0	0	0	0	0	0	0	1	1	0
0	0	0	0	0	0	0	1	1	1
0	0	0	0	0	0	0	1	1	1
0	0	1	1	1	1	1	1	1	0
0	0	1	1	1	1	0	1	1	0
0	0	0	0	0	0	0	1	1	0
0	0	0	0	0	0	0	1	1	0
0	0	0	1	0	0	0	1	1	0
0	0	1	1	0	0	0	1	1	0
0	0	1	0	0	0	1	1	1	0
0	0	0	0	0	0	0	1	1	0
0	0	1	1	1	1	0	1	1	0
0	0	0	1	0	0	0	1	1	0
1	1	0	0	0	0	0	1	1	0
0	0	1	0	0	0	0	1	1	0
0	0	1	0	0	0	0	1	1	0
0	0	1	0	0	0	0	1	1	0
0	0	1	1	1	1	1	1	1	0
1	1	0	0	0	0	0	1	1	0
1	1	0	0	0	0	0	1	1	0
0	0	1	0	1	0	0	1	1	0
0	0	1	1	0	0	0	1	1	0
0	0	1	1	1	0	1	1	1	0
0	0	1	1	0	0	1	1	1	0
0	0	0	0	0	0	0	1	1	0
0	0	1	0	0	0	0	1	1	0
0	0	0	0	0	0	0	1	1	0
0	0	1	0	0	0	1	1	1	0
0	0	0	0	0	0	0	1	1	0
0	0	0	0	1	0	1	1	1	0
0	0	1	0	0	0	0	1	1	0
0	0	0	0	0	0	0	1	1	1
1	1	0	0	0	0	0	1	1	0
0	0	0	0	0	0	0	1	1	0
1	1	0	0	0	0	1	1	1	0
0	0	1	1	1	0	1	1	1	0
0	0	0	0	0	0	1	1	1	0
0	0	1	0	0	0	0	1	1	0
0	0	0	1	1	0	1	1	1	0

0	0	1	0	0	0	0	1	1	0
0	0	0	0	0	0	0	1	1	0
0	0	1	0	0	0	0	1	1	0
0	0	1	1	1	0	0	1	1	0
0	0	1	0	1	0	0	1	1	0
0	0	0	0	0	0	0	1	1	0
0	0	0	0	0	0	0	1	1	0
0	1	1	0	0	0	0	1	1	0
0	0	0	0	0	0	0	1	1	0
0	1	0	1	0	0	1	1	1	0
0	0	0	0	0	0	0	1	1	0
1	1	1	0	1	0	0	1	1	0
0	0	1	0	1	0	0	1	1	0
0	0	0	0	0	0	0	1	1	0
0	0	0	0	1	0	0	1	1	0
0	0	0	0	0	0	0	1	1	0
0	0	1	0	1	1	0	1	1	0
0	0	1	0	1	0	0	1	1	0
0	0	1	0	1	0	1	1	1	0
0	0	1	0	1	0	0	1	1	0
0	1	0	0	0	0	0	1	1	0
0	0	1	0	1	0	1	1	1	0
0	0	1	0	1	0	0	1	1	0
0	0	1	0	0	0	0	1	1	0
0	0	1	0	1	0	0	1	1	0
0	0	0	0	0	0	0	1	1	0
0	0	1	1	1	0	0	1	1	0
0	0	1	0	1	0	0	1	1	0
0	0	1	0	1	1	1	1	1	0
0	0	1	0	1	0	1	1	1	0
0	0	1	0	0	0	0	1	1	0
0	0	0	0	0	0	0	1	1	0
0	0	0	0	0	0	0	1	1	0
0	0	1	0	0	0	0	1	1	0
0	0	1	0	0	0	0	1	1	0
1	1	1	0	0	0	0	1	1	0
0	0	1	1	1	1	1	1	1	0
0	0	1	0	0	0	0	1	1	0
0	0	1	0	0	0	0	1	1	0
0	0	1	0	1	0	0	1	1	0
0	0	1	0	0	0	0	1	1	0
0	0	0	1	0	0	0	1	1	1
0	0	0	0	0	0	0	1	1	1
0	0	1	1	1	0	0	1	1	0
0	0	1	0	0	1	0	1	1	1
0	0	1	0	1	0	0	1	1	0
0	0	1	0	1	0	0	1	1	0

0	0	1	0	0	0	0	1	1	0
0	0	0	0	0	0	0	1	1	0
0	0	1	0	0	0	0	1	1	0
0	0	1	0	1	0	0	1	1	0
0	0	0	0	0	0	0	1	1	0
0	0	1	0	1	0	0	1	1	0
0	0	1	0	0	0	0	1	1	0
0	0	1	0	0	0	0	1	1	0
0	0	1	0	0	0	0	1	1	0
0	0	1	0	1	0	1	1	1	0
0	0	1	0	0	0	0	1	1	0
0	0	1	0	1	0	0	1	1	0
0	0	1	0	1	0	0	1	1	1
0	0	0	0	0	0	0	1	1	0
0	0	1	0	1	0	0	1	1	0
0	0	0	0	0	0	0	1	1	0
0	0	1	0	0	0	0	1	1	0
0	0	0	0	0	0	0	1	1	0
0	0	1	1	1	0	0	1	1	0
0	0	1	0	0	0	0	1	1	0
0	0	1	0	1	0	0	1	1	0
0	0	1	0	0	0	0	1	1	0
0	0	1	0	0	0	0	1	1	0
0	0	0	0	0	0	0	1	1	0
0	0	0	0	0	0	0	1	1	0
0	0	0	1	0	1	0	1	1	0
0	0	0	0	0	0	0	1	1	0
0	0	1	0	0	0	0	1	1	0
0	0	1	0	0	0	0	1	1	0
0	0	1	0	0	0	0	1	1	0
0	0	1	0	0	0	0	1	1	0
0	0	0	0	0	0	0	1	1	0
0	0	0	0	0	0	0	1	1	1
0	0	0	0	0	0	0	1	1	0
0	0	1	0	1	0	0	1	1	0
0	0	1	0	0	0	0	1	1	0
0	0	0	0	0	0	0	1	1	0
0	0	0	0	0	0	0	1	1	0
0	0	1	0	1	0	0	1	1	0
0	0	1	0	1	0	0	1	1	0
0	0	0	0	0	0	0	1	1	0
0	0	0	0	0	0	0	1	1	0
0	0	1	0	0	0	0	1	1	0
0	0	1	0	1	0	0	1	1	0
0	0	1	0	0	0	0	1	1	0
0	0	1	0	0	0	0	1	1	0
0	0	0	0	0	0	0	1	1	0

0	0	0	0	0	0	0	1	1	0
0	0	0	1	0	0	0	1	1	0
0	0	0	0	0	0	0	1	1	0
0	0	1	1	1	0	0	1	1	0
0	0	0	0	0	0	0	1	1	0
0	0	0	0	0	0	0	1	1	0
0	0	1	1	1	0	0	1	1	0
0	0	1	1	1	1	0	1	1	0
0	0	0	0	0	0	0	1	1	0
0	0	1	0	0	0	0	1	1	0
0	0	0	0	0	0	0	1	1	0
0	0	0	0	0	0	0	1	1	0
0	0	1	1	1	0	0	1	1	0
0	0	0	0	0	0	0	1	1	1
0	0	0	0	0	0	0	1	1	0
0	0	1	0	0	0	0	1	1	0
0	0	1	1	1	0	0	1	1	0
0	0	0	0	0	0	0	1	1	0
0	0	1	0	1	0	0	1	1	0
9	0	0	0	0	0	0	1	1	0
0	0	0	0	0	0	0	1	1	0
0	0	1	0	0	0	0	1	1	0
0	0	0	0	0	0	0	1	1	0
0	0	1	0	0	0	0	1	1	0
0	0	1	1	0	0	0	1	1	0
0	0	1	0	1	0	0	1	1	0
0	0	1	0	1	0	0	1	1	0
0	0	1	0	0	0	0	1	1	0
0	1	1	1	0	1	1	1	1	0
0	0	1	0	1	0	1	1	1	0
0	0	0	1	0	0	0	1	1	0
0	0	0	0	0	0	0	1	1	0
0	0	1	0	0	0	0	1	1	0
1	1	0	0	0	1	1	1	1	1
0	1	1	0	1	0	0	1	1	0
0	0	0	0	0	0	0	1	1	0
0	0	1	0	0	0	0	1	1	1
0	0	0	0	0	0	0	1	1	0
0	0	0	1	0	0	0	1	1	0
0	0	1	0	1	0	0	1	1	0
0	0	1	0	0	0	0	1	1	0
0	0	1	0	1	0	0	1	1	0
0	0	0	1	0	0	0	1	1	0
0	0	1	0	0	0	0	1	1	0
0	0	0	1	1	0	0	1	1	0
0	0	1	0	1	0	1	1	1	0
0	0	0	0	0	0	0	1	1	0

0	0	1	0	1	0	0	1	1	0
0	0	0	0	0	0	0	1	1	0
0	0	1	0	1	0	0	1	1	0
0	0	0	0	0	0	0	1	1	0
0	0	1	0	1	0	0	1	1	0
0	0	1	1	1	1	1	1	1	0
0	0	1	0	0	0	0	1	1	0
0	0	1	0	0	0	1	1	1	0
0	0	1	0	0	0	0	1	1	0
0	0	1	0	1	0	0	1	1	0
0	0	1	0	0	0	1	1	1	0
0	0	0	0	0	0	0	1	1	0
0	0	0	0	0	0	0	1	1	0
0	0	1	1	1	1	0	1	1	0
0	0	1	0	0	0	1	1	1	0
0	0	1	0	0	0	0	1	1	0
0	0	0	0	0	0	0	1	1	0
0	0	0	0	0	0	0	1	1	0
0	0	0	0	0	0	0	1	1	0
0	0	0	0	1	0	1	1	1	0
1	0	0	0	0	0	0	1	1	0
0	0	1	0	1	1	1	1	1	0
0	0	1	0	0	0	0	1	1	0
0	0	1	0	1	0	1	1	1	0
0	0	0	0	0	0	1	1	1	0
0	0	1	1	1	0	0	1	1	0
0	1	1	0	0	0	0	1	1	0
0	1	1	0	0	0	0	1	1	0
0	0	0	0	0	0	0	1	1	0
0	0	1	0	1	1	1	1	1	0
0	0	0	0	0	0	0	1	1	0
0	0	0	0	0	0	0	1	1	0
0	0	0	0	0	0	1	1	1	0
0	1	0	0	0	0	0	1	1	0
0	0	1	0	1	0	1	1	1	0
0	0	0	0	0	0	1	1	1	0
1	0	0	0	0	0	0	1	1	0
0	0	0	0	0	0	1	1	1	0
0	0	1	0	0	0	0	1	1	0
0	0	1	1	1	0	1	1	1	0
0	0	1	0	0	0	0	1	1	0
0	0	1	0	0	0	0	1	1	0
0	0	0	0	0	0	0	1	1	0
0	0	0	0	0	0	0	1	1	0
0	0	1	0	0	0	0	1	1	0
0	0	1	0	0	0	0	1	1	0
0	0	0	0	0	0	0	1	1	0

0	0	1	1	1	0	1	1	1	0
0	0	1	0	0	0	0	1	1	0
0	0	1	0	0	0	0	1	1	0
0	0	0	0	0	0	0	1	1	0
0	0	0	0	0	0	0	1	1	0
0	0	1	0	0	0	1	1	1	0
0	0	0	0	1	0	0	1	1	0
0	1	1	0	0	0	0	1	1	0
0	0	1	0	0	0	0	1	1	0
0	0	1	0	1	0	0	1	1	0
0	0	1	0	0	0	0	1	1	0
1	0	0	0	0	0	0	1	1	0
0	0	1	0	0	0	0	1	1	0
0	0	1	0	1	0	0	1	1	0
0	0	0	0	0	0	0	1	1	0
0	0	1	0	0	0	0	1	1	0
0	0	0	0	0	0	0	1	1	0
0	0	0	0	1	0	0	1	1	0
1	1	0	0	0	0	0	1	1	1
0	0	1	0	0	0	0	1	1	0
0	0	1	0	0	0	0	1	1	0
0	0	1	0	0	0	0	1	1	0
0	0	1	0	1	0	0	1	1	0
0	0	1	0	0	0	0	1	1	0
0	0	1	0	0		0	1	1	0
0	0	1	0	1	0	0	1	1	1
0	0	0	0	0	0	1	1	1	0
0	0	1	0	1	0	1	1	1	0
1	1	1	1	1	0	0	1	1	0
0	0	0	0	1	0	0	1	1	0
0	0	0	0	0	0	0	1	1	1
0	0	1	1	0	0	1	1	1	0
0	0	0	0	0	0	0	1	1	0
0	0	0	0	0	0	1	1	1	0
0	0	1	0	1	0	0	1	1	0
0	0	1	0	0	0	0	1	1	0
0	0	0	0	0	0	0	1	1	0
0	0	0	0	0	0	0	1	1	0
0	0	0	1	0	0	0	1	1	0
9	0	0	1	0	0	0	1	1	0
0	0	1	1	0	1	0	1	1	1
0	0	0	0	0	0	1	1	1	0
0	0	1	0	1	0	1	1	1	0
0	0	1	0	0	0	0	1	1	0
0	0	1	1	0	0	0	1	1	0
0	0	0	0	0	0	0	1	1	0
0	0	0	0	0	0	0	1	1	0

0	0	0	0	1	0	1	1	1	0
0	0	1	1	1	1	0	1	1	0
0	0	1	1	1	0	1	1	1	0
0	0	0	0	0	0	0	1	1	0
0	0	1	1	1	0	1	1	1	0
0	0	0	0	0	0	0	1	1	0
0	0	1	0	0	0	0	1	1	0
0	0	1	0	0	0	0	1	1	0
0	0	1	0	0	0	0	1	1	0
0	0	1	0	0	0	0	1	1	0
9	0	1	1	1	1	0	1	1	0
0	0	1	0	1	0	0	1	1	0
0	0	0	0	0	0	0	1	1	0
0	0	1	0	1	0	0	1	1	0
0	0	1	1	0	0	1	1	1	0
0	0	0	0	0	0	0	1	1	0
0	0	1	0	0	0	0	1	1	0
0	0	0	0	0	0	0	1	1	0
0	0	0	0	0	0	0	1	1	0
0	0	1	0	0	1	0	1	1	0
0	0	0	0	0	0	0	1	1	0
0	0	0	0	0	0	0	1	1	0
0	0	1	0	0	0	0	1	1	0
0	0	0	0	0	0	0	1	1	0
0	0	0	0	0	0	0	1	1	0
0	0	1	0	0	0	0	1	1	0
0	0	1	1	1	0	0	1	1	0
0	0	1	0	1	0	0	1	1	1
0	0	1	0	0	0	0	1	1	0
1	0	1	0	1	1	1	1	1	0
0	0	0	0	0	0	0	1	1	0
0	0	0	0	0	0	0	1	1	0
0	0	0	0	0	0	0	1	1	0
0	0	0	0	0	0	0	1	1	0
1	0	1	1	1	0	0	1	1	0
1	0	1	1	1	0	0	1	1	0
0	0	1	0	1	0	0	1	1	0
0	0	1	0	1	0	0	1	1	0
0	0	0	0	0	0	0	1	1	0
0	0	0	1	0	0	0	1	1	0
0	0	0	0	0	0	0	1	1	0
0	0	0	0	0	0	0	1	1	0
0	0	1	0	1	0	0	1	1	1
0	0	1	1	1	0	0	1	1	0
0	0	1	0	1	0	0	1	1	0
0	0	1	1	1	0	0	1	1	0
0	0	0	0	0	0	0	1	1	0

0	0	0	1	0	0	1	1	1	0
0	0	1	0	0	0	0	1	1	0
0	0	1	0	0	0	1	1	1	0
0	0	0	0	0	0	0	1	1	0
0	0	0	0	0	0	0	1	1	0
0	0	0	0	0	0	1	1	1	0
0	0	0	0	0	0	1	1	1	0
0	0	1	0	0	0	0	1	1	0
0	0	1	0	1	0	1	1	1	0
0	0	0	0	0	0	0	1	1	0
0	0	0	0	0	0	0	1	1	1
0	0	0	0	0	0	0	1	1	1
0	0	0	0	0	0	0	1	1	0
0	0	1	1	0	1	0	1	1	0
1	0	0	0	0	0	0	1	1	0
0	0	1	0	0	0	1	1	1	0
0	0	1	0	0	1	0	1	1	1
0	0	1	0	0	0	0	1	1	0
0	0	1	1	1	1	1	1	1	0
0	0	1	0	1	0	0	1	1	0
0	0	0	0	0	0	0	1	1	0
0	0	1	0	1	0	0	1	1	0
0	0	0	0	0	0	0	1	1	0
0	0	1	0	0	0	0	1	1	0
0	0	1	0	0	0	0	1	1	0
0	0	1	0	1	0	1	1	1	0
0	0	0	0	0	0	0	1	1	0
0	0	1	0	1	1	1	1	1	0
1	0	1	0	1	0	0	1	1	0
0	0	0	0	0	0	0	1	1	0
0	0	1	0	0	0	0	1	1	0
0	0	0	0	0	0	0	1	1	0
0	0	0	0	0	0	0	1	1	0
0	0	1	0	0	0	0	1	1	0
0	0	0	0	0	0	1	1	1	0
0	0	1	0	1	0	1	1	1	0
0	0	1	0	1	0	1	1	1	0
0	0	1	0	1	0	1	1	1	0
1	0	0	0	0	1	0	1	1	0
0	0	1	0	1	0	0	1	1	0
0	0	1	0	1	0	0	1	1	0
0	0	0	0	0	0	0	1	1	0
1	1	1	0	0	0	0	1	1	0
0	0	0	0	0	0	0	1	1	0
0	0	1	1	0	1	0	1	1	0
0	0	0	0	0	0	0	1	1	0
0	0	1	0	0	0	0	1	1	0

0	0	0	0	0	0	0	1	1	0
0	0	1	0	0	0	0	1	1	0
0	0	1	0	0	0	0	1	1	0
1	1	1	0	0	0	0	1	1	0
0	0	0	0	0	0	0	1	1	0
0	0	0	0	0	0	0	1	1	0
0	0	1	1	1	0	0	1	1	0
0	0	1	1	1	0	0	1	1	0
0	0	0	1	1	0	0	1	1	0
0	0	0	1	1	0	0	1	1	0
0	0	1	0	0	0	0	1	1	0
0	0	0	0	0	0	0	1	1	0
1	1						1	1	0
0	0	0	0	0	0	0	1	1	1
1	0	1	0	0	0	0	1	1	1
0	0	0	0	0	0	0	1	1	0
0	1	0	0	0	0	0	1	1	0
0	0	1	0	0	0	0	1	1	0
0	0	1	1	0	0	0	1	1	0
0	0	0	0	0	0	0	1	1	0
0	0	0	0	0	0	0	1	1	0
0	0	0	0	0	0	0	1	1	0
0	0	0	0	0	0	0	1	1	0
0	0	1	1	1	0	0	1	1	0
0	0	1	0	0	0	0	1	1	0
0	0	1	1	1	0	0	1	1	0
0	0	1	1	0	0	0	1	1	0
0	0	1	0	0	0	0	1	1	0
0	0	1	0	0	0	0	1	1	1
0	0	0	1	0	0	0	1	1	0
0	0	1	0	0	0	0	1	1	0
0	0	1	1	1	1	1	1	1	0
0	0	1	1	1	0	0	1	1	0
0	0	0	0	0	0	0	1	1	0
0	0	1	0	0	0	0	1	1	0
0	0	1	0	0	0	0	1	1	0
0	0	1	1	0	0	0	1	1	0
0	0	1	0	0	0	0	1	1	0
0	0	0	0	0	0	0	1	1	0
0	0	1	0	0	0	0	1	1	0
0	0	1	1	1	1	1	1	1	0
0	0	0	0	0	0	0	1	1	0
0	0	1	0	0	0	0	1	1	0
0	0	1	1	1	0	1	1	1	0
0	0	1	0	0	0	0	1	1	0
0	0	1	0	0	0	0	1	1	0
0	0	1	0	0	0	0	1	1	0

0	0	1	0	0	0	0	1	1	0
0	0						1	1	0
1	0	1	0	0	0	0	1	1	0
0	0	0	0	0	0	0	1	1	0
0	0	1	0	0	0	0	1	1	0
0	0	1	0	0	0	0	1	1	0
0	0	1	1	0	0	0	1	1	0
0	0	1	1	1	0	0	1	1	0
0	0	1	1	1	0	0	1	1	0
1	1	1	0	0	0	0	1	1	0
0	0	0	0	0	0	0	1	1	0
0	0	0	0	0	0	1	1	1	0
0	0	1	0	0	0	0	1	1	0
0	0	1	1	1	0	0	1	1	0
0	0	1	0	0	0	0	1	1	0
0	0	1	0	0	0	0	1	1	0
0	0	1	0	0	0	0	1	1	0
0	0	1	0	1	0	0	1	1	1
0	0	1	1	0	0	1	1	1	0
0	0	1	0	1	0	0	1	1	0
0	0	1	0	0	0	0	1	1	0
0	0	1	1	0	0	0	1	1	0
0	0	1	0	1	0	0	1	1	0
0	0	1	0	0	0	0	1	1	0
0	0	1	0	0	0	0	1	1	0
0	0	0	0	0	0	0	1	1	0
0	0	1	0	0	0	0	1	1	0
0	0	1	0	0	0	0	1	1	0
0	0	0	0	0	0	0	1	1	0
0	0	0	0	0	0	0	1	1	0
0	1	0	0	0	0	0	1	1	0
0	0	0	0	0	0	0	1	1	0
0	0	0	0	0	0	0	1	1	0
0	0	1	0	0	0	0	1	1	0
0	0	1	1	0	0	0	1	1	0
0	0	1	0	0	0	0	1	1	0
0	0	0	0	0	0	0	1	1	0
0	0	0	0	0	0	1	1	1	1
0	0	1	0	0	0	0	1	1	0
0	0	1	0	0	0	0	1	1	0
0	0	0	0	0	0	0	1	1	0
0	0	1	0	0	0	0	1	1	0
0	0	1	1	0	0	1	1	1	0
0	0	0	0	0	0	0	1	1	0
0	0	1	0	0	0	0	1	1	0
0	0	1	0		0	0	1	1	0
0	0	0	0	0	0	1	1	1	0

0	0	1	0	0	0	0	1	1	0
0	0	0	0	0	0	0	1	1	0
1	1	0	1	0	0	0	1	1	0
0	0	0	0	0	0	0	1	1	0
0	0	1	0	0	0	1	1	1	0
0	0	0	0	0	0	1	1	1	0
0	0	0	0	0	0	1	1	1	0
0	0	0	1	0	0	1	1	1	0
0	0	1	0	0	0	0	1	1	0
0	0	0	0	0	0	0	1	1	0
0	1	0	1	0	0	0	1	1	0
0	0	0	0	0	0	0	1	1	0
1	1	0	0	0	0	0	1	1	1
0	0	0	0	0	0	0	1	1	0
1	1	1	1	1	0	1	1	1	0
0	0	0	0	0	0	0	1	1	0
0	0	0	0	0	0	0	1	1	0
0	0	0	0	0	0	0	1	1	0
0	0	0	0	0	0	0	1	1	0
0	0	0	0	0	0	0	1	1	0
0	0	0	0	0	0	0	1	1	0
0	0	0	0	0	0	0	1	1	0
0	0	1	0	0	0	1	1	1	0
0	0	0	0	0	0	0	1	1	0
0	0	0	0	0	0	0	1	1	0
0	0	0	0	0	0	0	1	1	0
0	0	0	0	0	0	0	1	1	0
0	0	1	0	0	0	0	1	1	0
0	0	0	0	0	0	0	1	1	0
1	1	0	0	0	0	1	1	1	0
1	1	0	0	0	0	0	1	1	0
0	0	0	0	0	0	0	1	1	0
0	0	1	1	0	1	1	1	1	0
1	0	1	0	0	0	0	1	1	0
0	0	0	0	0	0	0	1	1	0
0	0	0	0	0	0	0	1	1	0
0	0	1	0	1	0	0	1	1	0
0	0	0	0	0	0	0	1	1	0
0	0	1	1	0	1	1	1	1	0
0	0	0	0	0	0	0	1	1	0
0	0	0	0	0	0	0	1	1	0
0	0	0	0	0	0	0	1	1	0
0	0	0	0	0	0	0	1	1	0
0	0	0	0	1	0	0	1	1	0
0	0	1	0	0	0	0	1	1	0
0	0	0	0	0	0	0	1	1	0
0	0	1	0	0	0	0	1	1	0

1	0	1	0	0	0	0	1	1	0
1	0	1	0	0	0	0	1	1	0
0	0	0	0	0	1	1	1	1	0
0	0	0	0	0	0	1	1	1	0
0	0	0	0	1	1	1	1	1	0
0	0	0	0	0	0	0	1	1	0
0	0	0	0	0	0	0	1	1	0
0	1	0	0	0	0	0	1	1	0
1	0	0	0	0	0	0	1	1	0
0	0	1	0	0	0	0	1	1	0
0	0	0	0	0	0	0	1	1	0
0	0	0	0	0	0	0	1	1	0
1	1	0	0	0	0	0	1	1	1
0	0	0	0	0	0	0	1	1	0
0	0	0	0	0	0	0	1	1	0
0	0	1	0	0	0	0	1	1	0
0	0	1	0	1	0	0	1	1	0
0	0	1	1	0	0	1	1	1	0
0	0	0	0	1	0	1	1	1	0
0	0	1	1	1	0	1	1	1	0
0	0	1	0	0	0	0	1	1	0
0	0	1	0	1	0	0	1	1	0
0	0	0	0	0	0	0	1	1	0
0	0	1	0	0	0	0	1	1	0
0	0	1	1	0	0	0	1	1	0
0	0	1	0	0	0	0	1	1	0
0	0	1	1	1	0	1	1	1	0
0	0	1	0	0	0	1	1	1	0
0	0	1	1	1	0	0	1	1	0
0	0	0	0	0	0	0	1	1	0
0	0	1	0	0	0	0	1	1	0
0	0	0	0	0	0	0	1	1	1
0	0	1	0	0	0	0	1	1	0
0	0	0	0	0	0	0	1	1	0
0	0	0	1	0	0	0	1	1	0
0	0	0	0	0	0	0	1	1	0
0	0	1	0	1	0	0	1	1	0
0	0	1	1	1	1	1	1	1	0
0	0	1	1	1	0	1	1	1	0
0	0	1	0	0	0	0	1	1	0
0	0	1	0	0	0	0	1	1	0
0	0	1	0	1	0	0	1	1	0
0	0	1	0	1	0	0	1	1	0
0	0	1	1	1	0	1	1	1	0
0	0	1	0	0	0	1	1	1	0
0	0	0	0	0	0	0	1	1	0
0	0	0	1	0	0	0	1	1	0

0	0	0	1	0	0	0	1	1	0
0	0	0	0	0	0	0	1	1	0
1	1	1	0	0	0	0	1	1	0
0	0	0	0	0	0	1	1	1	1
0	0	0	0	0	0	1	1	1	0
0	0	0	0	0	0	1	1	1	0
0	0	0	0	0	0	0	1	1	0
0	0	0	0	0	0	0	1	1	0
0	0	0	1	0	0	0	1	1	0
0	0	1	1	1	0	0	1	1	0
0	0	0	0	0	0	1	1	1	0
0	0	1	0	1	0	0	1	1	0
0	0	0	0	1	0	1	1	1	0
1	1	1	0	0	1	0	1	1	0
0	0	0	0	0	0	0	1	1	0
0	0	1	0	0	0	0	1	1	0
0	0	0	0	0	0	0	1	1	0
0	0	0	0	0	0	0	1	1	0
1	1	0	0	1	0	0	1	1	0
0	0	1	1	1	0	0	1	1	0
1	1	0	0	0	0	0	1	1	0
0	0	1	1	1	0	0	1	1	0
0	0	1	0	0	0	0	1	1	0
0	0	1	0	1	0	0	1	1	0
0	0	1	1	1	1	1	1	1	0
0	0	1	0	0	0	1	1	1	0
0	0	1	0	0	1	0	1	1	0
0	0	1	0	0	0	0	1	1	0
0	0	1	0	0	0	0	1	1	0
0	0	1	0	1	0	0	1	1	0
0	0	1	0	0	0	0	1	1	0
0	0	1	1	0	0	0	1	1	0
0	0	0	0	0	0	0	1	1	0
0	0	1	0	0	0	0	1	1	0
0	0	1	0	1	0	0	1	1	0
0	0	1	0	1	0	0	1	1	0
0	0	1	0	0	0	0	1	1	0
0	0	1	0	1	0	0	1	1	0
0	0	0	0	0	1	0	1	1	0
0	0	1	0	1	0	0	1	1	0
0	0	1	0	1	0	0	1	1	0
0	0	1	0	0	0	0	1	1	0
0	0	0	0	0	0	0	1	1	0
0	0	1	0	0	0	0	1	1	1
0	0	1	0	1	0	0	1	1	0
0	0	1	0	0	0	0	1	1	0
0	0	1	0	1	0	0	1	1	0

0	0	0	0	0	0	0	1	1	0
0	0	1	0	1	0	0	1	1	0
0	0	1	0	1	0	0	1	1	0
0	0	1	1	0	0	1	1	1	0
0	0	1	0	0	0	0	1	1	0
0	0	0	0	0	0	0	1	1	0
0	0	0	0	0	0	0	1	1	0
0	0	1	0	0	0	0	1	1	0
0	0	0	0	0	0	0	1	1	0
0	0	1	0	0	0	0	1	1	0
0	0	0	0	0	0	0	1	1	0
0	0	1	0	1	0	0	1	1	0
0	0	1	0	0	0	0	1	1	0
0	0	1	0	0	0	0	1	1	0
0	0	0	0	0	0	0	1	1	0
0	0	1	0	1	0		1	1	0
0	0	1	0	1	0	0	1	1	0
0	0	1	0	0	0	0	1	1	0
1	0	1	0	0	0	0	1	1	1
0	0	1	0	1	0	0	1	1	0
0	0	1	0	1	0	0	1	1	0
0	0	1	0	0	0	0	1	1	0
0	0	1	0	0	0	0	1	1	0
0	0	1	0	0	0	0	1	1	0
0	0	1	1	0	0	0	1	1	0
0	1	0	0	0	0	0	1	1	0
0	0	0	0	0	0	0	1	1	0
0	0	0	0	0	0	0	1	1	0
0	0	1	0	0	0	0	1	1	0
0	0	1	0	1	0	0	1	1	1
0	0	1	0	1	0	0	1	1	0
0	0	1	0	1	0	0	1	1	0
0	0	1	0	0	0	0	1	1	0
0	0	1	0	1	0	0	1	1	0
0	0	0	0	1	0	0	1	1	0
0	0	1	0	1	0	0	1	1	0
0	0	1	0	1	0	0	1	1	0
0	0	1	0	1	0	0	1	1	0
0	0	1	0	0	0	0	1	1	0
1	1	1	0	0	0	0	1	1	0
0	0	0	0	0	0	0	1	1	0
0	0	1	0	0	0	0	1	1	0
0	0	1	0	1	0	0	1	1	0
0	0	0	0	0	0	0	1	1	0
0	0	0	0	0	0	0	1	1	0
0	0	1	0	0	0	0	1	1	0
0	0	1	0	0	0	0	1	1	0

0	0	1	0	0	0	0	1	1	0
0	0	1	0	0	0	0	1	1	0
0	0	1	0	0	0	0	1	1	0
0	0	1	0	1	0	0	1	1	0
0	0	0	0	0	0	0	1	1	0
0	0	1	0	0	0	0	1	1	0
0	0	0	0	0	0	0	1	1	1
0	0	1	0	0	0	0	1	1	0
0	0	1	0	1	0	0	1	1	0
0	0	1	0	0	0	0	1	1	0
0	0	1	0	1	0	0	1	1	0
0	0	0	0	0	0	0	1	1	0
0	0	0	0	0	0	0	1	1	0
0	0	1	0	0	0	0	1	1	0
0	0	0	0	0	0	0	1	1	0
0	0	1	0	0	0	0	1	1	0
0	0	0	0	0	0	0	1	1	0
0	0	0	0	0	0	0	1	1	0
0	0	0	0	0	0	0	1	1	0
0	0	0	0	0	0	0	1	1	0
0	0	0	0	0	0	0	1	1	0
0	0	0	0	0	0	0	1	1	1
0	0	1	1	1	1	0	1	1	0
0	0	0	0	0	0	0	1	1	0
0	0	0	0	0	0	0	1	1	0
0	0	0	0	0	0	0	1	1	0
9	1	1	0	0	0	0	1	1	0
0	0	1	0	0	0	0	1	1	0
0	0	1	0	0	0	0	1	1	0
0	0	0	0	0	0	0	1	1	0
0	0	0	0	0	0	0	1	1	0
0	0	0	0	0	0	0	1	1	0
9	0	1	0	0	0	0	1	1	0
0	0	1	0	0	0	0	1	1	0
0	0	0	0	0	0	0	1	1	0
0	0	1	0	0	0	0	1	1	0
9	0	1	0	0	0	0	1	1	0
0	0	0	0	0	0	0	1	1	0
0	0	1	0	1	0	0	1	1	0
0	0	1	0	0	1	1	1	1	1
0	0	0	0	0	0	0	1	1	0
	0	1	0	0	0	0	1	1	0
0	1	1	0	1	0	0	1	1	0
0	0	1	0	0	0	0	1	1	0
0	0	1	1	0	0	1	1	1	0
0	0	1	0	1	0	0	1	1	0
0	0	0	0	0	0	0	1	1	0

0	0	0	0	0	0	0	1	1	1
0	0	0	0	0	0	0	1	1	0
0	1	0	0	0	0	0	1	1	0
0	0	1	0	0	0	0	1	1	0
1	1	0	0	0	0	0	1	1	0
0	0	0	0	0	0	0	1	1	1
0	0	0	0	0	0	0	1	1	0
9	0	0	0	0	0	0	1	1	0
0	0	1	0	0	0	0	1	1	0
0	0	1	0	0	0	0	1	1	0
0	0	1	0	0	0	0	1	1	1
0	0	1	0	1	0	0	1	1	0
0	0	1	0	0	0	1	1	1	0
0	0	0	0	0	0	0	1	1	1
0	0	0	0	0	0	0	1	1	0
0	0	0	0	0	0	1	1	1	0
0	0	1	0	1	0	0	1	1	0
0	0	0	0	0	0	0	1	1	0
0	0	0	0	0	0	0	1	1	0
0	0	1	1	1	1	0	1	1	0
1	0	1	0	0	0	0	1	1	1
0	0	1	1	1	1	1	1	1	0
0	0	1	1	0	1	0	1	1	0
0	0	1	0	0	0	0	1	1	0
0	0	0	0	0	0	1	1	1	0
0	0	1	0	1	0	0	1	1	0
0	0	1	0	0	0	0	1	1	0
0	0	1	0	0	0	0	1	1	0
0	0	1	0	0	0	0	1	1	0
0	0	1	0	0	0	0	1	1	0
0	0	1	0	0	0	0	1	1	0
0	0	1	1	1	0	1	1	1	1
0	0	1	0	0	0	0	1	1	0
0	0	0	0	0	0	0	1	1	0
0	0	0	0	0	0	0	1	1	0
0	0	0	0	0	0	0	1	1	0
0	0	1	0	0	0	0	1	1	0
0	0	0	0	0	0	0	1	1	0
0	0	1	1	0	0	0	1	1	0
0	0	0	0	1	0	1	1	1	0
0	0	0	0	0	0	1	1	1	0
0	0	0	0	0	0	1	1	1	0
0	0	0	0	0	0	0	1	1	0
0	0	0	0	0	0	0	1	1	0
0	0	1	0	1	0	0	1	1	0
0	0	1	1	0	0	1	1	1	0
0	0	1	0	1	1	1	1	1	0

0	0	0	0	0	0	1	1	1	0
0	0	1	0	0	0	0	1	1	0
0	0	1	1	1	0	1	1	1	0
0	0	1	0	1	0	1	1	1	0
0	0	1	0	0	0	0	1	1	0
0	0	1	0	1	0	0	1	1	0
0	0	1	0	1	0	1	1	1	0
0	0	1	0	0	0	1	1	1	0
0	0	1	0	0	0	0	1	1	0
0	0	0	0	0	0	0	1	1	1
0	0	1	0	1	0	1	1	1	0
0	0	1	1	1	0	1	1	1	0
0	0	0	0	0	0	0	1	1	0
1	0	0	0	0	0	0	1	1	0
0	0	1	0	1		0	1	1	0
0	0	1	1	1	0	0	1	1	0
0	0	1	0	1	0	0	1	1	0
0	0	1	1	1	0	0	1	1	0
0	0	1	0	0	0	0	1	1	0
0	0	1	0	1	0	0	1	1	0
1	1	1	0	0	0	0	1	1	0
1	0	1	0	1	0	0	1	1	0
0	0	0	0	0	0	0	1	1	0
0	0	0	0	0	0	0	1	1	0
0	0	1	0	1	0	0	1	1	1
0	0	1	1	0	0	1	1	1	0
0	0	1	0	0	0	0	1	1	0
0	1	0	0	0	0	0	1	1	0
0	1	1	0	0	0	0	1	1	0
0	0	1	0	0	0	1	1	1	0
0	0	1	1	0	0	0	1	1	1
0	0	0	1	1	1	1	1	1	0
0	0	0	0	0	0	0	1	1	0
0	0	0	0	1	0	1	1	1	0
0	0	1	0	0	0	0	1	1	0
0	0	0	0	0	0	0	1	1	0
0	0	0	0	0	0	0	1	1	0
1	1	0	0	0	0	0	1	1	0
0	0	1	1	0	0	0	1	1	0
0	0	1	0	0	0	0	1	1	0
0	0	0	0	0	0	0	1	1	0
0	0	1	1	1	0	1	1	1	0
1	0	0	1	1	0	0	1	1	1
0	0	1	0	1	0	1	1	1	0
0	1	0	0	0	0	0	1	1	0
0	0	0	0	0	0	0	1	1	0
0	0	1	0	0	1	0	1	1	0

0	0	1	0	0	0	0	2	1	0
0	0	1	0	0	0	0	2	1	0
0	0	1	0	0	0	0	2	1	0
0	0	1	0	0	0	0	2	1	0
0	0	0	0	0	0	0	2	1	0
0	0	0	0	0	0	0	2	1	0
0	0	1	1	1	0	1	2	1	0
0	0	0	0	0	0	0	2	1	0
0	0	1	0	0	0	0	2	1	1
0	0	1	0	1	0	0	2	1	0
0	0	1	0	1	0	0	2	1	0
0	0	0	0	0	0	0	2	1	0
0	0	1	0	1	0	0	2	1	1
0	0	1	0	0		0	2	1	0
0	0	0	0	0	0	0	2	1	0
0	0	1	0	0	0	0	2	1	1
0	0	0	0	1	0	1	2	1	0
0	0	1	0	1	0	1	2	1	0
0	0	1	0	0	0	0	2	1	0
0	0	0	0	0	0	0	2	1	0
0	0	1	0	0	0	0	2	1	0
0	0	1	1	1	0	0	2	1	1
0	0	0	0	0	0	0	2	1	0
0	0	0	0	0	0	0	2	1	0
0	0	0	0	0	0	0	2	1	0
0	0	1	0	0	0	0	2	1	0
1	0	1	1	1	1	1	2	1	0
0	0	1	0	1	0	0	2	1	0
0	0	0	0	0	0	0	2	1	0
0	0	0	0	0	0	0	2	1	0
0	0	0	1	0	0	0	2	1	0
0	0	0	0	0	0	0	2	1	0
0	0	0	0	0	0	0	2	1	0
0	0	0	0	0	0	0	2	1	0
0	0	1	0	1	0	0	2	1	0
0	0	1	0	0	0	0	2	1	0
0	0	0	0	0	0	1	2	1	0
0	0	0	0	0	0	1	2	1	0
0	0	0	0	0	0	0	2	1	0
0	0	0	0	0	0	0	2	1	0
0	0	1	0	0	0	0	2	1	0
0	0	0	0	0	0	0	2	1	0
0	0	0	0	0	0	1	2	1	0
0	0	0	0	0	0	0	2	1	0
0	0	1	1	0	0	0	2	1	1
0	0	1	1	0	0	0	2	1	1
0	0	1	0	1	0	1	2	1	0

0	0	1	0	1	0	1	2	1	0
0	0	0	0	0	0	0	2	1	0
0	0	0	0	0	1	0	2	1	0
0	0	1	0	0	0	0	2	1	0
0	0	1	1	0	1	1	2	1	0
0	0	1	0	0	0	0	2	1	0
0	0	1	0	0	0	1	2	1	0
0	0	1	0	1	0	0	2	1	0
0	0	1	0	0	0	0	2	1	0
0	0	0	0	1	0	0	2	1	0
0	0	1	0	0	0	0	2	1	0
0	0	1	0	0	0	0	2	1	0
0	0	0	0	0	0	0	2	1	0
0	0	1	0	1	0	0	2	1	0
0	0	1	0	0	0	0	2	1	1
0	0	1	0	1	0	0	2	1	1
0	0	1	1	1	1	1	2	1	0
0	0	0	0	0	0	0	2	1	0
0	0	1	0	0	0	0	2	1	0
0	0	1	0	0	0	0	2	1	0
0	0	0	0	0	0	0	2	1	0
0	0	1	0	0	0	0	2	1	0
1	0	0	0	0	0	0	2	1	0
0	0	1	0	0	0	0	2	1	0
1	0	1	0	0	0	0	2	1	0
1	0	1	0	0	0	0	2	1	0
0	0	0	0	0	0	0	2	1	0
0	0	1	0	0	0	0	2	1	0
0	0	1	0	0	0	0	2	1	0
0	0	1	0	1	0	0	2	1	1
0	0	0	0	0	0	0	2	1	0
0	0	0	0	0	0	0	2	1	1
0	1	1	0	0	0	0	2	1	0
0	0	0	0	0	0	1	2	1	0
0	0	0	1	0	0	0	2	1	0
0	0	0	0	0	0	0	2	1	0
0	0	1	0	1	0	0	2	1	1
0	0	1	0	1	0	0	2	1	1
0	0	1	1	0	0	0	2	1	0
0	0	1	1	1	0	0	2	1	0
0	0	0	1	0	0	0	2	1	0
0	0	1	0	1	1	0	2	1	1
0	0	1	0	1	0	0	2	1	0
0	0	0	0	1	1	0	2	1	0
0	0	0	0	1	1	0	2	1	0
0	0	1	1	0	0	1	2	1	0
0	0	1	0	0	0	1	2	1	0

0	0	0	0	0	0	0	2	1	0
0	0	0	0	0	0	0	2	1	1
0	0	0	0				2	1	1
0	0	1	0	0	0	1	2	1	0
0	0	0	0	0	0	0	2	1	0
0	0	0	0	0	0	0	2	1	0
0	0	1	0	1	0	1	2	1	1
0	0	1	0	1	0	1	2	1	1
0	0	1	0	1	0	1	2	1	1
0	0	1	0	0	0	0	2	1	0
0	0	1	0	1	0	0	2	1	0
0	0	0	0	0	0	0	2	1	0
0	0	0	0	1	0	0	2	1	0
0	0	1	0	0	0	0	2	1	0
0	0	0	0	0	0	0	2	1	0
0	0	1	0	1	0	0	2	1	0
1	1	1	0	0	0	0	2	1	0
0	0	1	0	0	0	0	2	1	0
0	0	1	0	0	0	0	2	1	0
0	0	0	1	1	1	0	2	1	0
0	0	0	1	0	0	1	2	1	0
0	0	1	0	0	0	0	2	1	0
0	0	1	0	0	0	0	2	1	0
0	0	1	0	0	0	0	2	1	0
0	0	1	1	1	1	1	2	1	0
0	0	0	0	0	0	0	2	1	0
0	0	0	1	0	1	1	2	1	0
0	0	1	0	0	0	0	2	1	0
0	0	1	0	0	0	0	2	1	0
0	0	0	0	0	0	0	2	1	0
0	0	0	0	0	0	0	2	1	0
0	0	0	0	0	0	0	2	1	0
0	0	1	0	0	0	0	2	1	0
0	0	1	1	0	0	0	2	1	0
0	0	0	0	0	0	0	2	1	0
0	0	1	1	0	0	0	2	1	0
0	0	1	1	1	0	0	2	1	0
0	0	1	0	0	0	0	2	1	0
0	0	1	0	0	0	0	2	1	0
0	0	0	0	0	0	0	2	1	0
0	0	1	1	1	1	1	2	1	0
0	0	0	0	0	0	0	2	1	0
1	0	1	0	0	0	0	2	1	0
0	0	1	1	1	1	1	2	1	0
1	0	0	0	0	0	0	2	1	0
0	0	1	1	1	1	1	2	1	0
0	0	0	0	0	0	0	2	1	0

0	0	1	0	1	0	1	2	1	0
0	0	0	0	0	0	0	2	1	0
0	0	0	0	0	0	0	2	1	0
0	1	0	0	0	0	0	2	1	0
0	0	0	0	0	0	0	2	1	0
0	0	1	1	1	0	0	2	1	0
0	0	1	1	1	1	1	2	1	0
0	0	1	1	1	1	1	2	1	0
0	0	0	1	1	0	0	2	1	0
0	0	1	1	1	0	0	2	1	0
0	0	1	1	0	0	0	2	1	0
0	0	1	1	1	0	0	2	1	0
0	0	1	1	0	0	0	2	1	0
0	0	0	0	0	0	0	2	1	0
0	0	1	1	0	0	0	2	1	0
0	0	0	0	0	0	0	2	1	0
0	0	1	1	1	1	1	2	1	0
0	0	1	1	1	1	1	2	1	0
0	0	0	0	0	0	0	2	1	0
0	0	0	0	0	0	0	2	1	0
1	0	0	1	1	0	0	2	1	0
0	0	0	0	0	0	0	2	1	0
0	0	1	1	1	0	1	2	1	0
0	0	1	1	1	0	0	2	1	0
0	0	0	0	0	0	0	2	1	0
0	0	0	0	0	0	0	2	1	0
0	0	1	1	1	1	1	2	1	0
0	0	1	1	1	1	0	2	1	0
0	0	1	1	1	1	0	2	1	0
0	0	1	0	0	0	0	2	1	0
0	0	0	0	0	0	0	2	1	0
1	0	1	0	0	0	0	2	1	0
0	0	1	1	1	1	1	2	1	0
0	0	0	0	0	0	0	2	1	0
0	0	1	1	1	0	0	2	1	0
1	1	0	0	0	0	0	2	1	0
0	0	1	1	1	0	0	2	1	0
0	0	1	1	1	1	1	2	1	0
0	0	1	1	1	1	1	2	1	0
0	0	1	1	0	0	0	2	1	0
1	1	1	1	1	1	1	2	1	0
1	1	1	1	1	1	1	2	1	0
0	0	1	1	1	0	1	2	1	1
0	0	1	1	0	0	1	2	1	0
0	0	1	0	0	0	0	2	1	0
0	0	0	0	0	0	0	2	1	0
0	0	1	1	1	0	1	2	1	0

0	0	1	0	0	0	0	2	1	0
1	1	1	1	1	0	0	2	1	0
0	0	1	0	0	0	0	2	1	0
0	0	1	0	0	0	0	2	1	0
0	0	1	0	0	0	0	2	1	0
0	0	1	0	0	0	0	2	1	0
0	0	1	0	0	0	0	2	1	0
0	0	1	1	0	0	1	2	1	0
1	1	1	0	0	0	0	2	1	0
0	0	0	0	0	0	0	2	1	0
0	0	0	0	0	0	0	2	1	0
0	0	0	0	0	0	0	2	1	0
1	1	0	1	1	1	0	2	1	0
1	1	0	1	1	1	0	2	1	0
0	0	1	1	0	0	0	2	1	0
0	0	1	0	0	0	0	2	1	0
1	1	1	1	1	0	0	2	1	0
0	0	1	1	1	1	0	2	1	0
0	0	1	0	0	0	0	2	1	0
0	0	1	0	0	0	0	2	1	0
0	1	1	1	1	0	0	2	1	0
0	0	0	0	0	0	0	2	1	0
0	0	0	0	0	0	0	2	1	0
0	0	1	0	0	0	0	2	1	0
0	0	1	0	0	0	1	2	1	0
0	0	1	0	0	0	0	2	1	0
0	0	1	1	1	0	0	2	1	0
0	0	1	1	0	0	0	2	1	0
1	0	0	0	0	0	0	2	1	0
1	0	0	0	0	0	0	2	1	0
0	0	1	1	1	0	0	2	1	0
0	0	1	1	1	1	0	2	1	0
0	0	1	0	0	0	1	2	1	0
0	0	1	0	1	0	0	2	1	0
0	0	1	0	0	1	0	2	1	0
1	1	1	0	1	0	0	2	1	1
0	0	1	1	1	1	1	2	1	0
0	0	0	0	0	0	0	2	1	0
1	1	1	0	0	0	0	2	1	0
0	0	1	0	0	0	0	2	1	0
0	0	1	0	0	0	0	2	1	0
0	0	1	1	0	0	0	2	1	0
0	0	0	0	0	0	0	2	1	0
0	0	1	0	0	0	0	2	1	1
0	0	1	0	0	0	0	2	1	0
0	0	1	0	0	0	0	2	1	0
0	0	1	0	1	0	1	2	1	0

0	0	1	0	0	0	0	2	1	0
0	0	0	0	0	0	0	2	1	0
0	0	0	1	1	1	0	2	1	0
0	0	0	1	1	0	1	2	1	1
0	0	0	0	0	0	1	2	1	0
0	0	1	0	1	0	1	2	1	0
0	0	0	0	0	0	0	2	1	0
0	0	0	0	0	0	0	2	1	0
0	0	1	0	0	0	0	2	1	0
0	0	1	0	1	0	0	2	1	0
0	0	1	0	1	0	0	2	1	0
0	0	0	0	0	0	1	2	1	0
0	0	0	0	0	0	1	2	1	0
1	1	1	1	0	1	1	2	1	1
0	0	0	0	0	0	0	2	1	0
0	0	0	0	0	0	0	2	1	0
0	0	1	1	1	0	1	2	1	0
0	0	0	0	0	0	0	2	1	0
0	0	0	0	0	0	0	2	1	0
0	0	0	0	0	0	0	2	1	0
0	0	0	0	0	0	0	2	1	0
0	0	0	0	0	0	0	2	1	0
0	0	1	0	0	0	0	2	1	0
1	1	0	0	0	0	0	2	1	0
0	0	1	0	1	0	0	2	1	0
0	0	1	0	1	0	0	2	1	0
0	0	1	0	1	0	1	2	1	0
0	0	1	0	0	0	0	2	1	0
1	0	0	0	0	0	0	2	1	0
0	0	0	0	1	1	0	2	1	0
0	0	0	0	0	0	0	2	1	0
0	0	1	1	1	1	1	2	1	0
0	0	0	0	0	0	1	2	1	0
0	0	1	0	0	0	0	2	1	0
0	0	1	0	1	0	1	2	1	0
0	0	1	0	1	0	0	2	1	0
0	0	0	1	1	0	0	2	1	0
0	0	1	0	1	0	0	2	1	0
0	0	1	0	0	0	0	2	1	0
0	0	1	0	0	0	0	2	1	0
0	0	1	0	1	0	1	2	1	0
0	0	1	0	1	1	0	2	1	0
0	0	1	1	1	1	0	2	1	0
0	0	1	0	1	0	1	2	1	0
0	0	0	0	0	0	0	2	1	0
0	0	0	0	0	0	0	2	1	0
0	0	1	0	1	0	0	2	1	0

0	0	1	1	0	0	1	2	1	0
0	0	1	0	0	0	0	2	1	1
0	0	0	0	0	0	0	2	1	0
0	0	0	0	0	0	0	2	1	0
1	1	0	0	0	0	0	2	1	0
0	0	0	0	0	0	0	2	1	1
0	0	1	0	1	0	0	2	1	0
1	0	1	1	0	0	0	2	1	0
0	0	0	0	0	0	0	2	1	0
0	0	1	0	1	0	0	2	1	1
0	0	0	0	0	0	0	2	1	0
0	0	1	0	0	0	0	2	1	1
0	0	0	0	1	1	1	2	1	0
1	1	1	0	1	0	0	2	1	0
0	0	1	0	0	0	1	2	1	0
0	0	1	0	1	0	0	2	1	0
0	0	1	1	1	0	0	2	1	0
0	0	0	0	0	0	0	2	1	0
0	0	1	0	1	0	0	2	1	0
0	0	1	0	1	0	0	2	1	0
0	0	1	0	1	0	0	2	1	0
0	0	1	1	1	1	1	2	1	0
0	0	1	1	1	1	1	2	1	0
0	0	1	0	0	0	1	2	1	0
0	0	1	0	0	0	1	2	1	0
0	0	1	0	0	0	0	2	1	0
0	0	1	0	1	0	0	2	1	0
0	0	1	0	1	0	0	2	1	1
0	0	0	0	0	0	0	2	1	0
0	0	0	0	0	0	0	2	1	0
0	0	1	0	1	0	0	2	1	0
0	0	1	0	1	0	0	2	1	0
0	0	1	0	0	0	0	2	1	0
0	0	0	0	0	0	0	2	1	0
0	0	1	0	1	0	0	2	1	0
0	0	1	0	0	0	1	2	1	0
1	1	0	0	0	0	0	2	1	1
0	0	1	0	1	0	0	2	1	0
0	0	1	0	1	0	0	2	1	0
0	0	1	0	1	0	0	2	1	0
0	0	1	0	0	1	0	2	1	0
0	0	1	0	1	0	0	2	1	0
0	0	1	0	1	0	0	2	1	0
0	0	0	0	0	0	0	2	1	0
0	0	1	0	0	0	0	2	1	0
0	0	0	0	0	0	0	2	1	0
0	0	0	0	0	0	0	2	1	0

0	0	1	0	1	0	0	2	1	0
0	0	1	0	0	0	0	2	1	0
1	0	1	0	1	0	0	2	1	0
0	0	1	0		0	1	2	1	0
0	0	1	0	1	0	0	2	1	0
0	0	0	0	0	0	0	2	1	1
0	0	1	0	0	0	0	2	1	0
0	0	1	0	1	0	0	2	1	0
0	0	0	0	0	0	0	2	1	0
1	1	1	0	1	0	0	2	1	0
0	0	1	0	0	0	0	2	1	0
0	0	0	0	0	0	0	2	1	0
0	0	0	0	0	0	0	2	1	0
0	0	1	0	1	1	1	2	1	0
0	0	1	0	0	0	0	2	1	0
0	0	1	0	1	0	0	2	1	0
0	0	1	0	1	0	0	2	1	0
0	0	1	0	1	0	0	2	1	0
0	0	1	0	1	0	0	2	1	0
0	0	1	0	0	0	1	2	1	0
0	0	1	1	1	0	0	2	1	0
0	0	1	0	1	0	0	2	1	1
0	0	1	0	1	0	0	2	1	0
0	0	1	0	0	0	0	2	1	1
0	0	1	0	0	0	0	2	1	0
0	0	0	0	0	0	0	2	1	0
0	0	0	0	0	0	0	2	1	0
0	0	1	0	1	0	0	2	1	0
0	0	1	0	1	0	0	2	1	0
0	0	0	0	0	0	0	2	1	0
0	0	1	0	0	0	0	2	1	0
0	0	1	0	0	0	0	2	1	0
1	0	1	0	1	0	0	2	1	0
0	0	1	0	1	0	0	2	1	0
0	0	1	0	0	0	0	2	1	0
0	0	1	0	0	0	0	2	1	0
0	0	1	0	1	0	0	2	1	0
0	0	0	0	0	0	0	2	1	0
0	0	1	0	1	0	1	2	1	0
0	0	1	0	1	0	0	2	1	0
0	0	1	1	0	1	0	2	1	0
0	0	1	0	0	0	0	2	1	0
0	0	1	0	0	0	0	2	1	0
0	0	1	0	0	0	0	2	1	0
0	0	0	0	0	0	0	2	1	0
0	0	1	0	0	0	0	2	1	0
0	0	1	0	1	0	0	2	1	0

0	0	1	0	0	0	1	2	1	0
0	0	0	0	0	0	0	2	1	0
0	0	0	0	0	0	0	2	1	0
0	0	1	0	1	0	0	2	1	0
0	0	0	0	0	0	0	2	1	0
0	0	0	0	0	0	0	2	1	0
0	0	0	0	0	0	0	2	1	1
0	0	0	0	0	0	0	2	1	0
0	0	1	0	0	0	0	2	1	0
0	0	1	0	0	0	0	2	1	0
0	0	0	0	0	0	0	2	1	0
0	0	0	0	0	0	0	2	1	0
0	0	1	0	0	0	0	2	1	0
0	0	0	0	0	0	0	2	1	0
0	0	0	0	0	0	0	2	1	0
0	0	0	0	0	0	0	2	1	1
0	0	0	0	0	0	0	2	1	0
0	0	0	0	0	0	0	2	1	0
0	0	0	0	0	0	0	2	1	0
0	0	0	0	0	0	0	2	1	1
0	0	0	0	0	0	0	2	1	0
0	0	1	0	1	0	0	2	1	0
0	0	1	0	0	0	1	2	1	0
0	0	1	0	1	1	1	2	1	0
0	0	1	0	1	0	0	2	1	0
0	0	1	0	1	0	0	2	1	0
0	0	1	0	0	0	0	2	1	0
0	0	0	0	0	1	1	2	1	0
0	0	1	0	1	0	1	2	1	0
0	0	1	1	0	1	1	2	1	0
0	0	1	0	1	1	0	2	1	0
0	0	0	0	0	0	0	2	1	0
0	0	1	0	0	0	0	2	1	0
0	0	0	0	0	0	0	2	1	0
0	0	1	0	1	0	0	2	1	0
0	0	1	0	1	0	0	2	1	0
0	1	1	0	1	0	0	2	1	0
9	9	1	0	1	0	0	2	1	0
0	0	1	0	1	0	0	2	1	0
0	0	1	0	1	0	1	2	1	0
0	0	0	1	0	0	0	2	1	0
0	0	1	0	1	0	0	2	1	1
1	0	0	0	1	0	0	2	1	0
0	0	1	0	0	0	0	2	1	0
0	0	1	0	1	0	0	2	1	0
0	0	1	0	1	0	0	2	1	0
0	0	0	0	0	0	0	2	1	0

1	0	1	0	0	0	1	2	1	0
0	0	1	0	1	0	1	2	1	0
1	0	0	0	0	0	0	2	1	0
0	0	1	0	1	1	0	2	1	0
0	0	1	0	1	1	0	2	1	0
0	0	1	0	1	1	0	2	1	0
0	1	1	0	1	0	0	2	1	0
0	0	1	0	1	0	1	2	1	0
0	0	0	1	0	0	1	2	1	0
0	0	0	0	0	0	0	2	1	0
0	0	0	0	0	0	1	2	1	0
0	0	1	0	1	0	1	2	1	0
0	0	1	0	0	0	0	2	1	0
0	0	0	0	0	0	0	2	1	0
0	0	0	0	0	0	0	2	1	0
0	0	0	0	0	0	0	2	1	0
0	0	1	0	0	0	0	2	1	0
0	0	1	0	1	0	0	2	1	0
1	0	1	0	0	0	0	2	1	1
0	0	0	0	0	0	0	2	1	0
0	0	1	0	0	0	0	2	1	0
0	0	1	0	0	0	0	2	1	0
0	0	1	1	1	0	0	2	1	0
0	0	1	0	0	0	1	2	1	0
0	0	1	0	1	1	0	2	1	1
0	0	1	0	1	0	1	2	1	0
0	0	1	0	0	0	0	2	1	0
0	0	1	0	0	0	0	2	1	0
0	0	1	0	0	0	0	2	1	0
0	0	1	1	0	0	1	2	1	0
0	0	0	0	0	0	1	2	1	0
0	0	1	1	1	0	0	2	1	0
0	0	0	0	0	0	0	2	1	0
0	0	0	0	0	0	0	2	1	0
0	0	0	0	0	0	0	2	1	0
0	0	1	0	0	0	1	2	1	0
1	1	0	0	1	0	0	2	1	1
0	0	0	0	0	0	0	2	1	0
0	0	0	0	0	0	0	2	1	0
0	0	1	0	0	0	0	2	1	0
0	0	0	0	0	0	0	2	1	1
0	0	1	0	0	0	0	2	1	0
0	0	0	0	0	0	1	2	1	0
0	0	1	0	1	0	1	2	1	0
0	0	1	0	1	0	0	2	1	0
0	0	0	0	0	0	0	2	1	0
0	0	0	0	1	0	0	2	1	0

0	0	1	0	0	0	0	2	1	0
0	0	1	0	1	0	0	2	1	0
0	0	0	0	0	0	0	2	1	0
0	0	1	0	1	0	0	2	1	0
0	0	1	0	1	0	0	2	1	0
0	0	0	1	0	0	1	2	1	0
0	0	0	1	0	0	1	2	1	0
0	0	1	0	0	1	0	2	1	0
0	0	0	0	0	0	0	2	1	0
0	0	0	0	0	0	0	2	1	0
0	0	1	0	1	0	0	2	1	0
0	1	1	0	1	0	0	2	1	0
0	0	0	0	0	0	0	2	1	0
0	0	1	0	0	0	1	2	1	0
0	0	0	0	0	0	0	2	1	0
0	0	1	1	1	0	0	2	1	0
0	0	0	0	1	0	0	2	1	1
1	0	1	0	0	0	0	2	1	0
0	0	1	0	1	0	0	2	1	0
0	0	1	0	0	0	0	2	1	1
0	0	1	0	1	0	0	2	1	0
0	0	1	0	0	0	0	2	1	0
0	0	0	0	0	0	0	2	1	0
0	0	1	1	1	1	1	2	1	0
1	9	1	1	0		0	2	1	0
0	0	1	0	0		0	2	1	0
0	0	1	1	0	0	0	2	1	0
0	0	1	0	1	0	0	2	1	0
0	0	0	0	0	0	0	2	1	0
0	0	1	0	1	0	0	2	1	0
0	0	1	0	1	0	0	2	1	0
0	0	0	1	0	0	0	2	1	0
0	0	1	0	0	0	0	2	1	0
0	0	1	0	0	0	0	2	1	0
0	0	0	0	0	0	0	2	1	0
0	0	1	1	1	1	1	2	1	0
0	0	0	0	0	0	0	2	1	0
0	0	1	0	0	0	0	2	1	0
0	0	1	0	0	0	0	2	1	1
0	0	0	0	0	0	0	2	1	0
0	0	0	0	0	0	0	2	1	0
0	0	1	0	1	0	1	2	1	1
0	0	0	1	0	0	0	2	1	0
0	0	0	0	0	0	0	2	1	0
0	0	1	0	0	0	0	2	1	0
0	0	1	0	0	1	0	2	1	0
0	0	1	0	1	0	0	2	1	1

0	0	1	1	1	0	0	2	1	0
0	0	1	1	1	0	1	2	1	0
0	0	0	0	0	0	0	2	1	0
0	0	1	0	1	0	0	2	1	0
0	0	1	0	0	0	0	2	1	0
0	0	0	0	0	0	0	2	1	1
0	0	1	0	0	0	0	2	1	0
0	0	0	0	0	0	0	2	1	0
0	0	0	0	0	0	0	2	1	0
0	0	0	0	0	0	0	2	1	0
0	0	1	0	0	0	0	2	1	0
0	0	0	0	0	0	1	2	1	0
0	0	1	0	1	0	0	2	1	0
0	0	1	0	0	0	0	2	1	1
0	0	1	0	0	0	0	2	1	0
0	0	0	1	0	0	0	2	1	0
0	0	1	0	1	0	0	2	1	0
0	0	1	0	0	0	0	2	1	0
1	1	1	0	0	0	1	2	1	0
0	0	1	0	0	0	0	2	1	0
0	0	0	0	1	0	0	2	1	0
0	0	1	0	1	0	0	2	1	0
0	0	0	1	0	0	0	2	1	0
0	0	0	0	0	0	0	2	1	0
0	0	1	0	0	0	0	2	1	0
0	0	1	1	0	0	1	2	1	1
0	0	1	0	0	0	1	2	1	1
0	0	1	0	0	0	0	2	1	0
0	0	0	0	0	0	0	2	1	0
0	0	1	0	0	0	0	2	1	0
0	0	1	0	0	0	0	2	1	0
1	1	1	0	1	0	0	2	1	0
0	0	1	0	0	0	1	2	1	0
0	0	0	1	0	0	1	2	1	0
0	0	0	0	0	0	0	2	1	0
1	0	1	0	0	0	0	2	1	0
0	0	1	0	0	0	0	2	1	0
0	0	0	0	0	0	1	2	1	1
1	1	1	0	1	0	1	2	1	0
0	0	0	0	1	0	1	2	1	0
0	0	1	1	1	1	0	2	1	0
1	0	0	0	0	0	0	2	1	0
0	0	1	1	1	1	0	2	1	1
0	0	1	0	1	0	0	2	1	0
0	0	1	1	0	0	0	2	1	0
9	0	0	0	0	0	0	2	1	0
9	9	0	0	0	0	0	2	1	0

1	0	0	0	0	0	0	2	1	0
0	0	0	0	0	0	0	2	1	0
0	0	1	0	0	0	0	2	1	0
0	0	1	0	1	1	1	2	1	0
0	0	0	0	0	0	0	2	1	0
0	0	0	0	0	0	0	2	1	0
0	0	1	0	0	1	1	2	1	0
0	0	1	0	0	0	0	2	1	0
0	0	1	0	0	0	0	2	1	0
0	0	0	0	0	0	0	2	1	0
0	0	0	0	0	0	0	2	1	0
0	0	0	0	0	0	0	2	1	0
0	0	1	1	1	0	0	2	1	0
1	1	0	1	0	0	0	2	1	0
0	0	1	1	1	1	0	2	1	0
0	0	1	1	1	1	1	2	1	0
0	0	0	0	0	0	0	2	1	0
0	0	1	1	1	1	0	2	1	0
0	0	1	0	0	0	0	2	1	0
0	0	0	0	0	0	0	2	1	0
1	1	1	0	0	0	0	2	1	0
0	0	1	0	0	0	0	2	1	0
1	1	0	0	0	0	0	2	1	0
0	0	1	0	0	0	0	2	1	0
9	0	0	0	0	0	0	2	1	0
0	0	0	0	0	0	0	2	1	0
0	0	1	0	0	0	0	2	1	0
0	0	1	0	0	0	0	2	1	0
0	0	1	0	0	0	0	2	1	1
0	0	1	0	0	0	0	2	1	0
0	0	1	1	0	0	0	2	1	0
0	0	1	0	0	0	0	2	1	1
1	1	1	0	0	0	0	2	1	0
0	0	1	1	1	1	1	2	1	0
0	0	1	0	0	0	0	2	1	0
1	0	1	1	1	1	0	2	1	1
0	0	1	1	1	1	1	2	1	0
0	0	1	0	0	0	0	2	1	0
0	0	1	0	1	1	1	2	1	0
0	0	1	0	0	0	0	2	1	0
0	0	1	0	0	0	0	2	1	0
0	0	0	0	0	0	0	2	1	0
0	0	0	0	0	0	1	2	1	0
0	0	0	0	0	0	0	2	1	0
0	0	0	0	0	0	0	2	1	0
0	0	0	0	0	0	0	2	1	0
0	0	1	0	0	0	0	2	1	0

0	0	1	0	0	0	0	2	1	0
0	0	1	0	0	0	1	2	1	0
0	0	1	0	0	0	1	2	1	0
0	0	1	1	1	0	1	2	1	0
0	0	1	1	1	0	1	2	1	0
0	0	1	0	0	0	0	2	1	0
0	0	1	0	0	0	0	2	1	0
0	0	1	0	0	0	0	2	1	0
1	1	0	0	0	0	0	2	1	0
0	0	0	0	0	0	1	2	1	0
0	0	1	1	1	1	1	2	1	0
0	0	1	1	1	0	1	2	1	0
0	0	1	0	0	0	0	2	1	0
1	1	1	0	0	0	0	2	1	0
0	0	1	1	1	0	0	2	1	0
0	0	0	0	0	0	0	2	1	0
0	0	1	1	1	0	0	2	1	0
0	0	0	0	1	0	1	2	1	0
0	0	0	0	0	0	0	2	1	0
1	0	0	0	0	0	0	2	1	0
0	0	1	1	1	1	0	2	1	0
0	0	0	0	0	0	0	2	1	0
0	0	1	0	1	0	0	2	1	0
0	0	0	0	0	0	0	2	1	0
0	0	1	1	1	1	0	2	1	0
0	0	1	0	1	1	1	2	1	0
0	0	0	0	0	0	0	2	1	0
0	0	1	0	0	0	0	2	1	0
0	0	0	0	0	0	0	2	1	0
0	0	0	0	0	0	1	2	1	0
0	0	0	0	0	0	1	2	1	0
1	1	1	0	1	0	0	2	1	0
0	0	1	1	1	0	0	2	1	0
0	1	0	0	0	0	0	2	1	0
0	0	0	0	0	0	0	2	1	0
0	0	0	0	0	0	0	2	1	0
0	0	0	0	0	0	0	2	1	0
0	0	1	1	1	1	1	2	1	0
0	0	1	0	0	0	0	2	1	0
0	0	1	0	1	0	0	2	1	0
0	0	1	0	0	0	0	2	1	0
0	0	0	0	0	0	0	2	1	0
0	0	0	0	0	0	0	2	1	0
0	0	1	1	1	1	1	2	1	0
0	0	1	1	0	0	1	2	1	0
0	0	0	1	0	0	1	2	1	0
0	0	0	0	0	0	0	2	1	0

0	0	0	0	0	0	0	2	1	0
0	0	0	0	0	0	1	2	1	0
0	0	0	0	0	0	0	2	1	0
0	0	1	0	0	0	0	2	1	0
0	0	0	1	0	0	1	2	1	0
0	0	0	0	0	0	0	2	1	0
0	0	1	1	0	1	1	2	1	0
0	0	0	0	0	0	0	2	1	0
0	0	0	0	0	0	1	2	1	0
1	0	1	0	0	0	0	2	1	1
0	0	1	0	1	0	0	2	1	0
0	0	1	0	1	0	1	2	1	0
0	0	1	0	1	0	0	2	1	0
0	0	1	0	0	0	0	2	1	0
0	0	0	0	0	0	0	2	1	0
0	0	0	1	0	0	0	2	1	0
0	0	0	0	0	0	0	2	1	0
0	0	0	0	0	0	0	2	1	0
0	0	1	0	1	0	0	2	1	0
0	0	0	0	0	0	0	2	1	0
0	0	1	0	1	0	0	2	1	0
0	0	1	0	0	0	1	2	1	0
0	0	0	0	0	0	0	2	1	0
0	0	1	1	0	0	0	2	1	0
0	0	0	0	0	0	0	2	1	0
0	0	1	0	1	0	0	2	1	1
0	0	0	0	1	0	0	2	1	0
0	0	0	0	1	0	0	2	1	1
0	0	0	0	1	0	0	2	1	0
0	0	0	0	0	0	0	2	1	0
0	0	1	1	0	0	1	2	1	0
0	0	1	1	1	0	0	2	1	0
0	0	1	0	1	0	0	2	1	0
0	0	1	1	1	0	1	2	1	0
0	0	0	1	0	0	1	2	1	0
0	0	0	0	0	0	0	2	1	0
0	0	0	0	0	0	0	2	1	0
0	0	1	0	0	0	0	2	1	0
0	0	1	0	1	0	0	2	1	0
0	0	1	1	1	0	0	2	1	0
1	1	1	0	1	0	1	2	1	1
0	0	0	0	0	0	0	2	1	0
0	0	0	0	0	0	0	2	1	0
0	0	0	0	0	0	0	2	1	0
0	0	1	1	0	0	0	2	1	0
0	0	0	0	0	0	0	2	1	0
0	0	0	0	0	0	0	2	1	0

0	0	1	0	1	0	0	2	1	0
0	0	0	0	0	0	0	2	1	0
0	0	1	0	0	0	0	2	1	0
0	0	1	0	1	0	1	2	1	0
0	0	1	0	1	0	0	2	1	0
0	0	0	0	0	1	0	2	1	0
0	0	1	1	1	0	1	2	1	0
0	0	0	0	0	0	0	2	1	0
0	0	1	0	0	0	0	2	1	0
0	0	1	0	0	0	0	2	1	0
0	0	1	0	1	0	0	2	1	0
0	0	0	0	0	0	0	2	1	0
0	0	0	0	0	0	0	2	1	0
0	0	1	1	0	1	1	2	1	0
0	0	1	0	1	0	0	2	1	0
0	0	0	0	0	0	0	2	1	0
0	0	1	0	1	0	0	2	1	0
0	0	1	0	0	0	0	2	1	0
0	0	0	0	0	0	0	2	1	0
0	0	1	1	1	0	0	2	1	0
0	0	1	0	1	0	0	2	1	0
0	0	1	0	0	0	0	2	1	0
0	0	1	0	0	0	0	2	1	0
0	0	1	0	1	0	0	2	1	0
0	0	1	0	0	0	0	2	1	0
0	0	1	0	1	0	0	2	1	0
0	0	1	0	1	0	0	2	1	0
0	0	1	0	1	0	0	2	1	0
0	0	1	0	1	0	0	2	1	0
0	0	1	0	0	1	0	2	1	1
0	0	1	0	0	0	0	2	1	0
0	0	1	1	1	0	1	2	1	1
0	0	0	0	1	0	0	2	1	0
0	0	0	0	0	0	0	2	1	0
0	0	0	0	0	0	0	2	1	0
0	0	0	0	0	0	0	2	1	0
0	0	1	0	1	0	0	2	1	0
0	0	1	0	1	0	0	2	1	0
0	0	1	0	1	0	1	2	1	0
0	0	1	0	0	0	0	2	1	0
0	0	1	0	0	0	0	2	1	0
0	0	1	0	0	0	0	2	1	0
0	0	0	0	0	0	0	2	1	0
0	0	1	0	0	0	1	2	1	0
0	0	1	0	1	0	0	2	1	0
0	0	0	0	0	0	0	2	1	0
0	0	1	0	1	0	0	2	1	0

0	1	1	0	0	0	1	2	1	0
0	0	1	0	1	0	1	2	1	0
0	0	1	0	1	0	0	2	1	0
0	0	1	1	0	0	1	2	1	0
0	0	0	0	0	0	0	2	1	0
0	0	0	0	0	0	0	2	1	0
0	0	0	0	0	0	0	2	1	0
0	0	0	0	0	0	0	2	1	0
0	0	0	0	0	0	0	2	1	0
0	0	0	0	0	0	0	2	1	0
0	0	1	0	0	0	0	2	1	0
0	0	1	0	0	0	0	2	1	0
0	0	1	0	0	0	0	2	1	0
0	0	1	0	0	0	0	2	1	0
0	0	0	1	1	1	1	2	1	0
0	0	0	0	0	0	0	2	1	0
0	0	0	0	0	0	0	2	1	0
0	0	0	0	0	0	0	2	1	0
0	0	0	0	0	0	0	2	1	0
0	0	0	0	0	0	0	2	1	0
0	0	1	0	0	0	0	2	1	0
0	0	0	0	0	0	0	2	1	0
0	0	1	0	0	0	0	2	1	0
0	0	1	0	0	0	0	2	1	0
0	0	0	0	0	0	0	2	1	0
0	0	0	0	0	0	0	2	1	0
0	0	1	1	1	0	0	2	1	0
0	0	1	0	0	0	0	2	1	0
0	0	0	0	1	0	1	2	1	0
0	0	0	0	0	0	0	2	1	0
0	0	1	0	1	0	0	2	1	0
0	0	1	1	0	0	0	2	1	0
1	1	1	0	0	0	0	2	1	0
0	0	1	1	1	1	1	2	1	0
0	0	1	0	0	0	0	2	1	1
0	0	1	1	1	0	0	2	1	0
0	0	0	0	0	0	0	2	1	0
0	0	1	0	1	0	0	2	1	1
0	0	1	0	1	0	0	2	1	0
0	1	0	1	0	0	0	2	1	0
0	0	1	1	0	0	1	2	1	0
0	0	1	0	0	0	0	2	1	0
0	0	0	0	0	0	0	2	1	0
0	0	1	0	0	0	0	2	1	0
0	0	0	0	0	0	0	2	1	0
0	0	1	0	1	0	1	2	1	0
0	0	1	0	1	0	1	2	1	0

0	0	1	0	1	0	0	2	1	0
0	0	1	0	1	0	0	2	1	0
0	0	1	1	1	1	1	2	1	0
0	0	0	0	0	0	1	2	1	0
0	0	1	0	0	0	0	2	1	0
0	0	0	0	0	0	0	2	1	1
0	0	1	1	1	0	1	2	1	0
0	0	1	0	0	0	1	2	1	0
0	0	1	0	0	0	0	2	1	0
0	0	1	1	1	0	0	2	1	0
0	0	1	0	0	0	0	2	1	0
0	0	1	0	1	0	1	2	1	0
0	0	1	0	1	0	0	2	1	0
0	0	0	0	0	0	0	2	1	0
0	0	1	0	1	0	0	2	1	0
0	0	0	0	0	0	0	2	1	0
0	0	1	0	0	1	0	2	1	0
0	0	1	0	0	0	0	2	1	0
0	0	0	0	0	0	1	2	1	0
0	0	1	0	1	0	1	2	1	0
0	0	1	0	0	0	0	2	1	0
0	0	1	0	1	0	0	2	1	0
0	0	0	0	0	0	1	2	1	0
0	0	0	0	0	0	0	2	1	0
0	0	0	0	0	0	0	2	1	0
0	0	0	0	0	0	0	2	1	0
0	0	0	0	0	0	0	2	1	0
0	0	0	0	0	0	0	2	1	0
0	0	1	0	0	0	0	2	1	0
0	0	0	0	0	0	0	2	1	0
0	0	1	1	1	0	0	2	1	0
0	0	0	0	0	0	0	2	1	0
0	0	0	0	0	0	0	2	1	0
0	0	0	0	0	0	0	2	1	0
1	0	0	0	0	0	0	2	1	0
0	0	0	0	0	0	0	2	1	0
0	0	0	0	0	0	0	2	1	0
0	0	1	0	0	0	0	2	1	0
1	0	1	1	1	0	1	2	1	1
0	0	1	0	1	0	1	2	1	0
0	0	0	0	0	0	0	2	1	0
0	0	0	1	0	0	1	2	1	0
0	0	0	0	0	0	0	2	1	0
0	0	1	1	1	1	1	2	1	1
0	0	1	0	0	0	0	2	1	0
1	1	1	1	1	1	1	2	1	1
0	0	0	0	0	0	0	2	1	1

0	0	0	0	1	0	1	2	1	0
1	0	0	0	0	0	0	2	1	0
0	0	0	0	0	0	0	2	1	0
0	0	0	0	0	0	0	2	1	0
0	0	1	1	0	0	1	2	1	0
1	0	0	0	0	0	0	2	1	1
0	0	0	0	0	0	1	2	1	0
0	0	0	0	0	0	1	2	1	0
0	0	1	0	1	0	1	2	1	0
1	0	1	0	0	0	0	2	1	1
0	0	0	0	0	0	0	2	1	0
0	0	1	1	0	0	0	2	1	0
0	1	1	1	0	0	0	2	1	1
0	0	0	0	0	0	1	2	1	1
0	0	1	0	1	0	1	2	1	0
0	0	1	0	1	0	0	2	1	0
0	0	1	1	0	0	0	2	1	0
0	0	0	0	0	1	0	2	1	0
0	0	0	0	0	1	0	2	1	0
0	0	1	0	0	0	0	2	1	0
0	0	0	0	0	0	0	2	1	0
0	0	0	1	0	1	1	2	1	0
1	0	1	0	1	0	1	2	1	0
0	0	1	0	0	0	1	2	1	0
0	0	1	0	0	0	0	2	1	0
0	0	0	0	0	0	0	2	1	0
0	0	1	0	0	0	1	2	1	0
0	0	0	0	0	0	0	2	1	0
1	1	1	0	0	0	0	2	1	1
1	0	1	1	0	0	0	2	1	0
0	0	1	0	0	0	0	2	1	0
0	0	0	0	0	0	0	2	1	1
0	0	0	0	0	0	0	2	1	0
0	0	1	0	0	0	0	2	1	1
0	0	1	1	1	0	0	2	1	0
0	0	1	0	0	0	0	2	1	0
0	0	1	1	1	0	0	2	1	0
0	0	1	1	1	0	0	2	1	0
0	0	1	0	0	0	0	2	1	1
0	0	0	0	0	0	0	2	1	0
0	0	1	0	0	0	0	2	1	0
0	0	1	0	0	0	0	2	1	0
1	1	0	0	0	0	0	2	1	0
1	1	1	0	0	0	0	2	1	0
0	0	1	1	1	0	0	2	1	1
0	0	1	1	1	1	1	2	1	0
0	0	1	1	1	0	0	2	1	0

0	0	1	0	0	0	0	2	1	0
0	0	1	0	0	0	0	2	1	0
1	1	1	1	1	0	0	2	1	0
0	0	1	1	1	0	1	2	1	0
0	0	1	0	0	0	0	2	1	0
0	0	0	0	0	0	0	2	1	0
0	0	1	0	0	0	0	2	1	0
0	0	1	0	0	0	0	2	1	0
0	0	1	0	0	0	0	2	1	0
0	0	1	0	0	0	0	2	1	0
0	0	0	0	0	0	0	2	1	0
0	0	0	0	0	0	0	2	1	0
0	0	1	0	0	0	0	2	1	0
0	0	0	0	0	0	0	2	1	0
0	0	0	0	0	0	1	2	1	0
0	0	0	0	0	0	0	2	1	0
0	0	0	0	0	0	0	2	1	0
0	0	0	0	0	0	0	2	1	0
0	0	0	0	0	0	0	2	1	0
0	1	0	0	0	0	0	2	1	0
0	0	0	1	1	0	0	2	1	0
0	0	0	0	0	0	0	2	1	0
0	1	0	0	0	0	0	2	1	0
0	0	1	0	0	0	0	2	1	0
0	0	1	0	0	0	1	2	1	0
0	0	1	1	0	0	1	2	1	0
0	0	0	0	0	0	0	2	1	0
0	0	0	0	0	0	0	2	1	0
0	0	0	0	0	0	0	2	1	0
1	1	0	0	0	0	0	2	1	0
0	0	1	0	0	0	0	2	1	0
0	0	0	0	0	0	0	2	1	0
0	0	0	0	0	0	0	2	1	0
0	0	1	0	1	0	0	2	1	0
0	0	1	0	0	0	0	2	1	0
0	0	0	0	0	0	0	2	1	0
0	0	1	0	1	1	0	2	1	0
0	0	0	0	0	0	0	2	1	0
0	0	0	0	0	0	0	2	1	0
0	0	0	0	0	0	0	2	1	0
0	0	0	0	0	0	0	2	1	0
0	0	0	0	0	0	0	2	1	0
0	0	1	0	1	0	0	2	1	0
0	0	1	1	1	0	0	2	1	0
0	0	0	0	0	0	0	2	1	0
1	0	1	0	1	1	0	2	1	0
0	0	1	1	0	0	1	2	1	1

0	0	1	0	1	0	0	2	1	0
0	0	1	0	1	0	0	2	1	0
0	0	1	0	1	0	0	2	1	0
0	0	1	0	0	0	0	2	1	0
0	0	1	0	1	0	0	2	1	0
0	0	0	0	0	0	0	2	1	0
0	0	0	0	0	0	0	2	1	0
0	0	0	0				2	1	0
0	0	1	0	1	0	0	2	1	0
0	0	0	0	0	0	0	2	1	0
0	0	0	0	0	0	0	2	1	0
0	0	0	0	0	0	0	2	1	0
0	0	0	0	0	0	0	2	1	1
0	0	1	0	0	0	0	2	1	0
0	0	1	0	0	1	0	2	1	0
0	0	1	0	0	0	0	2	1	1
0	0	1	0	0	0	0	2	1	0
0	0	1	0	1	0	0	2	1	0
0	0	1	0	0	0	0	2	1	1
0	0	0	0	0	0	0	2	1	0
0	0	0	0	0	0	0	2	1	0
0	0	1	0	1	0	0	2	1	0
0	0	1	1	0	1	0	2	1	0
0	0	1	0	1	0	0	2	1	0
0	1	1	0	1	0	0	2	1	0
0	0	1	0	1	0	0	2	1	0
0	0	1	0	1	0	0	2	1	0
0	0	1	0	0		0	2	1	0
0	0	0	0	0	0	0	2	1	0
0	0	1	0	0	0	0	2	1	0
0	0	0	0	0	0	0	2	1	0
0	0	1	0	1	0	0	2	1	0
0	0	1	1	0	0	0	2	1	0
0	0	1	0	0	0	1	2	1	0
0	0	1	0	0	0	0	2	1	0
0	0	0	0	0	0	0	2	1	0
0	0	1	0	0	0	0	2	1	0
0	0	1	0	0	0	0	2	1	0
0	0	1	0	1	0	0	2	1	0
0	0	1	0	0	0	0	2	1	0
0	0	1	0	1	0	0	2	1	0
0	0	1	0	1	0	0	2	1	0
0	0	1	0	0	1	1	2	1	0
0	0	1	0	1	0	0	2	1	0
0	0	0	0	0	0	0	2	1	0
0	0	1	0	0	0	0	2	1	0
0	0	1	0	0	1	0	2	1	0

0	0	1	0	0	0	0	2	1	0
0	0	1	0	1	0	0	2	1	0
0	0	1	0	1	0	0	2	1	0
0	0	1	0	0	1	0	2	1	0
0	0	1	0	1	1	1	2	1	0
0	0	0	1	0	0	0	2	1	0
0	0	0	0	0	0	0	2	1	0
1	1	1	1	0	0	0	2	1	0
0	0	1	0	0	0	0	2	1	0
0	0	0	0	0	0	0	2	1	0
0	0	0	0	0	0	0	2	1	0
0	0	0	0	0	0	0	2	1	0
0	0	0	0	0	0	0	2	1	0
0	0	0	0	0	0	0	2	1	0
0	0	0	0	0	0	0	2	1	0
1	1	0	0	0	0	0	2	1	0
0	0	0	0	0	0	0	2	1	0
0	0	1	1	1	1	0	2	1	0
0	0	1	1	1	1	0	2	1	0
0	0	1	0	1	0	0	2	1	0
0	0	1	1	1	1	1	2	1	0
0	0	0	0	0	0	0	2	1	1
0	0	0	0	0	0	0	2	1	0
0	0	1	0	1		0	2	1	0
0	0	1	0	0	0	1	2	1	0
0	0	1	0	0	0	0	2	1	0
0	0	1	0	1	0	0	2	1	0
0	1	1	1	1	0	0	2	1	0
0	0	0	0	0	0	0	2	1	0
0	0	0	1	0	0	1	2	1	1
0	0	1	0	0	0	1	2	1	0
0	0	0	0	0	0	0	2	1	0
0	0	1	0	1	0	0	2	1	0
0	0	1	1	1	1	1	2	1	1
0	0	1	0	0		0	2	1	0
0	0	0	0	0	0	0	2	1	0
0	0	1	0	0	0	0	2	1	0
0	0	1	0	0	0	0	2	1	0
0	0	1	1	1	0	0	2	1	0
0	0	1	0	1	0	0	2	1	0
0	0	0	0	0	0	0	2	1	0
0	0	1	1	1	0	0	2	1	0
0	0	1	0	1	0	0	2	1	0
0	0	0	0	0	0	0	2	1	0
0	0	0	0	0	0	0	2	1	0
0	0	1	1	1	1	1	2	1	0
0	0	1	1	0	0	1	2	1	0

0	0	1	1	1	1	0	2	1	0
1	0	1	0	1	0	0	2	1	0
0	0	0	0	0	0	0	2	1	0
0	0	1	0	1	0	1	2	1	0
0	0	0	0	0	1	1	2	1	0
0	0	0	0	1	0	0	2	1	0
0	0	0	0	0	0	0	2	1	0
0	0	1	0	1	0	0	2	1	0
0	0	1	0	0	0	0	2	1	0
0	0	1	1	0	0	0	2	1	0
0	0	0	0	0	0	0	2	1	0
0	0	1	1	1	0	0	2	1	0
1	1	1	0	1	0	0	2	1	0
0	0	1	0	1	0	0	2	1	0
0	0	1	0	0	0	0	2	1	0
0	0	1	0	0	0	0	2	1	1
0	0	0	0	0	0	0	2	1	0
0	0	1	0	0	0	0	2	1	0
0	0	1	0	0	0	0	2	1	0
0	0	0	0	0	0	0	3	1	0
0	0	1	0	1	0	0	3	1	0
0	0	1	0	0	0	0	3	1	0
0	0	1	0	1	0	0	3	1	1
0	0	0	0	0	0	0	3	1	0
0	0	0	0	0	0	0	3	1	0
0	0	1	0	0		1	3	1	0
0	0	1	0	0	0	0	3	1	0
0	0	1	0	0	0	1	3	1	1
0	0	0	0	0		0	3	1	0
0	0	1	0	0	0	0	3	1	0
0	0	1	0	1	0	1	3	1	0
0	0	1	1	1	0	1	3	1	0
0	0	0	0	0	0	0	3	1	0
0	0	1	1	1	1	1	3	1	0
0	0	1	0	0	0	0	3	1	1
1	0	1	0	1	0	0	3	1	1
0	0	0	0	0	0	0	3	1	0
0	0	1	0	1	0	1	3	1	0
0	0	1	0	1	0	1	3	1	0
0	0	0	0	0	0	0	3	1	0
1	0	1	1	1	1	1	3	1	1
0	0	0	1	0	0	0	3	1	0
1	1	0	0	0	0	0	3	1	0
0	0	0	0	0	0	0	3	1	0
0	0	0	1	0	0	0	3	1	0
0	0	0	1	0	0	1	3	1	0
0	0	1	0	1	0	0	3	1	0

0	0	1	1	1	0	0	3	1	0
0	0	1	1	0	0	0	3	1	0
0	0	1	1	0	0	0	3	1	0
0	0	0	0	1	0	0	3	1	0
0	0	0	0	0	0	0	3	1	0
0	0	0	1	0	0	1	3	1	0
0	0	1	0	1	0	0	3	1	0
0	0	0	0	1	0	0	3	1	0
0	0	0					3	1	1
0	0	0	1	1	0	0	3	1	0
0	0	0	0	0	0	0	3	1	0
0	0	0					3	1	1
0	0	0	0	0	0	0	3	1	1
0	0	1	1	1	0	0	3	1	0
0	0	1	1	0	0	1	3	1	0
0	0	1	1	1	1	1	3	1	0
0	0	1	0	0	0	0	3	1	0
0	0	1	1	1	1	1	3	1	0
0	0	1	1	1	0	1	3	1	0
0	0	1	1	1	0	1	3	1	0
0	0	0	0	0	0	0	3	1	0
0	0	1	1	1	1	1	3	1	0
0	0	0	0	1	0	0	3	1	0
0	0	1	1	1	1	1	3	1	0
0	0	0	0	0	0	0	3	1	0
0	0	1	0	0	0	0	3	1	0
0	1	1	0	0	0	0	3	1	0
0	0	1	1	1	0	0	3	1	0
0	0	1	0	0	0	0	3	1	0
0	0	0	1	1	0	1	3	1	0
0	0	1	1	1	0	0	3	1	0
0	0	0	0	0	0	0	3	1	0
0	1	0	0	0	0	0	3	1	0
0	0	1	0	0	0	0	3	1	0
0	0	1	1	0	0	0	3	1	0
0	0	0	0	0	1	1	3	1	0
0	0	0	0	0	0	0	3	1	0
0	0	1	1	1	0	1	3	1	0
0	0	0	0	0	0	0	3	1	0
0	0	0	0	0	0	0	3	1	0
0	0	0	0	0	0	0	3	1	1
0	0	1	0	0	0	1	3	1	0
0	0	1	1	0	0	1	3	1	0
0	0	0	0	0	0	0	3	1	0
0	0	0	0	0	0	1	3	1	0
0	0	0	0	0	0	0	3	1	0
0	0	1	0	1	0	1	3	1	0

0	0	1	1	0	0	0	3	1	0
0	0	1	0	0	0	1	3	1	0
0	0	1	1	1	0	1	3	1	0
1	0	0	0	0	0	0	3	1	0
0	1	1	0	0	0	0	3	1	0
0	0	1	0	1	0	0	3	1	0
0	0	1	0	0	0	0	3	1	0
0	1	1	1	1	1	1	3	1	0
0	0	1	0	1	0	1	3	1	0
0	0	0	0	0	0	0	3	1	0
0	0	0	0	0	0	0	3	1	0
0	0	1	0	0	0	0	3	1	0
0	0	1	0	0	0	0	3	1	0
0	1	1	0	0	0	0	3	1	0
0	0	1	0	0	0	0	3	1	0
0	0	1	1	0	0	0	3	1	0
0	0	0	0	0	0	0	3	1	0
0	0	1	0	1	0	0	3	1	0
0	0	1	0	1	0	0	3	1	0
0	1	1	0	0	0	0	3	1	0
0	0	1	0	1	0	1	3	1	0
0	0	1	1	1	0	0	3	1	0
0	0	1	0	1	0	0	3	1	0
0	0	1	0	0	0	0	3	1	0
0	0	1	0	1	0	0	3	1	0
0	0	0	0	0	0	0	3	1	0
0	0	0	0	0	0	0	3	1	0
0	0	1	0	0	0	0	3	1	0
0	0	1	0	1	0	1	3	1	0
0	0	1	0	0	1	0	3	1	0
0	0	1	0	1	0	0	3	1	0
0	0	1	0	1	0	1	3	1	0
0	0	1	0	1	0	0	3	1	0
0	0	0	0	0	0	0	3	1	0
0	0	0	0	0	0	0	3	1	0
0	0	1	0	1	0	0	3	1	0
0	0	1	0	0	0	1	3	1	1
0	0	0	0	0	0	0	3	1	0
0	0	1	0	0	0	0	3	1	0
0	0	1	0	0	0	0	3	1	0
0	0	1	1	0	0	0	3	1	1
0	0	1	0	0	0	0	3	1	0
0	0	1	0	1	0	0	3	1	0
0	0	1	0	0	0	0	3	1	0
0	0	1	0	0	0	0	3	1	0
0	0	1	0	1	0	0	3	1	0
0	0	1	0	0	0	0	3	1	0

0	0	1	0	1	0	0	3	1	0
0	0	0	0	0	0	0	3	1	0
0	0	1	0	0	0	0	3	1	0
0	0	1	0	1	0	0	3	1	0
0	0	1	0	1	0	0	3	1	0
0	0	0	0	0	0	0	3	1	0
0	0	0	0	0	0	0	3	1	0
0	0	0	0	0	0	0	3	1	0
0	0	0	0	0	0	0	3	1	0
0	0	0	0	0	0	0	3	1	0
0	0	0	0	0	0	0	3	1	0
9	0	0	0	0	0	0	3	1	0
0	0	0	0	0	0	0	3	1	0
0	0	1	0	0	0	0	3	1	0
0	0	0	0	0	0	0	3	1	0
0	0	0	0	0	0	0	3	1	0
0	0	0	0	0	0	0	3	1	1
9	0	1	0	0	0	0	3	1	0
0	0	0	0	0	0	0	3	1	0
0	0	1	0	0	0	1	3	1	0
0	0	0	0	0	0	0	3	1	0
0	0	0	0	1	1	0	3	1	0
0	0	0	0	0	1	0	3	1	0
0	0	1	1	1	1	0	3	1	0
0	0	0	1	1	1	0	3	1	0
0	0	1	0	1	0	0	3	1	0
0	0	1	0	1	0	1	3	1	1
0	0	0	0	0	0	0	3	1	0
0	0	1	1	1		0	3	1	0
0	0						3	1	0
0	0	0	0	0	0	0	3	1	0
0	0	0	0	0	0	0	3	1	1
0	0	0	0	0	0	0	3	1	0
0	0	1	0	1	1	1	3	1	0
0	1	1	0	1	0	0	3	1	0
0	0	0	0	0	0	0	3	1	0
0	0	1	1	0	0	1	3	1	0
9	0	1	1	1	0	0	3	1	0
0	0	0	0	0	0	0	3	1	0
0	0	0	0	0	0	0	3	1	0
0	0	1	0	0	1	1	3	1	0
0	0	0	0	0	0	0	3	1	0
0	0	1	0	0	0	0	3	1	0
0	0	1	0	1	0	1	3	1	0
0	0	1	0	0	0	0	3	1	0
0	0	0	0	0	0	0	3	1	0
0	0	1	0	1	0	0	3	1	0

0	0	0	0	0	0	0	3	1	0
0	0	1	1	1	0	1	3	1	0
0	1	1	1	1	0	1	3	1	0
0	0	1	0	0	0	0	3	1	1
0	0	0	0	0	0	0	3	1	0
0	0	0	1	0	1	0	3	1	1
0	0	1	1	1	0	1	3	1	0
0	0	1	0	0	0	0	3	1	0
0	0	0	0	0	0	1	3	1	1
0	0	0	0	0	0	0	3	1	0
0	0	1	0	0	0	1	3	1	0
0	0	0	0	0	0	1	3	1	0
0	0	0	0	0	0	0	3	1	0
0	0	0	0	0	0	0	3	1	0
0	0	0	0	0	0	0	3	1	1
0	0	0	0	0	0	0	3	1	0
0	0	1	0	0	0	0	3	1	0
0	0	0	1	0	1	0	3	1	0
0	0	1	1	1		1	3	1	0
0	0	0	0	0	0	0	3	1	0
0	0	0	0	0	1	1	3	1	0
0	0	0	0	0	0	0	3	1	0
0	1	1	0	1	0	0	3	1	0
0	0	1	0	0	0	0	3	1	0
0	0	1	0	0	0	0	3	1	0
0	0	0	1	0	0	0	3	1	0
1	0	1	0	0	0	0	3	1	0
0	0	0	1	0	1	1	3	1	0
0	0	1	0	1	0	0	3	1	0
0	0	0	0	1	0	0	3	1	0
0	0	1	0	0	0	0	3	1	0
0	0	1	0	0	0	0	3	1	0
0	0	0	1	0	0	0	3	1	0
0	0	0	1	0	0	0	3	1	0
0	0	0	1	0	0	0	3	1	0
0	0	0	1	0	0	0	3	1	0
1	0	0	1	0	1	1	3	1	0
0	0	0	0	0	0	0	3	1	0
0	0	1	0	0	0	1	3	1	0
0	0	0	0	0	0	1	3	1	0
0	0	1	0	1	0	1	3	1	0
0	0	1	0	1	0	0	3	1	0
0	0	1	0	1	0	0	3	1	0
0	0	1	0	1	0	0	3	1	0
0	0	1	0	0	0	0	3	1	0
0	0	1	0	0	0	0	3	1	0
0	0	1	0	0	0	0	3	1	0

0	0	0	0	0	0	0	3	1	0
0	0	0	0	0	0	0	3	1	0
0	0	1	0	0	0	0	3	1	0
0	0	0	0	0	0	0	3	1	0
0	0	1	1	1	0	0	3	1	0
0	0	1	0	0	0	0	3	1	0
0	0	1	0	0	0	0	3	1	0
1	0	1	0	0	0	0	3	1	0
0	0	1	0	0	0	0	3	1	1
0	0	0	0	0	0	0	3	1	1
0	1	1	1	1	0	0	3	1	0
0	0	1	1	1	0	0	3	1	1
0	0	1	1	1	1	1	3	1	0
0	0	1	1	1	1	1	3	1	0
0	0	1	0	0	0	0	3	1	0
0	0	0	0	0	0	0	3	1	0
0	0	1	1	0	0	0	3	1	0
0	0	1	1	0	0	0	3	1	0
0	0	1	0	0	0	1	3	1	0
0	0	0	0	0	0	0	3	1	0
0	0	0	0	1	0	0	3	1	0
0	0	0	0	0	0	1	3	1	0
1	0	1	1	1	1	1	3	1	0
0	0	0	0	0	0	0	3	1	0
0	0	0	0	0	0	0	3	1	0
0	0	1	0	1	0	0	3	1	0
0	0	0	0	0	0	0	3	1	0
0	0	1	0	0	0	0	3	1	0
0	0	0	0	0	0	0	3	1	0
0	0	0	0	0	0	0	3	1	0
0	0	0	0	1	0	0	3	1	0
0	0	0	0	0	0	0	3	1	0
0	0	0	0	0	0	1	3	1	0
0	0	0	0	0	0	0	3	1	0
0	0	1	0	0	0	0	3	1	0
0	0	1	0	0	0	0	3	1	0
0	0	1	0	1	0	0	3	1	0
0	0	0	0	0	0	0	3	1	0
0	0	1	0	0	0	1	3	1	0
0	0	0	0	0	0	1	3	1	0
1	1	1	1	0	0	1	3	1	1
0	0	0	0	0	0	0	3	1	0
0	0	1	0	0	0	0	3	1	0
0	0	0	0	0	0	1	3	1	0
0	0	0	0	0	0	1	3	1	0
0	0	0	0	0	0	0	3	1	0
0	0	0	0	0	0	0	3	1	0

0	0	1	0	0	0	1	3	1	1
0	0	0	0	0	0	0	3	1	0
0	0	1	0	0	0	0	3	1	0
0	0	1	0	0	0	0	3	1	0
0	0	1	1	1	0	1	3	1	0
0	0	1	0	0	0	0	3	1	0
0	0	0	0	0	0	0	3	1	0
0	0	0	0	0	0	0	3	1	0
0	0	0	0	0	0	0	3	1	1
0	0	1	0	1	0	0	3	1	0
0	0	1	1	0	1	1	3	1	0
0	0	1	0	1	0	0	3	1	0
0	0	1	0	1	0	0	3	1	0
0	0	0	0	1	0	1	3	1	0
0	0	0	0	0	0	0	3	1	0
0	0	0	0	0	0	0	3	1	0
0	0	1	1	0	0	0	3	1	0
0	1	1	1	1	1	0	3	1	0
0	0	1	0	1	0	0	3	1	0
0	0	1	0	1	0	0	3	1	0
0	0	1	1	1	0	0	3	1	0
0	0	1	0	1	0	0	3	1	0
0	0	1	0	0	0	0	3	1	0
0	0	1	0	0	1	0	3	1	0
0	0	1	0	1	0	0	3	1	0
0	0	0	0	0	0	0	3	1	0
0	0	0	0	0	0	0	3	1	0
0	0	0	0	0	0	0	3	1	0
0	0	1	0	1	0	1	3	1	0
0	0	1	0	0	0	0	3	1	0
0	0	1	0	1	0	0	3	1	0
0	0	1	0	1	0	0	3	1	0
1	0	0	0	0	0	0	3	1	0
0	0	0	0	0	0	0	3	1	0
0	0	1	0	0	0	0	3	1	0
0	0	1	0	0	0	0	3	1	0
0	0	0	0	0	0	0	3	1	0
0	0	1	0	0	0	0	3	1	0
0	0	1	0	0	0	0	3	1	0
0	0	0	1	0	0	0	3	1	0
0	0	0	0	0	0	0	3	1	0
0	0	0	0	0	0	0	3	1	0
0	0	1	0	0	0	0	3	1	0
0	0	1	0	0	0	1	3	1	0
0	0	0	0	0	0	0	3	1	0
0	0	0	0	0	0	0	3	1	0
0	0	0	0	0	0	0	3	1	0

0	0	1	1	1		0	3	1	1
0	0	0	0	0	0	0	3	1	0
0	0	1	0	1	0	0	3	1	1
0	0	1	0	0	0	0	3	1	0
0	0	0	1	1	0	1	3	1	0
0	0	0	0	0	0	0	3	1	0
0	0	0	0	0	0	0	3	1	0
0	0	1	0	1	0	0	3	1	0
0	0	1	0	1	0	1	3	1	0
0	0	0	0	0	0	1	3	1	0
0	0	0	0	0	0	1	3	1	0
0	0	0	0	0	0	0	3	1	0
0	0	1	0	0	0	0	3	1	0
0	0	0	0	0	0	0	3	1	0
0	0	1	0	0	0	0	3	1	0
0	0	1	0	0	0	1	3	1	0
0	0	0	0	0	0	0	3	1	0
0	0	1	0	1	0	0	3	1	0
0	0	0	0	0	0	0	3	1	0
0	0	1	1	1	1	0	3	1	0
0	0	0	0	0	0	0	3	1	0
0	0	1	1	1	1	1	3	1	0
0	0	0	0	0	0	0	3	1	0
0	0	1	0	0	0	1	3	1	0
0	0	1	0	0	0	1	3	1	0
0	0	0	0	0	0	1	3	1	0
0	0	0	0	0	0	0	3	1	0
0	0	0	0	0	0	0	3	1	0
0	0	0	0	0	0	0	3	1	0
0	0	0	0	0	0	0	3	1	0
0	0	0	0	0	0	0	3	1	0
0	0	1	0	0	0	0	3	1	0
0	0	0	0	0	0	0	3	1	0
0	0	1	1	1	0	1	3	1	0
0	0	1	1	1	0	1	3	1	0
1	1	0	0	0	0	0	3	1	0
0	0	1	0	0	0	0	3	1	0
0	0	0	0	0	0	1	3	1	0
0	0	0	0	0	0	1	3	1	0
0	0	0	0	0	0	0	3	1	0
0	0	0	0	0	0	0	3	1	0
0	0	0	0	0	0	0	3	1	0
0	0	1	0	0	0	0	3	1	0
0	0						3	1	0
0	0	0	1	1	0	1	3	1	0
0	0	0	0	1	0	0	3	1	0
0	0	0	0	0	1	1	3	1	0

0	0	0	0	1	0	0	3	1	0
0	0	1	1	1	0	0	3	1	0
0	0	0	0	0	0	0	3	1	0
0	0	1	0	0	0	0	3	1	0
0	0	1	0	0	0	0	3	1	0
0	0	1	0	1	0	1	3	1	0
0	0	0	1	0	0	0	3	1	0
0	0	1	0	0	0	0	3	1	0
0	0	1	0	1	0	0	3	1	0
0	0	0	0	0	0	0	3	1	0
0	0	0	0	1	0	0	3	1	0
0	0	1	0	1	0	0	3	1	0
0	0	1	0	1	0	0	3	1	0
0	0	0	0	0	0	0	3	1	0
0	0	1	0	1	0	0	3	1	0
0	0	1	0	0	0	0	3	1	0
1	0	1	0	0	0	0	3	1	0
0	0	0	1	1	0	0	3	1	0
1	0	0	1	0	0	0	3	1	1
0	0	0	0	0	0	0	3	1	1
0	0	1	1	1	0	0	3	1	0
0	0	1	1	1	0	0	3	1	0
0	0	0	1	1	0	1	3	1	0
0	0	1	0	1	0	1	3	1	0
0	0	1	0	1	0	1	3	1	0
0	0	1	1	1	0	1	3	1	0
0	0	1	0	1	0	0	3	1	0
1	1	1	0	1	0	0	3	1	0
0	0	0	0	0	0	0	3	1	0
0	0	0	0	1	0	0	3	1	0
0	0	1	0	0	0	1	3	1	0
0	0	1	1	0	0	0	3	1	0
0	0	1	0	0	0	0	3	1	0
1	0	0	0	0	0	0	3	1	0
0	0	0	0	0	0	0	3	1	0
0	0	0	0	0	0	0	3	1	0
0	0	0	0	0	0	0	3	1	0
0	0	1	0	0	0	0	3	1	0
0	0	1	0	0	0	0	3	1	0
0	0	0	0	0	0	0	3	1	0
0	0	0	0	0	0	0	3	1	0
0	0	1	0	0	0	1	3	1	0
0	0	1	1	1	1	1	3	1	0
1	0	0	0	1	1	1	3	1	0
1	0	1	0	0	0	0	3	1	0
0	0	0	0	0	1	0	3	1	0
0	0	1	0	0	0	0	3	1	0

0	0	1	0	0	0	0	3	1	0
0	0	0	0	0	0	0	3	1	0
1	0	1	0	0	1	0	3	1	0
1	0	1	1	1	0	0	3	1	0
0	0	0	0	0	0	0	3	1	0
0	0	1	1	1	1	1	3	1	0
0	0	1	1	1	1	1	3	1	0
0	0	0	0	0	0	0	3	1	0
0	0	0	0	0	0	0	3	1	0
0	0	0	0	0	0	0	3	1	1
0	0	0	0	0	0	1	3	1	0
0	0	0	1	1	0	1	3	1	0
0	0	0	0	0	0	0	3	1	0
0	0	1	0	0	0	0	3	1	0
0	1	0	0	0	0	0	3	1	0
0	0	0	0	0	0	0	3	1	0
0	0	1	0	1	0	0	3	1	0
0	0	0	1	0	0	0	3	1	0
0	0	0	0	0	0	0	3	1	0
0	0	1	0	0	0	0	3	1	0
0	0	1	0	0	0	0	3	1	0
0	0	0	0	0	0	0	3	1	0
0	0	1	0	0	0	0	3	1	0
0	0	1	0	0	0	0	3	1	0
0	1	1	1	1	0	0	3	1	0
0	0	1	0	0	0	0	3	1	0
0	0	0	0	0	0	0	3	1	0
0	0	1	0	1	0	0	3	1	0
0	0	0	0	0	0	0	3	1	0
0	0	0	0	0	0	0	3	1	0
0	0	1	0	1	0	0	3	1	0
0	0	1	0	0	0	1	3	1	0
1	0	0	0	0	0	0	3	1	0
0	0	0	0	0	0	0	3	1	0
0	0	0	0	0	0	0	3	1	0
1	0	0	0	0	0	1	3	1	0
1	0	0	0	0	0	1	3	1	0
0	0	0	0	0	0	0	3	1	0
0	0	0	0	0	0	0	3	1	0
0	0	1	0	0	0	0	3	1	0
0	0	0	0	0	0	0	3	1	0
0	0	1	0	0	0	0	3	1	0
0	0	1	0	0	0	0	3	1	0
0	0	0	0	0	0	1	3	1	0
0	0	0	0	0	0	1	3	1	0
0	0	0	0	0	0	0	3	1	0
0	0	0	0	0	0	0	3	1	0

0	0	0	0	0	0	0	3	1	0
0	0	0	0	0	0	0	3	1	0
0	0	0	0	1	0	0	3	1	0
0	0	0	0	0	0	0	3	1	0
0	0	1	0	1	0	0	3	1	0
0	0	0	0	0	0	0	3	1	0
0	0	1	0	0	0	0	3	1	0
0	0	1	0	1	0	0	3	1	0
0	0	0	0	0	0	0	3	1	0
0	0	0	0	0	0	0	3	1	0
0	0	0	0	0	0	0	3	1	0
0	0	0	0	0	0	0	3	1	0
0	1	1	1	1	0	0	3	1	0
0	0	1	0	1	0	0	3	1	0
0	0	0	0	0	0	0	3	1	0
0	0	0	0	0	0	0	3	1	0
0	0	0	0	0	0	0	3	1	0
0	0	0	1	0	0	1	3	1	0
0	0	0	0	0	0	1	3	1	0
0	0	0	0	0	0	0	3	1	0
0	0	0	0	0	0	0	3	1	0
0	0	0	0	0	0	1	3	1	0
0	0	0	0	0	0	0	3	1	0
1	0	0	0	0	0	0	3	1	1
0	0	0	0	0	0	0	3	1	0
0	0	0	0		0	0	3	1	0
0	0	0	0	0	0	0	3	1	0
0	0	0	0	0	0	0	3	1	0
0	0	1	0	1	0	0	3	1	0
0	0	0	0	1	0	0	3	1	0
0	0	0	0	0	0	0	3	1	0
0	0	1	0	0	0	0	3	1	0
0	0	0	0	0	0	0	3	1	0
0	0	0	0	0	0	1	3	1	0
0	0	1	0	0	0	0	3	1	0
0	0	1	1	1	0	0	3	1	0
0	0	1	0	1	0	0	3	1	0
0	0	0	1	1	0	0	3	1	0
0	0	0	0	0	0	0	3	1	0
0	0	1	0	1	0	0	3	1	0
0	0	1	0	0	0	0	3	1	0
0	0	0	0	1	0	0	3	1	0
0	0	0	1	1	0	1	3	1	0
0	0	0	0	0	0	0	3	1	0
0	0	1	1	0	0	0	3	1	0
0	0	1	0	0	0	0	3	1	0
0	0	1	1	0	0	0	3	1	0

0	0	1	1	0	0	1	3	1	0
0	0	1	0	1	0	0	3	1	0
0	0	0	0	0	0	0	3	1	0
0	0	0	0	0	0	1	3	1	1
0	0	0	0	0	0	0	3	1	0
0	0	0	0	0	0	0	3	1	0
0	0	0	0	0	0	0	3	1	0
0	0	1	0	1	0	0	3	1	0
0	0	0	0	1	0	0	3	1	0
0	0	0	0	1	0	0	3	1	0
0	0	1	0	1	0	1	3	1	0
0	0	1	0	1	0	0	3	1	0
0	0	1	0	0	0	0	3	1	0
0	0	0	0	0	0	0	3	1	0
0	0	1	0	1	0	0	3	1	0
0	0	1	0	0	0	0	3	1	0
0	0	1	1	1	1	1	3	1	0
0	0	0	0	0	0	0	3	1	0
0	0	0	0	0	0	0	3	1	0
0	0	1	1	1	1	0	3	1	0
0	0	1	0	1	1	1	3	1	0
0	0	0	0	0	0	0	3	1	0
0	0	1	0	0	0	0	3	1	0
0	0	1	0	0	0	0	3	1	0
0	0	0	0	0	0	0	3	1	0
0	0	0	0	0	0	0	3	1	0
0	0	1	1	1	1	1	3	1	0
0	0	0	0	0	0	0	3	1	0
0	0	1	0	1	0	0	3	1	0
0	0	1	0	0	0	0	3	1	0
0	0	0	0	1	0	0	3	1	0
0	0	1	1	1	0	1	3	1	0
0	0	1	0	0	0	0	3	1	0
0	0	0	0	0	0	0	3	1	0
0	0	0	0	0	0	0	3	1	0
0	0	1	1		0	1	3	1	0
0	0	1	0	1	0	0	3	1	0
0	0	1	0	1	0	0	3	1	0
0	0	0	0	0	0	0	3	1	0
0	0	1	0	0	0	0	3	1	0
0	0	1	0	1	0	0	3	1	0
1	0	1	1	1	0	0	3	1	0
0	0	0	0	0	0	0	3	1	0
0	0	0	0	0	0	0	3	1	0
0	0	1	0	1	0	0	3	1	0
0	0	1	0	1	0	0	3	1	0
0	0	1	0	1	0	0	3	1	0

0	0	1	0	1	0	0	3	1	0
0	0	1	0	1	1	0	3	1	0
0	0	1	0	1	0	0	3	1	0
0	0	0	0	0	0	0	3	1	0
0	0	1	0	0	0	0	3	1	1
0	0	1	0	1	1	0	3	1	0
0	0	1	0	1	0	0	3	1	0
0	0	1	0	1	0	0	3	1	0
0	0	1	0	0	0	0	3	1	0
0	0	1	0	0	0	0	3	1	0
0	0	1	0	0	0	0	3	1	0
0	0	0	0	0	0	0	3	1	0
0	0	0	0	0	0	0	3	1	0
0	0	1	0	0	0	0	3	1	0
0	0	0	0	0	0	0	3	1	0
0	0	1	0	0	0	0	3	1	0
0	0	1	0	0	0	0	3	1	1
0	0	0	0	0	0	0	3	1	0
0	0	0	0	0	0	0	3	1	0
1	1	0	0	0	0	0	3	1	0
0	0	1	0	0	0	0	3	1	0
0	0	1	1	1	1	0	3	1	0
0	0	0	0	0	0	0	3	1	0
0	0	0	0	0	0	0	3	1	0
0	0	0	0	0	0	0	3	1	0
0	0	0	0	0	0	0	3	1	0
0	0	1	0	0	0	0	3	1	0
1	0	0	0	0	0	0	3	1	0
0	0	1	0	0	0	0	3	1	0
0	0	0	0	0	0	0	3	1	0
0	0	0	0	0	0	0	3	1	1
0	0	0	0	0	0	0	3	1	0
0	0	1	0	0	0	0	3	1	0
0	0	0	0	0	0	0	3	1	0
0	0	0	0	0	0	0	3	1	0
0	0	0	0	0	0	0	3	1	0
0	1	1	1	1	1	1	3	1	0
0	0	0	0	0	0	0	3	1	0
0	0	0	0	0	0	0	3	1	0
0	0	0	0	0	0	0	3	1	0
0	0	0	0	0	0	0	3	1	0
0	0	0	0	0	0	0	3	1	0
0	0	0	0	0	0	0	3	1	0
0	0	1	0	0	0	1	3	1	0
0	0	1	0	0	0	1	3	1	0
0	0	1	1	1	1	0	3	1	0
0	0	1	0	0	0	0	3	1	0

0	0	1	0	1	0	0	3	1	0
0	0	0	0	0	0	0	3	1	1
0	0	1	0	1	0	0	3	1	0
0	0	0	0	0	0	0	3	1	0
1	0	1	1	1	1	1	3	1	0
0	0	0	0	0	0	0	3	1	0
0	0	0	0	1	0	1	3	1	0
0	0	1	0	0	0	0	3	1	0
0	0	0	0	0	0	0	3	1	0
0	0	0	0	0	0	0	3	1	0
0	0	1	0	1	0	0	3	1	0
0	0	0	0	0	0	0	3	1	0
0	0	0	0	0	0	0	3	1	0
0	0	0	0	0	0	0	3	1	0
1	0	0	0	0	0	0	3	1	1
0	0	1	0	1	0	0	3	1	1
0	0	1	0	0	0	0	3	1	0
0	0	1	0	0	0	0	3	1	0
1	1	1	0	1	0	0	3	1	1
0	0	1	0	1	0	0	3	1	0
0	0	0	0	0	0	0	3	1	0
0	0	1	1	1	1	1	3	1	0
0	0	0	0	0	0	0	3	1	1
0	0	1	1	1	1	0	3	1	0
0	0	1	0	1	0	0	3	1	0
0	0	1	0	1	0	1	3	1	0
0	0	1	0	1	0	1	3	1	0
0	0	0	0	1	0	0	3	1	0
0	0	1	0	0	0	0	3	1	0
0	0	0	0	0	0	0	3	1	0
0	0	0	0	0	0	0	3	1	0
0	0	0	0	0	0	1	3	1	0
0	0	1	0	0	0	0	3	1	0
0	0	1	0	0	0	0	3	1	0
0	0	1	0	0	0	0	3	1	0
0	0	1	0	1	0	0	3	1	0
0	0	1	0	1	0	1	3	1	0
0	0	0	0	1	0	0	3	1	0
0	0	0	0	0	0	0	3	1	0
0	0	0	0	0	0	1	3	1	0
0	0	1	0	1	0	1	3	1	0
0	0	0	0	0	0	0	3	1	0
0	0	0	0	0	0	1	3	1	0
0	0	1	0	0	0	1	3	1	0
0	0	1	0	0	0	1	3	1	0
0	0	1	0	1	0	0	3	1	0
0	0	0	0	0	0	0	3	1	0

0	0	0	0	0	0	0	3	1	0
0	0	1	0	0	0	1	3	1	0
0	0	1	0	1	1	1	3	1	0
0	0	1	1	1	1	1	3	1	0
0	0	0	0	0	0	0	3	1	0
0	0	1	0	1	0	1	3	1	0
0	0	0	0	1	0	0	3	1	0
0	0	1	0	0	0	0	3	1	0
0	0	0	0	0	0	0	3	1	1
0	0	0	0	0	0	0	3	1	0
0	0	0	0	0	0	0	3	1	0
0	0	0	0	0	0	0	3	1	0
0	0	1	1	1	0	1	3	1	0
0	0	0	0	1	0	0	3	1	0
0	0	0	0	0	0	0	3	1	0
0	0	0	0	0	0	0	3	1	0
0	0	0	0	0	0	0	3	1	0
0	0	1	0	0	0	1	3	1	0
0	0	1	0	1	0	0	3	1	0
0	0	0	0	0	0	0	3	1	0
0	0	1	0	1	0	0	3	1	0
0	0	0	0	0	0	0	3	1	0
0	0	1	1	1	0	0	3	1	0
0	0	1	0	0	0	0	3	1	0
0	0	1	0	0	0	0	3	1	0
0	0	0	0	0	0	1	3	1	0
0	0	0	0	0	0	0	3	1	0
1	0	1	0	1	0	0	3	1	0
0	0	1	0	0	0	1	3	1	0
0	0	0	0	0	0	0	3	1	0
0	0	0	0	0	0	1	3	1	0
0	0	0	0	0	0	1	3	1	0
0	0	0	0	0	0	1	3	1	0
0	0	0	0	0	0	0	3	1	0
0	0	0	0	0	0	0	3	1	1
0	0	0	1	0	0	0	3	1	0
0	0	0	0	0	0	0		1	0
0	0	0	0	0	0	0		1	0
0	0	0	0	0	0	0		1	0
0	0	0	0	1	0	0		1	0

cesd21	ecipv12	ipv12	lifetrauma	childtraum	moneyfnd	educlevel	helpsco	foodrc1	incomerc1
0	0	0	0	14	1	2	0	2	0
0	0	0	0	14	2	2	0	2	0
0	0	0	0	14	1	2	1	2	0
0	0	0	0	19	1	2	0	1	0
0	0	0	0		1	1	0	2	1
0	0	0	0	14	1	2	1	2	1
0	0	0	0	15	1	1	0	1	0
0	0	0	0	14	1	2	1	2	0
0	0	0	0	15	1	2	1	2	0
0	0	0	0		1	1		1	0
0	0	0	1	28	1	2	1	1	0
1	2	1	3	19	1	2	0	1	0
0	0	0	0	16	1	2	0	2	0
0	0	0	0	16	1	1	1	2	1
0	2	1	0	16	1	2	2	1	1
0	0	0	0		1	2	2	2	1
0	0	0	0	16	1	2	2	2	1
0	0	0	3	17	2	2	0	2	0
1	1	1	2	27	1	1	1	2	0
0	0	0	0	17	1	1	1	2	1
0	2	1	0	25	1	1	0	2	0
1	2	1	2	19	1	2	1	1	0
0	0	0	2	17	2	2	2	2	0
0	0	0	0	21	2	1	5	1	0
0	1	1	2	24	1	2	4	1	1
0	0	0	0	15	1	1	2	1	0
1	0	0	0	18	1	2	1	2	0
1	2	1	1	28	1	2	3	2	0
1	0	0	3	23	1	1	1	1	0
1	2	1	3	22	2	1	0	2	1
0	0	0	0	15	2	1	0	2	0
0	0	0	0	15	2	2		2	0
0	0	0	1	23	1	1	0	2	0
0	0	0	5	17	1	2	1	2	0
0	0	0	0	18	1	2		2	0
0	0	0	1	21	2	2	0	2	0
0	1	1		20	2	1	4	2	1
0	0	0	2	18	1	2	0	2	0
0	0	0	1	25	1	2	0	1	0
0	0	0	1	24	1	2	1	2	0
0	0	0	0	15	1	2	0	2	0
0	0	0	1	17	2	2	1	2	0
0	0	0	1		1	2	1	2	0
1	0	0	0	20	1	1	1	1	0
0	0	0	1		1	2	2	2	0
0	0	0	1		2	2	0	2	1

0	0	0	3	22	2	2	1	2	0
0	0	0	3	17	1	2	0	2	1
0	0	0	0	16	1	2	1	2	0
0	0	0	0	22	1	2	0	2	0
0	0	0	0	14	2	2	0	2	0
0	0	0	0		1	2	0	1	0
1	0	0	0	22	1	2	0	2	0
1	0	0	0	22	1	2	0	2	0
1	0	0	2	16	1	2	0	2	1
0	2	1	2	34	1	1	0	2	1
0	0	0	1	14	2	2	1	2	0
0	0	0	0	15	2	2	0	1	0
0	0	0	0	18	1	2	1	2	0
1	2	1	1	17	1	2	0	2	1
0	0	0	0	17	2	2	0	2	0
0	2	1	3	20	2	2	1	2	0
0	0	0	0	16	1	2	0	2	0
0	0	0	0	16	2	2	0	2	1
0	0	0	0	20	1	2	4	1	0
1	0	0	1	18	1	2	1	2	0
0	0	0	1	16	2	2	1	2	0
0	0	0	0	16	2	1	0	2	1
0	0	0	0	14	1	2	0	2	0
0	0	0	0	18	1	2	0	2	0
0	2	1	0	17	1	2	0	2	0
0	0	0	0	14	2	2	0	2	0
0	0	0	0	15	1	2	0	1	0
0	0	0	1	31	2	1	1	2	0
0	0	0	0	21	1	2	0	1	1
0	0	0	0	21	1	1	3	2	0
0	0	0	0	32	1	2	2	1	0
0	0	0	0	20	1	2	4	2	0
0	0	0	0	31	2	1	1	2	0
0	0	0	0	43	2	1	1	2	0
0	2	1	0	22	1	1	4	1	0
0	0	0	0	14	2	2	0	2	0
0	0	0	0	14	1	2	0	1	1
1	0	0	0	16	2	1	1	2	0
1	0	0	0	14	1	1	0	2	0
0	0	0	0	14	2	1	1	2	0
0	0	0	0	14	1	1	0	1	0
0	0	0	0	14	1	2	0	1	0
0	0	0	2	23	1	2	3	1	1
0	1	1	0	18	1	2	4	2	0
0	0	0	0	18	1	2	0	1	0
0	2	1	1	14	2	1	1	2	0
0	0	0	0	23	1	2	4	1	0

0	2	1	0	41	2	1	1	2	0
0	0	0	0	14	1	2	1	2	1
0	1	1	1	17	1	2	1	2	1
0	2	1	2	28	1	1	1	1	0
1	2	1	2	17	1	1	1	2	1
0	0	0	2	19	1	1	1	1	0
1	0	0	2	21	1	1	1	1	1
0	2	1	2	20	1	1	1	2	0
1	2	1	1	23	1	2	1	2	0
0	0	0	0	22	1	2	1	2	1
0	2	1	1	26	1	1	1	2	0
0	2	1	0	18	1	1	0	2	1
1	2	1	0	22	1	1	1	2	1
0	0	0	0	27	1	1	0	1	0
1	2	1	0	21	1	1	1	1	0
0	0	0	0	20	2	2	0	2	0
0	2	1	1	27	1	1	0	1	0
1	2	1	0	27	1	1	1	1	1
1	2	1	1	22	1	1	1	1	0
0	1	1	0	14	1	1	0	1	0
0	2	1	1	17	2	2	0	2	0
0	2	1	0	27	1	1	1	1	0
0	2	1	0	24	1	2	0	2	0
0	0	0	0	19	2	1	1	2	0
0	2	1	0	18	1	1	1	1	0
1	0	0	0	21	1	1	0	1	1
0	0	0	0	26	1	2	0	2	1
0	1	1	1	20	1	1	0	1	0
0	1	1	1	20	1	1	0	1	0
0	0	0	0	15	1	2	0	1	0
0	1	1	0	18	2	2	0	2	1
0	0	0	0	17	2	1	0	1	0
0	2	1	1	23	1	2	0	2	0
0	0	0	0	15	1	2	1	1	0
0	0	0	0		1	1	0	2	1
0	2	1	0	17	1	1	0	2	0
0	0	0	0	14	2	1	0	2	1
0	0	0	0	15	1	1	0	2	0
0	0	0	0	14	2	1	0	2	0
0	1	1	0	34	2	1	0	2	0
1	0	0	0	18	1	1	1	1	0
0	2	1	0	23	1	1	1	1	0
0	2	1	0	22	2	1	0	2	0
0	0	0	0	17	1	1	0	2	0
0	0	0	0	14	1	2	0	1	0
1	0	0	0	21	1	1	1	1	0
1	2	1	0	24	1	1	3	1	1

0	2	1	2	18	1	1	2	1	0
0	0	0	3	18	1	1	2	1	0
0	0	0	0	18	1	2	1	2	0
0	0	0	0	20	1	1	1	1	0
1	0	0	2	23	2	1	3	1	1
0	0	0	1	17	1	1	2	2	0
0	2	1	0	21	1	1	2	2	1
0	0	0	0	24	2	1	1	2	0
0	2	1	0	14	2	2	2	2	0
0	2	1	0	14	2	2	2	2	0
0	2	1	0	20	1	2	2	1	0
0	2	1	0	20	1	1	0	1	0
0	0	0	0	24	1	2	2	2	0
0	0	0	0	18	1	2	0	2	0
0	0	0	1	20	1	1	0	1	1
0	0	0	2	14	1	1	2	2	0
0	0	0	1	14	1	2	2	2	0
0	0	0	0	25	2	1	2	2	0
0	2	1	0	15	1	1	2	1	0
0	2	1	0	15	1	1	2	1	0
0	2	1	0	18	1	2	1	1	0
0	2	1	0	18	1	2	2	1	0
0	0	0	1	21	2	2	2	2	0
1	2	1	0	26	1	1	2	2	0
0	1	1	1	18	1	1	0	1	0
0	0	0	0	16	2	2	0	2	0
0	0	0	0	17	1	2	1	1	0
0	0	0	1	20	2	2	1	2	0
0	0	0	0	17	2	2	0	2	0
0	1	1	1	21	1	2	1	2	0
0	0	0	0	16	1	2	0	1	0
0	0	0	0	19	1	2	1	2	1
0	0	0	0	16	2	2	0	2	0
0	0	0	0	15	2	2	0	2	0
0	0	0	0	14	1	1	0	1	0
0	1	1	1	20	1	1	1	1	0
0	0	0		16	1	2	0	2	0
1	2	1	0	32	1	2	1	2	0
0	0	0	1	19	1	2	1	2	0
1	2	1	0	16	1	1	1	1	1
0	0	0	0	15	1	2	0	2	1
0	2	1	0	25	2	2	1	2	0
1	2	1	0	15	2	2	1	2	0
0	0	0	0	19	1	1	0	2	0
0	0	0	0	20	1	1	1	1	0
0	2	1	2	17	2	1	1	2	1
0	0	0	0	15	1	1	0	2	0

0	0	0	0	15	2	1	2	2	0
0	0	0	0	18	2	2	1	2	0
0	0	0	0	22	1	2	1	2	0
0	2	1	0	22	1	2	1	1	1
0	0	0	0	20	1	2	0	1	0
0	0	0	1	16	2	2	1	2	0
0	1	1	0	21	1	2	1	1	0
0	0	0	0	18	1	2	0	2	1
0	0	0	1	23	2	2	1	2	0
0	1	1	1	14	2	2	2	2	0
0	0	0	2	17	1	2	2	1	0
0	0	0	0	15	2	2	1	2	0
0	0	0	0	21	1	2	3	1	0
0	0	0	1	19	1	1	1	2	0
0	0	0	0	14	2	2	1	2	0
0	1	1	0	18	1	1	3	1	0
0	0	0	0	18	1	2	1	2	0
0	0	0	0	15	2	2	0	1	0
0	0	0	0	19	1	1	0	2	1
0	2	1	0	15	1	1	0	2	0
0	0	0	0	20	1	1	0	1	0
0	0	0	0	18	1	1	0	1	0
0	0	0	0	20	1	1	0	1	0
0	0	0	0	17	1	1	0	2	0
0	1	1	0	18	1	1	3	1	0
0	0	0	0	18	1	1	0	2	0
0	0	0	0	14	1	1	0	2	0
0	0	0	0	14	1	2	4	2	0
0	0	0	0	16	1	1	0	1	0
0	0	0	0	18	1	1	0	1	0
1	2	1	1	21	1	1	0	2	0
0	0	0	0	17	1	1	0	2	0
0	0	0	0	17	1	1	0	2	0
0	0	0	0	17	1	2	0	2	0
0	1	1	1	23	1	2	1	2	0
1	2	1	3	29	1	1	2	2	0
0	0	0	2	21	1	2	1	1	0
0	0	0	3	22	1	2	1	2	0
0	0	0	0	20	1	1	0	2	1
0	1	1	0	15	2	2	1	2	0
0	1	1	0	14	1	2	1	2	0
0	2	1	0	39	1	3	4	2	0
0	1	1	1	27	1	2	1	1	1
1	1	1	4	25	1	1	1	2	1
0	0	0	0	20	1	1	0	2	0
0	0	0	0	20	1	1	1	1	1
0	0	0	0	21	1	1		2	0

0	0	0	1	17	1	1	0	2	0
0	1	1	1	22	1	2	2	2	0
0	0	0	1	17	1	2	1	2	0
0	0	0	0	14	2	2	2	2	0
0	0	0	0	14	2	2	0	2	0
0	0	0	0	16	1	2	2	2	0
0	0	0		19	1	2	1	2	1
0	0	0		19	2	1	1	2	1
0	2	1	0	22	1	2	1	2	0
0	2	1	0	16	1	1	1	2	0
0	2	1	0	21	1	1	0	2	0
0	0	0	0	18	1	1	3	2	0
0	1	1	0	14	1	2	0	2	0
1	1	1	0	21	1	1	2	2	0
0	0	0		14	1	1	0	2	0
0	0	0		17	1	2	0	2	0
0	0	0	1	15	1	1	0	1	0
0	0	0		16	1	2	0	2	0
0	0	0		16	1	2	0	2	0
0	0	0	0	15	1	2	1	1	0
0	0	0	0	14	1	2	2	1	0
1	0	0		19	1	2	2	2	0
0	2	1		25	1	2	0	2	1
1	1	1	3	35	1	1	2	2	0
1	2	1	1	26	1	1	0	1	0
0	0	0		16	2	2	0	2	0
0	0	0		33	1	1	0	2	1
0	1	1	0	16	2	2	0	2	0
0	0	0	1	20	1	1	0	1	0
0	0	0	2	20	1	1	2	2	0
0	0	0	2	16	1	2	2	2	0
0	0	0	1	20	2	2	1	2	1
0	0	0	1	20	2	2	1	2	1
0	2	1	0	17	1	2	1	2	1
1	0	0			1	1		2	0
0	0	0	1	16	2	1	0	1	0
0	0	0	0	19	2	2	1	1	0
0	0	0	0	18	2	2	0	2	0
0	2	1	1	21	2	2	1	2	0
0	0	0	1	29	1	1	2	1	0
0	0	0	0	16	1	1	1	2	0
0	0	0	0	15	1	1	1	1	0
0	0	0	0	15	1	2	1	2	0
0	0	0	0	17	2	2	1	2	0
0	0	0	0	14	1	2	1	2	0
0	0	0	0	16	1	2	1	2	0
0	0	0	1		2	3	1	2	0

0	0	0	0		2	2	0	2	1
0	0	0	0	19	1	2	3	2	0
0	0	0	0	14	1	2	1	2	0
0	0	0	2	16	1	2	1	1	0
0	0	0	0	14	1	2	1	2	0
0	0	0	0	17	1	2	3	2	0
0	0	0	3	19	1	2	1	2	1
0	0	0	0	14	2	3	1	2	1
0	0	0	0	22	1	3	0	2	0
0	1	1	2	26	2	2	2	1	1
0	0	0	0	14	1	2	1	2	0
1	0	0	2	16	1	2	1	2	1
0	0	0	0	16	1	1	1	2	0
0	0	0	0	16	2	1	4	2	0
0	0	0	0	16	2	1	4	2	0
1	2	1	2	20	1	2	0	1	0
1	1	1	3	30	1	2	1	2	0
0	0	0	0	14	2	2	4	1	0
0	0	0	0	18	1	2	1	2	1
0	0	0	1	21	2	2	0	2	0
0	0	0	0	21	1	2	0	2	1
1	2	1	1	18	1	2	4	2	0
0	1	1	0	20	1	2	1	1	1
0	0	0	0	16	1	1	1	2	0
0	0	0	1	19	1	2	1	2	0
1	1	1	2	20	2	2	0	2	0
0	0	0	0	15	1	2	1	2	0
0	0	0	0	19	1	2	0	1	0
0	0	0	0		1	2	2	2	0
0	1	1	1	29	1	2	1	2	1
0	0	0	1	25	1	2	1	2	0
0	1	1	2	18	1	2	2	2	0
0	0	0	0	20	1	2	0	2	0
0	0	0	1	26	1	2	2	2	0
0	0	0	1	23	1	2	1	1	0
1	2	1	1	18	1	2	3	2	1
0	0	0	1	17	1	2	1	1	0
0	0	0	0	18	1	2	1	1	0
1	0	0	0	21	1	2	0	2	1
1	0	0	1		1	2	0	2	0
0	0	0	1	20	2	2	0	2	0
0	0	0	0	24	1	2	0	2	0
0	0	0	1	14	2	2	1	2	0
0	2	1	0	20	2	2	2	2	1
0	0	0	0	26	2	2	2	2	0
0	0	0	0	18	1	2	1	2	0
0	1	1	0	19	1	2	1	1	0

0	1	1	0	19	1	2	1	1	0
0	0	0	1	16	1	2	0	2	0
0	2	1	0	26	2	2	1	1	0
1	0	0	3	18	1	2	1	2	1
1	0	0	1	23	2	2	2	2	0
0	0	0	0	26	2	2	2	2	1
0	1	1	0	16	1	2	2	2	1
0	1	1	0	16	1	2	2	2	1
1	0	0	0	16	1	3	3	1	0
0	0	0	0	19	1	2	2	1	0
0	1	1	0	22	1	2	1	1	0
0	0	0	0	30	1	2	0	1	0
1	1	1	0	28	1	2	1	2	0
1	0	0	1	22	1	2	2	2	0
0	1	1	0	18	1	2	3	2	0
0	0	0	1	22	1	2	3	2	0
0	2	1	2	18	2	3	1	2	0
0	0	0	1	19	1	2	0	2	0
1	1	1	0	30	1	2	1	2	0
0	0	0	0	24	1	2	2	2	0
0	0	0	2	14	2	3	0	2	0
0	2	1	0	16	1	2	0	1	0
0	0	0	2	19	2	1	1	2	0
0	0	0	1	25	1	1	0	2	0
0	0	0	2	20	1	2	2	2	0
0	0	0	0	19	1	2	2	2	0
0	0	0	3	18	1	3	1	2	0
0	0	0	1	16	2	3	0	2	0
1	2	1	1	21	1	2	0	2	0
0	1	1	0	19	2	2	1	2	0
1	0	0	3	18	2	2	2	2	0
0	0	0	0	20	1	1	1	2	1
0	0	0	0	14	2	2	0	2	0
0	0	0	1	17	1	2	3	2	0
0	2	1	0	22	1	2	2	2	1
0	2	1	2	23	2	2	2	2	0
0	2	1	1	17	1	2	0	2	0
0	0	0	1	14	2	2	0	2	0
0	0	0	0	14	1	2	3	2	0
0	0	0	0	15	1	2	5	2	1
0	0	0	0	21	1	2	2	2	0
0	0	0	0	14	2	2	0	2	0
0	0	0	0	26	1	1	5	1	0
0	1	1	0	24	1	1	1	1	0
0	0	0	2	24	1	2	1	1	0
0	0	0	0	20	1	1	1	1	0
0	0	0	2	14	2	2	0	2	0

0	0	0	0	16	1	2	0	2	1
0	0	0	0	14	2	2	5	1	0
0	0	0	0	15	2	2	5	1	0
0	0	0	0	14	1	1	0	2	0
0	0	0	0	19	1	1	1	1	0
0	0	0	0	14	1	2	0	1	0
0	0	0	0	14	2	2	0	2	0
0	0	0	0	14	2	2	0	2	0
0	1	1	0	21	1	2	0	2	0
1	0	0	5	29	1	2	0	1	0
1	1	1	0	29	1	2	0	1	0
0	0	0	0	15	2	2	5	2	0
0	1	1	0	24	1	1	4	1	0
0	2	1	1	24	1	2	0	2	0
0	2	1	2	22	1	2	0	1	0
1	1	1	0		1	1	1	1	0
0	0	0	0	20	1	1	2	2	0
0	0	0	0	14	2	1	2	2	0
0	2	1	0	25	1	2	0	1	0
0	0	0	0	18	1	2	4	2	1
0	1	1	1	22	1	1	1	1	0
0	1	1	0	29	1	1	0	1	0
0	1	1	0	23	1	1	1	1	0
0	1	1	0	20	1	1	1	1	1
0	0	0	0	26	1	1	1	2	0
0	0	0	0	22	1	2	5	1	0
1	1	1	1	26	1	1	0	2	1
0	1	1	1	21	1	1	0	1	0
0	0	0	0	17	1	1	2	1	0
0	2	1	2	21	2	2	2	2	1
0	1	1	1	29	1	1	4	2	0
0	0	0	0	14	1	1	3	2	0
0	0	0	0	18	2	2	0	1	0
0	0	0	0	18	1	1	1	1	0
0	0	0	0	14	2	2	0	2	0
0	2	1	0	19	2	2	2	1	1
0	1	1	0	16	1	1	0	1	0
0	0	0	0	15	1	1	2	1	0
0	0	0	0	16	2	2	1	2	0
0	2	1	0	20	1	2	0	2	0
0	0	0	0	18	1	1	0	1	0
0	1	1	0	16	1	2	0	1	1
0	0	0	0	26	1	1	1	1	0
0	0	0	0	17	2	2	4	2	0
0	0	0	0	21	2	1	1	2	0
0	0	0	0	16	2	2	1	2	1
0	0	0	1	20	1	2	3	1	0

0	0	0	0	17	1	2	1	2	0
0	0	0	3	16	1	1	0	1	0
0	1	1	0	17	1	1	1	1	0
0	1	1	1	18	1	1	3	1	0
0	2	1	0	16	1	1	2	1	0
0	0	0	0	21	1	1	0	1	0
0	0	0	0	28	1	1	0	1	0
0	0	0	0	19	1	1	1	1	0
0	0	0	0	19	1	2	0	2	0
0	0	0	0	15	1	2	2	2	0
0	0	0	0	17	2	1	0	1	0
1	1	1	0	14	1	1	2	1	0
0	0	0	1	17	1	1	2	1	0
0	0	0	0	22	1	1	0	1	0
0	0	0	0	19	1	2	1	1	1
0	0	0	0	14	2	2	0	2	0
1	1	1	1	21	1	1	3	1	0
0	2	1	0	18	1	2	2	2	1
1	2	1	0	15	2	2	3	2	0
0	0	0	0	17	2	2	2	2	0
0	1	1	1	17	1	2	0	1	0
0	0	0	3	22	1	2	3	2	1
0	2	1	1	22	1	2	2	2	0
0	0	0	0	22	2	2	1	1	1
0	0	0	0	16	1	1	2	2	1
0	0	0	0	18	1	1	0	2	0
1	2	1	7	28	2	2	3	2	1
0	0	0	1	19	1	2	2	1	0
0	2	1	0	16	2	3	4	2	0
0	0	0	0	18	1	2	3	2	0
0	1	1	3	21	1	2	1	1	0
0	0	0	0	16	1	2	0	1	1
0	0	0	0	16	1	2	0	1	1
0	0	0	1	20	2	1	1	2	0
0	1	1	0	16	1	1	1	2	0
1	1	1	3	21	1	1	1	1	0
1	0	0	1	21	1	1	5	1	1
0	1	1	0	15	1	1	1	1	0
0	0	0	0	20	1	2	1	2	0
0	2	1	0	18	2	2	2	2	1
0	0	0	1	16	1	2	1	2	0
0	2	1	2	29	1	1	1	2	0
1	1	1	0	16	2	1	0	2	0
0	2	1	1	18	2	2	3	2	1
1	0	0	0	18	2	3	2	2	0
0	0	0	0	18	2	3	2	2	1
0	0	0	1	22	1	1	2	1	0

0	2	1	0	21	1	2	1	1	0
0	0	0	0	17	2	1	0	2	0
0	0	0	3	15	1	3	1	2	0
0	2	1	1	23	2	1	2	1	0
0	2	1	2	15	1	1	0	1	0
0	0	0	1	17	2	2	2	2	0
0	1	1	2	20	2	2	1	2	0
0	0	0	1	20	1	2	1	2	0
0	2	1	0	18	1	2	1	2	0
0	0	0	1	21	1	1	3	2	0
1	0	0	0	20	1	1	1	2	1
0	2	1	0	24	1	1	2	1	0
0	0	0	2	19	1	2	2	2	1
0	0	0	0	16	1	2	0	1	0
0	0	0	1	19	1	1	2	1	1
0	0	0	0	18	1	2	0	2	0
0	1	1	1	22	2	2	1	2	0
0	0	0	0	18	1	1	0	2	0
0	2	1	3	18	1	2	3	1	0
0	1	1	0	19	1	2	1	2	0
0	2	1	1	18	1	2	2	2	0
0	0	0	1	23	1	2	1	2	0
0	0	0	1	21	1	2	1	2	1
0	0	0	0	18	1	3	0	2	0
0	0	0	0	17	1	1	0	1	1
1	2	1	0	18	1	2	2	1	0
0	0	0	0	16	2	2	0	2	0
0	2	1	1	20	2	2	1	2	0
0	2	1	2	21	2	2	1	2	0
0	2	1	1	22	1	2	1	2	0
0	1	1	2	20	2	2	1	2	1
0	0	0	0	17	2	2	0	2	0
0	0	0	0	22	1	2	0	2	0
0	0	0	2	23	1	2	0	1	0
0	1	1	1	19	2	2	2	2	0
0	0	0	1	16	2	2	1	2	0
0	0	0	0	18	2	2	0	2	0
0	0	0	1	21	1	1	0	1	0
0	1	1	0	19	1	2	2	2	0
0	2	1	0	16	2	1	2	1	1
0	2	1	0	17	1	1	0	1	0
0	1	1	0	24	1	1	0	2	0
0	0	0	0	19	1	3	1	2	0
0	2	1	2	21	1	2	2	2	0
0	0	0	1	19	1	1	1	1	0
0	0	0	1	15	1	1	1	1	0
0	0	0	0	17	1	1	0	1	0

0	0	0	0	15	1	1	0	1	0
0	0	0	0	18	1	1	1	1	0
1	1	1	0	18	1	1	0	1	0
0	0	0	0	15	1	3	3	2	1
0	0	0	0	20	1	1	0	1	0
0	0	0	0	20	1	1	0	1	0
1	1	1	1	14	1	1	3	2	0
0	2	1	1	18	1	1	4	2	0
0	0	0	0	18	1	1	0	1	0
0	1	1	0	14	1	2	1	1	0
0	0	0	0	16	1	1	0	1	0
0	2	1	0	17	1	1	0	2	0
0	0	0	1	15	1	1	3	2	0
0	0	0	0	15	1	1	0	2	0
0	0	0	0	17	2	2	0	2	0
0	0	0	0	15	1	1	1	1	0
0	2	1	1	15	1	1	3	1	0
0	0	0	0	17	1	1	0	2	0
0	0	0	0	21	1	1	2	1	0
0	0	0	0	14	1	2	0	1	0
0	0	0	0	15	1	1	0	1	0
0	0	0	1	15	1	1	1	1	0
0	0	0	0	15	1	1	0	1	0
0	2	1	1	19	1	2	1	2	1
0	1	1	4	20	1	2	2	1	0
0	0	0	2	19	1	1	2	2	1
0	0	0	0	19	1	2	2	2	0
0	2	1	0	15	1	2	1	2	0
0	2	1	4	28	1	2	4	2	0
0	0	0	2	20	1	2	3	2	1
0	0	0	1	16	1	2	1	1	0
0	0	0	1	22	1	2	0	1	0
0	0	0	0	16	2	3	1	2	0
1	0	0	4	27	1	2	2	2	1
1	1	1	5	28	1	1	2	1	0
0	0	0	0	16	1	3	0	2	1
1	1	1	4	20	1	1	1	2	0
0	0	0	0	15	1	2	0	2	0
0	2	1	3	25	1	2	1	2	1
0	0	0	1	31	1	1	2	2	1
0	1	1	1	18	1	2	1	2	0
1	1	1	1	25	1	1	2	2	0
0	0	0	0	17	1	1	1	2	0
0	0	0	2	24	1	2	1	2	1
0	1	1	0	17	1	2	2	2	1
0	0	0	3	23	1	2	3	2	0
1	0	0	3	24	1	2	0	2	1

0	0	0	1	24	1	1	2	1	0
0	0	0	0	18	1	1	0	2	0
0	0	0	0	18	1	1	2	2	0
0	2	1	1	22	1	2	0	2	0
0	0	0	0	15	2	2	2	2	1
0	0	0	2	15	1	2	5	2	1
0	1	1	0	17	1	2	1	2	0
1	0	0	0	16	1	1	2	1	0
0	0	0	0	20	1	1	1	1	0
0	0	0	0	21	1	1	2	2	0
0	0	0	1	15	1	2	2	1	0
0	0	0		20	1	1	0	1	0
0	2	1	0	19	2	2	0	2	0
0	0	0		20	1	2	4	2	0
0	0	0	0	15	1	1	2	2	0
1	1	1	1	19	2	2	1	2	0
1	0	0	0	18	1	1	0	1	0
0	0	0		14	1	2	0	1	0
0	0	0		19	1	1	0	1	0
0	2	1	3	17	2	2	2	2	0
0	0	0	5	26	1	2	0	2	0
0	0	0	0	16	2	2	4	2	1
0	0	0		14	1	2	1	2	0
0	2	1	0	16	1	1	3	1	0
0	0	0	0	16	1	2	1	2	0
0	2	1	1	16	2	2	3	2	0
1	2	1		20	1	3	1	2	0
1	0	0		17	1	2	1	2	0
0	0	0	0	16	1	1	0	2	0
0	0	0	0	14	1	2	4	2	0
0	2	1	0	27	1	1	0	1	0
0	0	0	0	20	1	1	0	2	0
0	1	1	0	14	1	2	1	2	0
0	0	0		16	1	1	0	2	0
0	0	0	0	15	1	2	3	1	0
0	0	0	0	14	2	2	1	1	0
0	0	0		18	1	1	0	2	0
0	0	0	1	19	1	1	1	2	0
0	1	1	0	18	1	2	1	2	0
0	0	0	0	20	1	1	4	2	0
0	0	0	0	15	2	2	1	2	0
0	0	0	1	16	1	2	1	1	0
1	2	1	3	20	1	2	0	2	0
0	0	0	0	20	1	2	0	1	0
0	0	0		16	1	2	1	2	0
0	0	0		14	2	2	1	2	0
0	1	1	1	21	1	1	0	2	0

0	0	0	3	18	1	1	4	1	1
0	0	0	0	17	1	2	1	1	0
0	0	0	0	17	1	2	1	1	0
0	2	1		19	1	2	0	2	0
0	0	0	0	15	1	1	0	2	0
0	0	0	0	14	1	2	2	2	0
0	0	0	0	21	1	1	1	2	0
1	1	1		18	1	2	1	1	0
1	0	0		18	2	1	1	2	0
0	2	1		24	1	2	2	2	0
0	0	0	2	19	2	2	1	2	0
0	0	0	0	17	1	2	0	2	0
0	0	0	0	14	1	2	1	2	0
0	0	0	4	21	1	1	2	1	0
0	0	0	0	17	1	2	0	2	1
0	0	0	0	17	1	2	1	2	1
0	0	0	0	14	1	2	0	2	0
0	0	0	0	17	1	1	1	2	0
1	0	0	3	20	1	2	0	1	0
0	0	0	0	14	1	2	1	2	0
0	0	0	0	16	2	2	1	1	1
0	0	0	0	14	1	2	1	2	0
0	0	0	0	15	1	2	2	2	0
0	0	0	0	20	1	2	1	1	0
0	0	0	0	16	1	1		2	0
1	1	1	3	28	1	1	2	2	0
0	0	0	0	14	1	2	1	2	0
0	0	0	0	15	1	2	3	2	0
0	0	0	1	26	1	1	3	1	1
1	0	0	0	22	1	1	1	2	0
0	0	0	0	18	1	1	0	1	0
0	0	0	0	25	2	2	3	2	0
0	0	0	0	17	1	1	0	1	0
0	0	0	1	17	2	3	1	2	1
0	0	0	0	14	2	3	2	2	1
0	0	0	1	25	2	2	1	2	1
0	0	0	0	19	2	2	0	2	0
0	0	0	0	15	1	2	0	2	0
0	0	0	2	18	2	3	1	2	0
0	0	0	0	14	1	2	1	1	1
1	1	1	3	24	1	3	3	2	0
0	0	0	0	37	1	1	1	2	1
0	0	0	3	22	2	2	3	2	1
0	0	0	1	30	2	2	1	2	1
0	0	0	0	19	2	3	2	1	1
0	0	0	0	16	1	2	0	2	0
0	0	0	0	15	2	2	0	2	0

1	0	0	1	15	1	2	2	1	0
1	1	1	4	17	1	2	4	1	0
0	1	1	0	24	2	1	4	2	0
0	0	0	2	15	2	2	0	2	0
1	1	1	0	22	1	2	4	2	0
1	1	1		22	1	1	0	2	1
0	2	1	2	20	1	1	1	1	0
0	2	1	2	20	1	1	1	1	0
0	0	0	0	15	2	1	1	2	0
0	0	0	0	19	2	2	1	2	0
1	0	0	2	16	1	2	4	1	0
0	0	0	1	21	1	2	2	2	0
0	0	0	1	18	1	2	0	2	0
0	0	0	1	17	2	2	2	2	1
0	0	0	0	14	1	2	3	1	0
0	0	0	0	16	2	2	0	2	1
0	1	1	2	23	1	2	1	2	0
0	1	1	0	16	1	2	0	1	0
0	0	0	1	20	1	1	0	2	0
0	0	0	0	18	1	2	2	1	0
1	1	1	1	18	1	1	0	2	0
1	1	1	1	17	1	2	0	1	0
0	0	0	0	14	1	3	1	1	1
0	0	0	0	16	1	2	0	2	1
0	0	0	1	16	1	2	0	2	0
0	1	1	0	21	1	1	1	1	0
0	2	1	3	14	1	3	3	2	0
0	0	0	4	34	1	2	2	2	0
0	0	0	1	21	1	2	1	1	0
1	0	0	2	23	1	2	4	2	0
0	1	1	0	23	1	1	0	1	0
1	0	0	1	16	2	2	0	2	0
1	0	0	1	16	2	2	0	2	0
0	0	0	0	17	1	2	0	2	0
0	0	0	1	18	1	2	3	2	1
0	0	0	1	18	1	2	3	2	1
0	0	0	0	14	1	2	2	2	1
0	0	0	0	14	1	2	2	2	1
0	0	0	2	16	1	2	0	2	0
0	1	1	0	20	1	2	1	1	0
0	0	0	0	14	1	2	0	2	0
1	0	0	0	18	1	1	0	1	0
0	1	1	0	17	1	2	2	1	1
0	0	0	1	24	2	2	3	2	0
0	0	0	2	18	1	2	2	1	0
0	0	0	0	21	1	1	3	1	1
0	0	0	0	18	2	2	0	2	0

1	0	0	0	15	1	2	2	2	0
0	0	0	0	14	1	2	1	1	0
1	0	0	0	15	1	2	2	1	1
1	0	0	1	23	1	2	0	2	0
0	0	0	1	23	1	2	0	2	0
0	1	1	0	28	1	1	1	1	1
0	1	1	0	28	1	1	1	1	1
0	2	1	2	18	1	2	1	2	0
1	2	1	1	15	1	2	3	2	1
0	2	1	1	17	1	1	0	1	0
0	1	1	0	19	1	1	0	1	1
0	1	1	0	19	1	1	0	1	1
0	0	0	0	18	1	2	0	2	0
1	1	1	1	15	1	2	3	1	0
0	1	1	3	26	1	2	0	1	0
1	1	1	4	21	1	1	2	2	0
1	2	1	2	31	1	2	2	2	0
0	2	1	1	17	2	2	1	2	0
0	0	0	3	16	2	2	5	2	1
1	1	1	0	24	2	2	2	2	0
0	0	0	1	15	1	2	0	1	0
1	2	1	3	24	1	2	2	1	0
0	0	0	3	17	2	3	0	2	1
0	0	0	0	15	2	2	1	2	0
0	0	0	0	15	2	2	1	2	0
0	0	0	1	18	1	3	3	2	0
1	0	0	0	22	1	1	0	2	0
0	0	0	1	28	1	2	4	1	0
1	2	1	1	22	1	2	2	2	0
0	1	1	0	14	2	1	0	2	0
0	0	0	0	17	2	3	1	2	1
0	0	0	1	16	2	3	0	2	1
0	0	0	2	15	2	2	0	2	1
0	0	0	2	21	2	3	1	2	0
0	1	1	3	14	2	2	1	2	0
1	2	1	1	16	2	3	3	2	1
1	2	1	1	16	2	3	3	2	1
0	0	0	0	15	1	2	3	2	0
0	0	0	2	21	1	2	1	2	0
0	2	1	1	15	1	2	2	2	1
0	0	0	3		1	2	2	1	0
0	0	0	2	18	1	1	0	2	0
1	0	0	3	28	2	2	1	2	0
1	0	0	2	15	1	3	0	1	0
0	0	0	0	18	2	2	3	2	0
0	0	0	0	15	1	2	0	2	0
0	0	0	2	19	2	2	1	2	0

0	0	0	0	14	1	1	0	2	1
0	1	1	1	20	2	2	1	2	0
0	0	0	1	19	2	2	1	2	1
0	2	1	0	14	1	2	1	1	0
0	0	0	0	20	1	1	0	1	0
0	0	0	0	14	1	2	0	1	0
0	0	0	0	16	1	1	3	1	0
0	1	1	0	22	1	2	3	2	0
0	0	0	0	18	1	2	2	2	0
0	0	0	0	23	1	2	2	2	1
0	2	1	0	14	2	1	1	2	1
0	0	0	0	28	2	1	0	2	0
0	0	0	2	16	1	1		2	0
0	0	0	0	14	2	1	0	2	1
0	1	1	0	14	2	2	1	1	1
0	0	0	0	28	1	1	0	1	0
0	0	0	0	19	1	1	0	1	0
0	0	0	0	25	1	1	1	2	0
0	1	1	0	22	1	1	2	1	0
0	0	0	1	17	1	2	0	1	0
0	0	0	0	19	1	2	0	1	0
0	0	0	0	18	1	2	0	2	0
0	0	0	0	14	2	2	0	2	0
0	0	0	1	21	1	2	3	1	0
0	0	0	0	14	1	2	1	1	0
0	0	0	0	23	2	2	3	2	0
0	0	0	0	14	1	2	2	2	0
0	0	0	0	14	1	1	1	2	0
0	0	0	0	14	1	2	1	1	0
0	0	0	1	18	1	1	1	2	1
0	1	1	0	20	1	1	1	1	0
0	0	0	3	21	1	2	5	2	0
0	1	1	0	23	1	1	3	1	0
0	0	0	0	19	1	2	0	2	1
0	0	0	0	28	2	2	1	1	1
0	0	0	0	28	2	2	1	2	1
0	0	0	0	17	1	2	2	2	1
0	0	0	0	17	1	2	1	2	0
0	0	0	0	35	2	3	0	2	1
0	0	0	0	14	1	2	1	2	0
0	0	0	0	14	1	2	5	1	0
0	0	0	0	28	1	1	0	1	0
0	2	1	0	14	1	1	1	1	0
0	0	0	0	20	1	2	4	2	0
0	0	0	0	19	2	2	1	2	0
0	0	0	1	14	2	1	1	1	0
0	0	0	0	16	1	2	1	2	1

0	0	0	0	24	1	2	1	2	0
0	0	0	2	29	1	2		1	0
0	2	1	0	24	2	2	1	2	1
0	0	0	2	14	2	3	0	2	1
0	0	0	1	17	2	2	1	2	0
0	0	0	0	21	2	2	1	2	0
0	2	1	0	23	1	1	2	1	0
0	0	0	0	29	1	2	3	2	1
0	0	0	1	26	1	2	3	2	1
1	1	1	1	32	1	1	1	1	0
0	1	1	1	22	1	1	0	1	1
0	0	0	0	26	1	2	1	2	1
0	2	1	0	34	1	1	1	2	1
0	0	0	0	28	1	1	3	1	0
0	2	1	2	19	1	2	1	2	1
0	0	0	1	17	1	1	1	2	1
0	0	0	0	14	2	2	1	2	0
1	0	0	0	28	1	2	2	2	1
1	2	1	0	22	2	2	3	2	1
0	0	0	1	23	2	3	2	2	1
0	1	1	0	16	1	3	1	1	1
0	2	1	0	19	1	2	2	2	0
0	1	1	1	23	1	2	2	2	0
0	1	1	1	19	1	2	1	2	0
0	2	1	0	18	1	2	1	2	1
0	0	0	1	16	2	2	0	2	0
0	0	0	0	19	2	2	1	2	0
1	0	0	2	17	1	1	1	1	0
1	1	1	1	18	1	1	0	1	0
1	0	0	0	17	1	1	0	1	0
1	1	1	1	21	1	1	0	1	0
0	2	1	0	27	1	2	0	1	1
0	2	1	0	27	1	2	0	1	1
1	0	0	0	21	2	2	1	2	0
0	0	0	2	20	1	2	2	1	1
0	1	1	0	17	2	2	1	2	0
0	2	1	0	24	2	1	0	2	1
0	0	0	0	16	2	3	1	2	1
0	0	0	0	18	1	2	1	2	0
0	2	1	0	18	1	2	1	2	0
0	1	1	1	23	1	2	0	2	1
1	1	1	1	24	1	2	1	2	1
0	0	0	0	20	1	2	3	1	0
0	2	1	1	24	1	1	0	1	1
0	0	0	0	14	1	1	1	1	1
1	2	1	1	18	1	2		2	0
0	0	0	1	18	1	1	1	1	1

0	0	0	0	17	1	1	1	1	0
0	0	0	1	25	2	1	0	2	0
0	0	0		15	1	1	1	2	1
0	1	1	0	26	2	1	0	2	1
0	0	0	0	14	2	2	2	2	0
0	0	0	0	19	1	1	1	1	0
0	0	0	0	14	2	2	1	2	0
0	0	0	1	20	1	2	2	1	0
1	1	1	0	16	1	1	1	2	0
0	0	0	0	14	1	1	0	2	1
0	0	0	0	16	1	1	1	2	0
0	0	0	0	18	1	1	0	1	0
0	2	1	0	20	1	1	0	1	0
0	2	1	0	16	1	1	0	1	0
0	0	0	0	17	1	1	4	1	0
0	0	0	0	15	1	1	0	2	0
0	2	1	1	23	1	1	0	1	0
0	0	0	0	17	2	2	0	2	0
0	0	0	0	15	2	2	0	2	1
0	0	0	0	15	2	2	0	2	1
0	0	0	0	17	2	2	0	2	0
0	0	0	0	15	1	2	0	1	0
0	2	1	0	18	1	1	2	1	0
0	0	0	0		1	1	0	1	0
0	0	0	0	17	1	1	0	2	0
0	2	1	0	15	1	1	0	2	0
0	0	0	0	15	2	1	0	2	0
0	0	0	0	14	1	1	1	2	0
0	2	1	0	16	1	1	0	1	0
1	1	1	0	15	1	1	1	2	0
1	1	1	0	14	1	1	0	1	0
0	1	1	1	20	2	1	0	1	1
0	0	0	0	14	2	1	4	2	1
0	0	0	3	15	1	1	1	2	1
0	1	1	1	18	1	1	0	1	0
0	0	0	0	14	1	1	0	1	0
0	0	0	0	16	1	1	2	2	0
0	0	0	0	21	1	2	0	2	1
0	0	0	0	14	2	2	4	2	1
0	2	1	0	15	1	1	0	1	0
0	2	1	0	15	1	1	0	1	0
0	0	0	0	17	2	2	0	2	1
0	0	0	0	14	1	2	0	2	0
0	0	0	0	18	1	2	1	2	0
0	0	0	0	15	2	2	1	2	0
0	2	1	0	14	1	1	0	2	0
0	0	0	0	16	1	1	1	1	0

0	2	1	0	20	1	1	1	1	0
0	2	1	0	20	1	1	1	1	0
0	0	0	0	15	2	2	2	2	0
0	2	1	0	24	1	1	1	1	0
0	0	0	0	23	1	1	3	1	0
0	0	0	0		1	1	0	2	0
0	2	1	0	25	1	1	0	1	0
0	2	1	0	17	1	1	0	1	0
0	2	1	0	24	1	1	0	1	1
0	2	1	0	17	1	2	1	1	0
0	2	1	0	17	1	1	0	1	1
0	0	0	0	19	1	1	0	1	0
0	2	1	6	14	2	1	0	2	0
0	2	1	0	16	2	3	0	2	1
0	2	1	0	16	2	3	0	2	1
0	0	0	0	19	2	2	1	2	0
0	0	0	1	18	1	1	2	1	0
1	1	1	4	24	1	2	3	2	1
1	0	0	1	17	2	1	2	2	0
0	0	0	3	33	2	3	4	2	1
0	0	0	0	16	1	2	1	2	0
0	2	1	0	18	1	2	2	2	0
0	0	0	0	20	1	1	0	2	1
0	0	0	0	23	2	1	1	2	1
0	1	1	3	26	2	1	2	2	0
1	2	1	0	18	1	2	1	1	0
0	0	0	0	18	2	2	4	2	1
0	0	0	0	22	1	2	2	1	0
0	2	1	1	25	2	2	3	1	0
0	2	1	0	17	2	2	0	2	0
1	1	1	0	18	2	2	1	2	1
0	1	1	2	17	1	2	0	2	0
0	0	0	1	20	2	2	1	1	0
0	1	1	0	20	1	1	0	2	0
0	0	0	0	21	1	1	1	2	0
1	1	1	1	15	1	1	0	1	0
0	0	0	2	14	2	1	2	2	0
1	0	0	1	17	2	2	5	2	0
0	2	1	0	18	2	2	4	2	1
0	2	1	3	18	1	2	1	2	0
0	1	1	1	15	1	1	1	1	0
0	1	1	0	18	1	2	2	1	0
0	1	1	0	23	1	2	2	2	1
0	2	1	1	15	2	3	4	2	1
0	2	1	1	22	1	1	2	1	1
0	2	1	2	26	1	1	0	1	0
0	1	1	0	32	1	1	1	2	0

0	1	1	0	32	1	1	1	2	0
1	0	0	1	18	1	1	0	2	0
0	2	1	0	31	1	2	1	2	0
0	1	1	2	23	2	3	1	2	1
0	0	0	0	21	2	2	1	2	0
0	0	0	0	15	1	2	1	1	0
0	0	0	0	18	1	1	0	2	1
0	0	0	0	18	1	1	0	2	1
1	1	1	1	18	1	1	1	1	1
1	2	1	2	21	1	1	3	2	0
1	0	0	4	23	1	2	1	1	0
0	0	0	1	21	1	1	2	1	0
0	2	1	1	23	2	2	2	2	0
0	0	0	0	22	2	2	2	2	1
0	1	1	0		1	2	0	1	0
0	0	0	0	18	1	1	1	1	1
0	0	0	1	19	1	1	0	2	0
0	0	0	0	15	1	2	0	1	0
1	2	1	4	25	1	1	1	1	1
1	1	1	2	20	1	1	3	1	0
1	2	1	3	23	2	1	0	2	0
1	0	0	0	15	1	1	3	1	0
0	0	0	1	17	1	1	1	2	0
0	0	0	0	22	1	1	2	2	0
0	0	0	3	24	1	2	5	1	1
0	0	0	0	25	1	2	2	1	1
0	0	0	0	18	2	2	2	2	1
0	0	0	0	14	2	1	1	2	0
1	1	1	3	20	1	1	1	1	1
0	1	1	3	19	1	1	2	2	1
0	0	0	3	21	2	1	1	2	1
0	0	0	0	17	1	1	2	2	0
1	1	1	3	22	1	1	0	1	1
0	0	0	0	17	1	1	1	1	1
0	2	1	1	19	1	2	2	1	1
0	1	1	1	18	2	2	2	2	1
0	1	1	2	15	2	2	1	2	0
1	0	0	1	17	2	3	2	2	1
0	0	0	1	15	2	3	1	2	1
1	2	1	0	19	2	2	2	2	1
0	0	0	0	16	1	1	2	1	0
0	0	0	0	17	1	2	1	1	0
0	0	0	0	16	2	3	0	2	1
0	2	1	1	21	1	2	1	2	0
0	0	0	0	16	1	1	2	2	0
0	0	0	1	26	1	2	1	2	0
0	1	1	1	22	1	2	2	1	0

0	0	0	0	17	2	2	0	2	0
0	2	1	1	21	1	1	2	2	0
0	0	0	0	18	1	1	2	2	0
0	0	0	0	15	2	2	3	2	1
0	2	1	0	16	1	1	1	1	0
0	2	1	1	20	1	1	0	1	0
0	0	0	0	19	1	2	0	2	0
0	2	1	0	16	1	2	1	2	0
0	0	0	0	16	1	1	0	2	1
0	0	0	0		2	1	1	2	0
0	1	1	0	23	1	1	0	2	0
0	1	1	0	16	1	2	2	1	0
0	2	1	0	20	1	2	1	2	0
0	0	0	0	16	2	2	1	2	0
0	0	0	1	17	1	1	0	1	0
0	2	1		25	1	2		1	0
0	0	0	0	16	2	2	2	2	0
0	0	0	1	19	1	2	1	1	0
0	0	0	1	17	2	2	1	2	0
0	0	0	2	14	2	2	2	2	0
0	0	0	0	16	1	1	2	1	0
0	0	0	0	25	1	2	1	1	0
1	0	0	2	20	1	2	1	1	0
0	0	0	0	16	2	3	1	2	1
0	0	0	0	17	1	2	2	1	0
0	0	0	0	23	1	1	0	2	0
0	0	0	0	18	1	2	0	2	0
0	0	0	0	17	2	2	0	2	1
0	2	1	1	20	1	1	1	1	0
0	0	0	0	21	1	2	2	2	0
0	0	0	2	24	2	2	2	2	1
0	2	1	0	25	1	2	2	2	1
0	0	0	0	20	2	2	1	2	0
0	0	0	0	15	1	2	2	2	0
0	2	1	0	15	2	3	1	2	1
0	2	1	0	18	1	2	2	2	1
0	2	1	0	21	1	1	2	1	0
0	0	0	0	19	1	1	2	1	0
0	0	0	0	18	2	2	1	2	0
0	0	0	1	22	1	2	1	2	1
0	0	0	0	17	2	2	0	2	0
0	1	1	0	20	2	2	1	2	1
0	0	0	1	22	1	2	2	2	1
0	2	1	0	20	1	1	0	2	0
0	1	1	0	20	2	2	0	2	0
0	0	0	0	17	2	2	1	2	1
0	0	0	0	18	2	3	1	2	1

0	0	0	1	20	2	2	1	2	1
0	1	1	1	25	2	2	1	2	0
0	0	0	1	18	2	2	1	2	0
0	0	0	0	14	1	2	2	1	0
0	0	0	1	19	1	2	0	1	0
1	2	1	1	23	1	1	1	1	0
1	2	1	0	19	1	2	0	2	0
0	0	0	1	20	2	2	1	2	1
0	0	0	0	16	1	1	2	1	0
0	0	0	1	23	1	1	1	2	0
0	1	1	0	19	1	2	2	2	0
0	0	0	0	17	1	1	0	2	0
0	0	0	0	17	1	1	0	2	0
0	0	0	0	17	1	2	1	1	1
0	0	0	0	14	1	1	0	1	0
0	1	1	0	14	2	1	1	2	0
0	0	0	0	14	1	1	0	1	0
0	0	0	0	14	1	2	0	1	0
0	0	0	0	14	1	1	0	2	0
1	0	0	0	16	1	2	0	1	0
1	0	0	0	16	1	2	0	1	0
1	0	0	3	17	1	1	0	2	0
1	2	1	3	16	1	2	4	2	1
0	0	0	0	16	1	1	0	2	0
0	0	0	0	18	1	1	0	1	0
0	0	0	0	17	1	1	0	1	0
1	0	0	0	14	1	1	1	1	0
0	0	0	0	17	2	1	1	2	0
0	1	1	0	14	2	1	1	2	0
0	0	0	0	14	2	1	0	2	0
0	0	0	0	14	1	1	0	1	0
0	0	0	0	23	1	1	0	2	0
0	1	1	0	14	2	2	1	2	0
1	1	1	0	17	1	1	1	1	0
0	2	1	0	20	1	1	0	1	0
0	0	0	1	14	1	1	1	1	0
0	0	0	0	14	1	1	1	1	0
0	0	0	0	15	1	1	0	1	0
1	1	1	2	21	1	2	2	2	0
1	0	0	0	16	1	3	3	2	0
0	0	0	0	22	1	1	0	2	1
0	0	0	1	17	1	2	1	2	0
0	1	1	1	26	1	1	2	2	1
0	0	0	0	22	1	2	1	2	0
0	0	0	2	17	1	2	3	2	0
1	0	0	2	27	1	2	2	2	1
0	0	0	0	22	1	2	0	1	0

1	0	0	1	28	1	1	0	1	0
0	1	1	3	24	1	1	0	2	0
1	0	0	2	29	1	1	0	1	0
0	0	0	0	17	1	2	1	1	0
0	0	0	1	18	1	1	0	2	0
0	0	0	0	15	1	1	0	1	0
1	1	1	2	23	1	1	0	1	0
0	0	0	0	15	1	1	0	2	0
0	0	0	0	28	1	1	1	2	0
0	0	0	1	18	2	2	1	2	0
1	1	1	4	35	1	2	1	2	0
0	0	0	0	17	1	1	2	2	0
0	1	1	0	14	1	1	2	1	0
0	0	0		16	1	1	0	1	0
0	0	0		16	1	2	0	2	0
1	0	0	0	24	1	2	1	2	0
0	0	0	0	17	1	2	2	2	0
0	2	1		21	1	2	0	2	0
0	0	0		20	1	2	0	2	1
1	0	0	0	17	2	2	4	2	0
1	2	1	0	18	2	2	1	2	0
1	1	1	2	18	2	2	5	2	1
1	0	0	2	16	2	2	3	2	1
0	0	0	0	16	1	1	1	2	0
0	0	0	7	17	1	2	1	1	0
1	2	1		18	1	2	2	2	0
0	0	0	0	16	2	2	1	2	0
0	0	0	0	16	2	2	1	2	0
0	0	0	1	16	1	2	1	2	1
0	0	0	1	16	1	2	1	2	1
0	0	0	0	15	1	1	1	2	0
0	0	0	0	16	1	2	4	2	1
0	0	0	1	14	2	3	1	2	0
0	0	0		17	1	2	0	2	1
0	0	0		14	2	2	0	2	0
0	0	0		14	2	2	0	2	0
0	0	0		20	2	3	1	2	1
0	0	0		18	2	3	0	2	1
1	0	0	2	19	1	1	2	2	0
0	0	0	1	14	2	2	2	2	0
0	0	0	0	14	1	3	1	2	0
0	0	0	0	14	1	3	1	2	0
0	0	0	0	15	2	3	0	2	1
0	0	0	0	16	2	2	0	2	0
0	2	1	1	17	1	1	2	1	0
0	0	0	0	19	1	1	3	2	0
0	0	0	0	14	1	3	4	2	1

0	2	1	0	16	2	2	1	1	0
0	0	0	0	17	2	2	1	2	0
0	2	1	0	17	2	2	4	2	0
0	2	1	0	18	1	1	3	2	0
0	0	0		28	1	1	1	1	0
0	0	0		15	1	2	2	1	0
0	0	0	1	25	1	2	3	1	0
0	0	0	0	15	1	2	2	2	0
1	0	0	1	16	1	2	1	2	0
0	0	0	0	22	1	1	0	2	0
0	2	1	1	15	1	1	3	2	0
0	2	1	2	28	1	1	4	1	1
0	0	0	0	15	1	1	0	2	0
1	1	1		15	1	1	0	1	0
0	0	0		15	2	2		2	0
0	1	1	1	16	1	2	3	2	0
0	0	0	0	15	1	2	2	2	1
0	1	1	0	17	1	2	3	1	0
0	0	0	0	15	1	1	1	2	0
0	1	1		17	1	1	2	1	0
1	1	1	2	18	1	1	1	2	0
1	0	0	3	16	1	2	2	2	0
0	0	0	0	15	2	2	0	2	0
0	0	0	1	17	1	2	0	1	0
0	1	1	0	20	2	2	2	2	0
1	0	0	0	14	2	2	3	2	0
0	2	1	1	18	1	1	1	2	0
0	2	1		17	1	1	0	2	0
0	0	0		19	2	2	1	2	0
0	0	0	0	15	2	2	2	2	1
0	0	0	0	17	2	2	2	2	0
0	1	1	2	17	1	2	4	2	0
0	2	1	0	22	2	2	0	2	0
0	0	0	1	31	1	1	2	1	0
0	0	0	0	19	2	2	1	2	0
0	2	1	1	18	2	2	0	2	1
0	0	0		14	1	2	0	2	0
1	1	1		33	1	1	0	1	0
1	0	0		15	1	3	2	1	1
0	1	1	2	30	1	1	1	1	0
0	2	1	0	19	1	1	0	1	0
0	0	0		18	2	1	4	1	0
1	0	0		31	2	2	2	2	0
0	0	0	0	17	1	2	3	2	0
0	0	0		20	1	3	0	2	0
0	0	0	0	16	1	2	0	1	0
0	0	0		16	2	2	2	2	0

0	0	0	0	16	1	1	1	1	0
0	0	0	0	15	1	1	1	1	0
0	0	0	0	15	1	2	1	2	0
0	0	0	0	14	2	2	1	2	0
0	0	0	0	15	1	2	0	2	0
0	0	0	1	15	2	2	0	2	0
1	1	1	4	24	1	2	4	2	1
0	0	0	0	15	1	2	0	2	0
0	0	0	0	17	1	1	1	1	0
0	0	0	1	17	2	2	2	2	1
1	0	0	2	15	1	1	2	2	0
0	0	0	0	15	1	1	0	2	1
0	0	0	0	15	1	2	2	2	0
0	0	0	0	16	1	2		2	0
0	0	0	1	18	1	1	0	1	0
0	1	1	0	21	1	1	1	1	1
0	0	0	0	19	1	2	2	2	0
0	0	0	0	14	1	2	3	2	0
0	2	1	0	24	1	1	1	1	0
0	0	0	0	15	1	2	0	2	1
0	0	0	0	14	1	2	1	2	0
1	0	0	1	19	2	2	3	2	1
0	0	0	0	21	1	2	0	1	0
0	2	1	2	14	2	2	0	2	1
1	0	0	0	14	1	1	0	1	0
0	0	0	0	15	2	2	1	2	0
0	1	1	0	17	1	2	5	1	1
0	0	0	1	17	1	1	2	2	1
0	0	0	3	17	2	2	0	2	0
0	0	0	3	17	2	2	0	2	0
0	0	0	1	15	1	2	1	2	0
0	1	1	1		1	2	0	1	1
0	0	0	0	14	1	1	0	2	0
0	0	0	1	18	2	3	0	2	1
0	0	0	3	22	2	2	2	2	0
1	1	1	4	19	1	2	1	1	0
0	0	0	0	19	2	1	1	1	0
0	0	0	0	19	2	1	1	1	0
0	0	0	0	17	2	2	0	2	0
1	0	0	3	16	1	1	0	1	0
0	0	0	3	21	1	2	1	1	0
0	0	0	0	14	1	2	0	2	0
1	1	1	6	21	1	2	1	1	0
0	0	0	0	15	1	2	0	1	0
0	0	0	0	33	1	2	2	2	1
0	0	0	0	33	1	2	2	2	1
0	0	0	0	15	1	2	3	2	0

0	0	0	0	15	1	2	3	2	0
0	0	0	4	16	1	2	0	2	1
1	1	1	5	23	1	2	1	1	0
0	0	0	0	14	1	1	1	2	0
0	0	0	0	20	1	2	4	2	0
1	0	0	5	19	1	2	1	1	0
0	0	0	1	17	1	2	2	1	0
0	0	0	0	17	1	2	2	2	0
0	0	0	0	17	1	2	1	1	0
0	0	0	3	19	1	2	1	1	0
0	0	0	2	17	1	2	1	1	0
0	0	0	2	17	1	2	1	1	0
0	0	0	3	18	1	2	0	2	0
0	0	0	1	17	1	3	2	2	1
0	0	0	2	23	1	2	1	1	1
1	1	1	5	30	1	2	2	1	1
0	0	0	0	16	1	2	5	1	1
0	0	0	0	14	1	2	0	1	0
0	0	0	0	19	2	2	1	2	0
0	0	0	0	23	1	2	1	2	0
0	2	1	1	22	2	2	0	2	1
0	0	0	0	15	1	1	1	1	0
0	0	0	1	20	1	2	0	2	0
0	0	0	0	17	1	2	1	1	0
1	1	1	2	30	1	2	1	2	1
0	1	1	2	19	2	2	1	2	0
1	0	0	0	19	1	2	0	1	0
0	0	0	0	19	1	2	1	2	0
0	0	0	0	19	1	2	1	2	0
0	0	0	0	22	1	2	2	1	0
0	0	0	1	21	2	2	0	2	1
1	1	1	4	34	1	1	0	1	0
1	1	1	1	18	1	1	1	2	1
0	0	0	1	20	1	2	1	1	0
0	0	0	1	19	2	2	1	1	0
1	1	1	5	22	1	1	0	1	0
0	2	1	3	26	2	3	2	2	1
1	0	0	0	28	1	1	2	2	0
0	1	1	0	17	1	2	2	1	0
1	1	1	0	16	2	2	3	1	0
0	0	0	3		2	1	1	2	0
1	0	0	3	23	2	3	3	2	1
1	0	0	0	17	1	3	2	2	1
1	2	1	1	23	1	3	2	2	0
1	2	1	1	23	1	3	2	2	0
0	0	0	2	18	1	3	3	2	0
0	0	0	1	18	2	2	2	2	0

0	0	0	1	17	1	2	0	2	0
1	0	0	1	22	1	1	0	1	1
0	2	1	1	22	2	3		2	1
0	2	1	2	18	2	3	2	2	1
0	1	1	0	15	2	2	0	1	1
0	1	1	0	15	2	2	0	1	1
0	2	1	0	25	2	3	3	2	1
0	2	1	0	25	2	3	3	2	1
0	2	1	0	25	2	3	3	2	1
0	0	0	4	18	1	2	1	2	0
0	0	0	1		1	2	2	1	0
0	0	0	0	14	1	2	0	2	1
1	1	1	2	19	1	2	1	2	0
0	0	0	0	14	2	2	1	2	0
1	0	0	2	19	2	2	0	2	0
0	0	0	1	14	1	2	2	2	1
0	2	1	0	35	2	1	1	2	0
0	0	0	0	14	1	2	1	1	0
0	0	0	0	14	1	1	1	1	0
0	2	1	0	19	1	2	3	2	0
0	0	0	1	18	1	2	2	2	1
1	0	0	4	17	1	1	1	1	0
0	0	0	0	20	1	2	1	2	1
0	1	1	2	18	1	1	1	1	0
0	0	0	2	19	1	2	5	2	1
0	0	0	0	14	1	2	0	1	0
0	0	0	0	14	1	2	3	1	0
0	0	0	1	15	1	1	1	1	0
0	0	0	0	21	1	2	1	2	0
0	2	1	0	17	1	2	0	1	0
0	0	0	0	14	2	2	0	2	0
0	0	0	0	14	1	2	0	1	0
0	2	1	0	22	2	2	1	2	0
0	1	1	1		1	1	2	1	0
0	0	0	0	14	1	2	0	1	0
0	2	1	0	20	2	2	2	2	0
0	0	0	0	18	1	2	3	2	0
0	1	1	0	14	1	2	1	1	0
1	0	0	1	15	1	2	1	2	0
0	0	0	0	14	1	2	0	1	1
0	0	0	0	29	1	1	5	1	0
0	1	1	0	14	1	2	0	2	0
0	2	1	0	14	2	1	1	2	0
0	2	1	0	19	1	1	5	1	0
0	2	1	0	26	2	2	0	2	0
0	0	0	0	17	1	2	5	1	0
0	0	0	0	16	1	2	0	1	0

0	0	0	0	14	1	1	3	1	0
0	1	1	1	18	2	2	0	2	1
0	0	0	0	19	1	2	0	1	0
0	0	0	0	14	1	1	0	1	0
0	0	0	0	15	1	2	0	1	0
0	0	0	0	18	1	2	3	1	0
0	0	0	0	14	1	2	5	1	0
1	0	0	1	14	2	2	5	2	1
0	0	0	0	23	1	2	2	2	1
0	2	1	0	17	1	2	3	2	1
0	2	1	1	17	2	2	2	2	0
0	0	0	0	15	1	2	3	2	1
0	0	0	0	28	1	2	2	1	0
0	0	0	1	14	1	2	0	2	0
0	0	0	0	14	2	2	2	2	0
0	1	1	0	14	1	1	0	1	0
0	2	1	5	18	2	3	5	2	1
0	1	1	0	20	1	1	5	1	0
0	0	0	0	14	1	1	0	1	0
0	0	0	0	14	1	1	0	1	0
0	0	0	3	29	2	1	2	2	0
0	0	0	0	14	2	2	0	2	0
0	1	1	1	19	2	2	4	2	1
0	0	0	0	22	1	2	3	2	1
0	0	0	0	25	2	3	0	2	1
0	0	0	0	18	1	2	0	1	0
0	0	0	0	20	1	1	5	1	0
0	0	0	0	15	1	2	4	1	0
0	0	0	0	14	1	2	4	1	0
1	0	0	0	18	2	3	1	2	1
0	0	0	1	20	1	1	0	1	0
0	0	0	0	47	2	2	1	2	0
0	0	0	0	20	1	2	5	1	0
0	0	0	0	14	1	2	0	1	0
0	0	0	0	24	1	1	3	1	0
0	1	1	0	23	2	1	0	2	1
0	0	0	0	19	2	2	3	2	1
0	0	0	0	14	1	2	5	1	0
0	0	0	0	14	2	2	5	2	1
0	2	1	0	16	1	2	2	2	0
0	1	1	0	14	2	3	5	2	1
0	1	1	10	56	1	2	5	1	0
0	0	0	1	20	1	2	4	2	1
0	1	1	2	25	1	2	3	2	1
0	0	0	0	18	2	1	1	2	0
0	1	1	0	25	1	1	0	1	0
0	2	1	0	16	1	2	4	2	0

0	2	1	0	28	1	1	1	1	1
0	0	0	2	34	1	1	3	1	1
0	1	1	0	24	1	1	1	1	0
1	0	0	0	20	1	2	1	1	0
0	0	0	1	21	1	1	1	2	1
1	2	1	3	27	1	1	1	1	1
0	2	1	2	23	1	1	1	2	1
0	0	0	0	14	2	2	3	2	1
1	1	1	3	25	1	1	1	1	1
0	0	0	0	17	2	1	0	2	0
0	1	1	1	23	1	2	0	1	0
0	1	1	1	23	1	2	0	1	0
0	2	1	2	19	1	1	3	1	0
0	2	1	2	19	1	1	3	1	0
0	1	1	0	25	1	2	2	2	0
0	1	1	1	25	1	1	1	2	0
0	1	1	1	19	1	2	3	1	1
0	1	1	1	20	1	2	4	1	1
1	0	0	2	23	1	2	1	2	1
0	0	0	1	15	2	3	1	2	1
0	1	1	0	23	1	2	3	1	0
1	1	1	2	24	1	1	0	2	1
0	0	0	0	15	1	1	0	2	0
0	2	1	3	25	1	1	1	1	0
0	0	0	0	16	1	1	2	1	0
0	1	1	0	21	1	1	1	1	0
0	0	0	0	19	1	2	3	1	0
0	1	1	0	17	1	1	2	1	0
0	1	1	1	24	1	2	0	1	0
0	1	1	1	24	1	2	0	1	0
1	2	1	1	21	1	1	3	1	1
0	2	1	1	23	1	2	4	2	1
0	1	1	2	25	2	3	2	2	1
0	1	1	0	17	1	2	2	2	1
0	0	0	1	22	2	2	2	2	1
1	2	1	3	23	1	2	2	2	0
0	0	0	2	23	1	2	5	2	0
0	2	1	1	21	1	1	0	2	1
1	1	1	1	22	1	1	1	1	1
0	0	0	0	29	1	1	1	1	0
0	0	0	0	29	1	1	1	1	0
0	0	0	1	15	1	2	2	2	0
0	0	0	0	17	1	2	0	1	0
0	0	0	1	22	2	1	1	2	0
0	0	0	0	20	1	1	1	1	0
0	0	0	0	16	1	1	1	1	1
0	0	0	2	17	2	3	3	2	1

1	0	0	0	15	1	1	1	2	0
0	0	0	0	14	2	1	0	2	0
1	0	0	0	17	1	2	3	1	0
0	0	0	0	18	1	1	3	1	0
0	0	0	0	16	2	2	1	1	1
0	0	0	0	22	1	2	3	1	0
0	0	0	1	15	2	2	0	2	0
0	0	0	0	14	1	2	0	2	0
0	0	0	0	20	2	2	1	2	0
0	0	0	0	17	1	2	2	2	0
0	0	0	0	17	1	2	2	2	0
0	0	0	0	21	1	1	1	1	0
0	0	0	0	14	1	2	1	2	0
1	1	1	0	29	1	1	4	2	1
0	2	1	0	15	1	1	0	1	0
0	2	1	0	15	1	1	0	1	0
0	2	1	0	16	2	2	4	1	1
0	0	0	0	20	1	1	0	1	0
0	0	0	0	19	1	2	0	1	0
0	0	0	0	17	1	2	0	2	0
0	0	0	0	14	1	1	0	1	0
0	0	0	1	17	2	2	0	2	0
0	0	0	0	23	1	1	1	2	0
0	0	0	1	21	1	1	0	1	0
0	0	0	0	15	2	2	2	2	0
0	0	0	0	15	2	2	2	2	0
0	0	0	0	16	1	1	3	2	0
0	0	0	0	15	1	1	1	1	0
0	0	0	0	18	1	1	0	1	0
0	0	0	0	20	1	1	2	1	0
0	0	0	0	14	2	2	0	2	0
0	0	0	0	21	2	1	5	2	1
0	0	0	0		1	1	1	1	0
0	0	0	0	14	1	2	1	1	0
0	0	0	3	18	1	2	3	1	0
0	0	0	3	21	2	1	2	2	0
0	0	0	0	16	2	2	2	2	0
0	0	0	1	20	1	1	2	2	0
0	0	0	1	25	1	2	1	2	0
0	0	0	1	25	1	2	1	2	0
0	2	1	2	18	1	2	3	2	0
0	0	0	0	21	2	2	3	2	1
0	1	1	1	18	2	2	4	2	0
1	0	0	3	18	1	2	3	2	0
0	0	0	1	24	1	1	0	1	0
0	0	0	1	24	1	1	0	1	0
0	0	0	0	14	2	2	2	2	1

1	2	1	1	27	1	1	3	2	0
1	0	0	1	28	1	1	1	1	0
0	0	0	4	17	2	1	0	2	0
0	0	0	0	25	1	1	0	1	0
0	1	1	1	22	1	2	0	1	0
0	2	1	0	23	2	1	0	2	0
0	0	0	3	23	1	2	2	2	0
0	0	0	2	24	1	1	2	2	0
1	1	1	3	19	2	2	0	1	1
1	1	1	3	25	1	2	2	2	0
1	1	1	3	17	1	2	0	2	0
0	0	0	0	20	1	2	1	1	1
1	2	1	5	19	1	2	3	1	0
1	1	1	1	14	1	2	2	1	0
0	1	1	3	19	2	2	2	2	0
0	0	0	2	14	2	2	2	2	0
0	0	0	0	15	1	2	3	1	0
1	1	1	1	16	1	2	0	1	0
0	0	0	0	14	1	2	2	1	1
0	0	0	1	18	2	2	2	2	0
0	1	1	1	18	1	1	2	2	0
1	0	0	3	15	1	1	5	1	0
1	0	0	3	15	1	1	5	1	0
1	0	0	4	18	1	1	2	1	0
1	0	0	4	18	1	1	2	1	0
0	0	0	0	20	1	2	1	1	0
0	1	1	3	21	1	1	2	1	0
1	1	1	2	22	2	3	2	2	1
0	1	1	1	20	1	1	0	1	1
0	1	1	1	20	1	1	0	1	1
0	1	1	0	21	1	1	2	1	0
0	1	1	0	21	1	1	2	1	0
0	1	1	0	20	1	1	1	2	1
0	0	0	0	21	1	1	0	1	0
0	0	0	0	14	1	1	2	1	1
0	0	0	0	16	1	1	2	1	0
0	1	1	0	26	1	2	0	1	1
0	0	0	0	20	1	2	2	1	0
0	0	0	0	16	1	2	2	2	0
0	1	1	0	19	2	2	2	2	0
1	2	1	0	15	1	1	2	1	0
0	2	1	0	17	2	2	2	2	0
0	2	1	0	19	1	1	2	1	0
0	0	0	0	17	1	2	0	2	0
0	0	0	1	22	2	2	1	2	0
0	0	0	0	18	1	2	0	2	0
0	0	0	1	21	2	2	0	1	0

0	2	1	0	19	1	1	2	1	0
0	2	1	0	19	1	2	1	1	1
1	1	1	0	17	1	2	2	2	0
1	0	0	1	31	1	1		1	0
0	0	0	1	23	1	2	2	1	0
0	1	1	3	19	2	2	0	2	1
0	0	0	5	16	1	2	1	2	1
0	1	1	2	21	1	1	2	1	0
0	2	1	0	19	1	2	0	1	1
0	2	1	2	26	1	1	2	2	1
0	0	0	0	17	1	2	1	2	0
0	0	0	0	18	1	2	0	1	0
0	0	0	0	16	1	2	0	2	0
0	0	0	0	18	1	1	4	1	0
0	1	1	1	25	1	1	1	2	0
0	0	0	0	19	2	1	2	2	0
0	0	0	1	18	2	2	2	1	0
0	1	1	0	20	2	2	2	2	1
0	0	0	1	17	1	2	2	2	0
0	0	0	1	19	1	2	2	2	1
0	0	0	3	17	1	2	3	1	0
0	0	0	1	18	1	2	2	2	0
0	0	0	1	14	2	1	2	1	0
0	0	0	0	19	2	3	1	2	1
0	2	1	0	15	1	1	1	1	0
0	0	0	0	14	2	1	0	2	1
0	0	0	1	14	1	3	0	2	0
0	0	0	0	14	2	3	2	2	1
0	2	1	1	22	1	2	2	1	0
0	0	0	0	14	2	3	0	2	1
0	0	0	0	19	1	2	1	1	1
0	2	1	3	23	2	3	1	2	1
0	0	0	0	17	1	2	2	1	0
0	0	0	2	15	2	3	2	2	1
0	2	1	0	17	1	3	1	1	0
0	0	0	1	15	1	2	1	1	0
1	0	0	0	20	2	2	2	2	0
0	0	0	0	14	1	2	0	2	0
0	0	0	0	22	1	2	3	2	0
0	0	0	0	26	2	1	2	2	1
0	0	0	0	19	1	2	3	1	0
0	0	0	0	16	2	2	1	2	0
0	0	0	1	22	2	3	1	2	1
0	0	0	1	19	2	2	1	2	0
0	0	0	0	17	1	1	0	2	0
0	0	0	1	21	1	1	1	1	0
0	1	1	1	25	1	2	2	1	0

0	1	1	1	19	1	2	2	1	1
0	0	0	0	14	1	1	0	1	0
0	0	0	2	22	1	1	0	1	0
0	0	0	0	15	1	2	2	2	0
0	0	0	0	15	1	2	0	2	0
0	0	0	0	15	1	1	0	2	0
0	0	0	0	17	1	2	0	2	1
0	1	1	1	15	2	2	0	2	1
0	0	0	0	18	1	1	1	1	0
0	0	0	1	15	1	1	1	1	0
0	0	0	0	17	2	1	0	2	0
0	0	0	0	15	1	1	0	1	0
0	0	0	0	16	1	1	1	2	0
0	0	0	0	17	1	1	0	2	0
0	0	0	0	19	1	1	0	1	0
0	0	0	0		1	1	0	1	0
0	0	0	1		1	3	0	1	1
0	0	0	0	15	1	1	0	1	0
1	0	0	0	19	1	1	0	1	0
1	0	0	0	19	1	1	0	1	0
0	0	0	0	19	1	1	0	1	0
0	0	0	3	18	1	2	2	2	1
0	0	0	0	15	1	2	2	2	1
0	0	0	2	28	1	2	4	1	1
0	0	0	0	14	1	2	2	2	1
0	0	0	2	29	1	1	2	2	0
0	0	0	3	37	2	2	1	2	0
0	0	0	1	35	2	2	2	2	1
0	2	1	2	25	1	1	3	2	0
1	0	0	1	17	2	3	4	2	1
0	0	0	1	16	2	2	3	2	1
0	0	0	0	15	1	2	0	2	0
0	0	0	0	14	1	1	1	2	1
0	1	1	3	14	1	1	0	2	0
0	0	0	1	27	2	1	2	2	0
0	0	0	2	39	1	1	2	1	0
0	1	1	1	17	2	2	2	1	0
0	0	0	0	17	1	2	2	2	0
0	0	0	2	21	1	1	2	2	1
0	1	1	1	20	1	1	3	2	0
0	0	0	1	15	1	1	1	2	0
0	1	1	3	20	2	2	2	2	0
0	2	1	0	20	1	1	1	2	0
0	0	0	0	19	1	1	1	2	0
0	0	0	0	22	1	1	2	1	1
0	0	0	2	23	1	1	2	2	0
0	0	0	1	20	1	1	0	2	1

1	1	1		21	1	2	2	2	0
0	0	0	0	16	1	1	3	1	0
0	0	0		16	1	2	0	1	0
0	0	0	0	16	2	3	3	2	1
0	0	0	0	16	2	3	3	2	1
0	2	1	1	24	2	2	3	2	1
1	0	0		19	1	1	2	1	0
0	0	0	0	15	1	1	3	1	0
0	1	1	4	18	2	2	2	2	1
0	2	1	2	14	2	2	0	2	1
0	0	0	1	18	2	2	1	2	1
0	0	0	1	15	2	2	3	2	0
0	0	0	0	16	2	2	1	2	0
0	0	0		15	2	3	0	2	0
0	0	0	0	14	2	2	0	2	0
0	0	0			1	2	0	2	1
0	0	0	1	14	2	2	1	2	0
0	0	0	0	17	1	1	2	2	0
1	0	0	0	33	2	2	1	2	0
0	2	1	1	15	1	2	0	2	0
0	0	0		14	1	2	1	1	0
0	1	1		27	1	1	1	2	0
0	0	0	0	14	1	2	3	2	0
0	0	0	0	18	1	1	2	2	0
0	0	0	0	17	1	1	3	2	0
0	2	1	0	16	1	1	3	1	0
0	0	0		18	1	1	1	1	0
0	0	0	1	14	1	2	1	2	0
0	0	0	1	15	1	1	1	2	0
0	0	0	0	16	1	1	3	2	0
0	2	1	0	14	1	2	1	2	0
0	0	0	0	15	1	1	3	2	0
0	0	0		17	1	1	0	1	1
0	0	0	1	16	2	1	0	2	0
0	2	1	1	18	1	1	0	1	0
0	0	0	3	21	1	2	2	2	0
1	2	1	0	15	1	3	1	2	1
0	0	0	0	14	2	1	0	2	0
0	0	0		16	1	2	0	2	0
0	0	0	2	15	1	2	1	2	1
0	0	0		18	2	2	0	2	0
0	0	0		17	1	1	1	2	0
0	2	1	1	17	2	2	1	2	0
0	0	0	0	17	1	1	3	2	1
0	1	1	3	18	2	2	2	2	0
0	0	0	0	17	1	2	0	1	0
1	0	0	1	14	1	2	1	2	1

0	1	1	5	21	1	1	1	2	0
0	0	0	0	14	1	3	2	2	1
0	0	0	0	17	1	1	0	1	0
0	0	0	0	14	2	1	2	2	0
0	0	0	0	14	2	1	2	2	0
0	0	0	1	16	1	2	2	2	0
0	0	0	1	16	1	2	2	2	0
0	0	0		15	1	3	2	2	1
0	0	0		16	2	2	0	2	0
0	0	0	0	19	1	1	0	2	0
0	0	0		17	1	1	2	2	0
0	0	0			2	2	2	2	0
0	2	1	0	18	1	1	0	1	0
0	2	1	0	22	2	1	2	2	0
0	0	0	1	25	1	1	0	2	0
0	0	0	0	16	1	1	3	2	0
1	0	0		26	1	2	1	2	0
0	2	1		21	1	1	1	2	0
1	2	1	4	18	1	2	2	1	0
1	1	1	0	30	1	1	1	2	0
0	0	0	0	15	1	1	2	1	0
0	0	0	0	14	2	2	1	2	0
0	0	0	0	16	1	2	0	2	0
1	0	0	3	16	1	2	5	1	0
1	0	0	3	18	1	2		1	0
1	2	1	5		1	2		1	0
1	0	0	3	15	1	2	2	1	0
0	0	0	1	17	2	2	2	2	1
0	0	0	1	17	1	2	0	2	1
0	0	0	0	15	2	2	2	2	1
0	0	0	0	15	1	2	2	2	0
0	0	0	0	19	1	2	1	2	1
0	0	0	0	14	1	2	1	2	1
0	0	0	0	14	1	2	1	2	0
0	0	0	0	15	1	2	0	2	0
1	0	0	0		1	1	5	1	1
0	0	0	0	14	1	1	0	2	1
0	0	0	0	18	2	1	1	2	0
0	0	0	0	31	1	1	1	2	1
0	0	0	0	15	1	1	0	1	0
0	0	0	0		1	2	0	2	0
0	2	1	3	31	2	2	3	2	1
0	1	1	3	14	2	2	1	2	1
0	0	0	0	14	1	2	0	2	1
1	0	0	2		1	2	1	1	1
0	0	0	0	16	2	1	2	2	0
0	1	1	2	23	2	2	2	2	1

0	1	1	3	25	1	2	3	1	1
0	0	0	0	14	1	2	4	1	0
0	0	0	2	18	2	2	0	2	0
0	0	0	3	22	1	2	2	2	0
0	0	0	0	27	1	2	1	1	0
1	0	0	2	17	1	2	0	1	0
0	1	1	0	24	1	2	1	2	0
0	0	0	0	14	2	2	0	2	1
0	1	1	0	29	1	2	0	1	0
0	0	0	0	15	1	2	0	2	0
1	1	1	3	16	1	2	1	2	1
0	0	0	1	15	1	2	1	1	0
1	0	0	1	16	1	2	2	1	0
0	0	0	1	26	2	2	1	2	1
0	0	0	0	14	1	2	1	2	0
0	1	1	0	21	2	2	1	2	0
1	0	0	3		1	2	2	2	0
0	1	1	0	25	1	1	1	2	0
1	2	1	3	19	2	2	2	2	1
0	0	0	1	15	1	2	1	2	0
0	0	0	0	19	1	2	1	1	0
0	0	0	1	16	1	2	2	2	1
0	0	0	0	18	1	2	1	2	0
1	0	0	2	15	1	1	0	2	0
0	0	0	0	25	1	2	1	2	0
1	0	0	1	17	1	2	3	1	0
0	1	1	3	19	1	2	2	1	1
0	0	0	1	17	1	2	1	2	0
0	0	0	0	15	1	3	0	2	1
0	2	1	0	14	1	3	1	2	0
0	0	0	2	16	1	2	1	2	0
1	1	1	0	21	1	1	2	2	0
1	0	0	0	14	2	3	2	2	1
0	2	1	3	18	2	3	2	2	1
0	0	0	0	16	1	2	0	2	0
1	0	0	1	23	1	2	1	1	0
0	0	0	1	21	1	1	1	1	0
0	0	0	2	24	1	1	1	2	0
0	1	1	0	27	1	2	3	2	0
0	0	0	2	14	1	1	2	2	0
0	0	0	0	19	2	3	4	2	1
0	1	1		23	1	1	0	1	0
0	1	1	2	23	2	2	4	2	1
0	0	0	0	28	1	1	2	2	0
0	0	0	0	17	2	2	2	2	1
0	0	0	0	14	1	1	0	1	0
0	0	0	0	17	1	2	0	1	0

0	0	0	0	14	1	1	0	1	0
0	0	0	0	14	1	2	0	2	0
0	0	0	1	20	1	1	1	1	0
0	0	0	0	22	1	2	4	1	1
0	0	0	0	14	1	2	0	1	0
0	2	1	0		1	1	0	1	0
0	1	1	0	28	1	2	3	1	0
0	0	0	0	20	2	2	1	2	0
0	0	0	0	26	2	2	1	2	1
0	0	0	0	26	1	2	0	1	0
0	2	1	0	23	1	1	0	1	0
0	2	1	1	26	1	2	0	1	0
0	0	0	1	20	1	1	3	1	1
0	0	0	0	25	2	1	1	2	1
0	0	0	0	19	1	2	4	1	0
0	0	0	0	18	1	2	5	1	0
0	0	0	0	27	1	2	0	1	0
0	1	1	0	23	1	1	4	1	0
0	0	0	0	16	1	2	1	1	0
0	2	1	0	14	1	2	0	1	0
0	0	0	0	14	1	2	1	1	0
0	0	0	0	18	1	1	1	1	1
0	0	0	1	14	2	2	0	2	0
0	0	0	1	14	2	3	1	2	1
0	0	0	0	14	1	2	0	1	0
0	1	1	0	26	1	2	0	1	0
0	0	0	0	16	2	2	1	1	1
0	0	0	2	16	2	1	1	2	0
1	0	0	3	33	2	2	1	1	1
0	1	1	0	16	1	1	1	1	0
0	0	0	0	14	1	1	2	1	0
0	2	1	1	25	2	2	1	2	1
0	2	1	0	22	2	2	1	2	0
0	0	0	0	23	2	2	5	2	1
0	0	0	0	14	2	2	1	2	1
0	0	0	0	24	1	1	4	1	0
0	0	0	0	14	1	2	5	1	0
0	1	1	0	20	2	3	1	2	1
0	0	0	0	27	1	2	4	1	1
0	0	0	0	17	1	2	1	1	0
1	1	1	3	23	1	1	1	1	1
1	0	0	2	16	1	1	0	1	1
1	0	0	0	14	2	2	1	2	0
1	0	0	1	31	1	1	0	2	1
0	1	1	0	16	1	1	0	1	0
0	1	1	0	16	1	1	0	1	0
1	1	1	2		1	1	1	1	0

1	1	1	2		1	1	1	1	0
0	0	0	1	18	1	1	2	1	0
0	0	0	1	18	1	1	2	1	0
0	0	0	0	18	1	1	4	1	0
0	0	0	0	18	1	1	4	1	0
0	2	1	1	22	1	1	1	2	0
0	1	1	0	21	2	3	1	2	1
0	0	0	0	22	1	1	1	1	0
1	2	1	1	26	1	1	0	1	1
0	0	0	0	15	1	2	1	1	0
0	0	0	0	20	1	2	5	2	1
0	1	1	0	15	1	1	4	2	0
0	2	1	1	25	1	2	1	2	0
1	2	1	4	26	1	2	1	2	0
0	0	0	0	17	1	1	3	1	0
0	2	1	0	22	1	1	0	1	1
1	0	0	4	25	1	1	3	1	0
0	0	0	0	15	1	1	2	2	1
0	0	0	0	18	1	2	0	1	0
0	0	0	4	21	1	1	0	1	0
0	0	0	2	21	2	1	4	1	0
1	2	1	2	21	1	1	0	1	0
0	0	0	0	15	1	1	2	2	1
0	0	0	0	17	1	1	0	1	0
0	0	0	0	16	2	2	4	2	0
0	0	0	0	22	1	1	4	1	0
0	0	0	0	16	1	1	0	1	0
0	0	0	0	17	1	1	1	1	0
0	0	0	0	16	1	1	0	2	0
0	0	0	0	15	1	1	1	1	0
0	0	0	0	15	1	1	1	1	0
0	0	0	1	19	1	1	2	2	0
0	0	0	0	16	1	1	3	1	0
0	0	0	0	14	1	1	0	1	0
0	0	0	2	17	2	3	0	1	0
0	0	0	0	14	2	3	0	2	1
0	0	0	0	16	2	3	0	2	1
0	0	0	0	19	1	2	5	1	0
0	2	1	1	18	1	1	1	1	0
0	0	0	0	18	1	1	2	2	0
0	0	0	0	15	1	2	1	2	0
0	0	0	0	19	1	1	0	1	0
0	0	0	0	14	1	2	0	2	0
0	0	0	0	20	1	1	5	1	0
0	0	0	0	24	1	2	3	2	0
0	0	0	0	28	1	2	2	1	0
0	0	0	0	15	1	1	0	1	0

0	0	0	0	15	1	1	0	1	0
0	0	0	0	16	2	1	1	2	1
0	0	0	0	19	1	1	0	1	0
0	0	0	0	17	1	2	1	2	0
0	0	0	0	19	2	1	2	2	0
0	0	0	1	16	2	1	0	2	1
1	0	0	5	15	1	1	4	1	0
0	0	0	0	20	1	1	0	2	0
0	0	0	0	17	2	2	1	2	0
0	1	1	0	24	1	1	1	1	0
0	0	0	0	15	2	2	2	2	0
0	0	0	3	19	1	1	3	1	0
0	0	0	0	19	1	1	2	1	0
0	0	0	0	18	1	2	1	2	0
0	0	0	0	14	1	1	0	1	0
0	0	0	0	15	1	1	1	1	0
0	0	0	0	22	2	1	0	2	0
0	0	0	0	22	2	1	0	2	0
0	0	0	2	19	1	2	2	2	0
1	2	1	0	17	1	1	0	2	0
0	2	1	0	19	1	2	2	1	0
0	0	0	0	16	1	1	2	2	0
0	0	0	0	22	2	2	0	2	0
0	0	0	4	15	1	2	2	2	0
0	0	0	3	28	1	1	0	2	1
1	1	1	1	20	1	2	2	1	0
1	1	1	2	25	1	2	1	1	1
1	0	0	2	19	1	1	1	1	0
0	0	0	1	18	1	1	1	1	1
0	0	0	0	17	1	3	0	2	0
1	0	0	2	16	1	2	3	1	0
0	0	0	0	19	2	1	3	2	0
0	1	1	0	17	1	1	2	1	0
0	0	0	0	17	2	2	4	2	1
0	2	1	0	18	2	2	2	2	0
0	0	0	2	16	1	1	0	1	0
0	0	0	2	16	1	1	0	1	0
0	0	0	1	19	1	1	1	1	0
0	1	1	3	16	1	1	2	1	1
0	1	1	0	25	1	2	3	2	0
0	1	1	1	30	1	1	3	1	0
0	1	1	2	24	1	1	0	1	0
0	0	0	0	16	2	1	0	2	1
0	0	0	0	16	2	1	0	2	1
0	0	0	1	24	1	1	2	1	0
0	2	1	0	14	1	1	0	1	0
0	1	1	0	17	1	1	0	1	1

0	0	0	0	15	1	1	2	1	0
1	1	1	2	19	1	1	0	1	0
0	0	0	2	19	1	1	1	1	0
0	1	1	2	24	1	1	3	2	0
0	2	1	1	23	1	1	2	1	0
0	0	0	0	14	2	2	1	2	1
0	0	0	0	21	2	3	4	2	1
0	0	0	0	15	2	1	0	2	1
0	1	1	2	20	1	2	1	2	0
0	0	0	0	18	1	2	1	1	0
0	2	1	1	15	2	3	2	2	1
0	0	0	0	14	1	1	0	1	0
0	2	1	1	19	2	3	0	2	1
0	0	0	2	22	1	2	4	2	1
0	0	0	0	20	1	1	2	1	0
0	0	0	0	16	2	2	0	2	1
0	0	0	1	24	1	2	2	1	0
0	1	1	1	18	1	2	1	2	1
0	0	0	0	18	1	2	0	2	0
1	1	1	1	22	2	2	3	2	1
1	1	1	0	19	1	1	2	1	0
0	0	0	1	16	1	2	1	1	0
0	1	1	1		2	2	1	2	1
1	2	1	4	19	1	2	2	2	1
0	0	0	0	17	2	2	1	2	0
1	0	0	1	25	1	1	2	1	0
0	0	0	0	23	2	2	2	2	0
0	0	0	1	20	2	3	2	2	1
0	1	1	0	19	2	2	2	2	1
0	0	0	1	20	2	3	2	2	1
0	0	0	1	15	1	2	1	2	0
0	1	1	4	18	2	2	4	2	1
0	0	0	0	15	1	2	1	1	0
0	0	0	0	17	1	1	0	1	1
0	0	0	0	17	2	3	0	2	1
0	0	0	0	14	2	2	0	2	0
0	0	0	2	22	1	1	2	1	0
0	0	0	0	23	1	1	2	1	0
1	1	1	1	27	1	2	3	1	0
0	0	0	0	15	1	2	1	1	0
1	0	0	1	21	1	2	1	1	0
0	2	1	0	14	1	2	1	1	0
0	0	0	1	18	2	2	0	2	1
0	0	0	1	18	2	3	2	2	1
0	0	0	0	18	2	2	2	2	1
1	0	0	1	24	1	1	0	1	0
0	0	0	1	23	1	1	2	1	0

0	2	1	0	19	1	2	2	2	0
0	0	0	1	14	1	1	3	1	0
0	0	0	0	17	1	1	2	1	0
1	1	1	0	38	2	1	3	1	1
0	0	0	0	17	1	1	0	2	0
0	0	0	4	21	1	1	0	1	0
0	0	0	0	14	1	1	0	1	0
1	0	0	0	21	1	1	0	1	0
1	0	0	0	18	1	2	0	1	0
0	0	0	0	20	1	1	0	1	0
0	0	0	0	16	1	1	1	1	0
1	0	0	4	16	2	1	1	2	1
0	0	0	0	14	1	1	1	2	0
0	0	0	0	20	1	1	1	1	0
0	0	0	3	14	1	3	4	2	1
0	0	0	0	16	1	1	0	1	0
0	0	0	0	17	1	1	0	1	0
0	0	0	0	14	1	1	0	2	0
0	0	0	0	17	1	1	0	1	0
0	0	0	0	18	1	1	0	1	0
0	0	0	0	17	1	1	1	1	0
0	0	0	0	16	1	1	0	1	0
0	0	0	2	14	1	1	1	1	0
1	0	0	2	14	1	1	1	1	0
0	0	0	0	17	1	1	0	1	0
0	0	0	0	14	2	2	0	2	1
1	2	1	6	22	1	2	3	2	0
0	0	0	1	19	1	2	1	1	0
1	1	1	4	30	1	1	2	1	1
0	0	0	0	16	1	1	0	2	0
0	0	0	1	22	1	2	2	2	0
0	0	0	0	21	1	2	2	2	1
0	1	1	3	19	1	1	1	1	0
1	0	0	8	24	1	1	5	1	0
0	0	0	2	22	1	2	1	1	0
0	0	0	3	16	1	1	3	1	1
0	0	0	0	19	1	1	0	2	0
1	1	1	0	25	1	1	2	2	0
0	0	0	0	16	1	1	2	1	0
1	0	0	0	14	1	2	1	2	0
0	0	0	1	14	1	3	3	2	0
0	0	0	0	14	1	2	1	2	0
1	0	0			1	1	0	1	0
0	0	0	1	21	2	1	1	2	0
0	0	0		17	1	2	0	2	0
0	1	1		17	1	1	3	2	0
0	0	0	2	17	1	1	3	1	0

1	0	0	2	22	1	1	2	2	0
0	0	0	1	16	1	2	2	1	1
1	0	0	2	23	1	2	5	1	1
0	0	0	1	17	1	1	1	1	0
0	0	0	4	23	2	1	1	2	0
0	1	1		27	1	1	0	1	0
0	0	0	1	16	1	1	4	1	1
0	2	1	1	20	1	1	2	1	1
0	0	0	0	14	2	2	1	2	0
1	0	0	6	23	1	2	3	1	1
0	0	0	0	14	1	1	1	2	0
0	0	0	0	14	1	1	3	1	0
0	0	0	0	17	2	1	2	1	0
0	0	0	0	15	1	1	0	2	0
1	1	1	2	22	2	1	2	1	0
0	0	0	0	14	2	2	0	1	0
0	0	0	0	17	2	2	2	2	0
0	0	0	0	17	1	1	1	2	0
0	0	0		20	1	1	1	1	0
0	1	1	0	19	1	2	3	2	0
0	1	1	1	16	1	1	1	2	0
0	1	1	1	18	2	2	2	2	0
0	1	1	2	16	2	3	1	2	1
0	0	0	1	19	1	1	0	2	0
1	0	0		20	1	1	0	1	0
0	0	0		17	1	2	0	1	0
0	0	0		15	1	2	0	2	0
0	0	0		17	1	1	0	1	0
0	0	0	0	14	1	1	1	1	0
0	0	0		20	2	3	0	2	1
0	0	0	0	19	1	2	3	2	0
0	0	0	1	20	2	2	0	2	0
0	0	0	1	20	2	2	0	2	0
0	0	0	0	15	1	2	0	2	0
0	0	0	0	14	1	2	0	2	1
0	0	0	1	14	1	1	0	2	0
1	1	1	0	18	1	2	0	1	0
0	0	0	0	14	1	2	1	1	1
0	0	0	0	24	1	1	4	1	0
0	0	0	0	17	1	1	3	2	0
0	0	0	0	17	1	1	0	1	0
0	0	0	3	15	2	2	2	2	0
0	0	0	0	19	1	2	0	2	0
0	2	1	1	21	2	2	5	2	1
1	0	0	1	19	1	2	1	2	1
0	1	1	10	24	2	2	5	2	1
0	1	1	0	19	1	1	0	1	0

0	0	0	2	14	1	1	2	2	0
1	1	1	0	27	1	1	0	1	0
0	0	0	1		1	2	0	2	0
0	0	0	1		1	2	0	2	0
0	0	0	1	17	1	1	3	1	0
0	1	1	4	25	2	2	0	2	1
0	0	0	0	20	2	2	1	1	0
0	0	0	0	20	2	2	1	1	0
0	2	1	4	16	2	3	3	2	1
0	2	1	7	31	1	1	1	2	0
0	0	0	0	14	1	2	0	1	0
0	1	1	1	25	1	2	2	1	1
1	1	1	3	27	1	2	2	1	0
1	1	1	2	23	2	3	1	1	1
0	0	0	2	24	1	2	3	2	0
0	0	0	1	15	2	2	2	2	0
0	1	1	0	15	1	1	2	2	0
1	0	0	2	28	1	2	1	2	0
1	0	0	2	28	1	2	1	2	0
0	0	0	2	19	1	2	1	2	0
1	0	0	0	15	2	2	0	2	1
0	0	0	2	14	2	3	3	2	1
0	1	1	1	25	1	2	3	1	1
0	0	0	0	14	2	2	2	2	0
0	0	0	0	17	1	2	1	1	0
0	0	0	0	28	1	2	0	1	0
1	0	0	6	17	2	3	2	2	1
0	0	0	0	14	1	2	0	2	1
0	0	0	3	28	1	1	1	1	0
0	0	0	0	21	1	1	2	2	0
0	2	1	5	20	1	1	1	1	0
0	0	0	3	31	1	1	0	1	1
0	0	0	0	14	2	2	0	2	1
0	0	0	0	14	2	2	1	2	1
0	0	0	0	22	2	2	3	2	0
0	2	1	1	19	1	1	1	2	0
0	2	1	1	24	2	1	3	2	1
0	0	0	1	16	1	2	3	2	1
0	0	0	1	18	1	1	1	2	0
0	0	0	1	28	1	1	0	1	0
0	2	1	0	43	2	1	1	2	0
0	0	0	0	42	2	2	1	2	1
0	0	0	0	25	1	1	0	1	0
0	0	0	1	23	2	2	1	2	0
0	2	1	1	20	1	1	3	1	0
0	0	0	0	19	1	2	5	2	0
0	0	0	0	19	1	2	3	2	0

0	0	0	0	19	2	1	1	2	1
0	1	1	0	18	2	2	1	2	0
0	1	1	0	22	1	2	3	1	0
0	0	0	0	18	1	1	4	1	0
0	1	1	2	29	1	1	1	2	1
0	1	1	0	15	1	1	0	1	1
0	0	0	1	15	2	2	1	2	0
0	0	0	1	15	2	2	1	2	0
1	2	1	1	19	1	3	1	2	1
0	2	1	4	31	1	3	1	2	1
0	0	0	0	18	1	2	0	1	1
1	2	1	1	26	1	1	0	2	0
1	1	1	3	26	1	1	1	2	0
0	0	0	1	15	1	1	0	1	0
0	0	0	0	14	2	2	1	2	0
0	0	0	0	14	1	1	0	2	0
0	0	0	0	14	1	1	0	2	0
0	0	0	0	14	1	1	0	2	0
0	0	0	0	17	1	1	0	1	0
0	0	0	1	16	1	1	0	1	0
0	0	0	0	26	1	2	2	1	0
0	0	0	0	14	1	1	0	1	0
0	0	0	0	18	1	1	0	2	0
0	0	0	0	21	1	2	1	1	0
0	0	0	0	14	1	1	2	1	0
0	0	0	0	24	1	1	3	1	0
0	0	0	1	18	1	1	0	1	0
0	0	0	0	16	1	2	0	1	0
0	0	0	1	15	1	1	0	1	0
1	1	1	1	23	1	1	0	1	0
0	0	0	0	15	1	2	1	1	0
0	0	0	1	16	2	1	0	2	0
0	0	0	0	22	1	1	0	1	0
0	0	0	0	17	1	1	2	1	0
0	0	0	0	21	1	1	1	1	0
0	0	0	0	14	1	2	0	1	0
0	0	0	0	14	1	2	3	1	0
0	0	0	0	14	2	2	0	2	0
0	0	0	0	15	1	1	0	2	0
0	0	0	0	15	1	1	0	2	0
0	0	0	0	14	1	1	0	1	0
0	0	0	0	17	1	1	0	1	0
0	0	0	1	19	1	2	2	2	0
1	1	1	5	16	1	1	3	1	0
0	0	0	1	18	1	1	0	1	0
0	0	0	0	22	1	2	3	1	0
0	2	1	2	21	2	2	3	2	1

0	0	0	3	23	1	1	2	1	1
0	0	0	3	14	2	2	2	2	0
0	0	0	4	18	1	1	2	1	0
0	0	0	2	19	1	1	1	1	0
0	0	0	2	19	1	1	2	2	0
0	0	0	2	22	1	1	0	1	0
0	0	0	0	20	2	2	0	2	0
0	0	0	1	15	1	1		1	0
0	0	0	0	20	2	1	2	2	0
0	0	0	0	17	1	1	0	1	0
0	0	0	0	21	1	1	0	1	0
0	0	0	2	18	1	1	0	2	0
0	2	1	1	14	1	1	0	2	0
0	2	1	0	20	1	1	1	2	0
0	0	0	0	15	2	2	2	2	1
0	0	0	0	20	1	2	1	1	0
0	0	0	0	22	1	1	1	2	0
0	0	0	1	23	2	3	2	2	1
0	0	0	0	24	2	2	1	2	1
1	0	0	0	26	1	1	0	1	0
0	0	0	2	21	2	1	0	2	1
0	0	0	2	15	1	2	2	2	0
1	0	0	1	17	2	2	3	1	0
0	0	0	0	23	1	2	2	2	0
0	0	0	1	33	1	1	2	2	0
0	0	0	1	17	1	2	2	2	0
0	0	0	1	19	1	2	2	1	0
0	0	0	1	20	1	1		1	0
0	0	0	0	14	2	3	0	2	1
0	0	0	1	18	2	2	1	2	0
0	0	0	1	17	2	2	0	2	0
0	0	0	0	22	1	1	2	1	0
0	0	0	0	17	1	2	2	2	1
0	1	1	1	26	2	2	2	2	1
0	0	0	0	18	2	2	1	1	0
0	0	0	0	16	1	1	0	2	0
0	0	0	1	24	2	3	1	2	1
0	0	0	0	19	1	3	1	2	1
0	0	0	1	14	1	1	2	1	1
0	1	1	2	18	1	2	1	2	0
0	0	0	0	20	1	2	2	1	0
0	0	0	2	23	2	2	2	2	0
0	0	0	1	17	2	3	3	2	1
0	0	0	0	15	1	3	2	2	1
0	0	0	0	19	2	1	0	2	0
0	0	0	1	20	1	2	1	2	0
0	0	0	2	24	1	1	2	1	0

0	0	0	1	21	1	1	1	2	0
0	0	0	1	23	2	3	2	2	0
0	2	1	1	24	1	1	2	2	1
0	0	0	1	18	2	1	2	2	0
0	0	0	2	20	2	3	4	2	1
0	0	0	0	20	1	1	1	1	0
0	0	0	0	14	1	1	0	2	0
1	0	0	3	17	1	1	2	1	0
0	0	0	1	15	1	1	1	2	0
0	0	0	0	18	1	1	0	1	0
0	0	0	0	15	1	1	0	1	0
0	0	0	0	15	1	1	0	1	0
0	0	0	1	14	1	1	0	2	0
0	0	0	0	15	1	1	0	1	0
0	0	0	0	14	1	1	0	1	0
0	0	0	0		1	1	0	2	0
0	0	0	3	19	1	2	0	1	0
0	0	0	1	14	1	1	4	2	0
0	0	0	0	15	1	1	4	2	1
0	0	0	2	22	2	1	2	1	1
0	0	0	1	15	1	1	5	1	0
0	1	1	3	22	1	2	0	1	0
0	0	0	1	24	1	2	0	2	0
0	0	0	2	25	1	2		2	0
0	0	0	0	20	1	3	2	2	1
0	2	1	0	18	1	2	1	2	1
1	0	0	1	22	2	2	2	2	1
1	1	1	3		1	1	3	1	0
1	0	0	5	20	1	1	0	2	0
1	1	1	5	23	1	2	2	1	0
0	0	0	2	20	1	1	2	1	0
0	0	0	0	15	1	2	0	2	0
0	0	0	0		1	1	2	1	0
1	1	1	6	32	1	1	5	2	1
0	0	0	0	18	1	1		1	0
0	0	0	0	21	2	1	0	1	0
1	0	0	0	19	2	1	1	2	0
0	0	0	0	17	2	2	1	2	0
0	2	1	2	18	1	2	3	2	0
1	0	0	3	22	1	2	2	2	0
0	0	0	3	15	1	1	0	1	0
0	0	0	4	20	1	1	3	2	0
0	0	0		15	1	1	2	1	0
1	0	0	1	21	2	2	0	2	0
1	0	0	0		1	1	0	2	0
0	0	0	5	21	1	2	5	2	0
0	0	0	5	18	2	2	3	2	1

0	0	0	1	19	1	2	4	1	1
0	1	1	4	17	1	2	2	2	1
0	0	0	2	16	2	1	0	2	0
0	0	0	1	16	1	1	3	1	0
0	0	0	1	17	1	2	2	2	1
0	0	0	0	19	2	2	1	2	0
0	0	0		21	1	2	0	1	1
1	0	0	0	22	2	2	2	2	0
0	0	0	0	18	1	1	1	1	0
0	0	0	0	14	1	1	2	2	0
0	0	0	1	15	1	1	0	2	1
0	0	0		15	1	2	3	2	0
0	0	0	3	21	1	1	2	1	0
1	2	1	0	17	2	1	2	2	0
0	0	0	0	17	2	1	1	2	0
0	0	0	3	24	1	2	1	1	0
0	0	0	1	17	1	1	0	1	0
0	1	1	2	28	1	1	1	1	0
0	0	0	2	14	2	1	1	2	0
0	0	0	0	14	2	2	0	2	1
0	1	1	0	23	2	2	2	2	0
0	0	0	0	23	1	2	1	1	0
0	2	1		22	1	1	2	2	1
0	0	0	0	16	2	1	0	2	0
0	0	0	1	16	1	1	0	2	0
0	0	0	3	15	1	1		1	0
0	0	0	0	15	1	2	1	2	0
0	0	0	1	16	1	2	2	1	0
0	2	1	1	22	1	2		2	0
0	0	0	0	15	1	2	1	2	0
0	0	0	0	14	1	2	3	2	0
0	0	0	0	14	1	2	4	1	0
0	0	0	0	18	1	3	0	2	0
1	0	0	4	15	1	2	5	2	0
0	0	0	0	32	1	1	1	2	0
1	0	0	3	26	1	2	2	2	0
0	0	0	0	14	1	1	0	2	0
0	1	1	0	16	1	2	3	1	0
0	1	1	0	16	1	2	3	1	0
0	0	0	3		1	1	0	2	0
0	0	0	4	38	2	2	5	1	1
0	0	0	0	14	1	2	1	2	0
1	2	1	2	17	1	1	0	1	0
0	0	0	1	29	1	1	0	1	0
1	0	0	0	26	1	1	1	2	0
0	0	0	0	23	1	1	2	1	0
0	2	1	1	22	2	1	2	1	0

0	1	1	1	20	1	2	3	1	1
0	0	0	0	23	2	3	2	2	1
0	0	0	0	23	2	3	2	2	1
0	0	0	0	14	2	2	1	2	1
0	0	0	2		1	1	0	1	0
0	1	1	1	20	1	2	2	1	0
0	0	0	0	21	2	2	2	2	0
0	0	0	6	22	1	1	1	2	0
0	0	0	0	21	1	1		1	0
0	0	0	0	19	1	1	2	2	0
0	1	1	0	14	1	2	0	2	1
0	0	0	0	21	1	1		1	0
0	0	0	0	21	1	1	0	1	0
0	0	0	0	19	1	1	3	2	0
0	0	0	1	23	1	2	3	2	1
0	0	0	0	14	1	2	5	1	1
0	2	1	0	32	2	1	1	2	1
0	0	0	1	20	1	2	5	2	1
0	1	1	0	19	1	1	4	2	0
0	1	1	0	19	1	1	4	2	0
0	2	1	1	18	1	1	0	1	0
0	0	0	0	22	1	3	5	2	1
0	0	0	0	21	1	1	1	2	0
0	0	0	0	14	1	2	5	1	0
0	0	0	1	14	2	1	0	2	1
0	2	1	2	21	1	2	1	2	0
0	0	0	3	26	1	1	1	1	0
0	0	0	0	20	2	2	3	2	1
1	1	1	0	16	1	1	1	1	0
0	0	0	0	24	1	1	3	1	0
0	0	0	0	14	1	1	3	1	0
1	2	1	3	26	1	2	0	2	0
0	0	0	3	15	1	1	0	1	0
1	1	1	3	29	1	1	1	1	1
0	0	0	3	24	1	1	2	1	0
0	0	0	4	17	1	1	2	1	0
0	0	0	1	17	1	1	0	2	0
0	0	0	1	19	1	2	4	2	0
0	0	0	1	17	1	1	0	2	0
0	0	0	0	19	1	1	0	1	0
0	0	0	0	14	1	2	0	1	1
1	0	0	1	17	1	1	2	2	0
0	0	0	0	17	2	1	3	2	0
0	0	0	0	15	1	2	0	1	0
0	0	0	0	18	1	1	1	1	0
0	0	0	0	15	1	1	0	2	0
0	0	0	0	20	1	1	3	1	0

0	0	0	0	14	1	2	2	2	0
0	0	0	0	15	2	1	2	2	0
0	0	0	0	21	1	1	4	2	0
0	0	0	0	23	2	2	0	2	0
0	0	0	0	26	1	1	1	1	0
0	0	0	1	18	1	2	2	2	0
0	0	0	0	17	1	1	1	1	0
0	0	0	0	19	2	2	5	2	0
0	0	0	0	18	1	1	3	1	0
0	0	0	1	14	1	1	0	2	0
0	0	0	1	14	1	1	0	2	0
0	0	0	1	17	1	2	1	2	0
0	0	0	1	17	1	2	1	2	0
1	0	0	2	30	1	1	1	1	0
0	0	0	3	25	1	1	1	1	0
0	0	0	0	17	1	2	2	2	0
1	2	1	0	18	1	1	0	1	0
0	0	0	0	16	1	2	2	1	0
0	0	0	1	19	1	1	2	2	0
0	1	1	4	20	1	1	1	1	0
1	0	0	2	16	1	1	3	1	0
0	0	0	1	15	1	1	3	2	0
0	0	0	3	23	2	1	2	2	0
0	0	0	0	15	2	2	1	2	0
0	0	0	0	18	2	2	2	2	0
0	0	0	1	21	1	1	0	1	0
0	0	0	2	15	1	2	0	1	1
0	0	0	4	16	1	1	1	1	1
0	2	1	5	24	1	1	3	2	0
0	0	0	0	26	2	1	2	2	0
0	0	0	0	20	2	3	2	2	1
0	1	1	1	21	1	1	3	2	0
0	2	1	1	16	1	1	2	1	0
0	0	0	1	17	2	1	0	2	0
0	0	0		15	1	2	0	2	0
0	0	0	0	24	2	1	2	2	0
0	1	1	0	18	2	3	2	2	1
0	0	0	1	15	1	1	0	1	0
0	0	0	0	25	1	1	1	2	0
0	0	0	0	16	2	2	1	2	0
0	0	0	1	16	1	2	2	2	0
0	0	0	0	20	1	1	1	2	0
1	0	0	1	18	1	1	2	1	0
0	0	0	0	19	2	2	1	2	1
0	0	0	0	18	2	3	1	2	1
0	2	1	2	20	1	2	2	1	0
0	0	0	2	17	2	2	1	2	0

0	0	0	1	19	1	2	2	2	1
0	0	0	1	25	1	1	0	1	0
0	2	1	0	16	1	2	1	2	1
0	0	0	1	22	1	1	2	1	0
0	0	0	0	22	2	3	2	2	1
0	0	0	2	26	1	1	0	1	0
0	0	0	1	23	1	1	0	1	0
0	0	0	0	28	1	1	0	1	0
0	0	0	2	26	1	1	0	1	0
0	0	0	0	18	1	1	0	1	0
1	0	0	2	15	1	1	0	2	0
0	0	0	0	14	1	1	0	1	0
0	0	0	0	28	1	1	0	1	0
0	0	0	0	14	2	1	1	2	0
0	0	0	0	18	1	1	0	1	0
0	0	0	0	14	1	1	0	2	0
0	0	0	0	22	1	1	0	1	0
0	0	0	2	14	2	2	1	2	0
0	0	0	0	14	1	1	0	2	0
0	0	0	3	22	2	2	2	1	1
0	0	0	0	17	1	1	0	2	0
0	0	0	0	15	1	1	2	2	0
1	0	0	2	18	1	2	1	2	0
1	0	0	4		1	1	4	1	0
1	1	1	3	40	1	1	3	1	1
0	0	0	4	21	1	1	2	1	1
1	0	0	2	20	1	1	3	1	0
0	0	0	1	19	1	1	0	2	0
0	2	1	2		1	1		2	0
1	0	0	4	17	1	2		2	1
0	2	1	1	23	1	1	0	1	0
1	2	1	5	35	1	1	0	1	0
0	0	0	4	20	1	1	0	2	0
1	2	1	2	20	1	1	4	2	1
0	0	0	2	16	1	1	2	2	0
0	1	1	3	22	1	1	0	2	0
0	0	0	5	23	1	1	3	1	0
0	0	0	0	14	1	2	3	2	0
1	0	0	3	16	1	1	0	2	0
0	0	0	1	14	1	1	0	2	0
0	0	0	1	15	1	2	3	1	1
0	0	0		26	2	2	0	2	0
1	2	1	0	21	1	2	1	2	0
0	0	0	1	25	1	1	3	2	0
1	0	0	3	16	1	1	1	2	0
0	0	0	1	24	2	2	0	2	0
0	0	0	2	15	1	1	2	2	0

0	0	0	1	14	2	2	0	2	0
0	0	0	1		1	1	4	2	0
1	0	0		20	1	1	4	1	0
0	0	0	4	18	1	3	1	2	1
0	0	0	1	16	1	1	0	1	0
0	0	0		19	1	3	2	2	1
0	0	0	3	23	2	1	4	2	0
0	0	0	2	15	1	1	1	1	0
0	0	0	2	20	1	2	1	1	0
0	0	0	0	15	2	2	0	2	0
0	0	0	0	16	1	2	2	1	0
0	0	0	0	21	1	1	1	2	0
0	0	0	1	14	2	1	0	2	0
0	0	0	0	14	1	1	0	2	0
0	0	0	0	16	1	2	0	2	0
0	0	0	0	15	1	1	0	1	0
0	0	0	0	14	2	2	1	2	0
0	0	0	0	14	1	1	2	1	0
1	0	0	1	16	1	1		1	0
0	0	0	0	15	1	1	0	1	1
0	0	0	0	18	1	1	2	1	0
0	0	0	2	18	1	1	0	1	0
0	0	0	1	15	1	2	2	1	0
0	0	0	0	15	2	2	1	1	1
0	0	0	0	26	1	1	1	2	0
0	0	0	0	14	2	2	1	2	0
0	2	1	3	23	2	3	1	2	1
1	0	0	1	24	1	1	3	1	1
0	0	0	0	22	2	3	2	2	0
0	0	0	2	14	2	1	1	2	0
0	0	0	2	24	1	1	1	2	0
0	1	1	0	21	1	1	1	1	0
0	0	0	0	17	1	1	1	1	0
0	0	0	0	17	1	1	1	1	0
0	0	0	0	22	1	1	1	2	0
0	0	0	0	22	1	1	1	2	0
1	0	0	7		1	1	3	1	0
0	0	0	0	15	1	1	0	2	0
1	0	0	1	18	1	1	2	2	0
0	0	0	0	24	1	1	1	1	0
1	1	1	1	15	1	2	3	1	0
0	0	0	1	18	1	1	2	2	0
0	0	0	5	17	2	1	2	2	0
0	0	0	0	14	2	1	2	2	0
0	0	0	2	14	1	1	1	1	0
0	0	0	0	23	2	2	1	2	1
0	0	0	3	19	1	1	1	1	0

0	0	0	0	17	1	1	0	1	0
0	0	0	0	16	1	1	0	1	0
0	0	0	0	14	1	2	1	1	0
0	0	0	0	14	2	1	0	2	0
0	0	0	1	21	1	1	3	2	0
0	2	1	0	17	2	2	1	2	0
0	0	0	0	14	2	3	1	2	1
0	0	0	0	31	2	2	1	2	0
0	0	0	1	16	1	1	1	2	0
0	0	0	0	14	2	1	0	2	1
0	0	0	0		1	3	3	2	0
0	0	0	1	19	1	1	3	2	1
0	0	0	0	21	1	1	5	1	0
0	0	0	0	14	2	1	5	2	1
1	0	0	3	22	1	1	1	2	1
0	0	0	0	15	1	1	0	1	1
1	0	0	0	19	1	1	2	2	0
1	0	0	0	19	1	1	2	2	0
0	0	0	0	17	1	1	2	1	0
1	1	1	2	31	1	1	0	1	0
1	2	1	0	23	1	1	1	1	0
0	0	0	0	19	1	1	1	1	0
1	0	0	3	16	1	1	5	1	0
0	0	0	0	18	1	1	0	1	0
0	0	0	2	17	1	1	0	1	0
0	0	0	0	19	1	1	2	1	0
0	0	0	1	14	1	1	0	1	0
0	0	0	1	16	1	1	1	2	0
0	0	0	0		1	1	0	1	0
0	0	0	0	16	1	1	0	1	0
0	2	1	2	19	1	1	1	1	0
0	0	0	0	19	2	1	0	2	0
0	0	0	0	19	1	1	1	1	0
0	0	0	1	27	1	1	0	1	0
0	0	0	0	17	1	1	1	1	0
0	0	0	0	18	1	1	1	1	0
0	0	0	1	18	2	1	2	2	0
0	0	0	0	16	1	1	0	1	0
1	0	0	0		1	1	2	2	0
0	1	1	1	19	1	1	1	1	0
0	0	0	3	27	2	1	3	2	0
0	0	0	0	21	2	1	0	2	0
0	0	0	0	15	1	1	1	1	0
0	0	0	0	20	1	1	1	2	0
0	0	0	1	14	1	1	1	1	0
0	0	0	0	15	1	1	0	1	0
0	0	0	1	14	1	1	0	1	0

0	0	0	3	16	1	1	2	1	0
0	0	0	4	15	2	1	0	2	0
0	2	1	4	16	2	1	1	2	1
1	0	0	3		1	1	1	2	0
0	1	1	4	20	1	1	4	1	1
0	0	0	0	23	1	1	1	1	0
0	0	0	0	17	1	1	0	1	0
1	2	1	5	26	1	1	0	1	0
1	0	0	6	31	1	1	0	1	1
0	0	0	2	14	2	1	2	2	0
0	0	0	3	20	1	2	4	1	0
0	0	0	5	17	1	1	2	1	0
0	0	0	5	17	1	1	2	1	0
1	0	0	4	20	1	1	2	1	0
1	0	0	1	26	1	1	0	1	0
1	0	0	1	26	1	1	0	1	0
1	0	0	0	23	1	1	2	1	0
1	0	0	7	30	2	1	4	1	1
1	0	0	4	18	2	1	2	2	0
1	0	0	3		1	1	2	1	0
0	0	0	0		1	1	3	2	0
0	0	0	1	14	1	1	2	1	0
0	0	0	1	18	1	1	1	2	0
1	0	0	2	19	1	1	2	2	0
0	0	0	0	16	1	1	2	1	0
0	0	0	3	20	1	1	0	2	0
0	0	0	2	16	1	1	0	1	0
0	0	0	1	19	1	1	0	2	0
0	0	0	1	20	2	1	3	2	0
0	0	0	1	21	2	1	1	2	0
0	0	0	1	16	1	1	2	1	0
0	0	0	1	20	1	1	2	2	0
0	2	1	3	23	1	1	0	1	0
0	0	0	0	15	1	1	0	1	0
0	1	1	1	16	1	1	1	1	0
0	0	0	0	16	1	1	1	1	0
0	0	0	0	17	1	1	0	1	0
0	0	0	2	14	1	1	1	2	0
0	0	0	1	14	1	1	1	2	0
0	0	0	4	27	1	1	1	1	0
0	0	0	3	19	1	2	0	2	0
0	0	0	0	15	1	1	0	1	0
0	0	0	1	14	1	1	1	2	0
0	0	0	1	19	1	1	2	2	0
0	0	0	0	14	2	1	0	2	0
0	0	0	0	14	1	2	0	2	0
0	0	0	0	17	1	1	0	2	0

0	1	1	7	26	1	1		2	0
0	1	1	5	23	1	1	0	2	0
1	1	1	4		1	1	2	1	1
0	0	0	5	16	1	1	1	2	1
0	0	0	3	26	1	1	3	2	1
0	0	0	1	19	1	2	0	2	0
1	1	1	3	26	1	1	0	1	0
0	0	0	0	22	1	1	2	2	0
0	0	0	1	16	1	1	3	2	0
0	0	0	4	14	2	2	1	2	1
1	0	0	4	21	1	1	1	2	0
0	0	0			1	1	0	1	1
0	0	0	0	21	1	1	1	2	0
1	0	0		14	1	1	0	1	0
1	0	0	1	16	1	1	1	2	0
0	0	0	1	18	1	1	2	2	0
0	0	0	1	14	1	1	0	2	0
0	0	0	0	17	2	1	2	2	0
0	0	0	0	20	1	1	0	2	0
0	0	0	0	23	2	1	4	2	1
0	0	0		16	1	1	0	1	0
0	0	0	2	17	1	1	5	1	0
0	0	0	0	14	2	1	0	2	0
0	0	0	1	21	1	1	2	1	0
0	0	0	1	21	1	1	2	1	0
0	0	0	2	18	2	1	1	2	0
0	0	0	1	17	1	1	0	2	0
1	0	0	2	18	2	1	0	2	0
1	1	1	4	23	1	1	0	1	0
0	0	0	2	24	2	1	0	2	0
0	0	0	4	16	2	2	0	2	1
0	0	0	2	19	1	1	1	1	0
0	0	0	1	18	1	1	0	2	0
0	0	0	0	21	2	1	4	2	0
0	0	0	0	21	2	1	4	2	0
1	0	0	4	20	2	1	0	2	0
0	0	0	2		1	1	1	2	0
0	0	0	0	21	1	1	1	2	0
1	2	1		18	1	1	1	2	0
0	0	0	0	30	1	1	0	1	0
0	0	0	0	18	1	1	0	2	0
0	0	0	0		1	1	0	2	0
0	0	0	0	15	1	1	1	2	0
0	0	0	0	14	1	1		2	0
1	0	0	1	16	2	2	3	1	0
0	0	0	0	20	1	2	1	1	0
0	0	0	1	17	1	1	2	2	1

1	0	0	10	22	1	1	1	2	0
0	0	0	1	16	1	1	3	2	0
0	0	0	2	17	1	1	0	1	0
0	0	0	0	27	1	2	1	2	1
0	0	0	0	19	1	1	1	1	0
0	0	0	2	18	2	1	3	2	0
0	0	0	0	21	1	1	1	1	0
0	0	0	2	14	1	1	1	2	0
0	2	1	1	22	1	1	2	2	0
0	0	0	0	15	1	1	0	1	0
0	1	1	0	20	1	1	1	1	1
0	0	0	3	15	2	1	2	2	0
0	0	0	4	14	1	1	2	1	0
0	0	0	0	16	1	1	0	1	0
0	0	0			1	1	2	1	0
0	0	0	0	29	1	1	1	2	1
0	2	1	0	31	1	1	1	1	0
0	2	1	1	19	1	1	2	1	0
0	2	1	0	22	1	2	1	1	1
1	0	0	3	23	2	1	0	1	0
0	0	0	6	14	1	1	3	2	0
0	0	0	0	18	1	1	3	2	0
0	1	1	0	24	1	1	3	1	0
0	0	0	0	16	1	1	3	2	0
1	0	0	3		1	2	3	1	0
0	0	0	1	14	1	1	4	1	0
0	0	0	5	14	1	1	2	1	0
0	0	0	0	24	1	1	2	1	1
0	0	0	3	16	1	1	0	1	0
0	0	0	5	14	1	1	1	1	0
0	0	0	0	17	1	1	2	1	0
0	0	0	1	18	2	1	2	2	1
1	0	0	4	24	1	1	1	2	0
0	0	0	0	29	1	1	0	1	0
0	0	0	0	14	2	1	0	2	0
0	0	0	0	19	1	1	0	1	0
0	0	0	0	17	1	1	0	1	0
0	2	1	0	26	1	2	1	1	0
0	0	0	0	28	1	2	1	1	0
0	0	0	0	15	1	1	0	1	1
1	0	0	0	14	1	2	0	1	0
0	0	0	0	17	2	3	2	2	1
0	0	0	0	14	1	2	5	1	0
0	2	1	0	23	1	1	3	1	0
1	0	0	1		1	1	1	1	0
0	0	0	3	14	2	2	1	2	0
0	0	0	0	14	1	1	1	1	0

0	0	0	9	20	1	1	1	1	0
0	0	0	0	15	1	1	0	1	0
0	0	0	0	14	2	2	2	2	0
0	0	0	2	26	1	1	3	2	0
0	0	0	0	19	1	1	0	1	0
0	0	0	0		1	1	5	1	0
0	2	1	0	16	1	2	5	1	1
0	0	0	0	20	1	1	0	1	0
0	0	0	0	34	2	2	0	2	0
0	0	0	0	34	1	2	0	1	0
0	1	1	0	20	1	2	1	1	0
0	0	0	1	21	1	1	3	1	0
0	0	0		30	1	1	0	2	0
0	2	1	1	14	2	1	1	2	1
0	0	0	0	26	1	2	0	1	0
0	0	0	1	21	1	1	0	2	1
1	0	0	3	29	1	1	2	2	0
0	0	0	0	16	1	1	1	2	0
0	0	0	0	17	1	1	0	2	0
0	0	0	2	22	1	1	1	2	1
0	2	1	1	19	1	1	1	2	1
0	0	0	3	21	1	1	0	2	0
0	0	0	0	25	1	1	1	1	0
0	0	0	0	25	1	1	1	1	0
0	0	0	1	25	1	1	3	1	0
1	1	1	1	24	1	1	1	1	0
0	0	0	0	18	1	1	0	1	0
0	0	0	2	22	1	1	2	2	0
0	0	0	2	23	1	1	0	2	1
0	0	0	0	15	1	1	0	2	0
0	0	0	1	27	1	1	2	1	0
0	0	0	0	22	1	1	2	1	0
0	0	0	0		1	1	0	1	0
1	0	0	0	20	1	1	0	1	0
0	0	0	1	20	2	1	0	2	1
1	0	0	2	15	1	1	1	1	0
1	0	0	2	15	1	1	1	1	0
0	0	0	2	16	2	1	0	2	0
0	0	0	2	16	2	1	0	2	0
0	0	0	1	24	1	1	1	2	1
0	0	0	0	20	1	1	0	2	0
1	0	0	3	17	1	1	1	2	0
1	1	1	4	27	1	1	1	2	1
0	0	0	2	16	1	1	1	1	0
0	0	0	2	16	1	1	1	1	0
0	0	0	0	22	1	1	0	1	0
0	0	0	0	22	1	1	0	1	0

0	0	0	1	17	1	1	0	1	0
0	0	0	0	21	1	1	0	1	0
0	0	0	1	18	1	1	1	1	0
0	0	0	0	18	1	1	0	1	0
0	0	0	1	18	1	1	2	1	0
0	0	0		20	1	1	0	2	0
0	0	0	1	21	1	1	1	1	0
0	0	0	1	17	1	1	2	1	0
0	0	0	1	15	1	1	0	1	0
0	0	0	0	18	1	1	0	1	0
0	0	0	1	19	1	1	0	1	0
0	0	0	1	26	1	1	0	2	0
0	0	0	0	21	1	1	3	2	0
0	0	0	0	17	1	1	2	1	0
0	0	0	1	14	1	1	0	1	0
0	0	0	0	16	1	1	0	2	0
0	0	0	0	14	1	1	0	1	0
0	0	0	0		1	1	2	1	0
0	0	0	0	18	1	1	1	2	0
0	0	0	1		1	1	0	1	0
0	0	0	0	17	1	1	0	1	0
0	0	0	1	15	1	1	1	2	0
0	0	0	1	16	1	1	0	1	0
0	0	0	1	18	1	1	0	1	0
0	0	0	0	14	1	1	0	1	0
0	2	1	1	22	1	1		1	1
0	0	0	1	14	1	1	0	1	0
0	0	0	0	18	1	1	0	2	0
0	0	0	1	18	1	1	2	2	0
0	0	0	1	14	1	1	1	1	0
0	0	0	1	15	1	1	0	2	0
0	0	0	1	17	1	1	1	2	0
0	0	0	0	26	1	1	0	1	0
0	0	0	0	20	1	1	1	1	0
0	0	0	1	14	1	1	1	1	0
1	0	0	3	20	1	1	3	1	0
0	0	0	4	14	1	1	2	1	0
0	0	0	3	17	1	1	2	2	0
0	0	0	3	14	1	1	0	2	0
0	0	0	1	14	1	1	2	1	0
0	0	0	2	15	1	1	1	1	0
0	0	0	1	23	1	1	1	1	0
0	0	0	4	19	1	1	3	2	0
0	0	0	0	14	1	1	0	1	0
0	1	1	3	23	1	1	2	1	0
1	0	0	2	25	1	1	1	1	0
0	0	0	1	24	1	1	2	2	0

0	0	0	3	14	1	1	3	1	0
1	0	0	3	19	2	1	2	2	0
0	0	0	2	21	1	1	0	2	0
0	0	0	3	22	1	1	1	2	0
1	0	0	3	24	1	1	0	1	0
1	0	0	3	24	1	1	0	1	0
0	0	0	0	14	1	1	0	2	0
0	0	0	1	23	1	1	2	1	0
1	0	0	1	17	1	1	1	1	1
1	0	0	3	27	1	1	1	2	0
0	0	0	1	16	1	1	3	1	0
0	0	0	4	20	1	1	2	1	0
0	0	0	1	21	1	1	1	1	0
0	0	0	0	14	2	1	0	2	0
0	0	0	3	14	1	1	2	2	0
0	0	0	2	17	1	1	1	1	0
1	0	0	0	16	1	1	5	1	0
0	0	0	0	19	1	1	0	2	0
0	0	0	0	19	1	1	0	2	0
0	0	0	3	20	1	1	4	2	0
0	0	0	0	15	1	1	4	1	0
0	0	0	0	14	1	1	0	1	0
0	2	1	1	22	1	1	1	1	1
0	0	0	0	18	1	1	1	2	0
0	0	0	5	25	1	1	0	2	0
0	0	0	0	17	1	1	0	1	0
0	0	0	3	15	1	1	5	2	0
0	2	1	1	26	1	1	0	2	0
0	0	0	1	16	1	2	2	1	1
0	0	0	0	22	2	3	1	2	0
0	0	0	2	23	1	1	1	2	0
0	0	0	3	25	1	1	4	1	1
0	0	0	0	18	2	1	1	2	0
0	0	0	2	20	1	1	0	2	0
0	0	0	1	19	1	1	0	1	0
0	0	0			1	1		1	0
0	0	0	0	19	1	1	2	2	0
0	0	0	1	18	1	1	2	2	0
0	0	0	2	20	1	1	0	2	0
0	0	0	0	22	1	1	1	2	0
0	0	0	1	16	1	1	2	1	0
1	1	1	1	16	2	1	3	2	0
0	0	0	0	18	2	1	0	2	0
0	0	0	1	15	2	1	0	2	0
0	0	0	1	22	1	1	2	1	0
0	2	1	1	19	1	1	2	1	0
0	0	0	0	16	1	1	2	2	1

0	0	0	2	20	1	1	2	1	0
0	0	0	0	16	1	1	3	1	0
0	0	0	3	17	1	1	2	1	0
0	0	0	0	15	2	2	0	2	1
1	0	0	0	21	2	1	1	2	0
0	0	0	1	21	2	2	3	2	1
0	0	0	0	18	1	1	2	2	0
1	0	0	1	16	1	1	2	2	0
0	0	0	5	22	1	1	1	1	0
0	0	0	3	22	1	1	1	1	0
0	0	0	2	15	1	1	1	1	0
0	0	0	0	17	1	1	0	2	0
0	0	0	0	17	1	1	0	1	0
0	0	0	1	14	1	1	1	1	0
0	0	0	0	16	1	1	0	1	0
0	0	0	2	17	1	1	1	1	0
1	0	0	2	19	1	1	1	1	0
0	0	0	0	14	1	1	0	1	0
0	0	0	0	22	1	1	0	1	0
1	0	0	2	17	1	1	0	1	0
0	0	0	0	15	1	1	1	1	0
1	0	0	5		1	1	4	2	0
0	0	0	0	16	1	1	0	1	0
0	0	0	0	14	1	1	0	2	0
0	0	0		19	1	1	0	2	0
0	0	0	0	17	1	1	0	2	0
0	0	0	2	15	1	1	1	1	0
0	0	0	0	15	1	1	0	1	0
0	0	0	0	14	1	1	1	2	0
0	0	0	0	18	1	1	0	1	0
0	0	0	0	15	1	1	0	1	0
0	0	0	0	15	1	1	0	1	0
1	0	0	5	14	1	1	1	1	0
0	0	0	0	14	1	1	0	1	0
0	0	0	0	14	1	1	0	2	0
0	0	0	0	18	1	1	0	1	0
1	1	1	5	22	1	1	5	1	0
0	0	0	0	21	1	1	0	1	0
0	0	0	0	17	1	1	0	1	0
0	0	0	0	14	1	1	0	2	0
0	0	0	0	14	1	1	0	1	0
0	0	0	0	21	1	1	0	2	1
0	0	0	2	15	1	1	0	1	1
1	0	0	5	19	1	1	2	1	0
1	0	0	6	25	1	1	2	1	0
1	0	0	3	29	1	1	4	2	1
0	0	0	2	18	1	1	1	2	0

1	0	0	5	34	2	2	2	2	1
1	2	1	3	22	1	1	0	2	0
1	0	0	3	32	1	1	2	2	0
1	0	0	1	16	1	1	0	1	0
1	0	0	1	24	1	2	5	2	1
0	0	0	4	23	2	1	0	2	0
1	0	0	4	20	1	1	2	1	0
0	0	0	0	14	1	2	1	2	0
0	0	0	3	17	1	1	0	1	1
0	0	0	0	14	1	1	0	1	1
0	0	0	2	19	1	1	2	1	0
1	1	1	0	25	1	1	0	1	0
0	0	0	1	14	1	1	0	2	0
1	0	0	0	26	1	1	0	1	0
0	0	0	3		1	1	0	2	0
0	0	0	2	18	1	1	2	1	0
1	0	0	7	26	1	1	1	1	0
0	0	0	0	24	1	1	1	1	0
1	0	0	3		1	1	2	1	0
0	0	0	1	16	1	1	2	2	0
0	0	0	1	18	1	1	0	2	0
0	1	1	0	21	1	1	5	2	0
1	0	0	1	20	1	1	0	2	0
0	0	0	3	18	1	2	4	2	0
1	0	0	4	28	1	1	2	1	0
0	0	0	1	23	1	1	3	1	0
0	0	0	1	16	1	1	3	1	0
1	0	0		23	1	1	1	1	0
0	0	0	1	14	1	1	1	2	0
1	0	0	1	18	2	1	0	2	0
0	0	0		18	1	1	0	2	0
1	0	0	0	14	2	1	1	2	0
0	0	0	1	18	1	1	1	1	0
0	0	0	1	15	1	1	1	2	0
0	0	0	1	18	1	1	1	2	0
0	0	0		14	1	1	2	2	0
0	0	0	2	17	1	1	3	2	0
0	0	0		18	1	1	1	2	0
0	0	0	0	15	2	1	0	2	0
0	0	0	2	18	1	1	1	1	0
0	0	0	2	15	1	1	3	1	0
1	0	0		16	1	1	0	1	0
0	0	0		27	1	1	1	2	0
0	0	0	1	14	1	1	2	2	0
0	0	0	1	14	1	1	2	2	0
0	0	0	1	16	1	1	2	2	0
0	0	0	3	19	1	1	0	2	0

0	0	0	0	21	2	1	0	2	0
0	2	1	2	24	1	1	2	2	0
0	0	0	0	19	1	1	4	1	0
0	0	0	5	15	1	1	5	2	0
0	0	0	1	19	1	1	0	1	0
0	0	0	1	15	1	1	3	1	0
0	0	0		16	1	1	1	1	0
0	0	0	0	17	1	1	1	2	0
0	0	0	2	17	1	1	0	2	0
0	0	0	1	17	2	1	0	2	0
0	0	0	0	18	1	1	0	2	0
0	0	0	0	15	1	1	0	2	0
0	0	0		16	1	1	4	1	0
0	0	0		14	1	1	1	1	0
0	0	0		16	2	1	0	2	0
0	0	0	1	18	1	1	0	1	0
0	0	0		16	1	1	0	2	0
0	0	0	2	15	1	1	2	1	0
0	0	0		14	1	1	2	1	0
0	0	0	1	36	1	1	0	2	0
1	0	0	1	35	1	1	2	1	0
0	0	0	4	18	1	1	0	1	0
0	0	0		17	1	1	3	1	0
0	0	0	1	20	1	1	1	2	0
0	0	0	0	15	1	1	1	2	0
0	2	1	1	15	2	1	1	1	0
0	0	0	0	17	1	1	0	2	0
1	0	0	3	18	1	1	2	1	0
0	0	0	1	17	1	1	2	1	0
0	0	0	1	18	1	1	0	1	0
0	1	1	1	20	1	1	1	2	0
0	0	0	1	22	1	1	1	2	0
0	0	0	4	16	1	1	1	2	0
1	0	0	4	15	2	1	0	2	0
0	0	0	2	17	1	1	0	2	0
0	0	0	3	16	1	2	1	2	0
0	0	0	0		1	1	0	1	0
0	1	1	0	25	1	1	0	1	0
0	0	0	0	19	1	1	0	1	0
0	0	0	0	16	1	1	1	1	0

trauma_ca	anychildca	alcpast12	support	hivrc	disclos
0	0	0	0	0	0
0	0	0	0	0	0
0	0	0	1	0	0
0	1	0	0	0	0
0	1	0	0	0	0
0	0	0	1	0	0
0	1	0	0	0	0
0	0	0	1	0	0
0	1	0	1	0	0
0	1	0	1	0	0
1	1	1	1	0	0
1	1	0	0	1	2
0	1	0	0	0	0
0	1	0	1	0	0
0	1	0	1	0	1
0	1	0	2	0	0
0	1	0	2	0	0
1	1	1	0	0	0
1	1	0	1	0	2
0	1	0	1	0	0
0	1	0	0	0	1
1	1	0	1	0	1
1	1	0	1	0	0
0	1	0	2	0	0
1	1	0	2	0	1
0	1	0	1	0	0
0	1	0	1	0	0
1	1	1	2	0	2
1	1	0	1	0	0
1	1	0	0	0	1
0	1	0	0	0	0
0	1	0	0	0	0
1	1	0	0	0	0
1	1	1	1	0	0
0	1	0	0	0	0
1	1	1	0	0	0
1	1	0	2	0	2
1	1	0	0	0	0
1	1	0	0	0	0
1	1	0	1	0	0
0	1	0	0	0	0
1	1	0	1	0	0
1	1	0	1	0	0
0	1	0	1	0	0
1	1	0	2	0	0
1	1	0	0	0	0

1	1	0	1	0	0
1	1	0	0	0	0
0	1	0	1	0	0
0	1	1	0	0	0
0	0	0	0	0	0
0	1	1	0	0	0
0	1	0	0	0	0
0	1	0	0	0	0
1	1	0	0	0	0
1	1	0	0	0	2
1	0	0	1	0	0
0	1	0	0	0	0
0	1	0	1	0	0
1	1	0	0	0	1
0	1	0	0	0	0
1	1	1	1	0	1
0	1	0	0	0	0
0	1	0	0	0	0
0	1	0	2	0	0
1	1	1	1	0	0
1	1	0	1	0	0
0	1	0	0	0	0
0	0	0	0	0	0
0	1	0	0	0	0
0	1	0	0	0	1
0	0	0	0	0	0
0	1	0	0	0	0
1	1	1	1	0	0
0	1	0	0	0	0
0	1	0	2	0	0
0	1	0	2	0	0
0	1	0	2	0	0
0	1	0	1	0	0
0	1	0	1	0	0
0	1	0	2	0	1
0	0	0	0	0	0
0	0	0	0	0	0
0	1	0	1	0	0
0	0	0	0	0	0
0	0	0	1	0	0
0	0	0	0	0	0
0	0	0	0	0	0
1	1	0	2	0	0
0	1	0	2	1	1
0	1	0	0	0	0
1	0	0	1	0	1
0	1	0	2	0	0

0	1	0	1	0	1
0	0	1	1	0	0
1	1	0	1	0	1
1	1	0	1	0	2
1	1	0	1	0	2
1	1	0	1	0	0
1	1	0	1	0	0
1	1	0	1	0	1
1	1	0	1	0	1
0	1	0	1	0	0
1	1	0	1	0	1
0	1	0	0	0	1
0	1	0	1	0	2
0	1	0	0	0	0
0	1	0	1	0	1
0	1	0	0	0	0
1	1	0	0	0	1
0	1	0	1	0	2
1	1	0	1	0	1
0	0	0	0	0	2
1	1	0	0	0	1
0	1	0	1	0	1
0	1	0	0	0	1
0	1	0	1	0	0
0	1	0	1	0	2
0	1	0	0	0	0
0	1	0	0	0	0
1	1	0	0	0	1
1	1	0	0	0	1
0	1	0	0	0	0
0	1	0	0	0	1
0	1	0	0	0	0
1	1	0	0	0	1
0	1	0	1	0	0
0	1	0	0	0	0
0	1	0	0	0	1
0	0	0	0	0	0
0	1	0	0	0	0
0	0	0	0	0	0
0	1	0	0	0	1
0	1	0	1	0	0
0	1	0	1	0	2
0	1	0	0	0	2
0	1	0	0	0	0
0	0	0	0	0	0
0	1	0	1	0	0
0	1	0	2	1	1

1	1	0	1	0	1
1	1	0	1	0	0
0	1	0	1	0	0
0	1	0	1	0	0
1	1	0	2	0	0
1	1	0	2	0	0
0	1	0	1	0	1
0	1	0	1	0	0
0	0	0	2	0	1
0	0	0	2	0	1
0	1	0	1	0	1
0	1	0	0	0	1
0	1	0	2	0	0
0	1	0	0	0	0
1	1	0	0	0	0
1	0	0	1	0	0
1	0	0	2	0	0
0	1	0	1	0	0
0	1	1	2	0	1
0	1	1	2	0	1
0	1	0	1	0	1
0	1	0	1	0	1
1	1	0	1	0	0
0	1	0	1	0	1
1	1	0	0	0	1
0	1	0	0	0	0
0	1	0	1	0	0
1	1	0	1	0	0
0	1	0	0	0	0
1	1	1	1	0	1
0	1	0	0	0	0
0	1	0	1	0	0
0	1	0	0	0	0
0	1	0	0	0	0
0	0	0	0	0	0
1	1	0	1	0	1
1	1	0	0	0	0
0	1	0	1	0	1
1	1	0	1	0	0
0	1	0	1	0	2
0	1	0	0	0	0
0	1	0	1	0	2
0	1	0	1	0	2
0	1	0	0	0	0
0	1	0	1	0	0
1	1	0	1	0	2
0	1	0	0	0	0

0	1	0	1	0	0
0	1	0	1	0	0
0	1	0	1	0	0
0	1	0	1	0	1
0	1	0	0	0	0
1	1	0	1	0	0
0	1	0	1	0	1
0	1	0	0	0	0
1	1	0	1	0	0
1	0	0	2	0	1
1	1	0	1	0	0
0	1	0	1	0	0
0	1	0	2	0	0
1	1	0	1	0	0
0	0	0	1	0	0
0	1	0	2	0	2
0	1	0	1	0	0
0	1	0	0	0	0
0	1	0	0	0	0
0	1	0	0	0	1
0	1	0	0	0	0
0	1	0	0	0	0
0	1	0	0	0	0
0	1	0	0	0	0
0	1	0	2	0	2
0	1	0	0	0	0
0	0	0	0	0	0
0	0	0	2	0	0
0	1	0	0	0	0
0	1	0	0	0	0
1	1	0	0	0	1
0	1	0	0	0	0
0	1	0	0	0	0
0	1	0	0	0	0
1	1	0	1	0	1
1	1	0	1	0	2
1	1	0	1	0	0
1	1	0	1	0	0
0	1	0	0	0	0
0	1	0	1	0	1
0	0	0	1	0	1
0	1	0	2	0	1
1	1	0	1	0	1
1	1	0	1	0	2
0	1	0	0	0	0
0	1	0	1	0	0
0	1	0	1	0	0

1	1	0	0	0	0
1	1	0	1	0	2
1	1	1	1	0	0
0	0	0	2	0	0
0	0	0	0	0	0
0	1	0	2	0	0
1	1	0	1	0	0
1	1	0	1	0	0
0	1	0	1	0	1
0	1	0	1	0	2
0	1	0	0	0	2
0	1	0	2	0	0
0	0	0	0	0	1
0	1	0	2	0	1
1	0	0	0	0	0
1	1	0	0	0	0
1	1	0	0	0	0
1	1	0	0	0	0
1	1	0	0	0	0
0	1	0	1	0	0
0	0	0	1	0	0
1	1	0	2	0	0
1	1	1	0	0	1
1	1	0	1	0	2
1	1	0	0	0	2
1	1	0	0	0	0
1	1	0	0	0	0
0	1	0	0	0	1
1	1	0	0	0	0
1	1	0	1	0	0
1	1	0	1	0	0
1	1	0	1	0	0
1	1	0	1	0	0
0	1	1	1	0	1
1	1	0	0	0	0
1	1	1	0	0	0
0	1	0	1	0	0
0	1	0	0	0	0
1	1	1	1	0	2
1	1	1	1	0	0
0	1	0	1	0	0
0	1	0	1	0	0
0	1	0	1	0	0
0	1	0	1	0	0
0	0	0	1	0	0
0	1	0	1	0	0
1	1	1	1	0	0

0	1	0	0	0	0
0	1	0	2	0	0
0	0	0	1	0	0
1	1	0	1	0	0
0	0	0	1	0	0
0	1	0	2	0	0
1	1	0	1	0	0
0	0	0	1	0	0
0	1	0	0	0	0
1	1	0	1	0	1
0	0	0	1	0	0
1	1	1	1	0	0
0	1	0	1	0	0
0	1	0	2	0	0
0	1	0	2	0	0
1	1	1	0	0	1
1	1	0	1	0	1
0	0	0	2	0	0
0	1	0	1	0	0
1	1	0	0	0	0
0	1	0	0	0	0
1	1	0	2	0	1
0	1	0	1	0	1
0	1	0	1	0	0
1	1	0	1	0	0
1	1	1	0	0	1
0	1	0	1	0	0
0	1	0	0	0	0
0	1	0	1	0	0
1	1	0	1	0	1
1	1	0	1	0	0
1	1	0	1	0	1
0	1	0	0	0	0
1	1	0	1	0	0
1	1	1	1	0	0
1	1	0	2	0	2
1	1	0	1	0	0
0	1	0	1	1	0
0	1	1	0	0	0
1	1	1	0	0	0
1	1	0	0	0	0
0	1	0	0	0	0
1	0	0	1	0	0
0	1	0	1	0	1
0	1	0	1	0	0
0	1	0	1	0	0
0	1	0	1	0	1

0	1	0	1	0	1
1	1	0	0	0	0
0	1	1	1	0	2
1	1	0	1	0	0
1	1	0	1	0	0
0	1	1	1	0	0
0	1	0	2	0	1
0	1	0	2	0	1
0	1	0	2	0	0
0	1	0	2	0	0
0	1	0	1	0	1
0	1	0	0	0	0
0	1	1	1	0	1
1	1	0	2	0	0
0	1	0	2	0	1
1	1	1	1	0	0
1	1	0	1	0	1
1	1	0	0	0	0
0	1	0	1	0	2
0	1	0	1	0	0
1	0	0	0	0	0
0	1	0	0	0	1
1	1	0	1	0	0
1	1	0	0	0	0
1	1	0	2	0	0
0	1	0	2	0	0
1	1	0	1	0	0
1	1	0	0	0	0
1	1	0	0	0	1
0	1	0	1	0	2
1	1	0	2	0	0
0	1	0	1	0	0
0	0	0	0	0	0
1	1	0	2	0	0
0	1	0	2	0	1
1	1	0	2	0	1
1	1	0	0	0	2
1	0	0	0	0	0
0	0	0	2	0	0
0	1	0	2	0	0
0	1	0	2	0	0
0	0	0	0	0	0
0	1	0	2	0	0
0	1	0	1	1	1
1	1	0	1	0	0
0	1	0	1	0	0
1	0	0	0	0	0

0	1	0	0	0	0
0	0	0	2	0	0
0	1	0	2	0	0
0	0	0	0	0	0
0	1	0	1	0	0
0	0	0	0	0	0
0	0	0	0	0	0
0	0	0	0	0	0
0	1	1	0	0	1
1	1	0	0	0	0
0	1	0	0	0	1
0	1	0	2	0	0
0	1	1	2	0	1
1	1	0	0	0	1
1	1	0	0	0	1
0	1	0	1	0	2
0	1	0	2	0	0
0	0	0	2	0	0
0	1	0	0	0	1
0	1	0	2	0	0
1	1	0	1	0	2
0	1	0	0	0	2
0	1	0	1	0	1
0	1	0	1	0	1
0	1	1	1	0	0
0	1	0	2	0	0
1	1	0	0	0	2
1	1	0	0	0	2
0	1	0	1	0	0
1	1	0	2	0	1
1	1	0	2	0	1
0	0	0	2	0	0
0	1	0	0	0	0
0	1	0	1	0	0
0	0	0	0	0	0
0	1	0	2	0	2
0	1	0	0	0	2
0	1	0	2	0	0
0	1	0	1	0	0
0	1	0	0	0	2
0	1	0	0	0	0
0	1	0	0	0	2
0	1	0	1	0	0
0	1	0	2	0	0
0	1	0	1	0	0
0	1	0	1	0	0
1	1	0	2	0	0

0	1	0	1	0	0
1	1	0	0	0	0
0	1	1	1	0	2
1	1	0	2	0	2
0	1	0	1	0	1
0	1	0	0	0	0
0	1	0	0	0	0
0	1	0	1	0	0
0	1	0	0	0	0
0	1	0	1	0	0
0	1	0	0	0	0
0	0	0	1	0	2
1	1	0	1	0	0
0	1	0	0	0	0
0	1	0	1	0	0
0	0	0	0	0	0
1	1	0	2	0	2
0	1	0	1	0	2
0	1	0	2	0	2
0	1	0	1	0	0
1	1	0	0	0	1
1	1	0	2	0	0
1	1	0	1	0	1
0	1	0	1	0	0
0	1	0	1	0	0
0	1	0	0	0	0
1	1	1	2	0	1
1	1	0	1	0	0
0	1	0	2	0	1
0	1	0	2	1	0
1	1	0	1	0	1
0	1	0	0	0	0
0	1	0	0	0	0
1	1	0	1	0	0
0	1	0	1	0	1
1	1	0	1	0	1
1	1	0	2	0	0
0	1	1	1	0	2
0	1	0	1	0	0
0	1	1	1	0	1
1	1	0	1	0	0
1	1	0	1	0	1
0	1	0	0	0	1
1	1	0	2	0	1
0	1	0	2	0	0
0	1	0	1	0	0
1	1	0	1	0	0

0	1	0	1	0	2
0	1	0	0	0	0
1	1	0	1	0	0
1	1	0	1	0	1
1	1	0	0	1	1
1	1	0	1	0	0
1	1	0	1	0	1
1	1	0	1	0	0
0	1	0	1	0	1
1	1	0	2	0	0
0	1	1	1	0	0
0	1	0	1	0	2
1	1	0	1	0	0
0	1	0	0	0	0
1	1	0	1	0	0
0	1	0	0	0	0
1	1	0	1	0	2
0	1	0	0	0	0
1	1	0	2	0	1
0	1	0	1	0	1
1	1	0	1	0	1
1	1	0	1	0	0
1	1	0	1	0	0
0	1	0	0	0	0
0	1	0	0	0	0
0	1	0	1	0	2
0	1	0	0	0	0
1	1	0	1	0	1
1	1	0	1	0	1
1	1	0	1	0	1
1	1	0	1	0	1
0	1	0	0	0	0
0	1	0	0	0	0
1	1	0	0	0	0
1	1	0	1	0	1
1	1	0	1	0	0
0	1	0	0	0	0
1	1	0	0	0	0
0	1	0	1	0	2
0	1	0	1	0	1
0	1	0	0	0	1
0	1	0	0	0	2
0	1	1	1	0	0
1	1	0	1	0	1
1	1	0	1	0	0
1	1	0	1	1	0
0	1	0	0	0	0

0	1	0	0	0	0
0	1	0	1	0	0
0	1	0	0	0	1
0	1	0	2	0	0
0	1	0	0	0	0
0	1	0	0	0	0
1	0	0	2	0	1
1	1	0	2	0	1
0	1	0	0	0	0
0	0	0	1	0	1
0	1	0	0	0	0
0	1	0	0	0	1
1	1	0	2	0	0
0	1	0	0	0	0
0	1	0	0	0	0
0	1	0	1	0	0
1	1	0	2	0	2
0	1	0	0	0	0
0	1	0	1	0	0
0	0	0	0	0	0
0	1	0	0	0	0
1	1	0	1	1	0
0	1	0	0	0	0
1	1	0	1	0	1
1	1	0	2	0	1
1	1	0	1	0	0
0	1	0	1	0	0
0	1	0	1	0	1
1	1	0	2	0	1
1	1	0	2	0	0
1	1	0	1	0	0
1	1	0	0	0	0
0	1	0	1	0	0
1	1	1	1	0	0
1	1	0	1	0	1
0	1	0	0	0	0
1	1	0	1	0	1
0	1	0	0	0	0
1	1	1	1	0	1
1	1	0	1	0	0
1	1	0	1	0	1
1	1	0	1	0	1
0	1	0	1	0	0
1	1	0	1	0	0
0	1	0	2	0	1
1	1	0	2	0	0
1	1	1	0	0	0

1	1	0	1	1	0
0	1	0	0	0	0
0	1	0	1	0	0
1	1	0	0	0	2
0	1	0	1	0	0
1	1	0	2	0	0
0	1	0	1	0	1
0	1	0	2	0	0
0	1	0	1	0	0
0	1	0	1	0	0
1	1	0	2	0	0
1	1	0	0	0	0
0	1	0	0	0	1
1	1	0	2	0	0
0	1	0	2	0	0
1	1	1	1	0	2
0	1	0	0	0	0
1	0	0	0	0	0
1	1	0	0	0	0
1	1	1	2	0	1
1	1	0	0	0	0
0	1	0	2	0	0
1	0	0	1	0	0
0	1	0	2	0	1
0	1	0	1	0	0
1	1	0	2	0	1
1	1	0	1	0	1
1	1	0	1	0	0
0	1	0	0	0	0
0	0	0	2	0	0
0	1	0	0	0	2
0	1	0	0	0	0
0	0	0	1	0	1
1	1	0	0	0	0
0	1	0	2	0	0
0	0	0	1	0	0
1	1	0	0	0	0
1	1	0	1	0	0
0	1	0	1	1	2
0	1	0	2	0	0
0	1	0	1	0	0
1	1	0	1	0	0
1	1	0	0	0	2
0	1	0	0	0	0
1	1	0	1	0	0
1	0	0	1	0	0
1	1	0	0	0	2

1	1	0	2	0	0
0	1	0	1	0	0
0	1	0	1	0	0
1	1	0	0	0	1
0	1	0	0	0	0
0	0	0	2	0	0
0	1	0	1	0	0
1	1	0	1	0	2
1	1	0	1	0	0
1	1	0	1	0	2
1	1	0	1	0	0
0	1	0	0	0	0
0	0	0	1	0	0
1	1	0	1	0	0
0	1	0	0	0	0
0	1	0	1	0	0
0	0	0	0	0	0
0	1	0	1	1	0
1	1	0	0	0	0
0	0	0	1	0	0
0	1	0	1	0	0
0	0	0	1	0	0
0	1	0	1	0	0
0	1	0	1	0	0
0	1	0	1	0	0
1	1	0	1	0	2
0	0	0	1	0	0
0	1	0	2	0	0
1	1	0	2	1	0
0	1	0	1	0	0
0	1	0	0	0	0
0	1	0	2	0	0
0	1	0	0	0	0
1	1	0	1	0	0
0	0	0	1	0	0
1	1	0	1	0	0
0	1	0	0	0	0
0	1	1	0	0	0
1	1	0	1	0	0
0	0	0	1	0	0
1	1	0	2	0	1
0	1	0	1	0	0
1	1	0	2	0	0
1	1	0	1	0	0
0	1	0	2	0	0
0	1	0	0	0	0
0	1	0	0	0	0

1	1	0	2	0	0
1	1	0	2	0	1
0	1	0	2	0	1
1	1	0	0	0	0
0	1	0	2	0	2
1	1	1	0	0	2
1	1	0	1	0	1
1	1	0	1	0	1
0	1	0	1	0	0
0	1	0	1	0	0
1	1	0	2	0	0
1	1	0	1	0	0
1	1	0	0	0	0
1	1	0	1	0	0
0	0	0	2	0	0
0	1	0	0	0	0
1	1	0	1	0	1
0	1	0	0	0	1
1	1	0	0	0	0
0	1	0	2	0	0
1	1	0	0	0	1
1	1	0	0	0	2
0	0	0	1	0	0
0	1	0	0	0	0
1	1	0	0	0	0
0	1	0	1	0	2
1	0	0	2	0	2
1	1	1	1	0	0
1	1	0	1	0	0
1	1	0	2	0	0
0	1	0	0	0	1
1	1	0	0	0	0
1	1	0	0	0	0
0	1	0	0	0	0
1	1	0	2	0	0
1	1	0	2	0	0
0	0	0	1	0	0
0	0	0	1	0	0
1	1	0	0	0	0
0	1	0	1	0	1
0	0	0	0	0	0
0	1	1	0	0	0
0	1	0	1	0	2
1	1	1	2	0	0
1	1	0	1	0	0
0	1	1	2	0	0
0	1	0	0	0	0

0	1	0	1	0	0
0	0	0	1	0	0
0	1	1	2	0	0
1	1	0	0	0	0
1	1	0	0	0	0
0	1	0	1	1	1
0	1	0	1	1	1
1	1	0	1	0	1
1	1	0	2	0	2
1	1	0	0	0	2
0	1	0	0	1	2
0	1	0	0	1	2
0	1	0	0	0	0
1	1	0	2	0	1
1	1	0	0	0	2
1	1	0	2	0	1
1	1	0	2	0	1
1	1	0	1	0	2
1	1	1	2	0	0
0	1	1	1	0	2
1	1	0	0	0	0
1	1	0	1	0	1
1	1	0	0	0	0
0	1	1	1	0	0
0	1	1	1	0	0
1	1	1	2	0	0
0	1	0	0	0	0
1	1	0	2	0	0
1	1	0	1	0	1
0	0	0	0	0	1
0	1	0	1	0	0
1	1	0	0	0	0
1	1	0	0	0	0
1	1	0	1	0	0
1	0	0	1	0	1
1	1	0	2	0	1
1	1	0	2	0	1
0	1	0	2	0	0
1	1	0	1	0	0
1	1	0	1	0	1
1	1	0	1	0	0
1	1	1	0	1	0
1	1	0	1	0	0
1	1	0	0	0	0
0	1	0	2	0	0
0	1	1	0	0	0
1	1	1	1	0	0

0	0	0	0	0	0
1	1	0	1	0	1
1	1	1	1	0	0
0	0	0	1	0	1
0	1	0	0	0	0
0	0	0	0	0	0
0	1	0	2	0	0
0	1	0	2	1	1
0	1	0	2	0	0
0	1	0	2	0	0
0	0	0	1	0	1
0	1	0	0	0	0
1	1	0	0	0	0
0	0	0	0	1	0
0	0	0	1	0	1
0	1	0	0	0	0
0	1	1	0	0	0
0	1	0	1	0	0
0	1	0	2	0	1
1	1	0	0	0	0
0	1	0	0	0	0
0	1	0	0	0	0
0	0	0	0	0	0
1	1	0	2	0	0
0	0	0	1	0	0
0	1	0	2	0	0
0	0	0	2	0	0
0	0	0	1	0	0
0	0	0	1	0	0
1	1	0	1	0	0
0	1	0	1	0	1
1	1	0	2	0	0
0	1	0	2	0	1
0	1	0	0	0	0
0	1	0	1	1	0
0	1	0	1	0	0
0	1	0	2	0	0
0	1	0	1	0	0
0	1	0	0	0	0
0	0	0	1	0	0
0	0	0	2	0	0
0	1	0	0	0	0
0	0	0	1	0	1
0	1	1	2	0	0
0	1	1	1	1	0
1	0	0	1	0	0
0	1	0	1	0	0

0	1	0	1	0	0
1	1	0	0	1	0
0	1	0	1	0	1
1	0	0	0	0	0
1	1	0	1	0	0
0	1	0	1	0	0
0	1	0	2	0	2
0	1	0	2	1	0
1	1	0	2	0	0
1	1	1	1	0	1
1	1	0	0	0	2
0	1	0	1	0	0
0	1	0	1	0	2
0	1	0	2	0	0
1	1	0	1	0	1
1	1	0	1	0	0
0	0	0	1	0	0
0	1	0	1	0	0
0	1	0	2	0	1
1	1	0	1	0	0
0	1	0	1	0	1
0	1	0	2	0	1
1	1	0	1	0	1
1	1	0	1	0	1
0	1	0	1	0	1
1	1	1	0	0	0
0	1	0	1	0	0
1	1	0	1	0	0
1	1	0	0	0	2
0	1	0	0	0	0
1	1	0	0	0	2
0	1	0	0	0	1
0	1	0	0	0	1
0	1	0	1	0	0
1	1	0	2	0	0
0	1	0	1	0	1
0	1	0	0	0	2
0	1	0	1	0	0
0	1	0	1	0	0
0	1	0	1	0	1
1	1	0	0	0	2
1	1	0	1	0	2
0	1	0	2	0	0
1	1	0	0	0	2
0	0	0	1	0	0
1	1	0	1	0	2
1	1	0	1	0	0

0	1	0	1	0	0
1	1	0	0	0	0
1	1	0	1	0	0
0	1	0	0	0	2
0	0	0	2	0	0
0	1	0	1	0	0
0	0	0	1	0	0
1	1	0	1	1	0
0	1	0	1	0	1
0	0	0	0	0	0
0	1	0	1	0	0
0	1	0	0	0	0
0	1	0	0	0	2
0	1	0	0	0	2
0	1	0	2	0	0
0	1	0	0	0	0
1	1	0	0	0	1
0	1	0	0	0	0
0	1	0	0	0	0
0	1	0	0	0	0
0	1	0	0	0	0
0	1	0	0	0	0
0	1	0	2	0	2
0	1	1	0	0	0
0	1	0	0	0	0
0	1	0	0	0	1
0	1	0	0	0	0
0	0	0	1	0	0
0	1	0	0	0	1
0	1	0	1	0	2
0	0	0	0	0	2
1	1	0	0	0	2
0	0	0	2	0	0
1	1	0	1	0	0
1	1	0	0	0	1
0	0	0	0	0	0
0	1	0	1	0	0
0	1	0	0	0	0
0	0	0	2	0	0
0	1	0	0	0	2
0	1	0	0	0	2
0	1	0	0	0	0
0	0	0	0	0	0
0	1	0	1	0	0
0	1	0	1	0	0
0	0	0	0	0	2
0	1	0	1	0	0

0	1	0	1	0	2
0	1	0	1	0	2
0	1	0	1	0	0
0	1	0	1	0	1
0	1	0	2	0	0
0	1	0	0	0	0
0	1	0	0	0	2
0	1	0	0	0	1
0	1	0	0	0	1
0	1	0	1	0	1
0	1	0	0	0	1
0	1	0	0	0	0
1	0	0	0	0	1
0	1	0	0	0	2
0	1	0	0	0	2
0	1	0	1	0	0
1	1	0	1	0	0
1	1	0	2	0	1
1	1	0	2	0	0
1	1	0	2	0	0
0	1	0	1	0	0
0	1	0	1	0	1
0	1	0	0	0	0
0	1	0	1	0	0
1	1	1	2	0	2
0	1	0	1	0	1
0	1	0	2	0	0
0	1	0	2	0	0
1	1	0	2	0	1
0	1	0	0	0	1
0	1	0	1	0	1
1	1	0	0	0	1
1	1	0	1	1	0
0	1	0	0	0	1
0	1	0	1	1	0
1	1	0	0	0	1
1	0	0	1	0	0
1	1	0	2	0	0
0	1	0	2	0	1
1	1	0	1	0	1
1	1	0	1	0	2
0	1	0	1	0	2
0	1	0	1	0	2
1	1	1	2	0	1
1	1	0	2	0	1
1	1	0	0	0	1
0	1	0	1	0	2

0	1	0	1	0	2
1	1	0	0	0	0
0	1	0	1	0	2
1	1	0	1	1	1
0	1	0	1	0	0
0	1	0	1	0	0
0	1	0	0	0	0
0	1	0	0	0	0
1	1	0	1	0	2
1	1	0	2	0	1
1	1	0	1	0	0
1	1	0	1	0	0
1	1	0	2	0	1
0	1	0	2	0	0
0	1	1	0	1	1
0	1	0	1	0	0
1	1	0	0	0	0
0	1	0	0	0	0
1	1	0	1	0	1
1	1	0	2	0	2
1	1	0	0	1	1
0	1	0	2	0	0
1	1	0	1	0	0
0	1	0	1	0	0
1	1	1	2	1	0
0	1	1	2	0	0
0	1	0	2	0	0
0	0	0	1	0	0
1	1	0	1	0	2
1	1	1	1	0	2
1	1	0	1	0	0
0	1	0	2	1	0
1	1	0	0	0	1
0	1	0	1	0	0
1	1	0	1	0	2
1	1	0	1	0	1
1	1	0	1	0	1
1	1	0	1	0	0
1	1	1	1	0	0
0	1	0	1	0	2
0	1	0	1	0	0
0	1	0	1	0	0
0	1	0	0	0	0
1	1	0	1	0	1
0	1	0	1	0	0
1	1	0	1	0	0
1	1	0	1	0	1

0	1	0	0	0	0
1	1	0	1	0	1
0	1	0	1	0	0
0	1	1	2	0	0
0	1	0	1	0	1
1	1	0	0	0	2
0	1	0	0	0	0
0	1	0	1	0	1
0	1	0	0	0	0
0	1	0	1	0	0
0	1	0	0	0	1
0	1	0	1	1	1
0	1	0	1	0	1
0	1	0	1	0	0
1	1	0	0	0	0
1	1	0	1	0	1
0	1	0	1	0	0
1	1	0	1	0	0
1	1	0	1	0	0
1	0	0	1	0	0
0	1	0	1	0	0
0	1	0	1	0	0
1	1	0	1	0	0
0	1	0	1	0	0
0	1	0	2	0	0
0	1	0	0	1	0
0	1	0	0	0	0
0	1	0	0	0	0
1	1	0	1	0	2
0	1	0	1	1	0
1	1	1	1	0	0
0	1	0	1	0	2
0	1	0	1	0	0
0	1	0	1	0	0
0	1	0	1	0	1
0	1	0	1	0	1
0	1	0	1	0	1
0	1	0	1	0	0
0	1	0	1	0	0
1	1	0	1	0	0
0	1	0	0	0	0
0	1	0	1	0	1
1	1	0	1	0	0
0	1	0	0	1	1
0	1	0	0	0	2
0	1	0	1	0	0
0	1	0	1	0	0

1	1	0	1	0	0
1	1	0	1	0	2
1	1	0	1	0	0
0	0	0	1	0	0
1	1	0	0	1	0
1	1	1	1	0	1
0	1	0	0	0	1
1	1	0	1	0	0
0	1	0	1	0	0
1	1	0	1	0	0
0	1	0	1	0	1
0	1	0	0	0	0
0	1	0	0	0	0
0	1	0	1	0	0
0	0	0	0	0	0
0	0	0	1	0	1
0	0	0	0	0	0
0	0	0	0	0	0
0	0	0	0	0	0
0	1	0	0	0	0
0	1	0	0	0	0
1	1	1	0	1	0
1	1	0	2	0	1
0	1	0	0	1	0
0	1	0	0	0	0
0	1	0	0	0	0
0	0	0	1	0	0
0	1	0	1	0	0
0	0	0	1	0	1
0	0	0	0	0	0
0	0	0	0	0	0
0	1	0	0	0	0
0	0	0	1	0	1
0	1	0	1	1	2
0	1	0	0	0	1
1	0	0	1	1	0
0	0	0	1	1	0
0	1	0	0	1	0
1	1	0	1	0	2
0	1	0	2	0	0
0	1	0	0	0	0
1	1	0	1	0	0
1	1	0	1	0	1
0	1	0	1	0	0
1	1	0	2	1	0
1	1	0	1	0	0
0	1	0	0	0	0

1	1	0	0	0	0
1	1	0	0	0	2
1	1	0	0	0	0
0	1	0	1	0	0
1	1	1	0	1	0
0	1	0	0	0	0
1	1	0	0	1	1
0	1	0	0	0	0
0	1	0	1	0	0
1	1	0	1	0	0
1	1	0	1	0	2
0	1	0	1	0	0
0	0	0	2	0	1
1	1	0	0	0	0
1	1	0	0	0	0
0	1	0	1	0	0
0	1	0	1	0	0
1	1	1	0	0	1
1	1	1	0	0	0
0	1	0	2	0	0
0	1	0	1	0	2
1	1	0	2	0	1
1	1	0	2	0	0
0	1	0	1	0	0
1	1	0	1	0	0
1	1	0	1	0	1
0	1	0	1	0	0
0	1	0	1	0	0
1	1	0	1	0	0
1	1	0	1	0	0
0	1	0	1	0	0
0	1	0	2	0	0
1	0	0	1	0	0
1	1	0	0	0	0
1	0	0	0	0	0
1	0	0	0	0	0
1	1	0	1	0	0
1	1	0	0	0	0
1	1	0	2	0	0
1	0	0	2	0	0
0	0	0	1	0	0
0	0	0	1	0	0
0	1	0	0	0	0
0	1	0	0	0	0
1	1	0	1	0	2
0	1	0	2	0	0
0	0	0	2	0	0

0	1	0	1	0	1
0	1	0	1	0	0
0	1	0	2	0	2
0	1	0	2	0	1
1	1	0	1	0	0
1	1	0	1	0	0
1	1	0	2	0	0
0	1	0	2	0	0
1	1	0	1	0	0
0	1	0	0	0	0
1	1	0	2	0	1
1	1	0	2	0	2
0	1	0	0	0	0
1	1	0	0	0	1
1	1	0	1	0	0
1	1	0	2	0	1
0	1	0	1	0	0
0	1	0	2	0	2
0	1	0	1	0	0
1	1	0	1	0	1
1	1	0	1	0	1
1	1	0	1	0	0
0	1	0	0	0	0
1	1	0	0	1	0
0	1	1	1	0	1
0	0	0	2	0	0
1	1	0	1	0	2
1	1	0	0	0	2
1	1	0	1	0	0
0	1	0	2	0	0
0	1	0	2	0	0
1	1	0	2	0	1
0	1	0	0	0	1
1	1	0	2	0	0
0	1	0	1	0	0
1	1	0	0	0	2
1	0	0	0	0	0
1	1	0	0	0	1
1	1	0	2	0	0
1	1	0	1	0	2
0	1	0	0	0	1
1	1	0	2	0	0
1	1	1	2	0	0
0	1	0	2	0	0
1	1	0	0	0	0
0	1	0	0	0	0
1	1	0	2	0	0

0	1	0	1	0	0
0	1	0	1	0	0
0	1	0	1	0	0
0	0	0	1	0	0
0	1	0	0	0	0
1	1	0	0	0	0
1	1	1	2	0	2
0	1	0	0	1	0
0	1	0	1	0	0
1	1	0	1	0	0
1	1	0	1	1	0
0	1	0	0	0	0
0	1	1	1	0	0
0	1	0	1	0	0
1	1	0	0	1	0
0	1	0	1	0	2
0	1	0	2	0	0
0	0	1	2	0	0
0	1	0	1	0	2
0	1	0	0	0	0
0	0	0	1	0	0
1	1	0	2	0	0
0	1	1	0	0	0
1	0	0	0	0	1
0	0	0	0	0	0
0	1	0	1	0	0
0	1	0	2	0	1
1	1	0	1	0	0
1	1	0	0	0	0
1	1	0	0	0	0
1	1	0	1	0	0
1	1	0	0	0	2
0	0	0	0	0	0
1	1	0	0	0	0
1	1	0	1	0	0
1	1	0	1	0	1
0	1	0	1	0	0
0	1	0	1	0	0
0	1	0	0	0	0
1	1	0	0	0	0
1	1	0	1	0	0
0	0	0	0	0	0
1	1	0	1	0	1
0	1	0	0	0	0
0	1	1	2	0	0
0	1	1	2	0	0
0	1	0	2	0	0

0	1	0	2	0	0
1	1	0	0	0	0
1	1	0	1	0	1
0	0	0	1	0	0
0	1	1	2	0	0
1	1	0	1	1	0
1	1	0	2	0	0
0	1	0	1	0	0
0	1	0	1	0	0
1	1	1	1	0	0
1	1	0	1	0	0
1	1	0	1	0	0
1	1	0	0	0	0
1	1	0	1	0	0
1	1	0	1	0	0
1	1	0	1	0	2
0	1	0	2	0	0
0	0	0	0	0	0
0	1	0	1	0	0
0	1	0	1	0	0
1	1	0	0	0	1
0	1	0	1	0	0
1	1	0	0	0	0
0	1	0	1	0	0
1	1	0	1	0	2
1	1	0	1	0	1
0	1	0	0	0	0
0	1	0	1	0	0
0	1	0	1	0	0
0	1	0	1	0	0
1	1	0	0	0	0
1	1	1	0	1	1
1	1	1	1	0	1
1	1	0	1	0	0
1	1	0	1	0	0
1	1	0	0	0	2
1	1	1	1	0	2
0	1	0	1	1	0
0	1	0	2	0	1
0	1	0	2	0	2
1	1	0	1	0	0
1	1	0	2	0	0
0	1	0	1	0	0
1	1	0	2	0	1
1	1	0	2	0	1
1	1	0	2	0	0
1	1	0	2	0	0

1	1	0	0	0	0
1	1	1	0	0	0
1	1	1	0	0	1
1	1	0	2	0	1
0	1	0	0	0	1
0	1	0	0	0	1
0	1	0	2	0	2
0	1	0	2	0	2
0	1	0	2	0	2
1	1	0	1	0	0
1	1	0	1	0	0
0	0	0	0	0	0
1	1	0	1	0	2
0	0	0	1	0	0
1	1	0	0	0	0
1	0	0	1	0	0
0	1	0	1	0	1
0	0	0	1	0	0
0	0	0	1	0	0
0	1	0	2	0	1
1	1	0	1	0	0
1	1	0	1	0	0
0	1	0	1	0	0
1	1	0	1	0	1
1	1	0	2	0	0
0	0	0	0	0	0
0	0	0	1	0	0
1	1	0	1	1	0
0	1	0	1	0	0
0	1	0	0	0	1
0	0	0	0	0	0
0	0	0	0	0	0
0	1	0	1	0	1
1	1	0	2	0	1
0	0	0	0	0	0
0	1	0	2	0	1
0	1	0	2	0	0
0	0	0	1	0	1
1	1	0	1	0	0
0	0	0	0	0	0
0	1	0	2	0	0
0	0	0	0	0	2
0	0	0	1	1	1
0	1	0	2	0	1
0	1	0	0	0	1
0	1	0	2	0	0
0	1	0	0	0	0

0	0	0	2	0	0
1	1	0	0	0	1
0	1	0	0	0	0
0	0	0	0	0	0
0	1	0	0	0	0
0	1	0	2	0	0
0	0	0	2	0	0
1	0	0	2	0	0
0	1	0	2	0	0
0	1	1	2	0	1
1	1	0	2	0	1
0	1	0	2	0	0
0	1	0	2	0	0
1	0	0	0	0	0
0	0	0	2	0	0
0	0	0	0	0	1
1	1	0	2	0	2
0	1	0	2	0	1
0	0	0	0	0	0
0	0	0	0	0	0
1	1	0	2	0	0
0	0	0	0	0	0
1	1	1	2	0	2
0	1	0	2	0	0
0	1	0	0	0	0
0	1	0	0	0	0
0	1	0	2	0	0
0	1	0	2	0	0
0	0	0	2	0	0
0	1	0	1	0	0
1	1	0	0	0	0
0	1	0	1	0	0
0	1	0	2	0	0
0	0	0	0	0	0
0	1	0	2	0	0
0	1	0	0	0	1
0	1	0	2	0	0
0	0	0	2	0	0
0	0	0	2	0	0
0	1	0	2	0	1
0	0	0	2	0	1
1	1	0	2	0	2
1	1	0	2	0	0
1	1	0	2	0	2
0	1	0	1	0	0
0	1	0	0	0	1
0	1	0	2	0	2

0	1	0	1	0	1
1	1	1	2	0	0
0	1	0	1	0	2
0	1	0	1	0	0
1	1	1	1	0	0
1	1	0	1	1	2
1	1	0	1	0	2
0	0	0	2	0	0
1	1	0	1	0	2
0	1	0	0	0	0
1	1	0	0	0	1
1	1	0	0	0	1
1	1	1	2	0	2
1	1	1	2	0	2
0	1	0	2	0	2
1	1	0	1	0	1
1	1	0	2	0	2
1	1	0	2	0	2
1	1	0	1	0	0
1	1	0	1	0	0
0	1	0	2	0	2
1	1	0	0	0	2
0	1	0	0	0	0
1	1	0	1	0	1
0	1	0	2	0	0
0	1	0	1	0	2
0	1	0	2	0	0
0	1	0	2	0	1
1	1	0	0	0	1
1	1	0	0	0	1
1	1	0	2	0	2
1	1	0	2	0	1
1	1	0	2	0	1
0	1	0	1	0	2
1	1	0	2	0	0
1	1	0	1	0	2
1	1	0	2	0	0
1	1	0	0	0	1
1	1	0	1	0	2
0	1	0	1	0	0
0	1	0	1	0	0
1	1	0	2	0	0
0	1	0	0	0	0
1	1	1	1	0	0
0	1	0	1	0	0
0	1	1	1	0	0
1	1	0	2	0	0

0	1	0	1	0	0
0	0	0	0	0	0
0	1	0	2	0	0
0	1	0	2	0	0
0	1	0	1	0	0
0	1	0	2	0	0
1	1	0	0	0	0
0	0	0	0	0	0
0	1	0	1	0	0
0	1	0	1	0	0
0	1	0	1	0	0
0	1	0	1	0	0
0	0	0	1	0	0
0	1	0	2	0	1
0	1	1	0	0	2
0	1	1	0	0	2
0	1	0	2	0	2
0	1	0	0	0	0
0	1	0	0	0	0
0	1	0	0	0	0
0	0	0	0	0	0
1	1	0	0	0	0
0	1	0	1	0	0
1	1	0	0	0	0
0	1	0	1	0	0
0	1	0	1	0	0
0	1	0	2	0	0
0	1	0	1	0	0
0	1	0	0	0	0
0	1	0	2	0	0
0	0	0	0	0	0
0	1	1	2	1	0
0	1	0	1	0	0
0	0	0	1	0	0
1	1	0	2	1	0
1	1	0	1	0	0
0	1	0	2	0	0
1	1	0	1	0	0
1	1	0	1	0	0
1	1	0	1	0	0
1	1	0	2	0	1
0	1	1	2	0	0
1	1	1	2	0	1
1	1	0	2	0	0
1	1	0	0	0	0
1	1	0	0	0	0
0	0	0	1	0	0

1	1	1	2	0	1
1	1	0	1	0	0
1	1	1	0	0	0
0	1	0	0	0	0
1	1	0	0	1	1
0	1	0	0	0	1
1	1	0	1	0	0
1	1	1	2	1	0
1	1	0	0	1	1
1	1	1	1	0	2
1	1	0	0	0	2
0	1	1	1	0	0
1	1	1	2	0	1
1	0	0	1	0	1
1	1	1	2	0	2
1	0	0	1	0	0
0	1	0	2	0	0
1	1	0	0	0	2
0	0	0	1	0	0
1	1	0	1	0	0
1	1	0	1	0	1
1	1	0	2	0	0
1	1	0	2	0	0
1	1	0	2	0	0
1	1	0	2	0	0
0	1	0	1	0	0
1	1	0	1	0	2
1	1	0	1	0	2
1	1	0	0	0	2
1	1	0	0	0	2
0	1	0	1	0	1
0	1	0	1	0	1
0	1	0	1	0	1
0	1	0	0	0	0
0	0	1	1	0	0
0	1	0	2	0	0
0	1	0	0	0	1
0	1	0	1	0	0
0	1	0	1	0	0
0	1	0	1	0	1
0	1	1	2	0	1
0	1	0	1	0	1
0	1	0	1	0	1
0	1	0	0	0	0
1	1	0	1	0	0
0	1	0	0	0	0
1	1	0	0	0	0

0	1	1	1	0	1
0	1	1	1	0	2
0	1	0	1	1	2
1	1	0	2	0	0
1	1	0	1	0	0
1	1	0	0	0	1
1	1	0	1	0	0
1	1	0	1	0	2
0	1	0	0	1	2
1	1	0	1	0	1
0	1	0	1	0	0
0	1	0	0	0	0
0	1	0	0	0	0
0	1	0	2	0	0
1	1	0	1	0	2
0	1	0	1	1	0
1	1	0	1	0	0
0	1	1	1	0	2
1	1	0	1	0	0
1	1	0	2	0	0
1	1	0	2	0	0
1	1	0	1	0	0
1	0	0	1	0	0
0	1	0	1	0	0
0	1	0	1	0	1
0	0	0	0	0	0
1	0	1	0	0	0
0	0	0	1	0	0
1	1	0	1	0	1
0	0	0	0	0	0
0	1	0	1	0	0
1	1	0	1	0	1
0	1	0	1	1	0
1	1	0	1	1	0
0	1	0	1	0	1
1	1	0	1	0	0
0	1	1	1	0	0
0	0	0	0	0	0
0	1	0	2	0	0
0	1	0	1	0	0
0	1	1	2	0	0
0	1	0	1	0	0
1	1	0	1	0	0
1	1	0	1	0	0
0	1	0	0	0	0
1	1	1	1	0	0
1	1	1	1	0	1

1	1	0	2	0	2
0	0	0	0	0	0
1	1	0	0	1	0
0	1	0	1	0	0
0	1	0	0	0	0
0	1	0	0	0	0
0	1	0	0	0	0
1	1	1	0	0	1
0	1	0	1	0	0
1	1	0	1	0	0
0	1	0	0	0	0
0	1	0	0	0	0
0	1	0	1	0	0
0	1	0	0	0	0
0	1	0	0	0	0
0	1	0	0	0	0
1	1	0	0	0	0
0	1	0	0	0	0
0	1	0	0	1	0
0	1	0	0	1	0
0	1	0	0	1	0
1	1	0	1	0	0
0	1	0	2	1	0
1	1	0	2	1	0
0	0	0	1	0	0
1	1	0	1	1	0
1	1	0	1	0	0
1	1	1	1	0	0
1	1	0	2	1	2
1	1	0	2	0	0
1	1	0	2	0	0
0	1	0	0	0	0
0	0	0	1	0	0
1	0	0	0	1	2
1	1	0	1	0	0
1	1	0	1	0	0
1	1	0	1	0	2
0	1	0	1	0	0
1	1	0	1	0	0
1	1	0	2	0	2
1	1	0	1	0	0
1	1	0	1	0	2
0	1	0	1	1	2
0	1	0	1	0	0
0	1	0	1	1	0
1	1	0	1	0	0
1	1	0	0	0	0

1	1	0	2	0	2
0	1	0	2	0	0
1	1	0	0	0	0
0	1	0	2	0	0
0	1	0	2	0	0
1	1	0	2	0	1
1	1	0	1	0	0
0	1	0	2	0	0
1	1	0	1	0	1
1	0	0	0	0	2
1	1	0	1	0	0
1	1	0	2	0	0
0	1	0	1	0	0
1	1	0	0	0	0
0	0	0	0	0	0
1	1	1	0	0	0
1	0	0	1	0	0
0	1	0	1	0	0
0	1	0	1	1	0
1	1	0	0	1	1
1	0	0	1	0	0
1	1	0	1	0	1
0	0	0	2	0	0
0	1	0	2	0	0
0	1	0	2	0	0
0	1	0	2	0	1
1	1	0	1	0	0
1	0	0	1	0	0
1	1	0	1	0	0
0	1	0	2	0	0
0	0	0	1	0	1
0	1	0	2	0	0
1	1	0	0	0	0
1	1	0	0	0	0
1	1	0	0	0	1
1	1	0	2	0	0
0	1	0	1	0	1
0	0	0	0	0	0
1	1	0	0	0	0
1	1	0	1	0	0
1	1	0	0	0	0
1	1	0	1	0	0
1	1	0	1	0	1
0	1	0	2	0	0
1	1	0	1	0	1
0	1	0	0	0	0
1	0	0	1	0	0

1	1	0	1	0	1
0	0	0	1	0	0
0	1	0	0	0	0
0	0	0	1	0	0
0	0	0	1	0	0
1	1	0	1	0	0
1	1	0	1	0	0
1	1	0	2	0	0
1	1	0	0	0	0
0	1	0	0	0	0
1	1	0	1	0	0
1	1	0	1	0	0
0	1	0	0	0	2
0	1	0	2	0	2
1	1	0	0	0	0
0	1	0	2	0	0
1	1	0	1	0	0
1	1	0	1	0	1
1	1	0	1	0	1
0	1	0	1	0	2
0	1	0	1	1	0
0	0	0	1	0	0
0	1	0	0	0	0
1	1	0	2	0	0
1	1	0	2	0	0
1	1	0	1	1	2
1	1	0	2	1	0
1	1	0	1	0	0
1	1	0	0	0	0
0	1	0	1	0	0
0	1	0	1	0	0
0	1	0	1	0	0
0	0	0	1	0	0
0	0	0	1	0	0
0	1	0	0	0	0
0	1	0	2	1	0
0	0	0	0	0	0
0	1	0	1	0	0
0	1	0	1	1	0
0	1	0	0	0	0
0	1	0	0	0	0
1	1	0	2	0	1
1	0	0	1	0	2
0	0	0	0	0	0
1	1	0	1	0	0
0	1	0	2	0	0
1	1	0	1	0	1

1	1	1	2	0	2
0	0	0	2	0	0
1	1	0	0	0	0
1	1	0	1	0	0
0	1	0	1	0	0
1	1	0	0	0	0
0	1	0	1	0	1
0	0	0	0	0	0
0	1	1	0	0	1
0	1	0	0	0	0
1	1	1	1	0	2
1	1	0	1	0	0
1	1	0	1	0	0
1	1	0	1	0	0
0	0	0	1	0	0
0	1	0	1	0	2
1	1	1	1	0	0
0	1	0	1	0	2
1	1	0	2	0	1
1	1	0	1	0	0
0	1	0	1	0	0
1	1	0	1	0	0
0	1	1	1	0	0
1	1	0	0	0	0
0	1	0	1	0	0
1	1	0	2	1	0
1	1	1	2	1	2
1	1	0	1	0	0
0	1	0	0	0	0
0	0	0	1	0	2
1	1	0	1	0	0
0	1	0	1	0	1
0	0	0	2	0	0
1	1	1	1	0	1
0	1	0	0	0	0
1	1	0	1	0	0
1	1	0	1	0	0
1	1	0	1	0	0
0	1	0	2	0	2
1	0	0	2	0	0
0	1	0	2	0	0
1	1	0	0	0	2
1	1	0	2	0	1
0	1	0	1	0	0
0	1	0	2	0	0
0	0	0	0	0	0
0	1	0	0	0	0

0	0	0	0	0	0
0	0	0	0	0	0
1	1	0	1	0	0
0	1	0	2	0	0
0	0	0	0	0	0
0	1	0	0	0	1
0	1	0	2	0	2
0	1	0	1	0	0
0	1	0	1	0	0
0	1	0	0	0	0
0	1	1	0	0	1
1	1	0	0	0	1
1	1	0	2	0	0
0	1	1	1	0	0
0	1	0	2	0	0
0	1	0	2	0	0
0	1	0	0	0	0
0	1	0	2	0	1
0	1	0	1	0	0
0	0	0	0	0	1
0	0	0	1	0	0
0	1	0	1	0	0
1	0	1	0	0	0
1	0	0	1	0	0
0	0	0	0	0	0
0	1	0	0	0	1
0	1	0	1	0	0
1	1	0	1	0	0
1	1	0	1	1	0
0	1	0	1	0	1
0	0	0	2	0	0
1	1	1	1	0	1
0	1	0	1	0	1
0	1	0	2	0	0
0	0	0	1	0	0
0	1	0	2	0	0
0	0	0	2	0	0
0	1	0	1	0	2
0	1	0	2	0	0
0	1	0	1	0	0
1	1	0	1	0	1
1	1	1	0	0	0
0	0	0	1	0	0
1	1	0	0	0	0
0	1	0	0	0	1
0	1	0	0	0	1
1	1	1	1	1	2

1	1	1	1	1	2
1	1	0	2	1	0
1	1	0	2	1	0
0	1	1	2	0	0
0	1	1	2	0	0
1	1	0	1	0	1
0	1	0	1	0	1
0	1	0	1	0	0
1	1	0	0	0	1
0	1	0	1	0	0
0	1	0	2	0	0
0	1	0	2	0	1
1	1	0	1	0	1
1	1	0	1	0	2
0	1	0	2	0	0
0	1	0	0	0	2
1	1	0	2	0	0
0	1	0	2	0	0
0	1	0	0	0	0
1	1	0	0	1	0
1	1	0	2	0	0
1	1	0	0	0	1
0	1	0	1	0	0
0	1	0	0	0	0
0	1	0	2	0	0
0	1	0	2	0	0
0	1	0	0	0	0
0	1	0	1	0	0
0	1	0	0	0	0
0	1	0	1	0	0
0	1	0	1	0	0
1	1	0	1	0	0
0	1	0	2	0	0
0	0	0	0	0	0
1	1	0	0	0	0
0	0	0	0	0	0
0	1	0	0	0	0
0	1	0	2	0	0
1	1	0	1	0	2
0	1	0	1	0	0
0	1	0	1	0	0
0	1	0	0	0	0
0	0	0	0	0	0
0	1	0	2	0	0
0	1	0	2	0	0
0	1	0	1	0	0
0	1	0	0	0	0

0	1	0	0	0	0
0	1	0	1	0	0
0	1	0	0	0	0
0	1	0	1	0	0
0	1	0	1	0	0
1	1	0	0	0	0
1	1	0	2	0	0
0	1	0	0	0	0
0	1	0	1	0	0
0	1	0	1	0	2
0	1	0	1	0	0
1	1	0	2	1	0
0	1	0	1	0	0
0	1	0	1	0	0
0	0	0	0	0	0
0	1	0	1	0	0
0	1	0	0	0	0
0	1	0	0	0	0
1	1	0	1	0	0
0	1	0	0	0	1
0	1	0	1	0	2
0	1	0	2	0	0
0	1	0	0	0	0
1	1	0	2	0	0
1	1	0	0	0	0
1	1	0	1	0	1
1	1	0	1	0	1
1	1	1	1	0	0
1	1	1	1	0	0
0	1	0	0	0	0
1	1	0	2	0	0
0	1	0	2	1	0
0	1	0	1	0	1
0	1	0	2	0	0
0	1	0	1	0	1
1	1	0	0	0	0
1	1	0	0	0	0
1	1	0	1	1	0
1	1	0	1	0	1
0	1	0	2	0	1
1	1	0	2	0	2
1	1	0	0	0	2
0	1	0	0	0	0
0	1	0	0	0	0
1	1	0	2	0	0
0	0	0	0	0	1
0	1	0	0	0	1

0	1	0	1	0	0
1	1	0	0	0	2
1	1	0	1	0	0
1	1	0	2	1	2
1	1	0	1	0	2
0	0	0	1	0	0
0	1	0	2	0	0
0	1	0	0	0	0
1	1	0	1	0	1
0	1	0	1	0	0
1	1	1	1	0	1
0	0	0	0	0	0
1	1	0	0	0	1
1	1	0	2	0	0
0	1	0	1	0	0
0	1	0	0	0	0
1	1	0	1	0	0
1	1	0	1	0	1
0	1	0	0	0	0
1	1	0	2	0	2
0	1	0	1	1	1
1	1	0	1	0	0
1	1	0	1	0	2
1	1	0	1	0	2
0	1	0	1	1	0
1	1	0	1	1	0
0	1	0	1	0	0
1	1	0	1	1	0
0	1	0	1	0	1
1	1	0	2	0	0
1	1	0	1	0	0
1	1	0	2	1	2
0	1	0	1	0	0
0	1	0	0	0	0
0	1	1	0	0	0
0	0	0	0	0	0
1	1	0	1	0	0
0	1	0	1	0	0
1	1	0	2	0	1
0	1	0	1	0	0
1	1	0	1	1	0
0	0	0	1	0	2
1	1	0	0	0	0
1	1	0	2	0	0
0	1	0	1	0	0
1	1	0	0	1	0
1	1	0	1	0	0

0	1	0	2	0	1
1	0	0	2	1	0
0	1	0	1	1	0
0	1	0	2	0	2
0	1	0	0	0	0
1	1	0	0	0	0
0	0	0	0	0	0
0	1	0	0	0	0
0	1	0	0	1	0
0	1	0	0	0	0
0	1	0	1	0	0
1	1	0	1	0	0
0	0	1	1	0	0
0	1	0	1	0	0
1	0	0	2	0	0
0	1	0	0	0	0
0	1	0	0	0	0
0	0	0	0	0	0
0	1	0	0	0	0
0	1	0	0	0	0
0	1	0	1	1	0
0	1	0	0	0	0
1	0	0	1	1	0
1	0	0	1	1	0
0	1	0	0	1	0
0	0	0	0	0	0
1	1	0	2	0	1
1	1	0	1	0	0
1	1	0	2	0	1
0	1	0	0	0	0
1	1	0	1	0	0
0	1	0	2	0	0
1	1	0	1	0	2
1	1	0	2	1	0
1	1	0	1	1	0
1	1	0	2	0	0
0	1	0	0	0	0
0	1	0	1	1	2
0	1	0	1	1	0
0	0	0	1	0	0
1	0	0	2	0	0
0	0	0	1	0	0
1	1	0	0	0	0
1	1	0	1	0	0
1	1	0	0	0	0
1	1	1	2	0	2
1	1	0	2	0	0

1	1	0	1	0	0
1	1	1	1	0	0
1	1	0	2	0	0
1	1	0	1	0	0
1	1	0	1	0	0
1	1	1	0	0	1
1	1	0	2	1	0
1	1	0	2	0	1
0	0	0	1	0	0
1	1	0	2	0	0
0	0	0	1	0	0
0	0	0	2	0	0
0	1	0	1	0	0
0	1	0	0	0	0
1	1	0	1	0	1
0	0	0	0	0	0
0	1	0	2	0	0
0	1	0	1	0	0
1	1	0	1	0	0
0	1	0	2	0	1
1	1	0	1	0	1
1	1	0	1	0	1
1	1	0	1	0	1
1	1	0	0	0	0
1	1	0	0	0	0
1	1	0	0	0	0
1	1	0	0	0	0
1	1	0	0	0	0
0	0	0	1	0	0
1	1	0	0	0	0
0	1	0	2	0	0
1	1	0	0	0	0
1	1	0	0	0	0
0	1	0	0	0	0
0	0	0	0	0	0
1	0	1	0	0	0
0	1	0	0	0	2
0	0	0	1	0	0
0	1	0	2	0	0
0	1	0	2	0	0
0	1	0	0	0	0
1	1	0	1	0	0
0	1	0	0	0	0
1	1	1	2	0	1
1	1	0	1	0	0
1	1	1	2	1	2
0	1	0	0	0	1

1	0	0	2	0	0
0	1	0	0	0	2
1	1	0	0	0	0
1	1	0	0	0	0
1	1	0	2	1	0
1	1	0	0	0	2
0	1	1	1	1	0
0	1	1	1	1	0
1	1	1	2	0	1
1	1	1	1	0	1
0	0	0	0	0	0
1	1	0	2	0	1
1	1	1	2	1	2
1	1	0	1	0	2
1	1	1	2	0	0
1	1	0	1	0	0
0	1	0	2	0	1
1	1	0	1	0	0
1	1	0	1	0	0
1	1	0	1	0	0
0	1	0	0	0	0
1	0	1	1	0	0
1	1	1	2	0	2
0	0	0	2	0	0
0	1	0	1	0	0
0	1	0	0	0	0
1	1	0	2	0	0
0	0	0	0	0	0
1	1	1	1	0	0
0	1	0	2	1	0
1	1	1	1	0	2
1	1	0	0	0	0
0	0	0	0	0	0
0	0	0	1	0	0
0	1	0	2	0	0
1	1	0	1	0	1
1	1	0	2	0	1
1	1	0	2	0	0
1	1	0	1	0	0
1	1	0	0	0	0
0	1	1	1	0	1
0	1	1	1	0	0
0	1	0	0	0	0
1	1	0	1	0	0
1	1	0	2	0	1
0	1	0	2	1	0
0	1	0	2	0	0

0	1	0	1	0	0
0	1	0	1	1	1
0	1	0	2	0	1
0	1	0	2	0	0
1	1	0	1	0	1
0	1	0	0	0	2
1	1	0	1	0	0
1	1	0	1	0	0
1	1	0	1	0	1
1	1	0	1	0	2
0	1	0	0	0	0
1	1	0	0	0	1
1	1	0	1	0	2
1	1	0	0	0	0
0	0	0	1	0	0
0	0	0	0	0	0
0	0	0	0	0	0
0	0	0	0	0	0
0	1	0	0	0	0
1	1	0	0	0	0
0	1	0	2	0	0
0	0	0	0	0	0
0	1	0	0	0	0
0	1	0	1	0	0
0	0	0	2	0	0
0	1	0	2	0	0
1	1	0	0	0	0
0	1	0	0	0	0
1	1	0	0	0	0
1	1	0	0	0	1
0	1	0	1	0	0
1	1	0	0	0	0
0	1	0	0	0	0
0	1	0	1	0	0
0	1	0	1	0	0
0	0	0	0	0	0
0	0	0	2	0	0
0	0	0	0	0	0
0	1	0	0	0	0
0	1	0	0	0	0
0	0	1	0	0	0
0	1	1	0	0	0
1	1	0	1	0	0
1	1	0	2	0	2
1	1	0	0	0	0
0	1	0	2	0	0
1	1	0	2	0	2

1	1	0	1	0	0
1	0	0	1	0	0
1	1	0	1	0	0
1	1	0	1	0	0
1	1	0	1	0	0
1	1	0	0	0	0
0	1	0	0	0	0
1	1	0	0	0	0
0	1	0	1	0	0
0	1	0	0	0	0
0	1	1	0	0	0
1	1	0	0	1	0
1	0	0	0	0	2
0	1	0	1	0	1
0	1	0	2	0	0
0	1	0	1	0	0
0	1	0	1	0	0
1	1	0	1	0	0
0	1	0	1	0	0
0	1	0	0	1	0
1	1	1	0	0	0
1	1	0	1	0	0
1	1	0	2	0	0
0	1	0	1	0	0
1	1	1	1	1	0
1	1	0	1	0	0
1	1	0	1	0	0
1	1	0	1	0	0
0	0	0	0	0	0
1	1	0	1	0	0
1	1	0	0	0	0
0	1	0	1	1	0
0	1	0	2	0	0
1	1	0	2	0	1
0	1	0	1	0	0
0	1	0	0	0	0
1	1	0	1	0	0
0	1	0	1	0	0
1	0	0	1	0	0
1	1	0	1	1	1
0	1	0	1	0	0
1	1	0	1	0	0
1	1	0	2	0	0
0	1	0	1	0	0
0	1	1	0	0	0
1	1	0	1	0	0
1	1	0	2	0	0

1	1	0	1	0	0
1	1	0	1	0	0
1	1	0	1	0	1
1	1	0	2	1	0
1	1	0	2	0	0
0	1	0	1	0	0
0	0	0	0	0	0
1	1	0	2	0	0
1	1	0	1	0	0
0	1	0	0	0	0
0	1	0	0	0	0
0	1	0	0	0	0
1	0	0	0	0	0
0	1	0	0	0	0
0	0	0	0	0	0
0	1	0	0	1	0
1	1	0	0	0	0
1	0	0	2	0	0
0	1	0	2	0	0
1	1	0	1	0	0
1	1	0	2	1	0
1	1	0	0	0	2
1	1	0	0	0	0
1	1	0	1	0	0
0	1	0	2	0	0
0	1	0	1	0	1
1	1	0	1	0	0
1	1	0	2	1	2
1	1	0	0	0	0
1	1	0	1	1	2
1	1	0	2	0	0
0	1	0	0	0	0
0	1	0	1	0	0
1	1	0	2	1	1
0	1	0	1	0	0
0	1	0	0	0	0
0	1	0	1	0	0
0	1	0	1	0	0
1	1	0	2	0	1
1	1	0	1	0	0
1	1	0	0	0	0
1	1	0	2	0	0
1	1	0	1	0	0
1	1	0	0	0	0
0	1	0	0	0	0
1	1	0	2	0	0
1	1	0	2	0	0

1	1	0	2	0	0
1	1	1	1	0	2
1	1	0	0	0	0
1	1	0	2	0	0
1	1	0	1	0	0
0	1	0	1	0	0
1	1	0	0	0	0
0	1	0	1	0	0
0	1	0	1	1	0
0	0	0	2	0	0
1	1	0	0	0	0
1	1	0	2	0	0
1	1	0	1	0	0
0	1	0	1	0	1
0	1	0	1	0	0
1	1	0	1	0	0
1	1	0	0	0	0
1	1	0	1	0	2
1	0	0	1	0	0
0	0	0	0	0	0
0	1	0	1	0	1
0	1	0	1	1	0
1	1	0	1	0	1
0	1	0	0	0	0
1	1	0	0	0	0
1	1	0	2	1	0
0	1	0	1	0	0
1	1	0	2	0	0
1	1	1	0	0	1
0	1	0	1	0	0
0	0	0	2	1	0
0	0	1	2	0	0
0	1	0	0	0	0
1	1	0	2	0	0
0	1	0	1	0	0
1	1	1	1	0	0
0	0	0	0	0	0
0	1	1	2	0	1
0	1	1	2	0	1
1	1	0	0	0	0
1	1	1	2	0	0
0	0	0	1	0	0
1	1	1	0	1	2
1	1	0	0	1	0
0	1	0	1	0	0
0	1	1	1	1	0
1	1	0	1	0	1

1	1	0	2	0	1
0	1	0	2	0	0
0	1	0	2	0	0
0	0	0	1	0	0
1	1	1	0	1	0
1	1	0	1	0	1
0	1	0	1	0	0
1	1	0	1	0	0
0	1	0	0	0	0
0	1	0	2	0	0
0	0	0	0	0	1
0	1	0	0	0	0
0	1	0	0	0	0
0	1	0	2	0	0
1	1	1	2	0	0
0	0	0	2	0	0
0	1	0	1	0	1
1	1	0	2	0	0
0	1	0	2	0	1
0	1	0	2	0	1
1	1	0	0	0	1
0	1	0	2	0	0
0	1	1	1	1	0
0	0	1	2	0	0
1	0	0	0	0	0
1	1	0	1	0	1
1	1	0	1	0	0
0	1	0	2	0	0
0	1	0	1	0	2
0	1	0	2	0	0
0	0	0	2	0	0
1	1	0	0	0	1
1	1	0	0	1	0
1	1	0	1	0	1
1	1	0	2	0	0
1	1	0	1	0	0
1	1	0	0	0	0
1	1	0	2	0	0
1	1	0	0	0	0
0	1	0	0	0	0
0	0	0	0	0	0
1	1	0	2	0	0
0	1	0	2	0	0
0	1	0	0	1	0
0	1	0	1	0	0
0	1	0	0	0	0
0	1	0	2	0	0

0	0	0	2	0	0
0	1	0	2	0	0
0	1	0	2	0	0
0	1	0	0	0	0
0	1	0	1	0	0
1	1	0	1	0	0
0	1	0	1	0	0
0	1	0	2	0	0
0	1	0	2	0	0
1	0	0	0	0	0
1	0	0	0	0	0
1	1	0	1	0	0
1	1	0	1	0	0
1	1	1	1	0	0
1	1	0	1	0	0
0	1	0	2	0	0
0	1	0	0	0	2
0	1	0	1	0	0
1	1	0	1	0	0
1	1	0	1	0	2
1	1	0	2	0	0
1	1	0	2	0	0
1	1	0	1	0	0
0	1	0	1	0	0
0	1	0	1	0	0
1	1	0	0	1	0
1	1	0	0	0	0
1	1	0	1	0	0
1	1	0	2	0	2
0	1	0	2	0	0
0	1	0	1	0	0
1	1	0	2	0	2
1	1	0	1	1	1
1	1	0	0	0	0
1	1	0	0	1	0
0	1	0	1	0	0
0	1	0	2	0	1
1	1	0	0	0	0
0	1	0	1	0	0
0	1	0	1	0	0
1	1	0	2	1	0
0	1	0	1	0	0
1	1	0	1	0	0
0	1	0	1	0	0
0	1	0	1	0	0
1	1	0	1	0	1
1	1	0	1	0	0

1	1	0	1	0	0
1	1	0	0	0	0
0	1	0	1	0	2
1	1	0	1	1	0
0	1	0	1	0	0
1	1	0	0	0	0
1	1	0	0	0	0
0	1	0	0	0	0
1	1	0	0	0	0
0	1	0	0	0	0
1	1	0	0	0	0
0	0	0	0	0	0
0	1	0	0	0	0
0	0	0	1	0	0
0	1	1	0	0	0
0	0	0	0	0	0
0	1	0	0	0	0
1	0	0	1	0	0
0	0	0	0	0	0
1	1	0	2	0	0
0	1	0	0	1	0
0	1	0	2	0	0
1	1	0	1	0	0
1	1	0	2	0	0
1	1	0	2	0	2
1	1	0	1	1	0
1	1	0	2	1	0
1	1	0	0	0	0
1	1	1	2	0	1
1	1	0	0	0	0
1	1	0	0	0	1
1	1	0	0	0	1
1	1	0	0	0	0
1	1	1	2	0	1
1	1	0	1	0	0
1	1	0	0	1	1
1	1	0	2	1	0
0	0	0	2	0	0
1	1	0	0	0	0
1	0	0	0	0	0
1	1	0	2	0	0
1	1	0	0	0	0
0	1	0	1	0	1
1	1	0	2	0	0
1	1	0	1	0	0
1	1	0	0	0	0
1	1	0	1	0	0

1	0	0	0	0	0
1	1	0	2	0	0
1	1	0	2	0	0
1	1	0	1	0	0
1	1	0	0	0	0
1	1	0	1	0	0
1	1	0	2	0	0
1	1	0	1	0	0
1	1	1	1	0	0
0	1	0	0	0	0
0	1	0	2	0	0
0	1	0	1	0	0
1	0	0	0	0	0
0	0	0	0	0	0
0	1	0	0	0	0
0	1	0	0	0	0
0	0	1	1	0	0
0	0	0	1	0	0
1	1	0	2	1	0
0	1	0	0	0	0
0	1	0	1	0	0
1	1	0	0	0	0
1	1	0	1	0	0
0	1	0	1	0	0
0	1	0	1	0	0
0	0	0	1	1	0
1	1	0	1	0	1
1	1	0	1	0	0
0	1	0	1	0	0
1	0	1	1	0	0
1	1	0	1	0	0
0	1	0	1	0	2
0	1	0	1	0	0
0	1	0	1	0	0
0	1	0	1	0	0
0	1	0	1	0	0
1	1	0	1	0	0
0	1	0	0	0	0
1	1	0	2	0	0
0	1	0	1	0	0
1	1	0	2	0	2
1	1	0	1	0	0
1	1	0	1	0	0
0	0	0	1	0	0
1	0	0	1	0	0
0	1	0	1	0	0
1	1	0	1	0	0

0	1	0	0	0	0
0	1	1	0	0	0
0	0	0	1	0	0
0	0	0	0	0	0
1	1	0	2	0	0
0	1	0	1	0	1
0	0	0	1	0	0
0	1	0	1	0	0
1	1	0	1	0	0
0	0	0	0	0	0
0	1	0	2	0	0
1	1	1	2	0	0
0	1	0	2	0	0
0	0	0	2	0	0
1	1	0	1	1	0
0	1	0	0	0	0
0	1	0	2	0	0
0	1	0	2	0	0
0	1	0	2	0	0
1	1	0	0	0	2
0	1	0	1	0	1
0	1	0	1	0	0
1	1	0	2	0	0
0	1	0	0	0	0
1	1	0	0	0	0
0	1	0	1	0	0
1	0	0	0	0	0
1	1	0	1	0	0
0	1	0	0	0	0
0	1	0	0	0	0
1	1	0	1	0	1
0	1	0	0	0	0
0	1	0	1	0	0
1	1	0	0	0	0
0	1	1	1	0	0
0	1	0	1	0	0
1	1	0	1	0	0
0	1	0	0	0	0
0	1	0	2	0	0
1	1	0	1	0	2
1	1	0	2	0	0
0	1	0	0	0	0
0	1	0	1	0	0
0	1	0	1	0	0
1	0	0	1	0	0
0	1	0	0	0	0
1	0	0	0	0	0

1	1	0	2	0	0
1	1	0	0	0	0
1	1	0	1	0	1
1	1	0	1	0	0
1	1	1	2	0	1
0	1	0	1	0	0
0	1	0	0	0	0
1	1	0	0	1	1
1	1	1	0	1	0
1	0	0	1	0	0
1	1	0	2	0	0
1	1	0	1	0	0
1	1	0	1	0	0
1	1	1	2	0	0
1	1	0	0	0	0
1	1	0	0	0	0
0	1	0	2	0	0
1	1	0	2	0	0
1	1	0	1	0	0
1	1	0	1	0	0
0	1	0	2	0	0
1	0	0	1	0	0
1	1	0	1	1	0
1	1	0	2	0	0
0	1	0	1	0	0
1	1	0	0	0	0
1	1	0	0	0	0
1	1	0	0	0	0
1	1	0	2	0	0
1	1	0	1	0	0
1	1	0	1	0	0
1	1	0	1	0	0
1	1	0	0	0	1
0	1	0	0	0	0
1	1	1	1	0	1
0	1	0	1	0	0
0	1	0	0	0	0
1	0	0	1	0	0
1	0	0	1	0	0
1	1	0	1	0	0
1	1	0	0	0	0
0	1	0	0	0	0
1	0	0	1	0	0
1	1	0	2	0	0
0	0	0	0	0	0
0	0	0	0	0	0
0	1	0	0	0	0

1	1	0	2	0	2
1	1	0	0	0	2
1	1	1	1	0	1
1	1	0	1	0	0
1	1	0	2	1	0
1	1	0	0	0	0
1	1	0	0	0	1
0	1	0	1	0	0
1	1	0	2	0	0
1	0	0	1	0	0
1	1	0	1	0	0
1	1	0	0	0	0
0	1	0	1	0	0
1	0	0	0	0	0
1	1	0	1	0	0
1	1	0	2	0	0
1	0	0	0	0	0
0	1	0	1	0	0
0	1	0	0	0	0
0	1	0	2	0	0
1	1	1	0	0	0
1	1	0	2	0	0
0	0	0	0	0	0
1	1	1	2	0	0
1	1	1	2	0	0
1	1	0	1	0	0
1	1	0	0	0	0
1	1	0	0	1	0
1	1	0	0	0	2
1	1	0	0	0	0
1	1	0	0	0	0
1	1	0	1	0	0
1	1	0	0	0	0
0	1	0	2	0	0
0	1	0	2	0	0
1	1	0	0	0	0
1	1	1	1	0	0
0	1	0	1	0	0
1	1	0	1	0	1
0	1	0	0	0	0
0	1	0	0	0	0
0	1	0	0	0	0
0	1	0	1	0	0
0	0	0	0	0	0
1	1	0	2	1	0
0	1	0	1	0	0
1	1	0	1	1	0

1	1	0	1	0	0
1	1	0	2	0	0
1	1	0	0	0	0
0	1	0	1	0	0
0	1	0	1	0	0
1	1	0	2	0	0
0	1	0	1	0	0
1	0	0	1	0	0
1	1	0	1	0	1
0	1	0	0	0	0
0	1	0	1	0	1
1	1	0	1	0	0
1	0	0	1	0	0
0	1	0	0	0	0
1	1	0	1	0	0
0	1	0	1	0	0
0	1	0	1	0	1
1	1	0	2	0	1
0	1	0	1	0	2
1	1	0	0	0	0
1	0	0	2	0	0
0	1	0	2	0	0
0	1	0	2	0	1
0	1	0	2	0	0
1	1	0	2	0	0
1	0	0	2	0	0
1	0	0	1	0	0
0	1	0	1	0	0
1	1	0	0	0	0
1	0	0	1	0	0
0	1	0	2	0	0
1	1	0	2	0	0
1	1	0	1	0	0
0	1	1	0	0	0
0	0	0	0	0	0
0	1	0	0	0	0
0	1	0	0	0	0
0	1	0	1	0	1
0	1	0	1	0	0
0	1	0	0	0	0
0	0	0	0	0	0
0	1	0	2	0	0
0	0	0	2	0	0
0	1	0	2	0	1
1	1	0	1	0	0
1	0	0	1	0	0
0	0	0	1	1	0

1	1	0	1	0	0
0	1	0	0	0	0
0	0	0	2	0	0
1	1	0	2	0	0
0	1	0	0	0	0
0	1	0	2	0	0
0	1	0	2	0	1
0	1	0	0	0	0
0	1	0	0	0	0
0	1	0	0	0	0
0	1	0	1	1	2
1	1	0	2	0	0
1	1	0	0	0	0
1	0	1	1	0	1
0	1	1	0	0	0
1	1	0	0	0	0
1	1	0	1	0	0
0	1	0	1	0	0
0	1	0	0	0	0
1	1	0	1	0	0
1	1	0	1	0	1
1	1	0	0	0	0
0	1	1	1	0	0
0	1	1	1	0	0
1	1	0	2	0	0
1	1	1	1	1	1
0	1	0	0	0	0
1	1	0	1	0	0
1	1	0	0	0	0
0	1	0	0	0	0
1	1	0	1	0	0
0	1	0	2	0	0
0	1	0	0	0	0
0	1	0	0	0	0
1	1	0	0	0	0
1	1	0	1	0	0
1	1	0	1	0	0
1	1	0	0	0	0
1	1	0	0	0	0
1	1	0	1	0	0
0	1	0	0	0	0
1	1	1	1	0	0
1	1	0	1	0	2
1	1	0	1	0	0
1	1	0	1	0	0
0	1	1	0	0	0
0	1	1	0	0	0

1	1	0	0	0	0
0	1	0	0	0	0
1	1	1	1	0	0
0	1	0	0	0	0
1	1	0	1	0	0
1	1	0	0	0	0
1	1	0	1	0	0
1	1	0	1	0	0
1	1	0	0	0	0
0	1	1	0	0	0
1	1	1	0	0	0
1	1	0	0	0	0
0	1	0	2	0	0
0	1	0	1	0	0
1	0	0	0	0	0
0	1	0	0	0	0
0	0	0	0	0	0
0	1	0	1	0	0
0	1	0	1	0	0
1	1	1	0	0	0
0	1	0	0	0	0
1	1	0	1	0	0
1	1	0	0	0	0
1	1	0	0	0	0
0	0	1	0	0	0
1	1	1	0	0	1
1	0	0	0	0	0
0	1	0	0	0	0
1	1	0	1	0	0
1	0	0	1	0	0
1	1	0	0	0	0
1	1	0	1	0	0
0	1	0	0	0	0
0	1	0	1	0	0
1	0	0	1	0	0
1	1	0	2	0	0
1	0	0	1	0	0
1	1	0	2	0	0
1	0	0	0	0	0
1	0	0	1	0	0
1	1	0	1	0	0
1	1	0	1	0	0
1	1	0	2	0	0
0	0	0	0	0	0
1	1	0	2	0	1
1	1	0	1	0	0
1	1	0	2	0	0

1	0	0	2	0	0
1	1	0	1	0	0
1	1	0	0	0	0
1	1	0	1	0	0
1	1	0	0	0	0
1	1	0	0	0	0
0	0	0	0	0	0
1	1	0	1	0	0
1	1	0	1	0	0
1	1	0	1	0	0
1	1	0	2	0	0
1	1	0	1	0	0
1	1	0	1	0	0
0	0	0	0	0	0
1	0	0	1	0	0
1	1	0	1	0	0
0	1	1	2	1	0
0	1	0	0	0	0
0	1	0	0	0	0
1	1	0	2	0	0
0	1	0	2	0	0
0	0	0	0	0	0
1	1	1	1	0	1
0	1	0	1	0	0
1	1	1	0	0	0
0	1	0	0	0	0
1	1	0	2	0	0
1	1	0	0	0	1
1	1	0	1	0	0
0	1	0	1	0	0
1	1	0	1	0	0
1	1	0	2	0	0
0	1	0	1	0	0
1	1	0	0	0	0
1	1	0	0	0	0
1	1	1	2	0	0
0	1	1	1	0	0
1	1	1	1	0	0
1	1	0	0	0	0
0	1	0	1	0	0
1	1	0	1	0	0
1	1	0	2	0	1
0	1	0	0	0	0
1	1	0	0	0	0
1	1	1	1	0	0
1	1	0	1	0	2
0	1	0	1	1	0

1	1	0	1	0	0
0	1	0	2	0	0
1	1	0	1	0	0
0	1	0	0	0	0
0	1	0	1	0	0
1	1	1	2	0	0
0	1	0	1	0	0
1	1	0	1	1	0
1	1	0	1	0	0
1	1	0	1	1	0
1	1	0	1	0	0
0	1	0	0	0	0
0	1	0	0	0	0
1	0	1	1	0	0
0	1	1	0	0	0
1	1	1	1	0	0
1	1	0	1	0	0
0	0	1	0	0	0
0	1	0	0	0	0
1	1	0	0	0	0
0	1	0	1	0	0
1	1	1	2	1	0
0	1	0	0	0	0
0	0	0	0	0	0
1	1	0	0	0	0
0	1	0	0	0	0
1	1	0	1	0	0
0	1	0	0	0	0
0	0	1	1	0	0
0	1	0	0	0	0
0	1	0	0	0	0
0	1	0	0	0	0
1	0	0	1	0	0
0	0	0	0	0	0
0	0	0	0	0	0
0	1	0	0	0	0
1	1	0	2	0	2
0	1	0	0	1	0
0	1	0	0	1	0
0	0	0	0	0	0
0	0	0	0	0	0
0	1	0	0	0	0
1	1	0	0	0	0
1	1	0	2	0	0
1	1	0	2	0	0
1	1	0	2	0	0
1	1	0	1	0	0

1	1	1	1	0	0
1	1	1	0	0	1
1	1	0	1	0	0
1	1	0	0	0	0
1	1	0	2	0	0
1	1	0	0	0	0
1	1	0	2	0	0
0	0	0	1	0	0
1	1	1	0	0	0
0	0	0	0	0	0
1	1	0	1	0	0
0	1	0	0	0	1
1	0	0	0	0	0
0	1	0	0	0	0
1	1	1	0	0	0
1	1	0	1	1	0
1	1	0	1	0	0
0	1	0	1	1	0
1	1	0	1	0	0
1	1	0	1	0	0
1	1	1	0	0	0
0	1	0	2	0	2
1	1	0	0	0	0
1	1	0	2	0	0
1	1	0	1	0	0
1	1	0	2	0	0
1	1	1	2	0	0
1	1	1	1	0	0
1	0	0	1	0	0
1	1	0	0	0	0
1	1	0	0	0	0
0	0	0	1	0	0
1	1	0	1	0	0
1	1	0	1	0	0
1	1	0	1	0	0
1	0	0	1	0	0
1	1	1	2	0	0
1	1	0	1	0	0
0	1	0	0	0	0
1	1	0	1	0	0
1	1	0	2	0	0
1	1	0	0	0	0
1	1	0	1	0	0
1	0	0	2	0	0
1	0	0	2	0	0
1	1	0	1	0	0
1	1	0	0	0	0

0	1	0	0	0	0
1	1	0	2	0	1
0	1	0	2	0	0
1	1	0	2	0	0
1	1	0	0	0	0
1	1	0	2	0	0
1	1	0	1	0	0
0	1	0	1	0	0
1	1	0	0	0	0
1	1	0	0	0	0
0	1	0	0	0	0
0	1	0	0	0	0
1	1	0	2	0	0
1	0	0	1	0	0
1	1	0	0	0	0
1	1	1	0	0	0
1	1	1	0	0	0
1	1	0	2	0	0
1	0	1	1	0	0
1	1	0	0	0	0
1	1	0	1	0	0
1	1	0	0	0	0
1	1	0	2	0	0
1	1	0	1	0	0
0	1	0	1	0	0
1	1	0	1	0	1
0	1	0	0	0	0
1	1	0	1	0	0
1	1	0	2	0	0
1	1	0	0	0	0
1	1	0	1	0	1
1	1	0	1	0	0
1	1	0	1	0	0
1	1	0	0	0	0
1	1	0	0	0	0
1	1	0	1	0	0
0	1	0	0	0	0
0	1	0	0	0	1
0	1	0	0	0	0
0	1	0	1	0	0
